# Performance and safety of a multi-cancer early detection test: the PATHFINDER 2 study

**DOI:** 10.1038/s41591-026-04618-w

**Published:** 2026-09-22

**Authors:** Nima Nabavizadeh, Charles McDonnell, Dax Kurbegov, Marc Matrana, Shirish Gadgeel, Raymond H. Kim, Gretchen Stipec, Kevin C. Oeffinger, Michael J. Demeure, James W. Lillard, Rebecca Kaltman, Jennifer Tran, Alan Roth, Sami Tahhan, Andrew Poklepovic, Donald Richards, Margarita Lopatin, Celine Marquez, Margaret McCusker, Karthik V. Giridhar

**Affiliations:** 1 Knight Cancer Institute, Oregon Health & Science University, Portland, OR USA; 2 Sutter Health Research Institute, Sacramento, CA USA; 3 HCA Healthcare Sarah Cannon Cancer Network, Nashville, TN USA; 4 Ochsner Clinic Foundation, New Orleans, LA USA; 5 Henry Ford Health, Detroit, MI USA; 6 University Health Network, Princess Margaret Cancer Centre, Toronto, Ontario Canada; 7 Long Beach Memorial Medical Center, Long Beach, CA USA; 8 Duke University Health System, Durham, NC USA; 9 Hoag Memorial Hospital Presbyterian, Newport Beach, CA USA; 10 Morehouse School of Medicine, Atlanta, GA USA; 11 Inova Fairfax Hospital, Fairfax, VA USA; 12 MedStar Washington Hospital Center/Georgetown University School of Medicine, Washington, DC USA; 13 Jamaica Hospital Medical Center, Queens, NY USA; 14 Eastern Virginia Medical School at Old Dominion University, Norfolk, VA USA; 15 VCU Health Stony Point, Richmond, VA USA; 16 Texas Oncology, Tyler, TX USA; 17 GRAIL, Inc., Menlo Park, CA USA; 18 Mayo Clinic, Rochester, MN USA; 19 Present Address: Winship Cancer Institute, Emory University, Atlanta, GA USA

**Keywords:** Cancer screening, Cancer epigenetics

## Abstract

Early cancer detection improves survival; however, most cancer types do not have guideline-recommended screening. We previously reported the first PATHFINDER study that established feasibility of cancer screening with a blood-based multi-cancer early detection (MCED) test that predicts cancer signal origin (CSO) to guide diagnostic workup. The prospective, interventional PATHFINDER 2 study expands clinical evidence for the MCED test by evaluating co-primary endpoints of MCED performance metrics and safety in participants aged 50 years or older without clinical suspicion of cancer. Performance measures included cancer detection rate, positive and negative predictive values, episode sensitivity, specificity and CSO prediction accuracy. Safety was evaluated in terms of number and type of invasive procedures performed and adverse events due to diagnostic testing, after 12 months of follow-up. Endpoints were analyzed descriptively; no hypothesis testing was performed. Between December 2021 and July 2024, 35,878 participants were enrolled. Among 32,007 performance-analyzable participants, cancer detection rate was 0.54% (95% confidence interval (CI): 0.47−0.63%), positive predictive value was 60.3% (95% CI: 54.5−65.8%), negative predictive value was 99.2% (95% CI: 99.1−99.3%) and specificity was 99.64% (95% CI: 99.57−99.70%). Episode sensitivity was 39.3% (95% CI: 34.9−44.0%; all cancers) and 69.8% (95% CI: 62.8−76.0%; prespecified 12-cancer subgroup). Positive and negative likelihood ratios were 108.9 (95% CI: 87.6−135.2) and 0.61 (95% CI: 0.56−0.66), respectively, and the number needed to screen to detect one cancer was 185 (95% CI: 159−215). CSO prediction accuracy was 91.3%. Among 35,335 safety-analyzable participants, 213 (0.6%) had invasive procedure(s) after a positive MCED test; 90.5% were nonsurgical. No serious study-related adverse events were reported by the time of this analysis. These results provide insights into performance and safety of the MCED test in an intended-use population. Randomized trials and longer follow-up are needed to assess the clinical utility of MCED tests. ClinicalTrials.gov identifier: NCT05155605.

## Abstract (web summary)

The interventional PATHFINDER 2 study provides insights on the safety and performance of a blood-based multi-cancer early detection test in an intended-use population, including more than 35,000 participants aged 50 years or older.

## Main

Detecting cancer before metastasis improves survival^1^. Although effective screening improves early cancer detection, in the United States only 14% of all cancers are detected by guideline-recommended screening tests^2^. As such, more than 70% of cancer deaths occur from cancers for which no guideline-recommended screening exists, rising to more than 80% when accounting for real-world adherence rates.^3^

Blood-based MCED testing has emerged as a means of screening for multiple cancer types, including many that lack recommended screening, with a single test. The MCED test in this study analyzes methylation patterns on cell-free DNA (cfDNA) fragments from peripheral blood samples to detect a shared cancer signal and predict CSOs to guide diagnostic evaluations^4,5^. This MCED test has been validated in both case−control and interventional studies^4–7^, including the first PATHFINDER study that demonstrated feasibility of MCED screening^7^, and has been successfully implemented in several real-world clinical settings^8–11^.

PATHFINDER 2 (NCT05155605) aimed to expand clinical evidence for the MCED test in a large, diverse intended-use population. Here we report 12-month performance and safety of the MCED test version used during the study.

## Results

### Population

**Fig. 1:**
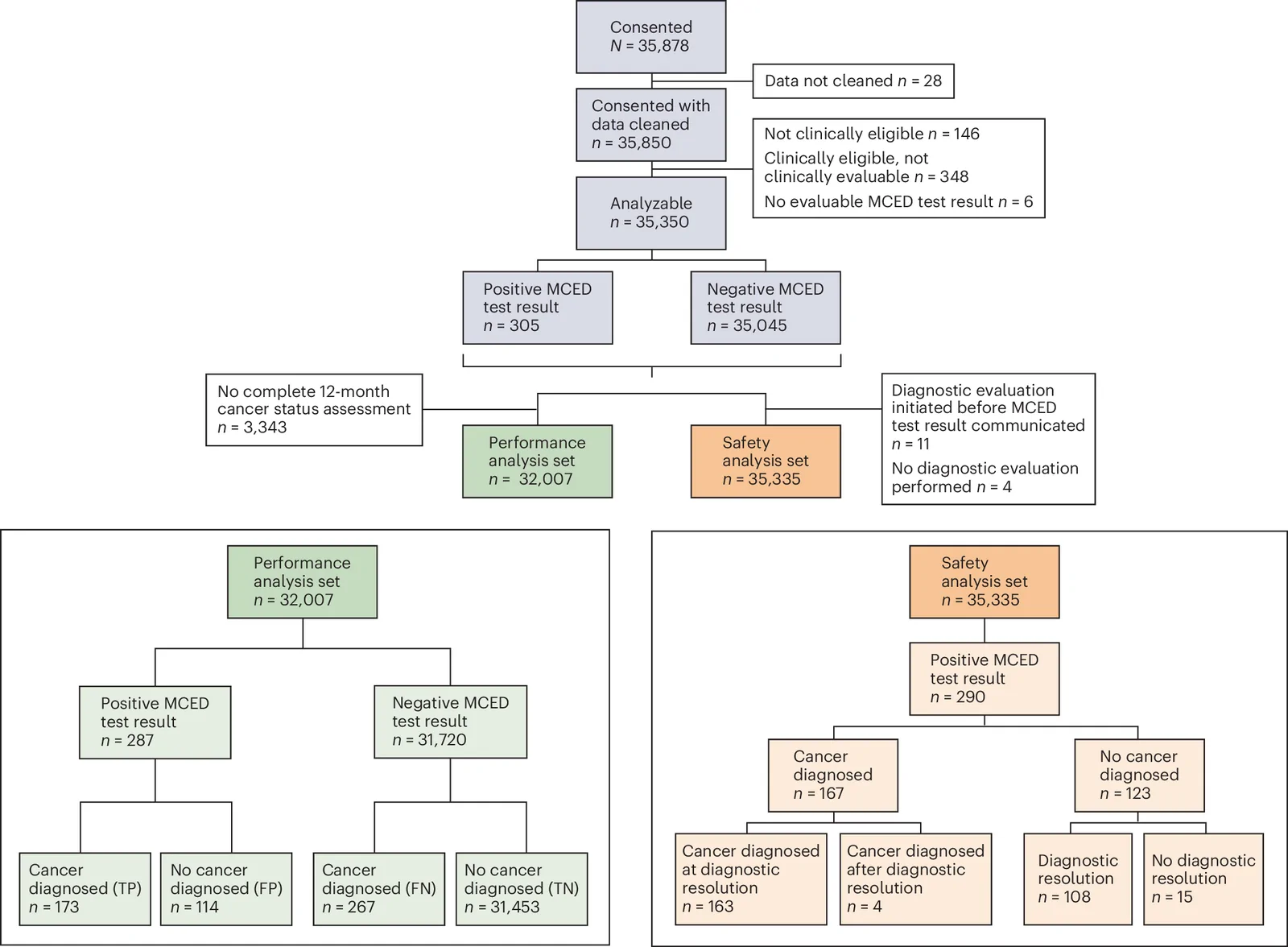
Participant flow diagram. Analyzable participants included 98.5% (35,350/35,878) of all consented participants. Participants with data not cleaned were those whose clinical data had not completed the predefined data cleaning and verification procedures specified in the study clinical data lock plan at the time of analysis. Participants who were not clinically eligible were those who consented but did not meet the study inclusion/exclusion criteria. Participants who were clinically eligible but not clinically evaluable were those who consented and met study inclusion/exclusion criteria but were not clinically evaluable (for example, did not complete blood draw or withdrew consent before blood draw). Analyzable participants were included in performance and/or safety analysis sets as indicated. These sets were not mutually exclusive. The performance analysis set included analyzable participants with a completed 12-month cancer status assessment. The safety analysis set included analyzable participants who had one or more diagnostic evaluations performed only after communication of their MCED test result. Diagnostic resolution was not required for inclusion. The performance analysis set insert illustrates the distribution of MCED test results: positive MCED test results were defined as true positive (TP) or false positive (FP) if a cancer was diagnosed or not diagnosed, respectively, within the 12-month follow-up period. Negative results were similarly defined as true negative (TN) or false negative (FN). The safety analysis set insert illustrates the distribution of participant groups after a positive MCED test result. All 15 participants excluded from the safety analysis set had positive MCED test results (therefore, 290 participants with a positive MCED test result were in the safety analysis set). Six of these 15 excluded participants were diagnosed with cancer, hence the difference in the number of participants with cancer diagnosed in the safety versus performance set (167 versus 173).

PATHFINDER 2 was a prospective, interventional study of an MCED test in participants aged 50 years or older without clinical suspicion of cancer who had not been diagnosed with or treated for cancer within 3 years prior to enrollment. A total of 35,878 participants were enrolled between 8 December 2021 (first participant in) and 10 July 2024 (last participant in). The data lock date for this analysis was 11 February 2026. Of the enrolled participants, 35,350 (98.5%) were analyzable (Fig. 1). Participant demographics were generally representative of US adults aged 50 years or older (Table 1). Participants were generally highly educated, and most were never-smokers. Most participants were up to date with United States Preventive Services Task Force (USPSTF) cancer screening recommendations at enrollment (Supplementary Table 1).

**Table 1:** Participant demographics and baseline characteristics

Characteristic	Analyzable participants *N* = 35,350
Median age (Q1, Q3), years	64.0 (58.0, 70.0)
Age groups, *n* (%), years	
50−59	10,053 (28.4)
60−69	15,549 (44.0)
70−79	8,764 (24.8)
≥80	984 (2.8)
Sex, *n* (%)	
Female	19,877 (56.2)
Male	15,473 (43.8)
Ethnicity (self-reported), *n* (%)	
Hispanic or Latino	2,632 (7.4)
Not Hispanic or Latino	32,274 (91.3)
Missing	444 (1.3)
Race (self-reported), *n* (%)	
American Indian or Alaska Native only	246 (0.7)
Asian only	2,091 (5.9)
Black or African American only	3,105 (8.8)
Native Hawaiian or other Pacific Islander only	80 (0.2)
White only	28,321 (80.1)
>1 race reported	474 (1.3)
Missing	1,033 (2.9)
Highest education level, *n* (%)	
Less than high school degree	842 (2.4)
High school degree or equivalent	2,778 (7.9)
Some college	7,535 (21.3)
Bachelor’s degree or higher	20,803 (58.8)
Missing	3,392 (9.6)
Smoking history, *n* (%)	
Never smoker	24,492 (69.3)
Former smoker	9,550 (27.0)
Current smoker	1,308 (3.7)
Alcohol use^a^, *n* (%)	
None	8,539 (24.2)
Light	15,493 (43.8)
Moderate	8,307 (23.5)
Heavy	2,407 (6.8)
Missing	604 (1.7)
BMI, *n* (%)	
<25 kg m^−^^2^	11,738 (33.2)
25 to <30 kg m^−^^2^	12,756 (36.1)
≥30 kg m^−^^2^	10,757 (30.4)
Missing	99 (0.3)
Prior cancer history^b^, *n* (%)	3,264 (9.2)
History of cancer in a first-degree relative^c^, *n* (%)	23,738 (67.2)
Genetic cancer predisposition, *n* (%)	2,047 (5.8)
Prior history of selected medical conditions, *n* (%)	
Autoimmune disease^d^	1,642 (4.6%)
Chronic liver disease^e^	2,004 (5.7%)
Chronic lung disease^f^	799 (2.3%)
Diabetes	4,138 (11.7%)
Nonmalignant gastrointestinal condition^g^	7,077 (20.0%)
Pancreatitis^h^	342 (1.0%)

^a^Over past 12 months. None is defined as 0 drinks per week; light is ≤3 drinks per week; moderate is >3 and ≤7 drinks per week for women and >3 and ≤14 drinks per week for men; heavy is >7 drinks per week for women and >14 drinks per week for men.

^b^Excluding nonmetastatic basal cell carcinoma and squamous cell carcinoma of the skin.

^c^Biologic parents, siblings or children.

^d^Multiple sclerosis, rheumatoid arthritis, systemic lupus erythematosus, Crohn’s disease, ulcerative colitis.

^e^Chronic hepatitis B infection, chronic hepatitis C infection, cirrhosis, fatty liver disease.

^f^Chronic obstructive pulmonary disease, emphysema, chronic bronchitis.

^g^Barrett’s esophagus, chronic gastritis, esophageal reflux disease, stomach ulcers.

^h^Acute pancreatitis, chronic pancreatitis.

### Primary objectives

The primary objectives were to evaluate the performance and safety of the MCED test, including diagnostic testing after a positive MCED test result. All study endpoints reported in this paper were designed to be descriptive; no prespecified success criteria or formal hypothesis testing were applied.

### Performance

**Extended Data Fig. 1:**
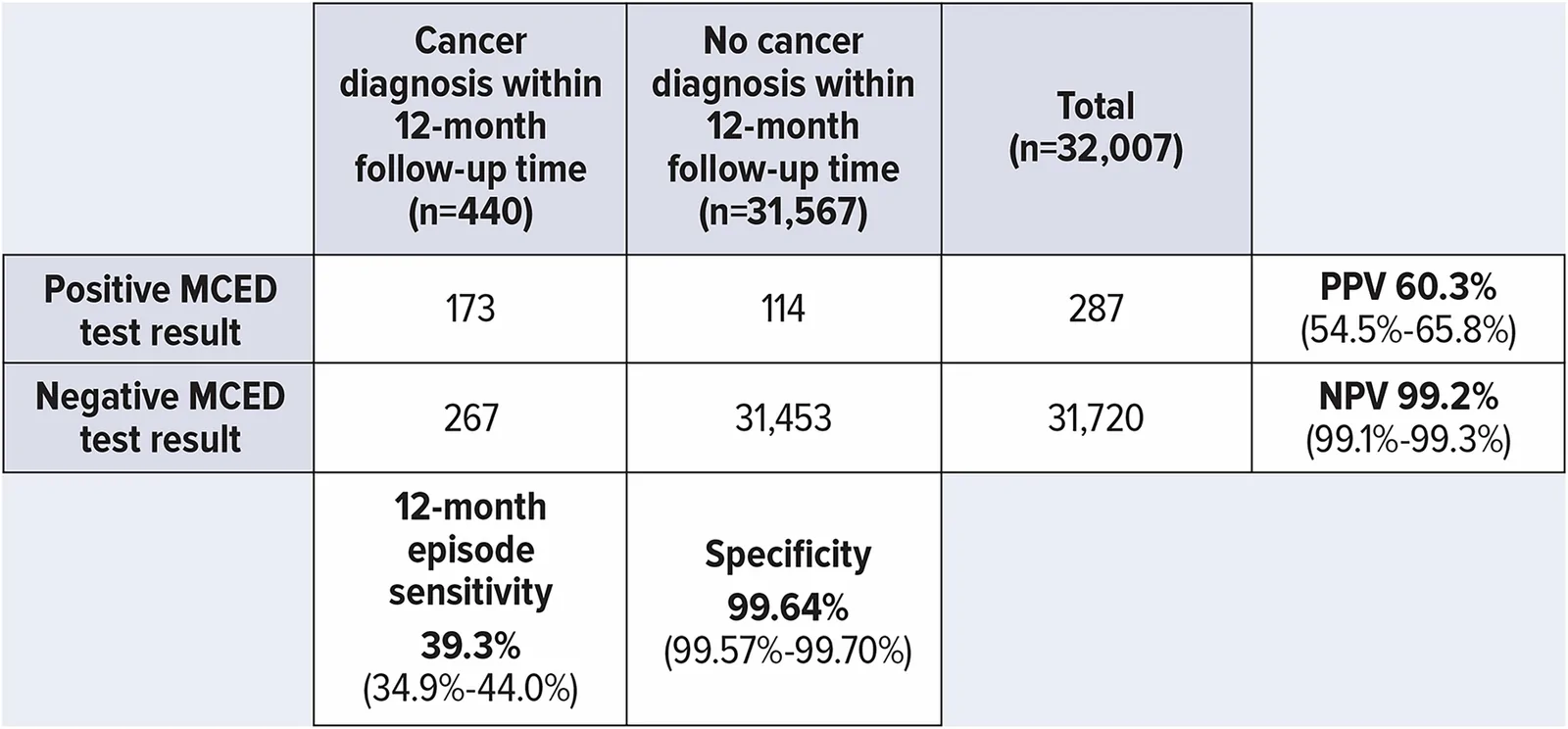
2×2 Contingency Table of Performance Metrics of the MCED Test. 95% CIs are included in parentheses for each performance metric. MCED, multi-cancer early detection; NPV, negative predictive value; PPV, positive predictive value.

**Extended Data Fig. 2:**
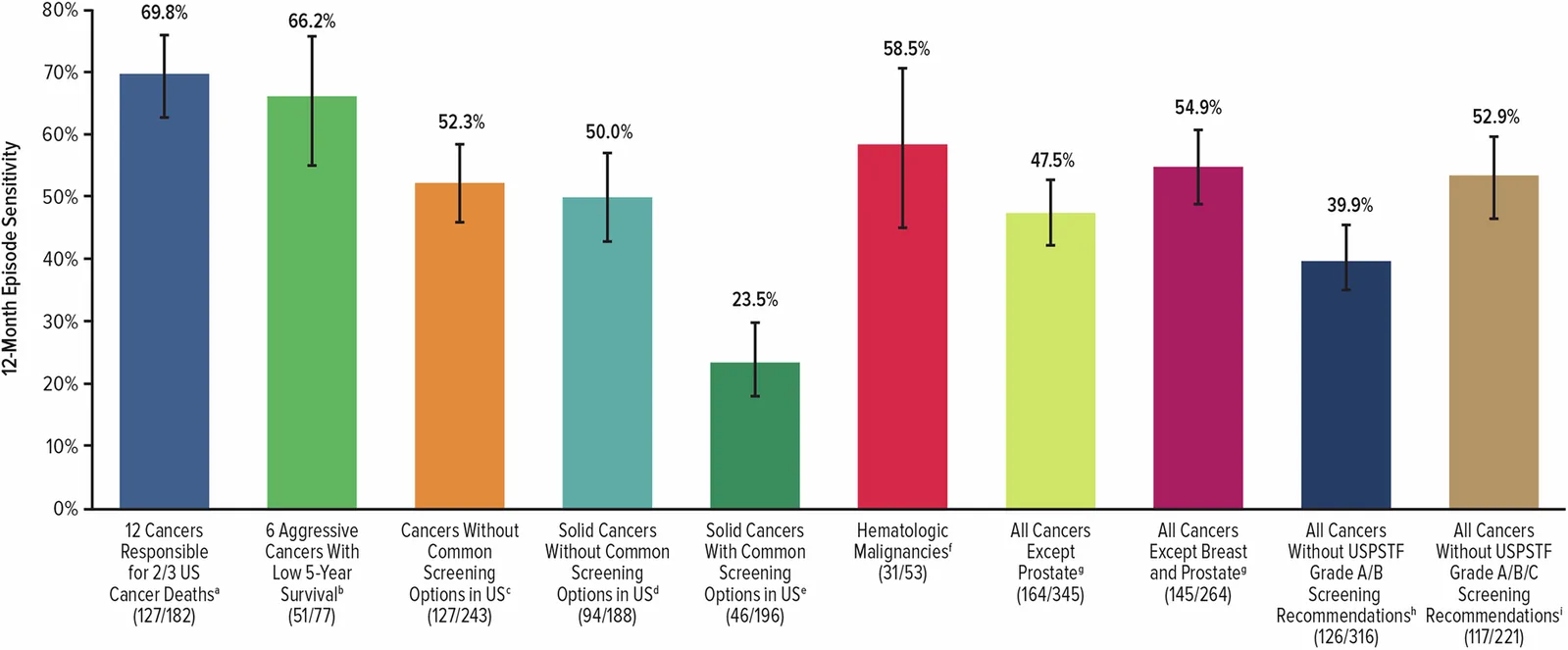
12-Month Episode Sensitivity in Clinically Relevant Cancer Subgroups. Data are presented as observed percentages. Error bars denote two-sided 95% CIs (Wilson score method). Participants with multiple cancers are included in a prespecified group only if all cancers belong to the group. ^a^Anus, Bladder/urothelial tract, Colon/rectum, Esophagus, Head & Neck, Liver/intrahepatic bile duct, Lung, Lymphoid lineage, Ovary/fallopian tube, Pancreas/extrahepatic bile duct/gallbladder, Plasma cell lineage, Stomach. ^b^Esophagus, Liver/intrahepatic bile duct, Lung, Ovary/fallopian tube, Pancreas/extrahepatic bile duct/gallbladder, Stomach. ^c^Anus, Bladder/urothelial tract, Bone/soft tissue sarcoma, Esophagus, Head & Neck, Kidney, Liver/intrahepatic bile duct, Lung, Lymphoid lineage, Myeloid lineage, Ovary/fallopian tube, Pancreas/extrahepatic bile duct/gallbladder, Plasma cell lineage, Skin, Stomach, Thyroid, Uterus, Other. ^d^Anus, Bladder/urothelial tract, Bone/soft tissue sarcoma, Esophagus, Head & Neck, Kidney, Liver/intrahepatic bile duct, Lung, Ovary/fallopian tube, Pancreas/extrahepatic bile duct/gallbladder, Skin, Stomach, Thyroid, Uterus, Other. ^e^Breast, Cervix, Colon/rectum, Prostate. ^f^Lymphoid lineage, Myeloid lineage, Plasma cell lineage. ^g^Post hoc analysis. ^h^Post hoc analysis, includes Anus, Bladder/urothelial tract, Bone/soft tissue sarcoma, Esophagus, Head & Neck, Kidney, Liver/intrahepatic bile duct, Lymphoid lineage, Myeloid lineage, Ovary/fallopian tube, Pancreas/extrahepatic bile duct/gallbladder, Plasma cell lineage, Prostate, Skin, Stomach, Thyroid, Uterus, Other. ^i^Post hoc analysis; includes Anus, Bladder/urothelial tract, Bone/soft tissue sarcoma, Esophagus, Head & Neck, Kidney, Liver/intrahepatic bile duct, Lymphoid lineage, Myeloid lineage, Ovary/fallopian tube, Pancreas/extrahepatic bile duct/gallbladder, Plasma cell lineage, Skin, Stomach, Thyroid, Uterus, Other.

**Extended Data Fig. 3:**
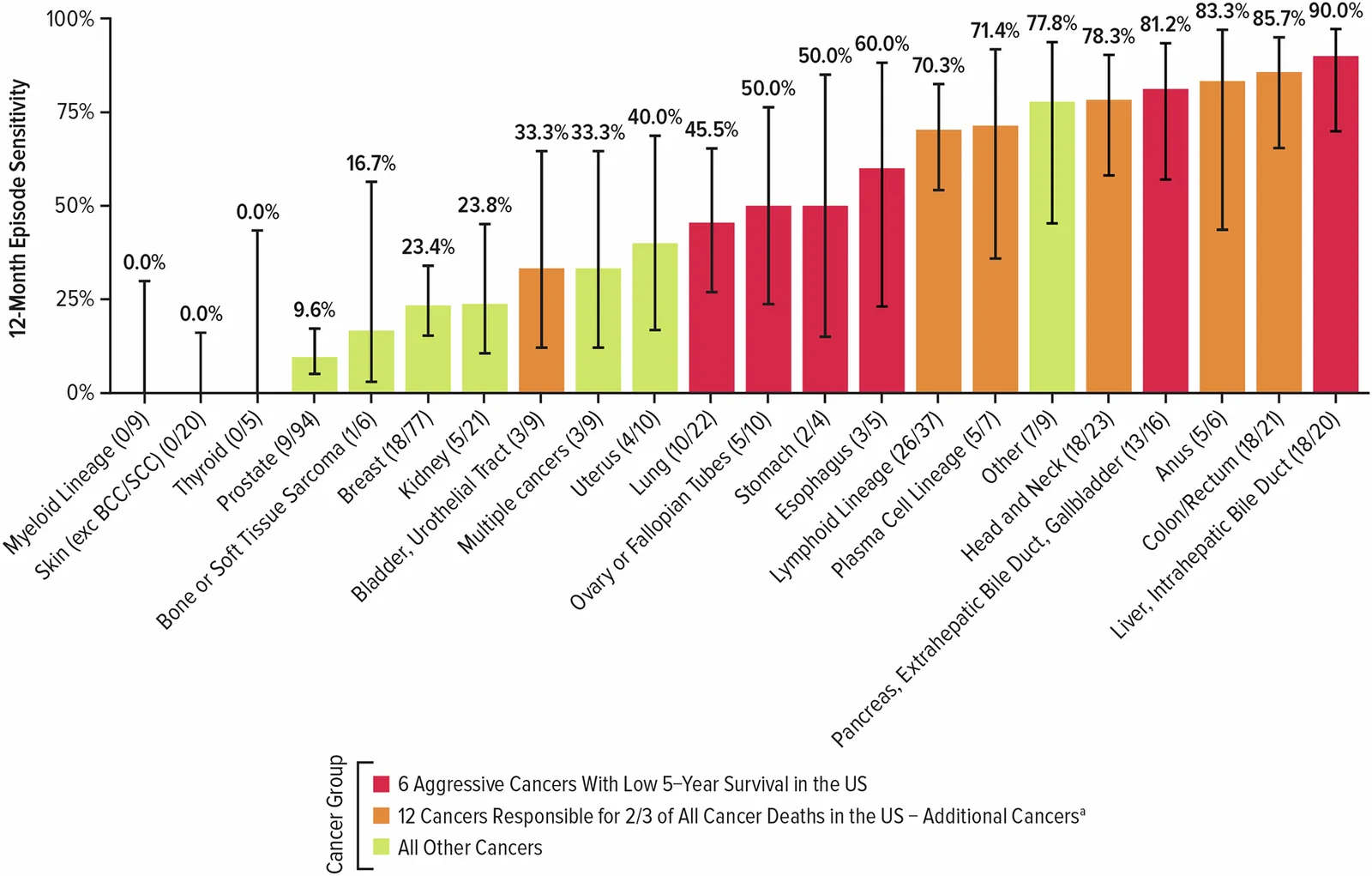
12-Month Episode Sensitivity by Cancer Type. Data are presented as observed percentages. Error bars denote two-sided 95% CIs (Wilson score method). ^a^In addition to the 6 aggressive cancers with low 5-year survival, which are all included in the 12 cancers responsible for two-thirds of US cancer deaths (see Table S22).

**Extended Data Fig. 4:**
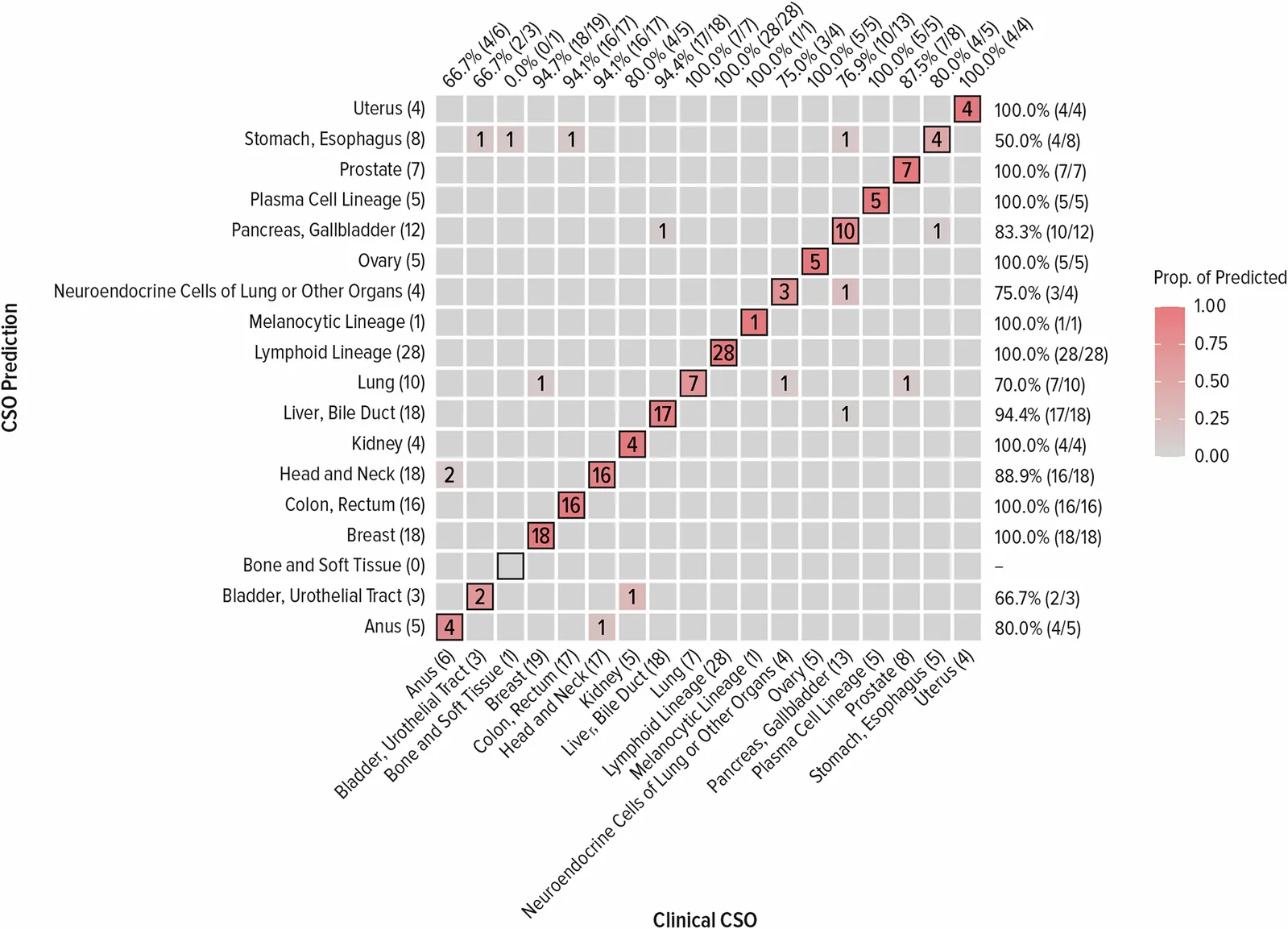
CSO Confusion Matrix. The number of participants for each combination of CSO prediction (y-axis) and clinical CSO (x-axis) is plotted in the confusion matrix. Correct CSO predictions are along the diagonal. The accuracy of each CSO prediction by clinical CSO (estimated by the proportion of the diagonal cell in each column) is indicated at the top of the matrix, and precision of each CSO prediction (estimated by the proportion of the diagonal cell in each row) along the right side. The color is associated with the proportions of matched predicted and clinical CSOs. The confusion matrix only includes participants with cancers with an assigned clinical CSO. The 7 participants with cancers without an assigned clinical CSO are not depicted here. CSO, cancer signal origin; Prop, proportion.

Of 35,350 analyzable participants, 32,007 (90.5%) had a completed 12-month cancer status assessment (Fig. 1). In this performance analysis set, 287 (0.9%) participants had a positive MCED test result (Table 2 and Extended Data Fig. 1). Of these, 173 had true-positive test results, for a cancer detection rate of 0.54%, positive predictive value (PPV) of 60.3% and negative predictive value (NPV) of 99.2%. Specificity was 99.64%. Twelve-month episode sensitivity was 39.3% across all cancers (Table 2) and higher for clinically relevant subgroups, including 69.8% in a prespecified subgroup of 12 cancers responsible for two-thirds of US cancer deaths^5^ and 66.2% in a prespecified subgroup of six aggressive cancers with low 5-year survival (Extended Data Fig. 2). Episode sensitivity varied by cancer type and was higher in more aggressive cancers (Extended Data Fig. 3). Positive and negative likelihood ratios were 108.9 and 0.61, respectively, and the number needed to screen to detect one cancer was 185 (Table 2). CSO1/CSO2 prediction accuracy was 91.3% and was largely consistent across cancer types (Extended Data Fig. 4).

**Table 2:** Performance of the MCED test

Metric	Performance analysis set^a^ *N* = 32,007
MCED test positive, *n*	
All	287
Cancer diagnosed (true positive (TP))	173
No cancer diagnosed (false positive (FP))	114
MCED test negative, *n*	
All	31,720
Cancer diagnosed (false negative (FN))	267
No cancer diagnosed (true negative (TN))	31,453
MCED cancer detection rate, % (95% CI) (TP / *N*)	0.54% (0.47−0.63%)
PPV, % (95% CI) (TP / (TP + FP))	60.3% (54.5−65.8%)
NPV, % (95% CI) (TN / (FN + TN))	99.2% (99.1−99.3%)
12-month episode sensitivity, % (95% CI) (TP / (TP + FN))	
All cancer types	39.3% (34.9−44.0%)
Prespecified subgroup of 12 cancers responsible for two-thirds of US cancer deaths^b^	69.8% (62.8−76.0%)
Prespecified subgroup of six aggressive cancers with low 5-year survival^c^	66.2% (55.1−75.8%)
Specificity, % (95% CI) (TN / (FP + TN))	99.64% (99.57−99.70%)
FP rate, % (95% CI) (FP / (FP + TN))	0.36% (0.30−0.43%)
Positive likelihood ratio, (95% CI) ((TP / (TP + FN))/ (FP / (FP + TN)))	108.9 (87.6−135.2)
Negative likelihood ratio, (95% CI) ((FN/(TP + FN)) / (TN / (FP + TN)))	0.61 (0.56−0.66)
Number needed to screen to detect one cancer, *n* (95% CI) (*N* / TP)	185 (159−215)
MCED cancer signal detection rate, % (95% CI) ((TP + FP) / N)	0.90% (0.80−1.01%)
CSO prediction accuracy^d^, % (95% CI)	
CSO1 prediction correct	87.3% (81.5−91.5%)
CSO1 or CSO2 prediction correct	91.3% (86.2−94.7%)

^a^The performance analysis set included analyzable participants with a completed 12-month cancer status assessment.

^b^Anus, bladder/urothelial tract, colon/rectum, esophagus, head and neck, liver/intrahepatic bile duct, lung, lymphoid lineage, ovary/fallopian tube, pancreas/extrahepatic bile duct/gallbladder, plasma cell lineage, stomach.

^c^Esophagus, liver/intrahepatic bile duct, lung, ovary/fallopian tube, pancreas/extrahepatic bile duct/gallbladder, stomach.

^d^Calculated out of participants with TP MCED test results (*n* = 173).

### Safety

Of 35,335 participants in the safety analysis set (Fig. 1), 290 had diagnostic evaluation(s), of whom 213 had a total of 295 invasive procedures after a positive MCED test result (Supplementary Tables 2 and 3). Prespecified safety measures (Table 3) were assessed as follows. Of all tested participants in the safety analysis set, the proportion of participants with an invasive procedure after a positive MCED test result was 0.6% (213/35,335), with 0.01 (295/35,335) invasive procedures per participant. Of participants with a positive MCED test result who underwent diagnostic evaluation, the proportion of participants with an invasive procedure after a positive MCED test result was 73.4% (213/290), with 1.02 (295/290) invasive procedures per participant. The median number of invasive procedures per participant with a positive MCED test result during diagnostic evaluation was 1.0 (Q1−Q3 0.0−1.0) (Supplementary Table 4). Most (1,694/1,989, 85.2%) evaluations performed were noninvasive (Supplementary Table 2). Invasive procedures were mainly nonsurgical (267/295, 90.5%).

**Table 3:** Safety measures during diagnostic evaluation (safety analysis set^a^)

Measure	Description	Outcome
A	Number of participants with a positive MCED test result and an invasive procedure divided by the total number of participants	0.6% (213/35,335)
B	Number of invasive procedures among all participants with a positive MCED test result by the total number of participants	0.01 (295/35,335)
C	Number of participants with a positive MCED test result and an invasive procedure divided by the total number of with a positive MCED test	73.4% (213/290)
D	Number of invasive procedures among participants with a positive MCED test result divided by the number of with a positive MCED test	1.02 (295/290)

^a^The safety analysis set included analyzable participants who had one or more diagnostic evaluations performed only after communication of their MCED test result.

**Fig. 2:**
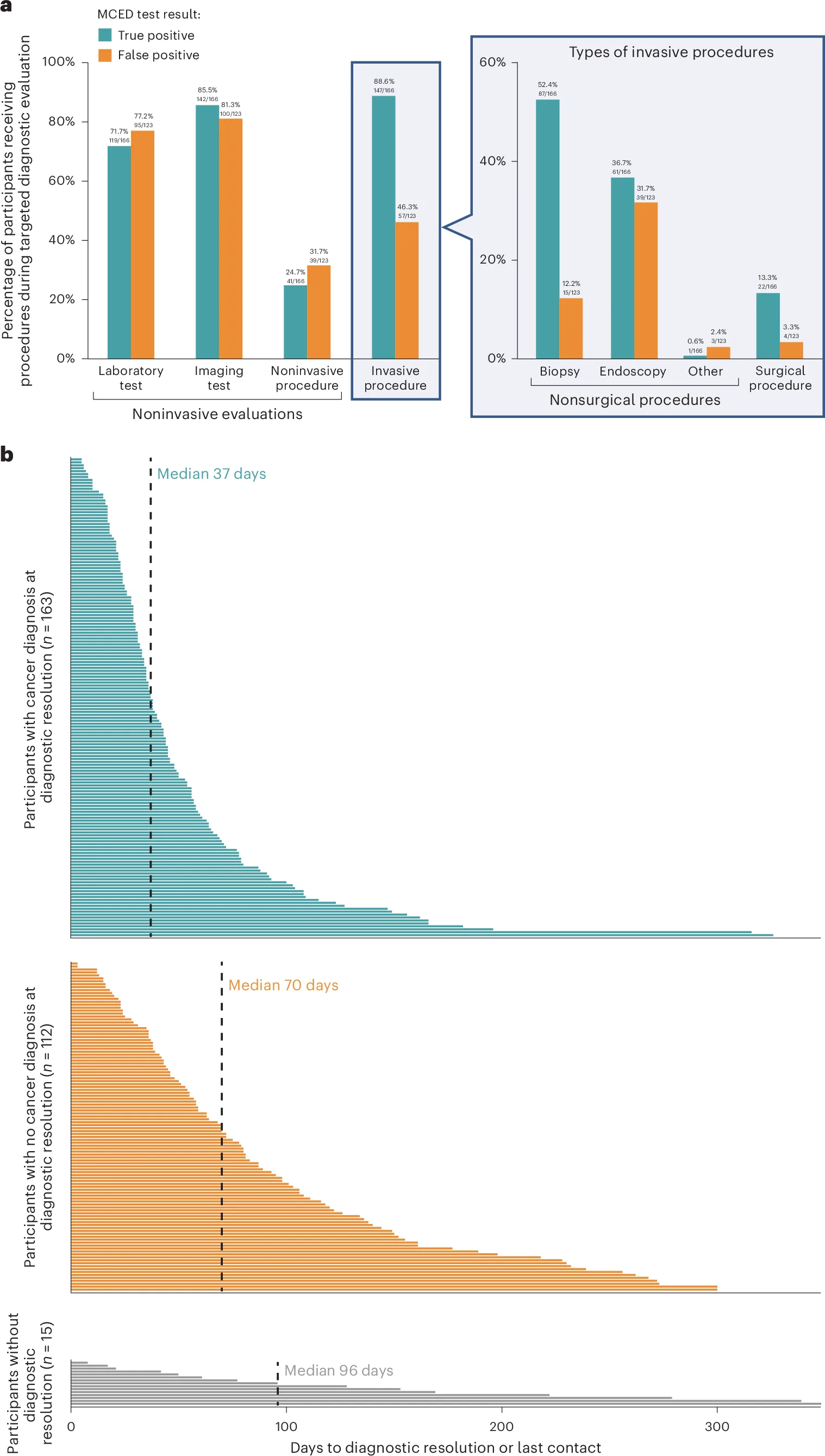
Targeted diagnostic evaluations and time to resolution in participants with positive MCED test results. **a**, Extent of diagnostic testing during targeted diagnostic evaluation, excluding any diagnostic PET/CT or diagnostic testing triggered by that diagnostic PET/CT, used to confirm cancer diagnosis after a negative targeted evaluation. Participants who did not achieve diagnostic resolution are included here as false positives. All four ‘Other’ nonsurgical procedures during targeted diagnostic evaluation were pap smears. All four participants with false-positive MCED test results and a surgical procedure had benign neoplasms diagnosed. **b**, Time to diagnostic resolution or date of last contact from communication of MCED test result. Here, participants with no cancer at diagnostic resolution who were later diagnosed with cancer within the 12-month follow-up time period (*n* = 4) were included in the ‘No cancer diagnosis at diagnostic resolution’ group. For participants who did not achieve diagnostic resolution within the 12-month follow-up time period, the last contact date (capped at 364 days from blood draw) was graphed and derived by prioritizing (1) the date of early discontinuation (for example, withdrawal or death); (2) any date mentioned in the reason for not achieving diagnostic resolution; and (3) the latest of the test result communication date and performed diagnostic procedure date. Black dashed lines represent the median time to diagnostic resolution in PATHFINDER 2.

**Extended Data Fig. 5:**
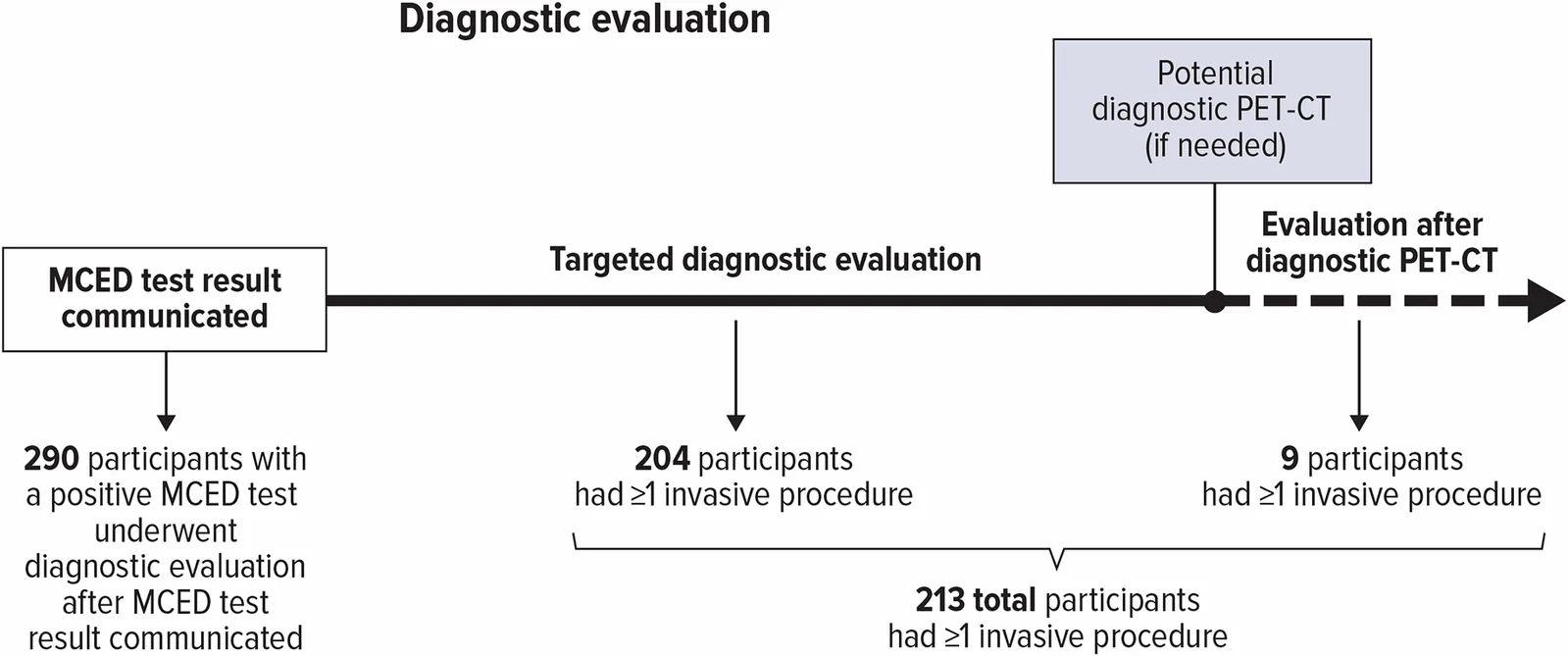
Use of Invasive Procedures During Phases of Diagnostic Evaluation Based on Use of Diagnostic PET-CT. The targeted diagnostic evaluation phase included all evaluations that occurred before a protocol-directed diagnostic PET-CT, if one was needed. Diagnostic PET-CT was performed in cases where cancer was not found during the CSO-guided targeted evaluation phase. There were 290 participants with a positive MCED test result with ≥1 diagnostic evaluation whose diagnostic evaluation was initiated after their positive MCED test result was communicated (safety analysis set criteria; see Fig. 1). In total, 213 participants had ≥1 invasive procedure performed at any point during diagnostic evaluation: 204 had invasive procedure(s) during targeted evaluation and 9 had invasive procedure(s) performed only after a diagnostic PET-CT. MCED, multi-cancer early detection; PET-CT, positron emission tomography–computed tomography.

Of the 213 participants with invasive procedures, 204 had one or more invasive procedures during targeted diagnostic evaluation (that is, evaluation before diagnostic positron emission tomography/computed tomography (PET/CT), if performed) (Extended Data Fig. 5 and Supplementary Table 3). Almost twice as many participants with true-positive versus false-positive MCED test results (88.6% versus 46.3%) had invasive procedures during targeted evaluation (Fig. 2a and Supplementary Table 5). Targeted evaluations were mainly CSO guided (254/290, 87.6%; Supplementary Table 6). Of 114 participants with false-positive MCED test results, 61 (53.5%) had a benign neoplasm and/or noncancer condition identified during evaluation (Supplementary Table 7). For the four participants with a false-positive MCED test result who had a surgical procedure (Supplementary Table 5), surgery was necessary to clear suspicion of cancer and uncovered benign neoplasms in all cases. Four of 290 (1.4%) participants were considered to have false-positive test results at the time of diagnostic resolution who were later diagnosed with cancer during the 12-month follow-up period (Fig. 1), of whom two of four had a cancer consistent with their predicted CSO(s).

During the time of diagnostic evaluation, nine of 290 (3.1%) participants experienced 10 adverse events. Five of the adverse events were not study related and included globus sensation, thrombocytopenia, anemia, increased creatinine and one unrelated serious adverse event of hyponatremia. The other five adverse events were study related and occurred only in those with a cancer diagnosis; none was serious (Supplementary Table 8). These included one emergency department visit for instruction on medication self-administration prior to a diagnostic workup procedure, one planned perioperative hospitalization to optimize pain control, one report of anxiety after receipt of a positive MCED test result, one report of mild postoperative pain and one report of low glomerular filtration rate due to nephrectomy. Among all consented participants with cleaned data at the time of this analysis (35,850 participants with clinical data verified per study data lock plan; Fig. 1), 56 (0.2%) experienced one or more study-related adverse events; none was serious, and most occurred in the phlebotomy setting (Supplementary Table 9).

### Secondary objectives

#### Performance in subgroups

Performance of the MCED test varied slightly by age group, sex and smoking status (Supplementary Tables 10−12). The cancer detection rate increased with age and was higher in male participants and participants with a smoking history. The ≥80 years age group had lower PPV, specificity and positive likelihood ratio than other age groups.

#### Use of diagnostic PET/CT

Of 63 participants who had PET/CT only after a negative targeted diagnostic evaluation, seven (11.1%) had cancer found (Supplementary Table 13). Of these cancers, five (8%) were diagnosed at the CSO1-predicted site and two (3%) had new diagnostic information uncovered by PET/CT.

#### Time to diagnostic resolution

Median (Q1−Q3) time to diagnostic resolution was 37 (24−61) and 70 (38−121) days for participants with and without cancer diagnosed at the time of diagnostic resolution, respectively (Fig. 2b). Median (95% CI) time to diagnostic resolution for all 290 participants based on Kaplan−Meier analysis was 48 (43−56) days.

#### Participant-reported outcomes

**Extended Data Fig. 6:**
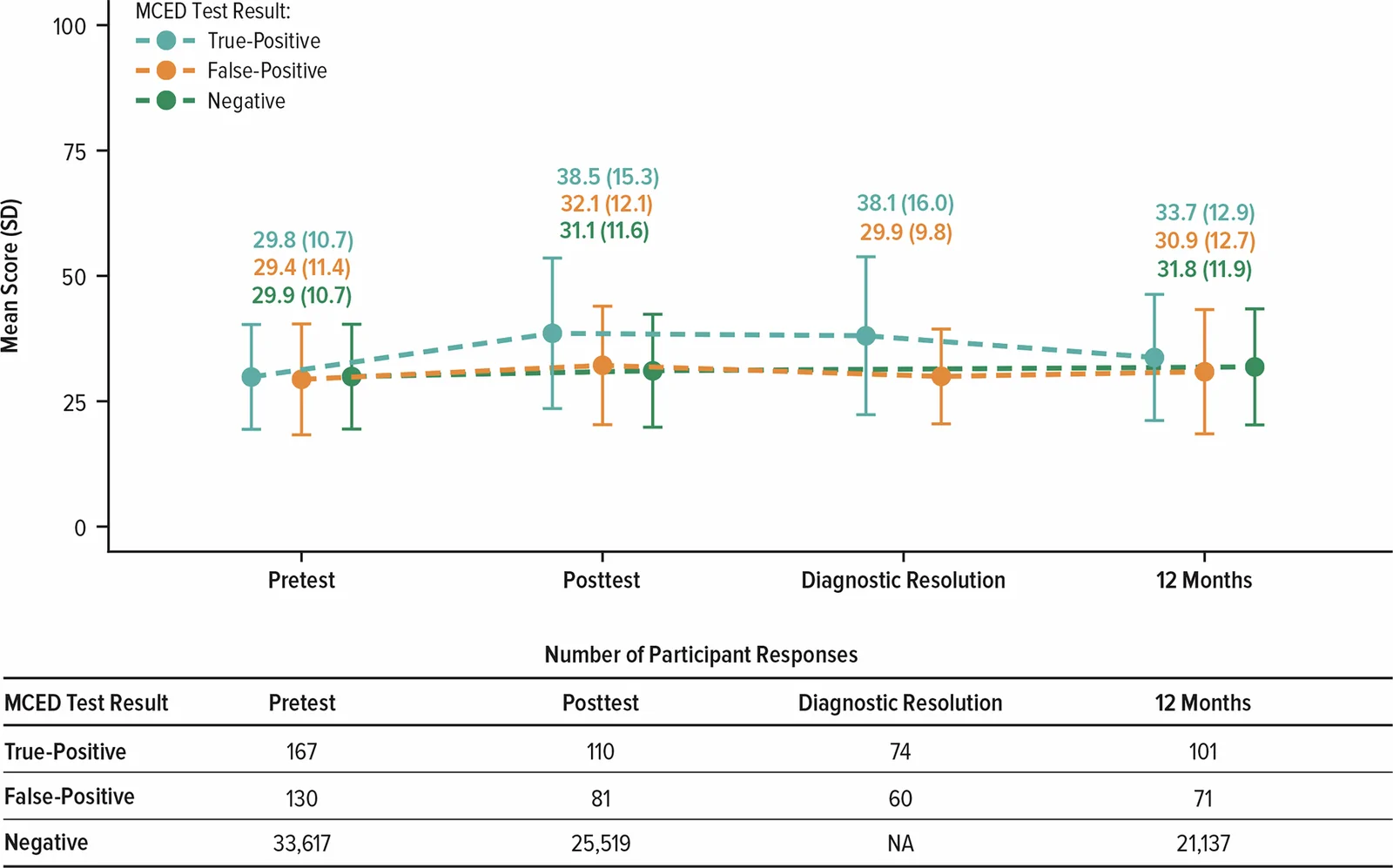
Participant-Reported Anxiety From Using the MCED Test. Data are presented as mean scores. Error bars denote standard deviation. Participant-reported anxiety was assessed by the STAI based on questionnaires completed ≤2 months (61 days) after questionnaire release for pretest, posttest, and diagnostic resolution time points and ≤3 months (91 days) after questionnaire release for the 12-month time point. Questionnaires at diagnostic resolution were administered only to participants with positive MCED test results. At the posttest timepoint, participants may have already begun diagnostic evaluation, with the results of those diagnostic tests influencing their score. For context, the STAI user manual indicates that mean scores were 32-34 (SD 8-10) for men and women aged 50-69 years; 42.38 (SD 13.79) for patients undergoing general, medical, and surgical procedures; and 49.02 (SD 11.67) for those with anxiety disorders^38^. MCED, multi-cancer early detection; NA, not applicable; STAI, State Trait Anxiety Inventory.

Participant-reported anxiety temporarily increased for participants with true-positive MCED test results, returning to baseline levels by 12 months (Extended Data Fig. 6). Anxiety did not increase for participants with either false-positive or false-negative test results.

### Descriptive summary of detected cancers

**Fig. 3:**
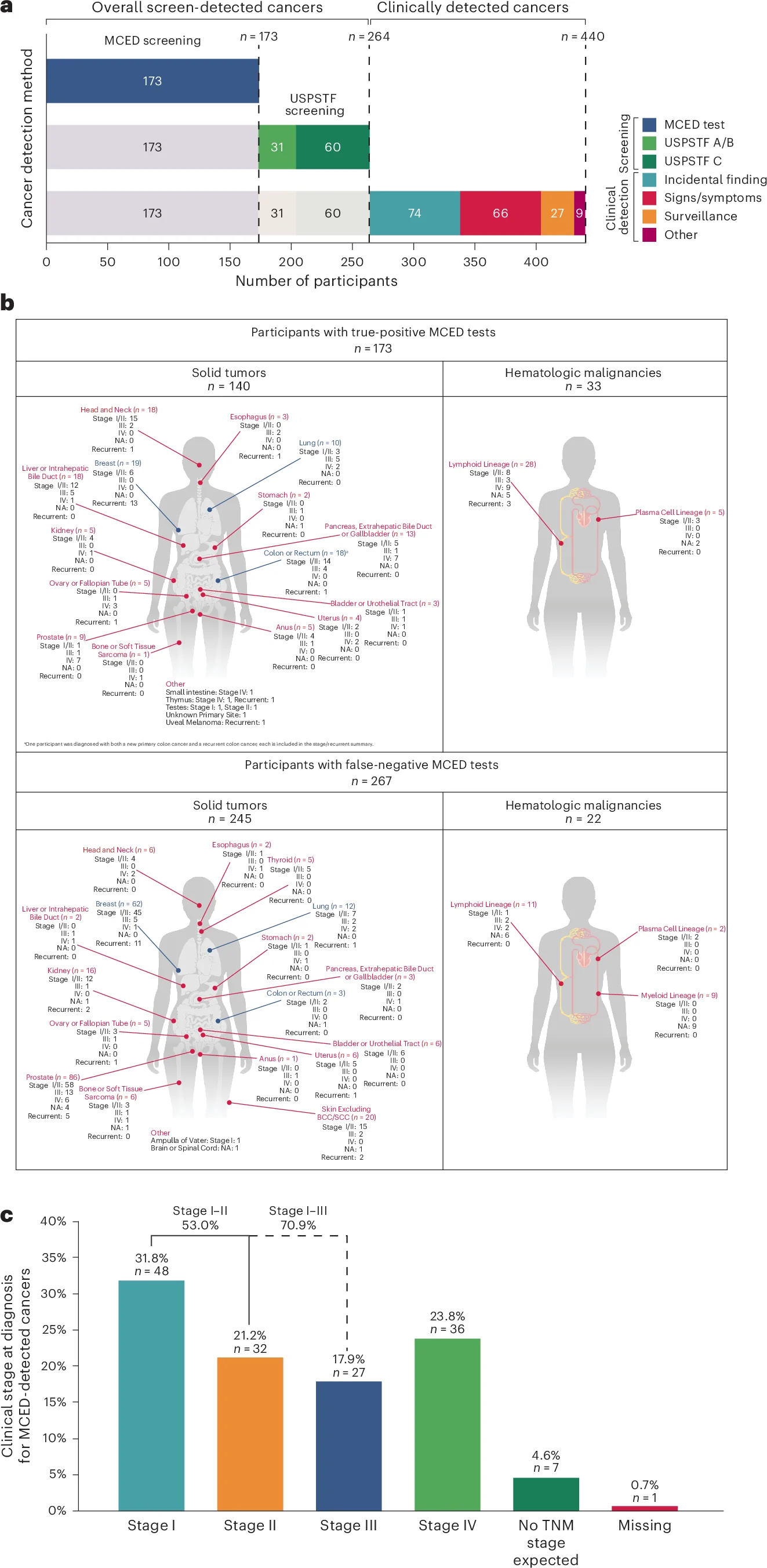
Cancer detection methods and location and stage of MCED-detected cancers. **a**, Method of detection for cancers diagnosed during the first 12 months of follow-up. For participants with multiple diagnosed cancers, only the method of detection for the first detected cancer is included. There is no overlap in cases detected by the MCED test and USPSTF-recommended screening tests because only participants with true-positive MCED test results were included in the ‘MCED screening’ group, whereas participants with negative MCED test results and a screen-detected cancer were included in the ‘USPSTF screening’ group. For the nine ‘Other’ clinically detected cancers, four were diagnosed based on follow-up of an abnormal test result, two were incidental findings during a nonsurgical procedure and three were other/unknown. **b**, Location and stage of MCED test-detected cancers for participants with true-positive and false-negative MCED test results. Cancers at locations colored blue have USPSTF grade A/B-recommended screening; cancers at locations colored red do not. Note that prostate cancer screening has a USPSTF grade C recommendation, meaning that screening is decided on an individual basis. Cancers not expected to be staged or with missing stage information are captured as ‘NA’ in the figure. For participants with multiple new primary cancers, only information from the first diagnosed cancer is included. The number of recurrent cancers listed captures local, regional and distant extent of disease spread (see Supplementary Table 17 for more detail). **c**, Clinical cancer stage at diagnosis for MCED test-detected cancers. For participants with multiple primary cancers, only information from the first diagnosed cancer was included. All seven cancers with no TNM stage expected were hematologic cancers: five lymphoid and two plasma cell neoplasms. The cancer with missing stage was a stomach cancer. BCC, basal cell carcinoma; NA, not available; SCC, squamous cell carcinoma; TNM, Tumor, Node and Metastasis.

In total, 440 participants were diagnosed with cancer during the 12-month follow-up period. Of these, 264 (60.0%) had cancer detected by any screening: 173 by MCED (that is, cancers in participants with true-positive MCED test results), 31 by USPSTF grade A/B-recommended screening and 60 by grade C screening (that is, USPSTF-detected cancers in participants with negative MCED test results) (Fig. 3a and Supplementary Table 14). Adding MCED testing to USPSTF grade A/B-recommended and A/B/C-recommended screenings increased the number of cancers detected by screening approximately 6.5-fold and approximately three-fold, respectively. There was no overlap in cases detected by the MCED test and USPSTF-recommended screening tests because only participants with true-positive MCED test results were counted as MCED detected, whereas participants with negative MCED test results and a screen-detected cancer during the 12-month follow-up period were counted as USPSTF screen detected. The remaining 176 (40.0%) cancers were clinically detected.

Among the 173 participants with MCED-detected cancers, 17 broad cancer types were represented (Fig. 3b), corresponding to 81 histologically distinct International Classification of Diseases for Oncology, 3rd Edition (ICD-O-3)-defined cancer types (Supplementary Table 15).

Of the 173 participants with MCED-detected cancers, 151 (87.3%) had one or more new primary cancers; 22 (12.7%) had a recurrent cancer (one participant had both a new and a recurrent cancer and, thus, is counted in both categories); and one had a cancer of unknown primary site (Supplementary Table 16). Of the first (if multiple) new primary cancers diagnosed, 80 of 151 (53.0%) were stage I−II and 107 of 151 (70.9%) were stage I−III (Fig. 3c and Supplementary Table 17). Within the aggressive cancer subgroups, 40.8−52.9% of MCED-detected cancers were stage I−II and 71.4−74.4% were stage I−III (Supplementary Table 18). Of recurrences detected by MCED, eight (36.4%) had local or regional spread (Supplementary Table 17).

Most (126/173, 72.8%) MCED-detected cancer types lack USPSTF grade A/B screening recommendations (Fig. 3b). Among MCED-detected early-stage cancers, 57 of 80 (71.3%) stage I−II and 75 of 107 (70.1%) stage I−III lack recommended screening.

Of 267 participants with false-negative MCED test results, 163 (61.0%) had cancer types with USPSTF grade A/B/C-recommended screening, including 103 (38.6%) stage I−II breast or prostate cancers (Supplementary Table 17). Over half (57/104, 54.8%) of false-negative cancers without grade A/B/C-recommended screening were stage I. Of 23 recurrent cancers among participants with false-negative MCED test results, most (16/23, 69.6%) were breast or prostate cancers.

### Post hoc analyses

Twelve-month episode sensitivity in additional subgroups was explored in post hoc analyses. Twelve-month episode sensitivity was 47.5% when prostate cancer was excluded and 54.9% when both breast and prostate cancers were excluded (Extended Data Fig. 2). Among cancer types without USPSTF grade A/B screening recommendations, 12-month episode sensitivity was 39.9%; among those without grade A/B/C recommendations, it was 52.9%.

## Discussion

PATHFINDER 2 provided evidence that the MCED test detected tumor-derived cfDNA in an intended-use population without clinical suspicion of cancer and demonstrated robust performance characteristics and a favorable safety profile. The high PPV (approximately two-fold to 20-fold higher than single-cancer screening tests^12–17^) and appropriate CSO-guided diagnostic evaluations resulted in a reasonable median time to diagnostic resolution, and the high specificity and corresponding low false-positive rate minimized unnecessary diagnostic workup. Most MCED-detected cancers were found at a stage when curative-intent therapy is feasible.

In PATHFINDER 2, participants’ cancer status was unknown at the time of MCED testing, and participants with a negative MCED test result did not undergo diagnostic evaluation to confirm absence of disease. Episode sensitivity was, therefore, assessed over a prespecified 12-month follow-up period, with any cancer diagnosed during that interval that was not detected by the MCED test classified as a false negative. Episode sensitivity is not equivalent to test sensitivity from case−control studies (where cancer status is known at the time of testing); these metrics should not be directly compared. For valid comparisons of sensitivity (and other performance metrics) observed for different tests/settings, standardization to a reference population is necessary, as sensitivity is highly influenced by the mix of cancer types in a population^18^. As an example, here we found that more than half the difference in episode sensitivity between all cancers (39.3%) and the prespecified 12-cancer subgroup (69.8%) was explained by the exclusion of breast and prostate cancers (54.9%), which highlights the influence that these two common cancer types have on sensitivity estimates.

The positive likelihood ratio exceeded 100, a magnitude rarely seen in clinical diagnostics (most have a positive likelihood ratio <10)^12,13,15,16,19–21^. This finding indicates that a positive MCED test result was strongly associated with the presence of a cancer, substantially increasing the post-test probability of disease. A positive likelihood ratio of this magnitude is informative to a clinician’s assessment of a patient’s cancer status and need for diagnostic evaluation for a potential cancer.

An accurate CSO prediction facilitates targeted diagnostic evaluation, minimizing unnecessary workups^22–24^. Reports from real-world clinical use of the MCED test show how CSO predictions effectively guide workups^8,9^. In PATHFINDER 2, there were few participants for whom diagnostic PET/CT yielded a cancer diagnosis after a negative targeted evaluation; for those who did, most had cancer consistent with the predicted CSO. With CSO-informed workups, median time to cancer diagnosis (for those with cancer) was much shorter than that found in a real-world retrospective claims analysis of more than 450,000 US patients with newly diagnosed cancer (37 days versus 118 days)^25^. Although the studies and populations were not directly analogous, this comparison provides some context for the duration of the diagnostic process for individuals diagnosed with cancer in the absence of a predicted cancer origin.

Safety concerns over MCED testing generally cite the potential harms associated with unnecessary diagnostic testing triggered by a false-positive MCED test result. We found these risks to be low. First, the MCED test demonstrated a low false-positive rate that strongly supports safe implementation of MCED testing in a screening population. Second, in participants with a positive MCED test result, most diagnostic evaluations were noninvasive; when invasive procedures were performed, their use was clinically justified given the CSO prediction. The rate of invasive procedures in MCED-tested participants observed in PATHFINDER 2 was 0.6%, lower than the approximately 2% and approximately 12.6% rate of biopsy after an abnormal mammogram or prostate-specific antigen test result, respectively^26–28^. Moreover, PATHFINDER 2 participants experienced few study-related adverse events, none of which was serious (although follow-up is ongoing, and any adverse events discovered through extended follow-up will be reported in future analyses); and the small rise in participant-reported anxiety for those with true-positive test results was temporary, consistent with experience observed for guideline-recommended screening tests^29,30^.

Most cancers missed by MCED were either stage I and/or had guideline-recommended screening available for early detection, suggesting that existing clinical detection or screening methods were sufficient in such cases. In turn, annual MCED screening could increase detection of cancers with low compliance to existing screening options (for example, lung cancer), cancers without existing screening options, interval cancers and recurrent cancers for which treatment was completed some years prior. Modeling analyses suggest that detection of more stage I−III cancers, which can largely be treated with curative intent, may reduce cancer mortality^31,32^.

Study strengths include a large cohort size from an intended-use population, minimal exclusion criteria and a representative participant population that supports the generalizability of these results^33,34^. Furthermore, test performance results were robust and consistent with previous studies^7,10,35^. Specificity was similar to the first PATHFINDER study (99.5%)^7^. PPV was higher than that observed in PATHFINDER (43.1%) and is in line with real-world PPV estimates reported from commercial use (49.4%)^10^ and from various health systems, such as Mayo Clinic (73.3%)^9^, Dana-Farber Cancer Institute (78.6%)^8^ and the Veterans Affairs Health System (50.0%)^35^. Importantly, test performance data should not be compared to estimates from observational or case−control studies of MCED tests due to the fundamental differences in study design.

Limitations include the following: a single-arm design with no comparator group; descriptive statistical endpoints with no defined success criteria or hypothesis testing; one-time MCED testing in a prevalence round where more late-stage cancers are expected than in subsequent incidence rounds; the fact that diagnostic testing costs were covered by the study sponsor such that the effect of real-world cost on workup decisions is unknown; and the use of an earlier version of the MCED test now replaced by a commercially available version with an updated CSO algorithm that predicts one CSO instead of two and provides supplemental biological information when relevant. Additionally, a higher percentage of participants reported characteristics associated with better health status compared to data from the United States National Health and Nutrition Examination Survey^34^, consistent with what has been observed in other cancer screening trials^36^. Although most eligible participants were up to date with USPSTF-recommended screening at enrollment, these data were missing for a sizable proportion of participants (especially for cervical and lung cancer screening), which may make adherence rates appear higher than they are in this population. The addition of MCED testing to existing screening programs represents a potential future scenario that will need to be supported by further clinical evidence, including clinical utility data, longitudinal testing data from multiple screening rounds and real-world implementation experience. Future analysis will evaluate the adherence of PATHFINDER 2 participants to guideline-recommended cancer screening over 3 years of follow-up after MCED testing. Future work will also examine the diagnostic journey for participants with positive MCED test results and intent of treatment (for example, curative versus palliative) for those diagnosed with cancer. In addition, the randomized controlled NHS-Galleri trial was designed to assess the clinical utility of MCED testing over 3 years of annual screening^37^ and will be reported separately.

PATHFINDER 2 provides useful insights into performance and safety of this MCED test in an intended-use population. The MCED test detected cancer signals in the blood of individuals without clinical suspicion of cancer at early stages when curative-intent therapy is possible. Randomized trials and longer follow-up are needed to establish the clinical utility of the MCED test in routine clinical practice.

## Methods

### Ethical approval and consent

This study was performed in accordance with the Declaration of Helsinki. All study procedures were approved by a central or local institutional review board (Supplementary Table 19). All participants provided written informed consent.

### Study design and participants

PATHFINDER 2 (NCT05155605) was a prospective, interventional study of the Galleri (GRAIL, Inc.) MCED test, enrolling adults aged 50 years or older from 32 clinical sites in the United States and Canada (Supplementary Table 19).

#### Inclusion criteria

- Aged 50 years or older, inclusive, at the time of signing the informed consent form.
- Capable of giving signed and legally effective informed consent, including compliance with the requirements and restrictions listed in the informed consent form and the protocol. Consent provided by a legally authorized representative was not permitted.

#### Exclusion criteria

- Undergoing or referred for diagnostic evaluation due to clinical suspicion of cancer (for example, referred to a medical or surgical oncologist or scheduled for biopsy on the basis of a suspicious imaging abnormality).
- Personal history of invasive solid tumor or hematologic malignancy diagnosed within the 3 years prior to expected enrollment date or diagnosed more than 3 years prior to expected enrollment date and never treated. Individuals with a diagnosis of nonmetastatic basal cell carcinoma or squamous cell carcinoma of the skin were not excluded.
- Prior or concurrent concomitant therapy: definitive treatment for invasive solid tumor or hematologic malignancy within the 3 years prior to expected enrollment date. Adjuvant hormone therapy for cancer (for example, for breast or prostate cancer) was not an exclusion criterion.
- Unable to comply with the protocol procedures.
- Not currently a registered patient at a participating center.
- Previous or current participation in another GRAIL-sponsored study, defined as having signed consent and provided a blood sample.
- Previous or current employee or contractor of GRAIL.
- Current pregnancy (by self-report of pregnancy status).

Participants were recruited at clinical sites via messages through the electronic health record, at health fairs and through promotional materials in hospitals and clinics. The study protocol and statistical analysis plan are provided in the Supplementary Information. Important protocol deviations are summarized in Supplementary Table 20.

### Procedures

At enrollment, participants provided a blood sample, from which plasma was isolated, cfDNA extracted and the MCED test performed as previously described^4,5,7^. MCED tests were ordered by and results returned to study investigators. The MCED test result was either negative (no cancer signal detected) or positive (cancer signal detected). Positive results included one or two CSO predictions (CSO1 and CSO2), with 21 possible predefined labels (Supplementary Table 21).

For participants with positive MCED test results, clinicians were strongly encouraged to conduct initial workups based on predicted CSO(s) and follow CSO-based targeted diagnostic evaluation recommendations described in the study protocol (Supplementary Table 21), investigating CSO1 first and then, if cancer was not found, CSO2 (if reported). If diagnostic resolution of cancer was not achieved from targeted evaluations, a diagnostic PET/CT was performed (if a PET/CT was not previously conducted during targeted evaluation; Extended Data Fig. 5). The date of diagnostic resolution for participants diagnosed with cancer was based on specimen collection date, date of imaging test or date of clinical investigation that confirmed the cancer diagnosis. A finding of no cancer was established if neither targeted diagnostic evaluation nor diagnostic PET/CT (and/or subsequent workup of abnormal findings) led to a cancer diagnosis. The date of diagnostic resolution for participants who were not diagnosed with cancer was based on the date of the last test or procedure (including diagnostic PET/CT, if performed) that was used to evaluate the positive MCED test result.

Participants were instructed not to interpret a negative MCED test result as absence of cancer and to continue to adhere to all guideline-recommended cancer screenings.

### Data collection

Data were collected by clinical site staff. Clinical data were collected from the blood collection tube, test requisition form, test result report, participants’ medical records and self-reported information and participant questionnaires. Clinical data were entered into and stored in the electronic case report forms within Medrio, a validated 21CFR Part 11-compliant electronic data capture system. Data collection was monitored by a contract research organization, PPD/Thermo Fisher Scientific. Categorization by demographic groups, including age, biological sex, ethnicity, race, education, smoking status, alcohol use and body mass index (BMI), was predefined in the statistical analysis plan. Race categories of American Indian or Alaska Native only (this category also included North American Indigenous to accommodate Canadian nomenclature from a clinical site in Canada), Asian only, Black or African American only, Native Hawaiian or other Pacific Islander only, White only or more than one race reported were aligned with US Food and Drug Administration (FDA) guidance. Race and ethnicity identities were determined by the participants.

### Clinical outcomes

Cancer was defined as a diagnosis of an invasive solid tumor, excluding nonmetastatic basal cell carcinoma and squamous cell carcinoma of the skin or hematologic malignancy (lymphoma, lymphoid leukemia, plasma cell neoplasm/multiple myeloma, myeloid neoplasm and additional malignant hematologic conditions with behavior code 3 based on ICD-O-3). ICD-O-3 was used as a reference system for what is considered to be a hematologic malignancy. Documentation of the ICD-O-3 behavior code was not required to establish a diagnosis of cancer. Cancer diagnosis was supported by pathologic confirmation of an invasive solid tumor or hematologic malignancy, by imaging confirmation in the absence of pathology, by assigned clinical stage of I−IV by the treating clinician or by other clinical confirmation of disease per treating clinician (for example, biochemical evidence of recurrence of prior cancer).

For stageable cancer types, clinical stage was determined by clinicians through clinical information, pathology and/or imaging for new primary cancers. Recurrences were documented as local, regional (lymph nodes) or distant (metastatic). A cancer type and clinical CSO (that is, as determined by clinical diagnosis) were assigned to each diagnosed cancer. Cancer types not included in the defined CSO list, typically less common cancers, were reported as ‘Other’ and were considered as ‘cancer types without clinical CSO’. Cancers of unknown primary site were not assigned a clinical CSO. Diagnosed cancers were also classified based on ICD-O-3 site and histology codes.

Cancer status assessment on all participants was performed at 1 year and is expected to be performed at 2 years and 3 years (±30 days) after enrollment. Assessment of 12-month cancer status was considered complete (that is, no loss to follow-up) when either (1) a participant’s cancer diagnosis date was within the first 12 months of follow-up or (2) no cancer diagnosis was documented within that time period based on electronic medical records or direct contact with participants. Participants with incomplete cancer status assessment at 12 months were those with no cancer diagnosis reported at the time of the last medical record review/direct contact and whose last medical record review/direct contact occurred fewer than 11 months after enrollment.

The detection method for cancers diagnosed during the 12-month follow-up period was recorded according to the groups outlined in Supplementary Table 14.

Participants continue to be followed for a total of 3 years after enrollment to assess cancer status and utilization of guideline-recommended cancer screening on an annual basis.

### Analysis sets

Analysis sets were prespecified in the statistical analysis plan. The analyzable set comprised consented, clinically eligible and clinically evaluable participants with an evaluable MCED test result (Fig. 1). The performance analysis set included analyzable participants with a completed 12-month cancer status assessment. The safety analysis set included analyzable participants who had one or more diagnostic evaluations performed only after communication of their MCED test result.

### Study objectives and endpoints

Primary objectives were performance of the MCED test and its safety in terms of diagnostic testing triggered by the MCED test result (Supplementary Table 23). The primary performance endpoints included PPV, NPV, sensitivity, specificity, false-positive rate, positive likelihood ratio, negative likelihood ratio, overall CSO accuracy, observed cancer detection rate, number needed to screen to detect a cancer, MCED cancer detection rate, MCED cancer signal detection rate, accuracy of a CSO prediction by clinical CSO and precision of a CSO prediction by CSO prediction. The primary safety endpoint included the number and type of invasive procedures performed and the number and type of adverse events due to diagnostic testing in all participants with a positive MCED test result (safety measures detailed in Table 3).

All secondary endpoints are described in Supplementary Table 23. Secondary endpoints reported here included participant-reported anxiety assessed by State-Trait Anxiety Inventory (STAI) questionnaires pre-test (baseline), post-test, at diagnostic resolution (if applicable) and at 1 year (+30 days); utilization of standard-of-care cancer screening tests prior to study enrollment; the proportion of participants with cancer diagnosis at diagnostic resolution out of all participants who had a PET/CT only after initial negative diagnostic evaluation (no PET/CT during initial diagnostic evaluation); the proportion of participants with cancer diagnosis at diagnostic resolution out of all participants with initial negative diagnostic evaluation who had a diagnostic PET/CT at any time during diagnostic evaluation; the number and type of imaging procedures, number and type of invasive procedures, number and type of laboratory tests and time to diagnostic resolution; and primary test performance endpoints assessed in selected demographic and clinical subgroups classified on the basis of different categories of age, sex and smoking history.

Secondary endpoints not reported here include intention to follow standard-of-care cancer screening tests assessed pre-test, post-test and at 1 year (+30 days) and utilization of standard-of-care cancer screening tests during the first year and the second year (±30 days) after MCED test; the number and type of imaging procedures with radiation exposure and total per-participant radiation exposure during diagnostic evaluation; concordance between initial MCED test results and research blood draw results, including agreement in cancer signal detection and concordance of CSO1 prediction among participants with both test results; participant-reported outcomes and perceptions of the MCED test, assessed using participant-directed questionnaires, including health-related quality of life and other questionnaire-specific scores or item responses; primary test performance endpoints assessed in selected demographic and clinical subgroups classified on the basis of different categories of race, ethnicity, BMI, prior cancer history and genetic cancer predisposition; and MCED test positive rate among participants with potentially cross-reactive conditions.

All study objectives and endpoints were prespecified in the protocol and statistical analysis plan. The statistical analysis plan included analyses for two test versions: (1) MCED-V2, the test version used in the study for which results were returned to healthcare providers to inform diagnostic evaluations (that is, the study protocol version) and (2) a different MCED test version (referred to as MCED-I in the statistical analysis plan) that was analyzed to support an FDA premarket approval (PMA) submission. The results of the MCED-I test version were not returned during the PATHFINDER 2 study and, therefore, did not inform participants’ diagnostic evaluations. The analysis plan for MCED-I included composite success criteria and hypothesis testing (detailed in statistical analysis plan section 14.2.2.2), which were specific to the PMA analysis and, thus, were not included in the study protocol. The protocol and the present paper were focused on evaluating the safety and performance of MCED-V2. These analyses were designed to be descriptive, with no defined success criteria or formal hypothesis testing. Thus, the PMA-specific success criteria and hypothesis testing specified for MCED-I are not applicable to the MCED-V2 12-month analyses reported in this paper.

### Performance

Performance of the MCED test, evaluated in analyzable participants with a completed 12-month cancer status assessment (performance analysis set), was assessed by multiple endpoints (see Glossary of Screening Terms for definitions and calculations). CSO prediction accuracy was calculated based on comparison of predicted CSO(s) with clinical CSO among participants with true-positive MCED test results. Twelve-month episode sensitivity was assessed by cancer type and within predefined cancer subgroups (Supplementary Table 22).

### Safety

The safety analysis focused on analyzable participants with one or more diagnostic evaluations initiated only after MCED test result communication. Primary safety analyses evaluated the number and type of invasive procedures performed and adverse events occurring during the time of diagnostic testing triggered by a positive MCED test result. Invasive procedures were surgical or nonsurgical (for example, endoscopy and biopsy). Secondary analyses described laboratory tests, imaging, noninvasive procedures and adverse events in all consented participants with cleaned data.

Time to diagnostic resolution was calculated from the time the MCED test result was communicated to the participant or to the date of last contact for participants who did not achieve diagnostic resolution.

### Participant-reported outcomes

Participant-reported anxiety resulting from MCED test use was assessed with the STAI^38^ based on questionnaires completed ≤2 months (61 days) after questionnaire release for pre-test, post-test and diagnostic resolution timepoints and ≤3 months (91 days) after questionnaire release for the 12-month timepoint. Questionnaires at the time of diagnostic resolution were administered only to participants with positive MCED test results.

### Cancer detection method

The fold change in screen-detected cancers with the addition of MCED testing was calculated as follows: (number cancers detected by MCED screening + number of cancers detected by USPSTF-recommended screening) / number of cancers detected by USPSTF-recommended screening. MCED-detected cancers were defined as cancers in participants with true-positive MCED test results. There was no overlap in cases detected by the MCED test and USPSTF-recommended screening tests because only participants with true-positive MCED test results were counted as MCED detected, whereas participants with negative MCED test results and a screen-detected cancer during the 12-month follow-up period were counted as USPSTF screen detected.

### Sample size and power

The sample size of this descriptive study was driven by the ability to enroll participants in the specific study subgroups based on age and sex to obtain information on diagnostic evaluation triggered by the MCED test result. The study was designed to enroll greater than or equal to 35,000 participants. Assuming that approximately 5% of participants would be excluded due to various clinical and assay evaluability reasons, approximately 33,250 analyzable participants were expected for analyses related to receiving MCED test results and associated diagnostic evaluation. Based on previous studies, MCED test specificity was expected to be in the range of 99−99.5%^7^.

A co-primary analysis objective focused on MCED test performance. The expected number of positive MCED test results, number of cancers diagnosed and test performance endpoints were estimated using microsimulations as previously described^39^. Under conservative assumptions about cancer incidence used in these microsimulations to account for a possible healthy volunteer effect (assuming underlying cancer incidence in the enrolled population was 65% of Surveillance, Epidemiology, and End Results (SEER) cancer incidence with the sex and age distribution described in the study protocol), approximately 370 cancers were expected to be diagnosed among 35,000 enrolled participants (or 33,250 analyzable participants) during 12 months of follow-up. The results of the microsimulations indicated that approximately 300−462 positive MCED test results were expected, with approximately 139 true-positive and 163−323 false-positive MCED test results. Based on these results, we expected to observe a PPV of approximately 30% and 46% if MCED test specificity is 99% and 99.5%, respectively. To illustrate the precision around these point estimates, an observed PPV of 30% based on 462 positive MCED test results would yield a two-sided Wilson 95% CI of 26.1−34.4%, and an observed PPV of 46% based on 300 positive MCED test results would yield a two-sided Wilson 95% CI of 40.4−51.7%. The other co-primary analysis objective focused on safety evaluation. The levels of precision for the primary safety Measure A (‘number of all participants with MCED positive test results and an invasive procedure divided by the total number of analyzable participants’) were estimated under six scenarios representing levels of false-positive rate (1.0% or 0.5%) and proportions of participants with invasive procedures ranging from 10% to 50%. Approximately 80% of participants with true-positive and 30% with false-positive MCED test results were estimated to have an invasive procedure based on data from the PATHFINDER study^7^. Based on the false-positive rate and the proportion of participants with false-positive MCED test results and invasive procedures, Measure A was estimated to range from approximately 0.4% to 0.8% across these scenarios. For reference, the proportion of participants with false-positive test results and invasive procedures among all screened participants for existing guideline-recommended noninvasive screening tests has been estimated as approximately 1.66% for breast cancer screening with mammography, 10.2% for colorectal cancer screening with a multi-target stool DNA test and 1.88% for lung cancer screening with low-dose CT^13,40,41^. The expected one-sided 95% upper confidence bound for Measure A based on all participants with positive MCED test results ranged from 0.45% to 0.92% across the scenarios prespecified in the protocol and was substantially lower than the reported point estimates for these existing screening tests among false-positive results. No formal comparison with existing screening tests was planned; for illustrative purposes only, the study would provide a high power (>99%) for such a comparison with even the lowest estimate reported above (1.66% for screening mammography) used as a threshold—that is, to test a null hypothesis that Measure A is above 1.66%.

### Statistical analysis

No formal hypothesis testing or success criteria were defined for the endpoints reported in this paper; all endpoints were prespecified and analyzed descriptively. Based on microsimulations with conservative assumptions, greater than or equal to 370 cancers were expected with 35,000 participants screened, including 156 with true-positive MCED test results, which was considered sufficient for descriptive analyses of test performance and safety. Data lock date was 11 February 2026.

Performance estimates were not calculated for subgroups with fewer than five participants. Two-sided 95% CIs were constructed using the Wilson (score) method for performance endpoints not very near 0 or 1. Modified Wilson (score) CIs were used for endpoints near 0 or 1.

For categorical/binary variables, number and percentage of participants in each category were described. For continuous variables, number of participants and median with first and third quartiles (Q1, Q3) were reported.

The Kaplan−Meier method was used to estimate time to diagnostic resolution for participants with positive MCED tests.

Analyses were conducted using software R version 4.3.2.

### Protocol and statistical analysis plan amendments

A complete list of amendments to the PATHFINDER 2 study protocol and statistical analysis plan is provided in Supplementary Table 24. Both documents are provided in full in the Supplementary Information.

### Reporting summary

Further information on research design is available in the Nature Portfolio Reporting Summary linked to this article.

## Online content

Any methods, additional references, Nature Portfolio reporting summaries, source data, extended data, supplementary information, acknowledgements, peer review information; details of author contributions and competing interests; and statements of data and code availability are available at https://doi.org/10.1038/s41591-026-04618-w.

## Supplementary information

Supplementary Information Glossary of Screening Terms, Tables 1−24, protocol and statistical analysis plan.

Reporting Summary

## Data Availability

Anonymized participant data will be available after the completion of the 3-year follow-up and database lock (anticipated December 2027). Requests for access to anonymized data may be emailed with a proposal to nabaviza@ohsu.edu. Investigators will assess proposals for scientific merit. Proposals should have a clearly specified research question supported by a methodologically sound analysis proposal and a prespecified statistical analysis plan, together with documentation of the requesting investigators’ qualifications and any applicable ethics and data protection approvals. Data will be provided after approval of the proposal and completion of a data transfer agreement. Decisions regarding the proposals will be communicated within 4−6 weeks.
