# Supplementary material for: Performance and safety of a multi-cancer early detection test: the PATHFINDER 2 study
Source: Nat Med. 2026 Sep 22;32(10):3760–9. doi: 10.1038/s41591-026-04618-w (PMC13668646; doi:10.1038/s41591-026-04618-w)
Supplement: Supplementary file 1 [file 41591_2026_4618_MOESM1_ESM.pdf]

nature medicine

Supplementary information https://doi.org/10.1038/s41591-026-04618-w

Performance and safety of a multi-cancer early detection test: the PATHFINDER 2 study

In the format provided by the authors and unedited

Supplemental Information

Table of Contents

Supplemental Information **1**

Glossary of Screening Terms **3**

Tables **5**

Supplementary Table S1. Adherence to Guideline-Recommended Cancer Screening at Enrollment for All Analyzable Participants (n=35,350). 5

Supplementary Table S2. Total Number of Diagnostic Evaluations Among Participants With a Positive MCED Test Result. 6

Supplementary Table S3. Number of Participants With Invasive Procedures Occurring During Targeted Diagnostic Evaluation vs After Diagnostic PET-CT. 7

Supplementary Table S4. Extent of Diagnostic Testing Per Participant Among Participants With a Positive MCED Test Result. 9

Supplementary Table S5. Number of Participants With Diagnostic Evaluations During Targeted Diagnostic Evaluation (Excluding Diagnostic PET-CT and Subsequent Evaluations) Among Participants With a Positive MCED Test Result. 11

Supplementary Table S6. Participants With Invasive Procedures Performed as Part of a CSO-Guided Diagnostic Evaluation. 12

Supplementary Table S7. Non-cancer Conditions Identified During Diagnostic Evaluation in Participants with False-Positive MCED Test Results by CSO1 Prediction. 13

Supplementary Table S8. Adverse Events Experienced During the Time of Diagnostic Evaluation Following a Positive MCED Test Result.[a] 18

Supplementary Table S9. Study-Related Adverse Events Reported in All Consented Participants.[a] 19

Supplementary Table S10. Performance of the MCED Test by Participant Age (10-Year Age Intervals). 21

Supplementary Table S11. Performance of the MCED Test by Participant Sex. 23

Supplementary Table S12. Performance of the MCED Test by Participant Smoking Status. 25

Supplementary Table S13. Cancers Diagnosed by Diagnostic PET-CT. 27

Supplementary Table S14. Definitions of Methods of Cancer Detection. 28

Supplementary Table S15. ICD-O-3 Categorization of Cancers Detected by the MCED Test. 29

Supplementary Table S16. Distribution of Primary and Recurrent Cancers by MCED Test Result. 36

Supplementary Table S17. Characteristics of First New Primary Cancers and Recurrences Diagnosed in PATHFINDER 2 Participants. 37

Supplementary Table S18. Early-Stage MCED-Detected Cancers in Predefined Cancer Subgroups..43

Supplementary Table S19. Clinical Sites and Primary Investigators for PATHFINDER 2. .44

Supplementary Table S20. Important Protocol Deviations...48

Supplementary Table S21. CSO Prediction–Guided Diagnostic Evaluation Recommendations. 49

Supplementary Table S22. Predefined Cancer Subgroups...52

Supplementary Table S23. Summary of PATHFINDER 2 Endpoints, Analysis Sets, and Statistical Methods. 55

Supplementary Table S24. Protocol and Statistical Analysis Plan Amendments. 62

Protocol and Statistical Analysis Plan 67

Glossary of Screening Terms

2×2 Contingency Table

		Cancer status	
		**Present**	**Absent**
**MCED test result for cancer signal detection**	**Positive**	True-positive (TP)	False-positive (FP)
	**Negative**	False-negative (FN)	True-negative (TN)

Term	Definition
**Cancer detection rate (CDR)**	The proportion of participants with a positive MCED test result and a cancer diagnosis (true positives) out of all participants in the performance analysis set<br>It is calculated as TP / (TP + FP + FN + TN)
**Cancer signal detection rate**	The proportion of participants with a positive MCED test result<br>It is calculated as (TP + FP) / (TP + FP + FN + TN)
**Cancer signal origin (CSO) prediction accuracy**	The proportion of participants with a correct CSO prediction (ie, CSO prediction matching clinical CSO) among participants with TP MCED test results
**12-month episode sensitivity**	Ability of a screening test to detect cancers that are diagnosed during the 12-month follow-up period after a specific round of a screening test<br>12-month episode sensitivity is estimated as the proportion of participants with a positive MCED test result out of all participants with a cancer diagnosis within the 12-month follow-up period<br>It is calculated as TP / (TP + FN)
**False-negative (FN)**	Participant with a negative MCED test result and a cancer diagnosis
**False-positive (FP)**	Participants with a positive MCED test result and no cancer diagnosis
**False-positive rate**	Complement to specificity

Term	Definition
	It is calculated as FP / (TN + FP)
**Negative likelihood ratio (NLR)**	The ratio of the probability that a participant with a cancer diagnosis has a negative test result vs the probability that a participant with no cancer diagnosis has a negative test result<br>It is calculated as (1 - episode sensitivity) divided by specificity, ie, [1 - TP/ (TP + FN)] / [TN/ (TN + FP)] = FN × (TN + FP) / [TN × (TP + FN)]
**Negative predictive value (NPV)**	The proportion of participants with no cancer diagnosis out of all participants with a negative MCED test result<br>It is calculated as TN / (TN + FN)
**Number needed to screen to detect 1 cancer**	The number of participants that need to be screened with an MCED test to detect a single cancer<br>It is calculated as 1 / CDR, ie, (TP + FP + FN + TN) / TP
**Positive likelihood ratio (PLR)**	The ratio of the probability that a participant with a cancer diagnosis has a positive test result vs the probability that a participant with no cancer diagnosis has a positive test result<br>It is calculated as episode sensitivity divided by (1 - specificity), ie, [TP/ (TP + FN)] / [1 - TN/ (TN + FP)] = TP × (TN + FP) / [FP × (TP + FN)]
**Positive predictive value (PPV)**	The proportion of participants with a cancer diagnosis out of all participants with a positive MCED test result<br>It is calculated as TP / (TP + FP)
**Specificity**	The proportion of participants with a negative MCED test result out of all participants with no cancer diagnosis within the follow-up period<br>It is calculated as TN / (TN + FP)
**True-negative (TN)**	Participant with a negative MCED test result and no cancer diagnosis
**True-positive (TP)**	Participant with a positive MCED test result and a cancer diagnosis

Tables

Supplementary Table S1. Adherence to Guideline-Recommended Cancer Screening at Enrollment for All Analyzable Participants (n=35,350).

Type of screening	Positive MCED test result (n=305)		Negative MCED test result (n=35,045)	
	Participants with screening information available out of all eligible, n/N (%)	Up to date in those with screening information available, n/N (%)	Participants with screening information available out of all eligible, n/N (%)	Up to date in those with screening information available, n/N (%)
**All USPSTF grade A/B recommendations**	169/235 (71.9)	146/169 (86.4)	23,007/32,007 (71.9)	19,830/23,007 (86.2)
**≥1 USPSTF grade A/B recommendation**	215/235 (91.5)	198/215 (92.1)	30,122/32,008 (94.1)	28,159/30,122 (93.5)
**Breast cancer[a]**	85/102 (83.3)	72/85 (84.7)	16,095/17,716 (90.9)	14,189/16,095 (88.2)
**Cervical cancer[b]**	33/53 (62.3)	28/33 (84.8)	6828/11,464 (59.6)	5913/6,828 (86.6)
**Colorectal cancer[c]**	199/234 (85.0)	181/199 (91.0)	27,815/31,942 (87.1)	25,645/27,815 (92.2)
**Lung cancer[d]**	7/26 (26.9)	3/7 (42.9)	440/1491 (29.5)	227/440 (51.6)

[a]Women aged 40-74 years are eligible for breast cancer screening.

[b]Women aged 21-65 years are eligible for cervical cancer screening.

[c]Women and men aged 45-75 years are eligible for colorectal cancer screening.

[d]Women and men aged 50-80 years satisfying USPSTF lung cancer screening criteria are eligible for lung cancer screening.

MCED, multi-cancer early detection; USPSTF, United States Preventive Services Task Force.

Supplementary Table S2. Total Number of Diagnostic Evaluations Among Participants With a Positive MCED Test Result.

Number of evaluations	Cancer diagnosis (n=167)[a]	No cancer diagnosis (n=123)	Total (n=290)
**All diagnostic evaluations, No.**	1068	921	1989
**Imaging tests**	281 (26.3)	255 (27.7)	536 (26.9)
Whole-body imaging[b]	101 (9.5)	115 (12.5)	216 (10.9)
**Laboratory tests[c]**	516 (48.3)	519 (56.4)	1035 (52.0)
**Noninvasive procedures[d]**	62 (5.8)	61 (6.6)	123 (6.2)
**Invasive procedures**	209 (19.6)	86 (9.3)	295 (14.8)
Surgical procedures	24 (2.2)	4 (0.4)	28 (1.4)
Nonsurgical procedures	185 (17.3)	82 (8.9)	267 (13.4)

Data are No. (%) unless otherwise noted.

[a]4 participants were diagnosed with cancer after diagnostic resolution.

[b]Whole-body imaging is defined as PET scan (ie, F-18 fluorodeoxyglucose or Ga68 Dotatate PET), PET-CT, PET-MRI, whole-body MRI or CT, or CT scan that includes all 3 of the following imaging fields: chest, abdomen, and pelvis.

[c]Includes blood- and urine-based tests.

[d]Includes physical exams.

CT, computed tomography; MCED, multi-cancer early detection; MRI, magnetic resonance imaging; PET, positron emission tomography.

Supplementary Table S3. Number of Participants With Invasive Procedures Occurring During Targeted Diagnostic Evaluation vs After Diagnostic PET-CT.

Number of participants with ≥1	Participants with an invasive procedure performed as part of a targeted diagnostic evaluation			Participants with an invasive procedure performed after the diagnostic PET-CT			Total (n=213), n (%)
	Participants with cancer diagnosed at diagnostic resolution (n=145), n (%)	All other participants (n=59)[a], n (%)	Total (n=204), n (%)	Participants with cancer diagnosed at diagnostic resolution (n=5), n (%)	All other participants (n=4)[a], n (%)	Total (n=9), n (%)	
**Invasive procedure**	145 (100.0)	59 (100.0)	204 (100.0)	5 (100.0)	4 (100.0)	9 (100.0)	213 (100.0)
**Surgical procedure**	22 (15.2)	4 (6.8)	26 (12.7)	2 (40.0)	0	2 (22.2)	28 (13.1)
**Nonsurgical procedure**	131 (90.3)	58 (98.3)	189 (92.6)	3 (60.0)	4 (100.0)	7 (77.8)	196 (92.0)
Endoscopy	60 (41.4)	41 (69.5)	101 (49.5)	1 (20.0)	3 (75.0)	4 (44.4)	105 (49.3)
Biopsy	88 (60.7)	18 (30.5)	106 (52.0)	1 (20.0)	1 (25.0)	2 (22.2)	108 (50.7)

Number of participants with ≥1	Participants with an invasive procedure performed as part of a targeted diagnostic evaluation			Participants with an invasive procedure performed after the diagnostic PET-CT			Total
	Participants with cancer diagnosed at diagnostic resolution (n=145), n (%)	All other participants (n=59)[a], n (%)	Total (n=204), n (%)	Participants with cancer diagnosed at diagnostic resolution (n=5), n (%)	All other participants (n=4)[a], n (%)	Total (n=9), n (%)	(n=213), n (%)
Other	1 (0.7)	3 (5.1)	4 (2.0)	1 (20.0)	0	1 (11.1)	5 (2.3)

[a]Includes participants who had no cancer diagnosed at diagnostic resolution, or participants who have not achieved diagnostic resolution within 12 months.

Supplementary Table S4. Extent of Diagnostic Testing Per Participant Among Participants With a Positive MCED Test Result.

	Cancer diagnosis (n=167)[a]	No cancer diagnosis (n=123)	Total (n=290)
**Any diagnostic evaluation**			
Mean (SD)	6.4 (3.6)	7.5 (5.2)	6.9 (4.4)
Median (Q1, Q3)	6.0 (4.0, 9.0)	7.0 (4.0, 10.0)	6.0 (4.0, 9.0)
Min, max	1.0, 17.0	1.0, 41.0	1.0, 41.0
**Any imaging test**			
Mean (SD)	1.7 (1.2)	2.1 (1.4)	1.8 (1.3)
Median (Q1, Q3)	2.0 (1.0, 2.0)	2.0 (1.0, 3.0)	2.0 (1.0, 3.0)
Min, max	0.0, 6.0	0.0, 7.0	0.0, 7.0
**Whole-body imaging[b]**			
Mean (SD)	0.6 (0.6)	0.9 (0.5)	0.7 (0.6)
Median (Q1, Q3)	1.0 (0.0, 1.0)	1.0 (1.0, 1.0)	1.0 (0.0, 1.0)
Min, max	0.0, 2.0	0.0, 2.0	0.0, 2.0
**Laboratory tests[c]**			
Mean (SD)	3.1 (3.0)	4.2 (4.6)	3.6 (3.8)
Median (Q1, Q3)	2.0 (0.0, 5.0)	3.0 (1.0, 6.0)	3.0 (0.0, 6.0)
Min, max	0.0, 13.0	0.0, 33.0	0.0, 33.0
**Noninvasive procedure[d]**			
Mean (SD)	0.4 (0.8)	0.5 (0.9)	0.4 (0.8)
Median (Q1, Q3)	0.0 (0.0, 0.5)	0.0 (0.0, 1.0)	0.0 (0.0, 1.0)
Min, max	0.0, 5.0	0.0, 6.0	0.0, 6.0
**Any invasive procedure**			
Mean (SD)	1.3 (0.8)	0.7 (0.9)	1.0 (0.9)

	Cancer diagnosis (n=167)[a]	No cancer diagnosis (n=123)	Total (n=290)
Median (Q1, Q3)	1.0 (1.0, 1.0)	0.0 (0.0, 1.0)	1.0 (0.0, 1.0)
Min, max	0.0, 5.0	0.0, 4.0	0.0, 5.0
**Surgical procedure**			
Mean (SD)	0.1 (0.4)	0.0 (0.2)	0.1 (0.3)
Median (Q1, Q3)	0.0 (0.0, 0.0)	0.0 (0.0, 0.0)	0.0 (0.0, 0.0)
Min, max	0.0, 1.0	0.0, 1.0	0.0, 1.0
**Nonsurgical procedure**			
Mean (SD)	1.1 (0.8)	0.7 (0.9)	0.9 (0.8)
Median (Q1, Q3)	1.0 (1.0, 1.0)	0.0 (0.0, 1.0)	1.0 (0.0, 1.0)
Min, max	0.0, 4.0	0.0, 4.0	0.0, 4.0

[a]4 participants were diagnosed with cancer after diagnostic resolution.

[b]Whole-body imaging is defined as PET scan (ie, F-18 fluorodeoxyglucose or Ga68 Dotatate PET), PET-CT, PET-MRI, whole-body MRI or CT, or CT scan that includes all 3 of the following imaging fields: chest, abdomen, and pelvis.

[c]Includes blood- and urine-based tests.

[d]Includes physical exams.

CT, computed tomography; MCED, multi-cancer early detection; MRI, magnetic resonance imaging; PET, positron emission tomography.

Supplementary Table S5. Number of Participants With Diagnostic Evaluations During Targeted Diagnostic Evaluation (Excluding Diagnostic PET-CT and Subsequent Evaluations) Among Participants With a Positive MCED Test Result.

Number of participants with ≥1	Cancer diagnosis (n=166)[a], n (%)	No cancer diagnosis (n=123), n (%)	Total (n=289)[b], n (%)
**Imaging tests**	142 (85.5)	100 (81.3)	242 (83.7)
Whole-body imaging[c]	80 (48.2)	51 (41.5)	131 (45.3)
**Laboratory tests[d]**	119 (71.7)	95 (77.2)	214 (74.0)
**Noninvasive procedures[e]**	41 (24.7)	39 (31.7)	80 (27.7)
**Invasive procedures**	147 (88.6)	57 (46.3)	204 (70.6)
**Surgical procedures**	22 (13.3)	4 (3.3)[f]	26 (9.0)
**Nonsurgical procedures**	133 (80.1)	56 (45.5)	189 (65.4)
Endoscopy	61 (36.7)	39 (31.7)	100 (34.6)
Biopsy	87 (52.4)	15 (12.2)	102 (35.3)
Other	1 (0.6)	3 (2.4)	4 (1.4)

[a]4 participants were diagnosed with cancer after diagnostic resolution.

[b]1 participant whose only targeted diagnostic evaluation was a diagnostic PET-CT was not included in this table.

[c]Whole-body imaging is defined as PET scan (ie, F-18 fluorodeoxyglucose or Ga68 Dotatate PET), PET-CT, PET-MRI, whole-body MRI or CT, or CT scan that includes all 3 of the following imaging fields: chest, abdomen, and pelvis.

[d]Includes blood- and urine-based tests.

[e]Includes physical exams.

[f]All 4 participants had a benign neoplasm.

CT, computed tomography; MCED, multi-cancer early detection; MRI, magnetic resonance imaging; PET, positron emission tomography.

Supplementary Table S6. Participants With Invasive Procedures Performed as Part of a CSO-Guided Diagnostic Evaluation.

Number of participants	Participants with a CSO-guided diagnostic evaluation (n=254), n (%)	Participants with a non–CSO-guided diagnostic evaluation (n=36), n (%)
**Invasive procedure**	197 (77.6)	16 (44.4)
**Surgical procedure**	28 (11.0)	0
**Nonsurgical procedure**	180 (70.9)	16 (44.4)
Endoscopy	98 (38.6)	7 (19.4)
Biopsy	99 (39.0)	9 (25.0)
Other	5 (2.0)	0

CSO, cancer signal origin.

Supplementary Table S7. Non-cancer Conditions Identified During Diagnostic Evaluation in Participants with False-Positive MCED Test Results by CSO1 Prediction.

**Non-cancer conditions identified during diagnostic evaluation in participants with false-positive MCED test results by CSO1 prediction[a]**	**Number of participants (n=114)**
**Total number of participants with benign neoplasms and/or non-cancer conditions, n (%)**	61 (53.3)
Benign neoplasm, n (%)	38 (33.3)
Non-cancer conditions, n (%)	32 (28.1)
**Total number of participants with no conditions identified, n (%)**	53 (46.5)
**CSO1 = Anus, n (%)**	1 (0.9)
No conditions identified, n	1
**CSO1 = Bone and Soft Tissue, n (%)**	1 (0.9)
Benign mass or nodule, n	1
**CSO1 = Breast, n (%)**	12 (10.5)
Benign mass or nodule, n	2
Other benign neoplasm, n	1
Non-cancer condition, n	1
Liver disease (eg; cirrhosis or fatty liver disease), n	1
No conditions identified, n	8
**CSO1 = Colon, Rectum, n (%)**	18 (15.8)
Benign mass or nodule, n	8
Other benign neoplasm, n	2
Non-cancer condition, n	3

Non-cancer conditions identified during diagnostic evaluation in participants with false-positive MCED test results by CSO1 prediction[a]	Number of participants (n=114)
Autoimmune disease, n	1
Esophageal disease (eg; Barrett's esophagus; GERD), n	1
Gastritis or gastric (peptic) ulcers, n	1
Inflammatory bowel disease (ie; Crohn's disease or ulcerative colitis), n	1
Other non-cancer condition, n	2
No conditions identified, n	6
**CSO1 = Head and Neck, n (%)**	8 (7.0)
Benign mass or nodule, n	2
Non-cancer conditions, n	1
Gastritis or gastric (peptic) ulcers, n	1
No conditions identified, n	5
**CSO1 = Liver, Bile Duct, n (%)**	3 (2.6)
Benign mass or nodule, n	1
Non-cancer condition, n	3
Autoimmune disease, n	1
Liver disease (eg; cirrhosis or fatty liver disease), n	1
Other non-cancer condition, n	1
**CSO1 = Lung, n (%)**	13 (11.4)
Benign mass or nodule, n	2

Non-cancer conditions identified during diagnostic evaluation in participants with false-positive MCED test results by CSO1 prediction[a]	Number of participants (n=114)
Other benign neoplasm, n	2
Non-cancer conditions, n	9
Gastritis or gastric (peptic) ulcers, n	2
Lung disease (eg; pulmonary fibrosis; emphysema; chronic bronchitis), n	2
Other non-cancer condition, n	6
No conditions identified, n	3
**CSO1 = Lymphoid Lineage, n (%)**	30 (26.3)
Benign mass or nodule, n	5
Lymphoproliferative disorder, n	2
MGUS, n	2
Other benign neoplasm, n	2
Non-cancer condition, n	4
Other non-cancer condition, n	4
No conditions identified, n	20
**CSO1 = Pancreas, Gallbladder, n (%)**	7 (6.1)
Benign mass or nodule, n	1
Other benign neoplasm, n	1
Non-cancer condition, n	1
Other non-cancer condition, n	1

Non-cancer conditions identified during diagnostic evaluation in participants with false-positive MCED test results by CSO1 prediction[a]	Number of participants (n=114)
No conditions identified, n	4
**CSO1 = Plasma Cell Lineage, n (%)**	4 (3.5)
MGUS, n	2
No conditions identified, n	2
**CSO1 = Prostate, n (%)**	8 (7.0)
MGUS, n	1
Non-cancer conditions, n	3
Other non-cancer condition, n	3
No conditions identified, n	4
**CSO1 = Stomach, Esophagus, n (%)**	4 (3.5)
Benign mass or nodule, n	1
Non-cancer conditions, n	3
Esophageal disease (eg; Barrett's esophagus; GERD), n	1
Gastritis or gastric (peptic) ulcers, n	1
Other non-cancer condition, n	3
**CSO1 = Uterus, n (%)**	5 (4.4)
Benign mass or nodule, n	2
Non-cancer conditions, n	4
Other non-cancer condition, n	4

[a]Participants may have ≥1 condition; therefore, subcategory counts are not mutually exclusive and may not sum to the total.

CSO, cancer signal origin; GERD, gastroesophageal reflux disease; MCED, multi-cancer early detection; MGUS, monoclonal gammopathy of undetermined significance.

Supplementary Table S8. Adverse Events Experienced During the Time of Diagnostic Evaluation Following a Positive MCED Test Result.[a]

Number of AEs	AEs in participants with a cancer diagnosis (Events=9), No. (%)	AEs in participants with no cancer diagnosis (Events=1), No. (%)	Total number of AEs (Events=10[b]), No. (%)
**Non-serious AE**	8 (88.9)	1 (100.0)	9 (90.0)[c]
Study-related AE	5 (55.6)	0	5 (50.0)
**Serious AE (SAE)**	1 (11.1)	0	1 (10.0)[d]
Study-related SAE	0	0	0
**AE setting**			
Diagnostic procedure prompted by a positive MCED test result	6 (66.7)	1 (100.0)	7 (70.0)
Other	3 (33.3)	0	3 (30.0)
**AE related to device among study-related AEs**			
Device-related	0	0	0

[a]As of data lock date 11 February 2026.

[b]9 participants experienced 10 AEs.

[c]4 participants experienced 4 non-serious, not study-related AEs, which included 1 report each of globus sensation, thrombocytopenia, anemia, and increased creatinine. 4 participants experienced 5 non-serious, study-related AEs, which included an Emergency Department visit; planned hospitalization for pain control; anxiety after receipt of a cancer signal detected MCED test result; postoperative pain; and low glomerular filtration rate.

[d]1 participant experienced 1 serious, not study-related AE of hyponatremia.

AE, adverse event; MCED, multi-cancer early detection.

Supplementary Table S9. Study-Related Adverse Events Reported in All Consented Participants.[a]

Number of participants[b]	All analyzable participants		All other participants (n=500),[c] n (%)	All consented (n=35,850), n (%)
	Participants with a positive MCED test result (n=305), n (%)	Participants with a negative MCED test result (n=35,045), n (%)		
No AE	291 (95.4)	35,011 (99.9)	492 (98.4)	35,794 (99.8)
1 AE only	13 (4.3)	34 (0.1)	8 (1.6)	55 (0.2)
> 1 AE	1 (0.3)	0	0	1 (0.0)
Serious AE	0	0	0	0
**AE severity**				
Mild	12 (3.9)	32 (0.1)	7 (1.4)	51 (0.1)
Moderate	2 (0.7)	2 (0.0)	1 (0.2)	5 (0.0)
Severe	0	0	0	0
**AE related to device among study-related AEs**				
Device related	0	0	0	0
Not device related	14 (4.6)	34 (0.1)	8 (1.6)	56 (0.2)
**AE outcome**				
Recovered/resolved	11 (3.6)	34 (0.1)	7 (1.4)	52 (0.1)
Recovering/resolving	1 (0.3%)	0	0	1 (0.0%)
Fatal	0	0	0	0
Missing	3 (1.0)	0	1 (0.2)	4 (0.0)
**AE setting[d]**				
Anticipation or communication of MCED test result	2 (0.7)	0	0	2 (0.0)

Number of participants[b]	All analyzable participants: Participants with a positive MCED test result (n=305), n (%)	All analyzable participants: Participants with a negative MCED test result (n=35,045), n (%)	All other participants (n=500),[c] n (%)	All consented (n=35,850), n (%)
Diagnostic procedure prompted by a positive MCED test result[e]	11 (3.6)	0	1 (0.2%)	12 (0.0)
Other	1 (0.3)	0	0	1 (0.0)
Phlebotomy	1 (0.3)	34 (0.1)	7 (1.4)	42 (0.1)

[a]As of data lock date 11 February 2026.

[b]Participants may report multiple AEs; therefore, column totals may exceed the number of unique participants and do not sum to the overall total.

[c]Includes all consented participants who are either not eligible or not evaluable, and therefore not part of the analyzable set.

[d]1 AE has been reported with multiple settings: "Anticipation or communication of MCED test result" and "Diagnostic procedure prompted by a positive test result."

[e]Participant counts with AEs in this table are higher than in Table S8 because Table S8 is limited to AEs reported during the time of diagnostic evaluations triggered by positive MCED test results. This table includes all study-related AEs in consented participants with clean data, regardless of when they occurred.

AE, adverse event; MCED, multi-cancer early detection.

Supplementary Table S10. Performance of the MCED Test by Participant Age (10-Year Age Intervals).

Metric	Performance analysis set n=32,007			
	Age 50-59 y (n=8759)	Age 60-69 y (n=14,259)	Age 70-79 y (n=8083)	Age ≥80 y (n=906)
**Cancers detected/cancers diagnosed, n/n**	24/63	82/203	54/150	13/24
**MCED cancer detection rate, % (95% CI)**	0.27% (0.18%-0.41%)	0.58% (0.46%-0.71%)	0.67% (0.51%-0.87%)	1.43% (0.84%-2.44%)
**Positive predictive value, % (95% CI)**	75.0% (57.9%-86.7%)	66.1% (57.4%-73.9%)	54.0% (44.3%-63.4%)	41.9% (26.4%-59.2%)
**Negative predictive value, % (95% CI)**	99.6% (99.4%-99.7%)	99.1% (99.0%-99.3%)	98.8% (98.5%-99.0%)	98.7% (97.8%-99.3%)
**12-month episode sensitivity, % (95% CI)**	38.1% (27.1%-50.4%)	40.4% (33.9%-47.3%)	36.0% (28.8%-43.9%)	54.2% (35.1%-72.1%)
**Specificity, % (95% CI)**	99.91% (99.82%-99.95%)	99.70% (99.60%-99.78%)	99.42% (99.23%-99.56%)	97.96% (96.80%-98.71%)
**False-positive rate, % (95% CI)**	0.09% (0.05%-0.18%)	0.30% (0.22%-0.40%)	0.58% (0.44%-0.77%)	2.04% (1.29%-3.20%)

Metric	Performance analysis set n=32,007			
	**Age 50-59 y (n=8759)**	**Age 60-69 y (n=14,259)**	**Age 70-79 y (n=8083)**	**Age ≥80 y (n=906)**
**Positive likelihood ratio, (95% CI)**	414.1 (193.5-886.2)	135.2 (95.7-190.9)	62.1 (43.4-88.9)	26.5 (14.8-47.7)
**Negative likelihood ratio, (95% CI)**	0.62 (0.51-0.75)	0.60 (0.53-0.67)	0.64 (0.57-0.73)	0.47 (0.30-0.72)
**Number needed to screen to detect 1 cancer, n (95% CI)**	365 (245-543)	174 (140-216)	150 (115-195)	70 (41-119)
**MCED cancer signal detection rate, % (95% CI)**	0.37% (0.26%-0.52%)	0.87% (0.73%-1.04%)	1.24% (1.02%-1.50%)	3.42% (2.42%-4.82%)
**CSO prediction accuracy,[a] % (95% CI)**				
CSO1 prediction correct	79.2% (59.5%-90.8%)	90.2% (81.9%-95.0%)	85.2% (73.4%-92.3%)	92.3% (66.7%-98.6%)
CSO1 or CSO2 prediction correct	91.7% (74.2%-97.7%)	90.2% (81.9%-95.0%)	92.6% (82.4%-97.1%)	92.3% (66.7%-98.6%)

[a]Calculated out of participants with true-positive MCED test results (n=173).

CSO, cancer signal origin; MCED, multi-cancer early detection.

Supplementary Table S11. Performance of the MCED Test by Participant Sex.

Metric	Performance analysis set n=32,007	
	**Female (n=18,117)**	**Male (n=13,890)**
**Cancers detected/cancers diagnosed, n/n**	80/206	93/234
**MCED cancer detection rate, % (95% CI)**	0.44% (0.35%-0.55%)	0.67% (0.55%-0.82%)
**Positive predictive value, % (95% CI)**	62.0% (53.4%-69.9%)	58.9% (51.1%-66.2%)
**Negative predictive value, % (95% CI)**	99.3% (99.2%-99.4%)	99.0% (98.8%-99.1%)
**12-month episode sensitivity, % (95% CI)**	38.8% (32.4%-45.6%)	39.7% (33.7%-46.1%)
**Specificity, % (95% CI)**	99.73% (99.64%-99.79%)	99.52% (99.39%-99.63%)
**False-positive rate, % (95% CI)**	0.27% (0.21%-0.36%)	0.48% (0.37%-0.61%)
**Positive likelihood ratio, (95% CI)**	142.0 (102.3-197.0)	83.5 (62.5-111.5)
**Negative likelihood ratio, (95% CI)**	0.61 (0.55-0.68)	0.61 (0.55-0.67)
**Number needed to screen to detect 1 cancer, n (95% CI)**	226 (182-282)	149 (122-183)
**MCED cancer signal detection rate, % (95% CI)**	0.71% (0.60%-0.85%)	1.14% (0.97%-1.33%)
**CSO prediction accuracy,[a] % (95% CI)**		

Metric	Performance analysis set n=32,007	
	**Female (n=18,117)**	**Male (n=13,890)**
CSO1 prediction correct	90.0% (81.5%-94.8%)	84.9% (76.3%-90.8%)
CSO1 or CSO2 prediction correct	92.5% (84.6%-96.5%)	90.3% (82.6%-94.8%)

[a]Calculated out of participants with true-positive MCED test results (n=173).

CSO, cancer signal origin; MCED, multi-cancer early detection.

Supplementary Table S12. Performance of the MCED Test by Participant Smoking Status.

Metric	Performance analysis set n=32,007	
	**Never smoker (n=22,103)**	**Ever smoker (n=9904)**
**Cancers detected/cancers diagnosed, n/n**	95/267	78/173
**MCED cancer detection rate, % (95% CI)**	0.43% (0.35%-0.53%)	0.79% (0.63%-0.98%)
**Positive predictive value, % (95% CI)**	57.6% (49.9%-64.9%)	63.9% (55.1%-71.9%)
**Negative predictive value, % (95% CI)**	99.2% (99.1%-99.3%)	99.0% (98.8%-99.2%)
**12-month episode sensitivity, % (95% CI)**	35.6% (30.1%-41.5%)	45.1% (37.9%-52.5%)
**Specificity, % (95% CI)**	99.68% (99.60%-99.75%)	99.55% (99.39%-99.66%)
**False-positive rate, % (95% CI)**	0.32% (0.25%-0.40%)	0.45% (0.34%-0.61%)
**Positive likelihood ratio, (95% CI)**	111.0 (83.5-147.5)	99.7 (71.1-139.8)
**Negative likelihood ratio, (95% CI)**	0.65 (0.59-0.71)	0.55 (0.48-0.63)
**Number needed to screen to detect 1 cancer, n (95% CI)**	233 (190-284)	127 (102-158)
**MCED cancer signal detection rate, % (95% CI)**	0.75% (0.64%-0.87%)	1.23% (1.03%-1.47%)
**CSO prediction accuracy,[a] % (95% CI)**		

Metric	Performance analysis set n=32,007	
	**Never smoker (n=22,103)**	**Ever smoker (n=9904)**
CSO1 prediction correct	90.5% (83.0%-94.9%)	83.3% (73.5%-90.0%)
CSO1 or CSO2 prediction correct	93.7% (86.9%-97.1%)	88.5% (79.5%-93.8%)

[a]Calculated out of participants with true-positive MCED test results (n=173).

CSO, cancer signal origin; MCED, multi-cancer early detection.

Supplementary Table S13. Cancers Diagnosed by Diagnostic PET-CT.

Use of PET-CT	Participants with targeted diagnostic evaluation[a] negative for cancer		
	Cancer diagnosis (n=7)	No cancer diagnosis (n=112)	Total (n=119)
**During negative targeted diagnostic testing only, n**	NA	41	41
**After negative targeted diagnostic evaluation only, n**	7	56	63
**Both during *and* after negative targeted diagnostic evaluation, n**	0	2	2
**No PET-CT performed at all, n**	NA	13	13
**Participants with cancer at diagnostic resolution out of all participants who had a PET-CT only after negative targeted diagnostic testing, % (n/N)**			11.1% (7/63)
**Participants with cancer at diagnostic resolution out of all participants with negative targeted diagnostic testing who had a diagnostic PET-CT at any time during diagnostic evaluation, % (n/N)**			6.6% (7/106)

[a]Targeted diagnostic testing is defined as any initial or subsequent testing performed before the diagnostic PET-CT (if any) or prior to diagnostic resolution.

NA, not applicable; PET-CT, positron emission tomography-computed tomography.

Supplementary Table S14. Definitions of Methods of Cancer Detection.

**Detection method**	**Definition**
Detected by MCED test	Participants with a positive MCED test result and cancer diagnosis during the 12-month follow-up period
Detected by cancer screening test	Participants with a negative MCED test result whose cancer diagnosis during the 12-month follow-up period was triggered by an existing screening test for cancers with a USPSTF grade A, B, or C recommendation: breast, cervix, colorectal, lung, or prostate cancers
Clinically detected	Participants with a negative MCED test result whose cancer diagnosis during the 12-month follow-up period was not triggered by cancer screening. Routes of clinical detection include:<br>● Participant-reported signs and symptoms<br>● Incidental finding on imaging test, laboratory test, physical exam, or surgical procedure<br>● Surveillance of prior imaging test finding or posttreatment surveillance for recurrence<br>● Other (eg, follow-up of an abnormal test result, incidental finding on nonsurgical procedure, or unknown)

MCED, multi-cancer early detection; USPSTF, United States Preventive Services Task Force.

Supplementary Table S15. ICD-O-3 Categorization of Cancers Detected by the MCED Test.

Clinical cancer type	Simplified site	Histology	ICD-O-3 site(s)	ICD-O-3 histology	Breast subtype(s)
Anus	Anus	Squamous cell carcinoma, NOS	C211	8070	
	Anus	Squamous cell carcinoma, keratinizing, NOS	C210	8071	
	Anus	Basaloid squamous cell carcinoma	C210	8083	
Bladder, Urothelial Tract	Bladder	Urothelial carcinoma	C675	8120	
	Renal pelvis	Urothelial carcinoma	C659	8120	
	Ureter	Papillary urothelial carcinoma	C669	8130	
Bone or Soft Tissue Sarcoma	Colon, NOS	Angiosarcoma	C189	9120	
Breast	Breast	Adenocarcinoma, NOS	C504, C509	8140	ER/PR positive, HER2 negative
	Breast	Invasive carcinoma of no special type	C502, C503, C504, C509	8500	ER/PR positive, HER2 negative; HER2 positive (regardless of ER/PR status); Unknown subtype
	Breast	Lobular carcinoma, NOS	C509	8520	ER/PR positive, HER2 negative

Clinical cancer type	Simplified site	Histology	ICD-O-3 site(s)	ICD-O-3 histology	Breast subtype(s)
	Breast	Infiltrating duct and lobular carcinoma	C509	8522	ER/PR positive, HER2 negative
Colon /Rectum	Ascending colon	Adenocarcinoma, NOS	C182	8140	
	Ascending colon	Mucinous adenocarcinoma	C182	8480	
	Cecum	Adenocarcinoma, NOS	C180	8140	
	Descending colon	Adenocarcinoma, NOS	C186	8140	
	Descending colon	Mucinous adenocarcinoma	C186	8480	
	Hepatic flexure of colon	Adenocarcinoma, NOS	C183	8140	
	Rectum	Squamous cell carcinoma, NOS	C209	8070	
	Rectum	Adenocarcinoma, NOS	C209	8140	
	Rectum	Adenocarcinoma in adenomatous polyp	C209	8210	
	Rectum	Adenocarcinoma in tubulovillous adenoma	C209	8263	
	Sigmoid colon	Adenocarcinoma, NOS	C187	8140	
	Sigmoid colon	Adenocarcinoma in tubulovillous adenoma	C187	8263	
	Transverse colon	Adenocarcinoma, NOS	C184	8140	
Esophagus	Esophagus	Adenocarcinoma, NOS	C155	8140	

Clinical cancer type	Simplified site	Histology	ICD-O-3 site(s)	ICD-O-3 histology	Breast subtype(s)
Head and Neck	Head, face, or neck, NOS	Squamous cell carcinoma, NOS	C760	8070	
	Hypopharynx	Squamous cell carcinoma, NOS	C139	8070	
	Larynx	Squamous cell carcinoma, NOS	C321	8070	
	Nasal cavity	Squamous cell carcinoma, HPV-positive	C300	8085	
	Oropharynx	Squamous cell carcinoma, HPV-positive	C109	8085	
	Pyriform sinus	Squamous cell carcinoma, NOS	C129	8070	
	Skin	Squamous cell carcinoma, NOS	C443	8070	
	Tongue	Squamous cell carcinoma, NOS	C019	8070	
	Tongue	Squamous cell carcinoma, HPV-positive	C019	8085	
	Tonsil	Squamous cell carcinoma, NOS	C099	8070	
	Tonsil	Squamous cell carcinoma, HPV-positive	C099	8085	
Kidney	Kidney	Clear cell adenocarcinoma, NOS	C649	8310	
	Kidney	Renal cell carcinoma, NOS	C649	8312	
Liver, Intrahepatic Bile-Duct	Intrahepatic bile duct	Cholangiocarcinoma	C221	8160	
	Liver	Cholangiocarcinoma	C220	8160	

Clinical cancer type	Simplified site	Histology	ICD-O-3 site(s)	ICD-O-3 histology	Breast subtype(s)
	Liver	Hepatocellular carcinoma, NOS	C220	8170	
	Liver	Combined hepatocellular carcinoma and cholangiocarcinoma	C220	8180	
Lung	Lung	Small cell carcinoma, NOS	C343, C349	8041	
	Lung	Non-small cell carcinoma	C341, C349	8046	
	Lung	Adenocarcinoma, NOS	C341, C343, C349	8140	
	Lung	Carcinoid tumor, NOS	C349	8240	
Lymphoid Lineage	Blood	Chronic lymphocytic leukemia/small lymphocytic lymphoma	C420	9823	
	Bone marrow	Diffuse large B-cell lymphoma, NOS	C421	9680	
	Bone of limb	Follicular lymphoma, NOS	C400	9690	
	Lymph nodes	Malignant lymphoma, non-Hodgkin, NOS	C774	9591	
	Lymph nodes	Hodgkin lymphoma, nodular lymphocyte predominance	C774	9659	
	Lymph nodes	Nodular sclerosis classical Hodgkin lymphoma	C770	9663	
	Lymph nodes	Diffuse large B-cell lymphoma, NOS	C770, C772, C773	9680	

Clinical cancer type	Simplified site	Histology	ICD-O-3 site(s)	ICD-O-3 histology	Breast subtype(s)
	Lymph nodes	Follicular lymphoma, NOS	C770, C772, C774	9690	
	Lymph nodes	Chronic lymphocytic leukemia/small lymphocytic lymphoma	C773	9823	
	Retroperitoneum	Diffuse large B-cell lymphoma, NOS	C480	9680	
	Retroperitoneum	Follicular lymphoma, NOS	C480	9690	
	Retroperitoneum	Follicular lymphoma, grade 3	C480	9698	
	Soft tissue	Diffuse large B-cell lymphoma, NOS	C494	9680	
	Spleen	Diffuse large B-cell lymphoma, NOS	C422	9680	
Ovary or Fallopian Tubes	Ovary	Serous carcinoma, NOS	C569	8441	
	Ovary	High-grade serous carcinoma	C569	8461	
Pancreas, Extrahepatic Bile Duct, Gallbladder	Extrahepatic bile duct	Adenocarcinoma, NOS	C240	8140	
	Gallbladder	Adenocarcinoma, NOS	C239	8140	
	Pancreas	Adenocarcinoma, NOS	C250, C251	8140	
	Pancreas	Neuroendocrine tumor, grade 2	C252	8249	
	Pancreas	Intraductal papillary-mucinous carcinoma, invasive	C252	8453	

**Clinical cancer type**	**Simplified site**	**Histology**	**ICD-O-3 site(s)**	**ICD-O-3 histology**	**Breast subtype(s)**
	Pancreas	Ductal adenocarcinoma	C251, C252	8500	
Plasma Cell Lineage	Bone marrow	Plasma cell myeloma	C421	9732	
Prostate	Prostate	Small cell carcinoma, NOS	C619	8041	
	Prostate	Adenocarcinoma, NOS	C619	8140	
Stomach	Stomach	Adenocarcinoma, NOS	C160, C163	8140	
Uterus	Uterus	Adenocarcinoma, NOS	C541	8140	
	Uterus	Endometrioid adenocarcinoma, NOS	C541, C559	8380	
	Uterus	High-grade serous carcinoma	C541	8461	
Other					
Uveal Melanoma	Choroid	Malignant melanoma, NOS (except juvenile melanoma M-8770/0)	C693	8720	
Thymus	Mediastinum	Thymoma, NOS	C383	8580	
Small Intestine	Small intestine	Adenocarcinoma, NOS	C171	8140	
Testis	Testis	Seminoma, NOS	C629	9061	
Thymus	Thymus	Squamous cell carcinoma, NOS	C379	8070	
Unknown Primary Site	Unknown primary site	Adenocarcinoma, NOS	C809	8140	

ER, estrogen receptor; HER2, human epidermal growth factor receptor 2; HPV, human papillomavirus; ICD-O-3, International Classification of Diseases for Oncology 3; MCED, multi-cancer early detection; NOS, not otherwise specified; PR, progesterone receptor.

Supplementary Table S16. Distribution of Primary and Recurrent Cancers by MCED Test Result.

Participants with cancer	True-positive MCED test result (n=173), n (%)	False-negative MCED test result (n=267), n (%)	Total (n=440), n (%)
**New primary cancer**	151 (87.3)[a]	244 (91.4)	395 (89.8)
Multiple new primary cancers[b]	3 (1.7)	6 (2.2)	9 (2.0)
**Recurrent cancer**	22 (12.7)[a]	23 (8.6)	45 (10.2)
**Unknown**[c]	1 (0.6)	0	1 (0.2)

[a]1 participant had a new primary colon cancer and a recurrent colon cancer during the study. This participant is included in both 'New primary' and 'Recurrent' rows in this table.

[b]All participants with multiple cancers had 2 new primary cancers diagnosed during the 12-month follow-up period.

[c]Participant with cancer of unknown primary site.

MCED, multi-cancer early detection.

Supplementary Table S17. Characteristics of First New Primary Cancers and Recurrences Diagnosed in PATHFINDER 2 Participants.

Cancer type diagnosed	Stage of new cancers, n					Extent of disease spread for recurrent cancers, n				Total, n
	I	II	III	IV	Missing or no TNM stage expected	Local	Regional	Distant	Missing	
**Participants with true-positive MCED test results[a]**										
**Anus**	1	3	1	0	0	0	0	0	0	5
**Bladder or urothelial tract**	1	0	1	1	0	0	0	0	0	3
**Bone or soft tissue sarcoma**	0	0	0	1	0	0	0	0	0	1
**Breast**	4	2	0	0	0	0	3	10	0	19
**Cervix**	0	0	0	0	0	0	0	0	0	0
**Colon or rectum**	8	6	4	0	0	0	0	1	0	18[c]
**Esophagus**	0	0	2	0	0	1	0	0	0	3
**Head and neck**	13	2	2	0	0	0	1	0	0	18

Cancer type diagnosed	Stage of new cancers, n					Extent of disease spread for recurrent cancers, n				Total, n
	I	II	III	IV	Missing or no TNM stage expected	Local	Regional	Distant	Missing	
**Kidney**	1	3	0	1	0	0	0	0	0	5
**Liver or intrahepatic bile duct**	8	4	5	1	0	0	0	0	0	18
**Lung**	2	1	5	2	0	0	0	0	0	10
**Lymphoid lineage**	3	5	3	9	5	0	2	0	1	28
**Myeloid lineage**	0	0	0	0	0	0	0	0	0	0
**Ovary or fallopian tube**	0	0	1	3	0	0	0	1	0	5
**Pancreas, extrahepatic bile duct, or gallbladder**	2	3	1	7	0	0	0	0	0	13
**Plasma cell lineage**	1	2	0	0	2	0	0	0	0	5
**Prostate**	1	0	1	7	0	0	0	0	0	9

Cancer type diagnosed	Stage of new cancers, n					Extent of disease spread for recurrent cancers, n				Total, n
	I	II	III	IV	Missing or no TNM stage expected	Local	Regional	Distant	Missing	
Skin (excluding BCC/SCC)	0	0	0	0	0	0	0	0	0	0
Stomach	0	0	1	0	1	0	0	0	0	2
Thyroid	0	0	0	0	0	0	0	0	0	0
Uterus	2	0	0	2	0	0	0	0	0	4
Other[b]	1	1	0	2	0	1	0	1	0	7[d]
Total	48	32	27	36	8	2	6	13	1	173[d]
Participants with false-negative MCED test results[a]										
Anus	0	0	1	0	0	0	0	0	0	1
Bladder or urothelial tract	6	0	0	0	0	0	0	0	0	6
Bone or soft tissue sarcoma	3	0	1	1	1	0	0	0	0	6
Breast	36	9	5	1	0	5	1	5	0	62

Cancer type diagnosed	Stage of new cancers, n					Extent of disease spread for recurrent cancers, n				Total, n
	I	II	III	IV	Missing or no TNM stage expected	Local	Regional	Distant	Missing	
**Cervix**	0	0	0	0	0	0	0	0	0	0
**Colon or rectum**	1	1	0	0	1	0	0	0	0	3
**Esophagus**	1	0	0	1	0	0	0	0	0	2
**Head and neck**	3	1	0	2	0	0	0	0	0	6
**Kidney**	12	0	1	0	1	1	0	1	0	16
**Liver or intrahepatic bile duct**	0	0	1	1	0	0	0	0	0	2
**Lung**	7	0	2	2	0	0	0	1	0	12
**Lymphoid lineage**	0	1	2	2	6	0	0	0	0	11
**Myeloid lineage**	0	0	0	0	9	0	0	0	0	9
**Ovary or fallopian tube**	3	0	1	0	0	0	0	1	0	5

Cancer type diagnosed	Stage of new cancers, n					Extent of disease spread for recurrent cancers, n				Total, n
	I	II	III	IV	Missing or no TNM stage expected	Local	Regional	Distant	Missing	
**Pancreas, extrahepatic bile duct, or gallbladder**	2	0	0	1	0	0	0	0	0	3
**Plasma cell lineage**	0	2	0	0	0	0	0	0	0	2
**Prostate**	24	34	13	6	4	1	2	2	0	86
**Skin (excluding BCC/SCC)**	15	0	2	0	1	0	0	1	1	20
**Stomach**	1	0	0	1	0	0	0	0	0	2
**Thyroid**	5	0	0	0	0	0	0	0	0	5
**Uterus**	5	0	0	0	0	0	0	1	0	6
**Other[b]**	1	0	0	0	1	0	0	0	0	2
**Total**	125	48	29	18	24	7	3	12	1	267

[a]For participants with multiple new primary cancers, only information from the first diagnosed cancer is included in the participant-level summary.

[b]Other cancer types include Ampulla of Vater (n=1), Brain and Spinal Cord (n=1), Unknown Primary Site (n=1), Small Intestine (n=1), Testis (n=2), and Thymus (n=2), Uveal Melanoma (n=1).

[c]1 participant had both a new and a recurrent colon cancer during the study; this participant is included in both the new and recurrent cancer categories in this row, but they are only counted once in the total.

[d]There was 1 additional “Other” cancer that had missing categorization of new or recurrent cancer and is thus only counted in the total.

BCC, basal cell carcinoma; MCED, multi-cancer early detection; SCC, squamous cell carcinoma; TNM, Tumor, Nodes, Metastasis.

Supplementary Table S18. Early-Stage MCED-Detected Cancers in Predefined Cancer Subgroups.

Cancer subgroup[a,b]	Participants with cancers detected by the MCED test[c]	
	Stage I-II, n/N (%)	Stage I-III, n/N (%)
**Cancers responsible for 2/3 of all cancer deaths in the US**	64/121 (52.9)	90/121 (74.4)
**Aggressive cancers with low 5-year survival**	20/49 (40.8)	35/49 (71.4)
**Cancers without common screening options[d]**	59/118 (50.0)	81/118 (68.6)
**Solid cancers with common screening options[d]**	21/33 (63.6)	26/33 (78.8)
**Solid cancers without common screening options[d]**	48/88 (54.5)	67/88 (76.1)
**Hematologic malignancies**	10/28 (35.7)	13/28 (46.4)

[a]Cancer subgroups are defined in Supplementary Table S22.

[b]No TNM stage is expected for cancers such as brain and spinal cord, leukemia, myeloma and plasma cell disorders, polycythemia vera, and cancer of unknown primary site.

[c]Participants with multiple cancers were included in a group only if all cancers belong to the group. The stage was counted per the first diagnosed cancer; if there were multiple cancers diagnosed on the same earliest day, the highest staged cancer was selected.

[d]Cancers with common screening options include cancers of the breast, cervix, colon or rectum, and prostate. For these cancers, population-wide cancer screening is recommended by several medical societies, and there is an age-based A, B, or C recommendation for screening made by the USPSTF. Lung cancer is not included in this definition because screening recommendations apply to a subset of individuals who are at high risk for this cancer due to their smoking history. Source: https://www.uspreventiveservicestaskforce.org/uspstf/.

MCED, multi-cancer early detection; TNM, Tumor, Nodes, Metastasis.

Supplementary Table S19. Clinical Sites and Primary Investigators for PATHFINDER 2.

We would like to thank the clinical study sites and investigators for their support and participation in the PATHFINDER 2 study.

Organization name	State/Province	Principal investigator name	IRB name
Cleveland Clinic	Ohio	Raevti Bole	Cleveland Clinic IRB
Duke University Health System	North Carolina	Kevin Oeffinger	Duke Health Institutional Review Board
Eastern Virginia Medical School at Old Dominion University	Virginia	Sami Tahan	Advarra Institutional Review Board
Flushing Hospital Medical Center	New York	Tamar Toronjadze	Advarra Institutional Review Board
HCA Healthcare Sarah Cannon Cancer Network	Tennessee	Dax Kurbegov	WCG IRB
Henry Ford Health	Michigan	Shirish Gadgeel	HFHS Institutional Review Board
Hoag Memorial Hospital	California	Michael Demeure	Advarra Institutional Review Board
Inova Schar Cancer Institute	Virginia	Rebecca Kaltman	Advarra Institutional Review Board
Jamaica Hospital Medical Center	New York	Alan Roth	Advarra Institutional Review Board

Organization name	State/Province	Principal investigator name	IRB name
Kelsey Research Foundation	Texas	Tri Vu	Advarra Institutional Review Board
Long Beach Memorial Medical Center	California	Gretchen Stipec	Advarra Institutional Review Board
Maryland Oncology Hematology, PA	Maryland	Jason Taksey	USOR IRB
Mayo Clinic	Minnesota	Karthik Giridhar	Mayo Clinic Institutional Review Boards
MedStar Washington Hospital Center	Washington, DC	Jennifer Tran	Advarra Institutional Review Board
Morehouse School of Medicine	Georgia	Roland Matthews	Advarra Institutional Review Board
Northwest Cancer Specialists, PC	Washington	David Cosgrove	USOR IRB
Ochsner Clinic Foundation	Louisiana	Marc Matrana	Ochsner Clinic Foundation IRB
Oregon Health & Science University	Oregon	Nima Nabavizadeh	OHSU IRB #3
Palo Alto Medical Foundation	California	Natalia Colocci	Sutter Health IRB
Sutter Institute for Medical Research	California	Charles McDonnell	Sutter Health IRB

Organization name	State/Province	Principal investigator name	IRB name
Texas Oncology, PA (West)	Texas	Srikar Malireddy	USOR IRB
Texas Oncology, PA (Plano West)	Texas	Carlos Taboada	USOR IRB
Texas Oncology, PA (Plano East)	Texas	Carlos Taboada	USOR IRB
Texas Oncology, PA (Tyler)	Texas	Donald Richards	USOR IRB
University Health Network, Princess Margaret Cancer Centre	Ontario	Raymond Kim	University Health Network Research Ethics Board
University of Illinois	Illinois	Vijayakrishna Gadi	UIC Research
University of Oklahoma	Oklahoma	Jerry Jaboin	Advarra Institutional Review Board
University of Pittsburgh	Pennsylvania	Joe Tung	Advarra Institutional Review Board
University of Texas Health Science Center at San Antonio	Texas	Ramon Cancino	Advarra Institutional Review Board
Virginia Commonwealth University Massey Comprehensive Cancer Center	Virginia	Andrew Poklepovic	Advarra Institutional Review Board
Weill Cornell Medicine	New York	Cora Sternberg	Biomedical Research Alliance of New

Organization name	State/Province	Principal investigator name	IRB name
			York IRB
Woodlands Medical Specialists, PA	Florida	Michael Poiesz	USOR IRB

IRB, Institutional Review Board.

Supplementary Table S20. Important Protocol Deviations.

Important protocol deviation category	Consented with cleaned data (n=35,850)
**Participants with ≥1 important protocol deviations, n (%)**	1922 (5.4)
Clinically ineligible participants excluded from analysis,[a] n	146
Clinically nonevaluable participants excluded from analysis,[b] n	21
**Participants with ≥1 important protocol deviations who were included in the analysis, n (%)**	1755 (4.9)
Participants with a negative MCED test result communicated >30 days after blood draw, n	1671
Participants with a positive MCED test result communicated >30 days after blood draw,[c] n	6
Participants whose informed consent was not documented per protocol, n	43
Other,[d] n	35

[a]Clinically ineligible participants did not meet the inclusion and/or exclusion criteria.

[b]Clinically nonevaluable participants were mainly excluded due to incomplete blood collection.

[c]All participants with a positive MCED test result and a delayed test result communication had results returned within 50 days after blood draw.

[d]Other includes blood draw completed out of window and deviation from protocol-specified activities.

Supplementary Table S21. CSO Prediction–Guided Diagnostic Evaluation Recommendations.

CSO prediction label	Possible cancer types	Recommended diagnostic evaluation[a]
Anus	Anus	Anoscopy or sigmoidoscopy
Bladder, Urothelial Tract	Bladder, Renal Pelvis, Ureter, Urethra	Flexible cystoscopy and CT urogram or retrograde pyelogram with contrast
Bone and Soft Tissue	Skeletal Muscle and Other Connective Tissue, Vascular<br>Tissue, Bone and Cartilage	MRI with contrast or CT of chest, abdomen, pelvis or PET-CT
Breast	Breast	Diagnostic mammography<br>MRI if participant has recently had screening mammogram
Cervix	Cervix	Cytology, HPV testing, colposcopy
Colon, Rectum	Colon, Rectum, Appendix	Colonoscopy
Head and Neck	Oropharynx, Hypopharynx, Nasopharynx, Larynx, Lip and Oral Cavity (including Oral Tongue), Nasal Cavity, Paranasal Sinuses, Major Salivary Glands	Flexible nasal endoscopy and Head and Neck CT or MRI
Kidney	Kidney	Triple phase renal CT
Liver, Bile Duct	Liver, Intrahepatic Bile Duct	Triple phase abdominal CT, AFP
Lung	Lung, Bronchus	Chest CT
Lymphoid Lineage	Lymphoid leukemia<br>Lymphoma	CBC with differential, peripheral blood smear,

CSO prediction label	Possible cancer types	Recommended diagnostic evaluation[a]
		LDH, hepatitis B and C serology, CMP<br>Bone marrow biopsy after blood tests if needed<br>CT of chest, abdomen, pelvis with contrast or PET-CT if needed
Melanocytic Lineage	Melanoma	Full body skin exam
Myeloid Lineage	Myeloid neoplasm	CBC with differential, peripheral blood smear, LDH, uric acid, CMP<br>Bone marrow biopsy after blood tests if needed
Neuroendocrine Cells of Lung or Other Organs	Neoplasms arising from cells of neuroendocrine lineage in the lung and high-grade cancer arising from neuroendocrine lineage in all body regions	CT of chest with or without contrast, CT of abdomen, pelvis, head & neck
Ovary	Ovary, Fallopian Tube, Primary Peritoneum	Transvaginal ultrasound and CA-125
Pancreas, Gallbladder	Pancreas, Extrahepatic Bile Duct, Gallbladder	Pancreas CT protocol or MRCP
Plasma Cell Lineage	Plasma cell neoplasm	CBC with differential, peripheral blood smear, LDH, uric acid, beta-2-microglobulin, creatinine clearance, serum immunoglobulins, serum free light chain assay, serum and urine protein electrophoresis, CMP<br>Bone marrow biopsy after blood and urine tests if needed<br>Whole-body low-dose CT or PET-CT if needed

CSO prediction label	Possible cancer types	Recommended diagnostic evaluation[a]
Prostate	Prostate	PSA and multi-parametric MRI of the prostate
Stomach, Esophagus	Stomach, Esophagus	Upper endoscopy
Thyroid	Thyroid Gland	Ultrasound of thyroid
Uterus	Uterus	Transvaginal ultrasound

[a]Additional evaluations recommended for all CSOs: CBC with differential test and CMP (includes albumin, blood urea nitrogen, calcium, carbon dioxide, chloride, creatinine, glucose, potassium, sodium, total bilirubin, total protein, alanine aminotransferase, alkaline phosphatase, aspartate aminotransferase).

AFP, alpha-fetoprotein; CBC, complete blood count; CMP, comprehensive metabolic panel; CSO, cancer signal origin; CT, computed tomography; HPV, human papillomavirus; LDH, lactate dehydrogenase; MRCP, magnetic resonance cholangiopancreatography; MRI, magnetic resonance imaging; PET, positron emission tomography; PSA, prostate-specific antigen.

Supplementary Table S22. Predefined Cancer Subgroups.

Cancer type	Cancers responsible for 2/3 of all cancer deaths in the US[a]	Aggressive cancers with low 5-year survival	Cancers without common screening options[b]	Solid cancers with common screening options[b]	Solid cancers without common screening options[b]	Hematologic malignancies
Anus	X		X		X	
Bladder or urothelial tract	X		X		X	
Bone or soft tissue sarcoma			X		X	
Breast				X		
Cervix				X		
Colon or rectum	X			X		
Esophagus	X	X	X		X	
Head and neck	X		X		X	
Kidney			X		X	
Liver or intrahepatic bile duct	X	X	X		X	
Lung	X	X	X		X	
Lymphoid lineage	X		X			X

Cancer type	Cancers responsible for 2/3 of all cancer deaths in the US[a]	Aggressive cancers with low 5-year survival	Cancers without common screening options[b]	Solid cancers with common screening options[b]	Solid cancers without common screening options[b]	Hematologic malignancies
**Myeloid lineage**			X			X
**Ovary or fallopian tube**	X	X	X		X	
**Pancreas, extrahepatic bile duct, or gallbladder**	X	X	X		X	
**Plasma cell lineage**	X		X			X
**Prostate**				X		
**Skin (excluding BCC/SCC)**			X		X	
**Stomach**	X	X	X		X	
**Thyroid**			X		X	
**Uterus**			X		X	
**Other[c]**			X		X	

[a]Data source for cancers responsible for two-thirds of all cancer deaths in the US: SEER*Stat Database: Mortality - All Causes of Death, Total US, 2018–2019, released June 2022, underlying data provided by National Center for Health Statistics: https://seer.cancer.gov/mortality/.

[b]Cancers with common screening options include cancers of the breast, cervix, colon or rectum, and prostate. For these cancers, population-wide cancer screening is recommended by several medical societies, and there is an age-based A, B, or C recommendation for screening made by the USPSTF. Lung cancer is not included in this definition because screening recommendations apply to a subset of individuals who are at high risk for this cancer due to their smoking history. Source: https://www.uspreventiveservicestaskforce.org/uspstf/.

[c]Cancers that are not included in the defined cancer types are reported under the “Other” category.

BCC, basal cell carcinoma; SCC, squamous cell carcinoma; USPSTF, United States Preventive Services Task Force.

Supplementary Table S23. Summary of PATHFINDER 2 Endpoints, Analysis Sets, and Statistical Methods.

Objective	Endpoint	Analysis set[a] and statistical method[b]	Location
**Primary Objectives**			
Evaluate performance of the MCED test in individuals eligible for cancer screening	Test performance endpoints include PPV, NPV, sensitivity, specificity, false-positive rate, positive likelihood ratio, negative likelihood ratio, overall CSO accuracy, observed cancer detection rate, number needed to screen to detect a cancer, MCED cancer detection rate, MCED cancer signal detection rate, accuracy of a CSO prediction by clinical CSO, and precision of a CSO prediction by CSO prediction. These endpoints are planned to be evaluated based on cancer status at multiple timepoints, including diagnostic resolution, 1 year, 2 years, and 3 years of follow-up.	**Analysis set**: Performance analysis set<br><br>**Statistical method**: Performance measures calculated using the 2×2 contingency table, with each calculation described in the **Supplemental Glossary of Screening Terms**.<br>Proportions are presented with two-sided 95% Wilson (score) CI for endpoints not near 0 or 1, including PPV, sensitivity, false-positive rate, positive likelihood ratio, negative likelihood ratio, CSO accuracy, observed cancer detection rate, number needed to screen to detect a cancer, MCED cancer detection rate, and MCED cancer signal detection rate; modified Wilson (score) CIs are used for endpoints near 0 or 1, including specificity and NPV.	Results Performance subsection; Table 2
Evaluate the safety of the MCED test in terms of diagnostic testing triggered by	Safety endpoints include the number and type of invasive procedures performed and the number and type of adverse events due to diagnostic testing in all participants with a positive MCED test result.	**Analysis set**: Safety analysis set<br><br>**Statistical method**: Descriptive summaries are reported for invasive procedures conducted during diagnostic testing prompted by a positive MCED test result, including the number of participants with ≥1 invasive procedure, the total number of	Results Safety subsection; Table 3; Figure 2A; Supplemental Tables S2-S6, S8, and S9

Objective	Endpoint	Analysis set[a] and statistical method[b]	Location
the MCED test result	Safety measures describing the use of invasive procedures include:<br>A) Number of participants with a positive MCED test result who undergo an invasive procedure divided by the total number of analyzable participants<br>B) Number of invasive procedures among participants with a positive MCED test result divided by the total number of analyzable participants<br>C) Number of participants with a positive MCED test result and an invasive procedure divided by the total number of participants with a positive MCED test<br>D) Number of invasive procedures among participants with a positive MCED test result divided by the number of participants	invasive procedures, and the distribution of per-participant procedure counts, overall and by procedure type: surgical and nonsurgical. Analyses are summarized overall and by subgroup, including cancer versus no cancer diagnosis, initial versus subsequent diagnostic evaluation, targeted evaluation versus evaluation after diagnostic PET-CT, and CSO-guided versus not CSO-guided diagnostic evaluation.<br>Safety measures A-D reported as proportions with two-sided 95% Wilson (score) CIs.	

Objective	Endpoint	Analysis set[a] and statistical method[b]	Location
	with a positive MCED test<br><br>Similar analyses are planned for participants with true-positive and false-positive MCED test results separately.		
**Secondary Objectives**			
Assess participant-reported anxiety resulting from use of the MCED test	Anxiety assessed by STAI + MCED-STAI questionnaires pre-test (baseline), post-test, at diagnostic resolution (if applicable), and at 1 year (+30D).	**Analysis set**: Analyzable set<br><br>**Statistical method**: STAI-6 total scores were calculated according to the STAI manual and summarized descriptively using mean and SD at each assessment timepoint. The present manuscript reports descriptive summaries of total scores only; no formal hypothesis testing was performed.<br><br>Change in the total score from baseline at post-test, diagnostic resolution and 12 months will be assessed. A two-sided t-test will be used to determine if mean changes from baseline are significantly different from zero at each post-baseline assessment at 0.05 significance level.	Results Participant-Reported Outcomes subsection; Extended Data Figure 6<br><br>Changes in the total score from baseline are not included in the present manuscript; no formal hypothesis testing was performed
Evaluate intention and utilization of guideline-	Intention to follow standard-of-care cancer screening tests assessed pre-test, post-test, and at 1 year (+30D).	**Analysis set**: Analyzable set	This endpoint is not reported in the present manuscript

Objective	Endpoint	Analysis set[a] and statistical method[b]	Location
recommended cancer screening procedures after use of the MCED test	Utilization of standard-of-care cancer screening tests prior to study enrollment, during the first year, and the second year (±30D) after MCED test.	**Statistical method**: The number and proportion of participants in each response category were reported at each timepoint by analysis set descriptively.	
Describe the ability of confirmatory PET-CT to detect cancer in participants where diagnostic resolution of cancer diagnosis is not achieved through the targeted diagnostic testing triggered by MCED test results	The endpoints include:<br>• Proportion of participants with cancer diagnosis at diagnostic resolution out of all participants who had a PET-CT only after initial negative diagnostic evaluation (no PET-CT during initial diagnostic evaluation).<br>• Proportion of participants with cancer diagnosis at diagnostic resolution out of all participants with initial negative diagnostic evaluation who had a diagnostic PET-CT at any time during diagnostic evaluation.	**Analysis set**: Safety analysis set<br><br>**Statistical method**: Proportions are summarized descriptively.	Results Use of Diagnostic PET-CT subsection; Supplemental Table S13
Characterize the number and type of diagnostic evaluations by predicted	Number and type of imaging procedures; number and type of invasive procedures; number and type of laboratory tests; time to diagnostic resolution.	**Analysis set**: Safety analysis set<br><br>**Statistical method**: Descriptive statistics; Kaplan-Meier method for time to diagnostic resolution.	Results Time to Diagnostic Resolution subsection; Figure 2B

Objective	Endpoint	Analysis set[a] and statistical method[b]	Location
cancer signal origin and outcome of diagnostic resolution			
Evaluate the radiation exposure during diagnostic evaluation	Number and type of imaging procedures with radiation exposure and total per-participant radiation exposure during diagnostic evaluation.	**Analysis set**: Safety analysis set<br><br>**Statistical method**: Descriptive statistics.	This endpoint is not reported in the present manuscript
Evaluate concordance of initial test result and CSO prediction with results from the research blood draw[c]	Concordance between initial MCED-V2 test results and research blood draw results, including agreement in cancer signal detection and concordance of CSO1 prediction among participants with both test results.	**Analysis set**: Analyzable set with a research blood draw<br><br>**Statistical method**: Initial and research blood draw results are compared using 2×2 matrices overall and by cancer diagnosis status, with PPA, NPA, OPA, and corresponding CIs reported. CSO1 prediction concordance is summarized descriptively using a prediction matrix and the proportion of participants with the same CSO1 prediction across both blood draws.	This endpoint is not reported in the present manuscript
Assess participants' reported outcomes and perceptions about the MCED test	Participant-reported outcomes and perceptions of the MCED test, assessed using participant-directed questionnaires, including health-related quality of life and	**Analysis set**: Analyzable set<br><br>**Statistical method**: Descriptive statistics for adapted MICRA, satisfaction, and attitudes toward subsequent testing; for SF-12v2 changes from baseline are assessed	This endpoint is not reported in the present manuscript

Objective	Endpoint	Analysis set[a] and statistical method[b]	Location
	other questionnaire-specific scores or item responses.	using two-sided t-test, with Hochberg adjustment for multiple testing.	
Evaluate performance of the MCED test in selected demographic and clinical subgroups	Primary test performance endpoints assessed in selected demographic and clinical subgroups classified on the basis of different categories of age, sex, race, ethnicity, BMI, smoking history, prior cancer history, and genetic cancer predisposition.	**Analysis set**: Performance analysis set<br><br>**Statistical method**: Proportions with 95% Wilson/modified Wilson CIs.	Results Performance in Subgroups subsection; Supplemental Tables S10-S12<br>Race, ethnicity, BMI, and cancer-history subgroups are not reported in the present manuscript
Evaluate MCED test positive rate in subgroups with potential cross-reactive conditions	MCED test positive rate among participants with potentially cross-reactive conditions.	**Analysis set**: Analyzable set<br><br>**Statistical method**: MCED test positive rates are compared between participants with versus without each potentially cross-reactive condition using Pearson chi-square tests.	This endpoint is not reported in the present manuscript
**Exploratory Objectives**			
Evaluate performance of the MCED test over three years of follow-up	MCED test performance over up to 3 years of follow-up, including cancer signal detection, CSO accuracy, and distribution of cancer types/stages in the final 12 months of follow-up.	**Analysis set**: Performance analysis set<br><br>**Statistical method**: Proportions with 95% Wilson/modified Wilson CIs.	This endpoint is not reported in the present manuscript
Evaluate changes in blood-based	Changes in blood-based biomarker signals between	**Analysis set**: Safety analysis set with a research blood draw	This endpoint is not reported in the present manuscript

Objective	Endpoint	Analysis set[a] and statistical method[b]	Location
biomarkers among the subset of participants with a research blood draw[c]	initial and research blood draws.	**Statistical method**: Descriptive statistics.	
Evaluate different CSO reporting classes based on clinically relevant subgroups	CSO prediction reporting classes based on clinically relevant subgroups.	**Analysis set**: Analyzable set<br><br>**Statistical method**: Descriptive statistics.	This endpoint is not reported in the present manuscript

[a]All analysis sets refer to participants tested with MCED-V2; definitions are included in the Analysis Sets section and in the statistical analysis plan.

[b]No prespecified study success criteria were defined for the endpoints reported in this manuscript; all endpoints were prespecified in the protocol and statistical analysis plan.

[c]A research blood draw was collected only in participants with a positive initial MCED test result, around the time of diagnostic PET-CT in cases where no cancer was identified by targeted diagnostic procedures. Results from the research blood draw were not returned to investigators or participants and were used for prespecified secondary analyses of concordance with the initial blood draw.

BMI, body mass index; CSO, cancer signal origin; MCED, multi-cancer early detection; MICRA, Multidimensional Impact of Cancer Risk Assessment; NPA, negative percent agreement; NPV, negative predictive value; PET-CT, positron emission tomography–computed tomography; OPA, overall percent agreement; PPA, positive percent agreement; PPV, positive predictive value; SF-12v2, Short Form-12 Health Survey version 2; STAI, State-Trait Anxiety Inventory.

Supplementary Table S24. Protocol and Statistical Analysis Plan Amendments.

Document	Version	Date	Summary of changes
Protocol	1.0	09 Jun 2021	Initial version
Protocol	2.0	28 Feb 2022	The protocol was amended to reflect changes to the study design, including the sample size and target population, to update exclusion criteria, to clarify aspects of the collection of blood and clinical data, and to update the definition of cancer used in the study. Specific changes:<br>● Section 3.2 Background of the Study Test: added NHS-Galleri as an ongoing study.<br>● Section 4 Objectives & Endpoints: updated definition of cancer.<br>● Section 5.1 Cancer Signal Origin Directed Diagnostic Evaluations: updated Recommended Workup.<br>● Section 5.2 Estimated Study Duration: updated to 4.5 years.<br>● Section 6 Study Population: updated Race Table superscript and added ethnicity to Hispanic.<br>● Section 6.2 Exclusion Criteria: added clarifying language that non-metastatic basal cell carcinoma and squamous cell carcinoma of the skin are not exclusion criteria.<br>● Section 6.2 Exclusion Criteria: added pregnancy exclusion.<br>● Section 7.2.1 Active Clinical Follow-Up: updated start of active follow-up from date of enrollment to date of blood draw.<br>● Section 9.3 Blood Draw: specified minimum number of tubes is 3, with at least 3 mL in each tube.<br>● Section 9.5 Diagnostic Work-Up of a "Cancer Signal Detected" Test Result: updated cancer diagnosis definition.<br>● Section 9.6 Clinical Data Collection: updated clinical data collection for individuals with a cancer signal detected test result.

Document	Version	Date	Summary of changes
			● Section 9.8.3 Adverse Device Effects (ADEs): clarified what is considered an ADE; added "other medically important serious event" to SAE criteria.<br>● Section 10.2 Sample Size Determination: updated determination based on enrollment of 20,000 subjects.<br>● Section 10.4.5 Interim Analyses: updated the timing of interim analyses.<br>● Appendix I(b) Financial Disclosure: removed "Financial compensation will not be provided to participants in this study."<br>● Appendix II Examples of Imaging and Invasive Tests: added additional examples of imaging and invasive tests.<br>● Throughout document: replaced Data Safety Monitoring Board (DSMB) with Independent Data Monitoring Committee (IDMC).<br>● Throughout document: updated GRAIL, Inc. to GRAIL, LLC.<br>● Throughout document: increased study enrollment to 20,000 subjects (at least 200 cancer signal detected MCED test results), enrollment period to 18 months, and number of institutions to 40.<br>● Throughout document: replaced Galleri with GRAIL's multi-cancer early detection (MCED) test.
Protocol	2.1	01 Apr 2022	Clarified adverse event definition.
Protocol	3.0	09 Aug 2023	The protocol amendment was undertaken primarily to increase the study size to at least 35,000 and no more than 38,500 participants in order to ensure adequate data on safety and efficacy for clinical validation. Additional changes were implemented to refine the age targets for enrollment to better match the study objectives and intended use. Specific changes:<br>● Section 4 Secondary Analysis Objectives and Endpoints: typographical error corrected; number of secondary study objectives corrected from six to seven.

Document	Version	Date	Summary of changes
			• Section 5.1 Overall Design: HPV testing added to recommended procedures for diagnostic evaluation of CSO Cervix.<br>• Section 5.2 Estimated Study Duration: updated to 6.75 years.<br>• Section 6 Study Population: age targets changed to 50-59: 25%; 60-69: 47.5%; 70-79: 25%; 80+: 2.5%, split evenly between men and women.<br>• Section 8.1 Participant Withdrawal From Study: clarified that participants may be withdrawn from the study if they cannot be reached to have their test results returned within 90 days.<br>• Section 8.1 Participant Withdrawal From Study: clarified that once withdrawn, participants are not eligible to re-enroll in the study.<br>• Section 8.1 Participant Withdrawal From Study: language added to clarify that failure to perform a repeat blood draw, when required, within 60 days following consent will result in withdrawal of the participant.<br>• Section 9.3 Blood Draw and Section 9.6 Clinical Data Collection: Personal Health Information updated to Protected Health Information.<br>• Section 10.2 Sample Size Determination: statistical section updated to reflect the increased study size.<br>• Section 10.4.2 Primary Analyses: updated safety endpoints to include the number and type of invasive procedures and the number and type of adverse events due to diagnostic testing in all participants with a positive MCED test result. Clarified that analyses will be conducted overall and for each CSO label and will include analysis for cancer detection only as well as combined cancer detection and CSO prediction.<br>• Section 10.4.3 Secondary Analyses: time required to achieve diagnostic resolution defined as the number of days from when the test result is communicated to the participant.<br>• Appendix II: "Type of Diagnostic Testing" column added.

Document	Version	Date	Summary of changes
			● Appendix IV: described additional consideration used in simulations.<br>● Throughout document: study expanded to at least 35,000 and no more than 38,500 participants; enrollment period extended to 45 months.
Protocol	4.0	21 Jan 2025	The protocol was amended to align with the SAP, remove tissue collection activities, and clarify use of the updated MCED test. Specific changes:<br>● Throughout document: updated GRAIL, LLC to GRAIL, Inc.<br>● Throughout document: removed exploratory objective of evaluating DNA-based and other biomarkers in tissue compared to blood from cancer cases and all mention of tissue collection activities. Aligns with the change in study design not to collect tissue specimens from participants with cancer diagnosis.<br>● Throughout document: clarified the use of the updated version of the MCED test on participant samples and that any new test results obtained will not be returned to study investigators or participants.<br>● Section 10.4.2 Primary Analysis: included description of additional test performance endpoints. Aligned with the test performance analysis detailed in the SAP.<br>● Section 10.4.3 Secondary Analysis: added two additional secondary analysis objectives: assessing test performance in select demographic and clinical subgroups, and evaluating the effect of certain medical conditions and clinical subgroups on MCED test positive rate.
SAP	1.0	13 May 2024	Initial version
SAP	2.0	07 Feb 2025	Specific changes from SAP v1.0:<br>● Added the description and analysis methods for MCED-I cancer biology signal prediction (Section 6.3, Section 8.1, Section 8.2, Section 14.2.2.1).

Document	Version	Date	Summary of changes
			● Updated and clarified the definition of cancer types, CSO and CBS (Appendices 3B, 6, 8).<br>● Updated the acceptance criteria to study success criteria, with a simplified hierarchical testing. Clarified the study success criteria based on the lower bound of the two-sided 95% confidence interval (Section 3.1, Section 14.2.2.2, Section 17). The success criteria apply to the investigational MCED-I test for PMA analysis and are not applicable to the MCED-V2 endpoints reported in this manuscript.<br>● Updated safety and diagnostic evaluation analyses to focus on MCED-V2 (Section 14.2.1, Section 14.3.2).<br>Removed exploratory objective related to evaluation of DNA-based and other biomarkers in tissue because tissue collection was discontinued (Section 3.3, Section 11, Section 14.4).

ADE, adverse device effect; CBS, cancer biology signal; CI, confidence interval; CSO, cancer signal origin; DSMB, Data and Safety Monitoring Board; HPV, human papillomavirus; MCED, multi-cancer early detection; MCED-I, the MCED test version that was analyzed to support an FDA PMA submission; MCED-V2, the version of the MCED test used in PATHFINDER 2; PMA, premarket approval; SAE, serious adverse event; SAP, statistical analysis plan; SF-12v2, Short Form-12 version 2 Health Survey; STAI, State-Trait Anxiety Inventory.

Protocol and Statistical Analysis Plan

This supplement contains the following items:

1. Original protocol, final protocol, summary of changes

2. Original statistical analysis plan, final statistical analysis plan, summary of changes

Clinical Study Protocol

PATHFINDER 2

Clinical Study Protocol Title: The PATHFINDER 2 Study: Evaluating the Safety and Performance of the GRAIL Multi-Cancer Early Detection Test in an Eligible Screening Population

Clinical Study Protocol Number: GRAIL-012

Version Number: 1.0

Version Date: 09-JUN-2021

Sponsor Name: GRAIL, Inc.

Sponsor Address: 1525 O'Brien Drive, Menlo Park, CA 94025

Sponsor Phone Number: (833) 694-2553

Medical Monitor: [redacted]

Medical Director Lead: [redacted]

Medical Monitor Contact Information: [redacted]

FDA Investigational Device Exemption (IDE) Application Number: TBD

Sponsor Approvals

This clinical study protocol has been approved by GRAIL, Inc. (GRAIL). The following signature(s) document this approval.

[redacted]

6/10/2021

Date

Medical Director Lead
GRAIL, Inc.

[redacted]

6/9/2021

Date

Clinical Operations Lead
GRAIL, Inc.

[redacted]

6/10/2021

Date

Regulatory Affairs
GRAIL, Inc.

Clinical Study Protocol

Sponsor Approvals

This clinical study protocol has been approved by GRAIL, Inc. (GRAIL). The following signature(s) document this approval.

[redacted]

Clinical Data Manager
GRAIL, Inc.

6/10/2021
Date

[redacted]

Biostatistical Director
GRAIL, Inc.

6/10/2021
Date

[redacted]

Clinical Program Manager
GRAIL, Inc.

6/9/2021
Date

Confidentiality

The information contained in this document is the property of or under control of GRAIL, and is provided to you in confidence. Except as required by federal, state, or local laws or regulations, this document may not be disclosed to others, in whole or in part, without the prior written permission of GRAIL.

Statement of Good Clinical Practice Compliance

The study will be conducted in accordance with the International Council for Harmonization of Technical Requirements for Pharmaceuticals for Human Use (ICH) - ICH Harmonized Guideline - Integrated Addendum to ICH E6(R1): Guideline for Good Clinical Practice E6(R2) (ICH/GCP), and the United States Department of Health & Human Services, Food & Drug Administration (FDA) - Code of Federal Regulations (CFR), Title 21 - Food & Drugs, Chapter I - Food & Drug Administration, Department of Health & Human Services, Subchapter A - General, Part 11 - Electronic Records: Electronic Signatures (21 CFR 11); Part 50 - Protection of Human Subjects (21 CFR 50); Part 54 - Financial Disclosure by Clinical Investigators (21 CFR 54); Part 56 - Institutional Review Boards; Subchapter H - Medical Devices, Part 812 - Investigational Device Exemption (21 CFR 812). All personnel involved in the conduct of this clinical study have completed human subjects protection training.

Principal Investigator Signature Page

The signature below constitutes the approval of this clinical study protocol (protocol) and the attachments, and provides the necessary assurances that this clinical study will be conducted according to all stipulations of the protocol, including all statements regarding confidentiality, and according to applicable federal, state, and local requirements including regulations administered by the Food & Drug Administration (FDA) (such as of 21 CFR Part 50 and 21 CFR Part 812) and International Conference on Harmonization of Technical Requirements for Registration of Pharmaceuticals for Human Use (ICH) guidelines. The Investigator also agrees to conduct the investigation in accordance with the investigational plan and any conditions of approval imposed by the reviewing Institutional Review Board (IRB)/Research Ethics Board (REB) or Food & Drug Administration (FDA). In addition, the Investigator agrees to supervise all testing of the device involving human subjects and ensure that the requirements for obtaining informed consent are met.

Principal Investigator Name: ____

Principal Investigator Title: ____

Principal Investigator Signature: _____________________________________

Date: ____

Table of Contents

Revision History **9**

Protocol Summary **10**
- Clinical Study Protocol Synopsis 10
- Schema 14
- Schedule of Activities (SOA) 15

Introduction **17**
- Study Rationale 17
- Background of the Study Test 18
- Benefit/Risk Assessment 20

Objectives & Endpoints **22**

Primary Analysis Objectives and Endpoints **22**

Study Design **25**
- Overall Design 25
- Estimated Study Duration 29
- Study Duration for Study Participants 29
- Study Design Schema 29
- Scientific Rationale for Study Design 29
- Study Enrollment Definition 29
- End-of-Study Definition 29

Study Population **30**
- Inclusion Criteria 30
- Exclusion Criteria 31
- Participant Recruitment 31
- Informed Consent Process 31

Measures to Minimize Bias **33**
- Blinding Methods 33
- Participant Follow-Up 33
  - Active Clinical Follow-Up 33
- Participant Retention Through Follow-Up 34

Participant Withdrawal **34**

Participant Withdrawal from the Study 34
Lost to Follow-Up 35

Study Assessments & Procedures 35
Enrollment 36
Participant Questionnaire 36
Blood Draw 36
Return of Results 37
Diagnostic Work-Up of a “Cancer Signal Detected” Test Result 38
Clinical Data Collection 39
Tissue Sample Collection 40
Safety Assessments 41
General 41
Adverse Events (AEs) and Serious Adverse Events (SAEs) 41
Adverse Device Effects (ADEs) 42
Unanticipated Adverse Device Effects (UADEs) 42
Follow-Up of ADEs & UADEs 43
Regulatory Reporting Requirements for UADEs 43
Test Deficiencies and Concerns with the Test 43
Study or Study Site Closure, Termination and Suspension 43
Healthcare Resource Utilization 44

Statistical Considerations 44
Analysis Methods 44
Populations for Analyses 46
Statistical Analyses 47
General Considerations 47
Primary Analyses 47
Secondary Analyses 49
Safety Assessments 52
Interim Analyses 53

Data & Safety Monitoring Board (DSMB) 53

Supporting Documentation & Operational Considerations 54
Appendix I(a). Regulatory & Ethical Considerations 55
Appendix I(a). Regulatory & Ethical Considerations 56
Appendix I(b). Financial Disclosure & Participant Compensation 57

Appendix I(c). Data Protection 58
Appendix I(d). Study Supplies 59
Appendix I(e). Source Documents 60
Appendix I(f). Study Data Quality Assurance (QA) 61
Appendix I(g). Record Retention 62
Appendix I(h). Publication Policy 63
Appendix II. Examples of Imaging and Invasive Tests 64
Appendix III. Cancer Screening Compliance 65
Appendix IV. Description of the Simulations 66
Appendix V. Abbreviations 67
Appendix VI. References 70
Appendix VII. Clinical Study Protocol Amendment History 72

1. Revision History

Refer to Appendix VII, *Clinical Study Protocol Amendment History* for details on protocol version history.

Version	Date Revised DD-MMM-YYYY	Revised By	Description of Revision
**1.0**	**09-JUN-2021**		**Initial Version**

2. Protocol Summary

2.1. Clinical Study Protocol Synopsis

Protocol Title: The PATHFINDER 2 Study: Evaluating the Safety and Performance of the GRAIL Multi-Cancer Early Detection (MCED) Test in an Eligible Screening Population

Protocol Short Title: PATHFINDER 2

Rationale: The purpose and rationale is to evaluate the safety and performance of the GRAIL Multi-Cancer Early Detection (MCED) Test in an eligible screening population.

Table 1. Objectives & Endpoints Table

Objectives	Endpoints
**Primary**	
1. Evaluate the safety of the MCED test in terms of diagnostic testing triggered by the MCED test result.<br><br>2. Evaluate performance of the MCED Test in individuals eligible for cancer screening.	1. Safety endpoints: Number and type of invasive procedures (as described in Appendix II) performed among false positive (FP) participants.<br><br>The safety measures will describe the use of invasive procedures and include the following:<br><br>Main measure:<br>○ Measure A: Number of FP participants with an invasive procedure divided by the total number of analyzable participants.<br><br>Secondary measures:<br>○ Measure B: Number of invasive procedures among FP participants divided by the total number of analyzable participants.<br>○ Measure C: Number of FP participants with an invasive procedure divided by the total number of participants with a positive MCED test<br>○ Measure D: Number of invasive procedures among FP participants divided by the number of participants with a positive MCED test.<br><br>2. Test performance endpoint: Diagnosis of invasive cancer.<br><br>The MCED test performance measures include positive predictive value (PPV), negative predictive value (NPV), sensitivity, specificity, cancer signal origin (CSO) accuracy,

	observed cancer detection rate (CDR), and number needed to screen to detect a cancer (NNS) and will be evaluated based on cancer status at multiple timepoints (e.g. at diagnostic resolution, 1 year, 2 years).
**Secondary**	
1. Assess participant-reported anxiety resulting from use of the MCED test<br><br>2. Evaluate intention to use, and utilization of, guideline recommended cancer screening procedures after use of the MCED test.<br><br>3. Describe the ability of PET-CT to detect cancer in cases for which cancer signal origin-directed workups do not result in diagnosis of cancer (initial workup negative cases).<br><br>4. Characterize the extent of diagnostic testing triggered by the MCED test result by predicted cancer signal origin and outcome of diagnostic resolution.<br><br>5. Evaluate the radiation exposure during	1. Anxiety will be assessed by State Trait Anxiety Inventory (STAI)+ MCED STAI questionnaires pre-test (baseline), post-test, at diagnostic resolution (if applicable), and at 1 year (+/-30D). Changes from the baseline will be evaluated at each time point.<br><br>2. Intention to follow standard of care cancer screening tests will be assessed pre-test, post-test, at 1 year (+/-30D). Changes from the baseline will be evaluated at each time point. Utilization of standard-of-care cancer screening tests prior to study enrollment, during the first year and the second year (+/-30D) after the initial MCED test will be assessed. Utilization during the study will be compared to that in a period prior to enrollment.<br><br>3. Diagnosis of invasive cancer after confirmatory PET-CT administered in participants where diagnostic resolution of cancer diagnosis is not achieved through the targeted diagnostic testing. Observed cancer detection rate will be calculated.<br><br>4. Number and type of diagnostic procedures, and time to diagnostic resolution, will be collected. The extent of diagnostic testing will be assessed across all test positive participants, FP participants, and true positive (TP) participants. The results will be reported for all diagnostic testing prior to diagnostic resolution, the targeted diagnostic testing triggered by MCED test result and conducted prior to confirmatory PET-CT (if applicable), and for diagnostic testing starting from confirmatory PET-CT and conducted until diagnostic resolution.<br><br>5. Per participant radiation exposure during diagnostic evaluation will be assessed. Distribution of radiation exposure will be evaluated across all test positive participants, FP, and TP. The results will be reported for all diagnostic testing prior to diagnostic resolution, the targeted diagnostic testing triggered by MCED test result and

diagnostic evaluation.<br>6. Evaluate concordance of initial GRAIL test result and CSO prediction, with results from the research blood draw<br>7. Assess participant-reported outcomes and perceptions about the MCED test.	conducted prior to confirmatory PET-CT (if applicable), and for diagnostic testing starting from confirmatory PET-CT and conducted until diagnostic resolution.<br>6. MCED test results for cancer detection and CSO prediction from the initial and research blood draws (in the subset of participants with a research blood draw) will be assessed. Agreement measures will be calculated.<br>7. Participant-reported outcomes will be assessed at different time points:<br>○ Health-related quality of life following the MCED test assessed by SF-12v2<br>○ Multidimensional Impact of Cancer Risk Assessment (adapted MICRA)<br>○ Satisfaction with the MCED test<br>○ Attitudes towards subsequent MCED screening<br>Changes from the baseline will be evaluated at each time point for SF-12v2.
**Exploratory**	
1. Evaluate performance of the MCED test in year 3.<br>2. Evaluate changes in blood-based biomarkers among the subset of participants with a research blood draw.<br>3. Evaluate different CSO reporting classes based on clinically relevant subgroups.<br>4. Evaluate DNA based and other biomarkers in tissue compared to blood from cancer cases.	1. Diagnosis of invasive cancer during year 3. The MCED test performance measures include positive predictive value (PPV), negative predictive value (NPV), sensitivity, specificity, cancer signal origin (CSO) accuracy, observed cancer detection rate (CDR), and number needed to screen to detect a cancer (NNS) and will be measured based on cancer status at year 3.<br>2. Blood biomarker signals from the initial and research blood draws in the subset of participants with a research blood draw will be assessed.<br>3. MCED test results for CSO prediction and blood biomarker signals from the initial and research blood draws will be evaluated in the context of diagnostic testing triggered by the test results.<br>4. DNA based and other biomarkers in tissue and blood samples in participants with cancer.

Overall Design: This is a prospective, multi-center, interventional study of the GRAIL MCED Test with return of results in healthcare systems in North America.

The study will enroll at least 10,000 participants, or as many as required to achieve at least 100 cancer signals detected MCED test results returned to the healthcare providers, as defined by eligibility criteria over an anticipated enrollment period of approximately 10 months. Following informed consent, the participant will undergo the GRAIL MCED Test. Test results will be returned to the healthcare providers and information on the ensuing diagnostic work-up will be captured. Participant disposition will be categorized into the following groups:

- GRAIL MCED positive: Participants will undergo diagnostic procedures based on the test returned cancer signal origin(s) (CSO). Diagnostic procedures corresponding to cancer signal origin are specified in Table 4. If a participant is unable to undergo the prescribed procedure, or the institution physician determines an alternative work-up is necessary, the institution physician will document the reason for deviating from the prescribed procedure(s) and list the alternative procedure(s) performed.
    - For participants where diagnostic resolution of cancer diagnosis is achieved through the targeted diagnostic testing based on the CSO, clinical information including but not limited to cancer type, pathologic, imaging and clinical staging information will be captured.
    - For participants where diagnostic resolution of cancer diagnosis is not achieved through the targeted diagnostic testing and PET-CT has not been used as part of diagnostic evaluation, a PET-CT will be performed to assess cancer status, henceforth referred to the 'confirmatory PET-CT' for the purposes of this study. For participants where diagnostic resolution of cancer is achieved through the confirmatory PET-CT, clinical information including but not limited to cancer type, pathologic, imaging, and clinical staging information will be captured. Participants in this group will also undergo a research blood draw around the time of the confirmatory PET-CT. Results of this research blood draw will not be provided back to the physician or participant.
    - All efforts should be made to achieve diagnostic resolution within 6 months after receipt of MCED test results.
- GRAIL MCED negative: Participants will be notified of "cancer signal not detected" test results by the study investigator or designated member of the study team, be reminded not to interpret "cancer signal not detected" test results as the absence of cancer and to continue to adhere to guideline-recommended cancer screening. Participants will continue to be followed as outlined in this protocol.

All participants will be actively followed by the enrolling institution for 3 years to assess for cancer status, uptake of routine cancer screening and other data collection with analyses performed at interim time points. In addition to a baseline questionnaire, additional participant-reported outcomes will be collected at multiple time points before and after test results are returned.

A subsequent version of the test, MCED-1 will be under development during the conduct of this study. Once MCED-1 is available for use in this study, residual blood samples from participants collected at day 1 (Table 2) may be tested using this updated version. Results from this testing will be returned to the healthcare provider, and if the results are discordant with results from the original study test, the healthcare provider may recommend the participant undergo diagnostic tests for cancer. Clinical data associated with these tests, evaluations and procedures as detailed in Section 9.6, will be collected. The study informed consent form will provide participants with information on this potential additional testing.

Data Safety Monitoring Board: Yes

2.2. Schema

Figure 1. Schema

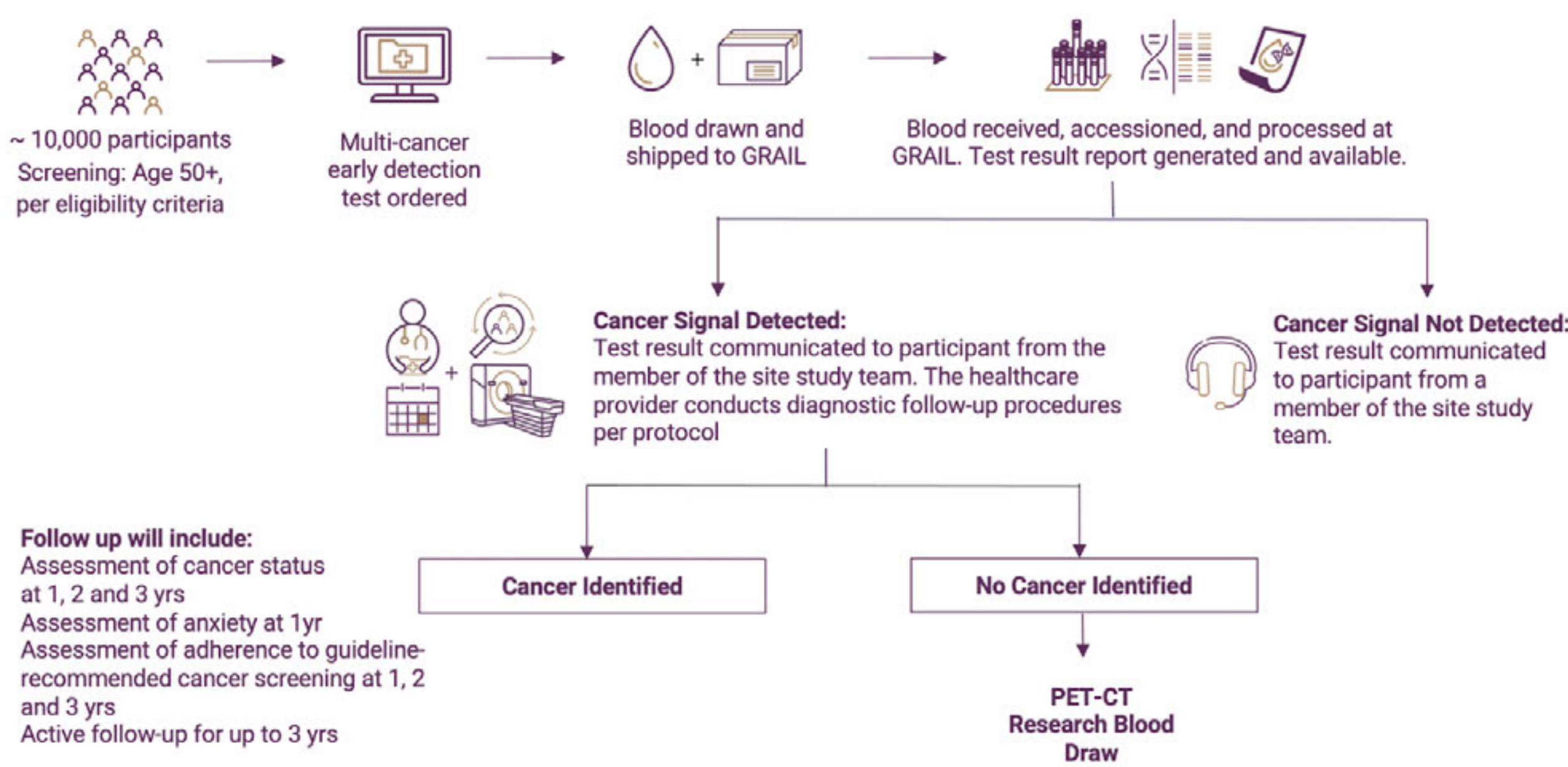

2.3. Schedule of Activities (SOA)

Table 2. Schedule of Activities Table

<table>
<tr><th colspan="2">Activity</th><th>Enrollment</th><th>Day 1</th><th>Day 15</th><th>By Day ~30</th><th>Diagnostic Evaluation</th><th>At Diagnostic Resolution</th><th>1yr +/-30D</th><th>2yr +/-30D</th><th>3yr +/-30D (EOS)</th></tr>
<tr><td colspan="2">Inclusion/Exclusion Criteria Review for Eligibility</td><td>x[c]</td><td>x[e,f]</td><td></td><td></td><td></td><td></td><td></td><td></td><td></td></tr>
<tr><td colspan="2">Informed Consent[a]</td><td>x</td><td></td><td></td><td></td><td></td><td></td><td></td><td></td><td></td></tr>
<tr><td colspan="2">Demographics and Baseline Assessment (Medical History, Cancer Screening History)</td><td colspan="2">x</td><td></td><td></td><td></td><td></td><td></td><td></td><td></td></tr>
<tr><td colspan="2">Participant Questionnaires[b]</td><td colspan="2">x[d]</td><td></td><td>x[h]</td><td></td><td>x[m]</td><td>x[n]</td><td></td><td></td></tr>
<tr><td colspan="2">Blood Draw for Study Test</td><td></td><td>x[f]</td><td></td><td></td><td></td><td></td><td></td><td></td><td></td></tr>
<tr><td colspan="2">Study Test Result Report Available to the Study Investigator and Study Team</td><td></td><td></td><td>x[g]</td><td></td><td></td><td></td><td></td><td></td><td></td></tr>
<tr><td colspan="2">Study Test Result Communicated to the Participant (“Return of Results”)</td><td></td><td></td><td></td><td>x[i]</td><td></td><td></td><td></td><td></td><td></td></tr>
<tr><td colspan="2">Cancer Status Assessment & Tissue Collection[j]</td><td></td><td></td><td></td><td></td><td></td><td></td><td>x</td><td>x</td><td>x</td></tr>
<tr><td rowspan="2">“Cancer Signal Detected” Test Result</td><td>Diagnostic Procedures Performed as Outlined in Section 5.1</td><td></td><td></td><td></td><td></td><td>x</td><td></td><td></td><td></td><td></td></tr>
<tr><td>PET-CT & Research Blood Draw</td><td></td><td></td><td></td><td></td><td>x[k]</td><td></td><td></td><td></td><td></td></tr>
<tr><td colspan="2">Assessment of Utilization of Routine Cancer Screening</td><td></td><td></td><td></td><td></td><td></td><td></td><td>x</td><td>x</td><td>x</td></tr>
<tr><td colspan="2">Study Related AE, SAE, and UADE Reporting and Review</td><td colspan="9">x[l]</td></tr>
</table>

[a]Eligibility criteria should be confirmed based on medical record review prior to obtaining informed consent. Informed consent must be obtained prior to study blood draw and any study procedures being performed.
[b]See Table 3 for additional details regarding participant questionnaires.
[c]Inclusion/exclusion criteria are confirmed prior to enrollment.
[d]Baseline/Pre-Test participant questionnaires should be completed prior to the Day 1 blood draw for the study test.

[e]Eligibility should be confirmed based on medical record review on Day 1 prior to the blood draw. For blood draw visits occurring 31-60 days following consent, eligibility criteria must be re-confirmed prior to blood draw.
[f]The Day 1 blood draw visit may occur on the same day as informed consent (enrollment visit) but may occur up to 60 days following informed consent. Repeat blood draws will be requested if technical issues with samples preclude sample processing or reporting.
[g]The test result report will be returned to the study investigator via paper or electronic methods (test results portal) typically within 15 calendar days from the date of blood collection, barring holidays and unforeseen circumstances affecting sample shipment and lab processing.
[h] Post-Return of Results participant questionnaires must be distributed no sooner than 7 days after communication of test results to the participant, and should be completed within 14 days.
[i]The test result should be communicated to the participant as soon as possible, typically within 15 days from the date of receipt of MCED test results. See Section 9.4.
[j]Sites will be requested to provide archived tumor tissue samples collected during standard of care procedures as described in section 9.7.
[k]PET-CT and research blood draw will be collected on "cancer signal detected" cases where no cancer is identified by diagnostic procedures performed as outlined in section 5.1.
[l]"Study-related" is defined as related to a study activity listed in this table. See Section 9.8 for additional information.
[m] Post-Diagnostic Resolution participant questionnaires must be distributed no sooner than 7 days after communication of diagnostic resolution to the participant, and should be completed within 14 days.
[n]1yr participant questionnaires will be triggered at 1 year (+/-30D) from the date of blood collection.

Table 3. Participant-Reported Outcomes Instruments (Questionnaires)

Instrument	Baseline/Pre-Test (Prior to blood draw)	Post-Return of Results (ROR) (7-21 days after ROR)		Post-Diagnostic Resolution (7-21 days following)	1yr +/-30D
**Participant Group**	**All (22 items)**	**Cancer Signal Not Detected (47 items)**	**Cancer Signal Detected (43 items)**	**Cancer Signal Detected (25 items)**	**All (22 items)**
SF-12v2 (12 items)	X	X	X	X	X
State Trait Anxiety Inventory 6 (STAI; 6 items) + MCED STAI (3 items)	X	X	X	X	X
Multidimensional Impact of Cancer Risk Assessment (adapted MICRA; 21 items)		X	X		
Satisfaction with the MCED test (de novo; 3 items)		X		X	
Attitude towards adherence to current cancer screening guidelines (de novo; 1 item)	X	X	X		X
Attitude towards subsequent MCED test (de novo; 1 item)		X		X	

3. Introduction

Detecting cancers early, when treatment is more likely to be curative, is an important way to reduce overall cancer mortality. The introduction of screening programs to detect cancers of the breast and colon has resulted in fewer cancers being detected after metastasis and corresponding reductions in cancer specific mortality. However, to date, organized screening programs focus on single cancers, utilizing technologies to visualize small cancers via mammography (breast) and low dose computed tomography (LDCT) (lung). While much scientific effort has been directed towards finding cancer biomarkers which could be tested for in body fluids, those that have been found do not have widespread screening utility, with the exception of certain fecal biomarkers which can signal the presence of colorectal cancers[1].

A promising new biomarker technology for early cancer detection involves sequencing of the cell-free nucleic acids (cfNA) shed by dying cells and circulating in the blood. This approach presumes that cancers will shed nucleic acids that can be distinguished from those shed from healthy cells, and further that features of the signals will be localizable to the site of origin.

Preliminary work suggests that this technology can detect a variety of clinically diverse cancers and their site of origin with high specificity and could thereby be used to test for multiple cancers simultaneously[2]. If routine testing could detect multiple cancers earlier, including cancers with a high mortality rate for which there are currently no screening strategies for early detection, it could potentially have a greater impact on overall mortality due to cancer than current approaches, which address one cancer at a time.

There has been significant international interest in developing a blood test for early cancer detection. A blood test would be minimally invasive and would require minimal physical infrastructure to deliver, which could substantially improve access to cancer screening benefits. Screening persons for multiple cancers simultaneously would also be expected to detect cancers before they have metastasized, when treatment is likely to be more successful, thereby reducing overall cancer morbidity and mortality.

GRAIL, Inc. (GRAIL) has developed a multi-cancer early detection test called Galleri™ that uses cell-free deoxyribonucleic acid (cfDNA) targeted methylation signals to distinguish cancer from non-cancer and identify the cancer signal of origin. Development and validation of this test is described in Section 3.2 below. Understanding the test's clinical utility, its relation to established diagnostic procedures, and other elements of its practical deployment as a screening tool are critical to its successful implementation in diverse clinical settings.

3.1. Study Rationale

This study is being conducted to demonstrate the safety and effectiveness of the Multi-cancer Early Detect Test (MCED). Results will be submitted to the FDA, in combination with results from additional clinical validation studies, in a Pre-Market Application (PMA). The study will continue to collect data that will be used in the post-approval setting.

Successful introduction of a novel multi-cancer early detection test warrants an assessment and consideration of both the potential benefit and risks of the test. Benefits include the opportunity to improve cancer outcomes by identifying cancer at earlier stages or even prior to symptomatic presentation when interventions are often more effective. Risks include those associated with diagnostic

evaluations and invasive procedures occurring in false positive cases, anxiety associated with test results, and risks associated with returning cancer signal detected without a correctly predicted cancer signal origin (CSO) (eg, risks associated with the diagnostic evaluations). The study will also follow test-negative participants to assess the false negative rate and assess impact on participants' adherence to standard of care cancer screening tests. Additionally, multi-cancer early detection test results may impact participants' understanding and perception of their risk of cancer. This protocol aims to assess these as part of its ultimate goal, to inform the broader implementation and dissemination of a blood-based multi-cancer screening paradigm.

3.2. Background of the Study Test

Study Test

The study test to be used in this protocol, GRAIL MCED test, is a qualitative, next-generation sequencing (NGS)-based screening test for the detection of a DNA methylation signal using cell-free DNA isolated from adult human peripheral whole blood. GRAIL's Galleri test is a screening test and cannot be used to confirm a cancer diagnosis. The test result of "cancer signal detected" (also referred to as a "positive" result throughout this protocol) may indicate the presence of cancer, and should be followed by diagnostic tests. These diagnostic tests should be ordered by qualified health care professionals in accordance with professional guidelines that take into account the Cancer Signal Origin result from the Galleri test.

When a cancer signal is detected, Cancer Signal Origin(s) is reported. Depending on the relative strength of the prediction of the cancer signal origin, one or two signal origins are reported. The Galleri test has 21 possible Cancer Signal Origins: Anus; Bladder, Urothelial Tract; Bone and Soft Tissue; Breast; Cervix; Colon, Rectum; Head and Neck; Kidney; Liver/Bile Duct; Lung; Lymphoid Lineage; Melanocytic Lineage; Myeloid Lineage; Neuroendocrine; Ovary; Pancreas, Gallbladder; Plasma Cell Lineage; Prostate; Stomach, Esophagus; Thyroid Gland; Uterus.

The test does not test for or report clinically-relevant single genetic mutations.

Completed Studies

GRAIL developed technologies to assay cell-free nucleic acid (cfNA) signals using broad, deep sequencing to detect multiple types of genomic and epigenomic alterations including methylation status, single nucleotide variants, insertions and deletions, copy number alterations, and structural rearrangements such as gene fusions. Multiple platforms, including analyses of whole genome and targeted methylation patterns, circulating RNA patterns and compartment specific factors (e.g. white blood cells and germline variants), have been tested to optimize sensitivity and specificity for an early detection blood test.

A preliminary evaluation of the GRAIL approach to blood markers was presented at the American Association for Cancer Research (AACR), American Society of Clinical Oncology (ASCO) and European Society for Medical Oncology (ESMO) annual meetings in 2018-2020[3-10]. The Circulating Cancer Genome Atlas (CCGA) study (Clinicaltrials.gov identifier: NCT02889978) is a large, prospective, longitudinal, multicenter clinical study which enrolled 15,254 participants with a recent diagnosis of cancer (n=8,584, 56%) and without a recent diagnosis of cancer (n=6,670; 44%), from 141 clinical institutions and multiple networks throughout the United States, as well as one site in Canada, from August 2016 to February 2019.

The first CCGA classification analysis (CCGA1) consisted of 2,800 participants, including 1,650 participants with newly diagnosed cancer who had not yet received treatment and 1,150 non-cancer participants. CCGA1 was divided into a training set with 1,785 participants and an independent test set with 1,015 participants. Three prototype sequencing assays were performed on blood samples. The three prototype sequencing assays included targeted sequencing of paired cell-free DNA (cfDNA) and white blood cells to detect somatic (non-inherited) mutations such as single nucleotide variants and small insertions and/or deletions; whole-genome sequencing of paired cfDNA and white blood cells to detect somatic copy number changes; and whole-genome bisulfite sequencing of cfDNA to detect abnormal cfDNA methylation patterns. The prototype assays detected a highly specific, strong biological signal in cancer types that are typically not screened for and have low survival rates (five-year cancer-specific fatality rate of greater than 50 percent): lung, ovarian, pancreatic, liver, and esophageal cancers. For these cancers in the CCGA1 Training set at 98% specificity, the sensitivity for stages I-III cancers (N=117) was 54%, with the highest performing prototype assay, whole genome bisulfite sequencing. As expected, sensitivity increased to 90% for stage IV cancers (N=81). Through longitudinal follow-up of participants in the training and test set of CCGA1, it has since been confirmed that three of eight “non-cancer” participants who had an elevated signal have been diagnosed with cancer. This suggests the signal indicated the presence of undiagnosed cancer[11].

The second CCGA classification analysis (CCGA2) utilized a targeted methylation cfDNA assay that serves as the basis for the development of the classifier that distinguishes cancer from non-cancer and identifies the cancer signal origin. CCGA2 includes approximately 4,800 participants from CCGA, comprising approximately 2,800 cancer and 2,000 non-cancer participants, and an independent validation set of approximately 2200 non-cancer participants. These participants are split into Training (~70%) and Validation (~30%) sets. Approximately 2700 were excluded from the Training and Validation, due to ineligibility or un evaluability, or because they were reserved for future analysis. An analysis population of approximately 3050 (1,531 cancer and 1521 non-cancer) participants were included in the Training set, and approximately 1260 (654 cancer, 610 non-cancer) participants were included in the Validation set. The classifier achieved a specificity of 99.8% versus 99.3% between the training and validation sets, respectively. Among all cancers, the overall sensitivity for cancer detection was 55.2% across all stages in the training set, and 54.9% in the validation set. In a pre-specified list of clinically significant cancers (anorectal, breast [HR-negative], colorectal, esophageal, gastric, head and neck, hepatobiliary, lung, lymphoid neoplasm [chronic lymphocytic leukemia, hairy cell leukemia, lymphoma], multiple myeloma, ovarian, pancreatic), overall sensitivity (stage I-IV) of 77.9% for cancer detection was achieved in the training set versus 76.4% in the validation set. Additionally, the preliminary analysis showed that a tissue of origin could be returned in 96% of the cases, and amongst these cases, was correct in 93% of the cases, which was consistent across stages[2].

The third CCGA classification cohort (CCGA3) is a large (approximately 4,000 participants) Clinical Validation cohort that was used to assess the performance of the GRAIL test, which was developed based on CCGA2 and has been further refined for use as a screening tool, to lay the foundation for population-scale clinical implementation of the test. Data analysis will be complete prior to the initiation of this study.

Ongoing Studies

The Circulating Cancer Genome Atlas (CCGA) study is ongoing with additional analyses planned.

The STRIVE Study (NCT03085888) is a prospective, observational, multicenter, longitudinal study of women undergoing screening mammography that will be used to support the development and validation of the GRAIL test for detection of multiple types of invasive cancers. The study has fully enrolled 99,252 participants from 35 clinical sites across the United States. While the enrolment strategy utilized screening mammography to identify women seeking screening for breast cancer, the study was designed to capture the broad range of cancers that develop in this population allowing for analyses of the GRAIL test which is designed to detect multiple types of cancer.

The SUMMIT Study (NCT03934866) is a prospective, observational, multicenter, longitudinal study of men and women with significant smoking history that will be used to support the development and validation of the GRAIL test for detection of multiple types of invasive cancers. The study has enrolled approximately 13,000 participants in London and surrounding geographies. Participants have undergone a lung health check assessment and screening for lung cancer using low dose CT at baseline. The study is ongoing with additional screening timepoints at year 1 and year 2.

The PATHFINDER Study (NCT04241796) is a prospective, interventional, multicenter study of participants receiving an investigational multi-cancer early detection test, developed by GRAIL. The study has fully enrolled 6,662 participants from clinical sites in the United States. The study will assess the implementation of and participant experience with this novel multi-cancer screening paradigm. Objectives include assessing the number and type of diagnostic procedures required to achieve diagnostic resolution, clinical test performance of the multi-cancer early detection test and participant-reported outcomes to assess participants' perceptions about the multi-cancer early detection test. Participants will be followed for approximately 12 months from the time of enrolment.

Further additional clinical studies are planned.

3.3. Benefit/Risk Assessment

It is anticipated that some participants will receive a “cancer signal detected” test result and that the subsequent work-up will lead to a diagnosis of cancer, which could then be treated appropriately. This may result in an earlier diagnosis than if the participant were not involved in the study. Individual participants may not experience a direct benefit from participation. However, the study will also help GRAIL further evaluate the feasibility and acceptability of a multi-cancer early detection test.

Summary of Known Potential Risks to Study Participants

Adverse Events Associated with Peripheral Venipuncture:

There are potential risks to study participants associated with peripheral venipuncture. The safety of peripheral venipuncture has been well established. When blood is drawn from a vein, there will be temporary discomfort and a minimal risk for slight bleeding, bruising, lightheadedness, slight irritation at the blood draw site and, in rare instances, infection or fainting.

Follow-Up Procedure-Related Complications:

Potential risks for study participants include those from diagnostic testing (procedure or imaging, e.g. endoscopy, colonoscopy, sigmoidoscopy, laparoscopy, biopsy, CT with or without intravenous (IV) contrast, magnetic resonance imaging (MRI), ultrasound, mammography, positron emission tomography–computed tomography (PET-CT)) as part of the diagnostic evaluation follow-up for a "cancer signal detected" test result. It is expected that the potential risks of any diagnostic procedures determined appropriate by the participant's medical team, which will be administered as part of clinical care, will be explained to the participant during the clinical consent process (in addition to other information, such as potential benefits, risks, and alternatives). The informed consent form (ICF) for this clinical protocol will describe the potential risks from the clinical diagnostic procedures. Diagnostic procedure potential risks include, but are not limited to:

- allergic reactions and respiratory problems, and in rare instances death from general anesthesia or sedatives that may be used with endoscopic procedures
- perforation of the colon, esophagus, or stomach by the colonoscope, sigmoidoscope, or endoscope
- prolonged or severe bleeding from endoscopy, colonoscopy, sigmoidoscopy, or laparoscopy injury
- infection from endoscopy, colonoscopy, sigmoidoscopy, or laparoscopy
- visceral injury and bleeding, injury to the bowel, or injury to the bladder from laparoscopy
- exposure to ionizing radiation from imaging procedures
- acute allergic or severe contrast reactions including acute renal failure, and in rare instances death from procedures using imaging contrast agents
- temporary discomfort, bruising, lightheadedness, and in rare instances infection or fainting associated with peripheral venipuncture

Follow-up procedures may also result in incidental findings unrelated to the multi-cancer early detection test result. It is the responsibility of the study investigator(s) to determine how to address incidental findings related to diagnostic testing.

After the identification of a suspicious abnormality by any procedure(s) or imaging that is administered as a result of a "cancer signal detected" test result, additional imaging and/or biopsy and/or surgery performed to obtain tissue for tumor analysis will be part of the participant's clinical care and will be determined by the participant's personal healthcare provider or study investigator, not by this protocol.

Risks Related to the Test Result:

There is a potential risk of receiving a test result indicating a cancer signal is detected when no cancer is present (sometimes referred to as "false positive"). A participant with this result may be recommended for unnecessary follow-up diagnostic procedures, and as a result, may incur physical and psychological risks and adverse events that are associated with those procedures.

There is also a potential risk of receiving a test result indicating no cancer signal is detected when cancer is actually present (sometimes referred to as a "false negative"). In this case, a participant may incorrectly assume that s/he does not have cancer, and may experience a delay in follow-up diagnostic procedures and subsequent delay in a potential cancer diagnosis. A "cancer signal not detected" test result should not be interpreted as absence of cancer and should not supersede guideline-recommended screening results or other standard of care diagnostic or treatment algorithms. Failure to adhere to guideline-recommended screening or other standard of care modalities could also lead to a delay in a potential cancer diagnosis. The specificity and sensitivity performance characteristics in the CCGA study are described in Section 3.2

Risks Related to Overdiagnosis:

With any screening test there is a risk of overdiagnosis – diagnosing subclinical disease that would never have progressed to clinical disease. In cancer screening, significant overdiagnosis is limited to screening tests that are sensitive to the very earliest cancers. It is not anticipated that overdiagnosis will be a substantial concern for the GRAIL MCED test because the test has lower sensitivity for stage I cancer and it is unlikely that many stage II+ cancers are truly indolent. Overdiagnosis in cancer screening is a particular issue for prostate and breast cancers, both of which have very long progression times compared to most other cancers. It is of note that the GRAIL test has lower sensitivity for detection of these two cancers.

Questionnaire Related Risks:

The questionnaires used in this study may make participants feel uncomfortable. Participants are encouraged to speak to the study team regarding any concerns prompted by the questionnaires.

Tumor Tissue:

Biopsies and surgeries performed are part of participants' clinical care and are not considered a study-related procedure in this protocol. Archival tumor tissue for study purposes will only be accessed ex vivo from the site pathology service and does not pose any additional risk to the participant.

Additional Considerations:

It is not within the scope of this study to return results to participants related to genetic assessment for germline cancer risk. If such assessments are desired, such testing should be sought elsewhere through clinically available means and as determined by the participant's healthcare provider. Incidental findings, such as those related to chromosomal aneuploidies (e.g., XXY or mosaic trisomy 8), will not be reported.

4. Objectives & Endpoints

Primary Analysis Objectives and Endpoints

There are two primary study objectives:

1) Evaluate the safety of the MCED test in terms of diagnostic testing triggered by the MCED test result.

2) Evaluate performance of the MCED Test in individuals eligible for cancer screening.

Safety endpoints include the number and type of invasive procedures (as described in Appendix II) performed in those with no cancer diagnosis at the time of diagnostic resolution (referred to as false positive (FP) henceforth). Safety measures for these endpoints are described in Section 10.4.2.

Test performance endpoint is diagnosis of invasive cancer, which will be incorporated into the measures associated with screening test performance for cancer detection and CSO prediction as described in Section 10.4.2. The endpoint will be assessed at multiple time points during the study including at diagnostic resolution following a signal detected result (and after confirmatory PET-CT in those cases undergoing PET-CT as described in section 5.1) and at 1 year and 2 years.

Cancer is defined as the following invasive solid and hematologic malignancies:

- Invasive solid cancer (stage I-IV), excluding non-metastatic basal cell carcinoma and squamous cell carcinoma of the skin
- Invasive brain cancer
- Hematologic malignancies, including lymphoma (stage I-IV), lymphoid leukemia with no expected staging by the American Joint Committee on Cancer (AJCC), plasma cell neoplasm (stage I-III), myeloid neoplasms (including myelodysplastic and myeloproliferative neoplasms with behavior code 3 (based on ICD-O-3).

Cancer diagnosis is based on pathologic confirmation of an invasive or hematologic malignancy, or imaging confirmation in the absence of pathology, or other clinical confirmation of disease per treating clinician (e.g. biochemical evidence of recurrence of prior cancer).

Secondary Analysis Objectives and Endpoints

There are six secondary study objectives:

1) Assess participant-reported anxiety resulting from use of the MCED test

   Endpoints:

   - Anxiety assessed by State Trait Anxiety Inventory (STAI)[12]+ MCED STAI questionnaires pre-test (baseline), post-test, at diagnostic resolution (if applicable) and at 1 year (+/-30D).

2) Evaluate intention and utilization of guideline recommended cancer screening procedures after use of the MCED test

   Endpoints:

   - Intention to follow standard of care cancer screening tests assessed pre-test, post-test and at 1 year (+/-30D).
   - Utilization of standard of care cancer screening tests prior to study enrollment, during the first year and the second year (+/-30D) after MCED test.

3) Describe the ability of confirmatory PET-CT to detect cancer in participants where diagnostic resolution of cancer diagnosis is not achieved through the targeted diagnostic testing triggered by MCED test results.

   Endpoint:

   - Diagnosis of invasive cancer after confirmatory PET-CT

4) Characterize the number and type of diagnostic evaluations by predicted cancer signal origin and outcome of diagnostic resolution.

   Endpoints:

   - Number and type of imaging procedures
   - Number and type of invasive procedures
   - Number and type of laboratory tests
   - Time to diagnostic resolution

5) Evaluate the radiation exposure during diagnostic evaluation.

   Endpoint:

   - Per participant radiation exposure during diagnostic evaluation

6) Evaluate concordance of initial GRAIL test result and CSO prediction, with results from the research blood draw.

   Endpoints:

   - MCED test results for cancer detection and CSO prediction from the initial and research blood draws in the subset of participants with research blood draw.

7) Assess participants reported outcomes and perceptions about the MCED test.

   Endpoints:

   - Health-related quality of life following the MCED test assessed by SF-12v2
   - Adapted MICRA
   - Satisfaction with the MCED test
   - Attitudes towards subsequent MCED screening

Analysis methods and measures for the secondary study objectives are described in Section 10.4.3.

Exploratory Analysis Objectives and Endpoints

There are four exploratory study objectives:

1) Evaluate performance of the MCED test in year 3.

   Endpoint:

   - Diagnosis of invasive cancer in year 3.

2) Evaluate changes in blood based biomarkers among the subset of participants with a research blood draw.

   Endpoints:

   - Blood biomarker signals from the initial and research blood draws in the subset of participants with a research blood draw.

3) Evaluate different CSO reporting classes based on clinically relevant subgroups.

   Endpoints:

   - MCED test results for CSO prediction and blood biomarker signals from the initial and research blood draws evaluated in the context of diagnostic testing triggered by the test results.

4) Evaluate DNA based and other biomarkers in tissue compared to blood from cancer cases.

   Endpoints:

   - DNA based and other biomarkers in tissue and blood samples in participants with cancer

5. Study Design

5.1. Overall Design

This is a prospective, multi-center interventional study of the GRAIL MCED Test with return of results in healthcare systems in the United States. The purpose is to evaluate the safety and efficacy of the GRAIL MCED Test in an eligible screening population.

The study will enroll approximately 10,000 participants as defined by eligibility criteria over an anticipated enrollment period of approximately 10 months at up to 30 clinical institutions within North America. Each enrolling institution will employ its own recruitment strategies to identify potential participants, which may include identification of participants through electronic health records, scripted phone calls, emails, recruitment campaigns, and other outreach strategies. Recruitment strategies at each enrolling institution may require Institutional Review Board (IRB) or Research Ethics Boards (REB) approval, as applicable.

Following informed consent, the participant will undergo the GRAIL MCED Test. Clinical information, including demographic and medical data relevant to cancer risk, will be collected from all participants via medical record, participant questionnaires, and the GRAIL Multi-cancer Test Requisition Form (TRF). The test will be ordered by a study investigator at an enrolling site.

Following blood collection, the study test will be performed at the GRAIL clinical laboratory, which has a Certificate of Accreditation under the Clinical Laboratory Improvement Amendments (CLIA) to perform high complexity testing. The Test Result Report will be returned to the study investigator and site study team via the test result portal and any ensuing diagnostic work-up will be captured (see Section 9.4).

GRAIL MCED Test Negative

Participants will be notified of "cancer signal not detected" test results via phone call, virtual visit, or an in-person clinic visit with the study investigator or designated member of the study team (e.g. research nurse), and the study team must remind participants not to interpret "cancer signal not detected" test results as the absence of cancer and to continue to adhere to guideline-recommended cancer screening.

GRAIL MCED Test Positive

Participants will be notified of "cancer signal detected" test results via phone call, virtual visit, or by in-person clinic visit with the study investigator or designated member of the study team (e.g. research nurse). As appropriate, during the communication of results, participants will be invited to follow-up with their personal healthcare provider (primary healthcare provider or specialist) or to follow-up with a study investigator. If the participant elects to follow-up with his/her personal healthcare provider, the study team will notify the personal healthcare provider of the "cancer signal detected" test results and confirm the healthcare provider's willingness to work-up the result in consultation with the study team. If the participant does not elect to follow-up with his/her personal healthcare provider or the personal healthcare provider declines to work-up the participant, a study investigator will be available to work-up the result. The personal healthcare provider may consult, as needed, with relevant specialists or review boards at their institution, to discuss the diagnostic work-up of a "cancer signal detected" test result.

It is intended that participants will receive targeted diagnostic tests, procedures or evaluations based on the test returned cancer signal origin(s) (CSO). For cancer signal detected test results, up to two cancer signal origins may be reported. It is intended for the healthcare provider to first evaluate the participant for the first cancer signal origin. If no cancer is identified, the healthcare provider should evaluate the participant for the second cancer signal origin reported. The healthcare providers are strongly encouraged to attempt to rule out cancer in the organ or anatomical location described by the predicted cancer signal origin, where applicable, using the diagnostic evaluations corresponding to the cancer signal origins listed in Table 4. In some cases, based on the predicted cancer signal origins, it may be appropriate to utilize work-ups that evaluate both the first and second predicted cancer signal origins simultaneously. If cancer is not identified by these cancer signal origins directed evaluations, the healthcare provider should perform a PET-CT (see Figure 2). If a participant is unable to undergo the prescribed procedure or the institution physician determines an alternative or additional work-up is necessary, the institution physician will document the reason for deviating from the prescribed procedure(s) and list the alternative and additional procedure(s) performed.

Figure 2. Diagnostic Evaluations- Scenario of Positive Result Qualifying for Two Cancer Signal Origins

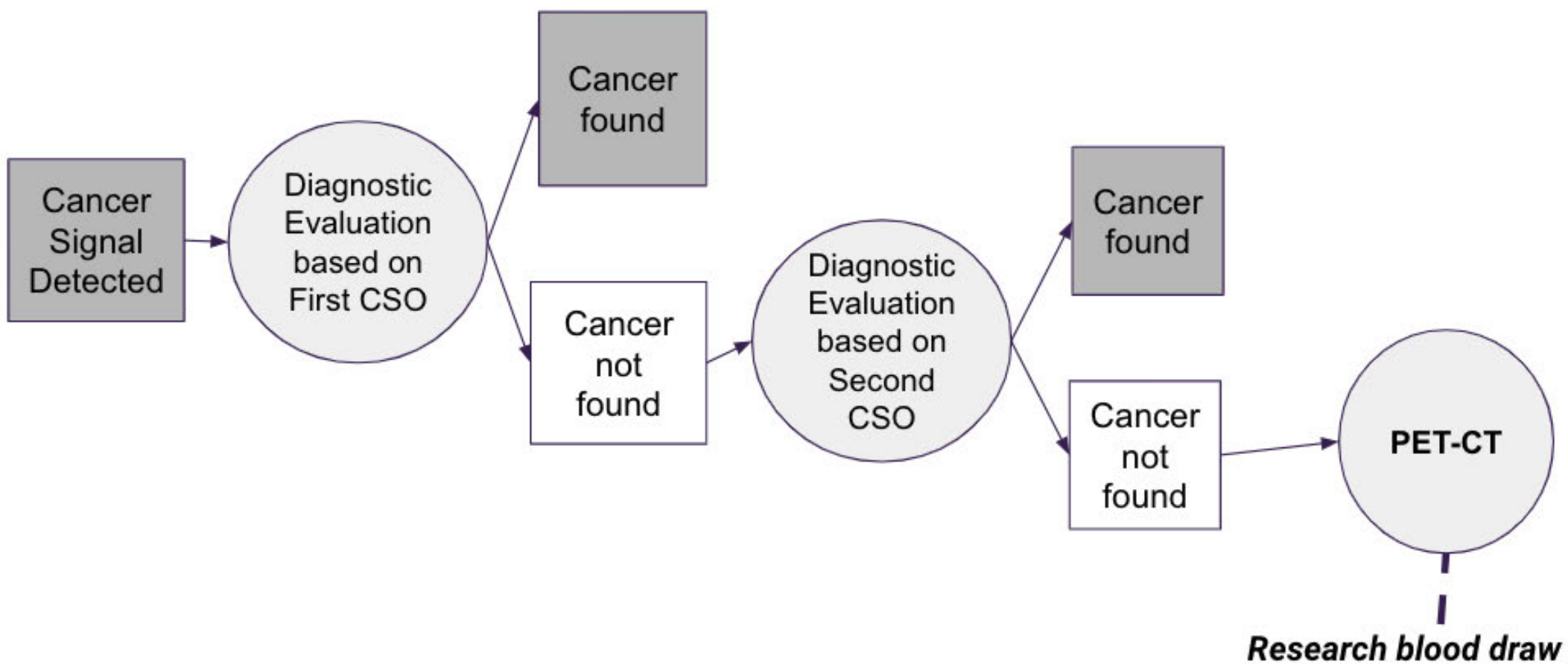

Table 4. Cancer Signal Origin Directed Diagnostic Evaluations

Cancer Signal Origin	Subcategories	Diagnostic Evaluation
Anus	Anus	anoscopy or sigmoidoscopy
Bladder, Urothelial Tract	Bladder, Renal Pelvis, Ureter, Urethra	flexible cystoscopy and CT urogram or retrograde pyelogram with contrast
Bone and Soft Tissue	Skeletal Muscle and Other Connective Tissue, Vascular Tissue, Bone and Cartilage	MRI with contrast or CT of chest, abdomen, pelvis or PET-CT
Breast	Breast	diagnostic mammography (MRI if participant has recently had screening mammogram)
Cervix	Cervix	cytology, colposcopy
Colon, Rectum	Colon, Rectum, Appendix	colonoscopy
Head and Neck	Oropharynx, Hypopharynx, Nasopharynx, Larynx, Lip and Oral Cavity (including Oral Tongue), Nasal Cavity, Paranasal Sinuses, Major Salivary Glands	flexible nasal endoscopy and Head and Neck CT or MRI
Kidney	Kidney	triple phase renal CT

Liver, Bile duct	Liver, Intrahepatic Bile Duct	triple phase abdominal CT, AFP
Lung	Lung, Bronchus	chest CT
Lymphoid Lineage	Lymphoid Lineage	PET-CT
Melanocytic Lineage	Melanocytic Lineage	full body skin exam
Myeloid Lineage	Myeloid Lineage	bone marrow biopsy after blood work
Neuroendocrine Cells of Lung or other Organs	Neuroendocrine Cells of Lung or Other Organs	CT of chest with or without contrast, CT of abdomen, pelvis, head & neck
Ovary	Ovary, Fallopian Tube, Primary Peritoneum	transvaginal U/S and CA-125
Pancreas, Gallbladder	Pancreas, Extrahepatic Bile Duct, Gallbladder	pancreas CT protocol or MRCP
Plasma Cell Lineage	Plasma Cell Lineage	bone marrow biopsy after blood work
Prostate	Prostate	PSA and multi-parametric MRI of the prostate
Stomach, Esophagus (upper GI)	Stomach, Esophagus	upper endoscopy
Thyroid Gland	Thyroid Gland	ultrasound of thyroid
Uterus	Uterus	transvaginal U/S

Additional evaluations for all Cancer Signal Origins: Complete Blood Count (CBC) with Differential Test and Comprehensive Metabolic Panel

- For participants where diagnostic resolution of cancer diagnosis is achieved through the targeted work-up based on the CSO, pathologic, imaging and clinical staging information will be captured.
- For participants where diagnostic resolution of cancer diagnosis is not achieved through the targeted diagnostic procedures, and where PET-CT was not already performed as part of the work-up, a confirmatory PET-CT will be performed to assess cancer status. For participants where diagnostic resolution of cancer is achieved through PET-CT, pathologic, imaging and clinical staging information will be captured. Participants in this group will also undergo a research blood draw, which may be completed on the same day and before PET-CT or within 14 days after the PET-CT. Results of this research blood draw will not be provided back to the physician or participant.
- All efforts should be made to achieve diagnostic resolution within 6 months after receipt of MCED test results.

- The result of any performed diagnostic procedure(s) will be recorded until the date of "diagnostic resolution." "Diagnostic resolution" is defined as the date when the study team (and/or the personal healthcare provider) determines to end diagnostic evaluation triggered by a "cancer signal detected" test result.

- Participant questionnaires will be deployed prior to the Day 1 blood draw, following receipt of study test results, following diagnostic resolution for "cancer signal detected" test results, and at 1 year (+/-30D) to assess parameters related to participant perceptions about the multi-cancer early detection test, including anxiety, health-related quality of life, and potential impact of test results on attitude towards adherence to guideline-recommended screening.

For participants diagnosed with invasive cancers at the time of diagnostic resolution and during a 3 year follow-up period, archived tumor tissues obtained from standard of care biopsy procedure or surgical resection will be collected (see Section 9.7).

5.2. Estimated Study Duration

The expected study duration is approximately 4 years.

5.3. Study Duration for Study Participants

The expected duration for each study participant will be 3 years from the Day 1 blood draw.

5.4. Study Design Schema

Please refer to Section 2.2., Schema, above.

5.5. Scientific Rationale for Study Design

This prospective, longitudinal, multicenter clinical study design is required to provide data to comprehensively evaluate the safety of the GRAIL MCED test. Additionally and in combination with other studies, this design will provide data to evaluate the efficacy of the GRAIL MCED test.

5.6. Study Enrollment Definition

Study enrollment commences when the first study participant meets all inclusion and no exclusion criteria and provides informed consent.

5.7. End-of-Study Definition

All participants will be actively followed by the enrolling institution for 3 years following their blood draw date.

The end of the study for all participants is defined as the completion of the last study assessments for the last participant, which may occur 3 years after the last participant's Blood Draw date or sooner, if the Sponsor ends the study early (See Section 9.8).

6. Study Population

The study will enroll at least 10,000 participants with the objective to capture at least 100 "cancer signal detected" cases. Enrollment will continue until at least 100 "cancer signal detected" results have been achieved. The study should ensure a diverse participant population is enrolled, generally representative of the US population with respect to race, ethnicity and sex. Targets for distribution by race and ethnicity are listed in Table 5 (2019 U.S. Census). The study will target the age and sex distribution listed in Table 6 which is enriched for individuals in the age categories 60-69 and 70-79 over the U.S. population. Enrichment for these age categories will serve as the primary method to enrich the cohort for cancer events and enrollment will be monitored to ensure these targets are achieved. Individual age and sex strata may be closed to new enrollments during the course of the study to achieve the target distribution.

Table 5. Targets for Distribution by Race and Ethnicity

Race/ Ethnicity	Target for Enrolled Study Population
White (non-Hispanic)	60%
Hispanic or Latino[a]	19%
African American or Black[a]	13%
Asian[b]	6%
Multiple Races	3%

(a) Hispanics may be of any race, so also are included in applicable race categories
(b) Includes persons reporting only one race

Table 6. Targets for Age and Sex Distribution

	Age 50-59 years	Age 60-69 years	Age 70-79 years	Age 80+ years
Female	7.5%	25%	15%	2.5%
Male	7.5%	25%	15%	2.5%

Prospective approval of clinical study protocol deviations (PDs) to recruitment and enrollment criteria, also known as clinical study protocol waivers (protocol waivers) or clinical study protocol exemptions (protocol exemptions), is not permitted.

6.1. Inclusion Criteria

Participants are eligible to be included in the study only if all of the following criteria apply.

1. Participants must be at least 50 years of age, inclusive, at the time of signing the Informed Consent Form (ICF).

2. Informed Consent

    - Capable of giving signed and legally effective informed consent as described in Section 5.4, Study Design Schema, which includes compliance with the requirements and restrictions listed in the Informed Consent Form (ICF) and in this protocol. Consent provided by a legally authorized representative is not permitted in this protocol.

6.2. Exclusion Criteria

1. Undergoing or referred for diagnostic evaluation due to clinical suspicion for cancer (e.g., referred to a medical or surgical oncologist, or scheduled for biopsy on the basis of a suspicious imaging abnormality).

2. Personal history of invasive or hematologic malignancy, diagnosed within the 3 years prior to expected enrollment date, or diagnosed greater than 3 years prior to expected enrollment date and never treated.

3. Prior/Concurrent Concomitant Therapy (Medications/Treatments):

    - Definitive treatment for invasive or hematologic malignancy within the 3 years prior to expected enrollment date. Adjuvant hormone therapy for cancer (e.g. for breast or prostate cancer) is not an exclusion criterion.

4. Individuals who will not be able to comply with the protocol procedures.

5. Individuals who are not currently registered patients at a participating center.

6. Previous or current participation in another GRAIL-sponsored study. “Participation” is defined as having signed consent and provided a blood sample.

7. Previous or current employees or contractors of GRAIL.

6.3. Participant Recruitment

Each enrolling institution will employ recruitment strategies to identify potential participants, which may include identification of participants through electronic health records, scripted phone calls, emails, recruitment campaigns, and other outreach strategies.

6.4. Informed Consent Process

Only participants who meet all inclusion and exclusion criteria will sign the informed consent. Once a participant is confirmed to meet all eligibility criteria, they will then sign informed consent. Participants who do not meet all eligibility criteria should not sign the informed consent.

- The study investigator or designated member of the study team will explain the nature of the study to the prospective participant and answer all questions regarding the study.

- Prospective participants must be informed that their participation is voluntary. Participants will be required to sign a statement of informed consent that meets the requirements of Title 21, Code of Federal Regulations, Part 50 - Protection of Human Subjects (21 CFR 50), local regulations, ICH guidelines, Health Insurance Portability and Accountability Act (HIPAA) requirements (if the HIPAA authorization is combined with the consent form in one document), and any stipulations or conditions imposed by the Institutional Review Board (IRB)/Independent Ethics Committee (IEC)/Research Ethics Board (REB) or study center.
- A legally authorized representative is not allowed for the purposes of this study. Participants must understand the protocol requirements and be able to give legally effective informed consent.
- The medical record must include a statement that written informed consent was obtained before the participant was enrolled in the study, and the date the written consent was obtained. The authorized person obtaining the informed consent must also sign the ICF.
- Participants must be re-consented to the most current version of the ICF(s) during their participation in the study, if required by the IRB/REB.
- Participants will be consented to allow for the option of return of results from a future version of the test.
- A copy of the ICF(s) must be provided to the participant.

Informed consent should occur within 30 days prior to the Day 1 blood draw and must be obtained prior to any study procedures being performed. Participants who are unable to perform the Day 1 blood draw within 30 days of consent may delay the Day 1 blood draw up to 60 days from informed consent. However, in these instances, all eligibility criteria must be re-confirmed prior to drawing blood. If blood is not drawn within 60 days following consent, the participant will be withdrawn from the study.

The ICF may be offered in several IRB/IEC/REB-approved formats so that participants can review or complete it in advance. These formats include remote e-consent (desktop/laptop, mobile tablet, or smartphone), in-clinic consent (tablet or paper), mailed consent (paper), and PDF (for pre-review only). Availability of each format to a given participant will depend on the clinical site's policies.

The ICF will contain a separate section that addresses the use of remaining samples for exploratory research. The study investigator or designated member of the study team will explain to each participant the objectives of the exploratory research. Participants will be told that they are free to refuse to participate and may withdraw their consent at any time and for any reason during the storage period.

No study procedures will be performed prior to obtaining informed consent. Informed Consent will be obtained per federal regulations and applicable ICH /Good Clinical Practice (GCP) guidelines.

Copies of all signed informed consent forms will be stored at the study site. Signed consent forms will be available for verification by GRAIL or its designee at any time.

7. Measures to Minimize Bias

In this prospective longitudinal study, information related to demographics, clinical characteristics, diagnostic procedure types, counts, and dates will be captured from the electronic medical records (EMR), which will minimize most biases (e.g., recall bias, observer bias, missing data bias).

An important source of bias in prospective studies is selection bias due to differences in response rates among participant subgroups, or with respect to study endpoints. Selection bias or poor representativeness could result in study results not being sufficiently generalizable to larger screening populations. To assess bias in enrollment, the Sponsor will monitor and continuously compare distributions of key demographic and clinical characteristics of the enrolled population to the target population. In particular, if there is disproportionately high enrollment of a particular stratum (based on age and sex), further enrollment of individuals in this stratum may be limited. This will be determined by the Sponsor.

7.1. Blinding Methods

This study is not blinded.

7.2. Participant Follow-Up

7.2.1. Active Clinical Follow-Up

Participants with a "cancer signal detected" test result will require additional evaluation for cancer and any follow-up per the discretion of the study investigator or personal healthcare provider (see Sections 9.5).

All participants will be followed for 3 years after the date of enrollment, and these study procedures will be conducted (see SoA):

- 1 year (+/-30D) questionnaire
- 1 year, 2 year, 3 year (+/-30D) cancer assessment
  - Follow-up clinical information for cancer status on all participants will be performed at 1 year, 2 year, 3 year (+/-30D) after enrollment from a search of medical records by the study team, which may be supplemented by direct contact to participants.
  - Collected clinical data will include but is not limited to: cancer diagnosis, type, date of cancer diagnosis, and method of detection.
  - Remaining tumor tissue obtained from standard of care biopsy procedure or surgical resection will be collected (see Section 9.7).
- 1 year, 2 year, 3 year (+/-30D) assessment of guideline-recommended cancer screening utilization

7.3. Participant Retention Through Follow-Up

The GRAIL-maintained study website and regular email communications will be the primary venue for providing updates and maintaining engagement of the participants throughout the study follow-up period. However, in the informed consent form, participants will be able to indicate their preferred method of communication, including but not limited to email, telephone, text/short message service (SMS), etc. The study sites will be responsible for sending communications to the participants.

The study participant will be considered active in the study per study investigator and/or Sponsor decision.

Upon successful contact, the study team member should confirm the eligibility of the study participant prior to any study assessments or procedures.

During the study period from return of results until EOS, the study site should make every attempt to contact the participant based on the participant's preferred method of communication. IRB/IEC/REB approval of such communications to the participant for follow-up will be obtained as required by study sites. The study participant may be contacted in order to meet the study assessments determined by this protocol and site specific ICF.

See Section 8.2 - Lost to Follow-Up for lost to follow-up determination.

8. Participant Withdrawal

8.1. Participant Withdrawal from the Study

- A participant may withdraw from the study at any time at their own request or may be withdrawn at any time at the discretion of the PI, GRAIL, overseeing IRB/IEC/REB, or regulatory authority, for safety, behavioral, compliance, or administrative reasons.
- If the participant withdraws consent for disclosure of future information, GRAIL may retain and continue to use any data collected before such a withdrawal of consent.
- If a participant withdraws from the study, they may request destruction of any samples taken and not tested, and the PI must document this in the study site records.
- Cancer status assessment will be collected at the time of study participant withdrawal.

A participant may be withdrawn from the study for the following reasons:

- **Failure to meet inclusion/exclusion criteria**:
  - If a participant has consented in the study but retrospectively found to not meet all of the inclusion/exclusion criteria, the participant will be withdrawn from the study.
- **Failure to perform Day 1 blood draw within 60 days following consent**
- **Participant withdrawal of consent:**
  - Participation in this study is voluntary. Participants may decide not to participate or may leave the study at any time. This decision will not result in any penalty or loss of benefits to which

participants are entitled. Information and biospecimens that have already been collected may still be used, but no new information will be collected.

    - If a participant's biospecimen has not been processed at the time the participant withdraws from the study, the biospecimen may be destroyed. If the biospecimen has already been tested, the data generated from the testing will not be destroyed.

- **Other withdrawal circumstances:**
    - If the reason for participant withdrawal does not fall under any of the reasons outlined above, then the withdrawal reason and the withdrawal date must be documented. Discussion with the Sponsor is encouraged.

8.2. Lost to Follow-Up

A participant will be considered lost to follow-up if he or she is repeatedly unable to be contacted by the study site at the end of the study.

The following actions must be taken if a participant fails to complete a required study procedure or assessment:

- The study site must attempt to contact the participant and inform the participant of the importance of protocol compliance and ascertain whether or not the participant wishes to and/or should continue in the study.
- Before a participant is deemed lost to follow-up, the study investigator or designated member of the study team must make three (3) contact attempts to regain contact with the participant (e.g. telephone calls or emails sent to participant). These contact attempts should be documented in the participant's study record. Should the participant continue to be unreachable at the 3 year's time point, he/she will be considered withdrawn from the study, due to being lost to follow-up. The study investigator or designated member of the study team will then document the date of withdrawal in EDC, and no further data will be collected.

9. Study Assessments & Procedures

- Study procedures and their timing are summarized in the SoA (refer to Section 2.3., Schedule of Activities [SoA]). Protocol waivers or exemptions are not allowed.
- Adherence to the study design requirements, including those specified in the SoA, is essential and required for study conduct.
- Tests, evaluations and procedures conducted as part of the participant's routine clinical management (e.g. scheduled mammogram) and obtained before signing of the ICF may be utilized for baseline purposes provided they met the protocol-specified criteria.

Study staff at each site will be trained on all study procedures prior to the activation of each site. Clinical data collection for the study is performed by site clinical research staff and by participants via electronic methods. Subsequent study assessments beyond the blood draw visit will be based on test results.

9.1. Enrollment

Enrollment into this study requires confirmation of eligibility and informed consent.

Individuals may only enroll into this study once.

The Sponsor will monitor and regularly evaluate distributions of key demographic and clinical characteristics of both the enrolled and target cohorts. For example, if there is disproportionately high enrollment of a particular risk group, further enrollment of individuals in this group may be limited. This will be determined solely by the Sponsor.

9.2. Participant Questionnaire

Questionnaires will be delivered to participants through mail or electronic mail. Electronic participant-reported outcomes (ePRO) can be accessed on tablets, computers, or handheld devices. See Table 3 for additional details regarding participant questionnaires.

9.3. Blood Draw

There are three potential time points of blood draws for this study:

1. Blood Draw
    a. First blood draw required for enrollment into the study.
    b. Results will be returned to the study investigator and communicated to the participant.
2. Repeat Blood Draw
    a. Occurs in the event of technical issues or processing failures at the request of GRAIL.
3. Research Blood Draw (see Section 9.5)
    a. For "cancer signal detected" cases only where no cancer diagnosis is obtained; will be used for future research.
    b. Results will not be returned to the study investigator or participant.

At each blood collection time point (described above), peripheral blood will be obtained by routine venipuncture. Blood will be collected in four blood collection tubes and each blood collection tube should be filled completely (approximately 10 mL of blood in each tube). Two tubes of blood will be used to run the investigational test. Participants will be asked to have repeat blood draw (four tubes) should technical issues with the samples preclude sample processing or reporting. Any remaining blood samples will be

stored without readily identifiable information and may be used for process improvement activities, proficiency testing tasks, or future research activities relevant to the investigational test.

Study blood collection must be completed in a single session on one day, and blood tubes should be collected from a single venipuncture session following the methods described in the study-specific lab manual. Blood collection and shipping should be performed according to the laboratory manual. Blood collection tubes will be labeled at the time of blood draw and include personal health information with at least two identifiers (e.g. name, date of birth). Personal health information will also be collected on the TRF that is used for the blood collection process.

The expected amount of blood collected from participants is approximately 40 mL. The maximum amount of blood collected from participants is approximately 120 mL (40 mL at first blood draw, 40 mL at repeat blood draw if processing issues, 40 mL blood draw for research for "Cancer Signal Detected" participants if cancer diagnostic resolution is not achieved).

Blood and plasma samples remaining after the testing is completed will be stored and accessed by GRAIL for further research relevant to the blood test, for laboratory process improvements, and for proficiency testing, unless otherwise required by applicable site-specific ICF or per applicable requirements. GRAIL will store the blood samples in a secure storage space with adequate measures to protect confidentiality. Blood samples obtained from participants who were retrospectively determined to have not met the eligibility criteria will be destroyed after the clinical data collection period for enrolled participants has concluded.

The Streck blood collection tubes used in this study are authorized for the collection, stabilization, and transport of venous whole blood samples for use in conjunction with cell-free DNA next-generation sequencing liquid biopsy assays. In addition, GRAIL has conducted extensive analytical and clinical validation on specimens collected in Streck blood collection tubes, including specimen stability studies, to ensure compatibility of Streck blood collection tubes with the GRAIL product. The blood collection tubes have not been cleared or approved for use with our assay by the FDA.

9.4. Return of Results

The Test Result Report (TRR) is a report with the result of the study test, which will specify "cancer signal detected" or "cancer signal not detected" test results. In some cases where a final result cannot be determined, a Cancellation Report will be issued. The TRR will be returned to the study investigator via paper or electronic methods (test results portal) typically within 15 calendar days from the date of blood collection, barring holidays and unforeseen circumstances affecting sample shipment and lab processing. Only the study investigator or designated member of the study team will have access to the test result portal, which is encrypted and requires a unique access code for each designated user. The study investigator or designated member of the study team will review all study test results prior to communicating results to the participant.

The study investigator or designated member of the study team (e.g., research nurse) will notify participants of "cancer signal not detected" test results via phone call, virtual visit, or by in-person clinic visit with the study investigator or designated member of the study team. The participant will be

reminded not to interpret “cancer signal not detected” test results as the absence of cancer and to continue to adhere to guideline-recommended cancer screening.

Participants will be notified of “cancer signal detected” test results via phone call, virtual visit, or by in-person clinic visit with the study investigator or designated member of the study team (e.g. research nurse).

The study test result should be communicated to the participant as soon as possible, but not later than 30 days from the date of the blood draw. The date on which the test result is communicated to the participant must be documented in EDC.

9.5. Diagnostic Work-Up of a “Cancer Signal Detected” Test Result

It is intended that participants will receive targeted diagnostic procedures based on the test returned cancer signal origin(s). For cancer signal detected test results, up to two cancer signal origins may be reported. It is intended for the healthcare provider to first evaluate the participant for the first cancer signal origin. If no cancer is identified, the healthcare provider should evaluate the participant for the second cancer signal origin reported if one is reported. Healthcare providers are strongly encouraged to evaluate possible presence of cancer in the organ or anatomical location described by the predicted cancer signal origin, where applicable, using the diagnostic evaluations corresponding to the cancer signal origin labels listed in Table 4. If cancer is not identified by these cancer signal origins directed evaluations, the healthcare provider should perform a full body (skull vertex to mid-thighs) diagnostic positron emission tomography-computed tomography (PET-CT) scan with contrast. PET-CT should be performed according to practice parameters provided by the American College of Radiology[13]. If a participant is unable to undergo the prescribed procedure or if the institution physician determines an alternative or additional work-up is necessary, the institution physician will document the reason for deviating from the prescribed procedure(s) and list the alternative and additional procedure(s) performed.

Diagnostic resolution is defined as the date when the study team determines to end diagnostic evaluation. The following outcomes of diagnostic resolution should be documented in EDC:

- **Cancer Diagnosis:** pathologic confirmation of an invasive or hematologic malignancy, or imaging confirmation in the absence of pathology, or other clinical confirmation of disease per treating clinician (e.g. biochemical evidence of recurrence of prior cancer)

- **No Cancer Diagnosis:** no cancer diagnosis at the conclusion of diagnostic evaluation

For participants where diagnostic resolution of cancer diagnosis is achieved through the targeted diagnostic procedures based on the CSO, pathologic, imaging and clinical staging information will be captured.

For participants where diagnostic resolution of cancer diagnosis is not achieved through the targeted diagnostic procedures and PET-CT has not been used as part of the diagnostic evaluation, a confirmatory PET-CT will be performed to assess cancer status. For participants where diagnostic resolution of cancer is achieved through confirmatory PET-CT, pathologic, imaging and clinical staging information will be captured. Participants in this group will also undergo a research blood draw around the time of the PET-CT

(see Diagram 1). Results of this research blood draw will not be provided back to the physician or participant.

All efforts should be made to achieve diagnostic resolution within 6 months after receipt of MCED test results.

9.6. Clinical Data Collection

Clinical data will be collected from the blood collection tube, Test Requisition Form (TRF), Test Result Report (TRR), the participant's medical record, and participant questionnaires, and will either be entered into and stored in GRAIL's Laboratory Information Management System (LIMS) or into electronic case report forms (eCRFs) in the EDC system.

Clinical data to be captured during the course of the study will include:

1. Personal Health Information, including name (or other identifier) and/or date of birth (DOB), will be collected and stored by GRAIL during the multi-cancer early detection testing process.
    a. Personal health information will be collected and entered into GRAIL's LIMS and kept encrypted and access restricted to trained GRAIL laboratory personnel.
    b. The participant study identification number (participant ID) will be used to link test results to the data captured in EDC. The site coordinators may enter participant email addresses provided by participants into the EDC system, only for the purposes of the questionnaire. The email addresses will be stored in the EDC system in such a way that the Sponsor Study Team does not have view or edit access. The analysis datasets exported from EDC and clinical study reports will not contain participant names, dates of birth or email addresses. Data used for analysis will include limited identifiable healthcare information required for research purposes, such as location of clinical site, age at enrollment, dates of study assessments, diagnostic work-up details, dates of cancer diagnosis, and date of death as applicable.
2. Demographics, such as but not limited to body mass index (BMI), biological sex, race/ethnicity, and age.
3. Past medical history including personal and family cancer history, cancer risk factors and cancer screening history, solid organ transplant history, bone marrow transplant history, and current health status, including pregnancy status and recent use of specific medications (such as cytotoxic or demethylating agents).
4. Test results, reports, and treatments including but not limited to laboratory tests, imaging procedures, endoscopic procedures, surgical procedures, other cancer screening and diagnostic tests and cancer treatments.
5. Cancer assessment-reporting of any cancer diagnosis, type, date of cancer diagnosis and method of detection.

For individuals with a "cancer signal detected" test result, clinical data collection may also include:

1. Documentation of criteria used by the healthcare providers to determine the diagnostic work-up selection.

2. Results from diagnostic work-up following a multi-cancer early detection test result, such as imaging, endoscopy, laboratory tests (e.g. hematology, blood chemistry), and other types of diagnostic procedures.

3. Clinical diagnostic resolution information (if invasive cancer is found) during diagnostic work-up.

4. All pathology reports and supporting diagnostic reports for biopsies and surgical resections relevant to cancer evaluation and diagnosis (e.g. anatomic pathology reports, molecular reports, IHC reports, flow cytometry reports, hemoglobin electrophoresis reports), clinical and/or pathologic stage.

5. Clinical findings, including incidental findings identified during the course of work-up and evaluations (e.g. lung nodules found on imaging, hematologic conditions identified during testing, adenomas detected during endoscopy)

6. Results of any molecular testing performed on tumor tissue or other specimens (e.g. urine, blood, cerebrospinal fluid) as part of standard of care.

7. Signs and symptoms.

8. Current use of cytotoxic or demethylating agents.

9.7. Tissue Sample Collection

For participants diagnosed with invasive cancer (excluding basal and squamous cell carcinomas), tumor tissue should be provided when sufficient and appropriate material is available. Suitable specimens will have been clinically obtained through standard of care biopsy procedure or surgical resection. Specimens collected after local or systemic anti-cancer therapy (radiation or chemotherapy) should not be selected for the purposes of this study. Tumor tissue should be provided when sufficient and appropriate material is available per requirements described below.

• 10x10 micron FFPE "thick" sections on unstained glass microscopic slides obtained from a representative, tumor containing pathology tissue specimen, along with bracketing 5 micron sections (12 slides total) to enable preparation of starting and ending hematoxylin and eosin (H&E) slides to guide macrodissection

• For small or limited tissue amounts, 10 x 4-5 micron "thin" sections along with bracketing 5 micron sections (12 sections total) are acceptable

Tumor tissue will be shipped to GRAIL or its designee per the approved Laboratory Manual.

9.8. Safety Assessments

9.8.1. General

Study participants may experience adverse events (AEs) associated with the study procedures or the investigational test. AEs associated with the investigational test are generally considered adverse device effects (ADEs), which could include unanticipated adverse device effects (UADEs). Study-related adverse events (AEs, serious adverse events [SAEs], ADEs, and UADEs), will be collected, documented, analyzed, and reported as applicable. “Study-related” is defined as related to a study activity listed in the protocol SoA (refer to Section 2.3, Schedule of Activities) [SOA]).

The safety of the study will be overseen by the study investigator, study Sponsor, reviewing IRBs, REBs, FDA, and a Data & Safety Monitoring Board (DSMB).

9.8.2. Adverse Events (AEs) and Serious Adverse Events (SAEs)

The definition of an AE is any untoward medical occurrence in a participant or study participant, temporally associated with the use of a study test, whether or not considered related to the study test. An AE can therefore be any unfavorable and unintended sign (including an abnormal laboratory finding), symptom, or disease (new or exacerbated) temporally associated with the use of the study test.

AEs will be reported by the participant (or, when appropriate, by a caregiver, surrogate, or the participant's legally authorized representative).

The PI and any qualified designees are responsible for detecting, documenting, recording, and reporting events that meet the definition of an AE, and remain responsible for following up AEs that are serious, considered related to the study test or study procedures, or that caused the participant to discontinue the [study test/study].

An SAE is an AE that meets any of the following criteria:

- Led to death;
- Led to serious deterioration in the health of the participant, that resulted in:
  - A life-threatening illness or injury. The term ‘life-threatening' in the definition of serious refers to an event in which the participant was at risk of death at the time of the event. It does not refer to an event that could have caused death, if it were more severe;
  - Inpatient or prolonged hospitalization;
  - A permanent impairment of a body structure or a body function; or
  - Medical or surgical intervention to prevent life-threatening illness or injury or permanent impairment to a body structure or a body function; or
- Led to fetal distress, fetal death or a congenital abnormality or birth defect.

Planned hospitalization for a pre-existing condition without serious deterioration in health, is not considered an SAE.

The investigator or designated member of the study team should document the SAE into GRAIL EDC (or complete a sponsor-provided SAE report if EDC is unavailable) within twenty-four (24) hours of notification/occurrence of the event, but in no event later than 10 working days after the investigator first learns of the effect. The study investigator will report the SAE to the IRB/IEC/REB according to the institution's IRB/IEC/REB reporting requirements.

After the initial SAE report, the study investigator is required to follow each participant at subsequent visits/contacts. All study-related AEs will be followed until resolution, stabilization, the event is otherwise explained, or the participant is lost to follow-up (as defined in Section 8.2, Lost to Follow-Up).

9.8.3. Adverse Device Effects (ADEs)

An ADE is an event related to the use of an investigational medical device. This definition includes AEs resulting from insufficient or inadequate instructions for use, deployment, implantation, installation, or operation, or any malfunction of the investigational medical device. This definition includes any event resulting from use error or from intentional misuse of the investigational medical device. The PI and any qualified designees are responsible for detecting, documenting, recording, and reporting events that meet the definition of an ADE or UADE and remain responsible for following up on ADEs that are serious, considered related to the study or study procedures, or that caused the participant to discontinue the study (see Section 8.1, Participant Withdrawal from a Study).

The PI will monitor for the occurrence of ADEs per the standard of care for each participant undergoing a study-specified blood draw. AEs that are associated with the blood collection tubes and AEs associated with the test result report (i.e. return of results to the participant) should be considered and documented as an ADE. Determination should be based on assessment of temporal relationships, biologic plausibility, association (or lack of association) with other procedures done as standard of care, and presence (or absence) of a more likely cause. The PI will report ADEs to the IRB/IEC/REB according to the institution's IRB/IEC/REB reporting requirements.

9.8.4. Unanticipated Adverse Device Effects (UADEs)

A UADE is any serious adverse effect on health or safety; any life-threatening problem or death caused by or associated with a device, if that effect, problem, or death was not previously identified in nature, severity, or degree of incidence in the application (including supplementary plan or application) or any other unanticipated serious problem associated with a device that is relates to the rights, safety, or welfare of participants.

Clinical study site personnel will complete a GRAIL-provided UADE report within 24 hours of notification/occurrence of the event. The PI will report the UADE to the IRB/IEC/REB according to the institution's IRB/IEC/REB reporting requirements.

The means of obtaining AE data should be described (volunteered, checklist, or questioning), as should any specific rating scales used and any specifically planned follow-up procedures for specific AE, or any planned rechallenge procedures in case study intervention is discontinued because of an AE.

9.8.5. Follow-Up of ADEs & UADEs

After the initial ADE/UADE report, the PI is required to proactively follow each participant at subsequent visits/contacts. All ADEs and UADEs will be followed until resolution, stabilization, the event is otherwise explained, or the participant is lost to follow-up (as defined in Section 8.2., Lost to Follow-Up).

9.8.6. Regulatory Reporting Requirements for UADEs

- Prompt notification by the PI to GRAIL of a UADE is essential so that legal obligations and ethical responsibilities towards the safety of participants and the safety of a study under clinical investigation are met.
- GRAIL has a legal responsibility to notify both the local regulatory authority and other regulatory agencies about the safety of a study under clinical investigation. GRAIL will comply with country-specific regulatory requirements relating to safety reporting to the regulatory authority, IRB/IEC/REB, and PIs.
- PI safety reports must be prepared for suspected UADEs according to local regulatory requirements and GRAIL policy and forwarded to PIs as necessary.
- A PI who receives a PI safety report describing a UADE or other specific safety information (e.g, summary or listing of UADEs) from GRAIL will review and then file it along with the PI's Brochure and will notify the IRB/IEC/REB, if appropriate according to local requirements.

9.8.7. Test Deficiencies and Concerns with the Test

A test deficiency is a perceived inadequacy of the investigational test with respect to its identity, quality, durability, reliability, safety, or performance, that does not result in an AE or ADE. Test deficiencies include but are not limited to malfunctions and inadequate labeling.

9.9. Study or Study Site Closure, Termination and Suspension

- GRAIL, the PI, or the IRB/IEC/REB reserves the right to close the study site or terminate the study at any time, for any reason at the sole discretion of GRAIL. Study sites will be closed upon study completion. A study site is considered closed when all required documents and study supplies have been collected and a study site closure visit has been performed. This can occur prior to study completion or upon study completion.
    - If GRAIL terminates or suspends the study, the PI must inform the IRB/IEC/REB and provide the IRB/IEC/REB a detailed written explanation of the termination or suspension.
    - If the PI terminates or suspends the study, PI must inform GRAIL, and the IRB/IEC/REB, and provide both GRAIL and the IRB/IEC/REB with a detailed written explanation of the termination or suspension.
    - If the IRB/IEC/REB terminates or suspends its approval of the study, the PI should notify GRAIL and provide GRAIL with a detailed written explanation of the termination or suspension.

- Reasons for the early closure of a study site by GRAIL or PI may include but are not limited to:
  - Failure of the PI to comply with the protocol, the requirements of the IRB/IEC/REB or local health authorities, GRAIL's procedures, or ICH/GCP guidelines.
  - Inadequate recruitment of participants by the PI.
  - Discontinuation of further study development.

9.10. Healthcare Resource Utilization

Healthcare resource utilization data will be collected from EMR and reported into the eCRF by the study investigator or designated member of the study team for all participants throughout the study. Clinical protocol-mandated procedures (e.g. blood draw) are excluded.

The data collected may be used to conduct future exploratory economic analyses, and will include:

Number and types of medical encounters and cancer-specific diagnostic procedures, including clinical lab visits, imaging tests, invasive tests, and clinic visits.

10. Statistical Considerations

10.1. Analysis Methods

This section contains a brief overview of the statistical analyses planned for this study. Formal Statistical Analysis Plan (SAP) will provide full details of the analyses. The SAP will be finalized and approved prior to conducting any analyses.

This is a descriptive study to assess the safety and performance of the MCED test. No formal hypothesis testing is planned. The results will be summarized using descriptive statistics and visual displays for each of the endpoints to enable evaluation of the implementation of an investigational MCED test in clinical practice.

10.2. Sample Size Determination

The sample size of this descriptive study is driven by an ability to enroll participants in the specific study subgroups based on age and sex to obtain information on diagnostic evaluation triggered by the MCED test result. The study will enroll at least 10,000 participants or as many as required to achieve at least 100 analyzable positive MCED test results. Assuming that approximately 5% of participants may be excluded due to various clinical and assay evaluability reasons, approximately 9500 analyzable participants would be expected for analyses related to receiving MCED test results and associated diagnostic evaluation. Based on previous studies we expect MCED test specificity to be in the range of 99-99.5%.

The first primary analysis objective is focused on safety evaluation. Table 7 illustrates the levels of precision that would be obtained for the primary safety Measure A ("Number of FP participants with an invasive procedure divided by the total number of analyzable participants") under six scenarios

representing two levels of FP rate (1%, 0.5%) and proportions of FP participants with invasive procedures ranging from 10% to 50%.

We estimate that approximately 25% of FP participants may have an invasive procedure assuming that distribution of cancer signal origin predictions in FP is consistent with expected cancer incidence multiplied by MCED test sensitivity and that all colorectal, 20% of breast and 5% of all other predicted cancer signal origins get invasive procedures. Based on the FP rate and the proportion of FP participants with invasive procedures, Measure A is estimated to range from 0.05% to 0.5% across these scenarios (Table 7).

For reference, the proportion of FP with invasive procedures among all screened participants for existing guideline recommended screening tests is estimated as:

- 1.66%[15] for screening mammography
- 10.2%[16] for colorectal screening w/ Cologuard
- 1.88%[17] for LDCT lung cancer screening

The expected 95% one-sided upper confidence bound for Measure A (Table 7) is substantially lower than the reported point estimates for the existing screening tests. No formal comparison to existing screening tests is planned for this study but for illustrative purposes only this study would provide a high power (>99%) for such a comparison even with the lowest estimate reported above (1.66% for screening mammogram) used as a threshold, i.e. to test a null hypothesis that Measure A is above 1.66%.

Table 7. Estimated Precision for Safety Measure A

Specificity	FP rate	% of FP with Invasive Procedures	Measure A[1]	Upper Bound of One-Sided 95% Wilson CI for Measure A
a	b = 1 - a	c	b * c	
99%	1%	10%	0.10%	0.18%
	1%	30%	0.30%	0.40%
	1%	50%	0.50%	0.64%
99.5%	0.5%	10%	0.05%	0.11%
	0.5%	30%	0.15%	0.23%
	0.5%	50%	0.25%	0.35%

[1] % of FP participants with an invasive procedure among the total number of analyzable participants with invasive procedures.
Note: For simplicity it is assumed that the FP rate reflects the proportion of FP participants among all analyzable participants (and not just those without cancer). This leads to a conservative slight overestimation of Measure A.

The second primary analysis objective is focused on MCED test performance. The expected number of positive test results and the number of cancers detected through diagnostic evaluation triggered by MCED test in analyzable participants was estimated using microsimulations described in Appendix IV. The results of the microsimulations in Table 8 indicate that approximately 117-164 positive MCED test results are expected with approximately 70 true positives (TP) and 46 to 94 FP. Based on these results, we expect to observe PPV of approximately 43% and 61% if MCED test specificity is 99% and 99.5%, respectively. The confidence intervals provided in Table 8 represent 2.5th and 97.5th percentiles of PPV values observed across simulations. If PPV of 43% is observed in a study with 164 MCED test positive results, the two-sided Wilson 95% CI around PPV estimate will be (35.1%, 50.3%). If PPV of 61% is observed in a study with 117 MCED test positive results, the two-sided Wilson 95% CI around PV estimate will be (51.6%, 69.1%). Table 8 also provides expected NPV estimates based on microsimulations.

Table 8. Results of Microsimulations[1]

Specificity	Number of Test Positive Results (95% CI)	% Test Positive (95% CI)	Number of True Positives (95% CI)	PPV (%) (95% CI)	NPV (%) (95% CI)
99%	164 (139, 189)	1.73 (1.46, 1.99)	70 (56, 91)	43.0 (35.6, 50.4)	98.5 (98.2-98.7)
99.5%	117 (99, 138)	1.23 (1.04, 1.45)	71 (58 ,86)	60.7 (51.7, 69.3)	98.5 (98.2-98.7)

[1] Based on SEER cancer incidence and reflecting age distribution in Table 6 (15% in 50-59 yr group, 50% in 60-69 yr group, 30% in 70-79 yr group and 5% in 80+).
Note: entries in the table represent median (2.5 and 97.5 percentiles) of a specific measure across 200 simulations as described in Appendix IV.

10.3. Populations for Analyses

For the purpose of the analysis, several populations are defined in Table 9.

Table 9. Populations for Analyses

Population	Description
Enrolled	Consented participants who satisfy the inclusion and exclusion criteria at the time of consent.
Clinically Evaluable	Consented participants who satisfy the inclusion and exclusion criteria and are clinically evaluable. Evaluability reasons will be prespecified in the Clinical Data Management Plan prior to analysis.
Analyzable	Clinically evaluable participants with evaluable MCED test results.
Cancer Signal Detected	Analyzable participants with positive MCED test result
Cancer Signal Not Detected	Analyzable participants with negative MCED test result

10.4. Statistical Analyses

The SAP will be developed and finalized before database lock and will describe the participant populations to be included in the analyses, analysis methods, and the procedures accounting for missing data. This section is a summary of the planned statistical analyses of the primary and secondary study objectives.

10.4.1. General Considerations

A summary of participant disposition will be provided for all enrolled participants. This summary will present the number and percentage of participants in each analysis set (Section 10.3) and whether they had cancer diagnosis or not during the study.
The analysis of the primary and secondary study objectives will be based on the Analyzable set unless otherwise specified.

Analysis results will be presented using descriptive statistics. For categorical (including binary) variables, the number and percentage of participants in each category will be presented. For continuous variables, the number of participants (n), mean, standard deviation (SD), median, first quartile (Q1), third quartile (Q3), minimum, and maximum will be presented.

The two-sided 95% confidence intervals (CIs) will be constructed using the Wilson (score) method (Wilson, 1927[18]) for performance measures such as PPV which are not very near 0 or 1. Modified Wilson (score) confidence intervals[19-21] will be used for performance measures such as specificity and NPV which are expected to be near 1.

As part of exploratory sensitivity analyses the results will be reported by presence of prior cancer history.

10.4.2. Primary Analyses

Primary analysis Objective 1

Evaluate the safety of the MCED test in terms of diagnostic testing triggered by the MCED test result.

Safety endpoints are the number and type of invasive procedures (as described in Appendix II) among FP participants.

The safety measures will describe the use of invasive procedures and include the following:

- Primary measure
    - Measure A: Number of FP participants with an invasive procedure divided by the total number of analyzable participants.
- Secondary measures:
    - Measure B: Number of invasive procedures among FP participants divided by the total number of analyzable participants.
    - Measure C: Number of FP participants with an invasive procedure divided by the total number of

participants with a positive MCED test

- Measure D: Number of invasive procedures among FP participants divided by the number of participants with a positive MCED test

The number of invasive procedures among FP participants and Measures A-D will be evaluated for all invasive procedures, minimally invasive and surgical procedures as described in Appendix II.

The results will be reported for invasive procedures prior to diagnostic resolution, during the targeted diagnostic testing triggered by MCED test result and conducted prior to confirmatory PET-CT (if applicable), and during diagnostic testing after confirmatory PET-CT.

Primary Analysis Objective 2

Evaluate performance of the MCED Test in individuals eligible for cancer screening.

Test performance endpoint is a diagnosis of invasive cancer as defined in Section 4.

Performance of the MCED test will be evaluated using performance measures defined based on a 2x2 Table 11 below which will be constructed based on the cancer status at a) 1 year and b) 2 years.

Table 11. 2x2 Contingency Table

		Invasive Cancer	
		**Present**	**Absent**
**MCED Test Result for Cancer Detection**	**Positive**	TP	FP
	**Negative**	FN	TN

Where

- TP = number of participants with true positive MCED test results,
- FP = number of participants with false positive MCED test results,
- FN = number of participants with false negative MCED test results,
- TN = number of participants with true negative MCED test results.

The following performance measures will be evaluated:

- PPV = TP / (TP + FP)
- NPV = TN / (TN + FN)
- Sensitivity = TP/ (TP + FN)
  - Sensitivity is estimated here based on the detection method which assumes that cases which are incorrectly screened as negative will manifest the disease clinically in a short interval such as 1 year[22]. The detection method is used widely to report sensitivity estimates of existing USPSTF approved screening tests in clinical trials such NLST and NELSON[23] while recognizing its limitations due to the fact that not all cancers present at time 0 may manifest

clinically within a given period of time and that some cancers which are not present at time 0 may get clinically diagnosed. To mitigate the first limitation sensitivity which could lead to overestimation of sensitivity, sensitivity will be evaluated at several time points including 1 year and 2 years.

- Specificity = TN / (TN + FP)
  - Specificity is also estimated based on detection method and hence is subject to similar limitations. However, due to overall relatively low incidence of cancer (<2%), its estimate is expected to be less affected. Similar to sensitivity, it will be evaluated at 1 year and 2 years.
- Observed cancer detection rate = TP / (TP + FP + FN + TN)
- NNS = (TP + FP + FN + TN) / TP
- CSO accuracy = (number of participants with accurate CSO prediction) / TP
  - Accuracy will be evaluated for the first CSO, second CSO prediction and either (first or second) CSO prediction.

Test performance will be evaluated at several time points:

- At the time of diagnostic resolution. At this time only performance measures based on positive MCED results can be evaluated: PPV and CSO accuracy.
- 1 year
- 2 years

The detailed definitions of each performance measure at different timepoints will be provided in the SAP.

As part of sensitivity analyses, test performance will be assessed by presence of prior cancer history.

10.4.3. Secondary Analyses

Secondary Analysis Objective 1

Assess participants reported anxiety resulting from use of the MCED test.

Anxiety following the MCED test result is assessed using STAI and MCED STAI questionnaires in all participants at baseline (pre-test), post-test and at 1 year (+/-30D). In addition, MCED test positives will be assessed at diagnostic resolution.

Descriptive statistics will be provided for a total score at each time point and change from baseline. A two-sided t-test will be used to determine if mean changes from baseline are significantly different from zero at each post-baseline assessment at 0.05 significance level. If differences in scores do not follow a normal distribution, Wilcoxon signed rank test (Wilcoxon, 1945) will be used instead. No adjustment for multiple testing is planned. The results will be summarized descriptively for positive and negative MCED test results, separately.

Secondary Analysis Objective 2

Evaluate intention and utilization of guideline recommended cancer screening procedures after use of the

MCED test.

Intention to follow standard of care cancer screening tests will be assessed using participant-reported questionnaire pre-test, post-test and at 1 year (+/-30D) in all participants. It is reported as Very Unlikely, Unlikely, Likely, or Very Likely to follow the healthcare provider's cancer screening recommendations.

Number and proportion of participants with each rating will be reported at each time point for the following analysis sets: Cancer Signal Not Detected, Cancer Signal Detected, Analyzable. Change from baseline (pre-test) to post-baseline assessments at each time point will be illustrated for each analysis set by:

- Proportion of participants who changed their rating from Likely or Very Likely at baseline to Unlikely or Very Unlikely post-baseline
- Proportion of participants who changed their rating from Unlikely or Very Unlikely at baseline to Likely or Very Likely post-baseline
- A matrix of baseline (rows) vs post-baseline (columns) ratings.

Utilization of standard of care cancer screening tests will be assessed in the period of time prior to study enrollment, during the first year and during the first two years after the initial MCED test is assessed. The use of most screening tests declined during pandemic[24] so only years prior to 2020 will be considered to establish a baseline for screening use. This study is expected to start enrollment in the second half of 2021 when cancer screening use may still be affected by COVID-19 to some degree so a comparison to prior utilization of cancer screening will need to be taken with caution. Utilization of cancer screening prior to enrollment will be compared to that post-test; analysis methods will be described in the SAP.

Based on use of cancer screening prior to year 2020 participants will be classified as compliant if they followed cancer screening recommendations (Appendix III), or non-compliant. Utilization of cancer screening during the study will be assessed for all participants, previously compliant and previously non-compliant participants, separately.

The results will be assessed in participants with negative MCED test (the main focus of this analysis), those with positive MCED test and in all participants.

Secondary Analysis Objective 3

Describe the ability of PET-CT to detect cancer in cases for which cancer signal origin directed workups do not result in diagnosis of cancer (initial workup negative cases).

The observed cancer detection rate for PET-CT will be calculated as a proportion of participants with cancer diagnosis in all participants with confirmatory PET-CT. The distribution of cancer types and clinical stage will be reported.

Secondary Analysis Objective 4

Characterize the number and type of diagnostic evaluations by test result, predicted cancer signal origin and outcome of diagnostic resolution.

The extent of diagnostic testing will be assessed as follows. Number and type of diagnostic procedures will be summarized per-participant and overall in the Signal Detected Analysis Set for imaging procedures (including anatomical and functional imaging) and for invasive procedures (minimally invasive, surgical) as described in Appendix II. Number of participants with at least one imaging or at least one invasive procedure will be reported. Distribution of the type of the first diagnostic procedure will be summarized. The distribution of the per-participant counts will be summarized using descriptive statistics (as described in section 10.4.1).

Time required to achieve diagnostic resolution is defined as the number of days from when the test result is returned through the reporting portal to the date of diagnostic resolution; it will be summarized using descriptive statistics by outcome of diagnostic resolution and using Kaplan-Meier methods to account for participants who did not reach a resolution at the time of the analysis.

The extent of diagnostic testing will be assessed across all participants and in the following subgroups:

- CSO prediction groups (solid tumors with common screening options, solid tumors without common screening options, hematologic malignancies)
- Top predicted CSO, where sample size allows (e.g. number of CSO calls ≥ 5)
- Number of reported CSOs (one vs two)
- Outcome of diagnostic resolution (cancer vs no cancer diagnosis)

The extent of diagnostic testing will be summarized for:

- All diagnostic testing prior to diagnostic resolution,
- The targeted diagnostic testing triggered by MCED test result and conducted prior to confirmatory PET-CT (if applicable), and
- The diagnostic testing starting from confirmatory PET-CT (inclusive) and conducted until diagnostic resolution.

In addition, the results will be evaluated for participants with diagnostic evaluations consistent with the expected targeted diagnostic testing for predicted CSO(s). The details of the analysis will be prespecified in the SAP.

Secondary Analysis Objective 5

Evaluate the radiation exposure due to the test result-directed evaluations.

Per participant radiation exposure will be assessed. Distribution of radiation exposure will be evaluated across all test positive participants, FP, and TP participants. The results will be reported for:

- All diagnostic testing prior to diagnostic resolution,
- The targeted diagnostic testing triggered by MCED test result and conducted prior to confirmatory

PET-CT (if applicable), and

- The diagnostic testing starts from confirmatory PET-CT (inclusive) and conducted until diagnostic resolution

Secondary Analysis Objective 6

Evaluate concordance of initial GRAIL test result and CSO prediction, with results from the research blood draw.

The results of the MCED test from D1 blood draw and research blood draw will be compared in participants who had both blood draws. It should be noted that research blood draws can be obtained only in participants with positive initial MCED test results. The number of negative and positive MCED results from research blood draw will be tabulated. In addition, top CSO predictions will be compared for initial vs research blood draws and proportion of cases with the same top one CSO among participants with positive MCED results from both the initial and research blood draws will be reported.

Secondary Analysis Objective 7

Assess participants reported outcomes and perceptions about the MCED test.

SF-12v2 is assessed in all participants at baseline (pre-test), post-test and at 1 year (+/-30D). In addition, MCED test positives will be assessed at diagnostic resolution. MICRA is assessed in all participants post-test, and satisfaction and attitude towards subsequent MCED testing are assessed post-test in MCED test negative and post diagnostic resolution in MCED test positive participants (Table 3).

The results from participant-directed questionnaires will be summarized at assessment time points for items, total score, domains and scales as appropriate for each survey (e.g. norm-based Physical and Mental Health Composite Scales computed for SF-12v2; Distress, Uncertainty and Positive experience scales for MICRA). Descriptive statistics will be provided for a total score at each time point and change from baseline.

For SF-12v2 a two-sided t-test will be used to determine if mean changes from baseline are significantly different from zero at each post-baseline assessment at 0.05 significance level. If differences in scores do not follow a normal distribution, Wilcoxon signed rank test (Wilcoxon, 1945) will be used instead. No adjustment for multiple testing is planned. The results will be summarized descriptively for positive and negative MCED test results, separately.

10.4.4. Safety Assessments

Study-related adverse events as described in Section 9.8 will be summarized by whether they were related to study procedure or investigational test, severity (mild, moderate, severe), whether they were considered an SAE, whether they were considered a UADE, and outcome (e.g. resolved, resolved with sequelae, ongoing at 30 minutes after end of blood draw).

10.4.5. Interim Analyses

Several interim analyses will be conducted to support evaluation of the MCED test safety and performance. No hypothesis testing is planned for the primary objectives of this study and there will be no stopping early for the overwhelmingly positive results; therefore, no type I error adjustments are planned.

The following interim analyses are planned:

- When diagnostic resolution is achieved for all participants or at least 6 months after the last enrolled participant has a Day 1 blood draw, whichever comes first
  - This analysis will provide a safety evaluation (primary objective 1), test performance evaluation (primary objective 2) for measures based on positive MCED test results, and focus on the secondary analysis objectives related to confirmatory PET-CT, extent of diagnostic testing and radiation exposure (secondary objectives 3, 4 and 5)
- After 1 year follow **up** is obtained, i.e 1 year + 30D after the last enrolled participant has a Day 1 blood draw
- After 2 year follow up is obtained, i.e 2 years + 30D after the last enrolled participant has a Day 1 blood draw

Additional analyses after 3 year follow up is obtained will be conducted for the exploratory objectives. Analysis methods for the interim analyses will be prespecified in the SAP.

11. Data & Safety Monitoring Board (DSMB)

A Data and Safety Monitoring Board (DSMB) will be utilized to assess the progress of the clinical study, the safety data, and key endpoints and provide recommendations to the study Sponsor on whether to continue, modify or stop the study. The DSMB is intended to provide additional assurance of participant safety and trial integrity.

The study Sponsor will appoint members of the DSMB and ensure appropriate composition with respect to relevant expertise and experience, including a clinician(s) with expertise in relevant clinical specialties and at least one biostatistician knowledgeable about statistical methods for clinical trials and sequential analysis of trial data. The DSMB will meet periodically to review cumulative study data to evaluate safety, study conduct, and scientific validity and data integrity of the study.

12. Supporting Documentation & Operational Considerations

12.1. Appendix I, *Regulatory, Ethical & Study Oversight Considerations*

12.1.1. Appendix I(a), *Regulatory & Ethical Considerations*

12.1.2. Appendix I(b), *Financial Disclosure & Participant Compensation*

12.1.3. Appendix I(c), *Data Protection*

12.1.4. Appendix I(d), *Study Supplies*

12.1.5. Appendix I(e), *Source Documents*

12.1.6. Appendix I(f), *Study Data Quality Assurance*

12.1.7. Appendix I(g), *Record Retention*

12.1.8. Appendix I(h), *Publication Policy*

12.2. Appendix II, Examples of Imaging and Invasive Tests

12.3. Appendix III, Cancer Screening Compliance

12.4. Appendix IV, Description of the Simulations

12.5. Appendix V, Abbreviations

12.6. Appendix VI, References

12.7. Appendix VII, Clinical Study Protocol Amendment History

Appendix I. Regulatory, Ethical & Study Oversight Considerations

Appendix I(a). Regulatory & Ethical Considerations

- The study sites will start enrolling upon receipt of written approval by GRAIL.
- This study will be conducted in accordance with the protocol and with the following:
    - Consensus ethical principles derived from international guidelines including the Declaration of Helsinki and Council for International Organizations of Medical Sciences (CIOMS) International Ethical Guidelines.
    - Applicable ICH/GCP guidelines.
    - The provisions specified in 21 CFR 11, 21 CFR 50, 21 CFR 54, 21 CFR 56, and 21 CFR 812.
    - Other applicable laws and regulations.
    - The study will maintain the following controls of study materials:
        - The protocol, protocol amendments, ICF(s), Instructions for Use (IFU), and other relevant documents (e.g, advertisements, other participant-facing materials) must be submitted to an IRB/IEC/REB by the PI and reviewed and approved by the IRB/IEC/REB before the study is initiated.
        - Any amendments to the protocol will require IRB/IEC/REB approval before implementation of changes made to the study design, except for changes necessary to eliminate an immediate hazard to study participants.
        - Clinical documents will be maintained according to the protocol and applicable laws and GCP standards to demonstrate the validity of the study and the integrity of the data collected.
        - Regulatory authorities, the IRBs/IECs/REBs, and/or GRAIL, or its designees or partners, may request access to all source documents, electronic CRFs (eCRFs), and other study documentation for audits or inspections, including on-site.

Obligations of PIs

It is the responsibility of the PI(s) to:

- Perform the study in accordance with ICH/GCP and the applicable regulatory requirements.
- Ensure compliance with all procedures required by the protocol.
- Provide the protocol-required data elements to GRAIL, in a manner that is attributable, legible, contemporaneous, and accurate.
- Comply with any privacy and security requirements applicable to their institution or organization.

Appendix I. Regulatory, Ethical & Study Oversight Considerations (Cont.)

Appendix I(a). Regulatory & Ethical Considerations

- Provide GRAIL representatives and/or other authorized individuals with direct access to clinical documents in order to allow for Source Data Review (SDR) and/or audit/inspection activities to be conducted.
- Understand the applicable laws, GCP standards, and protocol, and agree to abide by them.
- Provide written summaries of the status of the study to the IRB/IEC/REB annually or more frequently in accordance with the requirements, policies, and procedures established by the IRB/IEC/REB. At the end of the study, a summary of the study's outcome should be prepared for the IRB/IEC/REB.
- Document, report, and notify the IRB/IEC/REB of UADEs or other significant safety findings as required by IRB/IEC/REB procedures.
- Provide oversight of the conduct of the study at the study site and adhere to requirements of Title 21, Code of Federal Regulations (21 CFR), ICH/GCP guidelines, the IRB/IEC/REB, European regulation 536/2014 for clinical studies (if applicable), and all other applicable local regulations.

Obligations of the Sponsor

It is the responsibility of GRAIL, the Sponsor of this study to:

- Ensure the proper conduct of the study, including ethical considerations, protocol compliance, and integrity and validity of study data.
- Monitor the study execution to ensure a high level of ethical, scientific, technical, and regulatory quality.

At regular intervals during the study, the study sites will be contacted, through monitoring visits, letters, or telephone calls, by a representative of GRAIL to review study progress, PI and participant compliance with protocol requirements, and any emergent problems.

Appendix I. Regulatory, Ethical & Study Oversight Considerations

Appendix I(b). Financial Disclosure & Participant Compensation

PIs and Sub-Investigators will provide GRAIL with sufficient and accurate financial information, as requested to allow GRAIL to submit complete and accurate financial certification or disclosure statements to the appropriate regulatory authorities. PI and Sub-Is are responsible for providing information on financial interests during the course of the study and for one year after completion of the study.

Financial compensation will not be provided to participants in this study.

Confidential Property of GRAIL, Inc.

Appendix I. Regulatory, Ethical & Study Oversight Considerations

Appendix I(c). Data Protection

- The collection of participants' data for this protocol is described in Section 9.5, Clinical Data Collection.
- Participants will be assigned a unique identifier (ID) by GRAIL. Any participant records or datasets that are transferred to GRAIL will contain the identifier only; participant names or any information which would make the participant identifiable will not be transferred.
- The participant must be informed that their personal study-related data will be used by GRAIL in accordance with local data protection law. The level of disclosure must also be explained to the participant.
- The participant must be informed that their medical records may be examined by Clinical Quality Assurance auditors or other authorized personnel appointed by GRAIL, by appropriate IRB/IEC/REB members, and by inspectors from regulatory authorities.

Appendix I. Regulatory, Ethical & Study Oversight Considerations

Appendix I(d). Study Supplies

- Supplies and materials required for blood collection and shipping will be provided by GRAIL or its designee. GRAIL may provide tablets for electronic consent and questionnaire administration, as applicable by site requirements. The disposition of study supplies after study completion or termination is controlled by the Clinical Trial Agreement between GRAIL and the relevant clinical institution or study sites

- Blood collection tubes, study test kits (including shipping materials) will be supplied and replenished at participating study sites on an as-needed basis. These supplies and detailed instructions for blood collection, handling, and shipping will be provided by GRAIL or its designee.

- Clinical blood collection (study test kits and shipping materials) should be stored in a secure location at each participating clinical site. Arrangements can be made to have these supplies replenished more frequently so as to reduce the number that need to be stored at any given time. The blood collection packages will have expiration dates that will be located on the vacutainer tubes and these should be regularly checked prior to use. Expired clinical blood collection packages should be discarded, per GRAIL's instruction and clinical site operating procedures.

Appendix I. Regulatory, Ethical & Study Oversight Considerations

Appendix I(e). Source Documents

- Source documents provide evidence for the existence of the participant and substantiate the integrity of the data collected. Source documents are filed at the investigative study site.
- Data reported in the eCRF that are transcribed from source documents must be consistent with the source documents or the discrepancies must be explained. The PI may need to request previous medical records or transfer records, depending on the study. Also, current medical records must be available.
- Definition of what constitutes source data can be found in Source Data Location Form.

Appendix I. Regulatory, Ethical & Study Oversight Considerations

Appendix I(f). Study Data Quality Assurance (QA)

Regardless of the method(s) for study data collection, the data will be stored and displayed as an eCRF. The PI is responsible for affirming that the data recorded in the CRF are accurate and complete, to the best of their knowledge, by providing their signature.

- The PI must maintain accurate documentation (source data) that supports the information entered in the CRF, unless the CRF is being utilized as a source document.
- The PI must permit study-related monitoring, audits, IRB/IEC/REB review, and regulatory agency inspections and provide direct access to source data documents and CRFs.
- Clinical study Monitors will perform ongoing source data verification (SDV) to confirm that data entered into the CRF by authorized study site personnel are accurate, complete, and verifiable from source documents; that the safety and rights of participants are being protected; and that the study is being conducted in accordance with the currently approved protocol and any other study agreements, ICH/GCP, and all applicable regulatory requirements.

EDC systems used to capture and manage study data will allow for manual and automated checks to ensure that the records are complete, consistent, and accurate. Data discrepancies and resolutions will be managed within the EDC system. A proportion of electronic record-based items will be subject to SDR against the original medical record for accuracy, as applicable. GRAIL or its designee will monitor the clinical data captured in the EDC system by the study sites according to the approved Clinical Data Management Plan (CDMP) and Clinical Study Monitoring Plan (SMP), respectively. The degree to which data quality will be monitored by GRAIL and at each study site, will be determined by taking a risk-based approach. The EDC system will store audit trails of data entry and modification throughout the course of the study, as well as records of activities such as monitoring, data discrepancy resolution, PI signature, and locking of data.

Appendix I. Regulatory, Ethical & Study Oversight Considerations

Appendix I(g). Record Retention

Under 21 CFR 812 - Investigational Device Exemptions, Section 140 - Records, Part D - Retention Period, the study records must be retained for two years after the latter of the following two dates: (i) the date on which the investigation is terminated or completed, or (ii) the date that the Premarket Approval (PMA) is approved.

No records may be destroyed during the retention period without the written approval of GRAIL. No records may be transferred to another location or party without written notification to GRAIL.

Appendix I. Regulatory, Ethical & Study Oversight Considerations

Appendix I(h). Publication Policy

Any publication or other disclosure of the results of this study, including any publication in manuscript form and any presentation at scientific or medical meetings, will be made in accordance with the terms of the Clinical Trial Agreement (CTA) between GRAIL and the center or study site responsible for performing activities for the study.

Appendix II. Examples of Imaging and Invasive Tests

Category	Specific procedure
Anatomical Imaging Test	Plain X-Ray
	CT
	MRI
	Mammogram
	Ultrasound (thyroid, abdominal, pelvic)
	Transvaginal Ultrasound
Functional Imaging Test	PET-CT
	PET-MRI
	Nuclear Medicine Scan
Minimally Invasive Procedure	Esophagogastroduodenoscopy (EGD)
	Colonoscopy
	Endoscopic Ultrasound
	ERCP
	Bronchoscopy
	Cystoscopy
	Hysteroscopy
	Fine needle aspiration of the thyroid gland
	Liver biopsy
	Thoracentesis
	Pulmonary arterio-venous malformation embolization
Surgical procedure	Surgery + Biopsy

Appendix III. Cancer Screening Compliance

The following items (as applicable) need to be satisfied in order for a participant to be considered compliant with cancer screening recommendations:

1. Lung cancer: annual LDCT in participants satisfying USPSTF criteria[25].

2. Colon cancer: annual FOBT, annual FIT, FIT-DNA every 3 years, sigmoidoscopy every 5 years, or colonoscopy every 10 years in participants <=75 years old[26].

3. Breast cancer: mammography every 2 years in women 50-74 years old[15].

4. Cervix cancer: pap smear with HPV testing every 5 years, or pap smear every 3 years in women <=65 years old[27].

Appendix IV. Description of the Simulations

The following assumptions were used in simulations:

1. Cancer incidence in the study population.
   a. Cancer incidence is estimated based on the cancer incidence in Surveillance, Epidemiology, and End Results Program (SEER) in years 2006-2015 for the following 17 cancers: anus and anorectum, bladder, cervix uteri, colon and rectum, corpus and uterus, esophagus, head and neck, breast, kidney, liver and intrahepatic bile duct, lung, lymphoma, melanoma, ovary, pancreas, prostate, stomach.
   b. SEER incidence is weighted to reflect the following age distribution (consistent with Table 6): 15% in 50-59 yr group, 50% in 60-69 yr group, 30% in 70-79 yr group and 5% in 80+ yr group.
2. MCED test performance was estimated based on the results from the analysis of >4,000 participants from the Circulating Cell-free Genome Atlas study with an overall sensitivity of approximately 52% across cancer types[10].
3. Because the natural history of cfDNA-detectable cancer cases is not established, we explored a range of plausible assumptions for the distribution of dwell times (ie, how much time each cancer spends in a given stage) per cancer per stage (Hubbell et al 2020).

Participant histories were simulated by using incidence rates for clinical detection per year per cancer type and stage as described above. Exponential time to clinical detection of first cancer was generated for each participant, cancer type was generated by drawing from a multinomial cancer type distribution and then cancer stage at clinical detection was generated by drawing from multinomial stage distribution. Starting from the time of the clinically detected cancer and using the corresponding dwell times, the times when participants progress through the previous stages and when cancer started was back simulated. If cancer started before time 0 (time of the study test), it is expected to be detected with assumed sensitivity for a given cancer and stage. A total of 200 simulations were conducted and a median, 2.5th and 97.5% percentiles for the number of “signal detected” test results, number of cancers detected through diagnostic evaluation following a “signal detected” test result and performance measures across simulations were reported.

Appendix V. Abbreviations

Term	Definition
AACR	American Association for Cancer Research
ADE	Adverse Device Effect
AE	Adverse Effect
AJCC	American Joint Committee on Cancer
ASCO	American Society of Clinical Oncology
BMI	Body Mass Index
CBC	Complete Blood Count
CCGA	Circulating Cell-free Genome Atlas
CDMP	Clinical Data Management Plan
CDR	Cancer Detection Rate
cfDNA	Cell-free DNA
cfNA	Cell-free nucleic acids
CFR	Code of Federal Regulations
CI	Confidence Interval
CIOMS	Council for International Organizations of Medical Sciences
CLIA	Clinical Laboratory Improvement Amendments
CSO	Cancer Signal Origin
CT	Computed Tomography
CTA	Clinical Trial Agreement
DNA	Deoxyribonucleic Acid
DOB	Date of Birth
DSMB	Data and Safety Monitoring Board
ePRO	Electronic Participant-Reported Outcome
ESMO	European Society for Medical Oncology
HIPAA	Health Insurance Portability and Accountability Act
eCRF	Electronic Case Report Form
EDC	Electronic Data Capture
EMR	Electronic Medical Records
EOS	End of Study

ESMO	European Society for Medical Oncology
FDA	Food and Drug Administration
FP	False Positive
GCP	Good Clinical Practice
ICF	Informed Consent Form
ICH	International Council for Harmonization of Technical Requirements for Pharmaceuticals for Human Use
ID	Identifier
IEC	Independent Ethics Committee
IFU	Instructions For Use
IRB	Institutional Review Board
IV	Intravenous
LDCT	Low dose computed tomography
MCED	Multi-Cancer Early Detection
MICRA	Multidimensional Impact of Cancer Risk Assessment
MRI	Magnetic Resonance Imaging
NCCN	National Comprehensive Cancer Network
NGS	Next Generation Sequencing
NIH-AARP	National Institutes of Health - American Association of Retired Persons
NNS	Number Needed to Screen
NPV	Negative Predictive Value
PDs	Protocol Deviations
PET-CT	Positron Emission Tomography–Computed Tomography
PI	Principal Investigator
PPV	Positive Predictive Value
QA	Quality Assurance (in appendix)
REB	Research Ethics Board
ROR	Return of Result
SAE	Serious Adverse Event
SAP	Statistical Analysis Plan
SDR	Source Data Review
SDV	Source Data Verification

SEER	Surveillance, Epidemiology, and End Results Program
SMP	Study Monitoring Plan
SMS	Short Message Service
SoA	Schedule of Activities
STAI	State Trait Anxiety Inventory
TRF	Test Requisition Form
TRR	Test Result Report
TP	True Positive
UADE	Unanticipated Adverse Device Effects

Appendix VI. References

1. Imperiale TF, Ransohoff DF, Itzkowitz SH, et al. Multitarget stool DNA testing for colorectal cancer screening. New England Journal of Medicine. 2014; 370: 1287–97.
2. Liu MC, Oxford GR, Klein EA. Sensitive and specific multi-cancer detection and localization using methylation signatures in cell-free DNA. Annals of Oncology. 2020; 31(6):745-759.
3. Aravanis A, Oxnard G, Maddala T, et al. Development of plasma cell-free DNA (cfDNA) assays for early cancer detection: first insights from the Circulating Cell-Free Genome Atlas Study (CCGA). In: Proceedings of the American Association for Cancer Research Annual Meeting 2018; Apr 14-18, 2018; Chicago, IL. Philadelphia (PA): AACR; Cancer Res. 2018;78(13 Suppl): Abstract LB-343.
4. Oxnard G, et al. Genome-wide sequencing for early stage lung cancer detection from plasma cell-free DNA (cfDNA): The Circulating Cancer Genome Atlas (CCGA) study. Presented at: American Society of Clinical Oncology (ASCO) 2018; June 1-5, 2018;
Chicago, IL. J Clin Oncol. 2018;36(no. 18_suppl): Abstract LBA8501.
5. Liu M, et al. Breast cancer cell-free DNA (cfDNA) profiles reflect underlying tumor biology: The Circulating Cell-Free Genome Atlas (CCGA) study. Presented at: American Society of Clinical Oncology (ASCO) 2018; June 1-5, 2018; Chicago, IL. J Clin Oncol. 2018;36(no. 15_suppl): Abstract 536.
6. Klein E, et al. Development of a comprehensive cell-free DNA (cfDNA) assay for early detection of multiple tumor types: The Circulating Cell-free Genome Atlas (CCGA) study. Presented at: American Society of Clinical Oncology (ASCO) 2018; June 1-5, 2018; Chicago, IL. J Clin Oncol . 2018;36(no. 15_suppl): Abstract 12021.
7. Swanton C, et al. Prevalence of clonal hematopoiesis of indeterminate potential (CHIP) measured by an ultra-sensitive sequencing assay: Exploratory analysis of the Circulating Cancer Genome Atlas (CCGA) study. Presented at: American Society of Clinical Oncology (ASCO) 2018; June 1-5, 2018; Chicago, IL. J Clin Oncol . 2018;36(no. 15_suppl): Abstract 12003.
8. Liu M, et al. Plasma cell-free DNA (cfDNA) assays for early multi-cancer detection: the Circulating Cell-Free Genome Atlas (CCGA) study. Presented at: European Society for Medical Oncology (ESMO) 2018; October 20, 2018; Munich, Germany. Annals Oncol. 2018;29(suppl_8):viii14-viii57.
9. Liu M, Jamshidi A, Venn O, et al. Genome-wide cell-free DNA (cfDNA) methylation signatures and effect on tissue of origin (TOO) performance. Presented at: American Society of Clinical Oncology (ASCO) 2019; May 31- June 4, 2019. Chicago, IL. J Clin Oncol. 2019;37(15_suppl):3049. Cohn A, Seiden M, Kurtzman K, et
10. Klein EA, et al. Clinical validation of a targeted methylation-based multi-cancer early detection test. Presented at: American Association for Cancer Research Virtual Annual Meeting, Week 1; April 10-15, 2021. Abstract LB013.
11. Cohn A, Seiden M, Kurtzman K, et al. 2019. The Circulating Cell-free Genome Atlas (CCGA) study: follow-up (F/U) on non-cancer participants with cancer-like cell-free DNA signals. Poster session presented at: American Society of Clinical Oncology (ASCO) 2018; June 1-5, 2018; Chicago, IL.
12. Marteau TM, Bekker H. The development of a six-item short-form of the state scale of the Spielberger state-trait anxiety inventory (STAI). Br J Clin Psychol 1992;31 (Pt 3:301–6)
13. https://www.acr.org/-/media/ACR/Files/Practice-Parameters/FDG-PET-CT.pdf
14. Definitions, Statistics and Graphs: Statistics. United States: National Institutes of Health: National Cancer Institute Website. https://cancercontrol.cancer.gov/ocs/statistics/statistics.html Updated February 8, 2019.
15. Breast Cancer: Screening. U.S. Preventive Services Website. https://uspreventiveservicestaskforce.org/uspstf/recommendation/breast-cancer-screening

16. Imperiale TF, Ransohoff DF, Itzkowitz SH, Levin TR, Lavin P, Lidgard GP, Ahlquist DA, Berger BM. Multitarget stool DNA testing for colorectal-cancer screening. N Engl J Med. 2014 Apr 3;370(14):1287-97. doi: 10.1056/NEJMoa1311194. Epub 2014 Mar 19. PMID: 24645800. All false positives undergo colonoscopy, consider as invasive procedure.
17. National Lung Screening Trial Research Team, Aberle DR, Adams AM, et al. Reduced lung-cancer mortality with low-dose computed tomographic screening. N Engl J Med. 2011;365(5):395-409. doi:10.1056/NEJMoa1102873. table 3 % of Y0 screened individuals with invasive procedure subtracting % diagnosed with lung cancer.
18. Wilson, E. B. (1927). Probable Inference, the Law of Succession, and Statistical Inference. Journal of the American Statistical Association, 22(158), 209-212.
19. Newcombe, R. G. (1998a), "Two-sided confidence intervals for the single proportion: Comparison of seven methods," Statistics in Medicine, 17, 857–872.
20. Newcombe, R. G. (1998b), "Interval estimation for the difference between independent proportions: Comparison of eleven methods," Statistics in Medicine, 17, 873–890.
21. Agresti, A., and Coull, B. A. (1998), "Approximate is better than "exact" for interval estimation of binomial proportions," The American Statistician, 52, 119–126
22. Day NE. Estimating the sensitivity of a screening test. J Epidemiol Community Health. 1985;39(4):364-366. https://www.ncbi.nlm.nih.gov/pmc/articles/PMC1052473/
23. Klaveren, R. J. Management of Lung Nodules Detected by Volume CT Scanning. The New England Journal of Medicine. N Engl J Med 2009; 361:2221-2229. DOI: 10.1056/NEJMoa0906085 https://www.nejm.org/doi/full/10.1056/nejmoa0906085
24. Chen RC, Haynes K, Du S, Barron J, Katz AJ. Association of Cancer Screening Deficit in the United States With the COVID-19 Pandemic. JAMA Oncol. Published online April 29, 2021. doi:10.1001/jamaoncol.2021.0884
25. Lung Cancer: Screening. U.S. Preventive Services Website. https://uspreventiveservicestaskforce.org/uspstf/recommendation/lung-cancer-screening
26. Colorectal Cancer: Screening. U.S. Preventive Services Website. https://uspreventiveservicestaskforce.org/uspstf/recommendation/colorectal-cancer-screening
27. Cervical Cancer: Screening. U.S. Preventive Services Website. https://uspreventiveservicestaskforce.org/uspstf/recommendation/cervical-cancer-screening

Appendix VII. Clinical Study Protocol Amendment History

Clinical Study Protocol Amendment Summary for the current protocol amendment is located directly before the Table of Contents (TOC).

Clinical Study Protocol Amendment [amendment number]: Protocol V0.1 (original version)

Clinical Study Protocol

PATHFINDER 2

Clinical Study Protocol Title: The PATHFINDER 2 Study: Evaluating the Safety and Performance of the GRAIL Multi-Cancer Early Detection Test in an Eligible Screening Population

Clinical Study Protocol Number: GRAIL-012

Version Number: 4.0

Version Date: 21-JAN-2025

Sponsor Name: GRAIL, Inc.

Sponsor Address: 1525 O'Brien Drive, Menlo Park, CA 94025

Sponsor Phone Number: (833) 694-2553

Medical Monitor: [redacted]

Medical Director Lead: [redacted]

Medical Monitor Contact Information: [redacted]

FDA Investigational Device Exemption (IDE) Application Number: G210176

Sponsor Approvals

This clinical study protocol has been approved by GRAIL, Inc. (GRAIL). The following signature(s) document this approval.

[redacted]

Medical Director Lead
GRAIL, Inc.

22-Jan-2025
Date

[redacted]

Medical Monitor
GRAIL, Inc.

22-Jan-2025
Date

[redacted]

Clinical Trial Manager
GRAIL, Inc.

22-Jan-2025
Date

Sponsor Approvals

This clinical study protocol has been approved by GRAIL, Inc. (GRAIL). The following signature(s) document this approval.

[redacted]
Regulatory Affairs
GRAIL, Inc.

22-Jan-2025
Date

[redacted]
Data Management Lead
GRAIL, Inc.

22-Jan-2025
Date

[redacted]
Biostatistics Lead
GRAIL, Inc.

28-Jan-2025
Date

The information contained in this document is the property of or under control of GRAIL, and is provided to you in confidence. Except as required by federal, state, or local laws or regulations, this document may not be disclosed to others, in whole or in part, without the prior written permission of GRAIL.

Statement of Good Clinical Practice Compliance

The study will be conducted in accordance with the International Council for Harmonization of Technical Requirements for Pharmaceuticals for Human Use (ICH) - ICH Harmonized Guideline - Integrated Addendum to ICH E6(R1): Guideline for Good Clinical Practice E6(R2) (ICH/GCP), and the United States Department of Health & Human Services, Food & Drug Administration (FDA) - Code of Federal Regulations (CFR), Title 21 - Food & Drugs, Chapter I - Food & Drug Administration, Department of Health & Human Services, Subchapter A - General, Part 11 - Electronic Records: Electronic Signatures (21 CFR 11); Part 50 - Protection of Human Subjects (21 CFR 50); Part 54 - Financial Disclosure by Clinical Investigators (21 CFR 54); Part 56 - Institutional Review Boards; Subchapter H - Medical Devices, Part 812 - Investigational Device Exemption (21 CFR 812). All personnel involved in the conduct of this clinical study have completed human subjects protection training.

Principal Investigator Signature Page

The signature below constitutes the approval of this clinical study protocol (protocol) and the attachments, and provides the necessary assurances that this clinical study will be conducted according to all stipulations of the protocol, including all statements regarding confidentiality, and according to applicable federal, state, and local requirements including regulations administered by the Food & Drug Administration (FDA) (such as of 21 CFR Part 50 and 21 CFR Part 812) and International Conference on Harmonization of Technical Requirements for Registration of Pharmaceuticals for Human Use (ICH) guidelines. The Investigator also agrees to conduct the investigation in accordance with the investigational plan and any conditions of approval imposed by the reviewing Institutional Review Board (IRB)/Research Ethics Board (REB) or Food & Drug Administration (FDA). In addition, the Investigator agrees to supervise all testing of the device involving human subjects and ensure that the requirements for obtaining informed consent are met.

Principal Investigator Name: ____

Principal Investigator Title: ____

Principal Investigator Signature: ____________________________________

Date: ____

Table of Contents

1. Revision History **9**
2. Protocol Summary **10**
2.1. Clinical Study Protocol Synopsis 10
2.2. Schema 15
2.3. Schedule of Activities (SOA) 15
3. Introduction **18**
3.1. Study Rationale 19
3.2. Background of the Study Test 19
3.3. Benefit/Risk Assessment 22
4. Objectives & Endpoints **24**
5. Study Design **28**
5.1. Overall Design 28
5.2. Estimated Study Duration 32
5.3. Study Duration for Study Participants 32
5.4. Study Design Schema 33
5.5. Scientific Rationale for Study Design 33
5.6. Study Enrollment Definition 33
5.7. End-of-Study Definition 33
6. Study Population **33**
6.1. Inclusion Criteria 34
6.2. Exclusion Criteria 35
6.3. Participant Recruitment 35
6.4. Informed Consent Process 35
7. Measures to Minimize Bias **36**
7.1. Blinding Methods 37
7.2. Participant Follow-Up 37
7.2.1. Active Clinical Follow-Up 37
7.3. Participant Retention Through Follow-Up 37
8. Participant Withdrawal **38**
8.1. Participant Withdrawal from the Study 38
8.2. Lost to Follow-Up 39
9. Study Assessments & Procedures **39**
9.1. Enrollment 40
9.2. Participant Questionnaire 40

9.3. Blood Draw 40
9.4. Return of Results 41
9.5. Diagnostic Work-Up of a "Cancer Signal Detected" Test Result 42
9.6. Clinical Data Collection 43
9.7. Safety Assessments 45
9.7.1. General 45
9.7.2. Adverse Events (AEs) and Serious Adverse Events (SAEs) 45
9.7.3. Adverse Device Effects (ADEs) 46
9.7.4. Unanticipated Adverse Device Effects (UADEs) 47
9.7.5. Follow-Up of ADEs & UADEs 47
9.7.6. Regulatory Reporting Requirements for UADEs 47
9.7.7. Test Deficiencies and Concerns with the Test 47
9.8. Study or Study Site Closure, Termination and Suspension 48
9.9. Healthcare Resource Utilization 48
10. Statistical Considerations 48
10.1. Analysis Methods 48
10.2. Sample Size Determination 49
10.3. Populations for Analyses 51
10.4. Statistical Analyses 51
10.4.1. General Considerations 51
10.4.2. Primary Analyses 52
10.4.3. Secondary Analyses 54
10.4.4. Safety Assessments 60
10.4.5. Interim Analyses 60
11. Independent Data Monitoring Committee (IDMC) 60
12. Supporting Documentation & Operational Considerations 61
Appendix I(a). Regulatory & Ethical Considerations 62
Appendix I(b). Financial Disclosure 64
Appendix I(c). Data Protection 65
Appendix I(d). Study Supplies 66
Appendix I(e). Source Documents 67
Appendix I(f). Study Data Quality Assurance (QA) 68
Appendix I(g). Record Retention 69
Appendix I(h). Publication Policy 70
Appendix II. Examples of Imaging and Invasive Tests 71
Appendix III. Cancer Screening Compliance 72

Appendix IV. Description of the Simulations 73
Appendix V. Abbreviations 74
Appendix VI. References 77
Appendix VII. Clinical Study Protocol Amendment History 79

1. Revision History

Refer to Appendix VII, *Clinical Study Protocol Amendment History* for details on protocol version history.

Version	Date Revised DD-MMM-YYYY	Revised By	Description of Revision
1.0	09-JUN-2021	N/A	Initial Version
2.0	28-FEB-2022	Jack Youngren	Refer to Appendix VII
2.1	01-APR-2022	Dave Nguyen	Clarify adverse event definition
3.0	09-AUG-2023	Jack Youngren	Refer to Appendix VII
4.0	21-JAN-2025	Jack Youngren	Refer to Appendix VII

2. Protocol Summary

2.1. Clinical Study Protocol Synopsis

Protocol Title: The PATHFINDER 2 Study: Evaluating the Safety and Performance of the GRAIL Multi-Cancer Early Detection (MCED) Test in an Eligible Screening Population

Protocol Short Title: PATHFINDER 2

Rationale: The purpose and rationale is to evaluate the safety and performance of the GRAIL Multi-Cancer Early Detection (MCED) Test in an eligible screening population.

Table 1. Objectives & Endpoints Table

Objectives	Endpoints
**Primary**	
1. Evaluate the safety of the MCED test in terms of diagnostic testing triggered by the MCED test result.<br><br>2. Evaluate performance of the MCED Test in individuals eligible for cancer screening.	1. Safety endpoints: Number and type of invasive procedures (as described in Appendix II) performed, and number and type of adverse events due to diagnostic testing in all participants with a positive MCED test result.<br><br>The safety measures will describe the use of invasive procedures and include the following:<br><br>Primary measure:<br>○ Measure A: Number of participants with a positive MCED test result who undergo an invasive procedure divided by the total number of analyzable participants.<br><br>Secondary measures:<br>○ Measure B: Number of invasive procedures among participants with a positive MCED test result divided by the total number of analyzable participants.<br>○ Measure C: Number of participants with a positive MCED test result and an invasive procedure divided by the total number of participants with a positive MCED test<br>○ Measure D: Number of invasive procedures among participants with a positive MCED test result divided by the number of participants with a positive MCED test.

Objectives	Endpoints
	Similar analyses will be conducted for true positive (TP) and false positive (FP) participants, separately.<br><br>2. Test performance endpoint: Diagnosis of cancer.<br><br>The MCED test performance measures include positive predictive value (PPV), negative predictive value (NPV), episode sensitivity, specificity, positive likelihood ratio, negative likelihood ratio, cancer signal origin (CSO) accuracy, MCED cancer detection rate, and number needed to screen to detect a cancer (NNS) and will be evaluated based on cancer status at multiple timepoints (e.g. at diagnostic resolution, 1 year, 2 years).
**Secondary**	
1. Assess participant-reported anxiety resulting from use of the MCED test<br><br>2. Evaluate intention to use, and utilization of, guideline recommended cancer screening procedures after use of the MCED test.<br><br>3. Describe the ability of PET-CT to detect cancer in cases for which cancer signal origin-directed workups do not result in diagnosis of cancer (initial workup negative cases).<br><br>4. Characterize the extent of diagnostic testing triggered by the MCED test result by predicted cancer signal origin and outcome of diagnostic resolution.<br><br>5. Evaluate the radiation exposure during diagnostic evaluation.<br><br>6. Evaluate concordance of initial GRAIL test result and CSO prediction, with results from the research blood draw<br><br>7. Assess participant-reported outcomes	1. Anxiety will be assessed by State Trait Anxiety Inventory (STAI)+ MCED STAI questionnaires pre-test (baseline), post-test, at diagnostic resolution (if applicable), and at 1 year (+30D). Changes from the baseline will be evaluated at each time point.<br><br>2. Intention to follow standard of care cancer screening tests will be assessed pre-test, post-test, at 1 year (+30D). Changes from the baseline will be evaluated at each time point. Utilization of standard-of-care cancer screening tests prior to study enrollment, during the first year and the second year (+/-30D) after the initial MCED test will be assessed. Utilization during the study will be compared to that in a period prior to enrollment.<br><br>3. Diagnosis of cancer after confirmatory PET-CT administered in participants where diagnostic resolution of cancer diagnosis is not achieved through the targeted diagnostic testing. Proportion of cancers detected by PET-CT will be calculated.<br><br>4. Number and type of diagnostic procedures, and time to diagnostic resolution, will be collected. The extent of diagnostic testing will be assessed across all test positive participants, FP participants, and true positive (TP) participants. The results will be reported for all diagnostic testing prior to diagnostic resolution, the targeted diagnostic

Objectives	Endpoints
and perceptions about the MCED test.<br>8. Evaluate Performance of the MCED Test in Selected Demographic and Clinical Subgroups.<br><br>9. Evaluate MCED Test Positive Rate in Subgroups with Potential Cross-Reactive Conditions.	testing triggered by MCED test result and conducted prior to confirmatory PET-CT (if applicable), and for diagnostic testing starting from confirmatory PET-CT and conducted until diagnostic resolution.<br><br>5. Per participant radiation exposure during diagnostic evaluation will be assessed. Distribution of radiation exposure will be evaluated across all test positive participants, FP, and TP. The results will be reported for all diagnostic testing prior to diagnostic resolution, the targeted diagnostic testing triggered by MCED test result and conducted prior to confirmatory PET-CT (if applicable), and for diagnostic testing starting from confirmatory PET-CT and conducted until diagnostic resolution.<br><br>6. MCED test results for cancer detection and CSO prediction from the initial and research blood draws (in the subset of participants with a research blood draw) will be assessed. Agreement measures will be calculated.<br><br>7. Participant-reported outcomes will be assessed at different time points:<br>○ Health-related quality of life following the MCED test assessed by SF-12v2<br>○ Multidimensional Impact of Cancer Risk Assessment (adapted MICRA)<br>○ Satisfaction with the MCED test<br>○ Attitudes towards subsequent MCED screening<br>Changes from the baseline will be evaluated at each time point for SF-12v2.<br><br>8. Test performance endpoints (PPV, NPV, episode sensitivity, specificity, PLR, NLR, CSO accuracy, and combined cancer signal detection and CSO accuracy) will be evaluated in selected demographic and clinical subgroups, representing different categories of age, sex, race, ethnicity, BMI, smoking or cancer history.<br><br>9. MCED test positive rate, defined as the proportion of participants with MCED positive test results out of all participants with analyzable MCED test results, will be

Objectives	Endpoints
	evaluated in participants with the following conditions:<br>○ Non-malignant hematologic conditions (Langerhans cell histiocytosis, Lymphoproliferative disorders, Mastocytoma, Monoclonal B-cell lymphocytosis, Monoclonal gammopathy of undetermined significance, Post-transplant lymphoproliferative disorder, other)<br>○ Participants who took any cytotoxic medications in the past year<br>○ Participants who took any demethylating agents in the past year<br>○ Participants with a prior cancer history<br>○ Medical conditions which may shed cfDNA and/or may affect methylation
**Exploratory**	
1. Evaluate performance of the MCED test in year 3.<br>2. Evaluate changes in blood-based biomarkers among the subset of participants with a research blood draw.<br>3. Evaluate different CSO reporting classes based on clinically relevant subgroups.	1. Diagnosis of invasive cancer during year 3. The MCED test performance measures include positive predictive value (PPV), negative predictive value (NPV), sensitivity, specificity, cancer signal origin (CSO) accuracy, MCED cancer detection rate, and number needed to screen to detect a cancer (NNS) and will be measured based on cancer status at year 3.<br>2. Blood biomarker signals from the initial and research blood draws in the subset of participants with a research blood draw will be assessed.<br>3. MCED test results for CSO prediction and blood biomarker signals from the initial and research blood draws will be evaluated in the context of diagnostic testing triggered by the test results.

Overall Design: This is a prospective, multi-center, interventional study of the GRAIL MCED Test with return of results in healthcare systems in North America.

The study will enroll at least 35,000 and no more than 38,500 participants over an anticipated enrollment period of approximately 36 months. Following informed consent, the participant will receive the GRAIL MCED Test. Test results will be returned to the healthcare providers and information on the ensuing diagnostic work-up will be captured. Participant disposition will be categorized into the following groups:

- GRAIL MCED positive: Participants will undergo diagnostic procedures based on the test returned cancer signal origin(s) (CSO). Diagnostic procedures corresponding to cancer signal origin are specified in Table 4. If a participant is unable to undergo the prescribed procedure, or the institution physician determines an alternative work-up is necessary, the institution physician will document the reason for deviating from the prescribed procedure(s) and list the alternative procedure(s) performed.
    - For participants where diagnostic resolution of cancer diagnosis is achieved through the targeted diagnostic testing based on the CSO, clinical information including but not limited to cancer type, pathologic, imaging and clinical staging information will be captured.
    - For participants where diagnostic resolution of cancer diagnosis is not achieved through the targeted diagnostic testing and PET-CT has not been used as part of diagnostic evaluation, a PET-CT will be performed to assess cancer status, henceforth referred to the ‘confirmatory PET-CT’ for the purposes of this study. For participants where diagnostic resolution of cancer is achieved through the confirmatory PET-CT, clinical information including but not limited to cancer type, pathologic, imaging, and clinical staging information will be captured. Participants in this group will also undergo a research blood draw around the time of the confirmatory PET-CT. Results of this research blood draw will not be provided back to the physician or participant.

- GRAIL MCED negative: Participants will be notified of “cancer signal not detected” test results by the study investigator or designated member of the study team, be reminded not to interpret “cancer signal not detected” test results as the absence of cancer and to continue to adhere to guideline-recommended cancer screening. Participants will continue to be followed as outlined in this protocol.

All participants will be actively followed by the enrolling institution for 3 years to assess for cancer status, uptake of routine cancer screening and other data collection with analyses performed at interim time points. In addition to a baseline questionnaire, additional participant-reported outcomes will be collected at multiple time points before and after test results are returned.

A subsequent version of the test, MCED-I will be under development during the conduct of this study. Once MCED-I is available for use in this study, residual blood samples from some participants collected at day 1 (Table 2) may be tested using this updated version. Results from this testing will not be returned to study investigators or participants.

Independent Data Monitoring Committee: Yes

2.2. Schema

Figure 1. Study Design Schema

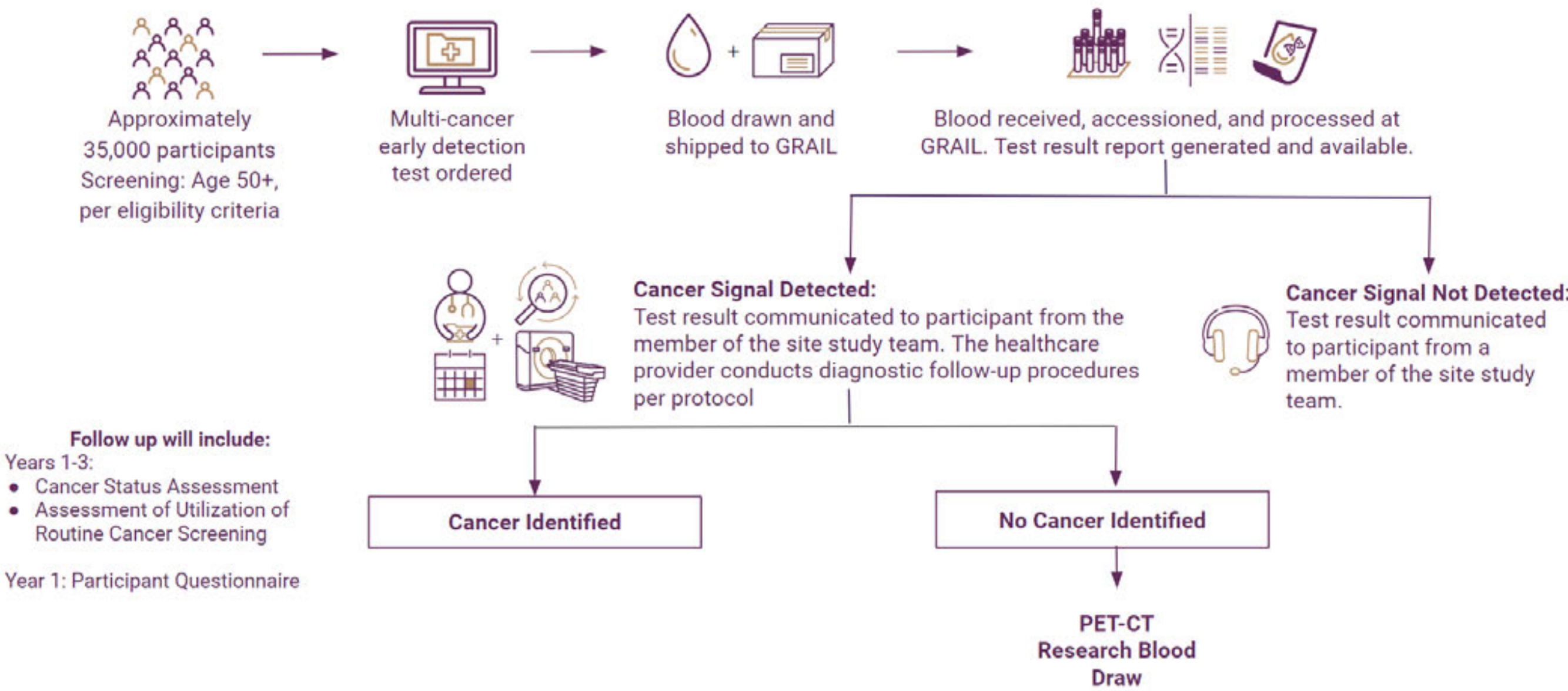

2.3. Schedule of Activities (SOA)

Table 2. Schedule of Activities Table

Activity	Enrollment	Day 1	Day 15	By Day ~30	Diagnostic Evaluation	At Diagnostic Resolution	1yr +/-30D	2yr +/-30D	3yr +/-30D (EOS)
Inclusion/Exclusion Criteria Review for Eligibility	x[c]	x[e,f]							
Informed Consent[a]	x								
Demographics and Baseline Assessment (Medical History, Cancer Screening History)	x								
Participant Questionnaires[b]	x[d]			x[h]		x[l]	x[m]		
Blood Draw for Study Test		x[f]							

Activity	Enrollment	Day 1	Day 15	By Day ~30	Diagnostic Evaluation	At Diagnostic Resolution	1yr +/-30D	2yr +/-30D	3yr +/-30D (EOS)
Assessment of Signs and Symptoms Prior to Blood Draw		x[n]							
Study Test Result Report Available to the Study Investigator and Study Team			x[g]						
Study Test Result Communicated to the Participant (“Return of Results”)				x[i]					
Cancer Status Assessment							x	x	x
**“Cancer Signal Detected” Test Result**: Diagnostic Procedures Performed as Outlined in Section 5.1					x				
**“Cancer Signal Detected” Test Result**: PET-CT & Research Blood Draw					x[j]				
Assessment of Utilization of Routine Cancer Screening (part of Cancer Status Assessment)							x	x	x
Study Related AE, SAE, and UADE Reporting and Review	x[k] (all time points)								

[a]Eligibility criteria should be confirmed based on medical record review prior to obtaining informed consent. Informed consent must be obtained prior to study blood draw and any study procedures being performed.
[b]See Table 3 for additional details regarding participant questionnaires.
[c]Inclusion/exclusion criteria are confirmed prior to enrollment.
[d]Baseline/Pre-Test participant questionnaires should be completed prior to the Day 1 blood draw for the study test.
[e]Eligibility should be confirmed based on medical record review on Day 1 prior to the blood draw. For blood draw visits occurring 31-60 days following consent, eligibility criteria must be re-confirmed prior to blood draw.

[f]The Day 1 blood draw visit may occur on the same day as informed consent (enrollment visit) but may occur up to 60 days following informed consent. Repeat blood draws will be requested if technical issues with samples preclude sample processing or reporting, but must also be completed within 60 days from informed consent.
[g]The test result report will be returned to the study investigator via paper or electronic methods (test results portal) typically within 15 calendar days from the date of blood collection, barring holidays and unforeseen circumstances affecting sample shipment and lab processing.
[h] Post-Return of Results participant questionnaires must be distributed no sooner than 7 days after communication of test results to the participant, and should be completed within 14 days.
[i]The test result should be communicated to the participant as soon as possible, typically within 15 days from the date of receipt of MCED test results. See Section 9.4.
[j]PET-CT and research blood draw will be collected on "cancer signal detected" cases where no cancer is identified by diagnostic procedures performed as outlined in section 5.1.
[k]"Study-related" is defined as related to a study activity listed in this table. See Section 9.8 for additional information.
[l] Post-Diagnostic Resolution participant questionnaires must be distributed no sooner than 7 days after communication of diagnostic resolution to the participant, and should be completed within 14 days.
[m]1yr participant questionnaires will be triggered at 12 months from the initial date of blood collection.
[n]Signs and Symptoms eCRF should be completed to record any abnormal signs and symptoms in the eCRF experienced by the participant within 90 days prior to the date of the initial blood draw for the study test.

Table 3. Participant-Reported Outcomes Instruments (Questionnaires)

Instrument	Baseline/Pre-Test (Prior to blood draw)	Post-Return of Results (ROR) (7-21 days after ROR)		Post-Diagnostic Resolution (7-21 days following)	1yr +30D
**Participant Group**	**All (22 items)**	**Cancer Signal Not Detected (47 items)**	**Cancer Signal Detected (43 items)**	**Cancer Signal Detected (25 items)**	**All (22 items)**
SF-12v2 (12 items)	X	X	X	X	X
State Trait Anxiety Inventory 6 (STAI; 6 items) + MCED STAI (3 items)	X	X	X	X	X
Multidimensional Impact of Cancer Risk Assessment (adapted MICRA; 21 items)		X	X		
Satisfaction with the MCED test (de novo; 3 items)		X		X	
Attitude towards adherence to current cancer screening guidelines (de novo; 1 item)	X	X	X		X
Attitude towards subsequent MCED test (de novo; 1 item)		X		X	

3. Introduction

Detecting cancers early, when treatment is more likely to be curative, is an important way to reduce overall cancer mortality. The introduction of screening programs to detect cancers of the breast and colon has resulted in fewer cancers being detected after metastasis and corresponding reductions in cancer specific mortality. However, to date, organized screening programs focus on single cancers, utilizing technologies to visualize small cancers via mammography (breast) and low dose computed tomography (LDCT) (lung). While much scientific effort has been directed towards finding cancer biomarkers which could be tested for in body fluids, those that have been found do not have widespread screening utility, with the exception of certain fecal biomarkers which can signal the presence of colorectal cancers[1].

A promising new biomarker technology for early cancer detection involves sequencing of the cell-free nucleic acids (cfNA) shed by dying cells and circulating in the blood. This approach presumes that cancers will shed nucleic acids that can be distinguished from those shed from healthy cells, and further that features of the signals will be localizable to the site of origin.

Preliminary work suggests that this technology can detect a variety of clinically diverse cancers and their site of origin with high specificity and could thereby be used to test for multiple cancers simultaneously[2]. If routine testing could detect multiple cancers earlier, including cancers with a high mortality rate for which there are currently no screening strategies for early detection, it could potentially have a greater impact on overall mortality due to cancer than current approaches, which address one cancer at a time.

There has been significant international interest in developing a blood test for early cancer detection. A blood test would be minimally invasive and would require minimal physical infrastructure to deliver, which could substantially improve access to cancer screening benefits. Screening persons for multiple cancers simultaneously would also be expected to detect cancers before they have metastasized, when treatment is likely to be more successful, thereby reducing overall cancer morbidity and mortality.

GRAIL, Inc. (GRAIL) has developed a multi-cancer early detection test that uses cell-free deoxyribonucleic acid (cfDNA) targeted methylation signals to distinguish cancer from non-cancer and identify the cancer signal of origin. Development and validation of this test is described in Section 3.2 below. Understanding the test's clinical utility, its relation to established diagnostic procedures, and other elements of its practical deployment as a screening tool are critical to its successful implementation in diverse clinical settings.

Confidential Property of GRAIL, Inc.

3.1. Study Rationale

This study is being conducted to demonstrate the safety and effectiveness of GRAIL's multi-cancer early detection (MCED) test. Results will be submitted to the FDA, in combination with results from additional clinical validation studies, in a Pre-Market Application (PMA). The study will continue to collect data that will be used in the post-approval setting.

Successful introduction of a novel MCED test warrants an assessment and consideration of both the potential benefit and risks of the test. Benefits include the opportunity to improve cancer outcomes by identifying cancer at earlier stages or prior to symptomatic presentation when interventions are often more effective. Risks include those associated with diagnostic evaluations and invasive procedures occurring in false positive cases, anxiety associated with test results, and risks associated with returning a cancer signal detected test result without a correctly predicted cancer signal origin (CSO) (eg, risks associated with the diagnostic evaluations). The study will also follow test-negative participants to assess the false negative rate and assess the impact on participants' adherence to standard of care cancer screening tests. Additionally, MCED test results may impact participants' understanding and perception of their risk of cancer. This protocol aims to assess these as part of its ultimate goal, to inform the broader implementation and dissemination of a blood-based multi-cancer screening paradigm.

3.2. Background of the Study Test

Study Test

The study test to be used in this protocol, GRAIL MCED test, is a qualitative, next-generation sequencing (NGS)-based screening test for the detection of a DNA methylation signal using cell-free DNA isolated from adult human peripheral whole blood. GRAIL's MCED test is a screening test and cannot be used to confirm a cancer diagnosis. The test result of "cancer signal detected" (also referred to as a "positive" result throughout this protocol) may indicate the presence of cancer, and should be followed by diagnostic tests. These diagnostic tests should be ordered by qualified health care professionals in accordance with professional guidelines that take into account the Cancer Signal Origin result from the MCED test.

The technology represents a new paradigm in early cancer detection, a two-step process for the detection of shared cancer signals followed by Cancer Signal Origin prediction. When a cancer signal is detected, Cancer Signal Origin(s) is reported. Depending on the relative strength of the prediction of the cancer signal origin, one or two signal origins are reported. The test has 21 possible Cancer Signal Origins: Anus; Bladder, Urothelial Tract; Bone and Soft Tissue; Breast; Cervix; Colon, Rectum; Head and Neck; Kidney; Liver/Bile Duct; Lung; Lymphoid Lineage; Melanocytic Lineage; Myeloid Lineage; Neuroendocrine; Ovary; Pancreas, Gallbladder; Plasma Cell Lineage; Prostate; Stomach, Esophagus; Thyroid Gland; Uterus.

The test does not test for or report clinically-relevant single genetic mutations.

Completed Studies

GRAIL developed technologies to assess cell-free nucleic acid (cfNA) signals using broad, deep sequencing to detect multiple types of genomic and epigenomic alterations including methylation status, single

nucleotide variants, insertions and deletions, copy number alterations, and structural rearrangements such as gene fusions. Multiple platforms, including analyses of whole genome and targeted methylation patterns, circulating RNA patterns and compartment specific factors (e.g. white blood cells and germline variants), have been tested to optimize sensitivity and specificity for an early detection blood test.

A preliminary evaluation of the GRAIL approach to blood markers was presented at the American Association for Cancer Research (AACR), American Society of Clinical Oncology (ASCO) and European Society for Medical Oncology (ESMO) annual meetings in 2018-2020[3-10]. The Circulating Cancer Genome Atlas (CCGA) study (Clinicaltrials.gov identifier: NCT02889978) is a large, prospective, longitudinal, multicenter clinical study which enrolled 15,254 participants with a recent diagnosis of cancer (n=8,584, 56%) and without a recent diagnosis of cancer (n=6,670; 44%), from 141 clinical institutions and multiple networks throughout the United States, as well as one site in Canada, from August 2016 to February 2019. The first CCGA classification analysis (CCGA1) consisted of 2,800 participants, including 1,650 participants with newly diagnosed cancer who had not yet received treatment and 1,150 non-cancer participants. CCGA1 was divided into a training set with 1,785 participants and an independent test set with 1,015 participants. Three prototype sequencing assays were performed on blood samples. The three prototype sequencing assays included targeted sequencing of paired cell-free DNA (cfDNA) and white blood cells to detect somatic (non-inherited) mutations such as single nucleotide variants and small insertions and/or deletions; whole-genome sequencing of paired cfDNA and white blood cells to detect somatic copy number changes; and whole-genome bisulfite sequencing of cfDNA to detect abnormal cfDNA methylation patterns. The prototype assays detected a highly specific, strong biological signal in cancer types that are typically not screened for and have low survival rates (five-year cancer-specific fatality rate of greater than 50%): lung, ovarian, pancreatic, liver, and esophageal cancers. For these cancers in the CCGA1 Training set at 98% specificity, the sensitivity for stages I-III cancers (N=117) was 54%, with the highest performing prototype assay, whole genome bisulfite sequencing. As expected, sensitivity increased to 90% for stage IV cancers (N=81). Through longitudinal follow-up of participants in the training and test set of CCGA1, it has since been confirmed that three of eight “non-cancer” participants who had an elevated signal have been diagnosed with cancer. This suggests the signal indicated the presence of undiagnosed cancer[11].

The second CCGA classification analysis (CCGA2) utilized a targeted methylation cfDNA assay that serves as the basis for the development of the classifier that distinguishes cancer from non-cancer and identifies the cancer signal origin. CCGA2 includes approximately 4,800 participants from CCGA, comprising approximately 2,800 cancer and 2,000 non-cancer participants, and an independent validation set of approximately 2,200 non-cancer participants. These participants are split into training (~70%) and validation (~30%) sets. Approximately 2,700 were excluded from the training and validation sets, due to ineligibility or un-evaluability, or because they were reserved for future analysis. An analysis population of approximately 3,050 (1,531 cancer and 1521 non-cancer) participants were included in the training set, and approximately 1,260 (654 cancer, 610 non-cancer) participants were included in the validation set. The classifier achieved a specificity of 99.8% versus 99.3% between the training and validation sets, respectively. Among all cancers, the overall sensitivity for cancer detection was 55.2% across all stages in the training set, and 54.9% in the validation set. In a pre-specified list of clinically significant cancers (anorectal, breast [HR-negative], colorectal, esophageal, gastric, head and neck, hepatobiliary, lung, lymphoid neoplasm [chronic lymphocytic leukemia, hairy cell leukemia, lymphoma], multiple myeloma, ovarian, pancreatic), overall sensitivity (stage I-IV) of 77.9% for cancer detection was achieved in the

training set versus 76.4% in the validation set. Additionally, the preliminary analysis showed that a tissue of origin could be returned in 96% of the cases, and amongst these cases, was correct in 93% of the cases, which was consistent across stages[2].

The third CCGA classification cohort (CCGA3) is a large (approximately 4,000 participants) Clinical Validation cohort that was used to assess the performance of the GRAIL test, which was developed based on CCGA2 and has been further refined for use as a screening tool, to lay the foundation for population-scale clinical implementation of the test. Data analysis has been completed for CCGA3.

The PATHFINDER Study (NCT04241796) was a prospective, interventional, multicenter study of participants receiving an investigational multi-cancer early detection test, developed by GRAIL. The study enrolled 6,662 participants without clinical suspicion of cancer from clinical sites in the United States. Participants were men and women at least 50 years old who were recruited into two cohorts. One cohort included participants with additional cancer risk (history of smoking, prior cancer with treatment completed >3 years ago, or genetic cancer predisposition). The other cohort included participants without additional cancer risk. Participants who received a "Cancer Signal Detected" result from an early version of the Galleri test underwent a diagnostic evaluation to assess whether they had cancer. Later, blood samples were reanalyzed with the current version of the Galleri test.

The primary objective of the PATHFINDER study was to assess the extent of diagnostic testing required to achieve diagnostic resolution following a "cancer signal detected" MCED test result.. Imaging procedures were used in a similar proportion of participants with cancer diagnosis at diagnostic resolution (90.9%) and those with no cancer diagnosis (92.9%). Invasive procedures were used more often in participants with cancer (81.8%) vs those with no cancer diagnosis (29.8%) at the end of the study. Notably, there was only 1 surgically invasive procedure among participants with no cancer at diagnostic resolution. The median time to diagnostic resolution was 78.5 days; it was shorter for participants with cancer diagnosis compared to participants without cancer diagnosis at resolution (57 vs 160 days, respectively). Few participants had adverse events (n=4); importantly no adverse events were observed due to diagnostic testing after "signal detected" MCED result.

The secondary objective of PATHFINDER study was to evaluate the performance of the MCED test for each test version. For the early test version, of 92 participants with a "cancer signal detected" MCED test result, 35 had a diagnosis of cancer, for a PPV of 38.0% (95% CI 28.8%-48.3%). The cancer signal origin accuracy was 85.3% (95% CI=69.9-93.6%) for the top cancer signal origin and increased to 97.1% (95% CI = 85.1-99.8%) when considering the top two cancer signal origin results. Among participants with one year follow up and without cancer diagnosis 99.1% had "cancer signal not detected result", i.e. specificity of 99.1% was observed. The current MCED test version returned fewer overall cancer signal detected results, most notably with a reduction of hematologic (lymphoid and plasma cell neoplasm) cancer signal origin (n= 53 for an early version compared to n=11 for the current version) which was consistent with the changes introduced in the current version of the Galleri test. Of 58 "cancer signal detected" cases, 25 had a diagnosis of cancer leading to an overall PPV for cancer detection of 43.1% (95% CI 31.2-55.9%). Similar to the early test version, the current test version had high cancer signal origin accuracy, with the top cancer signal origin accuracy of 84.0%, which increased to 88.0% when considering the second returned cancer signal origin. Overall specificity was 99.5% for the current MCED test version.

PATHFINDER participant feedback was generally positive, with a mean total satisfaction score of 83.3 on a 100 point scale. There was no evidence that the negative MCED test result induced any systematic change in self-reported likelihood of adherence to guideline recommended screening.

Ongoing Studies

The Circulating Cancer Genome Atlas (CCGA) study is ongoing with additional analyses planned.

The STRIVE Study (NCT03085888) is a prospective, observational, multicenter, longitudinal study of women undergoing screening mammography that will be used to support the development and validation of the GRAIL test for detection of multiple types of invasive cancers. The study has fully enrolled 99,252 participants from 35 clinical sites across the United States. While the enrollment strategy utilized screening mammography to identify women seeking screening for breast cancer, the study was designed to capture the broad range of cancers that develop in this population, allowing for analyses of the ability of the GRAIL test to detect multiple types of cancer prior to their clinical diagnosis.

The SUMMIT Study (NCT03934866) is a prospective, observational, multicenter, longitudinal study of men and women with significant smoking history that will be used to support the development and validation of the GRAIL test for detection of multiple types of invasive cancers. The study has enrolled approximately 13,000 participants in London and surrounding geographies. Participants have undergone a lung health check assessment and screening for lung cancer using low dose CT at baseline. The study is ongoing with additional screening timepoints at year 1 and year 2.

The NHS-Galleri study (ISRCTN 91431511) is a pragmatic, randomized controlled trial (RCT) intended to assess the performance and clinical utility of a multi-cancer early detection (MCED) test developed by GRAIL for population screening in the United Kingdom (UK) when added to standard of care. The study is being conducted in partnership with the National Health Service (NHS). The study has enrolled approximately 140,000 participants from sites across England. Participants were randomized to either the intervention arm, with blood collection and evaluation of the test with consequent investigation of a positive test through referral to the NHS urgent two week wait pathway, or to the control arm, where blood samples were collected at designated intervals and stored for potential future evaluation, but participants did not receive test results and otherwise continued to receive routine NHS care. Unless diagnosed with cancer, participants in both arms are asked to return for annual visits at approximately 12 and 24 months. All participants whether test positive, test negative or not tested are being followed for cancer and associated outcomes via NHS dataset linkages.

Additional clinical studies are planned.

3.3. Benefit/Risk Assessment

It is anticipated that some participants will receive a "cancer signal detected" test result and that the subsequent work-up will lead to a diagnosis of cancer, which could then be treated appropriately. This may result in an earlier diagnosis than if the participant were not involved in the study. Individual participants may not experience a direct benefit from participation. However, the study will also help GRAIL further evaluate the feasibility and acceptability of a multi-cancer early detection test.

Summary of Known Potential Risks to Study Participants

Risks Associated with Peripheral Venipuncture:

There are potential risks to study participants associated with peripheral venipuncture. The safety of peripheral venipuncture has been well established. When blood is drawn from a vein, there will be temporary discomfort and a minimal risk for slight bleeding, bruising, lightheadedness, slight irritation at the blood draw site and, in rare instances, infection or fainting.

Follow-Up Procedure-Related Risks:

Potential risks for study participants include those from diagnostic testing (procedure or imaging, e.g. endoscopy, colonoscopy, sigmoidoscopy, laparoscopy, biopsy, CT with or without intravenous (IV) contrast, magnetic resonance imaging (MRI), ultrasound, mammography, positron emission tomography–computed tomography (PET-CT)) as part of the diagnostic evaluation follow-up for a "cancer signal detected" test result. It is expected that the potential risks of any diagnostic procedures determined appropriate by the participant's medical team, which will be administered as part of clinical care, will be explained to the participant during the clinical consent process (in addition to other information, such as potential benefits, risks, and alternatives). The informed consent form (ICF) for this clinical protocol will describe the potential risks from the clinical diagnostic procedures. Diagnostic procedure potential risks include, but are not limited to:

- allergic reactions and respiratory problems, and in rare instances death from general anesthesia or sedatives that may be used with endoscopic procedures
- perforation of the colon, esophagus, or stomach by the colonoscope, sigmoidoscope, or endoscope
- prolonged or severe bleeding from endoscopy, colonoscopy, sigmoidoscopy, or laparoscopy injury
- infection from endoscopy, colonoscopy, sigmoidoscopy, or laparoscopy
- visceral injury and bleeding, injury to the bowel, or injury to the bladder from laparoscopy
- exposure to ionizing radiation from imaging procedures
- acute allergic or severe contrast reactions including acute renal failure, and in rare instances death from procedures using imaging contrast agents
- temporary discomfort, bruising, lightheadedness, and in rare instances infection or fainting associated with peripheral venipuncture

Follow-up procedures may also result in incidental findings unrelated to the MCED result. It is the responsibility of the study investigator(s) to determine how to address incidental findings related to diagnostic testing.

After the identification of a suspicious abnormality by any procedure(s) or imaging that is administered as a result of a "cancer signal detected" test result, additional imaging and/or biopsy and/or surgery

performed to obtain tissue for tumor analysis will be part of the participant's clinical care and will be determined by the participant's personal healthcare provider or study investigator, not by this protocol.

Risks Related to the Test Result:

There is a potential risk of receiving a test result indicating a cancer signal is detected when no cancer is present (sometimes referred to as "false positive"). A participant with this result may be recommended for unnecessary follow-up diagnostic procedures, and as a result, may incur physical and psychological risks and adverse events that are associated with those procedures.

There is also a potential risk of receiving a test result indicating no cancer signal is detected when cancer is actually present (sometimes referred to as a "false negative"). In this case, a participant may incorrectly assume that s/he does not have cancer, and may experience a delay in follow-up diagnostic procedures and subsequent delay in a potential cancer diagnosis. A "cancer signal not detected" test result should not be interpreted as absence of cancer and should not supersede guideline-recommended screening results or other standard of care diagnostic or treatment algorithms. Failure to adhere to guideline-recommended screening or other standard of care modalities could also lead to a delay in a potential cancer diagnosis.
The specificity and sensitivity performance characteristics in the CCGA study are described in Section 3.2.

Risks Related to Overdiagnosis:

With any screening test there is a risk of overdiagnosis – diagnosing subclinical disease that would never have progressed to clinical disease. In cancer screening, significant overdiagnosis is limited to screening tests that are sensitive to the very earliest cancers. It is not anticipated that overdiagnosis will be a substantial concern for the GRAIL MCED test because the test has lower sensitivity for stage I cancer and it is unlikely that many stage II and higher cancers are truly indolent. Overdiagnosis in cancer screening is a particular issue for prostate and breast cancers, both of which have very long progression times compared to most other cancers. It is of note that the GRAIL test has lower sensitivity for detection of these two cancers.

Questionnaire Related Risks:

The questionnaires used in this study may make participants feel uncomfortable. Participants are encouraged to speak to the study team regarding any concerns prompted by the questionnaires.

Additional Considerations:

It is not within the scope of this study to return results to participants related to genetic assessment for germline cancer risk. If such assessments are desired, such testing should be sought elsewhere through clinically available means and as determined by the participant's healthcare provider. Incidental findings, such as those related to chromosomal aneuploidies (e.g., XXY or mosaic trisomy 8), will not be reported.

4. Objectives & Endpoints

Primary Analysis Objectives and Endpoints

There are two primary study objectives:

1) Evaluate the safety of the MCED test in terms of diagnostic testing triggered by the MCED test result.

2) Evaluate performance of the MCED Test in individuals eligible for cancer screening.

Safety endpoints include the number and type of invasive procedures (as described in Appendix II) performed in all participants with a positive MCED test result. In addition, the type and number of adverse events due to diagnostic testing among all participants with MCED positive test will be reported. Safety measures for these endpoints are described in Section 10.4.2.

Test performance endpoints include measures associated with screening test performance for cancer detection and CSO prediction as described in Section 10.4.2. The endpoints will be assessed at multiple time points during the study including at diagnostic resolution following a signal detected result, at 1 year and 2 years.

Cancer is defined as the following:

- Invasive solid tumors, excluding non-metastatic basal cell carcinoma and squamous cell carcinoma of the skin

- Hematologic malignancies:
  - Lymphoma
  - Lymphoid leukemia
  - Plasma cell neoplasm/Multiple myeloma
  - Myeloid neoplasm
  - Additional malignant hematologic conditions representing proliferative or dysplastic disorders or neoplasms with behavior code 3 based on ICD-O-3

- Cancer diagnosis is supported by pathologic confirmation of an invasive solid tumor or hematologic malignancy, or by imaging confirmation in the absence of pathology, or by assigned clinical stage of I-IV by the treating physician, or by other clinical confirmation of disease per treating clinician (e.g. biochemical evidence of recurrence of prior cancer).

Secondary Analysis Objectives and Endpoints

There are nine secondary study objectives:

1) Assess participant-reported anxiety resulting from use of the MCED test.

   Endpoints:

   - Anxiety assessed by State Trait Anxiety Inventory (STAI)[12]+ MCED STAI questionnaires pre-test (baseline), post-test, at diagnostic resolution (if applicable) and at 1 year (+30D).

2) Evaluate intention and utilization of guideline recommended cancer screening procedures after use of the MCED test.

Endpoints:

- Intention to follow standard of care cancer screening tests assessed pre-test, post-test and at 1 year (+30D).
- Utilization of standard of care cancer screening tests prior to study enrollment, during the first year and the second year (+/-30D) after MCED test.

3) Describe the ability of confirmatory PET-CT to detect cancer in participants where diagnostic resolution of cancer diagnosis is not achieved through the targeted diagnostic testing triggered by MCED test results.

   Endpoint:

   - Diagnosis of cancer after confirmatory PET-CT

4) Characterize the number and type of diagnostic evaluations by predicted cancer signal origin and outcome of diagnostic resolution.

   Endpoints:

   - Number and type of imaging procedures
   - Number and type of invasive procedures
   - Number and type of laboratory tests
   - Time to diagnostic resolution

5) Evaluate the radiation exposure during diagnostic evaluation.

   Endpoint:

   - Per participant radiation exposure during diagnostic evaluation

6) Evaluate concordance of initial GRAIL test result and CSO prediction, with results from the research blood draw.

Endpoints:

- MCED test results for cancer detection and CSO prediction from the initial and research blood draws in the subset of participants with research blood draw.

7) Assess participants reported outcomes and perceptions about the MCED test.

   Endpoints:

   - Health-related quality of life following the MCED test assessed by SF-12v2
   - Adapted MICRA
   - Satisfaction with the MCED test
   - Attitudes towards subsequent MCED screening

8) Evaluate performance of the MCED test in selected demographic and clinical subgroups

   Endpoints:

   - Test performance for cancer detection as described in Section 10.4.2, assessed in selected demographic and clinical subgroups classified on the basis of different categories of age, sex, race, ethnicity, BMI, smoking or cancer history.

9) Evaluate MCED test positive rate in subgroups with potential cross-reactive conditions

   Endpoints:

   - Proportion of participants with MCED positive test results out of all participants with analyzable MCED test results, assessed in participants with certain non-malignant hematological conditions, participants who took cytotoxic medications in the past year, participants who took demethylating agents in the past year, participants with a prior cancer history, and other conditions that might affect cfDNA shedding or methylation.

Analysis methods and measures for the secondary study objectives are described in Section 10.4.3.

Exploratory Analysis Objectives and Endpoints

There are three exploratory study objectives:

1) Evaluate performance of the MCED test over three years of follow up.

   Endpoint:

   - Diagnosis of cancer over three years of follow up.

2) Evaluate changes in blood based biomarkers among the subset of participants with a research blood draw.

Endpoints:

- Blood biomarker signals from the initial and research blood draws in the subset of participants with a research blood draw.

3) Evaluate different CSO reporting classes based on clinically relevant subgroups.

Endpoints:

- MCED test results for CSO prediction and blood biomarker signals from the initial and research blood draws evaluated in the context of diagnostic testing triggered by the test results.

5. Study Design

5.1. Overall Design

This is a prospective, multi-center interventional study of the GRAIL MCED Test with return of results in healthcare systems in North America. The purpose is to evaluate the safety and efficacy of the GRAIL MCED Test in an eligible screening population.

The study will enroll at least 35,000 and no more than 38,500 participants as defined by eligibility criteria over an anticipated enrollment period of approximately 36 months at up to 40 clinical institutions within North America. Each enrolling institution will employ its own recruitment strategies to identify potential participants, which may include identification of participants through electronic health records, scripted phone calls, emails, recruitment campaigns, and other outreach strategies. Recruitment strategies at each enrolling institution may require Institutional Review Board (IRB) or Research Ethics Boards (REB) approval, as applicable.

Following informed consent, the participant will undergo the GRAIL MCED Test. Clinical information, including demographic and medical data relevant to cancer risk, will be collected from all participants via medical record, participant questionnaires, and the GRAIL Multi-cancer Test Requisition Form (TRF). The test will be ordered by a study investigator at an enrolling site.

Following blood collection, the study test will be performed at the GRAIL clinical laboratory, which has a Certificate of Accreditation under the Clinical Laboratory Improvement Amendments (CLIA) to perform high complexity testing. The Test Result Report will be returned to the study investigator and site study team via the test result portal and any ensuing diagnostic work-up will be captured (see Section 9.4).

GRAIL MCED Test Negative

Participants will be notified of “cancer signal not detected” test results via phone call, virtual visit, or an in-person clinic visit with the study investigator or designated member of the study team (e.g. research nurse), and the study team must remind participants not to interpret “cancer signal not detected” test results as the absence of cancer and to continue to adhere to guideline-recommended cancer screening.

GRAIL MCED Test Positive

Participants will be notified of "cancer signal detected" test results via phone call, virtual visit, or by in-person clinic visit with the study investigator or designated member of the study team (e.g. research nurse). As appropriate, during the communication of results, participants will be invited to follow-up with their personal healthcare provider (primary healthcare provider or specialist) or to follow-up with a study investigator. If the participant elects to follow-up with his/her personal healthcare provider, the study team will notify the personal healthcare provider of the "cancer signal detected" test results and confirm the healthcare provider's willingness to work-up the result in consultation with the study team. If the participant does not elect to follow-up with his/her personal healthcare provider or the personal healthcare provider declines to work-up the participant, a study investigator will be available to work-up the result. The personal healthcare provider may consult, as needed, with relevant specialists or review boards at their institution, to discuss the diagnostic work-up of a "cancer signal detected" test result.

It is intended that participants will receive targeted diagnostic tests, procedures or evaluations based on the test returned cancer signal origin(s) (CSO). For cancer signal detected test results, up to two CSOs may be reported. It is intended for the healthcare provider to first evaluate the participant for the first CSO. If no cancer is identified, the healthcare provider should evaluate the participant for the second CSO reported. The healthcare providers are strongly encouraged to attempt to rule out cancer in the organ or anatomical location described by the predicted CSO, where applicable, using the diagnostic evaluations corresponding to the CSOs listed in Table 4. In some cases, based on the predicted CSOs, it may be appropriate to utilize work-ups that evaluate both the first and second predicted CSOs simultaneously. If cancer is not identified by these CSO-directed evaluations, the healthcare provider should perform a PET-CT (see Figure 2). If a participant is unable to undergo the prescribed procedure or the institution physician determines an alternative or additional work-up is necessary, the healthcare provider will document the reason for deviating from the recommended procedure(s) and list the alternative and additional procedure(s) performed.

Figure 2. Diagnostic Evaluations- Scenario of Positive Result Qualifying for Two Cancer Signal Origins (CSO)

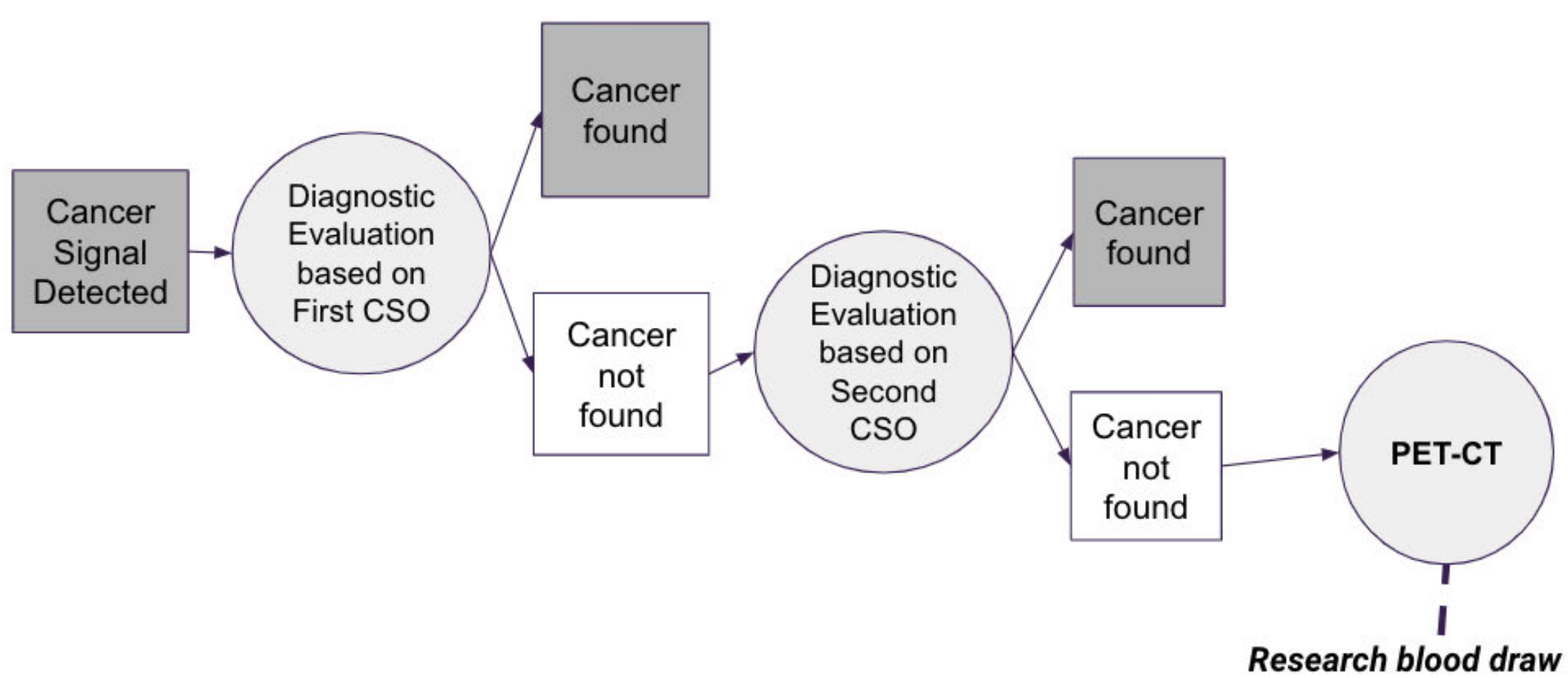

Table 4. Cancer Signal Origin Directed Diagnostic Evaluations

Cancer Signal Origin	Predicted Cancers	Diagnostic Evaluation
Anus	Anus	Anoscopy or sigmoidoscopy
Bladder, Urothelial Tract	Bladder, Renal Pelvis, Ureter, Urethra	Flexible cystoscopy and CT urogram or retrograde pyelogram with contrast
Bone and Soft Tissue	Skeletal Muscle and Other Connective Tissue, Vascular Tissue, Bone and Cartilage	MRI with contrast or CT of chest, abdomen, pelvis or PET-CT
Breast	Breast	Diagnostic mammography<br><br>MRI if participant has recently had screening mammogram
Cervix	Cervix	Cytology, HPV testing, colposcopy
Colon, Rectum	Colon, Rectum, Appendix	Colonoscopy
Head and Neck	Oropharynx, Hypopharynx, Nasopharynx, Larynx, Lip and Oral Cavity (including Oral Tongue), Nasal Cavity, Paranasal Sinuses, Major Salivary Glands	Flexible nasal endoscopy and Head and Neck CT or MRI
Kidney	Kidney	Triple phase renal CT
Liver, Bile duct	Liver, Intrahepatic Bile Duct	Triple phase abdominal CT, AFP
Lung	Lung, Bronchus	Chest CT
Lymphoid Lineage	Lymphoid leukemia<br>Lymphoma	Complete blood count (CBC) with differential, peripheral blood smear, LDH, Hepatitis B and C serology, comprehensive metabolic panel (CMP)<br><br>Bone marrow biopsy after blood tests if needed<br><br>CT of chest, abdomen, pelvis with contrast or PET-CT if needed
Melanocytic Lineage	Melanoma	Full body skin exam
Myeloid Lineage	Myeloid neoplasm	CBC with differential, peripheral blood smear, LDH, uric acid, CMP

Cancer Signal Origin	Predicted Cancers	Diagnostic Evaluation
		Bone marrow biopsy after blood tests if needed
Neuroendocrine Cells of Lung or other Organs	Neoplasms arising from cells of neuroendocrine lineage in the lung and high-grade cancer arising from neuroendocrine lineage in all body regions	CT of chest with or without contrast, CT of abdomen, pelvis, head & neck
Ovary	Ovary, Fallopian Tube, Primary Peritoneum	Transvaginal ultrasound and CA-125
Pancreas, Gallbladder	Pancreas, Extrahepatic Bile Duct, Gallbladder	Pancreas CT protocol or MRCP
Plasma Cell Lineage	Plasma cell neoplasm	CBC with differential, peripheral blood smear, LDH,uric acid, beta-2-microglobulin, creatinine clearance, serum immunoglobulins, serum free light chain assay, serum and urine protein electrophoresis, CMP<br><br>Bone marrow biopsy after blood and urine tests if needed<br><br>Whole-body low-dose CT or PET-CT if needed
Prostate	Prostate	PSA and multi-parametric MRI of the prostate
Stomach, Esophagus (upper GI)	Stomach, Esophagus	Upper endoscopy
Thyroid Gland	Thyroid Gland	Ultrasound of thyroid
Uterus	Uterus	Transvaginal ultrasound

Additional evaluations recommended for all CSOs: complete blood count (CBC) with differential test and comprehensive metabolic panel (CMP; includes albumin, blood urea nitrogen, calcium, carbon dioxide, chloride, creatinine, glucose, potassium, sodium, total bilirubin, total protein, alanine aminotransferase, alkaline phosphatase, aspartate aminotransferase).

- Diagnostic resolution resulting in a diagnosis of cancer will be documented based on pathologic confirmation of an invasive solid tumor or hematologic malignancy, or by imaging confirmation in the absence of pathology, or by the assignment of clinical stage I-IV by the treating clinician, or by other

clinical confirmation of disease per treating clinician (e.g. biochemical evidence of recurrence of prior cancer).

- For participants where diagnostic resolution of cancer diagnosis is achieved through the targeted work-up based on the predicted CSO(s), all available pathology, imaging and clinical staging information will be captured.
- For participants with a cancer signal detected where diagnostic resolution of cancer diagnosis is not achieved through the targeted diagnostic procedures recommended for the predicted CSO(s), and where PET-CT was not already performed as part of the work-up, a confirmatory PET-CT will be performed to assess cancer status.
    - For participants where diagnostic resolution of cancer is achieved through a confirmatory PET-CT, all available pathologic, imaging and clinical staging information will be captured.
    - Participants in this group will also undergo a research blood draw, which may be completed on the same day as and before undergoing PET-CT or within 14 days after the PET-CT. Results of this research blood draw will not be provided back to the study investigator or participant.
- The result of any performed diagnostic procedure(s) will be recorded until the date of “diagnostic resolution.” Diagnostic resolution is defined as the end of diagnostic evaluations used to determine whether or not a cancer is present. This is intended to include all of the tests and procedures ordered to evaluate the predicted CSO(s) up to and including confirmatory PET-CT, if performed, and tests ordered directly as a result of findings reported from the confirmatory PET-CT.
- The date of diagnostic resolution is defined in section 9.5.
- Participant questionnaires will be deployed prior to the Day 1 blood draw, following receipt of study test results, following diagnostic resolution for “cancer signal detected” test results, and at 1 year (+30D) to assess parameters related to participant perceptions about the multi-cancer early detection test, including anxiety, health-related quality of life, and potential impact of test results on attitude towards adherence to guideline-recommended screening.

Cancer status will be assessed for all participants on an annual basis. For participants diagnosed with a cancer at any time during the 3 year follow-up period, all available pathologic, imaging and clinical staging information will be captured.

5.2. Estimated Study Duration

The expected study duration is approximately 6.75 years.

5.3. Study Duration for Study Participants

The expected duration for each study participant will be 3 years from the Day 1 blood draw.

5.4. Study Design Schema

Please refer to Section 2.2., Schema, above.

5.5. Scientific Rationale for Study Design

This prospective, longitudinal, multicenter clinical study design is required to provide data to comprehensively evaluate the safety of the GRAIL MCED test. Additionally and in combination with other studies, this design will provide data to evaluate the efficacy of the GRAIL MCED test.

5.6. Study Enrollment Definition

Study enrollment commences when the first study participant meets all inclusion and no exclusion criteria and provides informed consent.

5.7. End-of-Study Definition

All participants will be actively followed by the enrolling institution for 3 years following their blood draw date.

The end of the study for all participants is defined as the completion of the last study assessments for the last participant, which may occur 3 years after the last participant's Blood Draw date or sooner, if the Sponsor ends the study early (See Section 9.9).

6. Study Population

The study will enroll at least 35,000 and no more than 38,500 participants. The study should ensure a diverse participant population is enrolled, generally representative of the US population for persons aged 50 years or older with respect to race, ethnicity and sex. Targets for distribution by race and ethnicity are listed in Table 5 and are based on the race and ethnicity composition of Americans who are aged 50 years or older as reported in the 2019 American Community Survey per the United States Census Bureau[28]. Individual race/ethnicity strata may be closed to new enrollments during the course of the study to achieve the target distribution.

Table 5. Targets for Distribution by Race and Ethnicity

Race/ Ethnicity	**Target for Enrolled Study Population** (Note: final study enrollment percentages may not add to 100% since categories are not mutually exclusive)
White alone (non-Hispanic)[a]	72%
Hispanic or Latino[b]	11%
African American or Black[c]	11%
Asian, Native Hawaiian or Other Pacific Islander[c]	6%
American Indian/Alaska Native[c]	1%

(a) Includes persons reporting only one race and not of Hispanic ethnicity

(b) Includes persons of Hispanic ethnicity regardless of race
(c) Includes persons who report this race regardless of ethnicity; may include persons who report more than one race

The study will target the age and sex distribution listed in Table 6 which is enriched for individuals in the age categories 60-69 years and 70-79 years in comparison to the U.S. population based on 2020 Census data[29]. Enrichment for these age categories will serve as the primary method to enrich the cohort for cancer events and enrollment will be monitored to ensure these targets are achieved. Individual age and sex strata may be closed to new enrollments during the course of the study to achieve the target distribution.

Table 6. Targets for Age and Sex Distribution

	Age 50-59 years	Age 60-69 years	Age 70-79 years	Age 80+ years	Total
Female	12.5%	23.75%	12.5%	1.25%	50%
Male	12.5%	23.75%	12.5%	1.25%	50%
Total	25%	47.5%	25%	2.5%	100%

Prospective approval of clinical study protocol deviations (PDs) to recruitment and enrollment criteria, also known as clinical study protocol waivers (protocol waivers) or clinical study protocol exemptions (protocol exemptions), is not permitted.

6.1. Inclusion Criteria

Participants are eligible to be included in the study only if all of the following criteria apply.

1. Participants must be at least 50 years of age, inclusive, at the time of signing the Informed Consent Form (ICF).
2. Informed Consent
    - Capable of giving signed and legally effective informed consent as described in Section 5.4, Study Design Schema, which includes compliance with the requirements and restrictions listed in the Informed Consent Form (ICF) and in this protocol. Consent provided by a legally authorized representative is not permitted in this protocol.

6.2. Exclusion Criteria

1. Undergoing or referred for diagnostic evaluation due to clinical suspicion for cancer (e.g., referred to a medical or surgical oncologist, or scheduled for biopsy on the basis of a suspicious imaging abnormality).

2. Personal history of invasive solid tumor or hematologic malignancy, diagnosed within the 3 years prior to expected enrollment date, or diagnosed greater than 3 years prior to expected enrollment date and never treated.
   - Individuals with a diagnosis of non-metastatic basal cell carcinoma and squamous cell carcinoma of the skin are not excluded.
3. Prior/Concurrent Concomitant Therapy (Medications/Treatments):
   - Definitive treatment for invasive solid tumor or hematologic malignancy within the 3 years prior to expected enrollment date. Adjuvant hormone therapy for cancer (e.g. for breast or prostate cancer) is not an exclusion criterion.
4. Individuals who will not be able to comply with the protocol procedures.
5. Individuals who are not currently registered patients at a participating center.
6. Previous or current participation in another GRAIL-sponsored study. “Participation” is defined as having signed consent and provided a blood sample.
7. Previous or current employees or contractors of GRAIL.
8. Current pregnancy (by self-report of pregnancy status)

6.3. Participant Recruitment

Each enrolling institution will employ recruitment strategies to identify potential participants, which may include identification of participants through electronic health records, scripted phone calls, emails, recruitment campaigns, and other outreach strategies.

6.4. Informed Consent Process

Only participants who meet all inclusion and exclusion criteria will sign the informed consent. Once a participant is confirmed to meet all eligibility criteria, they will then sign informed consent. Participants who do not meet all eligibility criteria should not sign the informed consent.

- The study investigator or designated member of the study team will explain the nature of the study to the prospective participant and answer all questions regarding the study.
- Prospective participants must be informed that their participation is voluntary. Participants will be required to sign a statement of informed consent that meets the requirements of Title 21, Code of Federal Regulations, Part 50 - Protection of Human Subjects (21 CFR 50), local regulations, ICH guidelines, Health Insurance Portability and Accountability Act (HIPAA) requirements (if the HIPAA authorization is combined with the consent form in one document), and any stipulations or conditions imposed by the Institutional Review Board (IRB)/Independent Ethics Committee (IEC)/Research Ethics Board (REB) or study center.

- A legally authorized representative is not allowed for the purposes of this study. Participants must understand the protocol requirements and be able to give legally effective informed consent.
- The medical record must include a statement that written informed consent was obtained before the participant was enrolled in the study, and the date the written consent was obtained. The authorized person obtaining the informed consent must also sign the ICF.
- Participants must be re-consented to the most current version of the ICF(s) during their participation in the study, if required by the IRB/REB.
- A copy of the ICF(s) must be provided to the participant.

Informed consent should occur within 30 days prior to the Day 1 blood draw and must be obtained prior to any study procedures being performed. Participants who are unable to perform the Day 1 blood draw within 30 days of consent may delay the Day 1 blood draw up to 60 days from informed consent. However, in these instances, all eligibility criteria must be re-confirmed prior to drawing blood. If blood is not drawn within 60 days following consent, the participant will be withdrawn from the study.

The ICF may be offered in several IRB/IEC/REB-approved formats so that participants can review or complete it in advance. These formats include remote e-consent (desktop/laptop, mobile tablet, or smartphone), in-clinic consent (tablet or paper), mailed consent (paper), and PDF (for pre-review only). Availability of each format to a given participant will depend on the clinical site's policies.

The ICF will contain a separate section that addresses the use of remaining samples for exploratory research. The study investigator or designated member of the study team will explain to each participant the objectives of the exploratory research. Participants will be told that they are free to refuse to participate and may withdraw their consent at any time and for any reason during the storage period.

No study procedures will be performed prior to obtaining informed consent. Informed Consent will be obtained per federal regulations and applicable ICH /Good Clinical Practice (GCP) guidelines.

Copies of all signed informed consent forms will be stored at the study site. Signed consent forms will be available for verification by GRAIL or its designee at any time.

7. Measures to Minimize Bias

In this prospective longitudinal study, information related to demographics, clinical characteristics, diagnostic procedure types, counts, and dates will be captured from the electronic medical records (EMR), which will minimize most biases (e.g., recall bias, observer bias, missing data bias).

An important source of bias in prospective studies is selection bias due to differences in response rates among participant subgroups, or with respect to study endpoints. Selection bias or poor representativeness could result in study results not being sufficiently generalizable to larger screening populations. To assess bias in enrollment, the Sponsor will monitor and continuously compare distributions of key demographic and clinical characteristics of the enrolled population to the target population. In particular, if there is disproportionately high enrollment of a particular stratum (based on

age and sex), further enrollment of individuals in this stratum may be limited. This will be determined by the Sponsor.

7.1. Blinding Methods

This study is not blinded.

7.2. Participant Follow-Up

7.2.1. Active Clinical Follow-Up

Participants with a "cancer signal detected" test result will require additional evaluation for cancer and any follow-up per the discretion of the study investigator or personal healthcare provider (see Sections 9.5).

All participants will be followed for 3 years after the date of blood draw, and these study procedures will be conducted (see SoA):

- 1 year (+30D) questionnaire
- 1 year, 2 year, 3 year (+/-30D) cancer assessment
  - Follow-up clinical information for cancer status on all participants will be performed at 1 year, 2 year, 3 year (+/-30D) after enrollment from a search of medical records by the study team, which may be supplemented by direct contact with participants.
  - Collected clinical data will include but is not limited to: cancer diagnosis, cancer type, date of cancer diagnosis, method of detection, cancer treatments, cancer screening as well as any pathology and imaging reports underlying the clinical diagnosis (see Section 9.6).
- 1 year, 2 year, 3 year (+30D) assessment of guideline-recommended cancer screening utilization

7.3. Participant Retention Through Follow-Up

The GRAIL-maintained study website and regular email communications will be the primary venue for providing updates and maintaining engagement of the participants throughout the study follow-up period. However, in the informed consent form, participants will be able to indicate their preferred method of communication, including but not limited to email, telephone, text/short message service (SMS), etc. The study sites will be responsible for sending communications to the participants.

The study participant will be considered active in the study per study investigator and/or Sponsor decision.

Upon successful contact, the study team member should confirm the eligibility of the study participant prior to any study assessments or procedures.

During the study period from return of results until EOS, the study site should make every attempt to contact the participant based on the participant's preferred method of communication. IRB/IEC/REB

approval of such communications to the participant for follow-up will be obtained as required by study sites. The study participant may be contacted in order to meet the study assessments determined by this protocol and site specific ICF.

See Section 8.2 - Lost to Follow-Up for lost to follow-up determination.

8. Participant Withdrawal

8.1. Participant Withdrawal from the Study

- A participant may withdraw from the study at any time at their own request or may be withdrawn at any time at the discretion of the PI, GRAIL, overseeing IRB/IEC/REB, or regulatory authority, for safety, behavioral, compliance, or administrative reasons.
- If the participant withdraws consent for disclosure of future information, GRAIL may retain and continue to use any data collected before such a withdrawal of consent.
- If a participant withdraws from the study, they may request destruction of any samples taken and not tested, and the PI must document this in the study site records.
- Cancer status assessment will be collected at the time of study participant withdrawal.
- Once withdrawn, participants are not eligible to re-enroll in the study.

A participant may be withdrawn from the study for the following reasons:

- **Failure to meet inclusion/exclusion criteria**:
  - If a participant has consented in the study but retrospectively found to not meet all of the inclusion/exclusion criteria, the participant will be withdrawn from the study.
- **Failure to perform Day 1 blood draw, or, if required, Repeat Blood Draw within 60 days following consent**
- **Participant withdrawal of consent:**
  - Participation in this study is voluntary. Participants may decide not to participate or may leave the study at any time. This decision will not result in any penalty or loss of benefits to which participants are entitled. Information and biospecimens that have already been collected may still be used, but no new information will be collected.
  - If a participant's biospecimen has not been processed at the time the participant withdraws from the study, the biospecimen may be destroyed. If the biospecimen has already been tested, the data generated from the testing will not be destroyed.
- **Failure to return test results**
  - Participants can be withdrawn if they cannot be reached to receive their test results within 90 days from the test result date at the discretion of the PI.
- **Other withdrawal circumstances:**

- If the reason for participant withdrawal does not fall under any of the reasons outlined above, then the withdrawal reason and the withdrawal date must be documented. Discussion with the Sponsor is encouraged.

8.2. Lost to Follow-Up

A participant will be considered lost to follow-up if he or she is repeatedly unable to be contacted by the study site at the end of the study.

The following actions must be taken if a participant fails to complete a required study procedure or assessment:

- The study site must attempt to contact the participant and inform the participant of the importance of protocol compliance and ascertain whether or not the participant wishes to and/or should continue in the study.
- The study investigator or designated member of the study team must make three (3) contact attempts to reach the participant (e.g. telephone calls or emails sent to participant) at the time of each annual cancer status assessment. These contact attempts should be documented in the participant's study record. Should the participant continue to be unreachable at the 3 year time point, he/she will be considered withdrawn from the study at that time, due to being lost to follow-up. The study investigator or designated member of the study team will then document the date of withdrawal in EDC, and no further data will be collected.

9. Study Assessments & Procedures

- Study procedures and their timing are summarized in the SoA (refer to Section 2.3., Schedule of Activities [SoA]). Protocol waivers or exemptions are not allowed.
- Adherence to the study design requirements, including those specified in the SoA, is essential and required for study conduct.
- Tests, evaluations and procedures conducted as part of the participant's routine clinical management (e.g. scheduled mammogram) and obtained before signing of the ICF may be utilized for baseline purposes provided they met the protocol-specified criteria.

Study staff at each site will be trained on all study procedures prior to the activation of each site. Clinical data collection for the study is performed by site clinical research staff and by participants via electronic methods. Subsequent study assessments beyond the blood draw visit will be based on test results.

9.1. Enrollment

Enrollment into this study requires confirmation of eligibility and informed consent.

Individuals may only enroll into this study once.

The Sponsor will monitor and regularly evaluate distributions of key demographic and clinical characteristics of both the enrolled and target cohorts. For example, if there is disproportionately high

enrollment of a particular group, further enrollment of individuals in this group may be limited. This will be determined solely by the Sponsor.

9.2. Participant Questionnaire

Questionnaires will be delivered to participants through mail or electronic mail. Electronic participant-reported outcomes (ePRO) can be accessed on tablets, computers, or handheld devices. See Table 3 for additional details regarding participant questionnaires.

9.3. Blood Draw

There are three potential time points of blood draws for this study:

1. Blood Draw
    a. First blood draw required for enrollment into the study.
    b. Results will be returned to the study investigator and communicated to the participant.
2. Repeat Blood Draw
    a. Occurs in the event of technical issues or processing failures at the request of GRAIL.
3. Research Blood Draw (see Section 5.1)
    a. For “cancer signal detected” cases only where no cancer diagnosis is obtained after targeted diagnostic evaluation based on predicted CSO.
    b. Research blood draw samples will be used for future research and results will not be returned to the study investigator or participant.

At each blood collection time point (described above), peripheral blood will be obtained by routine venipuncture. Blood will be collected in four blood collection tubes and each blood collection tube should be filled completely (approximately 10 mL of blood in each tube), with a minimum of 3 tubes of blood with at least 3 ml in each tube. Two tubes of blood will be used to run the investigational test. Participants will be asked to have repeat blood draw (four tubes) if the minimum amount of blood is not obtained, or if technical issues with the samples preclude sample processing or reporting. Any remaining blood samples will be stored without readily identifiable information and may be used for process improvement activities, proficiency testing tasks, or future research activities relevant to the investigational test.

Study blood collection must be completed in a single session on one day, and blood tubes should be collected from a single venipuncture session following the methods described in the study-specific lab manual. Blood collection and shipping should be performed according to the laboratory manual. Blood collection tubes will be labeled at the time of blood draw and include protected health information with three unique identifiers (e.g. GRAIL ID, Participant ID, date of birth or age). Protected health information will also be collected on the TRF that is used for the blood collection process.

The expected amount of blood collected from participants is approximately 40 mL. The maximum amount of blood collected from participants is approximately 120 mL (40 mL at first blood draw, 40 mL at repeat blood draw if processing issues, 40 mL blood draw for research for "Cancer Signal Detected" participants if cancer diagnostic resolution is not achieved).

Blood and plasma samples remaining after the testing is completed will be stored and accessed by GRAIL for further research relevant to the blood test, laboratory process improvements, and for proficiency testing, unless otherwise required by applicable site-specific ICF or per applicable requirements. GRAIL will store the blood samples in a secure storage space with adequate measures to protect confidentiality. Blood samples obtained from participants who were retrospectively determined to have not met the eligibility criteria will be destroyed after the clinical data collection period for enrolled participants has concluded.

The Streck blood collection tubes used in this study are authorized for the collection, stabilization, and transport of venous whole blood samples for use in conjunction with cell-free DNA next-generation sequencing liquid biopsy assays. In addition, GRAIL has conducted extensive analytical and clinical validation on specimens collected in Streck blood collection tubes, including specimen stability studies, to ensure compatibility of Streck blood collection tubes with the GRAIL product. The blood collection tubes have not been cleared or approved for use with our assay by the FDA.

9.4. Return of Results

The Test Result Report (TRR) is a report with the result of the study test, which will specify "cancer signal detected" or "cancer signal not detected" test results. In some cases where a final result cannot be determined, a Cancellation Report will be issued and a repeat blood draw will be requested as described in section 9.3. The TRR will be returned to the study investigator via paper or electronic methods (test results portal) typically within 15 calendar days from the date of blood collection, barring holidays and unforeseen circumstances affecting sample shipment and lab processing. Only the study investigator or designated member of the study team will have access to the test result portal, which is encrypted and requires a unique access code for each designated user. The study investigator or designated member of the study team will review all study test results prior to communicating results to the participant.

The study investigator or designated member of the study team (e.g., research nurse) will notify participants of "cancer signal not detected" test results via phone call, virtual visit, or by in-person clinic visit with the study investigator or designated member of the study team. The participant will be reminded not to interpret "cancer signal not detected" test results as the absence of cancer and to continue to adhere to guideline-recommended cancer screening.

Participants will be notified of "cancer signal detected" test results via phone call, virtual visit, or by in-person clinic visit with the study investigator or designated member of the study team (e.g. research nurse).

The study test result should be communicated to the participant as soon as possible, but not later than 30 days from the date of the blood draw. The date on which the test result is communicated to the participant must be documented in EDC.

9.5. Diagnostic Work-Up of a "Cancer Signal Detected" Test Result

It is intended that participants will receive targeted diagnostic tests, procedures or evaluations based on the test returned cancer signal origin(s) (CSO). For cancer signal detected test results, up to two CSOs may be reported. It is intended for the healthcare provider to first evaluate the participant for the first CSO. If no cancer is identified, the healthcare provider should evaluate the participant for the second CSO reported. Healthcare providers are strongly encouraged to attempt to rule out cancer in the organ or anatomical location described by the predicted CSO, where applicable, using the diagnostic evaluations corresponding to the CSOs listed in Table 4. In some cases, based on the predicted CSOs, it may be appropriate to utilize work-ups that evaluate both the first and second predicted CSOs simultaneously. If cancer is not identified by these CSO-directed evaluations, the healthcare provider should perform a full body (skull vertex to mid-thighs) diagnostic positron emission tomography-computed tomography (PET-CT) scan with contrast. PET-CT should be performed according to practice parameters provided by the American College of Radiology (see Figure 2).[13] If a participant is unable to undergo the prescribed procedure or the healthcare provider determines an alternative or additional work-up is necessary, the healthcare provider will document the reason for deviating from the recommended procedure(s) and list the alternative and additional procedure(s) performed.

- Diagnostic resolution resulting in a diagnosis of cancer will be documented based on pathologic confirmation of an invasive solid tumor or hematologic malignancy, or by imaging confirmation in the absence of pathology, or by the assignment of clinical stage I-IV by the treating clinician, or by other clinical confirmation of disease per treating clinician (e.g. biochemical evidence of recurrence of prior cancer).
- For participants with a cancer signal detected where diagnostic resolution of cancer diagnosis is not achieved through the targeted diagnostic procedures recommended for the predicted CSO(s), and where PET-CT was not already performed as part of the work-up, a confirmatory PET-CT should be performed to assess cancer status.
    - Participants in this group will also undergo a research blood draw, which may be completed on the same day as and before undergoing PET-CT or within 14 days after the PET-CT (Figure 1. Study Design Schema).
    - Any test results from this research blood draw will not be provided back to the study investigator or participant.
- The result of any performed diagnostic procedure(s) will be recorded until the date of "diagnostic resolution." Diagnostic resolution is defined as the end of diagnostic evaluations used to determine whether or not a cancer is present. This is intended to include all of the tests and procedures ordered to evaluate the predicted CSO(s) up to and including confirmatory PET-CT, if performed, and tests ordered directly as a result of abnormal findings reported from the confirmatory PET-CT.
- The date of diagnostic resolution is defined as follows:
    - For cases in which cancer is diagnosed (resolution of " Cancer Diagnosis"):

        - If cancer is diagnosed based on pathologic confirmation: the date of diagnostic resolution is the specimen collection date for the blood or tissue sample that confirmed the cancer diagnosis.
        - If cancer is diagnosed without pathologic confirmation: the date of diagnostic resolution is the date of the imaging test or clinical investigation that confirmed the cancer diagnosis.
    - For cases in which no cancer is diagnosed (resolution of "No Cancer Diagnosis"):
        - If targeted diagnostic evaluation does not lead to a cancer diagnosis and the confirmatory PET-CT is negative, the date of diagnostic resolution is the date of the confirmatory PET-CT.
        - If the confirmatory PET-CT indicates abnormal findings that warrant additional investigation, the date of diagnostic resolution is the date of the last test to evaluate these abnormal findings ordered directly after PET-CT.

9.6. Clinical Data Collection

Clinical data will be collected from the blood collection tube, Test Requisition Form (TRF), Test Result Report (TRR), the participant's medical record, and participant questionnaires, and will either be entered into and stored in GRAIL's Laboratory Information Management System (LIMS) or into electronic case report forms (eCRFs) in the EDC system.

Clinical data to be captured during the course of the study will include:

- Protected Health Information, including participant date of birth (DOB) or age, will be collected and stored by GRAIL during the multi-cancer early detection testing process.
    - Protected health information will be collected and entered into GRAIL's LIMS and kept encrypted and access restricted to trained GRAIL laboratory personnel.
    - The participant study identification number (participant ID) will be used to link test results to the data captured in EDC. The site coordinators may enter participant email addresses provided by participants into the EDC system, only for the purposes of the questionnaire. The email addresses will be stored in the EDC system in such a way that the Sponsor Study Team does not have view or edit access. The analysis datasets exported from EDC and clinical study reports will not contain participant names, dates of birth or email addresses. Data used for analysis will include limited identifiable healthcare information required for research purposes, such as location of clinical site, age at enrollment, dates of study assessments, diagnostic work-up details, dates of cancer diagnosis, and date of death as applicable.
- Demographics, such as, but not limited to, body mass index (BMI), biological sex, race/ethnicity, and age.

- Past medical history including personal and family cancer history, cancer risk factors and cancer screening history, solid organ transplant history, bone marrow transplant history, and current health status, including pregnancy status and recent use of specific medications (such as cytotoxic or demethylating agents).
- Test results, reports, and treatments including but not limited to laboratory tests, imaging procedures, endoscopic procedures, surgical procedures, other cancer screening and diagnostic tests and cancer treatments.
- Cancer assessment - reporting of any cancer diagnosis, type, date of cancer diagnosis, clinical stage and method of detection.
- For participants diagnosed with cancer, all available pathologic, imaging and clinical staging information should be captured.

For individuals with a "cancer signal detected" test result, clinical data collection may also include:

- Documentation of criteria used by the healthcare providers to determine the diagnostic work-up selection.
- Results from diagnostic work-up following a multi-cancer early detection test result, such as imaging, endoscopy, laboratory tests (e.g. hematology, blood chemistry), and other types of diagnostic procedures.
- Clinical diagnostic resolution information (if cancer is found) during diagnostic work-up.
- Clinical findings, including incidental findings identified during the course of work-up and evaluations (e.g. lung nodules found on imaging, hematologic conditions identified during testing, adenomas detected during endoscopy).
- Current use of cytotoxic or demethylating agents.

For all individuals diagnosed with cancer as a result of working up a "cancer signal detected" test result, or as a result of routine clinical care following a negative MCED test result, clinical data collection may also include:

- All pathology reports and supporting diagnostic reports for biopsies and surgical resections relevant to cancer evaluation and diagnosis (e.g. anatomic pathology reports, molecular reports, IHC reports, flow cytometry reports, hemoglobin electrophoresis reports), clinical and/or pathologic stage.
- Results of any molecular testing performed on tumor tissue or other specimens (e.g. urine, blood, cerebrospinal fluid) as part of standard of care.
- Signs and symptoms at the time of diagnosis.

9.7. Safety Assessments

9.7.1. General

Study participants may experience adverse events (AEs) associated with the study procedures or the investigational test. AEs associated with the investigational test are generally considered adverse device effects (ADEs), which could include unanticipated adverse device effects (UADEs). Study-related adverse events (AEs, serious adverse events [SAEs], ADEs, and UADEs), will be collected, documented, analyzed, and reported as applicable. “Study-related” is defined as related to a study activity listed in the protocol SoA (refer to Section 2.3, Schedule of Activities [SOA]), and includes complications from procedures performed as part of a diagnostic evaluation following a “cancer signal detected” test result.

The safety of the study will be overseen by the study investigator, study Sponsor, reviewing IRBs, REBs, FDA, and an Independent Data Monitoring Committee (IDMC).

9.7.2. Adverse Events (AEs) and Serious Adverse Events (SAEs)

The definition of an AE is any untoward medical occurrence in a participant or study participant, <u>temporally associated with the use of a study test</u>, whether or not considered related to the study test. An AE can therefore be any unfavorable and unintended sign (including an abnormal laboratory finding), symptom, or disease (new or exacerbated) temporally associated with the use of the study test.

AEs may be reported by the participant (or, when appropriate, by a caregiver, surrogate, or the participant's legally authorized representative).

The PI and any qualified designees are responsible for detecting, documenting, recording, and reporting events that meet the definition of an AE, and remain responsible for following up AEs that are serious, considered related to the study test or study procedures, or that caused the participant to discontinue the [study test/study].

An SAE is an AE that meets any of the following criteria:

- Led to death;
- Led to serious deterioration in the health of the participant, that resulted in:
  - A life-threatening illness or injury. The term ‘life-threatening' in the definition of serious refers to an event in which the participant was at risk of death at the time of the event. It does not refer to an event that could have caused death, if it were more severe;
  - Inpatient or prolonged hospitalization;
  - A permanent impairment of a body structure or a body function; or
  - Medical or surgical intervention to prevent life-threatening illness or injury or permanent impairment to a body structure or a body function; or

- Led to fetal distress, fetal death or a congenital abnormality or birth defect.
- Led to any other medically important serious event.

Planned hospitalization for a pre-existing condition without serious deterioration in health, is not considered an SAE.

The investigator or designated member of the study team should document the SAE into EDC (or complete a sponsor-provided SAE report if EDC is unavailable) within twenty-four (24) hours of notification/occurrence of the event, but in no event later than 10 working days after the investigator first learns of the effect. The study investigator will report the SAE to the IRB/IEC/REB according to the institution's IRB/IEC/REB reporting requirements.

After the initial SAE report, the study investigator is required to follow each participant at subsequent visits/contacts. All study-related AEs will be followed until resolution, stabilization, the event is otherwise explained, or the participant is lost to follow-up (as defined in Section 8.2, Lost to Follow-Up).

9.7.3. Adverse Device Effects (ADEs)

An ADE is an event related to the use of an investigational medical device. This definition includes AEs resulting from insufficient or inadequate instructions for use, deployment, implantation, installation, or operation, or any malfunction of the investigational medical device. This definition includes any event resulting from use error or from intentional misuse of the investigational medical device. AEs that are associated with the investigational test blood collection tubes or the test result report (i.e. return of results to the participant) should be considered and documented as an ADE. The PI and any qualified designees are responsible for detecting, documenting, recording, and reporting events that meet the definition of an ADE or UADE and remain responsible for following up on ADEs that are serious, considered related to the investigational test, or that caused the participant to discontinue the study (see Section 8.1, Participant Withdrawal from a Study).

The PI will monitor for the occurrence of ADEs per the standard of care for each participant undergoing a study-specified blood draw. Determination should be based on assessment of temporal relationships, biologic plausibility, association (or lack of association) with other procedures done as standard of care, and presence (or absence) of a more likely cause. The PI will report ADEs to the IRB/IEC/REB according to the institution's IRB/IEC/REB reporting requirements.

9.7.4. Unanticipated Adverse Device Effects (UADEs)

A UADE is any serious adverse effect on health or safety; any life-threatening problem or death caused by or associated with a device, if that effect, problem, or death was not previously identified in nature, severity, or degree of incidence in the investigational device exemption application (including supplementary plan or application) or any other unanticipated serious problem associated with a device that is related to the rights, safety, or welfare of participants.

Clinical study site personnel will complete a GRAIL-provided UADE report within 24 hours of notification/occurrence of the event. The PI will report the UADE to the IRB/IEC/REB according to the institution's IRB/IEC/REB reporting requirements.

The means of obtaining AE data should be described (volunteered, checklist, or questioning), as should any specific rating scales used and any specifically planned follow-up procedures for specific AE, or any planned rechallenge procedures in case study intervention is discontinued because of an AE.

9.7.5. Follow-Up of ADEs & UADEs

After the initial ADE/UADE report, the PI is required to proactively follow each participant at subsequent visits/contacts. All ADEs and UADEs will be followed until resolution, stabilization, the event is otherwise explained, or the participant is lost to follow-up (as defined in Section 8.2, Lost to Follow-Up).

9.7.6. Regulatory Reporting Requirements for UADEs

- Prompt notification by the PI to GRAIL of a UADE is essential so that legal obligations and ethical responsibilities towards the safety of participants and the safety of a study under clinical investigation are met.
- GRAIL has a legal responsibility to notify both the local regulatory authority and other regulatory agencies about the safety of a study under clinical investigation. GRAIL will comply with country-specific regulatory requirements relating to safety reporting to the regulatory authority, IRB/IEC/REB, and PIs.
- PI safety reports must be prepared for suspected UADEs according to local regulatory requirements and GRAIL policy and forwarded to PIs as necessary.
- A PI who receives a PI safety report describing a UADE or other specific safety information (e.g, summary or listing of UADEs) from GRAIL will review and then file it along with the PI's Brochure and will notify the IRB/IEC/REB, if appropriate according to local requirements.

9.7.7. Test Deficiencies and Concerns with the Test

A test deficiency is a perceived inadequacy of the investigational test with respect to its identity, quality, durability, reliability, safety, or performance, that does not result in an AE or ADE. Test deficiencies include but are not limited to malfunctions and inadequate labeling.

9.8. Study or Study Site Closure, Termination and Suspension

- GRAIL, the PI, or the IRB/IEC/REB reserves the right to close the study site or terminate the study at any time, for any reason at the sole discretion of GRAIL. Study sites will be closed upon study completion. A study site is considered closed when all required documents and study supplies have been collected and a study site closure visit has been performed. This can occur prior to study completion or upon study completion.
    - If GRAIL terminates or suspends the study, the PI must inform the IRB/IEC/REB and provide the IRB/IEC/REB a detailed written explanation of the termination or suspension.

    - If the PI terminates or suspends the study, PI must inform GRAIL, and the IRB/IEC/REB, and provide both GRAIL and the IRB/IEC/REB with a detailed written explanation of the termination or suspension.
    - If the IRB/IEC/REB terminates or suspends its approval of the study, the PI should notify GRAIL and provide GRAIL with a detailed written explanation of the termination or suspension.
- Reasons for the early closure of a study site by GRAIL or PI may include but are not limited to:
    - Failure of the PI to comply with the protocol, the requirements of the IRB/IEC/REB or local health authorities, GRAIL's procedures, or ICH/GCP guidelines.
    - Inadequate recruitment of participants by the PI.
    - Discontinuation of further study development.

9.9. Healthcare Resource Utilization

Healthcare resource utilization data will be collected from EMR and reported into the eCRF by the study investigator or designated member of the study team for all participants throughout the study. Clinical protocol-mandated procedures (e.g. blood draw) are excluded.

The data collected may be used to conduct future exploratory economic analyses and will include:

Number and types of medical encounters and cancer-specific diagnostic procedures, including clinical lab visits, imaging tests, invasive tests, and clinic visits.

10. Statistical Considerations

10.1. Analysis Methods

This section contains a brief overview of the statistical analyses planned for this study. Formal Statistical Analysis Plan (SAP) will provide full details of the analyses. The SAP will be finalized and approved prior to conducting any analyses.

This is a descriptive study to assess the safety and performance of the MCED test. No formal hypothesis testing is planned. The results will be summarized using descriptive statistics and visual displays for each of the endpoints to enable evaluation of the implementation of an investigational MCED test in clinical practice.

In the PATHFINDER 2 study, results of the MCED-V2 test are returned to healthcare providers, and will guide diagnostic evaluation for cancer in participants with MCED-V2 test positive results. In addition, the MCED-I test will be performed on the frozen plasma samples from a subset of PATHFINDER 2 participants at a time that is at least 12 months after the date of the blood draw. This will enable evaluation of performance and safety of the MCED-I test in the PATHFINDER 2 study, and concordance of MCED-V2 with MCED-I. A sampling scheme will be implemented so that a subset of samples will be tested with the MCED-I test in order to ensure that the analysis results are representative of the overall study population.

Additional details about this bridging analysis will be described in the SAP.

For participants with discordant MCED-V2 and MCED-I results, where MCED-V2 did not detect a cancer signal and MCED-I did detect a cancer signal, the cancer status will be assessed. A descriptive analysis of cancer status and any diagnostic evaluations performed for participants with discordant results will be performed.

10.2. Sample Size Determination

The sample size of this descriptive study is driven by an ability to enroll participants in the specific study subgroups based on age and sex to obtain information on diagnostic evaluation triggered by the MCED test result. The study will enroll at least 35,000 participants. Assuming that approximately 5% of participants may be excluded due to various clinical and assay evaluability reasons, approximately 33,250 analyzable participants would be expected for analyses related to receiving MCED test results and associated diagnostic evaluation. Based on previous studies we expect MCED test specificity to be in the range of 99-99.5%.

The first primary analysis objective is focused on safety evaluation. Table 7 illustrates the levels of precision that would be obtained for the primary safety Measure A (“Number of all participants with MCED positive test results and an invasive procedure divided by the total number of analyzable participants”) under six scenarios representing two levels of FP rate (1%, 0.5%) and proportions of participants with invasive procedures ranging from 10% to 50%.

We estimate that approximately 80% of true positives (TP) and 30% of FP participants may have an invasive procedure based on data from the PATHFINDER study. Based on the FP rate and the proportion of FP participants with invasive procedures, Measure A is estimated to range from approximately 0.4% to 0.8% across these scenarios (Table 7).

For reference, the proportion of FP with invasive procedures among all screened participants for existing guideline recommended non-invasive screening tests is estimated as:

- 1.66%[15] for breast cancer screening with mammography
- 10.2%[16] for colorectal cancer screening with Cologuard
- 1.88%[17] for lung cancer screening with LDCT

The expected 95% one-sided upper confidence bound for Measure A (Table 7) based on all participants with positive MCED test result is substantially lower than the reported point estimates for the existing screening tests among FP. No formal comparison to existing screening tests is planned for this study but for illustrative purposes only this study would provide a high power (>99%) even for comparison to the lowest estimate of invasive procedure rate in FP alone for guideline recommended tests reported above (1.66% for screening mammogram), i.e. to test a null hypothesis that Measure A is above 1.66%.

Table 7. Estimated Precision for Safety Measure A

FP rate	% FP with Invasive Procedures	% FP with Invasive Procedures among Analyzable Pts	% CDR[1]	% TP with Invasive Procedures	% TP with Invasive Procedures among Analyzable Pts	Measure A[2]	Upper Bound of One-Sided 95% Wilson CI for Measure A
b = 1 - a	c	d = b * c	e	f	g = e * f	d+g	
1%	10%	0.10%	0.42%	80%	0.34%	0.44%	0.50%
1%	30%	0.30%	0.42%	80%	0.34%	0.64%	0.71%
1%	50%	0.50%	0.42%	80%	0.34%	0.84%	0.92%
0.50%	10%	0.05%	0.42%	80%	0.34%	0.39%	0.45%
0.50%	30%	0.15%	0.42%	80%	0.34%	0.49%	0.55%
0.50%	50%	0.25%	0.42%	80%	0.34%	0.59%	0.66%

[1] Cancer detection rate based on simulations described in Appendix IV.
[2] % of test positive participants with an invasive procedure among the total number of analyzable participants .
Note: For simplicity, it is assumed that the FP rate reflects the proportion of FP participants among all analyzable participants (and not just those without cancer). This leads to a conservative slight overestimation of Measure A.

The second primary analysis objective is focused on MCED test performance. The expected number of positive test results and the number of cancers detected through diagnostic evaluation triggered by MCED test in analyzable participants was estimated using microsimulations described in Appendix IV. The results of the microsimulations in Table 8 indicate that approximately 300 to 462 positive MCED test results are expected with approximately 139 true positives (TP) and 163 to 323 FP. Based on these results, we expect to observe PPV of approximately 30% and 46% if MCED test specificity is 99% and 99.5%, respectively. The confidence intervals provided in Table 8 represent 2.5th and 97.5th percentiles of PPV values observed across simulations. During the study analysis, two-sided Wilson 95% CI will be provided for all test performance measures. To illustrate potential width of the Wilson CI, the following examples are provided: if PPV of 30% is observed in a study with 462 MCED test positive results, the two-sided Wilson 95% CI around PPV estimate will be (26.1%, 34.4%). If PPV of 46% is observed in a study with 300 MCED test positive results, the two-sided Wilson 95% CI around PPV estimate will be (40.4%, 51.7%). Table 8 also provides expected NPV estimates based on microsimulations.

Table 8. Results of Microsimulations[1]

Specificity	Number of Test Positive Results (95% CI)	% Test Positive (95% CI)	Number of True Positives (95% CI)	PPV (%) (95% CI)*	NPV (%) (95% CI)*
99.0%	462 (420,501)	1.39 (1.26, 1.51)	139 (119, 161)	30.2 (26.0, 35.1)	99.0 (98.9-99.1)
99.5%	300 (274, 330)	0.90 (0.82, 0.99)	137 (116, 160)	45.8 (40.2, 51.9)	99.0 (98.9-99.1)

[1] Based on SEER cancer incidence and reflecting sex and age distribution in Table 6..
*Entries in the table represent median (2.5 and 97.5 percentiles) of a specific measure across 200 simulations as described in Appendix IV.

10.3. Populations for Analyses

For the purpose of the analysis, several populations are defined in Table 9.

Table 9. Populations for Analyses

Population	Description
Enrolled	Consented participants who satisfy the inclusion and exclusion criteria at the time of consent.
Clinically Evaluable	Consented participants who satisfy the inclusion and exclusion criteria and are clinically evaluable. Evaluability reasons will be prespecified in the Clinical Data Management Plan prior to analysis.
Analyzable	Clinically evaluable participants with evaluable MCED test results.
Cancer Signal Detected	Analyzable participants with positive MCED test result
Cancer Signal Not Detected	Analyzable participants with negative MCED test result

10.4. Statistical Analyses

The SAP will be developed and finalized before database lock and will describe the participant populations to be included in the analyses, analysis methods, and the procedures accounting for missing data. This section is a summary of the planned statistical analyses of the primary and secondary study objectives.

10.4.1. General Considerations

A summary of participant disposition will be provided for all enrolled participants. This summary will present the number and percentage of participants in each analysis set (Section 10.3) and whether they had cancer diagnosis or not during the study.

The analysis of the primary and secondary study objectives will be based on the Analyzable set unless otherwise specified.

Analysis results will be presented using descriptive statistics. For categorical (including binary) variables, the number and percentage of participants in each category will be presented. For continuous variables, the number of participants (n), mean, standard deviation (SD), median, first quartile (Q1), third quartile (Q3), minimum, and maximum will be presented.

The two-sided 95% confidence intervals (CIs) will be constructed using the Wilson (score) method (Wilson, 1927[18]) for performance measures such as PPV which are not very near 0 or 1. Modified Wilson (score) confidence intervals[19-21] will be used for performance measures such as specificity and NPV which are expected to be near 1.

As part of exploratory sensitivity analyses the results will be reported by presence of prior cancer history.

10.4.2. Primary Analyses

Primary analysis Objective 1

Evaluate the safety of the MCED test in terms of diagnostic testing triggered by the MCED test result.

Safety endpoints include the number and type of invasive procedures (as described in Appendix II) and the number and type of adverse events due to diagnostic testing in all participants with a positive MCED test result.

The safety measures will describe the use of invasive procedures and include the following:

- Primary measure
    - Measure A: Number of all participants with a positive MCED test result and an invasive procedure divided by the total number of analyzable participants.
- Secondary measures:
    - Measure B: Number of invasive procedures among all participants with a positive MCED test result divided by the total number of analyzable participants.
    - Measure C: Number of all participants with a positive MCED test result and an invasive procedure divided by the total number of participants with a positive MCED test
    - Measure D: Number of invasive procedures among all participants with a positive MCED test result divided by the number of participants with a positive MCED test

The number and type of invasive procedures among all participants with a positive MCED test result and Measures A-D will be evaluated for all invasive procedures, minimally invasive (endoscopy, biopsy, other) and surgical procedures as described in Appendix II.

The results will be reported for invasive procedures prior to diagnostic resolution, during the targeted diagnostic testing triggered by MCED test result and conducted prior to confirmatory PET-CT (if applicable), and during diagnostic testing after confirmatory PET-CT (if applicable). In addition, the results will be reported for the following groups of participants:

- Cancer vs no cancer diagnosis
- Initial (the first test(s) completed to evaluate a positive MCED test result) vs subsequent (follow up test(s) ordered after review of results from initial diagnostic evaluation) evaluation
- CSO-guided vs not CSO-guided diagnostic evaluation

The number of adverse events due to follow up diagnostic testing in all participants with a positive MCED test result will be reported. The adverse events will be characterized by severity (mild, moderate, severe), whether they were considered an SAE, whether they were considered a UADE, and outcome (e.g. resolved, resolved with sequelae, ongoing at 30 minutes after end of blood draw).

A similar analysis will be conducted for TP and FP participants, separately.

Primary Analysis Objective 2

Evaluate performance of the MCED Test in individuals eligible for cancer screening.

Test performance endpoints for cancer detection and signal origin prediction are described below. The diagnosis of cancer is defined in Section 4.

Performance of the MCED test will be evaluated using performance measures defined based on a 2x2 Table 10 below which will be constructed based on the cancer status at a) 1 year and b) 2 years.

Table 10. 2x2 Contingency Table

		Cancer	
		**Present**	**Absent**
**MCED Test Result for Cancer Detection**	**Positive**	TP	FP
	**Negative**	FN	TN

Where

- TP = number of participants with true positive MCED test results,
- FP = number of participants with false positive MCED test results,
- FN = number of participants with false negative MCED test results,
- TN = number of participants with true negative MCED test results.

The following performance measures will be evaluated:

- PPV = TP / (TP + FP)
- NPV = TN / (TN + FN)
- Episode Sensitivity = TP/ (TP + FN)
    - Episode sensitivity is an ability of a screening test to detect cancers that would be diagnosed

during a follow-up period after a specific round of a screening test. Episode Sensitivity is estimated here based on the detection method which assumes that cases which are incorrectly screened as negative will manifest the disease clinically in a short interval such as 1 year[22]. The detection method is used widely to report sensitivity estimates of existing USPSTF approved screening tests in clinical trials such NLST and NELSON[23] while recognizing its limitations due to the fact that not all cancers present at time 0 may manifest clinically within a given period of time and that some cancers which are not present at time 0 may get clinically diagnosed. To mitigate the first limitation sensitivity which could lead to overestimation of sensitivity, episode sensitivity will be evaluated at several time points including 1 year and 2 years.

- Specificity = TN / (TN + FP)
    - Specificity is also estimated based on detection method and hence is subject to similar limitations. However, due to overall relatively low incidence of cancer (<2%), its estimate is expected to be less affected. Similar to sensitivity, it will be evaluated at 1 year and 2 years.
- Positive Likelihood Ratio (PLR) = episode sensitivity / (1 - specificity)
- Negative Likelihood Ratio (NLR) = (1-episode sensitivity) / specificity
- MCED cancer detection rate = TP / (TP + FP + FN + TN)
- NNS = (TP + FP + FN + TN) / TP
- CSO accuracy = (number of participants with accurate CSO prediction) / TP
    - Accuracy will be evaluated for the first CSO, second CSO prediction and either (first or second) CSO prediction.

Test performance will be evaluated at several time points:

- At the time of diagnostic resolution. At this time only performance measures based on positive MCED results can be evaluated: PPV and CSO accuracy.
- 1 year
- 2 years

The primary analysis will include overall test performance measures calculated across all cancer types for cancer detection only (as illustrated in Table 10). In addition, test performance measures will be evaluated for each CSO label. The SAP will describe pre-specified cancer groups which will be evaluated as well.

The analysis will include evaluation of test performance based on the combined cancer detection and cancer signal origin prediction. Test performance overall and by CSO label will be reported.

The detailed definitions of each performance measure at different timepoints will be provided in the SAP.

10.4.3. Secondary Analyses

Secondary Analysis Objective 1

Assess participants reported anxiety resulting from use of the MCED test.

Anxiety following the MCED test result is assessed using STAI and MCED STAI questionnaires in all participants at baseline (pre-test), post-test and at 1 year (+30D). In addition, MCED test positives will be assessed at diagnostic resolution.

Descriptive statistics will be provided for a total score at each time point and change from baseline. A two-sided t-test will be used to determine if mean changes from baseline are significantly different from zero at each post-baseline assessment at 0.05 significance level. If differences in scores do not follow a normal distribution, Wilcoxon signed rank test (Wilcoxon, 1945) will be used instead. No adjustment for multiple testing is planned. The results will be summarized descriptively for positive and negative MCED test results, separately.

Secondary Analysis Objective 2

Evaluate intention and utilization of guideline recommended cancer screening procedures after use of the MCED test.

Intention to follow standard of care cancer screening tests will be assessed using participant-reported questionnaire pre-test, post-test and at 1 year (+30D) in all participants. It is reported as Very Unlikely, Unlikely, Likely, or Very Likely to follow the healthcare provider's cancer screening recommendations.

Number and proportion of participants with each rating will be reported at each time point for the following analysis sets: Cancer Signal Not Detected, Cancer Signal Detected, Analyzable. Change from baseline (pre-test) to post-baseline assessments at each time point will be illustrated for each analysis set by:

- Proportion of participants who changed their rating from Likely or Very Likely at baseline to Unlikely or Very Unlikely post-baseline
- Proportion of participants who changed their rating from Unlikely or Very Unlikely at baseline to Likely or Very Likely post-baseline
- A matrix of baseline (rows) vs post-baseline (columns) ratings.

Utilization of standard of care cancer screening tests will be assessed in the period of time prior to study enrollment, during the first year and during the first two years after the initial MCED test is assessed. The use of most screening tests declined during pandemic[24] so only years prior to 2020 will be considered to establish a baseline for screening use. This study is expected to start enrollment in the second half of 2021 when cancer screening use may still be affected by COVID-19 to some degree so a comparison to prior utilization of cancer screening will need to be taken with caution. Utilization of cancer screening prior to enrollment will be compared to that post-test; analysis methods will be described in the SAP.

Based on use of cancer screening prior to year 2020 participants will be classified as compliant if they followed cancer screening recommendations (Appendix III), or non-compliant. Utilization of cancer screening during the study will be assessed for all participants, previously compliant and previously non-compliant participants, separately.

Confidential Property of GRAIL, Inc.

The results will be assessed in participants with negative MCED test (the main focus of this analysis), those with positive MCED test and in all participants.

Secondary Analysis Objective 3

Describe the ability of PET-CT to detect cancer in cases for which cancer signal origin directed workups do not result in diagnosis of cancer (initial workup negative cases).

The proportion of cancers detected by PET-CT will be calculated as a proportion of participants with cancer diagnosis in all participants with confirmatory PET-CT. The distribution of cancer types and clinical stage will be reported.

Secondary Analysis Objective 4

Characterize the number and type of diagnostic evaluations by test result, predicted cancer signal origin and outcome of diagnostic resolution.

The extent of diagnostic testing will be assessed as follows. Number and type of diagnostic procedures will be summarized per-participant and overall in the Signal Detected Analysis Set for imaging procedures (including anatomical and functional imaging) and for invasive procedures (minimally invasive, surgical) as described in Appendix II. Number of participants with at least one imaging or at least one invasive procedure will be reported. Distribution of the type of the first diagnostic procedure will be summarized. The distribution of the per-participant counts will be summarized using descriptive statistics (as described in section 10.4.1).

Time required to achieve diagnostic resolution is defined as the number of days from when the test result is communicated to the participant to the date of diagnostic resolution; it will be summarized using descriptive statistics by outcome of diagnostic resolution and using Kaplan-Meier methods to account for participants who did not reach a resolution at the time of the analysis.

The extent of diagnostic testing will be assessed across all participants and in the following subgroups:

- CSO prediction groups (solid tumors with common screening options, solid tumors without common screening options, hematologic malignancies)
- Top predicted CSO, where sample size allows (e.g. number of CSO calls ≥ 5)
- Number of reported CSOs (one vs two)
- Outcome of diagnostic resolution (cancer vs no cancer diagnosis)

The extent of diagnostic testing will be summarized for:

- All diagnostic testing prior to diagnostic resolution,
- The targeted diagnostic testing triggered by MCED test result and conducted prior to confirmatory PET-CT (if applicable), and
- The diagnostic testing starting from confirmatory PET-CT (inclusive) and conducted until diagnostic

resolution.

In addition, the results will be evaluated for participants with diagnostic evaluations consistent with the expected targeted diagnostic testing for predicted CSO(s). The details of the analysis will be prespecified in the SAP.

Secondary Analysis Objective 5

Evaluate the radiation exposure due to the test result-directed evaluations.

Per participant radiation exposure will be assessed. Distribution of radiation exposure will be evaluated across all test positive participants, FP, and TP participants. The results will be reported for:

- All diagnostic testing prior to diagnostic resolution,
- The targeted diagnostic testing triggered by MCED test result and conducted prior to confirmatory PET-CT (if applicable), and
- The diagnostic testing starts from confirmatory PET-CT (inclusive) and conducted until diagnostic resolution

Secondary Analysis Objective 6

Evaluate concordance of initial GRAIL test result and CSO prediction, with results from the research blood draw.

The results of the MCED test from D1 blood draw and research blood draw will be compared in participants who had both blood draws. It should be noted that research blood draws can be obtained only in participants with positive initial MCED test results. The number of negative and positive MCED results from research blood draw will be tabulated. In addition, top CSO predictions will be compared for initial vs research blood draws and proportion of cases with the same top one CSO among participants with positive MCED results from both the initial and research blood draws will be reported.

Secondary Analysis Objective 7

Assess participants reported outcomes and perceptions about the MCED test.

SF-12v2 is assessed in all participants at baseline (pre-test), post-test and at 1 year (+30D). In addition, MCED test positives will be assessed at diagnostic resolution. MICRA is assessed in all participants post-test, and satisfaction and attitude towards subsequent MCED testing are assessed post-test in MCED test negative and post diagnostic resolution in MCED test positive participants (Table 3).

The results from participant-directed questionnaires will be summarized at assessment time points for items, total score, domains and scales as appropriate for each survey (e.g. norm-based Physical and Mental Health Composite Scales computed for SF-12v2; Distress, Uncertainty and Positive experience scales for MICRA). Descriptive statistics will be provided for a total score at each time point and change from baseline.

For SF-12v2 a two-sided t-test will be used to determine if mean changes from baseline are significantly different from zero at each post-baseline assessment at 0.05 significance level. If differences in scores do not follow a normal distribution, Wilcoxon signed rank test (Wilcoxon, 1945) will be used instead. No adjustment for multiple testing is planned. The results will be summarized descriptively for positive and negative MCED test results, separately.

Secondary Analysis Objective 8

Evaluate Performance of the MCED Test in Selected Demographic and Clinical Subgroups.

The following test performance endpoints (as defined in section 10.4.2) will be evaluated in selected demographic and clinical subgroups classified on the basis of different categories of age, sex, race, ethnicity, BMI, smoking or cancer history (a detailed description of the subgroups will be provided in the SAP):

- PPV, NPV
- Episode sensitivity, specificity
- PLR, NLR
- Accuracy of CSO prediction
- Combined cancer signal detection and CSO accuracy

A descriptive summary of the distribution of the breast, lung and prostate cancer subtypes will be provided. In addition, the episode sensitivity will be summarized descriptively.

Secondary Analysis Objective 9

Evaluate MCED Test Positive Rate in Subgroups with Potential Cross-Reactive Conditions.

MCED test positive rate is defined as the proportion of participants with MCED positive test results out of all participants with analyzable MCED test results.

MCED test positive rate will be evaluated in participants with the following conditions:

1. Non-malignant hematologic conditions (Langerhans cell histiocytosis, Lymphoproliferative disorders, Mastocytoma, Monoclonal B-cell lymphocytosis, Monoclonal gammopathy of undetermined significance, Post-transplant lymphoproliferative disorder, other)[1]
2. Participants who took any cytotoxic medications in the past year
3. Participants who took any demethylating agents in the past year
4. Participants with a prior cancer history
5. Conditions described in Table 11 which may shed cfDNA and/or may affect methylation

[1] Answered "Yes" to the question "Has the participant ever been diagnosed with any of the following hematologic conditions prior to study enrollment?"

Table 11. Medical conditions to be evaluated for cross-reactivity

**Condition**[2]	**Description of condition**
Autoimmune disease	Multiple sclerosis, Rheumatoid arthritis, Systemic lupus erythematosus (lupus), Crohn's disease, ulcerative colitis
Chronic lung disease	Chronic obstructive pulmonary disease (COPD), emphysema, chronic bronchitis
HIV Infection	HIV infection
Pancreatitis	Acute pancreatitis, Chronic pancreatitis
Chronic liver disease	Chronic hepatitis B infection, Chronic hepatitis C infection, Cirrhosis (Liver cirrhosis), Fatty liver disease
Non-malignant gastrointestinal conditions	Barrett's esophagus, Chronic gastritis, Esophageal reflux disease, Stomach ulcers
Diabetes	Diabetes
Transplantation	Solid organ or stem cell (bone marrow) transplant

[2] Answered “Yes” to “Please confirm if a doctor has ever told the participant that they have any of the following health/medical conditions (check all that apply).”

10.4.4. Safety Assessments

Study-related adverse events as described in Section 9.8 will be summarized by whether they were related to study procedure or investigational test, severity (mild, moderate, severe), whether they were considered an SAE, whether they were considered a UADE, and outcome (e.g. resolved, resolved with sequelae, ongoing at 30 minutes after end of blood draw).

10.4.5. Interim Analyses

Several interim analyses will be conducted to support evaluation of the MCED test safety and performance. No hypothesis testing is planned for the primary objectives of this study and there will be no stopping early for the overwhelmingly positive results; therefore, no type I error adjustments are planned.

The following interim analyses are currently planned:

- After 1 year follow up is obtained for approximately 60% of study participants
- After 1 year follow up is obtained on all participants

Analysis methods for the interim analyses will be prespecified in the SAP.

11. Independent Data Monitoring Committee (IDMC)

An Independent Data Monitoring Committee (IDMC) will be utilized to assess the progress of the clinical study, the safety data, and key endpoints and provide recommendations to the study Sponsor on whether to continue, modify or stop the study. The IDMC is intended to provide additional assurance of participant safety and trial integrity. The IDMC will be operating under a charter developed in accordance with FDA guidance for clinical trial sponsors on “Establishment and Operation of Clinical Trial Data Monitoring Committees” (March 2006).

The study Sponsor will appoint members of the IDMC and ensure appropriate composition with respect to relevant expertise and experience, including a clinician(s) with expertise in relevant clinical specialties and at least one biostatistician knowledgeable about statistical methods for clinical trials and sequential analysis of trial data. The IDMC will meet periodically to review cumulative study data to evaluate safety, study conduct, and scientific validity and data integrity of the study.

12. Supporting Documentation & Operational Considerations

12.1. Appendix I, *Regulatory, Ethical & Study Oversight Considerations*

12.1.1. Appendix I(a), *Regulatory & Ethical Considerations*

12.1.2. Appendix I(b), *Financial Disclosure*

12.1.3. Appendix I(c), *Data Protection*

12.1.4. Appendix I(d), *Study Supplies*

12.1.5. Appendix I(e), *Source Documents*

12.1.6. Appendix I(f), *Study Data Quality Assurance*

12.1.7. Appendix I(g), *Record Retention*

12.1.8. Appendix I(h), *Publication Policy*

12.2. Appendix II, Examples of Imaging and Invasive Tests

12.3. Appendix III, Cancer Screening Compliance

12.4. Appendix IV, Description of the Simulations

12.5. Appendix V, Abbreviations

12.6. Appendix VI, References

12.7. Appendix VII, Clinical Study Protocol Amendment History

Appendix I. Regulatory, Ethical & Study Oversight Considerations

Appendix I(a). Regulatory & Ethical Considerations

- The study sites will start enrolling upon receipt of written approval by GRAIL.
- This study will be conducted in accordance with the protocol and with the following:
    - Consensus ethical principles derived from international guidelines including the Declaration of Helsinki and Council for International Organizations of Medical Sciences (CIOMS) International Ethical Guidelines.
    - Applicable ICH/GCP guidelines.
    - The provisions specified in 21 CFR 11, 21 CFR 50, 21 CFR 54, 21 CFR 56, and 21 CFR 812.
    - Other applicable laws and regulations.
    - The study will maintain the following controls of study materials:
        - The protocol, protocol amendments, ICF(s), Instructions for Use (IFU), and other relevant documents (e.g, advertisements, other participant-facing materials) must be submitted to an IRB/IEC/REB by the PI and reviewed and approved by the IRB/IEC/REB before the study is initiated.
        - Any amendments to the protocol will require IRB/IEC/REB approval before implementation of changes made to the study design, except for changes necessary to eliminate an immediate hazard to study participants.
        - Clinical documents will be maintained according to the protocol and applicable laws and GCP standards to demonstrate the validity of the study and the integrity of the data collected.
        - Regulatory authorities, the IRBs/IECs/REBs, and/or GRAIL, or its designees or partners, may request access to all source documents, electronic CRFs (eCRFs), and other study documentation for audits or inspections, including on-site.

Obligations of PIs

It is the responsibility of the PI(s) to:

- Perform the study in accordance with ICH/GCP and the applicable regulatory requirements.
- Ensure compliance with all procedures required by the protocol.
- Provide the protocol-required data elements to GRAIL, in a manner that is attributable, legible, contemporaneous, and accurate.
- Comply with any privacy and security requirements applicable to their institution or organization.

Appendix I. Regulatory, Ethical & Study Oversight Considerations (Cont.)

Appendix I(a). Regulatory & Ethical Considerations

- Provide GRAIL representatives and/or other authorized individuals with direct access to clinical documents in order to allow for Source Data Review (SDR) and/or audit/inspection activities to be conducted.
- Understand the applicable laws, GCP standards, and protocol, and agree to abide by them.
- Provide written summaries of the status of the study to the IRB/IEC/REB annually or more frequently in accordance with the requirements, policies, and procedures established by the IRB/IEC/REB. At the end of the study, a summary of the study's outcome should be prepared for the IRB/IEC/REB.
- Document, report, and notify the IRB/IEC/REB of UADEs or other significant safety findings as required by IRB/IEC/REB procedures.
- Provide oversight of the conduct of the study at the study site and adhere to requirements of Title 21, Code of Federal Regulations (21 CFR), ICH/GCP guidelines, the IRB/IEC/REB, and all other applicable local regulations.

Obligations of the Sponsor

It is the responsibility of GRAIL, the Sponsor of this study to:

- Ensure the proper conduct of the study, including ethical considerations, protocol compliance, and integrity and validity of study data.
- Monitor the study execution to ensure a high level of ethical, scientific, technical, and regulatory quality.

At regular intervals during the study, the study sites will be contacted, through monitoring visits, letters, or telephone calls, by a representative of GRAIL to review study progress, PI and participant compliance with protocol requirements, and any emergent problems.

Appendix I. Regulatory, Ethical & Study Oversight Considerations

Appendix I(b). Financial Disclosure

PIs and Sub-Investigators will provide GRAIL with sufficient and accurate financial information, as requested to allow GRAIL to submit complete and accurate financial certification or disclosure statements to the appropriate regulatory authorities. PI and Sub-Is are responsible for providing information on financial interests during the course of the study and for one year after completion of the study.

Appendix I. Regulatory, Ethical & Study Oversight Considerations

Appendix I(c). Data Protection

- The collection of participants' data for this protocol is described in Section 9.6, Clinical Data Collection.
- Participants will be assigned a unique identifier (ID) by GRAIL. Any participant records or datasets that are transferred to GRAIL will contain the identifier only; participant names or any information which would make the participant identifiable will not be transferred.
- The participant must be informed that their personal study-related data will be used by GRAIL in accordance with local data protection law. The level of disclosure must also be explained to the participant.
- The participant must be informed that their medical records may be examined by Clinical Quality Assurance auditors or other authorized personnel appointed by GRAIL, by appropriate IRB/IEC/REB members, and by inspectors from regulatory authorities.

Appendix I. Regulatory, Ethical & Study Oversight Considerations

Appendix I(d). Study Supplies

- Supplies and materials required for blood collection and shipping will be provided by GRAIL or its designee. GRAIL may provide tablets for electronic consent and questionnaire administration, as applicable by site requirements. The disposition of study supplies after study completion or termination is controlled by the Clinical Trial Agreement between GRAIL and the relevant clinical institution or study sites.
- Blood collection tubes, study test kits (including shipping materials) will be supplied and replenished at participating study sites on an as-needed basis. These supplies and detailed instructions for blood collection, handling, and shipping will be provided by GRAIL or its designee.
- Clinical blood collection (study test kits and shipping materials) should be stored in a secure location at each participating clinical site. Arrangements can be made to have these supplies replenished more frequently so as to reduce the number that need to be stored at any given time. The blood collection packages have visible expiration dates that reflect the expiration dates of the enclosed vacutainer tubes and these should be regularly checked prior to use. Expired clinical blood collection packages should be discarded, per GRAIL's instruction and clinical site operating procedures.

Appendix I. Regulatory, Ethical & Study Oversight Considerations

Appendix I(e). Source Documents

- Source documents provide evidence for the existence of the participant and substantiate the integrity of the data collected. Source documents are filed at the investigative study site.
- Data reported in the eCRF that are transcribed from source documents must be consistent with the source documents or the discrepancies must be explained. The PI may need to request previous medical records or transfer records, depending on the study. Also, current medical records must be available.
- Definition of what constitutes source data can be found in Source Data Location Form.

Appendix I. Regulatory, Ethical & Study Oversight Considerations

Appendix I(f). Study Data Quality Assurance (QA)

Regardless of the method(s) for study data collection, the data will be stored and displayed as an eCRF. The PI is responsible for affirming that the data recorded in the CRF are accurate and complete, to the best of their knowledge, by providing their signature.

- The PI must maintain accurate documentation (source data) that supports the information entered in the CRF, unless the CRF is being utilized as a source document.
- The PI must permit study-related monitoring, audits, IRB/IEC/REB review, and regulatory agency inspections and provide direct access to source data documents and CRFs.
- Clinical Study Monitors will perform ongoing source data verification (SDV) to confirm that data entered into the CRF by authorized study site personnel are accurate, complete, and verifiable from source documents; that the safety and rights of participants are being protected; and that the study is being conducted in accordance with the currently approved protocol and any other study agreements, ICH/GCP, and all applicable regulatory requirements.

EDC systems used to capture and manage study data will allow for manual and automated checks to ensure that the records are complete, consistent, and accurate. Data discrepancies and resolutions will be managed within the EDC system. A proportion of electronic record-based items will be subject to SDR against the original medical record for accuracy, as applicable. GRAIL or its designee will monitor the clinical data captured in the EDC system by the study sites according to the approved Clinical Data Management Plan (CDMP) and Clinical Study Monitoring Plan (SMP), respectively. The degree to which data quality will be monitored by GRAIL and at each study site, will be determined by taking a risk-based approach. The EDC system will store audit trails of data entry and modification throughout the course of the study, as well as records of activities such as monitoring, data discrepancy resolution, PI signature, and locking of data.

Appendix I. Regulatory, Ethical & Study Oversight Considerations

Appendix I(g). Record Retention

Under 21 CFR 812 - Investigational Device Exemptions, Section 140 - Records, Part D - Retention Period, the study records must be retained for two years after the latter of the following two dates: (i) the date on which the investigation is terminated or completed, or (ii) the date that the Premarket Approval (PMA) is approved.

No records may be destroyed during the retention period without the written approval of GRAIL. No records may be transferred to another location or party without written notification to GRAIL.

Appendix I. Regulatory, Ethical & Study Oversight Considerations

Appendix I(h). Publication Policy

Any publication or other disclosure of the results of this study, including any publication in manuscript form and any presentation at scientific or medical meetings, will be made in accordance with the terms of the Clinical Trial Agreement (CTA) between GRAIL and the center or study site responsible for performing activities for the study.

Appendix II. Examples of Imaging and Invasive Tests

Type of Diagnostic Testing	Category	Specific procedure
Imaging	Anatomical Imaging Test	Plain X-Ray
		CT
		MRI
		Mammogram
		Ultrasound (thyroid, abdominal, pelvic, transvaginal)
	Functional Imaging Test	PET-CT
		PET-MRI
		Nuclear Medicine Scan
Invasive Procedures	Minimally Invasive Procedure	Anoscopy
		Colonoscopy
		Sigmoidoscopy
		Esophagogastroduodenoscopy (EGD)
		Endoscopy
		Endoscopic Ultrasound
		Endoscopic retrograde cholangiopancreatography (ERCP)
		Bronchoscopy
		Cystoscopy
		Colposcopy
		Hysteroscopy
		Fine needle aspiration (FNA) biopsy
		Core needle biopsy
		Bone marrow biopsy
		Incisional or excisional biopsy
	Surgical procedure	Surgery with perioperative biopsy

Appendix III. Cancer Screening Compliance

The following items (as applicable) need to be satisfied in order for a participant to be considered compliant with cancer screening recommendations:

1. Lung cancer: annual LDCT in participants satisfying USPSTF criteria[25].
2. Colon cancer: annual FOBT, annual FIT, FIT-DNA every 3 years, sigmoidoscopy every 5 years, or colonoscopy every 10 years in participants <=75 years old[26].
3. Breast cancer: mammography every 2 years in women 50-74 years old[15].
4. Cervix cancer: pap smear with HPV testing every 5 years, or pap smear every 3 years in women <=65 years old[27].

Appendix IV. Description of the Simulations

The following assumptions were used in simulations:

1. Cancer incidence in the study population.
   a. Cancer incidence is estimated based on the cancer incidence in Surveillance, Epidemiology, and End Results Program (SEER) in years 2006-2015 for the following 17 cancers: anus and anorectum, bladder, cervix uteri, colon and rectum, corpus and uterus, esophagus, head and neck, breast, kidney, liver and intrahepatic bile duct, lung, lymphoma, melanoma, ovary, pancreas, prostate, stomach.
   b. SEER incidence is weighted to reflect the following age distribution (consistent with Table 6): 25% in 50-59 yr group, 47.5% in 60-69 yr group, 25% in 70-79 yr group and 2.5% in 80+ yr group.
2. MCED test performance was estimated based on the results from the analysis of >4,000 participants from the third substudy of Circulating Cell-free Genome Atlas study (CCGA3) with an overall sensitivity of approximately 52% across cancer types[10]. To account for possible differences in test performance in detection of cancers which were not yet identified clinically (compared to cancers diagnosed clinically at enrollment in CCGA3 study), a reduction in sensitivity was considered.
3. Because the natural history of cfDNA-detectable cancer cases is not established, we explored a range of plausible assumptions for the distribution of dwell times (ie, how much time each cancer spends in a given stage) per cancer per stage (Hubbell et al 2020).

Participant histories were simulated by using incidence rates for clinical detection per year per cancer type and stage as described above. Exponential time to clinical detection of first cancer was generated for each participant, cancer type was generated by drawing from a multinomial cancer type distribution and then cancer stage at clinical detection was generated by drawing from multinomial stage distribution. Starting from the time of the clinically detected cancer and using the corresponding dwell times, the times when participants progress through the previous stages and when cancer started was back simulated. If cancer started before time 0 (time of the study test), it is expected to be detected with assumed sensitivity for a given cancer and stage. A total of 200 simulations were conducted and a median, 2.5th and 97.5% percentiles for the number of “signal detected” test results, number of cancers detected through diagnostic evaluation following a “signal detected” test result and performance measures across simulations were reported.

Appendix V. Abbreviations

Term	Definition
AACR	American Association for Cancer Research
ADE	Adverse Device Effect
AE	Adverse Effect
ASCO	American Society of Clinical Oncology
BMI	Body Mass Index
CBC	Complete Blood Count
CCGA	Circulating Cell-free Genome Atlas
CDMP	Clinical Data Management Plan
cfDNA	Cell-free DNA
cfNA	Cell-free nucleic acids
CFR	Code of Federal Regulations
CI	Confidence Interval
CIOMS	Council for International Organizations of Medical Sciences
CLIA	Clinical Laboratory Improvement Amendments
CMP	Comprehensive Metabolic Panel
CSO	Cancer Signal Origin
CT	Computed Tomography
CTA	Clinical Trial Agreement
DNA	Deoxyribonucleic Acid
DOB	Date of Birth
eCRF	Electronic Case Report Form
EDC	Electronic Data Capture
EMR	Electronic Medical Records
EOS	End of Study
ePRO	Electronic Participant-Reported Outcome
ESMO	European Society for Medical Oncology
FDA	Food and Drug Administration
FP	False Positive
GCP	Good Clinical Practice
HIPAA	Health Insurance Portability and Accountability Act

HPV	Human Papillomavirus
ICD-O-3	International Classification of Diseases for Oncology, third edition
ICF	Informed Consent Form
ICH	International Council for Harmonization of Technical Requirements for Pharmaceuticals for Human Use
ID	Identifier
IDMC	Independent Data Monitoring Committee
IEC	Independent Ethics Committee
IFU	Instructions For Use
IRB	Institutional Review Board
IV	Intravenous
LDCT	Low dose computed tomography
MCED	Multi-Cancer Early Detection
MICRA	Multidimensional Impact of Cancer Risk Assessment
MRI	Magnetic Resonance Imaging
NGS	Next Generation Sequencing
NNS	Number Needed to Screen
NPV	Negative Predictive Value
PDs	Protocol Deviations
PET-CT	Positron Emission Tomography–Computed Tomography
PI	Principal Investigator
PPV	Positive Predictive Value
QA	Quality Assurance (in appendix)
REB	Research Ethics Board
ROR	Return of Result
SAE	Serious Adverse Event
SAP	Statistical Analysis Plan
SDR	Source Data Review
SDV	Source Data Verification
SEER	Surveillance, Epidemiology, and End Results Program
SMP	Study Monitoring Plan
SMS	Short Message Service
SoA	Schedule of Activities

STAI	State Trait Anxiety Inventory
TRF	Test Requisition Form
TRR	Test Result Report
TP	True Positive
UADE	Unanticipated Adverse Device Effects

Appendix VI. References

1. Imperiale TF, Ransohoff DF, Itzkowitz SH, et al. Multitarget stool DNA testing for colorectal cancer screening. New England Journal of Medicine. 2014; 370: 1287–97.
2. Liu MC, Oxford GR, Klein EA. Sensitive and specific multi-cancer detection and localization using methylation signatures in cell-free DNA. Annals of Oncology. 2020; 31(6):745-759.
3. Aravanis A, Oxnard G, Maddala T, et al. Development of plasma cell-free DNA (cfDNA) assays for early cancer detection: first insights from the Circulating Cell-Free Genome Atlas Study (CCGA). In: Proceedings of the American Association for Cancer Research Annual Meeting 2018; Apr 14-18, 2018; Chicago, IL. Philadelphia (PA): AACR; Cancer Res. 2018;78(13 Suppl): Abstract LB-343.
4. Oxnard G, et al. Genome-wide sequencing for early stage lung cancer detection from plasma cell-free DNA (cfDNA): The Circulating Cancer Genome Atlas (CCGA) study. Presented at: American Society of Clinical Oncology (ASCO) 2018; June 1-5, 2018;
Chicago, IL. J Clin Oncol. 2018;36(no. 18_suppl): Abstract LBA8501.
5. Liu M, et al. Breast cancer cell-free DNA (cfDNA) profiles reflect underlying tumor biology: The Circulating Cell-Free Genome Atlas (CCGA) study. Presented at: American Society of Clinical Oncology (ASCO) 2018; June 1-5, 2018; Chicago, IL. J Clin Oncol. 2018;36(no. 15_suppl): Abstract 536.
6. Klein E, et al. Development of a comprehensive cell-free DNA (cfDNA) assay for early detection of multiple tumor types: The Circulating Cell-free Genome Atlas (CCGA) study. Presented at: American Society of Clinical Oncology (ASCO) 2018; June 1-5, 2018; Chicago, IL. J Clin Oncol . 2018;36(no. 15_suppl): Abstract 12021.
7. Swanton C, et al. Prevalence of clonal hematopoiesis of indeterminate potential (CHIP) measured by an ultra-sensitive sequencing assay: Exploratory analysis of the Circulating Cancer Genome Atlas (CCGA) study. Presented at: American Society of Clinical Oncology (ASCO) 2018; June 1-5, 2018; Chicago, IL. J Clin Oncol . 2018;36(no. 15_suppl): Abstract 12003.
8. Liu M, et al. Plasma cell-free DNA (cfDNA) assays for early multi-cancer detection: the Circulating Cell-Free Genome Atlas (CCGA) study. Presented at: European Society for Medical Oncology (ESMO) 2018; October 20, 2018; Munich, Germany. Annals Oncol. 2018;29(suppl_8):viii14-viii57.
9. Liu M, Jamshidi A, Venn O, et al. Genome-wide cell-free DNA (cfDNA) methylation signatures and effect on tissue of origin (TOO) performance. Presented at: American Society of Clinical Oncology (ASCO) 2019; May 31- June 4, 2019. Chicago, IL. J Clin Oncol. 2019;37(15_suppl):3049. Cohn A, Seiden M, Kurtzman K, et
10. Klein EA, et al. Clinical validation of a targeted methylation-based multi-cancer early detection test. Presented at: American Association for Cancer Research Virtual Annual Meeting, Week 1; April 10-15, 2021. Abstract LB013.
11. Cohn A, Seiden M, Kurtzman K, et al. 2019. The Circulating Cell-free Genome Atlas (CCGA) study: follow-up (F/U) on non-cancer participants with cancer-like cell-free DNA signals. Poster session presented at: American Society of Clinical Oncology (ASCO) 2018; June 1-5, 2018; Chicago, IL.
12. Marteau TM, Bekker H. The development of a six-item short-form of the state scale of the Spielberger state-trait anxiety inventory (STAI). Br J Clin Psychol 1992;31 (Pt 3:301–6)
13. https://www.acr.org/-/media/ACR/Files/Practice-Parameters/FDG-PET-CT.pdf
14. Definitions, Statistics and Graphs: Statistics. United States: National Institutes of Health: National Cancer Institute Website. https://cancercontrol.cancer.gov/ocs/statistics/statistics.html Updated February 8, 2019.

15. Breast Cancer: Screening. U.S. Preventive Services Website. https://uspreventiveservicestaskforce.org/uspstf/recommendation/breast-cancer-screening
16. Imperiale TF, Ransohoff DF, Itzkowitz SH, Levin TR, Lavin P, Lidgard GP, Ahlquist DA, Berger BM. Multitarget stool DNA testing for colorectal-cancer screening. N Engl J Med. 2014 Apr 3;370(14):1287-97. doi: 10.1056/NEJMoa1311194. Epub 2014 Mar 19. PMID: 24645800. All false positives undergo colonoscopy, consider as invasive procedure.
17. National Lung Screening Trial Research Team, Aberle DR, Adams AM, et al. Reduced lung-cancer mortality with low-dose computed tomographic screening. N Engl J Med. 2011;365(5):395-409. doi:10.1056/NEJMoa1102873. table 3 % of Y0 screened individuals with invasive procedure subtracting % diagnosed with lung cancer.
18. Wilson, E. B. (1927). Probable Inference, the Law of Succession, and Statistical Inference. Journal of the American Statistical Association, 22(158), 209-212.
19. Newcombe, R. G. (1998a), “Two-sided confidence intervals for the single proportion: Comparison of seven methods,” Statistics in Medicine, 17, 857–872.
20. Newcombe, R. G. (1998b), “Interval estimation for the difference between independent proportions: Com- parison of eleven methods,” Statistics in Medicine, 17, 873–890.
21. Agresti, A., and Coull, B. A. (1998), “Approximate is better than "exact" for interval estimation of binomial proportions,” The American Statistician, 52, 119–126
22. Day NE. Estimating the sensitivity of a screening test. J Epidemiol Community Health. 1985;39(4):364-366. https://www.ncbi.nlm.nih.gov/pmc/articles/PMC1052473/
23. Klaveren, R. J. Management of Lung Nodules Detected by Volume CT Scanning. The New England Journal of Medicine. N Engl J Med 2009; 361:2221-2229. DOI: 10.1056/NEJMoa0906085 https://www.nejm.org/doi/full/10.1056/nejmoa0906085
24. Chen RC, Haynes K, Du S, Barron J, Katz AJ. Association of Cancer Screening Deficit in the United States With the COVID-19 Pandemic. JAMA Oncol. Published online April 29, 2021. doi:10.1001/jamaoncol.2021.0884
25. Lung Cancer: Screening. U.S. Preventive Services Website. https://uspreventiveservicestaskforce.org/uspstf/recommendation/lung-cancer-screening
26. Colorectal Cancer: Screening. U.S. Preventive Services Website. https://uspreventiveservicestaskforce.org/uspstf/recommendation/colorectal-cancer-screening
27. Cervical Cancer: Screening. U.S. Preventive Services Website. https://uspreventiveservicestaskforce.org/uspstf/recommendation/cervical-cancer-screening
28. United States Department of Health and Human Services (US DHHS), Centers for Disease Control and Prevention (CDC), National Center for Health Statistics (NCHS), Bridged-Race Population Estimates, United States July 1st resident population by state, county, age, sex, bridged-race, and Hispanic origin. Compiled from 1990-1999 bridged-race Vintage 2020 (2010-2020) postcensal population estimates (released by NCHS on 9/22/2021). Available on CDC WONDER Online Database. Accessed at http://wonder.cdc.gov/bridged-race-v2020.html on Jan 5, 2022).
29. Age and Sex Composition in the United States: 2020. United States Census Bureau. Retrieved April 3, 2023, from https://www.census.gov/data/tables/2020/demo/age-and-sex/2020-age-sex-composition.html

Appendix VII. Clinical Study Protocol Amendment History

Clinical Study Protocol Amendment Summary for the current protocol amendment is located directly before the Table of Contents (TOC).

Clinical Study Protocol Amendment 3, Protocol Version 4, Version Date 21JAN2025

This protocol amendment is considered to be substantial based on the criteria set forth in Article 10(a) of Directive 2001/20/EC of the European Parliament and the Council of the EU.

Table 11, Overall Rationale for the Clinical Study Protocol Amendment

Amendment 3, Protocol Version 4, Version Date 21JAN2025

Section Number & Name	Description of Change	Brief Rationale
Throughout document	Update GRAIL, LLC to GRAIL, Inc.	General administrative updates
Throughout document	Removed exploratory objective of evaluating DNA based and other biomarkers in tissue compared to blood from cancer cases and all mention of tissue collection activities.	Aligns with the change in study design not to collect tissue specimens from participants with cancer diagnosis.
Throughout document	Clarified the use of the updated version of the MCED test on participant samples and that any new test results obtained will not be returned to study investigators or participants.	Clarifies that results of testing with an updated version of the MCED test using residual participant samples will not be returned.
10.4.2 Primary Analysis	Included description of additional test performance endpoints.	Aligned with the test performance analysis detailed in the SAP.
10.4.3 Secondary Analysis	Added two additional secondary analysis objectives: assessing test performance in select demographic and clinical subgroups, and evaluating the effect of certain medical conditions and clinical subgroups on MCED test positive rate.	Changes align the protocol with updates to the secondary analysis objectives detailed in the SAP.

Clinical Study Protocol Amendment 2, Protocol Version 3, Version Date 09AUG2023

This protocol amendment is considered to be substantial based on the criteria set forth in Article 10(a) of Directive 2001/20/EC of the European Parliament and the Council of the EU.

Table 12, Overall Rationale for the Clinical Study Protocol Amendment

The protocol amendment was undertaken primarily to increase the study size to at least 35,000 and no more than 38,500 participants in order to ensure adequate data on safety and efficacy for clinical validation. Additional changes were implemented to refine the age targets for enrollment to better match the study objectives and intended use. Minor modifications to the text of the protocol were performed to clarify the study guidelines and procedures for the clinical sites.

Amendment 2, Protocol Version 3, Version Date 09AUG2023

Section Number & Name	Description of Change	Brief Rationale
2.1 Clinical Study Protocol Synopsis	Language used to describe the percentage of participants diagnosed with cancer as a result of a positive MCED test across the full cohort (TP / (TP + FP + FN + TN) changed from "proportion of cancers detected by screening" to "MCED detection rate".	MCED detection rate better describes those cases where cancer diagnosis resulted from screening with the MCED test vs. other screening modalities.
2.3 Schedule of Activities	Added: Assessment of Signs and Symptoms Prior to Blood Draw	Provides clarity to the sites by adding the completion of the existing Signs and Symptoms eCRF into the study timeline, consistent with the manner in which the form has been completed in the study to date.
3.2 Background of the Study Test	Movement of PATHFINDER Study to completed studies section with inclusion of results.	PATHFINDER is now completed and the results are relevant background information for evaluating the study protocol.
4. Primary Analysis Objectives and Endpoints	Clarified that invasive procedure rate will be assessed for both those participants diagnosed with cancer	Better reflects the safety of the MCED test and aligns with FDA guidance.

Section Number & Name	Description of Change	Brief Rationale
	and in those with no cancer diagnosis at the time of diagnostic resolution.<br>Also, added number and type of adverse events due to diagnostic testing in all participants with a positive MCED test result as a primary endpoint.	
4. Secondary Analysis Objectives and Endpoints	Typographical error corrected, number of secondary study objectives corrected from six to seven.	There is no change in the number of secondary objectives, previous versions of the protocol incorrectly stated there were six secondary objectives instead of seven.
5.1 Overall Design	HPV testing added to recommended procedures for diagnostic evaluation of CSO Cervix.	Acknowledges the standard of care practice for cervical cancer evaluation and diagnosis.
5.2 Estimated Study Duration	Update to 6.75 years.	Enrollment period extended, follow up period remains 36 months.
6. Study Population	Age targets changed to:<br>50-59: 25%<br>60-69: 47.5%<br>70-79: 25%<br>80+: 2.5%<br>Split evenly between men and women	The age enrollment targets are updated to reflect more realistic targets to enrich the study population for older age compared to the US census. In addition, the proportion of the 80+ age group is reduced to align with guideline recommendations for cancer screening that generally focus on individuals who are younger than 80 years of age, and to take into account the health status of older individuals and the benefits/risks of screening and diagnostic workup.
8.1 Participant Withdrawal From Study	Clarified that participants may be withdrawn from the study if they can not be reached to have their test results returned within 90 days.	Ensures that all participants receive their test results within a consistent time period after the initial blood draw.

Section Number & Name	Description of Change	Brief Rationale
8.1 Participant Withdrawal From Study	Clarifies that once withdrawn, participants are not eligible to re-enroll in the study.	Ensures that the study includes unique participants only and multiple study IDs are not assigned to participants.
8.1 Participant Withdrawal From Study	Language added to clarify that failure to perform a repeat blood draw, when required, within 60 days following consent will result in withdrawal of the participant.	Ensures that eligibility criteria and baseline assessment is accurate at time of blood draw.
9.3 Blood Draw & 9.6 Clinical Data Collection	Personal Health Information updated to Protected Health Information.	Reflects current unique identifiers collected on blood collection tube and TRF.
10.2 Sample Size Determination	Statistical section updated to reflect the increased study size.	The increase in participant enrollment required a recalculation of expected estimates for safety analyses and test performance analyses.
10.4.2 Primary Analyses	Updated safety endpoints to include the number and type of invasive procedures and the number and type of adverse events due to diagnostic testing in all participants with a positive MCED test result.<br>Clarified that analyses will be conducted overall and for each CSO label and will include analysis for cancer detection only as well as combined cancer detection & CSO prediction.	Better reflects the safety of the MCED test and aligns with FDA guidance. Additional details will be provided in the future SAP.
10.4.3 Secondary Analyses	Time required to achieve diagnostic resolution is defined as the number of days from when the test result is communicated to the participant.	Improves the precision of measuring the time required for diagnostic workup.
Appendix II	"Type of Diagnostic Testing" column added.	Clarifies categories that fall under imaging and invasive procedures.

Section Number & Name	Description of Change	Brief Rationale
Appendix IV	Described additional consideration used in simulations.	Clarifies assumption about sensitivity for cancer detection in the simulations
Throughout Document	Study expanded to at least 35,000 and no more than 38,500 participants, enrollment period to 45 months.	Study size increased to ensure adequate data on safety and efficacy for clinical validation.

Clinical Study Protocol Amendment 1, Protocol Version 2, Version Date 28FEB2022

This protocol amendment is considered to be substantial based on the criteria set forth in Article 10(a) of Directive 2001/20/EC of the European Parliament and the Council of the EU.

Table 13, Overall Rationale for the Clinical Study Protocol Amendment

The protocol has been amended to reflect changes to the study design, including the sample size and target population, to update exclusion criteria, to clarify aspects of the collection of blood and clinical data, and to update the definition of cancer used in the study.

Amendment 1, Protocol Version 2, Version Date 28FEB2022

Section Number & Name	Description of Change	Brief Rationale
3.2 Background of the Study Test	Add NHS-Gallerias an ongoing study	New GRAIL studies initiated since Version 1.0 of protocol was released
4 Objectives & Endpoints	Update definition of cancer	Clarify language on cancer definition for hematologic conditions that are classified as cancer and remove references to stage
5.1 (Table 4) Cancer Signal Origin Directed Diagnostic Evaluations	Update Recommended Workup	Additional recommendations provided for hematologic CSOs to

Section Number & Name	Description of Change	Brief Rationale
		align with clinical practice guidelines.
5.2 Estimated Study Duration	Update to 4.5 years	Enrollment period extended to 18 months
6 Study Population	Update Race Table, superscript and add ethnicity to Hispanic	Align enrollment targets for race and ethnicity to population distribution in adults aged 50 years and older
6.2 Exclusion Criteria	Add clarifying language that non-metastatic basal cell carcinoma and squamous cell carcinoma of the skin are not exclusion criteria	Align with definition of cancer in section 4
6.2 Exclusion Criteria	Add pregnancy exclusion	Change is being implemented due to lack of evidence regarding test performance in women who are pregnant
7.2.1 Active Clinical Follow-Up	Update start of active follow up from date of enrollment to date of blood draw	Clarify that active follow begins on date of blood draw since enrollment and blood draw may not occur on the same day
9.3 Blood Draw	Specify minimum number of tubes is 3 with at least 3 ml in each tube	Align protocol specifications with study specimen collection and handling manual
9.5 Diagnostic Work-Up of a "Cancer Signal Detected" Test Result	Update Cancer Diagnosis definition	Align with definition of cancer in section 4
9.6 Clinical Data Collection	Update clinical data collection for individuals with a "cancer signal detected" test result	Clarified what clinical data are collected depending on how a cancer is detected and diagnosed

Section Number & Name	Description of Change	Brief Rationale
9.8.3 Adverse Device Effects (ADEs)	Adverse Events	Clarify what is considered an ADE, add "Other medically important serious event" to SAE criteria
10.2 Sample Size Determination	Update determination based on enrollment of 20,000 subjects	Increase clinical evidence generation to support evaluation of MCED test safety and effectiveness
10.4.5 Interim Analyses	Update the timing of interim analyses	Align interim analyses with Year 1 follow up
Appendix I(b) Financial Disclosure	Remove "Financial compensation will not be provided to participants in this study."	Gift cards will be provided for completion of patient reported questionnaires
Appendix II. Examples of Imaging and Invasive Tests	Add additional examples of imaging and invasive tests	Align examples provided in the appendix with expected procedures based on CSO-directed diagnostic evaluations
Throughout document	Replace Data Safety Monitoring Board (DSMB) with Independent Data Monitoring Committee (IDMC)	Align terminology with FDA guidance
Throughout document	Update GRAIL, Inc. to GRAIL, LLC	General administrative updates
Throughout document	Increase study enrollment to 20,000 subjects (at least 200 cancer signal detected MCED test results), enrollment period to 18 months, and number of institutions to 40	Increase clinical evidence generation to support evaluation of MCED test safety and effectiveness
Throughout document	Replace Galleri with GRAIL's multi-cancer early detection test (MCED)	Remove references to GRAIL's commercial test Galleri as samples may be processed on different versions of the MCED test

STATISTICAL ANALYSIS PLAN (SAP) for PREMARKET APPROVAL (PMA) SUBMISSION

GRAIL-012 The PATHFINDER-2 Study

GRAIL Multi-Cancer Test

**Study Title:**	The PATHFINDER 2 Study: Evaluating the Safety and Performance of the GRAIL Multi-Cancer Early Detection Test in an Eligible Screening Population
**Study Number:**	GRAIL-012, NCT05155605
**Sponsor:**	GRAIL, LLC<br>1525 O'Brien Dr<br>Menlo Park, CA 94025
**SAP Version:**	1.0
**SAP Date:**	May 13, 2024

CONFIDENTIALITY
This document is the confidential and proprietary information of GRAIL, LLC. Except as required by federal, state, or local laws or regulations, this document may not be disclosed to others, in whole or in part without prior written permission of GRAIL, Inc.

DOCUMENT HISTORY

Version	Date	Replaces	Description of Change
1.0	May 13, 2024	N/A	Initial Release

CONFIDENTIALITY
This document is the confidential and proprietary information of GRAIL, LLC. Except as required by federal, state, or local laws or regulations, this document may not be disclosed to others, in whole or in part without prior written permission of GRAIL, Inc.

The signature below constitutes the approval of this SAP.

[redacted]

14-May-2024

Date

Distinguished Scientist, Medical Affairs

[redacted]

13-May-2024

Date

Sr Vice President, Data Science & CSO, Research

[redacted]

14-May-2024

Date

Vice President, Biostatistics and Data Management

CONFIDENTIALITY

This document is the confidential and proprietary information of GRAIL, LLC. Except as required by federal, state, or local laws or regulations, this document may not be disclosed to others, in whole or in part without prior written permission of GRAIL, Inc.

Co-authors of the SAP:

[redacted]

Sr Director, Biostatistics

13-May-2024

Date

[redacted]

Vice President, Clinical Development

13-May-2024

Date

CONFIDENTIALITY

This document is the confidential and proprietary information of GRAIL, LLC. Except as required by federal, state, or local laws or regulations, this document may not be disclosed to others, in whole or in part without prior written permission of GRAIL, Inc.

Contents

1. Definitions and Abbreviations 9
2. Background and Rationale 11
3. Study Objectives 11
   3.1 Primary Objectives 11
   3.2 Secondary Objectives 13
   3.3 Exploratory Objectives 14
4. Study Design 14
   4.1 PATHFINDER 2 Study Design 14
   4.2 Bridging Analysis for MCED-I Test 15
5. Study Population 16
6. Data 16
   6.1 Data Sources 16
   6.2 Clinical Data 17
   6.3 MCED Test Data 18
   6.4 Data Lock Process and Merge 18
7. Measures to Minimize Bias 19
8. Outcome Definitions 19
   8.1 GRAIL MCED Test Result 19
   8.2 Target Clinical Outcome 20
      8.2.1 Cancer 20
      8.2.2 Cancer Clinical Stage 21
      8.2.3 Cancer Type 21
      8.2.4 Clinical CSO 21
      8.2.5 Cancer of Unknown Primary 21
      8.2.6 Multiple Cancers 22
      8.2.7 Definition of Cancer Participant for Specific Analysis 22
      8.2.8 Definition of Non-cancer Participant for Specific Analysis 22
9. Handling of Dropouts or Missing Data 23
10. Analysis Sets 23
   10.1 Consented Analysis Set 23
   10.2 Clinically Eligible and Clinically Evaluable Analysis Set 23
   10.4 Analyzable Sets 24
   10.5 Cancer Signal Detected Analysis Sets 24
   10.6 No Cancer Signal Detected Analysis Sets 24

CONFIDENTIALITY

This document is the confidential and proprietary information of GRAIL, LLC. Except as required by federal, state, or local laws or regulations, this document may not be disclosed to others, in whole or in part without prior written permission of GRAIL, Inc.

11. Study Analyses 24
11.1 PMA Analysis 26
11.2 First Post PMA Analysis 26
11.3 Additional Post PMA Analyses 26
11.4 IDMC Analyses 26
12. Multiplicity and Multiple Hypothesis Testing 27
13. Study Cohort Characteristics and Participant Disposition 27
13.1 General Considerations 27
13.2 Enrollment 27
13.3 Demographics and Baseline Characteristics 27
13.4 Participant Disposition 29
14. Statistical Analysis Methods 30
14.1 General Considerations 30
14.1.1 Study Objectives and Analysis Sets 30
14.1.2 Subgroup Considerations and Confidence Intervals 31
14.1.3 Cancer Status Assessments and Loss to Follow-Up 32
14.1.4 Weighted Analyses for MCED-I 32
14.1.5 Analysis Time Window for PRO Questionnaires 33
14.2 Analysis Methods for Primary Objectives 33
14.2.1. Primary Objective One (Safety) 33
14.2.1.1 Safety Endpoints 33
14.2.1.2 Analysis Methods for Safety Analyses of MCED-V2 Test 34
14.2.1.3 Analysis Methods for Safety Analyses of MCED-I Test 36
14.2.2. Primary Objective Two (Test Performance) 39
14.2.2.1 Test Performance Endpoints 39
14.2.2.1.1 Test Performance Endpoints for Cancer Signal Detection 39
14.2.2.1.2 Test Performance Endpoints for CSO Prediction 42
14.2.2.1.3 Exploratory Test Performance Endpoints for Combined Cancer Signal Detection and CSO Prediction 43
14.2.2.1.4 Analyses by Stage 44
14.2.2.1.5 Concordance of MCED-V2 Test and MCED-I Test Results 45
14.2.2.1.6 Descriptive Summaries 46
14.2.2.2 Acceptance Criteria for PMA Analysis 47
14.2.2.3 Evaluation of MCED-V2 Test Performance 49
14.2.2.3.1 Analysis at the time of diagnostic resolution time point 49
14.2.2.3.2 Analysis at 12 months follow-up time point 50
14.2.2.3.3 Analysis at 24 months time point 50

CONFIDENTIALITY
This document is the confidential and proprietary information of GRAIL, LLC. Except as required by federal, state, or local laws or regulations, this document may not be disclosed to others, in whole or in part without prior written permission of GRAIL, Inc.

14.2.2.4 Evaluation of MCED-I Test Performance 50
14.2.2.4.1 Analysis at 12 months follow-up time point 50
14.2.2.4.2 Analysis at 24 months time point 51
14.3 Analysis Methods for Secondary Objectives 51
14.3.1 Participant reported outcomes and behavioral intentions 51
14.3.1.1 Evaluate intention and utilization of guideline recommended cancer screening procedures after use of the MCED test 51
14.3.1.2 Assess Participants Reported Anxiety Resulting from Use of the MCED Test 54
14.3.1.3 Assess participants' reported outcomes and perceptions about the MCED test 55
14.3.1.3.1 Adapted MICRA 55
14.3.1.3.2 SF-12v2 55
14.3.1.3.3 Satisfaction with MCED Test 55
14.3.1.3.4 Attitude Towards Subsequent MCED Test 56
14.3.2 Diagnostic evaluations 56
14.3.2.1 Characterize the number and type of diagnostic evaluations by predicted cancer signal origin and outcome of diagnostic resolution 56
14.3.2.2. Evaluate the radiation exposure due to the test result-directed evaluation 58
14.3.2.2.1 Evaluating radiation exposure for MCED-V2 test positive participants 58
14.3.2.2.2 Evaluate radiation exposure for MCED-I test positive participants 58
14.3.2.3 Describe the ability of diagnostic PET-CT to confirm cancer in participants where diagnostic resolution of cancer diagnosis is not achieved through the targeted diagnostic testing triggered by MCED test results 58
14.3.3 Test performance 59
14.3.3.1 Evaluate Performance of the MCED Test in Selected Demographic and Clinical Subgroups. 59
14.3.3.2 Evaluate MCED Test Positive Rate in Subgroups with Potential Cross-Reactive Conditions 60
14.3.3.3 Evaluate concordance of initial GRAIL test result and CSO prediction, with results from the research blood draw 61
14.4 Analysis Methods for Exploratory Objectives 62
14.4.1 Evaluate performance of the MCED test over three years of follow-up. 62
14.4.2 Evaluate changes in blood-based biomarkers among the subset of participants with a research blood draw. 62
14.4.3 Evaluate different CSO reporting classes based on clinically relevant subgroups. 62
14.4.4 Evaluate DNA based and other biomarkers in tissue compared to blood from

CONFIDENTIALITY
This document is the confidential and proprietary information of GRAIL, LLC. Except as required by federal, state, or local laws or regulations, this document may not be disclosed to others, in whole or in part without prior written permission of GRAIL, Inc.

cancer cases. 62
15. Analysis Methods for Adverse Events 62
16. Protocol Deviations 63
17. Sample Size and Power 63
Appendix 1 PATHFINDER 2 Inclusion/Exclusion Criteria 66
Appendix 2 Participant-reported outcome data collection time points 67
Appendix 3 CSO predictions for the MCED-V2 test 68
Appendix 4 Provisional CSO predictions for the MCED-I test 69
Appendix 5 MCED-I and MCED-V2 CSO Predictions 70
Appendix 6 MCED-I CSO Specification 73
Appendix 7 Prespecified Cancer Subgroups 78
Appendix 8 Cancer Type Specifications 80
Appendix 9 Sample Size and Power for Bridging Analysis, Stratification Factors, Approximate Sampling and Analysis Weights 84
Appendix 10 Examples of invasive procedures 87
Appendix 11 Recommended diagnostic evaluations by CSO prediction 88
Appendix 12 USPSTF cancer screening recommendations 91
Appendix 13 Description of Participant-Directed Questionnaires. 92
Appendix 14 Estimated effective radiation dose estimates for imaging tests. 95

CONFIDENTIALITY
This document is the confidential and proprietary information of GRAIL, LLC. Except as required by federal, state, or local laws or regulations, this document may not be disclosed to others, in whole or in part without prior written permission of GRAIL, Inc.

1. Definitions and Abbreviations

Term	Definition
AE	Adverse event
AJCC	American Joint Committee on Cancer
BMI	body mass index
CSO	cancer signal origin
CCGA	Circulating Cell-free Genome Atlas (NCT02889978)
cfDNA	cell-free deoxyribonucleic acid
CI	confidence interval
CSR	clinical study report
CUP	cancer of unknown primary
eCRF	electronic case report form
EDC	electronic data capture
EMR	electronic medical record
FN	false negative
FP	false positive
ICD-O	International Classification of Diseases for Oncology
IDE	investigational device exemption
IDMC	Independent Data Monitoring Committee
LDCT	low-dose computed tomography
MICRA	Multidimensional Impact of Cancer Risk Assessment
MCED	multi-cancer early detection
NCCSO	No clinical cancer signal origin assigned
NCCN	National Comprehensive Cancer Network

CONFIDENTIALITY
This document is the confidential and proprietary information of GRAIL, LLC. Except as required by federal, state, or local laws or regulations, this document may not be disclosed to others, in whole or in part without prior written permission of GRAIL, Inc.

NGS	next generation sequencing
NNTS	number needed to screen
NPV	negative predictive value
PET-CT	positron emission tomography-computed tomography scan
PMA	premarket application
PPV	positive predictive value
PRO	participant reported outcomes
PSA	prostate-specific antigen
SAE	serious adverse event
SAP	statistical analysis plan
SF-12	short form health survey
SOC	standard of care
STAI	State-Trait Anxiety Inventory
TNM	tumor, lymph node, metastasis description for cancer staging
TN	true negative
TP	true positive
USPSTF	United States Preventive Services Task Force
WHO	World Health Organization

CONFIDENTIALITY
This document is the confidential and proprietary information of GRAIL, LLC. Except as required by federal, state, or local laws or regulations, this document may not be disclosed to others, in whole or in part without prior written permission of GRAIL, Inc.

2. Background and Rationale

This statistical analysis plan (SAP) describes the statistical methods to be used in the analysis of GRAIL-012 The PATHFINDER 2 Study: Evaluating the Safety and Performance of the GRAIL Multi-Cancer Early Detection Test in an Eligible Screening Population. The study is conducted under an IDE (G210176) and is registered at clinicaltrials.gov (NCT05155605).

PATHFINDER 2 is a prospective, interventional multi-center study of the GRAIL MCED test in healthcare systems in North America in which approximately 35,000 participants aged 50 years or older are expected to be enrolled. The purpose is to evaluate the safety and performance of the GRAIL MCED test in an eligible screening population. Safety for the MCED test will be evaluated during the study in terms of diagnostic procedures performed during the diagnostic journey, including imaging, biopsy, endoscopy, and surgery, in participants with positive MCED test results and adverse events reported for all study participants.

The GRAIL MCED test version 2.0 (henceforth referred to as MCED-V2) will be ordered by and results returned to a study investigator. In cases with a “cancer signal detected” test result (henceforth referred to interchangeably as “positive” or “cancer signal detected” result), the diagnostic work-up will be coordinated by the study medical team at the enrolling sites. In cases with a “no cancer signal detected” test result (henceforth referred to interchangeably as “negative” or “no cancer signal detected” result), participants will be instructed not to interpret negative test result as the absence of cancer. All participants are encouraged to continue to adhere to guideline-recommended cancer screening tests. Study participants will be followed for 3 years after enrollment with annual assessments of cancer status and utilization of guideline-recommended (routine) cancer screening tests.

A subset of blood samples collected from PATHFINDER 2 participants will be analyzed retrospectively with the final to-be-marketed version of the GRAIL MCED test (version 3.0, henceforth referred to as MCED-I) in order to demonstrate concordance between the two versions of the test. The sampling scheme and rationale for the sample size of the subset selected for MCED-I analysis are included in this document.

This SAP will describe the methods for the following analyses:

1. Safety and test performance for MCED-V2
2. Safety and test performance for MCED-I
3. Concordance between MCED-V2 and MCED-I

3. Study Objectives

3.1 Primary Objectives

There are two primary study objectives:

1. Evaluate the safety of the MCED test in terms of diagnostic testing triggered by the MCED test result (Section 14.2.1).

   Safety for this objective will be evaluated in terms of the number and type of invasive procedures (Appendix 10) and the number and type of adverse events reported during

CONFIDENTIALITY
This document is the confidential and proprietary information of GRAIL, LLC. Except as required by federal, state, or local laws or regulations, this document may not be disclosed to others, in whole or in part without prior written permission of GRAIL, Inc.

diagnostic testing in participants with a positive MCED test result.

2. Evaluate performance of the MCED test in individuals eligible for cancer screening (Section 14.2.2).

   As part of the second primary objective, the acceptance criteria for PMA analysis will be evaluated for MCED-I test based on the 12 months follow-up time analysis period using hierarchical testing. For each criterion, the passing threshold represents the target which the lower bound of the two-sided 95% confidence interval needs to exceed.

   - First, two co-primary endpoints of episode sensitivity and specificity will be tested to demonstrate that episode sensitivity for cancers responsible for ⅔ of all cancer deaths in the US (as defined in Appendix 7) is significantly higher than 22% and that specificity is significantly higher than 99%. To claim success on the primary acceptance criteria, statistical significance (with one sided p-value < 0.025) needs to be achieved for both of the co-primary endpoints.

   - Second, conditional on the success of the co-primary endpoints, episode sensitivity for cancers without common screening options (as defined in Appendix 7) will be tested to demonstrate that it is significantly higher than 19% with one sided p-value < 0.025.

   - Third, conditional on the success of all the previous endpoints, episode sensitivity for all cancers will be tested to demonstrate that it is significantly higher than 10% with one sided p-value < 0.025.

   - Subsequently, conditional on the success of all the previous endpoints, accuracy of CSO prediction in true positives will be tested to demonstrate that it is significantly higher than 70% with one sided p-value < 0.025

   The hierarchical testing sequence for MCED-I test acceptance criteria is illustrated in Figure 1 below.

CONFIDENTIALITY
This document is the confidential and proprietary information of GRAIL, LLC. Except as required by federal, state, or local laws or regulations, this document may not be disclosed to others, in whole or in part without prior written permission of GRAIL, Inc.

Figure 1. MCED-I test acceptance criteria testing sequence

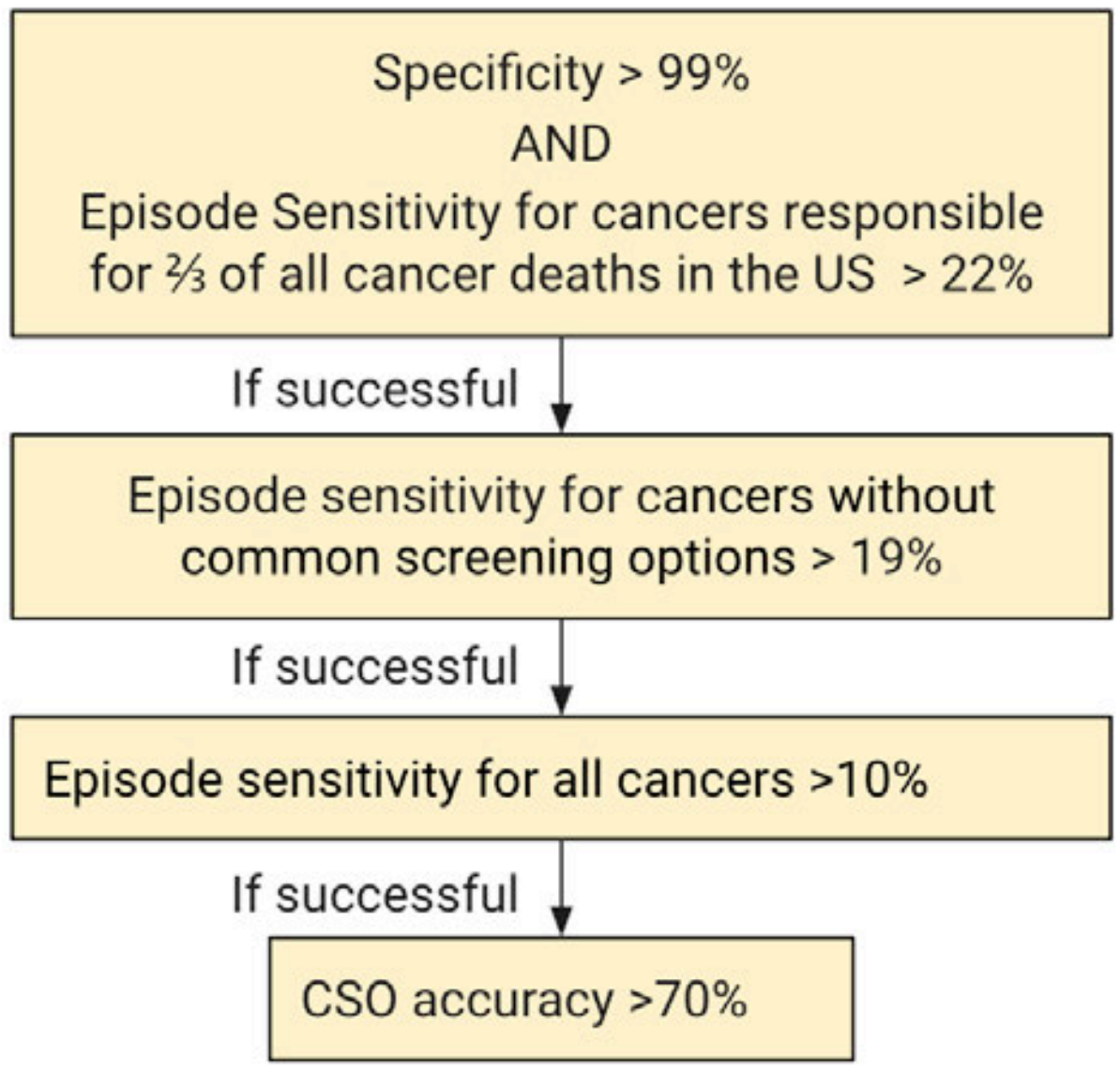

In addition, a set of clinically relevant cancer and CSO-specific performance endpoints will be provided descriptively for evaluation of MCED-I test performance.

3.2 Secondary Objectives

The secondary objectives of the study fall into three categories: participant reported outcomes and behavioral intentions, diagnostic evaluations, and test performance, as follows:

1. Participant reported outcomes and behavioral intentions

   1.1. Evaluate intention and utilization of guideline recommended cancer screening procedures after use of the MCED test (Section 14.3.1.1) (study protocol secondary objective 2).

   1.2. Assess participant-reported anxiety resulting from use of the MCED test (Section 14.3.1.2) (study protocol secondary objective 1).

   1.3. Assess participants' reported outcomes and perceptions about the MCED test (Section 14.3.1.3) (study protocol secondary objective 7).

2. Diagnostic evaluations

   2.1. Characterize the number and type of diagnostic evaluations by predicted cancer signal origin and outcome of diagnostic resolution (Section 14.3.2.1) (study protocol secondary objective 4).

   2.2. Evaluate the radiation exposure during diagnostic evaluation by imaging tests to inform safety (Section 14.3.2.2) (study protocol secondary objective 5).

   2.3. Describe the ability of diagnostic PET-CT to confirm cancer in participants where

CONFIDENTIALITY
This document is the confidential and proprietary information of GRAIL, LLC. Except as required by federal, state, or local laws or regulations, this document may not be disclosed to others, in whole or in part without prior written permission of GRAIL, Inc.

diagnostic resolution of cancer diagnosis is not achieved through the targeted diagnostic testing triggered by MCED test results (Section 14.3.2.3) (study protocol secondary objective 3).

3. Test performance

   3.1. Evaluate performance of the MCED test in selected demographic and clinical subgroups (Section 14.3.3.1) (secondary objective not included in the study protocol).

   3.2. Evaluate MCED test positive rate in subgroups with potential cross-reactive conditions (Section 14.3.3.2) (secondary objective not included in the study protocol)

   3.3. Evaluate concordance of initial GRAIL test result and Cancer Signal Origin (CSO) prediction, with results from the research blood draw (Section 14.3.3.3) (study protocol secondary objective 6).

3.3 Exploratory Objectives

The exploratory objectives of the study include:

1. Evaluate performance of the MCED test over three years of follow-up
2. Evaluate changes in blood based biomarkers among the subset of participants with a research blood draw
3. Evaluate different CSO reporting classes based on clinically relevant subgroups
4. Evaluate DNA based and other biomarkers in tissue compared to blood from cancer cases.

4. Study Design

4.1 PATHFINDER 2 Study Design

This section briefly describes the design of the PATHFINDER 2 study. More details can be found in the study protocol.

PATHFINDER 2 is a prospective, interventional multi-center study of the GRAIL MCED test in which approximately 35,000 participants aged 50 years or older (inclusion/ exclusion criteria in Appendix 1) are expected to be enrolled at clinical sites in North America. The MCED test will be ordered by, and results returned to, a study investigator. In cases with a positive test result, the diagnostic work-up will be coordinated by the study medical team at the enrolling sites. Up to two cancer signal origin (CSO) predictions may be returned. The healthcare providers are strongly encouraged to evaluate participants for cancer based on the organ or anatomical location described by first CSO prediction using the CSO-based diagnostic evaluations described in the study protocol. If no cancer is identified based on the first CSO, the healthcare provider should evaluate the participant for the second CSO, if it was reported.

CONFIDENTIALITY
This document is the confidential and proprietary information of GRAIL, LLC. Except as required by federal, state, or local laws or regulations, this document may not be disclosed to others, in whole or in part without prior written permission of GRAIL, Inc.

For participants where diagnostic resolution of cancer diagnosis is not achieved through the CSO-guided diagnostic testing and PET-CT has not been used as part of diagnostic evaluation, a full body (skull vertex to mid-thighs) diagnostic PET-CT will be performed to assess cancer status (referred to the 'confirmatory PET-CT' in the study protocol). Participants in this group will also undergo a research blood draw around the time of this diagnostic PET-CT. Results of this research blood draw will not be provided back to the physician or participant.

If a participant is unable to undergo the recommended diagnostic evaluations or the healthcare provider determines an alternative or additional test is necessary, the healthcare provider will document the reason for deviating from the recommended evaluation(s) and list the alternative and additional test(s) performed. In cases with a negative test result, participants are instructed not to interpret a negative test result as the absence of cancer and to continue to adhere to guideline-recommended cancer screening.

Safety for the MCED test will be evaluated during the study in terms of diagnostic procedures performed in participants with positive MCED test results and adverse events reported for all study participants.

Additionally, participant-reported outcomes (PRO) will be collected at several time points (Appendix 2) to assess participants' perceptions about the MCED test, including anxiety, health-related quality of life, and the potential impact of test results on attitude towards adherence to guideline-recommended screening.

Participants will be followed for three (3) years from the time of enrollment, and cancer status and utilization of guideline-recommended cancer screening will be assessed annually.

All study procedures were approved by a central or local Institutional Review Board.

4.2 Bridging Analysis for MCED-I Test

In the PATHFINDER 2 study, results of the MCED-V2 test are returned to healthcare providers, and will guide diagnostic evaluation for cancer in participants MCED-V2 test positive results. In addition, the MCED-I test will be performed on the frozen plasma samples from a subset of PATHFINDER 2 participants at a time that is at least 12 months after the date of the blood draw. This will enable evaluation of performance and safety of the MCED-I test in the PATHFINDER 2 study.

A sampling scheme will be implemented so that a subset of samples will be tested with the MCED-I test in order to ensure that the analysis results are representative of the overall study population. Plasma samples (if available) from the following groups of participants will be selected:

- All participants with positive MCED-V2 test results
- All participants with cancer diagnosis during 12 months of follow-up time
- Samples from a subset of participants with negative MCED-V2 test results and no cancer diagnosis during 12 months of follow-up time.

The subset selection of participants with negative MCED-V2 test results and no cancer diagnosis during 12 months of follow-up time will be stratified on the following factors (see Appendix 9 for

CONFIDENTIALITY
This document is the confidential and proprietary information of GRAIL, LLC. Except as required by federal, state, or local laws or regulations, this document may not be disclosed to others, in whole or in part without prior written permission of GRAIL, Inc.

details):

- Age
- Race
- Ethnicity
- Prior cancer history
- Smoking status
- Selected conditions that may affect cfDNA shedding or methylation such as inflammatory conditions (e.g. pancreatitis), use of demethylating agents or prior transplant

Stratification factors were chosen to provide sufficient representation of subsets of interest, especially relatively small subsets of population important to characterize:

- Diversity and representativeness in terms of race and ethnicity
- MCED test performance in terms of false positive rate (FPR)

The detailed description of sampling strata, approximate size of the strata and sampling weights are summarized in Appendix 9.

5. Study Population

The study will enroll approximately 35,000 participants aged 50 years or older as described in the inclusion/exclusion criteria (see Appendix 1). The goal is to enroll a diverse participant population, generally representative of the US population aged 50 years and older with respect to race, ethnicity and sex. Target distributions for race, ethnicity, age and sex are listed in the study protocol (Section 6, Table 5).

If inclusion/exclusion criteria are modified through a protocol amendment, the latest inclusion/exclusion criteria available during preparation for analysis will be utilized for the main analysis. If changes to these criteria are substantial, an exploratory analysis may be conducted with earlier inclusion/exclusion criteria. SAP Appendix 1 will specify which inclusion/exclusion criteria will be used in the main analysis.

6. Data

This section briefly describes the types of data collected in the PATHFINDER 2 study. More details can be found in the PATHFINDER 2 study protocol.

6.1 Data Sources

The main sources of data in PATHFINDER 2 study include the following:

- Laboratory data generated from the MCED test
    - MCED-V2 test result
    - MCED-I test result

CONFIDENTIALITY
This document is the confidential and proprietary information of GRAIL, LLC. Except as required by federal, state, or local laws or regulations, this document may not be disclosed to others, in whole or in part without prior written permission of GRAIL, Inc.

- Clinical data entered into and stored in the electronic case report forms (eCRF) within Medrio, a validated 21CFR Part 11 compliant electronic data capture system (EDC). These data are collected from:
    - Blood collection tube label
    - Test Requisition Form
    - Test Result Report
    - Participant electronic medical records (EMR)
    - Self-reported participant information for baseline study questionnaires
    - PRO questionnaires administered either online or by paper questionnaires, depending on participant ability to access PROs online and preference.

A more comprehensive description can be found in the PATHFINDER 2 study protocol, Clinical Data Management Plan, and eCRF Specifications.

6.2 Clinical Data

The clinical data will include participant related fields, demographics and participant information, clinical information, information about imaging, laboratory tests and invasive procedures, and tumor-related information. Examples of key clinical and demographic variables include:

- Demographic and Participant Information: age, sex, race/ethnicity, personal and family history of cancer, smoking status, alcohol use, body mass index (BMI), cancer screening history.
- Clinical Information: type and number of diagnostic tests and procedures, outcome of diagnostic resolution, cancer status at 12, 24 and 36 months follow-up.
- Tumor Information: cancer type, clinical stage.

Tumor stage will be defined based on the clinical stage reported by clinical sites. Sites are instructed to report stage according to the staging system that is relevant for the type of cancer that is diagnosed.

PRO data include ratings for the following questionnaires (see details in Appendix 13) collected at various time points during the study (as described in Appendix 2):

- State Trait Anxiety Inventory (STAI) questionnaire and MCED STAI questionnaires
- Adapted Multidimensional Impact of Cancer Risk Assessment (MICRA)
- The 12-Item Short Form Health Survey version 2 (SF-12v2)
- Satisfaction with the MCED test
- Attitude towards adherence to guideline-recommended screening
- Attitude towards subsequent MCED test

Based on the collected information, we will derive and document the logic for defining variables in the dataset specifications per the GRAIL Analysis Variable Specifications Process standard operating procedure (SOP).

CONFIDENTIALITY
This document is the confidential and proprietary information of GRAIL, LLC. Except as required by federal, state, or local laws or regulations, this document may not be disclosed to others, in whole or in part without prior written permission of GRAIL, Inc.

6.3 MCED Test Data

GRAIL's MCED test is a targeted methylation, next-generation sequencing (NGS)-based assay that analyzes cell-free DNA (cfDNA) isolated from peripheral blood. The test measures the extent and location of DNA methylation in the genome by sequencing a subset of genomic regions, and applies a computational algorithm to determine whether or not a shared cancer signal is present, regardless of cancer type. If a shared cancer signal is detected, the algorithm then makes a prediction of the origin(s) of the detected signal. CSO predictions are based on observed methylation patterns consistent with the biologic origins of a cancer, and are the result of the classifier, bioinformatics pipeline and medical device software. CSO predictions are intended to inform the workup of a positive MCED test result.

Source data for sample processing will be curated and managed as described in the GRAIL Sample and Data Analysis Workflow SOP.

The MCED-V2 Test data will be generated from processing one plasma tube for each participant. In case the assay fails, a back up tube may be used to rerun the assay. The MCED-V2 test results are returned to the study investigator. A similar process will be used to generate MCED-I test data.

All elements of the V2 assay including assay processing method, sample quality criteria, classifier and CSO reporting, were locked prior to sample processing. In addition, staff involved in assay result generation are blinded and do not have access to clinical data except for the limited information collected on the test requisition form (pregnancy, solid organ transplant and bone marrow transplant status) and/or used for generation of assay results (age (based on date of birth), sex) until the test report sign-off for release to study investigator.

All elements of the MCED-I test including assay processing method, sample quality criteria, classifier and signal origin reporting, will be locked prior to PATHFINDER 2 samples being processed with MCED-I test. The MCED-I classifier was locked after the start of the PATHFINDER 2 study. To ensure that MCED-I developers are not exposed to the MCED-V2 PATHFINDER 2 data, a study-specific Blinding Plan was developed according to the Clinical Study Data Blinding SOP.

The MCED-V2 test data were generated from processing sample data (refer to related Multi-Cancer Test V2 Automated Process and Multi-Cancer Test Sequencing documents for details) and through the bioinformatics analysis (refer to RES-DEV-0017, *Multicancer V2 Bioinformatics Pipeline Architecture and Design Specification* and related Multi-Cancer V2 Targeted Methylation Pipeline documents for details).

6.4 Data Lock Process and Merge

MCED test data will undergo a quality control (QC) process. The data will need to pass the predefined sample QC criteria as documented in the GRAIL Multi-Cancer Test V2 Quality Specification and GRAIL Multi-Cancer Test V3 (MCED-I) Quality Specification.

CONFIDENTIALITY
This document is the confidential and proprietary information of GRAIL, LLC. Except as required by federal, state, or local laws or regulations, this document may not be disclosed to others, in whole or in part without prior written permission of GRAIL, Inc.

Likewise, before being released for analysis, clinical data will undergo the cleaning and clinical data lock process including cross-functional approval of the clinical data approval form and export of the EDC data for analysis, as described in *CDM-SOP-0004, Clinical Data Lock Process*, the study-specific Clinical Data Lock Plan and the PATHFINDER 2 Clinical Data Management Plan. Site staff will be permitted to make changes to clinical data after each specified analysis. If data updates occur, additional review and Principal Investigator signature are required as part of the clinical data lock process prior to the inclusion of the data in a subsequent analysis. The eCRFs will be locked to remove edit access at the time of the end-of-study analysis.

After clinical data are cleaned and/or locked, the data will be deposited into TidyData, a repository of GRAIL MCED test data and clinical data with system infrastructure to assemble the data, maintain versions of the data, and manage access to the data, where it will be merged for analysis purposes. The PATHFINDER 2 TidyData Plan describes the flow of merging the GRAIL MCED test data and clinical data into the TidyData and its contents along with other features of TidyData.

Releasing the TidyData with merged GRAIL Multi-Cancer MCED-V2 test data, MCED-I test data and clinical data will be considered as unblinding to MCED-V2 and MCED-I test results and will follow the GRAIL Clinical Study Data Blinding SOP.

7. Measures to Minimize Bias

In this prospective longitudinal study, information related to demographics, clinical characteristics, diagnostic procedure types, counts, dates, and cancer diagnoses will be captured from the EMR, which will minimize most biases (e.g., recall bias, observer bias, missing data bias).

To assess bias in enrollment, GRAIL will monitor and continuously compare distributions of key demographic and clinical characteristics of the enrolled population to the target population. In particular, if there is disproportionately high enrollment of a particular stratum (based on age and sex), further enrollment of individuals in this stratum may be limited.

To minimize conscious and unconscious bias, test developers and SAP developers were blinded to the study data according to the PATHFINDER 2 Data Access and Blinding Plan.

8. Outcome Definitions

8.1 GRAIL MCED Test Result

The outcome of GRAIL's MCED test (irrespective of version) includes:

- A binary result of "Cancer signal detected" or "No cancer signal detected"
    - The terms "Cancer signal detected" and "positive" MCED test result are used interchangeably, and
    - The terms "No Cancer Signal Detected' and "negative" MCED test result are used interchangeably in this document.
- Cancer signal origin (CSO) prediction(s) for a positive test result

CSO predictions come from a predefined list of CSOs. Appendices 3 and 4 contain the full lists of CSOs for the MCED-V2 test and MCED-I test, respectively. CSO specifications in Appendix 6

CONFIDENTIALITY
This document is the confidential and proprietary information of GRAIL, LLC. Except as required by federal, state, or local laws or regulations, this document may not be disclosed to others, in whole or in part without prior written permission of GRAIL, Inc.

describe which cancers are associated with each MCED-I CSO prediction. This allows the accuracy of the CSO predictions to be rigorously assessed against the clinical diagnosis of cancer in the study (clinical CSO).

The MCED-V2 test provides up to two CSO predictions to inform diagnostic workup. If there is a strong match between the observed methylation patterns and a CSO, then one CSO prediction is returned. Otherwise the two most likely CSO predictions are returned. In this SAP, CSO will be described as follows:

- The first CSO prediction is referred to as CSO1
- The second CSO prediction is referred to as CSO2 (if more than one CSO is returned by the MCED-V2 test)

The MCED-I test will provide one CSO prediction, which will be referred to as CSO1.

8.2 Target Clinical Outcome

8.2.1 Cancer

The target clinical outcome of cancer in the study is defined as any of the following cancers diagnosed during the study period:

- Invasive solid tumors, excluding non-metastatic basal cell carcinoma and squamous cell carcinoma of the skin

- Hematologic malignancies:
    - Lymphoma
    - Lymphoid leukemia
    - Plasma cell neoplasm/Multiple myeloma
    - Myeloid neoplasm
    - Additional malignant hematologic conditions representing proliferative or dysplastic disorders or neoplasms with behavior code 3 based on ICD-O-3

Note that ICD-O-3 is used as a reference system for what is considered to be a hematologic malignancy. Documentation of the ICD-O-3 behavior code is not required to establish a diagnosis of cancer.

Cancer diagnosis is supported by pathologic confirmation of an invasive solid tumor or hematologic malignancy, or by imaging confirmation in the absence of pathology, or by assigned clinical stage of I-IV by the treating physician, or by other clinical confirmation of disease per treating clinician (e.g. biochemical evidence of recurrence of prior cancer).

Cancer status for a specific analysis period is determined based on whether or not a participant has at least one confirmed clinical outcome of cancer (cancer participant vs non-cancer participant) in the specific analysis period (as defined in Section 11).

CONFIDENTIALITY
This document is the confidential and proprietary information of GRAIL, LLC. Except as required by federal, state, or local laws or regulations, this document may not be disclosed to others, in whole or in part without prior written permission of GRAIL, Inc.

8.2.2 Cancer Clinical Stage

A cancer is considered stageable if a recognized staging system exists for its histology (morphology) with or without anatomic site (topography). The "stageable cancer" will be used in descriptive summaries of cancer type/ stage distribution, as well as performance metrics by cancer type/ stage.

Clinical stage is determined by the healthcare providers through clinical information, pathology reports and/or imaging findings. Cancers that do not have a tumor, node, metastasis (TNM)-type staging system defined in the AJCC cancer staging manual[1], such as cancers of the brain and spinal cord, lymphoid leukemias, myeloid neoplasms and additional malignant hematologic conditions, will be analyzed based on the clinical stage provided by the study site using the relevant staging system for each condition, if applicable.

For recurrent cancers, the extent of recurrence is documented as local, lymph nodes (regional) or distant (metastatic).

8.2.3 Cancer Type

For each observed clinical cancer diagnosis, a cancer type is assigned. Cancer types are listed in Appendix 8. Cancers that are not included in the defined cancer types are reported under the "Other" category. Cancer type is a clinical construct for staging, prognosis and treatment, and is used in the context of data analysis and results presentation.

Participants who do not have a cancer type assigned will be considered to have "Unassigned" cancer type.

8.2.4 Clinical CSO

For each observed cancer diagnosis, a clinical CSO is assigned based on histologic type (morphology), anatomic site (topography) and behavior code according to ICD-O-3. The Clinical CSO represents the clinical truth for the methylation based CSO prediction and is used to evaluate whether an MCED CSO prediction was accurate. CSO definitions relevant to CSO predictions and clinical CSOs are documented in the CSO specification (Appendix 6). Note that any cancer that is not reflected in the CSO specification definitions will not have a clinical CSO assigned. Only participants who have a clinical CSO assigned will be included in the analysis evaluating accuracy of CSO prediction.

Participants who do not have a clinical CSO assigned will be considered to have "Unassigned" clinical CSO.

8.2.5 Cancer of Unknown Primary

A cancer of unknown primary (CUP) will not be assigned a clinical CSO. A CUP diagnosed during the specific analysis period will be considered for analysis. CUPs are unstageable; if the cancer

---

[1] Amin MB, Edge S, Greene F, Byrd DR, Brookland RK, Washington MK, Gershenwald JE, Compton CC, Hess KR, et al. (Eds.). AJCC Cancer Staging Manual (8th edition). Springer International Publishing: American Joint Commission on Cancer; 2017.

CONFIDENTIALITY

This document is the confidential and proprietary information of GRAIL, LLC. Except as required by federal, state, or local laws or regulations, this document may not be disclosed to others, in whole or in part without prior written permission of GRAIL, Inc.

type is unknown then it cannot be staged under the appropriate system. Specific considerations with respect to CUP will be discussed at the definition of each endpoint.

8.2.6 Multiple Cancers

Participants with more than one cancer diagnosed during the analysis period are considered to have multiple cancers, irrespective of whether these were recurrent or new primary cancers. For example, participants with a prior cancer history who had both a recurrent cancer and a new subsequent primary cancer diagnosed during the analysis period will be included in the multiple cancers group. Participants with a prior cancer history who are diagnosed with a new subsequent primary cancer during the analysis period and who do not have any additional cancer diagnoses (i.e., no recurrences) will not be included in the multiple cancers group for the purposes of this analysis.

Participants with multiple cancers will have multiple clinical CSOs.

8.2.7 Definition of Cancer Participant for Specific Analysis

A cancer participant is defined as a participant for whom a cancer diagnosis (e.g., at least one clinical outcome of "cancer" as defined in section 8.2.1) has been confirmed within a specific analysis period (e.g. 12 months follow-up time analysis period for PMA analysis).

When test performance endpoints are summarized by cancer type, participants with multiple cancers, with CUP or with unassigned cancer type are treated as separate cancer types, namely 'Multiple Cancers', 'Cancer of Unknown Primary' and 'Unassigned', besides the cancer types defined in Appendix 8. In order to avoid double counting, participants for whom there were multiple cancer diagnoses where at least one of them was CUP or unassigned are included in the 'Multiple Cancers" category, for reporting purposes.

8.2.8 Definition of Non-cancer Participant for Specific Analysis

The clinical outcome of non-cancer is defined as absence of the diagnosis of cancer as described in section 8.2.1. Absence of cancer diagnosis is determined at several time points for participants with MCED-V2 positive test results: at the time of the diagnostic resolution and at annual assessments. For participants with MCED-I negative test results, absence of cancer diagnosis is determined at the annual assessments only.

Non-cancer status in participants with MCED-V2 negative test results is determined by review of the medical records at approximately 12, 24, and 36 months after the blood draw for evidence of cancer diagnosis in the participant's EMR or participant self-report through a phone call. See the study protocol for additional details of the follow-up process.

Thus a non-cancer participant is defined as a participant for whom an absence of cancer diagnosis is determined at the end of the specific analysis period (e.g. 12 months follow-up time analysis period for PMA analysis).

CONFIDENTIALITY
This document is the confidential and proprietary information of GRAIL, LLC. Except as required by federal, state, or local laws or regulations, this document may not be disclosed to others, in whole or in part without prior written permission of GRAIL, Inc.

9. Handling of Dropouts or Missing Data

We expect the key demographic and baseline clinical characteristics that will be used to describe the cohort (e.g., age, race/ethnicity, sex) to be available for the majority of the participants (<5% missing data expected). The number of participants with missing clinical characteristics will be provided.

Cancer staging is determined by study sites through clinical information, pathology reports and/or imaging findings (<5% missing data expected for cancers which have an AJCC TNM-type staging system). We will report the number of cancer participants with missing clinical stages.

Approximately 5% of non-cancer participants in this study are expected not to have a 12-month cancer status assessment. A similar proportion (5%) of the remaining participants is expected not to complete 24-month cancer assessment and 36-month cancer assessment, respectively. This loss to follow-up is estimated based on the previously conducted similar study[2].

As described in Section 14, only complete case analysis, i.e. analysis of participants with completed cancer status assessment at the end of the analysis period, will be used to evaluate specificity.

Sensitivity analyses that include participants lost to follow-up will be conducted for measures such as time to diagnostic resolution and PPV.

10. Analysis Sets

Analysis sets define the participants to be included in an analysis. Analysis sets (including subsets for specific analyses) and their definitions are provided in this section.

10.1 Consented Analysis Set

This set comprises all participants who consented to take part in the study. The summaries specified in Section 13.4 (Participant Disposition) will be based on consented participants.

10.2 Clinically Eligible and Clinically Evaluable Analysis Set

This set comprises consented participants who are clinically eligible (satisfy the inclusion and exclusion criteria) and are clinically evaluable.

First, participants who satisfy study inclusion/exclusion criteria (Appendix 1) are determined. During the data cleaning process, it is possible that some consented participants who did not satisfy study inclusion/exclusion criteria are identified. For example, a study site may determine that a study participant had a history of invasive cancer less than 3 years prior to study enrollment. Such participants will be considered as eligibility failures and excluded from the clinically evaluable analysis set.

[2] Schrag et. al. Blood-based tests for multicancer early detection (PATHFINDER): a prospective cohort study, Lancet, 2023 Oct 7;402(10409):1251-1260

CONFIDENTIALITY
This document is the confidential and proprietary information of GRAIL, LLC. Except as required by federal, state, or local laws or regulations, this document may not be disclosed to others, in whole or in part without prior written permission of GRAIL, Inc.

Second, consented participants who satisfy study inclusion/exclusion criteria and clinical evaluability criteria are determined. The clinical evaluability criteria are described in the PATHFINDER 2 Clinical Data Management Plan. Examples include the presence of an evaluable blood draw.

Participants who withdraw consent after the MCED-V2 test result becomes available may be eligible for inclusion in some of the analyses. See Section 15 for details.

10.4 Analyzable Sets

Participants who were in the Clinically Evaluable Analysis Set and had an evaluable MCED-V2 test result will comprise the MCED-V2 Analyzable Set.

Participants who were in the Clinically Evaluable Analysis Set and had an evaluable MCED-I test result will comprise the MCED-I Analyzable Set.

10.5 Cancer Signal Detected Analysis Sets

The MCED-V2 Cancer Signal Detected Analysis Set will be composed of MCED-V2 analyzable participants with positive MCED-V2 test results.

The MCED-I Cancer Signal Detected Analysis Set will be composed of MCED-I analyzable participants with positive MCED-I test results.

10.6 No Cancer Signal Detected Analysis Sets

The MCED-V2 No Cancer Signal Detected Analysis Set will be composed of analyzable participants with negative MCED-V2 test results.

The MCED-I No Cancer SIgnal Detected Analysis Set will be composed of analyzable participants with negative MCED-I test results.

11. Study Analyses

Several analyses are planned for the PATHFINDER 2 study: PMA analysis, first post PMA analysis, additional post-PMA analyses and IDMC analyses. Study objectives addressed in the PMA-related analyses are described in Table 1.

CONFIDENTIALITY
This document is the confidential and proprietary information of GRAIL, LLC. Except as required by federal, state, or local laws or regulations, this document may not be disclosed to others, in whole or in part without prior written permission of GRAIL, Inc.

Table 1. Study objectives in PMA-related analyses.

Objective type	N	Study objective	PMA analysis	First post PMA analysis	Additional post PMA analyses
**Primary**	1	Evaluate the safety of the MCED test in terms of diagnostic testing triggered by the MCED test result	✓	✓	
	2	Evaluate performance of the MCED Test in individuals eligible for cancer screening	✓	✓	
**Secondary**	1	**Participant reported outcomes and behavioral intentions**			
	1.1	Evaluate intention and utilization of guideline recommended cancer screening procedures after use of the MCED test	✓	✓	✓
	1.2	Assess participant-reported anxiety resulting from use of the MCED test	✓	✓	
	1.3	Assess participants' reported outcomes and perceptions about the MCED test	✓	✓	
	2	**Diagnostic evaluations**			
	2.1	Characterize the number and type of diagnostic evaluations by predicted cancer signal origin and outcome of diagnostic resolution	✓	✓	
	2.2	Evaluate the radiation exposure during diagnostic evaluation by imaging tests to inform safety	✓	✓	
	2.3	Describe the ability of diagnostic PET-CT to confirm cancer in participants where diagnostic resolution of cancer diagnosis is not achieved through the targeted diagnostic testing triggered by MCED test results	✓	✓	
	3	**Test performance**			
	3.1	Evaluate performance of the MCED test in selected demographic and clinical subgroups	✓	✓	
	3.2	Evaluate MCED Test Positive Rate in Subgroups with Potential Cross-Reactive Conditions		✓	
	3.3	Evaluate concordance of initial GRAIL test result and Cancer Signal Origin (CSO) prediction, with results from the research blood draw	✓		

CONFIDENTIALITY

This document is the confidential and proprietary information of GRAIL, LLC. Except as required by federal, state, or local laws or regulations, this document may not be disclosed to others, in whole or in part without prior written permission of GRAIL, Inc.

Objective type	N	Study objective	PMA analysis	First post PMA analysis	Additional post PMA analyses
**Selected exploratory**	1	Evaluate performance of the MCED test in year 3.			✓

11.1 PMA Analysis

The first analysis of the PATHFINDER 2 study will take place when the majority of the consented participants will have 12 months of follow-up time after the blood draw.

This analysis is expected to include approximately 25,000 participants with 12 months of follow-up time and the remaining approximately 10,000 enrolled participants with at least 4 months of follow-up time after the blood draw. It is expected that most MCED test positive participants will finish their diagnostic evaluation in less than 6 months. Therefore, safety of the diagnostic evaluations (primary objective 1 [safety], secondary objective 2 [2.1 extent of diagnostic testing, 2.2 radiation exposure, 2.3 PET-CT]); as described in Table 1) will be summarized for 25,000 participants with 12 months of follow-up, and for 35,000 participants. Test performance (primary objective 2) and secondary objective 1 (1.1 intention and utilization of guideline recommended cancer screening, 1.2 anxiety and 1.3 PROs) as described in Table 1 will be evaluated for the 25,000 participants with 12 months of follow-up. In addition, PPV and CSO Accuracy will be assessed for MCED-V2 on 35,000 participants.

This analysis will serve as the basis for the Pre Market Application (PMA) to establish the safety and evaluate performance of the MCED-I test.

11.2 First Post PMA Analysis

The first post PMA analysis will take place when all approximately 35,000 consented participants will have 12 months of follow-up time after the blood draw. This analysis will provide additional evidence for the MCED-I test performance and safety.

11.3 Additional Post PMA Analyses

Additional analyses of the PATHFINDER 2 study will include all analyzable participants with

- 24 months of follow-up time
- 36 months of follow-up time

11.4 IDMC Analyses

An independent Data Monitoring Committee (IDMC) composed of external clinical and statistical experts will monitor the safety of the interventions in the PATHFINDER 2 study, evaluate participant recruitment and accrual and make recommendations to GRAIL regarding the

CONFIDENTIALITY
This document is the confidential and proprietary information of GRAIL, LLC. Except as required by federal, state, or local laws or regulations, this document may not be disclosed to others, in whole or in part without prior written permission of GRAIL, Inc.

continuation, modification, or termination of the study according to its charter. Several interim analyses of the PATHFINDER 2 study for the IDMC are planned when approximately 4,000, 9,000, 16,000 and 25,000 participants are enrolled. No formal evaluation of MCED test performance is planned at these interim time points. Data reviews will be scheduled based on the enrollment progress, and data for participants enrolled up to a projected date will be reviewed. Additional IDMC data reviews may be requested by the IDMC members and/or GRAIL.

For the purposes of the IDMC interim analyses described in this section, data will undergo cleaning, including cross-functional approval of the clinical data export of EDC data for analysis, as described in CDM-SOP-0004 Clinical Data Lock Process and the study-specific Clinical Data Cleaning Plan.

12. Multiplicity and Multiple Hypothesis Testing

At the time of the PMA analysis, the acceptance criteria for MCED-I test performance will be tested at the one-sided 0.025 significance level, following a hierarchical testing approach (as described in Sections 3.1 and 14.2.2) and therefore no type I error adjustment is needed.

No hypothesis testing is planned for first post PMA Analysis, additional post PMA analyses or IDMC analyses and therefore no type I error adjustment is needed.

13. Study Cohort Characteristics and Participant Disposition

13.1 General Considerations

Analysis results will be presented using descriptive statistics. For categorical (including binary) variables, the number and percentage of participants in each category will be presented. For continuous variables, the number of participants (n), mean, standard deviation (SD), median, first quartile (Q1), third quartile (Q3), minimum, and maximum will be presented.

13.2 Enrollment

Enrollment by network and site will be summarized using descriptive statistics in the enrolled participants.

13.3 Demographics and Baseline Characteristics

Participant demographics and baseline characteristics will be summarized for the following groups in the MCED-V2 Analyzable Set and MCED-I Analyzable Set, separately:

- Overall
- By MCED test result: positive vs negative

Participant characteristics will be summarized using descriptive statistics for the following variables:

1. Age (at enrollment) as a continuous variable (years)
2. Age groups (50-59, 60-69,70-79, 80+ years) and (50-64 and 65+ years)

CONFIDENTIALITY
This document is the confidential and proprietary information of GRAIL, LLC. Except as required by federal, state, or local laws or regulations, this document may not be disclosed to others, in whole or in part without prior written permission of GRAIL, Inc.

3. Biological sex (male, female)
4. Gender identity (male, female, transgender, non-binary, other, choose not to respond)
5. Sexual orientation (heterosexual, gay or lesbian, bisexual, other, choose not to respond)
6. Race categories aligned with FDA guidance[3]
    - American Indian or Alaska Native (including North American Indigenous[4]) only
    - Asian only
    - Black or African American only
    - Native Hawaiian or Other Pacific Islander only
    - White (including Middle Eastern or North African[5]) only
    - More than one race reported
7. Race categories for full representation, i.e. if reported alone or as part of multiple race categories (not mutually exclusive):
    - American Indian or Alaska Native (including North American Indigenous)
    - Asian, Native Hawaiian or Other Pacific Islander
    - Black or African American
    - White (including Middle Eastern or North African)
8. Ethnicity (Hispanic or Latino vs not Hispanic or Latino)
9. Marital status (never married, married or domestic partnership, previously married, separated, divorced, widowed)
10. Highest level of education completed (less than high school degree, high school degree or equivalent, some college, bachelor's degree or higher)
11. Smoking status (never smoker, former smoker, current smoker)
12. United States Preventive Services Task Force (USPSTF) lung cancer risk indicator (Yes, No) defined as follows:
    - Participants who met the draft updated USPSTF recommendation for low-dose computed tomography (LDCT) screening published on July 7, 2020: adults aged 50 to 80 years who have at least a 20 pack-year smoking history and currently smoke or have quit within the past 15 years, will be assigned a "Yes".
13. Alcohol use (none, light, moderate, heavy)
14. Prior history of cancer (Yes, No)
15. History of cancer in a first-degree relative (biologic parents, siblings or children) (Yes, No)
16. Presence of genetic cancer predisposition (Yes, No)
17. Body-mass index (BMI) categories (<25 kg/m^2, 25 to <30 kg/m^2, ≥30 kg/m^2)
18. Prior history of in situ cancer (Yes, No)
19. Prior history of benign hematologic condition (Yes, No)
    - Langerhans cell histiocytosis
    - Lymphoproliferative disorder
    - Mastocytoma
    - Monoclonal B-cell lymphocytosis
    - Monoclonal gammopathy of undetermined significance

---

[3] Race categories are aligned with the FDA guidance 'Collection of Race and Ethnicity Data in Clinical Trials and Clinical Studies for FDA-Regulated Medical Products', 2024

[4] This category was included specifically to accommodate a clinical site in Canada, where a different nomenclature is used in census.

[5] The US census defines white race as a person having origins in any of the original peoples of Europe, the Middle East, or North Africa.

CONFIDENTIALITY

This document is the confidential and proprietary information of GRAIL, LLC. Except as required by federal, state, or local laws or regulations, this document may not be disclosed to others, in whole or in part without prior written permission of GRAIL, Inc.

- Post-transplant lymphoproliferative disorder
- Other

20. Prior history of selected medical conditions (Yes, No)
    - Acute pancreatitis
    - Barrett's esophagus
    - Chronic gastritis
    - Chronic hepatitis B infection
    - Chronic hepatitis C infection
    - Chronic obstructive pulmonary disease (COPD), emphysema or chronic bronchitis
    - Chronic pancreatitis
    - Cirrhosis
    - Crohn's disease or ulcerative colitis
    - Diabetes
    - Esophageal reflux disease
    - Fatty liver disease
    - Human immunodeficiency virus (HIV)
    - Liver cirrhosis
    - Multiple sclerosis
    - Rheumatoid arthritis
    - Stomach ulcers
    - Systemic lupus erythematosus (lupus)
21. Use of cytotoxic medications in the past year (Yes, No)
22. Use of demethylating agents in the past year (Yes, No)
23. Use of hormone therapy in the past 90 days (Yes, No)

13.4 Participant Disposition

A summary of participant disposition will be provided for all PATHFINDER 2 participants. This summary will present the number and percentage of participants in each of the categories listed below:

- Consented Analysis Set
  - Not clinically eligible
  - Not clinically evaluable
- Clinically Evaluable Analysis Set
  - No evaluable assay results
- MCED-V2 Analyzable Set
  - MCED-V2 Cancer Signal Detected Analysis Set
  - MCED-V2 No Cancer Signal Detected Analysis Set
- MCED-I Analyzable Set
  - MCED-I Cancer Signal Detected Analysis Set
  - MCED-I No Cancer Signal Detected Analysis Set

In addition, the number of participants with and without a cancer diagnosis in the MCED-V2 Cancer Signal Detected Analysis Set, MCED-V2 No Cancer Signal Detected Analysis Set, MCED-I Cancer Signal Detected Analysis Set, and MCED-I No Cancer Signal Detected Analysis Set will be reported.

CONFIDENTIALITY
This document is the confidential and proprietary information of GRAIL, LLC. Except as required by federal, state, or local laws or regulations, this document may not be disclosed to others, in whole or in part without prior written permission of GRAIL, Inc.

14. Statistical Analysis Methods

This section will describe the statistical analysis methods for the evaluation of the PATHFINDER 2 study objectives.

14.1 General Considerations

14.1.1 Study Objectives and Analysis Sets

Table 2 describes the analysis sets used for the primary, secondary and selected exploratory study objectives.

Table 2. Study objectives and analysis sets.

Objective type	N	Study objective	Analysis Set
**Primary**	1	Evaluate the safety of the MCED test in terms of diagnostic testing triggered by the MCED test result	MCED-V2 Analyzable Set<br>MCED-I Analyzable Set
	2	Evaluate performance of the MCED Test in individuals eligible for cancer screening	MCED-V2 Analyzable Set<br>MCED-I Analyzable Set
**Secondary**	1	**Participant reported outcomes and behavioral intentions**	
	1.1	Evaluate intention and utilization of guideline recommended cancer screening procedures after use of the MCED test	MCED-V2 Analyzable Set
	1.2	Assess participant-reported anxiety resulting from use of the MCED test	MCED-V2 Analyzable Set
	1.3	Assess participants' reported outcomes and perceptions about the MCED test	MCED-V2 Analyzable Set
	2	**Diagnostic evaluations**	
	2.1	Characterize the number and type of diagnostic evaluations by predicted cancer signal origin and outcome of diagnostic resolution	MCED-V2 Cancer Signal Detected Analysis Set<br>MCED-I Cancer Signal Detected Analysis Set
	2.2	Evaluate the radiation exposure during diagnostic evaluation by imaging tests to inform safety	MCED-V2 Cancer Signal Detected Analysis Set<br>MCED-I Cancer Signal Detected Analysis Set
	2.3	Describe the ability of diagnostic PET-CT to confirm cancer in participants where diagnostic resolution of	MCED-V2 Cancer Signal Detected Analysis Set

CONFIDENTIALITY

This document is the confidential and proprietary information of GRAIL, LLC. Except as required by federal, state, or local laws or regulations, this document may not be disclosed to others, in whole or in part without prior written permission of GRAIL, Inc.

Objective type	N	Study objective	Analysis Set
		cancer diagnosis is not achieved through the targeted diagnostic testing triggered by MCED test results	
	3	**Test performance**	
	3.1	Evaluate performance of the MCED test in selected demographic and clinical subgroups	MCED-V2 Analyzable Set<br>MCED-I Analyzable Set
	3.2	Evaluate MCED test positive rate in subgroups with potential cross-reactive conditions	MCED-I Analyzable Set
	3.3	Evaluate concordance of initial GRAIL test result and CSO prediction, with results from the research blood draw	MCED-V2 Cancer Signal Detected Analysis Set<br>MCED-I Analyzable Set
**Exploratory (selected)**	1	Evaluate performance of the MCED test over three years of follow-up	MCED-V2 Analyzable Set<br>MCED-I Analyzable Set

14.1.2 Subgroup Considerations and Confidence Intervals

Distribution of variables will be presented using descriptive statistics as described in Section 13.1.

If the number of participants within a certain subgroup (e.g. cancer type) is less than 5 (five), performance endpoints such as PPV or change in health-related quality of life for the corresponding subgroups will not be reported individually in all analyses, unless otherwise specified. Instead, the results for these subgroups with small sample sizes (<5) will be provided in the by-participant listings in the clinical study report (CSR).

Two-sided 95% confidence intervals (CIs) will be constructed using the Wilson (score) method[6,7,8] for performance measures such as PPV which are not expected to be very near 0 or 1. Modified Wilson (score) confidence intervals[9,10,11] will be used for performance measures such as specificity and NPV which are expected to be near 0 or 1.

---

[6] Wilson, E. B. (1927). Probable Inference, the Law of Succession, and Statistical Inference. *Journal of the American Statistical Association, 22*(158), 209-212

[7] EP12-A2 (2008), User Protocol for Evaluation of Qualitative Test Performance; Approved Guideline-Second Edition. CLSI document EP12-A2. Wayne, PA. Clinical Laboratory Standards Institute; 2008.

[8] FDA (2007), "Guidance for industry and FDA staff: Statistical guidance on reporting results from studies evaluating diagnostic tests," Silver Spring: US Food and Drug Administration.

[9] Newcombe, R. G. (1998a), "Two-sided confidence intervals for the single proportion: Comparison of seven methods," *Statistics in Medicine*, 17, 857–872

[10] Newcombe, R. G. (1998b), "Interval estimation for the difference between independent proportions: Com- parison of eleven methods," *Statistics in Medicine*, 17, 873–890

[11] Agresti, A., and Coull, B. A. (1998), "Approximate is better than "exact" for interval estimation of binomial proportions," *The American Statistician*, 52, 119–126

CONFIDENTIALITY
This document is the confidential and proprietary information of GRAIL, LLC. Except as required by federal, state, or local laws or regulations, this document may not be disclosed to others, in whole or in part without prior written permission of GRAIL, Inc.

The analyses will be conducted using software R version 3.6.0 or later.

14.1.3 Cancer Status Assessments and Loss to Follow-Up

Cancer status assessment will be conducted annually:

1. Assessment of cancer status for the 12 months of follow-up is considered to be completed (i.e. no loss to follow-up) when
   - cancer diagnosis date is within the first 12 months of follow-up period, or
   - no cancer diagnosis has been documented within the first 12 months of follow-up period based on EMR/direct contact.

   Loss to follow-up is defined as no cancer diagnosis reported at the time of the last EMR/direct contact and the last EMR/direct contact occurred less than 11 months after the blood draw.

2. Assessment of cancer status for the first 24 months of follow-up is considered to be completed (i.e. no loss to follow-up) when
   - cancer diagnosis is reported at any time during the first 24 months of follow-up, or
   - no cancer diagnosis has been documented within the first 24 months of follow-up period based on EMR/direct contact.

   Loss to follow-up is defined as no cancer diagnosis reported at the time of the last EMR/direct contact and the last EMR/direct contact occurred less than 23 months after the blood draw.

3. Assessment of cancer status for the entire 36 months of follow-up (end of study cancer status assessment) is considered to be completed (i.e. no loss to follow-up) when
   - cancer diagnosis is reported at any time during the entire study period, or
   - no cancer diagnosis has been documented during the entire study period based on EMR/direct contact.

   Loss to follow-up is defined as no cancer diagnosis reported at the time of the last EMR/direct contact and the last EMR/direct contact less than 35 months after the blood draw.

Note that if a participant was "lost to follow-up" during the first 12 months but the site was able to conduct cancer status assessment at 24 months, this participant will be included in the analysis of 24 months follow-up. In addition, such participants will be included in the analysis of the first 12 months follow-up if this analysis is conducted after the 24 months assessment was completed. Similar logic applies for the 36 months follow-up period.

14.1.4 Weighted Analyses for MCED-I

As described in Section 4.2, blood samples from a subset of PATHFINDER 2 participants will be analyzed with MCED-I test as part of the bridging analysis. Participants for the bridging analysis will be selected via stratified random sampling as described in Section 4.2 and Appendix 9.

CONFIDENTIALITY
This document is the confidential and proprietary information of GRAIL, LLC. Except as required by federal, state, or local laws or regulations, this document may not be disclosed to others, in whole or in part without prior written permission of GRAIL, Inc.

Estimates of safety and test performance endpoints for MCED-I will be calculated with adjustment for sampling weights. The weight will be 1 for participants with a cancer diagnosis or MCED-V2 positive test result because all of these participants will be selected for the bridging analysis. The weights for participants with MCED-V2 negative test results and no cancer diagnosis will be calculated as the reciprocal of the sampling probabilities. Sampling probabilities are calculated as the proportion of participants to be selected out of all participants in the MCED-V2 Analyzable Set eligible for selection within each stratum.

14.1.5 Analysis Time Window for PRO Questionnaires

The analysis of the PRO questionnaires will be based on questionnaires completed ≤ 2 months (61 days) after questionnaire release for pre-test, post-test (following return of MCED test results) and diagnostic resolution timepoints, and ≤ 3 months (91 days) after questionnaire release for end of study timepoint. Questionnaires completed outside of these windows are considered to be 'out of window' and not included in the analysis. Questionnaires will be considered to be filled out at a 'Different timepoint' and not included in the analysis if they were completed in the time interval not intended for a given timepoint, for example, if a pre-test questionnaire was filled out after the test result was communicated.

As a sensitivity analysis, a window of ≤ 1 month (30 days) will be applied to the post-test and diagnostic resolution timepoints as those assessments are most likely to be impacted by return of MCED test result and diagnostic resolution, respectively.

14.2 Analysis Methods for Primary Objectives

There are two primary objectives for the PATHFINDER 2 study:

1. Evaluate the safety of the MCED test in terms of diagnostic testing triggered by the MCED test result.
2. Evaluate performance of the MCED Test in individuals eligible for cancer screening.

14.2.1. Primary Objective One (Safety)

14.2.1.1 Safety Endpoints

Safety endpoints during diagnostic testing triggered in participants with a positive MCED test result include:

- Number and type of invasive procedures (as described in Appendix 10).
- Number and type of adverse events

If diagnostic testing was initiated prior to the return of the MCED-V2 test results based on signs and symptoms suspicious for cancer, incidental findings or other reasons to the MCED-V2 test result, these participants will not be included in the analysis of the first primary study objective. Their diagnostic testing will be presented in a listing in the CSR.

All safety endpoints will be evaluated for each participant from the date when the MCED-V2 test result is communicated to the participant to the date of diagnostic resolution or the end of the

CONFIDENTIALITY
This document is the confidential and proprietary information of GRAIL, LLC. Except as required by federal, state, or local laws or regulations, this document may not be disclosed to others, in whole or in part without prior written permission of GRAIL, Inc.

analysis period, whichever comes first.

In addition, safety with respect to the number and type of invasive procedures will be evaluated using the following endpoints:

- Primary endpoint
  - Safety Measure A: Number of participants with a positive MCED test result and an invasive procedure divided by the total number of analyzable participants.
- Secondary endpoints:
  - Safety Measure B: Number of invasive procedures among all participants with a positive MCED test result divided by the total number of analyzable participants.
  - Safety Measure C: Number of participants with a positive MCED test result and an invasive procedure divided by the total number of participants with a positive MCED test result
  - Safety Measure D: Number of invasive procedures among all participants with a positive MCED test result divided by the number of participants with a positive MCED test result

14.2.1.2 Analysis Methods for Safety Analyses of MCED-V2 Test

Invasive Procedures

The number and type of invasive procedures conducted during diagnostic testing triggered by MCED-V2 positive test results in participants with a positive MCED-V2 test result will be summarized descriptively for each of the following categories (see details in Appendix 10):

- All invasive procedures
  - Surgical procedures
  - Non-surgical procedures
    - Endoscopy
    - Biopsy
    - Other non-surgical procedures

The following will be summarized:

- The number of participants with at least one invasive procedure from each of the categories described above will be reported overall and by type
- The total number of invasive procedures from each of the categories will be reported overall and by type
- The distribution of the per-participant counts for each of the categories will be summarized using descriptive statistics

These analyses will be reported for the following groups of participants overall and by type of invasive procedure as described above:

- Cancer vs no cancer diagnosis

CONFIDENTIALITY
This document is the confidential and proprietary information of GRAIL, LLC. Except as required by federal, state, or local laws or regulations, this document may not be disclosed to others, in whole or in part without prior written permission of GRAIL, Inc.

    - Participants with cancer diagnosis (TP)
        - Participants with cancer diagnosis at diagnostic resolution
        - Participants with no cancer diagnosis at diagnostic resolution who have a cancer diagnosis at the end of the analysis period
    - Participants with no cancer diagnosis (FP)
        - Participants with no cancer diagnosis at diagnostic resolution and no cancer diagnosis at the end of the analysis period
        - Participants who did not reach a diagnostic resolution and so do not have a cancer diagnosis at the end of the analysis period

- Initial vs subsequent diagnostic evaluation
    - Participants with an invasive procedure performed as part of their initial diagnostic evaluation (e.g., the first test(s) completed to evaluate a positive MCED-V2 test result)
    - Participants with an invasive procedure performed after initial diagnostic evaluation (e.g., test(s) ordered after review of results from initial diagnostic evaluation)

- CSO-guided vs not CSO-guided diagnostic evaluation
    - CSO-guided diagnostic evaluation based on the diagnostic evaluation including imaging tests, laboratory tests, non-invasive procedures and invasive procedures: participants with the diagnostic evaluations consistent with the recommended targeted diagnostic testing for predicted CSO(s) as described in the study protocol (Appendix 11)[12].
    - Not CSO-guided diagnostic evaluation based on the diagnostic evaluation including imaging tests, laboratory tests, non-invasive procedures and invasive procedures: participants with diagnostic evaluations inconsistent with the recommended targeted diagnostic testing for predicted CSO(s)

The results will be reported for:

- All invasive procedures performed after return of MCED-V2 test result and prior to diagnostic resolution
- Invasive procedures performed after the diagnostic PET-CT performed after the initial negative diagnostic evaluation (referred to as the 'confirmatory PET-CT' in the study protocol) and prior to diagnostic resolution in participants who had such diagnostic PET-CT

Adverse Events During Diagnostic Testing

The number of adverse events (AE) reported during diagnostic testing triggered by MCED positive test results in participants with a positive MCED-V2 test result will be described. This includes AEs reported from the date when the MCED-V2 test result is communicated to the participant to the date of diagnostic resolution or the end of the analysis period, whichever comes first. The AEs will be summarized by the following characteristics:

---

[12] Evaluation of consistency is based on whether the participant received the expected CSO-guided diagnostic evaluations described in the protocol during their initial diagnostic evaluation.

CONFIDENTIALITY

This document is the confidential and proprietary information of GRAIL, LLC. Except as required by federal, state, or local laws or regulations, this document may not be disclosed to others, in whole or in part without prior written permission of GRAIL, Inc.

- Seriousness (whether AE was considered an SAE)
  - If yes, seriousness criteria (death, life threatening, required hospitalization-initial or prolonged, results in persistent or significant disability/incapacity, required intervention to prevent permanent impairment, congenital anomaly/birth defect, other medically important serious event)
- Severity (mild, moderate, severe)
- Relatedness to study activities (i.e., study activities listed in the protocol Schedule of Activities) or device
- Outcome (e.g. recovered/ resolved, recovered/ resolved with sequelae, recovering/ resolving, not recovered/ not resolved, fatal)

AEs will be described for the following subsets:

- Related to study activities, related to device
- SAEs

AEs will be reported for the following time periods:

- All AEs reported after return of MCED-V2 test result and prior to diagnostic resolution
- AEs reported after diagnostic PET-CT (among participants who had confirmatory PET-CT) and prior to diagnostic resolution

These analyses will be conducted for all participants and for the following groups of participants, separately:

- Participants with a cancer diagnosis at diagnostic resolution
- Participants with no cancer diagnosis at diagnostic resolution
- Participants who did not reach a diagnostic resolution during the analysis period.

A per-participant listing of SAEs in participants with no cancer diagnosis at diagnostic resolution will be provided.

14.2.1.3 Analysis Methods for Safety Analyses of MCED-I Test

Invasive Procedures

Diagnostic evaluations informed by MCED-V2 test results will be leveraged to estimate the number of TP and FP participants with at least one invasive procedure and the number of invasive procedures expected for the diagnostic evaluations informed by MCED-I test results in the PATHFINDER 2 study.

The number of participants with at least one invasive procedure for MCED-I will be estimated as follows:

1. For each MCED-I CSO which corresponds to a single MCED-V2 CSO (see Appendix 5A), Table 3 will be constructed
   - The number of invasive procedures during diagnostic evaluation may be impacted by cancer status at diagnostic resolution and accuracy of CSO prediction, which is why Table 3 includes these categories

CONFIDENTIALITY
This document is the confidential and proprietary information of GRAIL, LLC. Except as required by federal, state, or local laws or regulations, this document may not be disclosed to others, in whole or in part without prior written permission of GRAIL, Inc.

2. Using notation in Table 3, the number of participants with at least one invasive procedure for MCED-I CSO J will be estimated as $Y_J = (X_1/N_1)* M_1 + (X_2/N_2)* M_2 + (X_3/N_3)* M_3$
   - If one of the categories in Table 3 was not observed for MCED-V2, the values will be imputed based on another category closest in terms of cancer status, if available (e.g. if "TP & correct CSO" is missing, values for "TP & incorrect CSO" will be used, and vice versa). If no TP were observed for a given MCED-V2 CSO, data for FPs for that CSO will be used and vice versa. If there were no calls of a given MCED-V2 CSO at all, the results for the CSO with the most similar recommended diagnostic evaluation will be used (Appendix 11).
3. A similar approach will be utilized for MCED-I CSO of "Blood, lymphatic system, or bone marrow" which has 3 corresponding MCED-V2 CSOs (Table 4).
4. Using notation in Table 4, the number of participants with at least one invasive procedure for MCED-I CSO = "Blood, lymphatic system, or bone marrow" will be calculated as

$$Y_{Blood} = (X_{11} + X_{12} + X_{13}) / (N_{11} + N_{12} + N_{13}) * M_1 + (X_{21} + X_{22} + X_{23}) / (N_{21} + N_{22} + N_{23})* M_2 + (X_{31} + X_{32} + X_{33}) / (N_{31} + N_{32} + N_{33}) * M_3$$

5. The resulting counts across all MCED-I CSOs will be summed up to obtain the total number of participants with at least one invasive procedure.

Table 3. Data for estimating the number of participants with at least one invasive procedure for MCED-I CSO predictions with a single corresponding MCED-V2 CSO prediction

MCED-I CSO prediction	Category based on cancer status and accuracy of CSO prediction	Number of MCED-V2 CSO predictions	Number of participants with at least one invasive procedures for MCED-V2	Number of MCED-I CSO predictions
CSO J such as "Breast"	TP & correct CSO	N_1	X_1	M_1
	TP & incorrect CSO	N_2	X_2	M_2
	FP*	N_3	X_3	M_3

*Participants who did not reach diagnostic resolution at the end of the analysis period will be included in this group

CONFIDENTIALITY
This document is the confidential and proprietary information of GRAIL, LLC. Except as required by federal, state, or local laws or regulations, this document may not be disclosed to others, in whole or in part without prior written permission of GRAIL, Inc.

Table 4. Data for estimating the number of participants with at least one invasive procedure for MCED-I CSO prediction of "Blood, lymphatic system, or bone marrow"

Category based on cancer status and accuracy of CSO prediction	MCED-V2 CSO prediction	Number of MCED-V2 CSO predictions	Number of invasive procedures for MCED-V2	Number of MCED-I CSO predictions
TP with correct CSO (heme)	Lymphoid neoplasm	N_{11}	X_{11}	M_1
	Myeloid neoplasm	N_{12}	X_{12}	
	Plasma cell neoplasm	N_{13}	X_{13}	
TP with incorrect CSO (solid)	Lymphoid neoplasm	N_{21}	X_{21}	M_2
	Myeloid neoplasm	N_{22}	X_{22}	
	Plasma cell neoplasm	N_{23}	X_{23}	
FP	Lymphoid neoplasm	N_{31}	X_{31}	M_3
	Myeloid neoplasm	N_{32}	X_{32}	
	Plasma cell neoplasm	N_{33}	X_{33}	

To describe the estimated number of invasive procedures in participants with a positive MCED-I test result the following estimates will be reported for all invasive procedures, non-surgical procedures and surgical procedures as described in Appendix 10:

- Number of participants with at least one invasive procedure,
- Total number of invasive procedures
- Safety measures A-D.

These analyses will be conducted for the following groups of participants with positive MCED-I test results, separately:

- Participants with a cancer diagnosis during the analysis period (TP),
- Participants with no cancer diagnosis during the analysis period (FP).

Adverse Events During Diagnostic Testing

The number of adverse events reported during diagnostic testing in participants with a positive MCED-I test result will be estimated using the approach illustrated in Tables 3 and 4. These calculations will be repeated to characterize the number of adverse events by:

- Severity (mild, moderate, severe)
- Outcome (e.g. recovered/ resolved, recovered/ resolved with sequelae, recovering/ resolving, not recovered/ not resolved, fatal)

CONFIDENTIALITY
This document is the confidential and proprietary information of GRAIL, LLC. Except as required by federal, state, or local laws or regulations, this document may not be disclosed to others, in whole or in part without prior written permission of GRAIL, Inc.

- Whether adverse event was considered an SAE
    - If yes, seriousness criteria (death, life threatening, required hospitalization-initial or prolonged, results in persistent or significant disability/incapacity, required intervention to prevent permanent impairment, congenital anomaly/birth defect, other medically important serious event)
- Whether adverse event was related to the device
- Whether adverse event was related to study activities

These analyses will be conducted for all participants and for the following groups of participants, separately:

- Participants with a cancer diagnosis during the analysis period,
- Participants with no cancer diagnosis during the analysis period.

14.2.2. Primary Objective Two (Test Performance)

14.2.2.1 Test Performance Endpoints

Test performance of each MCED test version will be evaluated for:

- Cancer signal detection – based on the comparison of the MCED test result for cancer signal detection (positive or negative) with the target clinical outcome for a participant (presence or absence of cancer diagnosis during a follow-up period)
- CSO prediction – based on the comparison of the CSO prediction with the clinical CSO
- Combined cancer signal detection and CSO prediction

In addition, concordance of the MCED-V2 test and MCED-I test results will be evaluated.

14.2.2.1.1 Test Performance Endpoints for Cancer Signal Detection

Performance of the MCED test will be evaluated using test performance endpoints defined based on a 2x2 contingency table (Table 5) which will be constructed based on the cancer status at a given analysis time point.

Table 5. 2x2 Contingency Table

		**Cancer Status**	
		**Present**	**Absent**
**MCED Test Result for Cancer SIgnal Detection**	**Positive**	TP	FP
	**Negative**	FN	TN

Where

- TP = True Positives: number of participants with positive MCED test result and a cancer diagnosis

CONFIDENTIALITY
This document is the confidential and proprietary information of GRAIL, LLC. Except as required by federal, state, or local laws or regulations, this document may not be disclosed to others, in whole or in part without prior written permission of GRAIL, Inc.

- FP = False Positives: number of participants with positive MCED test result and no cancer diagnosis
- FN = False Negatives: number of participants with negative MCED test result and a cancer diagnosis
- TN = True Negatives: number of participants with negative MCED test result and no cancer diagnosis

The following endpoints will be used to evaluate overall test performance for cancer signal detection:

Positive Predictive Value

Positive Predictive Value (PPV) for cancer signal detection is defined as the proportion of participants with a cancer diagnosis out of all participants with a positive MCED test result. It is calculated as TP / (TP + FP) from the 2x2 contingency table above (Table 5).

Negative Predictive Value

Negative Predictive Value (NPV) for cancer signal detection is defined as the proportion of participants with no cancer diagnosis out of all participants with a negative MCED test result. It is calculated as TN / (TN + FN).

Episode Sensitivity

Episode sensitivity is defined as the ability of a screening test to detect cancers that would be diagnosed during a follow-up period after a specific round of a screening test [13,14,15,16].

Episode Sensitivity for cancer signal detection is estimated as the proportion of participants with a positive MCED test result out of all participants with a cancer diagnosis within the follow-up period. It is calculated as TP / (TP + FN).

Specificity

Specificity for cancer signal detection is defined as the proportion of participants with a negative MCED test result out of all participants with no cancer diagnosis within the follow-up period. Participants who did not have a completed cancer status assessment at the end of the follow-up period are not included in the calculations. Specificity is calculated as TN / (TN + FP).

Positive Likelihood Ratio

The Positive Likelihood Ratio (PLR) for cancer signal detection is defined as the ratio of the probability that a participant with cancer diagnosis has a positive test result vs the probability that a participant with no cancer diagnosis has a positive test result. It is calculated as episode sensitivity divided by (1 - specificity), i.e., [TP/ (TP + FN)] / [1 - TN/ (TN + FP)] = TP * (TN + FP) / [FP * (TP + FN)].

---

[13] Day NE, Walter SD. Simplified models of screening for chronic disease: estimation procedures from mass screening programmes. Biometrics 1984 40: 1–14.
[14] Cole P, Morrison AS. Basic Issues in Population Screening for Cancer, JNCI 1980 64: 1263–72.
[15] Hakama et al, Sensitivity in cancer screening. J Med Screen 2007:174–177
[16] Sarkeala et al, Episode sensitivity in association with process indicators in the Finnish breast cancer screening program. Int J of Cancer Screening 2006; 118: 174-179

CONFIDENTIALITY
This document is the confidential and proprietary information of GRAIL, LLC. Except as required by federal, state, or local laws or regulations, this document may not be disclosed to others, in whole or in part without prior written permission of GRAIL, Inc.

Negative Likelihood Ratio

The Negative Likelihood Ratio (NLR) for cancer signal detection is defined as the ratio of the probability that a participant with cancer diagnosis has a negative test result vs the probability that a participant with no cancer diagnosis has a negative test result. It is calculated as (1 - episode sensitivity) divided by specificity, i.e., [1 - TP/ (TP + FN)] / [TN/ (TN + FP)] = FN * (TN + FP) / [TN * (TP + FN)].

MCED Cancer Detection Rate

MCED Cancer Detection Rate (CDR) is defined as the proportion of participants with a positive MCED test result and a cancer diagnosis (true positives) out of all participants in the analyzable set. It is calculated as TP / (TP + FP + FN + TN).

Number Needed to Screen

Number needed to screen is defined as the number of participants that need to be screened with an MCED test to detect a single cancer. It is calculated as 1 / CDR, i.e. (TP + FP + FN + TN) / TP.

In addition, the following measures will be used to evaluate CSO prediction or cancer type specific test performance for cancer signal detection:

PPV for cancer signal detection by CSO prediction

PPV for cancer signal detection by a specific CSO prediction is calculated for each CSO prediction (Appendix 3 and 4), where sample size allows. It is calculated as the proportion of participants with cancer diagnosis out of all participants with positive MCED test result and a specific CSO1 prediction within the follow-up period, whenever the sample size allows.

Episode Sensitivity for cancer signal detection by cancer type

Episode sensitivity for cancer signal detection by a specific cancer type is calculated as the proportion of participants with a positive MCED test result out of all participants with a cancer diagnosis of a specific cancer type within the follow-up period, whenever the sample size allows.

Episode Sensitivity for cancer signal detection for prespecified cancer subgroups

Episode sensitivity for cancer signal detection for prespecified cancer subgroups is calculated as the proportion of participants with a positive MCED test result out of all participants with a cancer diagnosis of one of the cancer types in the prespecified cancer subgroup within the follow-up period.

Prespecified cancer subgroups (defined in Appendix 7) include the following:

- Cancers Responsible for 2/3 of all Cancer Deaths in the US
- Cancers without Common Screening Options
- Solid Tumors with Common Screening Options
- Solid Tumors without Common Screening Options
- Hematologic Malignancies

CONFIDENTIALITY
This document is the confidential and proprietary information of GRAIL, LLC. Except as required by federal, state, or local laws or regulations, this document may not be disclosed to others, in whole or in part without prior written permission of GRAIL, Inc.

14.2.2.1.2 Test Performance Endpoints for CSO Prediction

Test performance endpoints for CSO prediction are focused on participants with a positive MCED test result and a cancer diagnosis at a given time point, i.e., TP.

Accuracy of CSO prediction is defined as the proportion of participants with a correct CSO prediction (i.e. CSO prediction matching clinical CSO) among TP participants, including CUP and cases with "Unassigned" clinical CSO.

Specifically, the following CSO accuracy calculations are used in this study:

- **Primary endpoint: accuracy of CSO prediction in true positives** is defined as the proportion of correct CSO1 predictions among TP participants.
    - For participants with multiple cancers, if CSO1 prediction matches one of the clinical CSOs, then it is considered a correct prediction and this participant with multiple cancers will be included in the numerator.
- **Secondary endpoint: accuracy of CSO1/2 prediction in true positives** is defined as the proportion of correct CSO1 or CSO2 predictions among TP participants. In this calculation, if any of the CSO1 or CSO2 predictions match the clinical CSO, then it is considered a correct prediction. This measure will only be calculated for the MCED-V2 test.
    - For participants with multiple cancers, if CSO1 or CSO2 predictions match one of the clinical CSOs, then it is considered a correct prediction and this participant with multiple cancers will be included in the numerator.

Confusion Matrix

A confusion matrix comparing the CSO1 prediction (rows) with the clinical CSOs (columns) will be constructed for TP participants (Table 6).

Participants diagnosed with cancer with no clinical CSO, such as a CUP or a cancer diagnosis with "Unassigned" clinical CSO, will not be included in the confusion matrix. For TP participants with multiple cancers, if at least one of the cancers has an assigned clinical CSO, those participants will be included in the confusion matrix. If one of the clinical CSOs is matched with the CSO prediction, then this participant will be counted under that clinical CSO diagonal entry. If none of the clinical CSOs is matched with the CSO1 prediction, then this participant will be counted under the clinical CSO linked to the first cancer diagnosed during the follow-up period.

CONFIDENTIALITY
This document is the confidential and proprietary information of GRAIL, LLC. Except as required by federal, state, or local laws or regulations, this document may not be disclosed to others, in whole or in part without prior written permission of GRAIL, Inc.

Table 6. Sample Confusion Matrix

CSO1 for a given MCED test	Clinical CSO 1	Clinical CSO 2	Clinical CSO 3	…	Clinical CSO_n	Total	CSO-Specific Precision
**CSO Prediction 1**	a_{11}	a_{12}	a_{13}	…	a_{1n}	X1	a_{11} / X1
**CSO Prediction 2**	a_{21}	a_{22}	a_{23}	…	a_{2n}	X2	a_{22} / X2
**CSO Prediction 3**	a_{31}	a_{32}	a_{33}	…	a_{3n}	X3	a_{33} / X3
**…**	…	…	…	…	…	…	…
**CSO Prediction n**	a_{n1}	a_{n2}	a_{n3}	…	a_{nn}	Xn	a_{nn} / Xn
**Total**	Y1	Y2	Y3	…	Yn		
**CSO-Specific Accuracy**	a_{11} / Y1	a_{22} / Y2	a_{33} / Y3	…	a_{nn} / Yn		

The following endpoints are calculated from the confusion matrix:

- **Accuracy of a CSO prediction in true positives by clinical CSO** is defined as the proportion of participants with a correct CSO prediction out of all the TP participants who have been diagnosed with a cancer corresponding to that clinical CSO. It is estimated by the proportion of diagonal cells in each column of the confusion matrix.
- **Precision of a CSO prediction in true positives by CSO prediction** is defined as the proportion of participants whose CSO prediction matches their clinical CSO out of all TP participants with a given CSO prediction. It is estimated as the proportion of diagonal cells in each row of the confusion matrix

14.2.2.1.3 Exploratory Test Performance Endpoints for Combined Cancer Signal Detection and CSO Prediction

Test performance endpoints for combined cancer signal detection and CSO prediction are based on Table 7 and include performance endpoints described below.

CONFIDENTIALITY
This document is the confidential and proprietary information of GRAIL, LLC. Except as required by federal, state, or local laws or regulations, this document may not be disclosed to others, in whole or in part without prior written permission of GRAIL, Inc.

Table 7. CSO prediction by clinical CSO.

CSO1 for a given MCED test	Clinical CSO 1	..	Clinical CSO n	Cancers with no clinical CSO (NCCSO)	No cancer diagnosis	Total
**CSO Prediction 1**	$N_{1,1}$			$NCCSO_1$	NC_1	N_1
**..**						
**CSO Prediction n**			$N_{n,n}$	$NCCSO_n$	NC_n	N_n
**TOTAL**	C_1	..	C_n	NCCSO	NC	N

PPV accounting for CSO accuracy

PPV accounting for CSO accuracy is calculated as the proportion of participants whose CSO prediction matches their clinical CSO out of all participants with positive MCED test results. This is defined as $\sum N_{J,J} / N$ using nomenclature of Table 7.

PPV accounting for CSO accuracy by CSO prediction

PPV accounting for CSO accuracy by CSO prediction is calculated as the proportion of participants whose CSO prediction matches their clinical CSO out of all participants with positive MCED test results with a specific CSO prediction, whenever the sample size allows. This is defined as $N_{J,J} / N_J$ using nomenclature of Table 7.

Episode Sensitivity accounting for CSO accuracy

Episode sensitivity accounting for CSO accuracy is calculated as the proportion of participants whose CSO prediction matches their clinical CSO out of all participants with a cancer diagnosis within the follow-up period. This is defined as $\sum N_{J,J} / (\sum C_J + NCCSO + FN)$ using nomenclature of Table 7.

Episode Sensitivity accounting for CSO accuracy by clinical CSO

Episode sensitivity accounting for CSO accuracy by clinical CSO is calculated as the proportion of participants whose CSO prediction matches their clinical CSO out of all participants with a cancer diagnosis and specific clinical CSO assigned, whenever the sample size allows. This is defined as $N_{J,J} / (C_J + FN_J)$ using nomenclature of Table 7, where FN_J is the number of cancers with clinical CSO_J and MCED negative test result.

14.2.2.1.4 Analyses by Stage

Distribution of stage for new cancers and extent of recurrence for recurrent cancers will be

CONFIDENTIALITY
This document is the confidential and proprietary information of GRAIL, LLC. Except as required by federal, state, or local laws or regulations, this document may not be disclosed to others, in whole or in part without prior written permission of GRAIL, Inc.

summarized for all cancers with:

- MCED positive test results
- MCED negative test results

Stage-specific estimates of episode sensitivity (as defined in Section 14.2.1.1) may be biased because cancer status is not ascertained for all study participants at the time of blood draw. This phenomenon has been reported in a recent simulation study[17]. Specifically, while the stage for an MCED test detected cancer would be determined close to the time of the blood draw, the stage for a cancer that is not detected by the MCED test would be determined at the time of clinical presentation, which may occur much later than the time of blood draw. Therefore clinically presenting cancers are more likely to be diagnosed at a more advanced stage than the screen-detected cancers, which is expected to cause differential bias when describing episode sensitivity by stage.

14.2.2.1.5 Concordance of MCED-V2 Test and MCED-I Test Results

For participants in both the MCED-V2 Analyzable Set and MCED-I Analyzable Set, a 2x2 matrix of MCED-V2 and MCED-I test results will be constructed separately for participants with cancer diagnosis, participants without cancer diagnosis, and all participants combined (see Table 8)

Table 8. Concordance of MCED-V2 and MCED-I test results for cancer signal detection

Category	MCED-I positive	MCED-I negative
MCED-V2 positive	A	B
MCED-V2 negative	C	D

Percent agreement between the two test versions and corresponding confidence intervals will be calculated:

- Positive Percent Agreement (PPA) = 100% * A / (A+B)
- Negative Percent Agreement (NPA) = 100% * D / (C+D)
- Overall Percent Agreement (OPA) = 100% * (A+D) / (A+B+C+D)

In addition, a descriptive summary of the concordance between MCED-V2 CSO predictions and MCED-I CSO predictions in participants with positive test results for both test versions will be provided. It will be presented as a matrix (Appendix 5B) and the proportion of participants with the same MCED-V2 CSO1 prediction and MCED-I CSO1 prediction will be reported in participants with cancer diagnosis, participants without cancer diagnosis, and all participants combined.

[17] Pinsky P, Lange J, Etzioni R, Estimating stage-specific sensitivity for cancer screening tests, Journal of Medical Screening, 2023

CONFIDENTIALITY
This document is the confidential and proprietary information of GRAIL, LLC. Except as required by federal, state, or local laws or regulations, this document may not be disclosed to others, in whole or in part without prior written permission of GRAIL, Inc.

14.2.2.1.6 Descriptive Summaries

Participants with TP, FP, FN and TN results at each time point will be described as follows:

- TP: distribution of
    - CSO predictions
    - Cancer types
    - Number (proportion) of cancers in each of the pre-specified cancer groups (as defined in Appendix 7)
    - Stage for new cancers (stage I, II, III, IV, I-II, I-III)
    - Extent of recurrence for recurrent cancers
    - Multiple cancers
    - Pathology-confirmed cancers by cancer type
    - Treatment
        - Number of participants who had a treatment planned for their cancer
        - Type of treatment among participants with treatment planned (Chemotherapy, Hormone therapy, Immunotherapy, Radiation therapy, Targeted therapy, Stem cell or bone marrow transplant, Surgery, Other)
        - Intent of treatment among participants with treatment planned (Curative, Palliative, Surveillance, Other, Unknown)
- FP: distribution of
    - CSO predictions
    - Non-cancer conditions identified during diagnostic evaluation
        - Benign neoplasms (benign solid neoplasm, lymphoproliferative disorder, monoclonal gammopathy of undetermined significance, other)
        - Benign non-neoplastic conditions (Autoimmune disease, Chronic viral infection, Esophageal disease (e.g., Barrett's esophagus, GERD), Gastritis or gastric (peptic) ulcers, Inflammatory bowel disease (i.e., Crohn's disease or ulcerative colitis), Liver disease (e.g., cirrhosis or fatty liver disease), Lung disease (e.g., pulmonary fibrosis, emphysema, chronic bronchitis), Pancreatic disease (e.g., pancreatitis), Other
- FN: distribution of
    - Cancer types
    - Number (proportion) of cancers in each of the pre-specified cancer groups (as defined in Appendix 7)
    - Stage for new cancers (stage I, II, III, IV, I-II, I-III)
    - Extent of recurrence for recurrent cancers
    - Multiple cancers
    - Pathology-confirmed cancers by cancer type
    - Treatment
        - Number of participants who had a treatment planned for their cancer
        - Type of treatment among participants with treatment planned (Chemotherapy, Hormone therapy, Immunotherapy, Radiation therapy, Targeted therapy, Stem cell or bone marrow transplant, Surgery, Other)
        - Intent of treatment among participants with treatment planned (Curative, Palliative, Surveillance, Other, Unknown)

CONFIDENTIALITY
This document is the confidential and proprietary information of GRAIL, LLC. Except as required by federal, state, or local laws or regulations, this document may not be disclosed to others, in whole or in part without prior written permission of GRAIL, Inc.

    - Method of cancer diagnosis (Follow-up surveillance of prior imaging test finding, Incidental finding, GRAIL MCED test taken as part of study, GRAIL MCED test taken outside of study, Patient reported signs or symptoms, Post treatment surveillance for recurrence, Cancer screening test, Other)
- TP, FP, TN, FN: distribution of
    - Age: 50-59, 60-69,70-79, 80+ years
    - Biological sex
    - Race categories aligned with FDA guidance (as described in Section 13.3)
    - Ethnicity
    - BMI categories (<25 kg/m^2, 25 to <30 kg/m^2, ≥30 kg/m^2)
    - Smoking status (never smoker, former smoker, current smoker)
    - USPSTF lung cancer risk indicator (as described in Section 13.3)
    - Prior history of cancer (Yes, No)
    - Presence of genetic cancer predisposition (Yes, No)

We will also describe participants with positive MCED test results, no cancer diagnosis at time of diagnostic resolution and cancer diagnosis at 12, 24 or 36 months in terms of:

- CSO predictIons
- Cancer type
- Number (proportion) of cancers in each of the pre-specified cancer groups (as defined in Appendix 7)
- Stage for new cancers (stage I, II, III, IV, I-II, I-III)
- Extent of recurrence for recurrent cancers
- Multiple cancers
- Pathology-confirmed cancers by cancer type
- Method of cancer diagnosis
- Age: 50-59, 60-69,70-79, 80+ years
- Biological sex
- Race categories aligned with FDA guidance (as described in Section 13.3)
- Ethnicity
- BMI categories (<25 kg/m^2, 25 to <30 kg/m^2, ≥30 kg/m^2)
- Smoking status (never smoker, former smoker, current smoker)
- USPSTF lung cancer risk indicator (as described in Section 13.3)
- Prior history of cancer (Yes, No)
- Presence of genetic cancer predisposition (Yes, No)

14.2.2.2 Acceptance Criteria for PMA Analysis

Acceptance criteria

As part of the primary objective, the acceptance criteria for PMA analysis will be evaluated for the MCED-I test based on the 12 months follow-up time analysis period using hierarchical testing.

- First, two co-primary endpoints of episode sensitivity and specificity will be tested to demonstrate that episode sensitivity is significantly higher than 22% and that specificity is

CONFIDENTIALITY
This document is the confidential and proprietary information of GRAIL, LLC. Except as required by federal, state, or local laws or regulations, this document may not be disclosed to others, in whole or in part without prior written permission of GRAIL, Inc.

significantly higher than 99%. These targets represent what the lower bound of the 95% confidence interval for each endpoint should exceed.

Hypothesis testing for the acceptance criteria co-primary endpoints:

1. H_0: Episode sensitivity for cancers responsible for ⅔ of all cancer deaths in the US (as defined in Appendix 7) ≤ 22% vs

   H_A: Episode sensitivity for cancers responsible for ⅔ of all cancer deaths in the US (as defined in Appendix 7) > 22%

2. H_0: Specificity ≤ 99% vs

   H_A: Specificity > 99%

To claim success on the primary acceptance criteria, statistical significance (with one sided p-value < 0.025) needs to be achieved for both of the co-primary endpoints.

- Second, conditional on the success of the co-primary endpoints, episode sensitivity for cancers without common screening options (as defined in Appendix 7) will be tested to demonstrate that it is significantly higher than 19% with one sided p-value < 0.025.

Hypothesis testing for the acceptance criteria secondary endpoint:

H_0: Episode sensitivity for cancers without common screening options (as defined in Appendix 7) ≤ 19% vs

H_A: Episode sensitivity for cancers without common screening options (as defined in Appendix 7) > 19%

- Third, conditional on the success of all the previous endpoints, episode sensitivity for all cancers will be tested to demonstrate that it is significantly higher than 10% with one sided p-value < 0.025.

Hypothesis testing for the acceptance criteria secondary endpoint:

H_0: Episode sensitivity for all cancers ≤ 10% vs

H_A: Episode sensitivity for all cancers > 10%

- Subsequently, conditional on the success of all the previous endpoints, accuracy of CSO prediction in true positives will be tested to demonstrate that it is significantly higher than 70% with one sided p-value < 0.025.

Hypothesis testing for the acceptance criteria quaternary endpoint:

H_O: accuracy of CSO prediction in true positives ≤ 70% vs

H_A: accuracy of CSO prediction in true positives > 70%

CONFIDENTIALITY

This document is the confidential and proprietary information of GRAIL, LLC. Except as required by federal, state, or local laws or regulations, this document may not be disclosed to others, in whole or in part without prior written permission of GRAIL, Inc.

Key cancer and CSO-specific performance endpoints as a descriptive summary

In addition, a set of clinically relevant cancer and CSO-specific performance endpoints will be provided descriptively for comprehensive evaluation of MCED-I test performance. No acceptance criteria will be set for these endpoints.

- Episode Sensitivity for cancer signal detection for additional prespecified cancer subgroups (Appendix 7)
    - Solid tumors with common screening options
    - Solid tumors without common screening options
    - Hematologic malignancies
- Episode Sensitivity for cancer signal detection by cancer type
- Episode Sensitivity accounting for CSO accuracy by clinical CSO
- Accuracy of CSO prediction in true positives by clinical CSO
- Precision of CSO prediction in true positives by CSO prediction
- PPV for cancer signal detection by CSO prediction
- PPV accounting for CSO accuracy by CSO prediction

14.2.2.3 Evaluation of MCED-V2 Test Performance

MCED-V2 test performance will be evaluated at several time points (see Section 14.1.3 for details on annual cancer status assessments):

- Time of diagnostic resolution - secondary time point
- 12 months after the date of the first blood draw[18] - primary time point
- 24 months after the date of the first blood draw - secondary time point

14.2.2.3.1 Analysis at the time of diagnostic resolution time point

Note that at the time of diagnostic resolution only performance endpoints based on positive MCED-V2 test results can be evaluated because there is no corresponding cancer status evaluation in MCED-V2-negative participants (defined as described in Section 14.2.2.1.1):

- PPV for cancer signal detection
- PPV for cancer signal detection by CSO prediction
- PPV accounting for CSO accuracy by CSO prediction
- Accuracy of CSO prediction in true positives
- Accuracy of a clinical CSO in true positives by clinical CSO
- Precision of a CSO prediction in true positives by CSO prediction

Two versions of the PPV endpoints will be evaluated:

1. Main endpoint: denominator includes MCED-V2 positive participants who achieved diagnostic resolution
2. Secondary endpoint: denominator includes all MCED-V2 positive participants. This means that participants who did not achieve diagnostic resolution by the time of the cancer status

[18] The blood draw date will be the first blood draw date if multiple blood draws have been performed

CONFIDENTIALITY
This document is the confidential and proprietary information of GRAIL, LLC. Except as required by federal, state, or local laws or regulations, this document may not be disclosed to others, in whole or in part without prior written permission of GRAIL, Inc.

assessment at 12 months and participants lost to follow-up are included in the denominator.

14.2.2.3.2 Analysis at 12 months follow-up time point

All test performance endpoints described in Section 14.2.2.1 will be evaluated based on the 12 months follow-up time period.

MCED Cancer Detection in Addition to Existing Cancer Screening Tests

To illustrate the benefit of MCED when used in addition to existing standard of care (SOC) cancer screening tests for cancer detection, the number of cancers detected during the 12 months follow-up period will be reported for the following groups:

- Group 1: Participants with MCED test positive results and cancer diagnosis
- Group 2: Participants with MCED test negative results whose cancer diagnosis was triggered by an existing screening test with grade A or B recommendation by USPSTF: breast, cervix, colorectal, and lung cancers
- Group 3: Participants with MCED test negative results whose cancer diagnosis was triggered by other cancer screening tests
    - Screening tests with grade C recommendation by USPSTF: prostate cancer
    - Screening tests without grade A, B or C USPSTF recommendations such as for thyroid or melanoma cancers
- Group 4: Participants with MCED test negative results whose cancer diagnosis was not triggered by cancer screening (e.g., clinically detected cancers)

Distribution of cancer type and stage will be summarized for each group.

14.2.2.3.3 Analysis at 24 months time point

Analyses described in Section 14.2.2.3.2 will be evaluated based on the 24 months follow-up.

14.2.2.4 Evaluation of MCED-I Test Performance

Evaluation of MCED-I test performance at the time of diagnostic resolution is not possible because cancer status for MCED-V2-negative: MCED-I-positive cases may not be directly assessed via diagnostic workup. Therefore, MCED-I test performance will be evaluated at 12 months and 24 months time points.

14.2.2.4.1 Analysis at 12 months follow-up time point

All performance endpoints described in Section 14.2.2.1 will be evaluated, including test performance endpoints for cancer signal detection, CSO prediction, combined cancer signal detection and CSO prediction, stage distribution, concordance of MCED-V2 test and MCED-I test results. All analyses for MCED-I will be adjusted for sampling weights as described in Section 14.1.4.

CONFIDENTIALITY
This document is the confidential and proprietary information of GRAIL, LLC. Except as required by federal, state, or local laws or regulations, this document may not be disclosed to others, in whole or in part without prior written permission of GRAIL, Inc.

Note that MCED-I test performance estimates are expected to be conservative because MCED-I positive cases that are MCED-V2 negative will not have a diagnostic workup for cancer at the time of the PMA analysis and therefore their cancer status is based on cancer status assessment after 12 months of follow-up time. Such cases therefore will be treated as FP in the PMA analysis. If such participants have a cancer diagnosis during year 2 follow-up, they will be treated as TP in the additional post-PMA analyses.

Analyses described in Section 14.2.2.3.2 will be carried out.

14.2.2.4.2 Analysis at 24 months time point

All performance endpoints described in Section 14.2.2.1 will be evaluated based on the 24 months follow-up.

14.3 Analysis Methods for Secondary Objectives

The following analyses will be performed to achieve the secondary objectives of this study.

14.3.1 Participant reported outcomes and behavioral intentions

14.3.1.1 Evaluate intention and utilization of guideline recommended cancer screening procedures after use of the MCED test

Intention to follow cancer screening recommendations

Intention to follow cancer screening recommendations will be assessed using participant-reported questionnaire pre-test, post-test and at 12 months (+30 days) in all participants. Responses to the question "How likely are you to follow your healthcare provider's cancer screening recommendations?" were represented by a 4-point Likert-type scale (i.e., Very Unlikely, Unlikely, Likely, and Very Likely).

Number and proportion of participants with each rating will be reported at each time point for the following analysis sets:

- No Cancer Signal Detected
  - No Cancer Signal Detected and cancer diagnosis (i.e. FN)
  - No Cancer Signal Detected and no cancer diagnosis (i.e. TN)
- Cancer Signal DetectedAnalyzable.

Individual item responses for baseline vs post-baseline assessment will be cross-tabulated. Change from baseline (pre-test) to post-baseline assessments at each time point will be illustrated for each analysis set by:

- Proportion of participants who changed their rating from Likely or Very Likely at baseline to Unlikely or Very Unlikely post-baseline
- Proportion of participants who changed their rating from Unlikely or Very Unlikely at baseline to Likely or Very Likely post-baseline

CONFIDENTIALITY
This document is the confidential and proprietary information of GRAIL, LLC. Except as required by federal, state, or local laws or regulations, this document may not be disclosed to others, in whole or in part without prior written permission of GRAIL, Inc.

Utilization of guideline recommended cancer screening

The utilization of guideline recommended standard of care (SOC) cancer screening tests will be assessed in the period of time prior to study enrollment (5 years for all screening modalities and at any time for colonoscopies), during the first 12 months, 24 months and 36 months after the MCED test is assessed. The analysis will focus on the cancer screening tests with grade A or B recommendations from USPSTF (Appendix 12). The number of men who have ever received screening for prostate cancer will be reported. However, prostate cancer screening will not be included in the analyses described below because the USPSTF makes no recommendation for or against routine provision of this screening test (grade C recommendation), and the recommendation for periodic prostate-specific antigen (PSA)-based screening is intended to be made on an individual basis after discussion of benefits and harms with clinician.

Colorectal cancer screening for participants aged 76 years and older will also not be included in these analyses because the USPSTF makes no recommendation for or against routine provision of this screening test (grade C recommendation), and suggest that decision to screen should be made on an individual basis in consideration of health status.

Based on the information about cancer screening for a given cancer prior to study enrollment, participants eligible for screening per USPSTF grade A or B recommendations with be characterized at the time of enrollment as:

- Up to date with their recommended cancer screening (per USPSTF screening recommendations in Appendix 12). “Up to date” is defined as being within a recommended cancer screening interval + 90 days from the date of the last reported cancer screening.
- Not up to date with their recommended cancer screening
- Number of of participants with at least one cancer screening test prior to enrollment will be reported overall and by type of test.

The definition of being up to date with recommended SOC cancer screening tests will be also applied throughout the analysis period. The following information will be reported for cancers with USPSTF Grade A or B recommendations by cancer type and overall:

- Number of cancers diagnosed in all study participants
    - Number of interval cancers[19] (cancers diagnosed while a participant is considered to be up to date with their recommended SOC cancer screening test for a given cancer during the analysis period)
    - Number of cancers diagnosed in participants who are not considered to be up to date with their recommended SOC cancer screening test for a given cancer during the analysis period
- Number of cancers diagnosed in participants with MCED-I positive test results

[19] Farber, Rachel, Nehmat Houssami, Isabelle Barnes, Kevin McGeechan, Alexandra Barratt, and Katy J. L. Bell. 2022. "Considerations for Evaluating the Introduction of New Cancer Screening Technology: Use of Interval Cancers to Assess Potential Benefits and Harms" International Journal of Environmental Research and Public Health 19, no. 22: 14647. https://doi.org/10.3390/ijerph192214647

CONFIDENTIALITY
This document is the confidential and proprietary information of GRAIL, LLC. Except as required by federal, state, or local laws or regulations, this document may not be disclosed to others, in whole or in part without prior written permission of GRAIL, Inc.

- Number of interval cancers (cancers diagnosed while a participant is considered to be up to date with their recommended SOC cancer screening test for a given cancer during the analysis period)
- Number of cancers diagnosed in participants who are not considered to be up to date with their recommended SOC cancer screening test for a given cancer during the analysis period

Based on utilization of cancer screening for a given cancer during the analysis period after MCED testing, participants eligible for screening at enrollment will be characterized as:

- Due for cancer screening during the analysis period
- Due for cancer screening during the analysis period and had at least one cancer screening test during the analysis period
- Up to date with their recommended cancer screening at the end of the analysis period
- Not up to date with their recommended cancer screening at the end of the analysis period

The utilization of cancer screening during the analysis period after MCED testing will be assessed for:

- All analyzable participants
- Participants who were up to date with their recommended screening at the time of enrollment
- Participants who were not up to date with their recommended screening at the time of enrollment

The utilization of cancer screening prior to enrollment and during the analysis period after MCED testing will be assessed in participants with MCED-V2 test negative results (the main focus of this analysis), those with MCED-V2 test positive results and in all participants.

In addition, the utilization of cancer screening tests during the analysis period after MCED testing will be compared to utilization prior to enrollment descriptively.

Cancer screening rates declined sharply in March through May of 2020, during the first peak of COVID-19 incidence, followed by a “return to normal or near-normal levels” in the summer of that year[20,21]. A subsequent surge in COVID-19 incidence in Jan-Mar 2022 seems to have had a low impact on the cancer screening rates[21]. PATHFINDER 2 study enrollment started in December 2021, which is more than 18 months after the start of the COVID-19 pandemic, so we expect the initial rise in COVID-19 incidence to have limited, if any, impact on our evaluation of cancer screening utilization prior to enrollment. As an exploratory analysis, we will examine the patterns of cancer screening utilization over time before and after the start of the COVID-19 pandemic where sufficient data are available.

---

[20] Doan C et al, Breast and Lung Cancer Screening Among Medicare Enrollees During the COVID-19 Pandemic, JAMA Netw Open. 2023 Feb; 6(2)
[21] Chen RC et al, Association of Cancer Screening Deficit in the United States With the COVID-19 Pandemic, *JAMA Oncol.* 2021;7(6):878-884

CONFIDENTIALITY
This document is the confidential and proprietary information of GRAIL, LLC. Except as required by federal, state, or local laws or regulations, this document may not be disclosed to others, in whole or in part without prior written permission of GRAIL, Inc.

14.3.1.2 Assess Participants Reported Anxiety Resulting from Use of the MCED Test

Participant reported anxiety is assessed using STAI (6 items) and MCED STAI (3 items) questionnaires in all participants at baseline (pre-test), post-test and at 12 months. In addition, MCED test positives will be assessed at diagnostic resolution (Appendix 2).

For the STAI (6 item) questionnaire, descriptive statistics will be provided for individual item responses on a 4-point scale (i.e., Not at all, Somewhat, Moderately, Very much so) and a total score calculated according to the STAI manual[22] at each time point. In addition, change in the total score from baseline at post-test, diagnostic resolution and 12 months will be assessed. A two-sided t-test will be used to determine if mean changes from baseline are significantly different from zero at each post-baseline assessment at 0.05 significance level. If differences in scores do not follow a normal distribution, Wilcoxon signed rank test[23] will be used instead. No adjustment for multiple testing is planned.

In the STAI user manual, mean scores were 32-34 (SD=8-10) for men and women aged 50-69 years, 42.38 (SD=13.79) for patients undergoing general, medical and surgical procedures and 49.02 (SD:11.67) for those with anxiety disorders. STAI scores are commonly classified as "no or low anxiety" (20–37), "moderate anxiety" (38–44) , and "high anxiety" (45–80)[24]. Distribution of these anxiety categories will be summarized at each timepoint. Change from baseline in the proportion of participants with 'high anxiety' will be assessed at each timepoint. Descriptive statistics will be provided for STAI standardized total scores for each timepoint.

In addition, distribution of the individual responses to item 6 ("I am worried") will be summarized at each timepoint. Individual item responses for baseline vs post-baseline assessment will be cross-tabulated as a 4 x 4 matrix. Change from baseline in the proportion of participants with "Moderately" or "Very much so" responses assessed at each timepoint.

For MCED STAI (3 item) questionnaire, descriptive statistics will be provided for individual item responses at each timepoint. Individual item responses for baseline vs post-baseline assessment will be cross-tabulated. Change from baseline in the proportion of participants with "Moderately" or "Very much so" responses to the question "I am worried because of my multi-cancer early detection test" will be assessed at each timepoint.
The results will be summarized for:

- All MCED-V2 analyzable participants
- Participants with MCED-V2 test positive and negative results, separately
- Participants with MCED-V2 test positive results with and without cancer diagnosis at diagnostic resolution, separately.

---

[22] Spielberger C.D., 1983. Manual for the State-Trait Anxiety Inventory STAI (Form Y). Palo Alto, CA: Consulting Psychologists Press
[23] Wilcoxon, F (1945) Individual comparisons by ranking methods. Biometrics 1:80–83
[24] Millar, K., Jelilic, M., et al. 1995. Assessment of preoperative anxiety: comparison of measures in patients awaiting surgery for breast cancer. British Journal of Anaesthesia

CONFIDENTIALITY
This document is the confidential and proprietary information of GRAIL, LLC. Except as required by federal, state, or local laws or regulations, this document may not be disclosed to others, in whole or in part without prior written permission of GRAIL, Inc.

14.3.1.3 Assess participants' reported outcomes and perceptions about the MCED test

Analysis methods

Participants' reported outcomes and perceptions will be assessed using participant-directed questionnaires evaluated at different time points during the study (see Appendix 2). Description of each questionnaire and scoring mechanism is provided in Appendix 13. Missing responses will be handled according to the corresponding scoring manual for each questionnaire.

The results will be reported for all participants with at least one analyzable score for a given questionnaire for the following participant groups:

- MCED-V2 Analyzable Set
- MCED-V2 Cancer Signal Detected Set
    - MCED-V2 Cancer Signal Detected and cancer diagnosis at diagnostic resolution
    - MCED-V2 Cancer Signal Detected and no cancer diagnosis at diagnostic resolution
- MCED-V2 No Cancer Signal Detected Set

Completion rates for each questionnaire at each time point will be reported.

For SF-12v2, a two-sided t-test will be used to determine if mean changes from baseline are significantly different from zero at each post-baseline assessment at 0.05 significance level. If differences in scores do not follow a normal distribution, Wilcoxon signed rank test will be used instead. No adjustment for multiple testing is planned.

14.3.1.3.1 Adapted MICRA

Adapted MICRA is assessed in all participants post-MCED test only. Descriptive statistics for the following scale scores will be provided:

- Distress scale
- Uncertainty scale
- Positive experience scale
- Total score

14.3.1.3.2 SF-12v2

Health-related quality of life is assessed in all participants at baseline (pre-test), post-test, at diagnostic resolution (MCED-V2 test positive only), and at 12 months (Appendix 2). Descriptive statistics will be provided for the scale scores derived from SF-12v2 (as described in Appendix 13) at each time point and change from baseline for each component summary measure:

- Mental component summary
- Mental health
- Role-emotional
- Social functioning

***14.3.1.3.3* Satisfaction with MCED Test**

Satisfaction with the MCED test is assessed post-test in MCED-V2 test negative participants and

CONFIDENTIALITY

This document is the confidential and proprietary information of GRAIL, LLC. Except as required by federal, state, or local laws or regulations, this document may not be disclosed to others, in whole or in part without prior written permission of GRAIL, Inc.

at diagnostic resolution in MCED-V2 test negative participants (Appendix 2). Descriptive statistics will be provided for a total score across all questions and individual ratings for each question.

14.3.1.3.4 Attitude Towards Subsequent MCED Test

Attitude towards subsequent MCED testing is assessed post-test in MCED-V2 test negative participants and at diagnostic resolution in MCED-V2 test negative participants (Appendix 2). Responses to the question "How likely are you to undergo a subsequent multi-cancer early detection test?" were represented by a 4-point Likert-type scale (i.e.Very Unlikely, Unlikely, Likely, and Very Likely). Number and proportion of participants with each rating will be reported.

14.3.2 Diagnostic evaluations

14.3.2.1 Characterize the number and type of diagnostic evaluations by predicted cancer signal origin and outcome of diagnostic resolution

The number and type of diagnostic evaluations triggered by MCED-V2 positive test results in participants with a positive MCED test result will be summarized for the following four categories:

1. Imaging tests
    a. With radiation
    b. Without radiation
    c. Whole body imaging defined as PET-CT, whole body MRI or CT that includes all three imaging fields (chest, abdomen and pelvis)
2. Laboratory tests (including blood- and urine-based tests)
3. Non-invasive procedures (including physical exam)
4. Invasive procedures as described in Appendix 10
    a. Non-surgical invasive procedures
    b. Surgical invasive procedures

The following will be summarized:

- The number of participants with at least one diagnostic evaluation from each of the four categories described above will be reported overall and by type
- The total number of diagnostic evaluations from each of the four categories will be reported overall and by type
- The distribution of the per-participant counts for each of the four categories will be summarized using descriptive statistics.

Similar to Section 14.2.1.2, these analyses will be reported for the following groups of participants:

- Cancer vs no cancer diagnosis
    - Participants with cancer diagnosis (TP)
        - Participants with cancer diagnosis at diagnostic resolution
        - Participants with no cancer diagnosis at diagnostic resolution who have a cancer diagnosis at the end of the analysis period
    - Participants with no cancer diagnosis (FP)

CONFIDENTIALITY
This document is the confidential and proprietary information of GRAIL, LLC. Except as required by federal, state, or local laws or regulations, this document may not be disclosed to others, in whole or in part without prior written permission of GRAIL, Inc.

        - Participants with no cancer diagnosis at diagnostic resolution and no cancer diagnosis at the end of the analysis period
        - Participants who did not reach a diagnostic resolution and so do not have a cancer diagnosis at the end of the analysis period

- Initial vs subsequent diagnostic evaluation
    - Participants with an invasive procedure performed as part of their initial diagnostic evaluation (e.g., the first test(s) completed to evaluate a positive MCED-V2 test result)
    - Participants with an invasive procedure performed after initial diagnostic evaluation (e.g., test(s) ordered after review of results from initial diagnostic evaluation)

- CSO guided vs not CSO guided diagnostic evaluation
    - CSO guided diagnostic evaluation based on the diagnostic evaluation including imaging tests, laboratory tests,non-invasive procedures and invasive procedures: participants with the diagnostic evaluations consistent with the recommended targeted diagnostic testing for predicted CSO(s) as described in the study protocol (Appendix 11).
    - Not CSO guided diagnostic evaluation based on the diagnostic evaluation including imaging tests, laboratory tests,non-invasive procedures and invasive procedures: participants with diagnostic evaluations inconsistent with the recommended targeted diagnostic testing for predicted CSO(s)

In addition, the extent of diagnostic testing will be assessed in the following subgroups:

- CSO prediction groups (solid tumors, hematologic malignancies)
- CSO prediction, where sample size allows (e.g. number of CSO calls ≥ 5)
- Number of reported CSOs (one vs two) for MCED-V2 only

The time required to achieve diagnostic resolution is defined as the number of days from the date when the MCED-V2 test result is communicated to the participant to the date of diagnostic resolution; it will be summarized using descriptive statistics by outcome of diagnostic resolution. If some participants did not reach a diagnostic resolution at the end of the analysis period, Kaplan-Meier methods will be used to estimate the time to diagnostic resolution.

The extent of diagnostic testing will be summarized for:

- All diagnostic evaluations prior to diagnostic resolution,
- The diagnostic evaluations starting from the diagnostic PET-CT performed after the initial negative diagnostic evaluation (referred to as the ‘confirmatory PET-CT’ in the study protocol) (inclusive) and conducted until diagnostic resolution in participants who had such diagnostic PET-CT

CONFIDENTIALITY
This document is the confidential and proprietary information of GRAIL, LLC. Except as required by federal, state, or local laws or regulations, this document may not be disclosed to others, in whole or in part without prior written permission of GRAIL, Inc.

14.3.2.2. Evaluate the radiation exposure due to the test result-directed evaluation

14.3.2.2.1 Evaluating radiation exposure for MCED-V2 test positive participants

The number and type of imaging procedures with radiation exposure will be reported for participants with diagnostic evaluations triggered by MCED-V2 test positive results. To quantify radiation exposure for a participant as a result of their diagnostic workup, the radiation dose for each imaging test will be assessed based on the effective radiation dose estimates in Appendix 14.

The total amount of radiation exposure and the extent of radiation exposure on a per-participant basis will be summarized using descriptive statistics across:

- All MCED-V2 test positive participants
- Participants with MCED-V2 test positive results and cancer diagnosis at diagnostic resolution
- Participants with MCED-V2 test positive results and no cancer diagnosis at diagnostic resolution
- Participants with MCED-V2 test positive results who did not reach diagnostic resolution during the analysis period

The results will be reported for:

- All imaging tests performed prior to diagnostic resolution
- Imaging tests performed after diagnostic PET-CT (including confirmatory PET-CT, as referred to in the study protocol) and until diagnostic resolution

14.3.2.2.2 Evaluate radiation exposure for MCED-I test positive participants

The number and type of imaging procedures with radiation exposure for MCED-I test positive participants will be summarized using methods described in Section 14.2.1.3.

The total amount of radiation exposure and the extent of radiation exposure on a per-participant basis will be estimated as described in Section 14.3.2.2.1.

14.3.2.3 Describe the ability of diagnostic PET-CT to confirm cancer in participants where diagnostic resolution of cancer diagnosis is not achieved through the targeted diagnostic testing triggered by MCED test results

The ability of the PET-CT to detect cancer in cases for which CSO-guided workups do not result in diagnosis of cancer will be evaluated using the following endpoints:

1. Proportion of participants with cancer diagnosis at diagnostic resolution out of all participants who had a PET-CT only after initial negative diagnostic evaluation (no PET-CT during initial diagnostic evaluation)
2. Proportion of participants with cancer diagnosis at diagnostic resolution out of all

CONFIDENTIALITY
This document is the confidential and proprietary information of GRAIL, LLC. Except as required by federal, state, or local laws or regulations, this document may not be disclosed to others, in whole or in part without prior written permission of GRAIL, Inc.

participants with initial negative diagnostic evaluation who had a diagnostic PET-CT at any time during diagnostic evaluation

The distribution of cancer types, stage for new cancers and extent of recurrence for recurrent cancers among cancers detected after diagnostic PET-CT will be reported.

14.3.3 Test performance

14.3.3.1 Evaluate Performance of the MCED Test in Selected Demographic and Clinical Subgroups.

Evaluation of MCED Test Performance in Selected Demographic and Clinical Subgroups

The following test performance endpoints will be evaluated:

- Cancer signal detection
  - PPV, NPV
  - Episode sensitivity, specificity
  - PLR, NLR
- CSO prediction
  - Accuracy of CSO1 prediction in TP
- Combined cancer signal detection and CSO1 prediction
  - PPV accounting for CSO accuracy
  - Episode sensitivity accounting for CSO accuracy

Selected Demographic and Clinical Subgroups

Test performance for MCED-V2 test and MCED-I test will be summarized descriptively using endpoints described above in the following subgroups:

1. Age: 50-64 vs 65+ years
2. Age: 50-59, 60-69,70-79, 80+ years
3. Biological sex: male, female
4. Race categories for full representation, i.e. if reported alone or as part of multiple race categories (not mutually exclusive):
   - American Indian or Alaska Native (including North American Indigenous)
   - Asian, Native Hawaiian or Other Pacific Islander
   - Black or African American
   - White (including Middle Eastern or North African)
5. Ethnicity (Hispanic or Latino vs not)
6. BMI: <25 kg/m^2, 25 to <30 kg/m^2, ≥30 kg/m^2
7. Smoking: never smoker, ever smoker
8. Prior cancer history: yes, no

Evaluation of MCED Test Performance in Specific Cancer Subtypes

CONFIDENTIALITY
This document is the confidential and proprietary information of GRAIL, LLC. Except as required by federal, state, or local laws or regulations, this document may not be disclosed to others, in whole or in part without prior written permission of GRAIL, Inc.

A descriptive summary of the distribution of the following cancer subtypes will be provided. In addition, the episode sensitivity for MCED-V2 and MCED-I will be summarized descriptively.

- Breast
  - Estrogen-receptor (ER) and progesterone-receptor (PR) negative and human epidermal growth factor receptor 2 (HER2) negative ('triple negative')
  - ER/PR positive and HER2 negative (‘hormone receptor positive’)
  - HER2 positive (regardless of ER/PR status)

- Lung
  - Small cell lung cancer
  - Adenocarcinoma non-small cell lung cancer
  - Squamous cell carcinoma non-small cell lung cancer
  - Other non-small cell lung cancer (includes Large cell carcinoma and NOS)

- Prostate (based on Gleason Grade Group[25])
  - Gleason Grade Group 1
  - Gleason Grade Group 2
  - Gleason Grade Group 3-5

14.3.3.2 Evaluate MCED Test Positive Rate in Subgroups with Potential Cross-Reactive Conditions

Endpoint and Subgroups

MCED test positive rate is defined as the proportion of participants with MCED positive test results out of all participants with analyzable MCED test results.

MCED test positive rate will be evaluated in participants with the following conditions:

1. Non-malignant hematologic conditions (Langerhans cell histiocytosis, Lymphoproliferative disorders, Mastocytoma, Monoclonal B-cell lymphocytosis, Monoclonal gammopathy of undetermined significance, Post-transplant lymphoproliferative disorder, other)[26]
2. Participants who took any cytotoxic medications in the past year
3. Participants who took any demethylating agents in the past year
4. Participants with a prior cancer history
5. Conditions described in Table 9 which may shed cfDNA and/or may affect methylation

[25] Grade Group 1 (Gleason score ≤ 6), Grade Group 2 (Gleason score 3+4), Grade Group 3 (Gleason score 4+3), Grade Group 4 (Gleason score 8), Grade Group 5 (Gleason score 9-10) https://journals.lww.com/ajsp/abstract/2016/02000/the_2014_international_society_of_urological.10.aspx

[26] Answered “Yes” to the question “Has the participant ever been diagnosed with any of the following hematologic conditions prior to study enrollment?”

CONFIDENTIALITY
This document is the confidential and proprietary information of GRAIL, LLC. Except as required by federal, state, or local laws or regulations, this document may not be disclosed to others, in whole or in part without prior written permission of GRAIL, Inc.

Table 9. Medical conditions to be evaluated for cross-reactivity

Condition[27]	Description of condition
Autoimmune disease	Multiple sclerosis, Rheumatoid arthritis, Systemic lupus erythematosus (lupus), Crohn's disease, ulcerative colitis
Chronic lung disease	Chronic obstructive pulmonary disease (COPD), emphysema, chronic bronchitis
HIV Infection	HIV infection
Pancreatitis	Acute pancreatitis, Chronic pancreatitis
Chronic liver disease	Chronic hepatitis B infection, Chronic hepatitis C infection, Cirrhosis (Liver cirrhosis), Fatty liver disease
Non-malignant gastrointestinal conditions	Barrett's esophagus, Chronic gastritis, Esophageal reflux disease, Stomach ulcers
Diabetes	Diabetes
Transplantation	Solid organ or stem cell (bone marrow) transplant

Analysis Methods

A Pearson chi-square test will be used to assess if participants with any of the potential cross-reactive conditions have a significantly different MCED test positive test rate compared to the MCED test positive rate in participants without the condition. If any condition is found to have a significantly higher MCED test positive rate, an estimate of the impact on false positives and specificity will be assessed based on the prevalence of these conditions in the intended use population.

This analysis will be completed for MCED-V2 test and MCED-I test.

14.3.3.3 Evaluate concordance of initial GRAIL test result and CSO prediction, with results from the research blood draw

The results of the MCED-I test from the blood draw at enrollment (initial blood draw) and research blood draw at the time of the diagnostic PET-CT will be compared in participants who had both

[27] Answered "Yes" to "Please confirm if a doctor has ever told the participant that they have any of the following health/medical conditions (check all that apply)."

CONFIDENTIALITY
This document is the confidential and proprietary information of GRAIL, LLC. Except as required by federal, state, or local laws or regulations, this document may not be disclosed to others, in whole or in part without prior written permission of GRAIL, Inc.

blood draws. It should be noted that research blood draws can be obtained only in participants with positive initial MCED-V2 test results. The number of negative and positive MCED results from research blood draw will be tabulated. In addition, CSO predictions will be compared for initial vs research blood draws and proportion of cases with the same CSO among participants with positive MCED results from both the initial and research blood draws will be reported.

14.4 Analysis Methods for Exploratory Objectives

14.4.1 Evaluate performance of the MCED test over three years of follow-up.

MCED test performance endpoints for overall cancer signal detection and overall CSO accuracy (as described in Sections 14.2.2.1.1 and 14.2.2.1.2) will be assessed based on cancer status after 3 years (36 months) or less of follow-up time for both test versions. The distribution of cancer types and stages for cancers reported in the last 12 months of study follow-up will be summarized for participants with MCED test positive results and MCED test negative test results.

14.4.2 Evaluate changes in blood-based biomarkers among the subset of participants with a research blood draw.

Blood biomarker signals from the initial and research blood draws will be assessed in the subset of participants with a research blood draw.

14.4.3 Evaluate different CSO reporting classes based on clinically relevant subgroups.

MCED test results for CSO prediction and blood biomarker signals from the initial and research blood draws will be evaluated in the context of diagnostic testing triggered by the test results.

14.4.4 Evaluate DNA based and other biomarkers in tissue compared to blood from cancer cases.

DNA based and other biomarkers will be evaluated in tissue and blood samples in participants with cancer.

15. Analysis Methods for Adverse Events

The number of adverse events (AEs) observed in participants in the Consented Analysis set (as described in Section 10.1) will be summarized. The number of AEs in participants with MCED-positive, MCED-negative, no analyzable test results and all consented participants will be characterized by the following:

- Setting (phlebotomy, anticipation or communication of MCED test result, diagnostic procedure prompted by a positive MCED test result, other setting)
- Seriousness (whether AE was considered an SAE)
    - If yes, seriousness criteria (death, life threatening, required hospitalization-initial or prolonged, results in persistent or significant disability/incapacity, required intervention to prevent permanent impairment, congenital anomaly/birth defect,

CONFIDENTIALITY
This document is the confidential and proprietary information of GRAIL, LLC. Except as required by federal, state, or local laws or regulations, this document may not be disclosed to others, in whole or in part without prior written permission of GRAIL, Inc.

other medically important serious event)

- Severity (mild, moderate, severe)
- Relatedness to study activities (i.e., study activities listed in the protocol Schedule of Activities) or device
- Outcome (e.g. recovered/ resolved, recovered/ resolved with sequelae, recovering/ resolving, not recovered/ not resolved, fatal)

AEs will be described separately for participants with MCED-positive, MCED-negative, no analyzable test results and all consented participants for the following subsets:

- Related to study activities, related to device
- SAEs

A per-participant listing of SAEs will be provided.

16. Protocol Deviations

Protocol deviations that occur after participants entered the study are documented during routine monitoring.

Important protocol deviations are a subset of protocol deviations that might significantly affect the completeness, accuracy, and/or reliability of the study data or that might significantly affect participant's rights, safety, or well-being[28].

Important protocol deviations will be summarized for all PATHFINDER 2 participants for the following categories:

- Participants who had at least one important protocol deviation
- Participants who did not meet at least one eligibility criterion but were enrolled into the study
- Participants whose informed consent form deviated from protocol version.

A per-participant listing of important protocol deviations will also be provided.

17. Sample Size and Power

The expected number of positive test results, number of cancers diagnosed, number of cancers detected through diagnostic evaluation triggered by the MCED test in analyzable participants and test performance endpoints were estimated using microsimulations[29].

---

[28] FDA (2013). *E3 Structure and Content of Clinical Study Reports - Questions and Answers (R1)*. Retrieved https://www.fda.gov/downloads/Drugs/GuidanceComplianceRegulatoryInformation/Guidances/UCM336889.pdf

[29] Dai JY et al, 'Clinical performance and utility: A microsimulation model to inform the design of screening trials for a multi-cancer early detection test', Journal of Medical Screening OnlineFirst, https://journals.sagepub.com/doi/full/10.1177/09691413241228041

CONFIDENTIALITY

This document is the confidential and proprietary information of GRAIL, LLC. Except as required by federal, state, or local laws or regulations, this document may not be disclosed to others, in whole or in part without prior written permission of GRAIL, Inc.

Overall Expected Results

The results of the microsimulations in Table 10 describe the number of participants with MCED positive test results expected among 25,000 enrolled participants (23,750 analyable participants assuming 5% exclusions) with 12 months of follow-up for the PMA analysis. The results are provided for two scenarios: based on specificity of 99.0% and 99.5%. In the scenario with 99.5% specificity, a total of 218 MCED positive test results are expected with approximately 102 TP and 116 FP (47% PPV). Under conservative assumptions about cancer incidence used in these microsimulations to account for a possible healthy volunteer effect (assuming that underlying cancer incidence in the enrolled population is 65% of SEER cancer incidence with sex and age distribution described in the study protocol) we expect 244 cancers diagnosed among 25,000 enrolled participants during 12 months follow-up time. Note, that the microsimulation accounts for 17 cancer types only (cancer types for which dwell time scenarios have been developed and which have sufficient stage data in SEER to support modeling); if cancers not included in the simulation are taken into account, we estimate the total number of cancers to be 264 or higher. Table 10 provides expected estimates of PPV, NPV and episode sensitivity for all cancers.

Table 10. Results of Microsimulations for 25,000 enrolled participants

Specificity	Number of Test Positive Results (95% CI)	% Test Positive (95% CI)	PPV (%) (95% CI)*	NPV (%) (95% CI)*	Episode Sensitivity (%) (95% CI)*
99.0%	335 (304,365)	1.41 (1.28, 1.54)	30.1 (25.4, 35.1)	99.4 (99.3-99.5)	41.5 (35.5, 47.3)
99.5%	218 (193, 248)	0.92 (0.81, 1.04)	47.0 (40.3, 53.2)	99.4 (99.3-99.5)	42.2 (36.9, 48.1)

*Entries in the table represent median (2.5 and 97.5 percentiles) of a specific measure across 200 simulations

Using similar logic, a total of approximately 370 cancers are expected to be diagnosed among 35,000 enrolled participants (or 33,250 analyable participants) during 12 months of follow-up time.

Power Calculations for Acceptance Criteria

The acceptance criteria for PATHFINDER 2 study are described in Section 14.2.2.2. Power calculations for specificity, taking sampling into account, are described in Appendix 9. Power calculations for acceptance criteria based on episode sensitivity for prespecified cancer subgroups, and accuracy of CSO prediction in participants with TP MCED test results are provided in Table 11 and Table 12, respectively. As described above, a total of 264 cancers are expected to be diagnosed among 25,000 participants with 12 months of follow-up at the time of the PMA analysis. Power calculations were conducted using the Wilson method to compute two-sided 95% confidence intervals based on 10,000 simulations using binomial distribution at expected sample sizes for each endpoint. The power was determined as the proportion of the simulated results where the lower bound of the 95% confidence interval exceeded the pre-specified acceptance criteria threshold for the corresponding endpoint.

CONFIDENTIALITY

This document is the confidential and proprietary information of GRAIL, LLC. Except as required by federal, state, or local laws or regulations, this document may not be disclosed to others, in whole or in part without prior written permission of GRAIL, Inc.

Table 11. Power calculations for episode sensitivity acceptance criteria

Cancer Group*	Acceptance Criteria Threshold	Potential Episode Sensitivity Estimate	Power
Cancers responsible for ⅔ of all cancer deaths in the US (N=149)	22%	30%	65%
		40%	>99%
		50%	>99%
Cancers without common screening options (N=157)	19%	30%	91%
		40%	>99%
		50%	>99%
All cancers (N=264)	10%	20%	99%
		30%	>99%
		40%	>99%

*See Appendix 7 for cancer types included in each group

Table 12. Power calculations for acceptance criterion for the accuracy of CSO prediction in participants with a true positive MCED test result

Acceptance Criteria Threshold for the accuracy of CSO prediction	Potential CSO accuracy Estimate*	Power
70%	75%	16%
	80%	54%
	85%	91%
	90%	>99%

*Based on conservative estimate of having at least 86 expected true positive participants

CONFIDENTIALITY
This document is the confidential and proprietary information of GRAIL, LLC. Except as required by federal, state, or local laws or regulations, this document may not be disclosed to others, in whole or in part without prior written permission of GRAIL, Inc.

APPENDIX 1 PATHFINDER 2 INCLUSION/EXCLUSION CRITERIA

Inclusion and exclusion criteria below are from the "The PATHFINDER 2 Study: Evaluating the Safety and Performance of the GRAIL Multi-Cancer Early Detection Test in an Eligible Screening Population" Protocol Version Number: 3.0, dated 09Aug2023. Any modifications to these criteria will be reflected in the clinical study report. Participants are eligible to be included in the study only if all of the following criteria apply:

Inclusion Criteria

Participants are eligible to be included in the study only if all of the following criteria apply.

1. Participants must be at least 50 years of age, inclusive, at the time of signing the Informed Consent Form (ICF).
2. Informed Consent
    - Capable of giving signed and legally effective informed consent as described in Section 5.4, Study Design Schema, which includes compliance with the requirements and restrictions listed in the Informed Consent Form (ICF) and in this protocol. Consent provided by a legally authorized representative is not permitted in this protocol.

Exclusion Criteria

1. Undergoing or referred for diagnostic evaluation due to clinical suspicion for cancer (e.g., referred to a medical or surgical oncologist, or scheduled for biopsy on the basis of a suspicious imaging abnormality).
2. Personal history of invasive solid tumor or hematologic malignancy, diagnosed within the 3 years prior to expected enrollment date, or diagnosed greater than 3 years prior to expected enrollment date and never treated.
    - Individuals with a diagnosis of non-metastatic basal cell carcinoma and squamous cell carcinoma of the skin are not excluded.
3. Prior/Concurrent Concomitant Therapy (Medications/Treatments):
    - Definitive treatment for invasive solid tumor or hematologic malignancy within the 3 years prior to expected enrollment date. Adjuvant hormone therapy for cancer (e.g. for breast or prostate cancer) is not an exclusion criterion.
4. Individuals who will not be able to comply with the protocol procedures.
5. Individuals who are not currently registered patients at a participating center.
6. Previous or current participation in another GRAIL-sponsored study. "Participation" is defined as having signed consent and provided a blood sample.
7. Previous or current employees or contractors of GRAIL.
8. Current pregnancy (by self-report of pregnancy status)

CONFIDENTIALITY
This document is the confidential and proprietary information of GRAIL, LLC. Except as required by federal, state, or local laws or regulations, this document may not be disclosed to others, in whole or in part without prior written permission of GRAIL, Inc.

Appendix 2 Participant-reported outcome data collection time points

Instrument	Pre-Test	Post-Test	Post-Test	At Diagnostic Resolution	12 months
Participant Group	All	No Signal Detected	Signal Detected	Signal Detected	ALL
SF-12v2 Health Survey	X	X	X	X	X
Multidimensional Impact of Cancer Risk Assessment (Adapted MICRA)		X	X		
State Trait Anxiety Inventory (STAI)+ MCED STAI questionnaires	X	X	X	X	X
Satisfaction with the MCED test		X		X	
Attitude towards adherence to guideline-recommended screening	X	X			X
Attitude towards subsequent MCED test		X		X	

CONFIDENTIALITY
This document is the confidential and proprietary information of GRAIL, LLC. Except as required by federal, state, or local laws or regulations, this document may not be disclosed to others, in whole or in part without prior written permission of GRAIL, Inc.

APPENDIX 3 CSO PREDICTIONS FOR THE MCED-V2 TEST

N	CSO predictions as displayed on the Test Result Report
1	Anus
2	Bladder, Urothelial tract
3	Breast
4	Bone and Soft tissue
5	Cervix
6	Colon, Rectum
7	Head and Neck
8	Kidney
9	Liver, Bile duct
10	Lung
11	Lymphoid Lineage
12	Melanocytic Lineage
13	Myeloid Lineage
14	Neuroendocrine cells of lung or other organs
15	Ovary
16	Pancreas, Gallbladder
17	Plasma Cell Lineage
18	Prostate
19	Stomach, Esophagus
20	Thyroid gland
21	Uterus

* Neuroendocrine cancers include

- lung neuroendocrine cancers (low grade or high grade)
- high grade neuroendocrine tumors for: bladder, cervix, colon/rectum, esophagus, head and neck, gallbladder, stomach, pancreas, prostate, urothelial tract, uterus

CSOs are mutually exclusive so the endocrine tumors included in “Neuroendocrine” CSO are excluded from other CSOs. For example, high grade neuroendocrine bladder cancer will be included in the “Neuroendocrine” CSO and not be included in the “Bladder / Urothelial tract” CSO.

CONFIDENTIALITY
This document is the confidential and proprietary information of GRAIL, LLC. Except as required by federal, state, or local laws or regulations, this document may not be disclosed to others, in whole or in part without prior written permission of GRAIL, Inc.

APPENDIX 4 PROVISIONAL CSO PREDICTIONS FOR THE MCED-I TEST

Note: any changes to the displayed names will be documented in the clinical study report.

N	CSO predictions
1	Anus
2	Bladder or Urothelial tract
3	Blood, Lymphatic system, or Bone marrow
4	Bone or Soft tissue
5	Breast
6	Cervix
7	Colon or Rectum
8	Head and Neck
9	Kidney
10	Liver or Intrahepatic bile duct
11	Lung
12	Ovary or Fallopian tubes
13	Pancreas, Extrahepatic bile duct, or Gallbladder
14	Prostate
15	Skin
16	Stomach or Esophagus
17	Thyroid
18	Uterus

CONFIDENTIALITY
This document is the confidential and proprietary information of GRAIL, LLC. Except as required by federal, state, or local laws or regulations, this document may not be disclosed to others, in whole or in part without prior written permission of GRAIL, Inc.

Appendix 5 MCED-I and MCED-V2 CSO Predictions

Appendix 5A: Crosstabulation of MCED-I CSO Predictions and Corresponding MCED-V2 CSO Predictions

N	MCED-I CSO	MCED-V2 CSO
1	Anus	Anus
2	Bladder or Urothelial tract	Bladder, Urothelial Tract
3	Blood, Lymphatic system, or Bone marrow	Lymphoid Lineage
		Myeloid Lineage
		Plasma Cell Lineage
4	Bone or Soft Tissue	Bone and Soft Tissue
5	Breast	Breast
6	Cervix	Cervix
7	Colon or Rectum	Colon, Rectum
8	Head and Neck	Head and Neck
9	Kidney	Kidney
10	Liver, intrahepatic Bile duct	Liver, Bile Duct
11	Lung	Lung
12	Ovary or Fallopian Tubes	Ovary
13	Pancreas, extrahepatic Bile ducts, or Gallbladder	Pancreas, Gallbladder
14	Prostate	Prostate
15	Skin	Melanocytic Lineage
16	Stomach or Esophagus	Stomach, Esophagus
17	Thyroid	Thyroid Gland
18	Uterus	Uterus

Note: MCED-V2 has an additional CSO of "Neuroendocrine Cells of Lung or other Organs" which has no corresponding MCED-I CSO. Instead, the classification algorithm will redistribute cases with this MCED-V2 CSO to MCED-I CSO for each respective organ or organ system affected by cancer.

CONFIDENTIALITY

This document is the confidential and proprietary information of GRAIL, LLC. Except as required by federal, state, or local laws or regulations, this document may not be disclosed to others, in whole or in part without prior written permission of GRAIL, Inc.

Appendix 5B: Matrix of MCED-I CSO Predictions and Corresponding MCED-V2 CSO Predictions among test positives for both tests

MCED-I CSO1	MCED-V2 CSO1																				
	Anus	Bladder, Urothelial tract	Lymphoid Lineage	Myeloid Lineage	Plasma Cell Lineage	Bone and Soft Tissue	Breast	Cervix	Colon, rectum	Head, neck	Kidney	Liver, Intrahepatic bile duct	Lung	Neuroendocrine	Ovary	Pancreas, Gallbladder	Prostate	Melanocytic Lineage	Stomach, Esophagus	Thyroid	Uterus
Anus																					
Bladder, Urothelial tract																					
Blood, Lymphatic system, or Bone marrow																					
Bone and Soft Tissue																					
Breast																					
Cervix																					
Colon, Rectum																					
Head, Neck																					
Kidney																					
Liver, Intrahepatic bile duct																					
Lung																					
Ovary																					
Pancreas, Gallbladder																					
Prostate																					
Skin																					
Stomach or Esophagus																					

CONFIDENTIALITY

This document is the confidential and proprietary information of GRAIL, LLC. Except as required by federal, state, or local laws or regulations, this document may not be disclosed to others, in whole or in part without prior written permission of GRAIL, Inc.

MCED-I CSO1	MCED-V2 CSO1																				
	Anus	Bladder, Urothelial tract	Lymphoid Lineage	Myeloid Lineage	Plasma Cell Lineage	Bone and Soft Tissue	Breast	Cervix	Colon, rectum	Head, neck	Kidney	Liver, Intrahepatic bile duct	Lung	Neuroendocrine	Ovary	Pancreas, Gallbladder	Prostate	Melanocytic Lineage	Stomach, Esophagus	Thyroid	Uterus
Thyroid																					
Uterus																					

Note: MCED-V2 CSO of “Neuroendocrine Cells of Lung or other Organs” has no direct corresponding MCED-I CSO. Instead, the MCED-I classification algorithm will seek to predict the organ or organ system affected by neuroendocrine cancers.

CONFIDENTIALITY
This document is the confidential and proprietary information of GRAIL, LLC. Except as required by federal, state, or local laws or regulations, this document may not be disclosed to others, in whole or in part without prior written permission of GRAIL, Inc.

APPENDIX 6 MCED-I CSO SPECIFICATION

CSOs	Description	Examples for included cancers	Examples for excluded cancers
Anus	Carcinomas and other solid tumors of the anus and anal canal. The main contributor to this CSO is HPV-associated squamous cell carcinoma of the anus.	HPV-associated squamous cell carcinoma of the anus	Skin cancers reported in the anal region
		Anal gland adenocarcinoma	Extramammary Paget disease in this region
		HPV-positive squamous cell carcinoma reported with primary site rectum	Squamous cell carcinoma reported in the colon, even if HPV-positive
			Sarcoma or lymphoma reported in the anal region
Bladder or Urothelial tract	Carcinomas and other solid tumors of the bladder, ureter, and renal pelvis. Main contributors are transitional and urothelial carcinomas.	Adenocarcinoma of the bladder	Renal cell carcinomas reported at the renal pelvis as anatomic site
		Urothelial cell carcinoma of the bladder	Sarcoma or lymphoma reported in the bladder, ureter or renal pelvis
		Urothelial carcinoma of the renal pelvis	
		Carcinoma of the ureter	
		Small cell carcinoma of the bladder	
		Adenocarcinoma NOS in the renal pelvis	
		Urothelial carcinoma in the kidney	
Blood, lymphatic system, or bone marrow	Cancers of the blood, the lymphatic system, or bone marrow of lymphoid, myeloid, or plasma cell origin.	Lymphomas, including CNS lymphomas and MALT lymphomas of the GI tract	Hematologic conditions with behavior code other than 3 ICD-O-3
		Lymphoid leukemia	
		Myeloid leukemia	
		Multiple myeloma or plasma cell myeloma	
		Additional malignant hematologic conditions representing proliferative or dysplastic disorders or neoplasms with behavior code 3 based on ICD-O-3	
Bone or soft	Sarcomas and other cancers of	Uterine leiomyosarcoma	Myeloid Sarcoma

CONFIDENTIALITY
This document is the confidential and proprietary information of GRAIL, LLC. Except as required by federal, state, or local laws or regulations, this document may not be disclosed to others, in whole or in part without prior written permission of GRAIL, Inc.

CSOs	Description	Examples for included cancers	Examples for excluded cancers
tissue	mesenchymal origin in bone and soft tissue independent from anatomic site (includes brain and central nervous system)	Malignant solitary fibrous tumor	
		Osteosarcoma	
		Gastrointestinal stromal tumor	
		Malignant fibrous histiocytoma (of skin)	
		Malignant Hemangiopericytoma in the brain	
Breast	Cancers with a methylation signal and anatomic site most similar to carcinomas and other solid tumors in the breast. The main contributor to this CSO is invasive ductal carcinoma.	Invasive ductal breast carcinoma	Sarcoma or lymphoma reported in the breast
		Invasive lobular breast carcinoma	Any invasive skin cancer of the breast
		Breast cancer of no special type (NST)	Paget's disease
Cervix	Carcinomas and other solid tumors of the cervix. The main contributor to this CSO is HPV-associated squamous cell carcinoma of the cervix.	HPV-associated squamous cell carcinoma of the cervix	
		HPV-associated adenocarcinoma of the cervix	
		Neuroendocrine carcinoma of the cervix	
		Non-HPV-associated adenocarcinoma of the cervix	
Colon or Rectum	Carcinomas and other solid cancers of the colon and rectum. Colorectal adenocarcinomas are the major contributor; neuroendocrine cancers are also included.	Colorectal adenocarcinoma	HPV-positive squamous cell carcinoma reported in the rectum
		Signet ring cell carcinoma of colon	Sarcoma or lymphoma reported in colon or rectum
		Large cell neuroendocrine carcinoma in colon	Adenocarcinoma or carcinoid of the small intestines
		Carcinoid in colon	Gastrointestinal stromal tumors
		Adenocarcinoma in the appendix	
		Carcinoid of the appendix	
		Squamous cell carcinoma in colon	
Head or Neck	Carcinomas and other solid tumors	Oropharyngeal	Sarcoma or lymphoma reported in the H&N region

CONFIDENTIALITY
This document is the confidential and proprietary information of GRAIL, LLC. Except as required by federal, state, or local laws or regulations, this document may not be disclosed to others, in whole or in part without prior written permission of GRAIL, Inc.

CSOs	Description	Examples for included cancers	Examples for excluded cancers
	in the head and neck region. This includes HPV-associated squamous cell carcinomas and HPV-negative carcinomas, including neuroendocrine carcinomas and tumors.	HPV-associated squamous cell carcinoma	
		HPV-negative squamous cell carcinoma of the larynx	Skin cancers reported in the H&N region
		Salivary gland adenocarcinoma	
Kidney	Carcinomas and other solid tumors of the renal cortex and renal lobules. These are typically renal cell carcinomas.	Renal cell carcinoma	Sarcoma or lymphoma reported in the kidney
		Carcinoid in kidney	Transitional or urothelial carcinomas reported in the kidney
		Adenocarcinoma NOS in the kidney	
Liver or Intrahepatic bile duct	Carcinomas and other solid tumors of the liver. These are predominantly cancers arising from cells derived from the hepatic progenitor cell.	Hepatocellular carcinoma	Carcinoma of the perihilar bile duct
		Intrahepatic cholangiocarcinoma	Sarcoma or lymphoma reported in the liver
		Small cell carcinoma in the liver	
		Carcinoid in the liver	
Lung	Carcinomas and other solid tumors in the lung. This includes NSCLC, SCLC and low-grade neuroendocrine tumors	Lung adenocarcinoma	Sarcoma or lymphoma reported in the lung
		Lung squamous cell carcinoma	
		NSCLC NOS	
		SCLC	
		Carcinoid in the lung	
Ovary or Fallopian Tubes	Carcinomas and other solid tumors with cell of origin from the ovary or Fallopian tubes. The carcinomas in this region have heterogeneous histologic types, for example serous cystadenocarcinomas, endometrioid carcinomas, Malignant Mullerian mixed tumor (carcinosarcoma) and others.	Fallopian tube-derived serous cystadenocarcinoma of the ovaries	Germ cell tumors
		Endometrioid ovarian carcinoma	Sarcoma or lymphoma reported in the ovaries or peritoneal region
		Malignant mullerian mixed carcinoma of the ovaries	Mesothelioma in the peritoneal region
		Clear cell carcinoma of the ovaries	

CONFIDENTIALITY
This document is the confidential and proprietary information of GRAIL, LLC. Except as required by federal, state, or local laws or regulations, this document may not be disclosed to others, in whole or in part without prior written permission of GRAIL, Inc.

CSOs	Description	Examples for included cancers	Examples for excluded cancers
		Small cell carcinoma of the ovaries	
		Malignant Brenner tumor	
Pancreas, Extrahepatic bile ducts, or Gallbladder	Carcinomas and other solid cancers of pancreas, gallbladder, and extrahepatic bile ducts (perihilar, distal, and cystic ducts). Exocrine and endocrine cancers of the pancreas are included.	Pancreatic Ductal Adenocarcinoma	Sarcoma or lymphoma reported in pancreas, gallbladder, or bile ducts
		Gallbladder adenocarcinoma	Intrahepatic cholangiocarcinoma
		Extrahepatic bile duct carcinoma	
		Cystic duct cholangiocarcinoma	
		Pancreatic neuroendocrine tumor	
Prostate	Cancers with a methylation signal and anatomic site most similar to carcinomas and other solid tumors of the prostate. This includes epithelial carcinomas, and neuroendocrine carcinomas and tumors.	Invasive ductal prostate adenocarcinoma	Sarcoma or lymphoma reported in the prostate
		Invasive acinar adenocarcinoma of the prostate	
		Small cell carcinomas of the prostate	
Skin	Cancers of the skin. These are predominantly melanomas. Other skin cancers include sweat gland carcinomas and Merkel cell carcinomas.	Melanoma of extremities	Basal cell carcinoma of skin (unless metastatic)
		Melanoma in the H&N region	Squamous cell carcinoma of skin (unless metastatic)
		Merkel cell carcinoma	Cutaneous lymphomas
		Digital papillary adenocarcinoma	
Stomach or Esophagus	Carcinomas and other solid tumors of the stomach or esophagus. These are typically epithelial cancers.	Gastric adenocarcinoma	Gastrointestinal stromal tumor (GIST)
		Esophageal adenocarcinoma	MALT lymphoma in stomach
		Esophageal squamous cell carcinoma	Cancers of the small intestines
		Small cell carcinoma in stomach	
		Carcinoid in stomach	

CONFIDENTIALITY
This document is the confidential and proprietary information of GRAIL, LLC. Except as required by federal, state, or local laws or regulations, this document may not be disclosed to others, in whole or in part without prior written permission of GRAIL, Inc.

CSOs	Description	Examples for included cancers	Examples for excluded cancers
Thyroid	Carcinomas and other solid tumors of the thyroid.	Medullary carcinoma of the thyroid	Sarcoma or lymphoma reported in the thyroid
		Papillary carcinoma of the thyroid	
Uterus	Carcinomas and other solid tumors with cell of origin in endometrium or reported at primary anatomic site uterus.	Endometrial carcinoma	Germ cell tumors
		Carcinosarcoma of the uterus	Uterine sarcomas
		High-grade serous cystadenocarcinoma reported with uterus as primary site	Endometrial stromal tumors

CONFIDENTIALITY

This document is the confidential and proprietary information of GRAIL, LLC. Except as required by federal, state, or local laws or regulations, this document may not be disclosed to others, in whole or in part without prior written permission of GRAIL, Inc.

APPENDIX 7 PRESPECIFIED CANCER SUBGROUPS

N	Cancer Type	Cancers Responsible for 2/3 of all Cancer Deaths in the US[1]	Cancers without Common Screening Options	Solid Tumors with Common Screening Options[2]	Solid Tumors without Common Screening Options	Hematologic Malignancies
1	Anus	X	X		X	
2	Bladder or Urothelial tract	X	X		X	
3	Bone or Soft tissue sarcoma		X		X	
4	Breast			X		
5	Cervix			X		
6	Colon or Rectum	X		X		
7	Esophagus	X	X		X	
8	Head and Neck	X	X		X	
9	Kidney		X		X	
10	Liver or Intrahepatic bile duct	X	X		X	
11	Lung	X	X		X	
12	Myeloid Lineage		X			X
13	Lymphoid Lineage	X	X			X
14	Ovary or Fallopian tube	X	X		X	
15	Pancreas, Extrahepatic bile duct or Gallbladder	X	X		X	
16	Plasma cell lineage	X	X			X
17	Prostate			X		
18	Skin (excluding BCC/SCC)		X		X	
19	Stomach	X	X		X	
20	Thyroid		X		X	
21	Uterus		X		X	
22	Other[3]		X		X	

[1] Data source for cancers responsible for ⅔ of all cancer deaths in the US: SEER*Stat Database: Mortality - All Causes of Death, Total US, 2018–2019, released June 2022, underlying data provided by National Center for Health Statistics.

[2] Cancers with common screening options include cancers of the breast, cervix, colon or rectum and prostate. For these cancers, population-wide cancer screening is recommended by several medical societies, and there is an age-based A,B, or C recommendation for screening made by the USPSTF. Lung

CONFIDENTIALITY
This document is the confidential and proprietary information of GRAIL, LLC. Except as required by federal, state, or local laws or regulations, this document may not be disclosed to others, in whole or in part without prior written permission of GRAIL, Inc.

cancer is not included in this definition because screening recommendations apply to a subset of individuals who are at high risk for this cancer due to their smoking history. Source: https://www.uspreventiveservicestaskforce.org/uspstf/

[3] Cancers that are not included in the defined cancer types are reported under the “Other” category. Examples of such cancers are described in Appendix 8.

CONFIDENTIALITY
This document is the confidential and proprietary information of GRAIL, LLC. Except as required by federal, state, or local laws or regulations, this document may not be disclosed to others, in whole or in part without prior written permission of GRAIL, Inc.

Appendix 8 Cancer Type Specifications

Based on AJCC Cancer Staging Manual, 8th edition for solid cancer types and WHO guidance for hematologic malignancies

Cancer Type	Cancer Type Description
Anus	Anus
Bladder or Urothelial Tract	Urinary Bladder<br>Renal Pelvis and Ureter
Bone or Soft tissue sarcoma	Bone<br>Soft Tissue Sarcoma of the Head and Neck<br>Soft Tissue Sarcoma of the Trunk and Extremities<br>Soft Tissue Sarcoma of the Abdomen and Thoracic Visceral Organs<br>Gastrointestinal Stromal Tumors<br>Soft Tissue Sarcoma of the Retroperitoneum<br>Soft Tissue Sarcoma - Unusual Histologies and Sites<br>Corpus Uteri: Leiomyosarcoma, Endometrial Stromal Sarcoma and Adenosarcoma<br>Orbital Sarcoma<br>Subset: Brain and Spinal Cord Sarcomas (CNS Sarcomas [Mesenchymal, Non-meningothelial Tumors])
Breast	Breast
Cervix	Cervix Uteri
Colon or Rectum	Colon Rectum<br>Appendix - Carcinoma<br>Neuroendocrine Tumors of the Appendix<br>Neuroendocrine Tumors of the Colon and Rectum
Esophagus	Esophagus and Esophagogastric Junction
Head and Neck	Mucosal Melanoma of the Head and Neck<br>Cutaneous carcinoma of the Head and Neck<br>Oral Cavity<br>Major Salivary Glands<br>Nasopharynx<br>HPV-Mediated (p16+) Oropharyngeal Cancer<br>Oropharynx (p16-) and Hypopharynx<br>Nasal Cavity and Paranasal Sinuses<br>Larynx
Kidney	Kidney
Liver or Intrahepatic bile duct	Liver<br>Intrahepatic bile ducts
Lung	Lung

CONFIDENTIALITY

This document is the confidential and proprietary information of GRAIL, LLC. Except as required by federal, state, or local laws or regulations, this document may not be disclosed to others, in whole or in part without prior written permission of GRAIL, Inc.

Cancer Type	Cancer Type Description
Myeloid Lineage	Myeloproliferative Neoplasms (Chronic myeloid leukemia,<br>Polycythemia vera,<br>Essential thrombocythemia,<br>Primary myelofibrosis,<br>Chronic neutrophilic leukemia,<br>Chronic eosinophilic leukemia,<br>Juvenile myelomonocytic leukemia,<br>Myeloproliferative neoplasm, NOS)<br>Mastocytosis (Cutaneous mastocytosis, Systemic mastocytosis, Mast cell sarcoma)<br>Myelodysplastic/Myeloproliferative Disorders (Myelodysplastic neoplasms (with defining genetic abnormalities or morphologically defined),<br>Childhood Myelodysplastic Neoplasms,<br>Chronic myelomonocytic leukemia,<br>Myelodysplastic/myeloproliferative neoplasm with neutrophilia,<br>Myelodysplastic/myeloproliferative neoplasm with SF3B1 mutation and thrombocytosis, myelodysplastic/myeloproliferative neoplasm NOS)<br>Acute Myeloid Leukemia (AML) and related neoplasms<br>Dendritic cell and histiocytic neoplasms (Plasmacytoid dendritic cell neoplasms, Langerhans cell and other dendritic neoplasms, Histiocytic neoplasms)
Lymphoid Lineage	B Cell Lymphoid Proliferations and Lymphomas (B-cell predominance,<br>B-cell lymphoblastic leukaemias/lymphomas,<br>Mature B cell,<br>Small Lymphocytic Proliferations,<br>Splenic B-cell lymphomas and leukaemias,<br>Lymphoplasmacytic lymphoma,<br>Marginal zone lymphoma,<br>Follicular Lymphoma,<br>Cutaneous follicle center lymphoma,<br>Mantle cell lymphoma,<br>Transformations of indolent B-cell lymphomas<br>Large B-cell lymphomas,<br>Burkitt lymphoma,<br>Hodgkin Lymphoma,<br>KSHV/HHV8-associated B-cell lymphoid proliferations and lymphomas,<br>Lymphoid proliferations and lymphomas associated with immune deficiency and dysregulation)<br>T Cell and NK Lymphoid Proliferations and Lymphomas (T-cell predominance,<br>T-lymphoblastic leukemia/lymphoma,<br>Mature T-cell and NK-cell neoplasms,<br>Primary cutaneous T-cell lymphomas)<br>Stroma Derived Neoplasms of Lymphoid Tissues (Mesenchymal dendritic cell neoplasms,<br>Myofibroblastic tumor,

CONFIDENTIALITY

This document is the confidential and proprietary information of GRAIL, LLC. Except as required by federal, state, or local laws or regulations, this document may not be disclosed to others, in whole or in part without prior written permission of GRAIL, Inc.

Cancer Type	Cancer Type Description
	Spleen-specific vascular-stromal tumors)
Ovary or Fallopian tubes	Ovary, Fallopian Tube, and Primary Peritoneal Carcinoma
Pancreas, Extrahepatic bile duct or Gallbladder	Gallbladder<br>Perihilar Bile Ducts<br>Distal Bile Duct<br>Exocrine Pancreas<br>Neuroendocrine Tumors of the Pancreas
Plasma Cell Lineage	Plasma Cell Myeloma<br>Solitary plasmacytoma of bone<br>Extraosseous plasmacytoma
Prostate	Prostate
Skin (excluding BCC/SCC)	Merkel Cell Carcinoma<br>Melanoma of the Skin
Stomach	Stomach<br>Neuroendocrine Tumors of the Stomach
Thyroid	Thyroid - Differentiated and Anaplastic Carcinoma<br>Thyroid - Medullary
Uterus	Corpus Uteri - Carcinoma and Carcinosarcoma
Other	Examples include:<br>Brain and Spinal Cord tumors (other than CNS Lymphomas, CNS Sarcomas Mesenchymal, Non-meningothelial Tumors)<br>Unknown Primary Tumors<br>Conjunctival Melanoma<br>Uveal Melanoma<br>Parathyroid<br>Ampulla of Vater<br>Neuroendocrine Tumors of the Duodenum and Ampulla of Vater<br>Small Intestine<br>Neuroendocrine Tumors of the Jejunum and Ileum<br>Thymus<br>Malignant Pleural Mesothelioma<br>Vulva<br>Vagina<br>Gestational Trophoblastic Neoplasms<br>Penis<br>Testis<br>Eyelid Carcinoma<br>Conjunctival Carcinoma<br>Retinoblastoma

CONFIDENTIALITY
This document is the confidential and proprietary information of GRAIL, LLC. Except as required by federal, state, or local laws or regulations, this document may not be disclosed to others, in whole or in part without prior written permission of GRAIL, Inc.

Cancer Type	Cancer Type Description
	Lacrimal Gland Carcinoma<br>Adrenal Cortical Carcinoma<br>Adrenal - Neuroendocrine Tumors<br>Prostatic Urethra (Urothelial Carcinomas, Squamous Cell Carcinoma)

CONFIDENTIALITY
This document is the confidential and proprietary information of GRAIL, LLC. Except as required by federal, state, or local laws or regulations, this document may not be disclosed to others, in whole or in part without prior written permission of GRAIL, Inc.

Appendix 9 Sample Size and Power for Bridging Analysis, Stratification Factors, Approximate Sampling and Analysis Weights

The objective of bridging sampling for MCED-I re-testing among true negatives (TN) for MCED-V2 is to assess the false positive (FP) rate (or specificity) for MCED-I, and to ensure that the lower bound of the 95% confidence interval for the estimated MCED-I specificity after sampling will exceed the specificity target of 99% with 90% power.

Other than the 99% specificity threshold, two factors affect power and sample size planning for bridging sampling: the true specificity in the PATHFINDER 2 study population and the degree of overlap for FP results between MCED-V2 and MCED-I. The more sharing of FPss between MCED-V2 and MCED-I, the more MCED-I FPs will be contained in MCED-V2 FPs, and so fewer samples will be needed from the TN for MCED-V2 group to assess MCED-I FPs.

The test design features and the preliminary data from GRAIL suggest that the true specificity of MCED-I in PATHFINDER 2 will be most likely in the range of 99.3% to 99.5%, and the degree of FP sharing is about ½, which means that about half of MCED-V2 FPs will also be FPs for MCED-I. To be conservative in sample size planning, we will assume that about 1/3 of FPs are shared between MCED-V2 and MCED-I.

By selecting approximately 7,500 samples from the PATHFINDER 2 MCED-V2 TN group for MCED-I testing, we will have >90% power to pass the acceptance criterion target of 99% specificity under the most conservative scenario for true specificity and the amount of FP sharing between MCED-V2 and MCED-I.

As discussed in Section 4.2, the subset selection of participants with negative MCED-V2 test results and no cancer diagnosis during 12 months of follow-up time (i.e., MCED-V2 TN) will be stratified based on the following factors:

1. Age (50-59, 60-69, 70-79, 80+ years)
2. Race (American Indian or Alaska Native; Asian; Black or African American; Native Hawaiian or Other Pacific Islander; White; More than one race, Not reported)
3. Ethnicity (Hispanic or Latino, Not Hispanic or Latino, Missing)
4. Prior cancer history (yes, no)
5. Smoking status (Current smoker, Former Smoker, Non-Smoker)
6. Selected conditions that may affect cfDNA shedding or methylation (as described in Section 14.3.3.2) and which represent relatively small subsets of the PATHFINDER 2 population:
    - 6.1. Those who took any cytotoxic medications in the past year
    - 6.2. Those who took any demethylating agents in the past year
    - 6.3. Participants with solid organ or stem cell (bone marrow) transplant
    - 6.4. HIV Infection
    - 6.5. Pancreatitis
    - 6.6. Chronic lung disease

CONFIDENTIALITY
This document is the confidential and proprietary information of GRAIL, LLC. Except as required by federal, state, or local laws or regulations, this document may not be disclosed to others, in whole or in part without prior written permission of GRAIL, Inc.

The proposed approximate sampling weights for each stratum, defined by the stratification factors, are presented in the table below. The aim of this sampling approach is to ensure sufficient representation of subsets of interest (especially relatively small subsets of the population) important to characterize diversity and representativeness and MCED test performance in terms of FP rate. The final sampling and analysis weights will be defined based on the locked analysis dataset.

Note, that enumeration of all possible combinations of the 11 stratification factors listed above would lead to an unfeasibly large number of strata (>16,000). Therefore, the following approach was applied:

1. Small important subsets were selected sequentially including participants with
   - Cytotoxic medication use
   - Demethylating agent use
   - Transplant
   - HIV Infection
   - Pancreatitis
   - Chronic lung disease
   - Current smoker
   - Those who checked "Native Hawaiian or Pacific Islander"
   - Those who checked "American Indian or Alaska Native"
   - More than one race
2. Cross-tabulation of the remaining factors was examined to identify relatively large subsets which could be downsampled (such as White, Non Hispanic or Latino, No prior cancer history) for each age category.
3. The remaining smaller subsets for each age category were pooled together and upsampled compared to step #2 above.
4. After steps 1, 2 and 3 were implemented, the distribution of all stratification factors was examined to assure sufficient representation.

Stratification Factors, Approximate Sampling and Analysis Weights

Note: strata are defined sequentially and should be implemented in the order in which they appear in the table.

N	Stratum	N (estimated)	Sampling probability	N Sampled (approximate)	Analysis Weight (approximate)
1	Demethylating agents = Yes	17	100%	17	1.0
2	Cytotoxic medications = Yes	375	50%	188	2.0
3	Transplant = Yes	48	100%	48	1.0
4	HIV = Yes	78	100%	78	1.0

CONFIDENTIALITY
This document is the confidential and proprietary information of GRAIL, LLC. Except as required by federal, state, or local laws or regulations, this document may not be disclosed to others, in whole or in part without prior written permission of GRAIL, Inc.

N	Stratum	N (estimated)	Sampling probability	N Sampled (approximate)	Analysis Weight (approximate)
5	Pancreatitis = Yes	234	100%	234	1.0
6	Chronic lung disease = Yes	567	35%	198	2.9
7	Current smoker = Yes	784	60%	470	1.7
8	Native Hawaiian or Other Pacific Islander	89	100%	89	1.0
9	American Indian or Alaska Native	295	100%	295	1.0
10	More than one race	122	100%	122	1.0
11	Age >=80, White, Not Hispanic or Latino, No prior hx	550	75%	412	1.3
12	Age >=80 all others	248	100%	248	1.0
13	Age 70-79, White, Not Hispanic or Latino, No prior hx	4134	15%	620	6.7
14	Age 70-79, White, Not Hispanic or Latino, Prior hx	755	40%	302	2.5
15	Age 70-79, Black, Not Hispanic or Latino, No prior hx	323	40%	129	2.5
16	Age 70-79, Asian, Not Hispanic or Latino, No prior hx	175	40%	70	2.5
17	Age 70-79, White, Hispanic or Latino, No prior hx	161	40%	64	2.5
18	Age 70-79 all others	240	100%	240	1.0
19	Age 60-69, No prior hx, White, Not Hispanic or Latino,	7567	10%	757	10.0
20	Age 60-69, White, Not Hispanic or Latino, Prior hx	762	35%	267	2.9
21	Age 60-69, Black, Not Hispanic or Latino, No prior hx	671	35%	235	2.9
22	Age 60-69, Asian, Not Hispanic or Latino, No prior hx	416	35%	146	2.8
23	Age 60-69, White, Hispanic or Latino, No prior hx	392	35%	137	2.9
24	Age 60-69 all others	447	100%	447	1.0
25	Age 50-59, White, Hispanic or Latino, No prior hx	422	35%	148	2.9
26	Age 50-59, White, Not Hispanic or Latino, No prior hx	3787	15%	568	6.7
27	Age 50-59, White, Not Hispanic or Latino, Prior hx	286	50%	143	2.0
28	Age 50-59, Black, Not Hispanic or Latino, No prior hx	592	35%	207	2.9
29	Age 50-59, Asian, Not Hispanic or Latino, No prior hx	667	35%	233	2.9
30	Age 50-59 all others	475	100%	475	1.0

CONFIDENTIALITY
This document is the confidential and proprietary information of GRAIL, LLC. Except as required by federal, state, or local laws or regulations, this document may not be disclosed to others, in whole or in part without prior written permission of GRAIL, Inc.

APPENDIX 10 EXAMPLES OF INVASIVE PROCEDURES

The National Cancer Institute definition states that invasive procedures are medical procedures which invade (enter) the body, usually by cutting or puncturing the skin or by inserting instruments into the body.[30]

Invasive procedures include surgical and non-surgical procedures. Surgical procedures are associated with the greatest risk of developing complications and involve a structural alteration of the human body and/or incision or destruction of tissues using instruments.[31]

Type of Diagnostic Testing	Category	Specific procedure
Surgical Procedure	All	Laparoscopy
		Thoracoscopy
		Mediastinoscopy
		Thoracotomy
		Laparotomy
Non-Surgical Procedure	Endoscopy	Anoscopy
		Colonoscopy
		Sigmoidoscopy
		Esophagogastroduodenoscopy (EGD)
		Endoscopic Ultrasound
		Endoscopic retrograde cholangiopancreatography (ERCP)
		Bronchoscopy
		Cystoscopy
	Biopsy	Fine needle aspiration biopsy
		Core needle biopsy
		Bone marrow biopsy
		Incisional biopsy
		Excisional biopsy (of skin)
	Other	Pap smear
		Paracentesis
		Thoracocentesis

30 https://www.cancer.gov/search/results?swKeyword=invasive+procedure
31 This language is consistent with the SNOMED definition of surgical procedure.

CONFIDENTIALITY
This document is the confidential and proprietary information of GRAIL, LLC. Except as required by federal, state, or local laws or regulations, this document may not be disclosed to others, in whole or in part without prior written permission of GRAIL, Inc.

APPENDIX 11 RECOMMENDED DIAGNOSTIC EVALUATIONS BY CSO PREDICTION

MCED-I CSO prediction	MCED-V2 CSO prediction	V2-based Predicted Cancer(s)	Expected Diagnostic Evaluation	Type of Diagnostic Evaluation
Anus	Anus	Anus	Anoscopy or sigmoidoscopy	Endoscopy
Bladder or Urothelial tract	Bladder, Urothelial tract	Bladder, Renal Pelvis, Ureter, Urethra	Flexible cystoscopy and CT urogram or retrograde pyelogram with contrast	Endoscopy<br><br>Imaging
Bone or Soft Tissue	Bone and Soft Tissue	Skeletal Muscle and Other Connective Tissue, Vascular Tissue, Bone and Cartilage	MRI with contrast or CT of chest, abdomen, pelvis or PET-CT	Imaging
Blood, lymphatic system, or bone marrow	Lymphoid Lineage	Lymphoid leukemia<br><br>Lymphoma	CBC with differential, peripheral blood smear, LDH, Hepatitis B and C serology, comprehensive metabolic panel (CMP)<br><br>Bone marrow biopsy after blood tests if needed<br><br>CT of chest, abdomen, pelvis with contrast or PET-CT if needed	Lab test<br><br>Biopsy<br><br>Imaging
	Myeloid Lineage	Myeloid neoplasm	CBC with differential, peripheral blood smear, LDH, uric acid, CMP<br><br>Bone marrow biopsy after blood tests if needed	Lab test<br><br>Biopsy
	Plasma Cell Lineage	Plasma cell neoplasm	CBC with differential, peripheral blood smear, LDH, uric acid, beta-2-microglobulin, creatinine clearance, serum immunoglobulins, serum free light chain assay, serum and urine protein electrophoresis, CMP<br><br>Bone marrow biopsy after blood and urine tests if needed<br><br>Whole-body low-dose CT or PET-CT if needed	Lab test<br><br>Biopsy<br><br>Imaging

CONFIDENTIALITY
This document is the confidential and proprietary information of GRAIL, LLC. Except as required by federal, state, or local laws or regulations, this document may not be disclosed to others, in whole or in part without prior written permission of GRAIL, Inc.

MCED-I CSO prediction	MCED-V2 CSO prediction	V2-based Predicted Cancer(s)	Expected Diagnostic Evaluation	Type of Diagnostic Evaluation
Breast	Breast	Breast	Diagnostic mammography<br><br>MRI if participant has recently had screening mammogram	Imaging
Cervix	Cervix	Cervix	Cytology (i.e., Pap smear), HPV testing, colposcopy	Other non-surgical procedure<br><br>Endoscopy
Colon or Rectum	Colon, Rectum	Colon, Rectum, Appendix	Colonoscopy	Endoscopy
Head and Neck	Head and Neck	Oropharynx, Hypopharynx, Nasopharynx, Larynx, Lip and Oral Cavity (including Oral tongue), Nasal Cavity, Paranasal Sinuses, Major Salivary glands	Flexible nasal endoscopy and Head and Neck CT or MRI	Endoscopy<br><br>Imaging
Kidney	Kidney	Kidney	Triple phase renal CT	Imaging
Liver or Intrahepatic bile duct	Liver, Bile duct	Liver, Intrahepatic bile duct	Triple phase abdominal CT, AFP	Imaging<br><br>Lab test
Lung	Lung	Lung, Bronchus	Chest CT<br><br>PET-CT	Imaging
Ovary or Fallopian tube	Ovary	Ovary, Fallopian tube, Primary Peritoneum	Transvaginal ultrasound and CA-125	Imaging<br><br>Lab test
Pancreas, Extrahepatic bile ducts or Gallbladder	Pancreas, Gallbladder	Pancreas, Extrahepatic bile duct, Gallbladder	Pancreas CT protocol or MRCP	Imaging
Prostate	Prostate	Prostate	PSA and multi-parametric MRI of the prostate	Imaging<br><br>Lab test

CONFIDENTIALITY
This document is the confidential and proprietary information of GRAIL, LLC. Except as required by federal, state, or local laws or regulations, this document may not be disclosed to others, in whole or in part without prior written permission of GRAIL, Inc.

MCED-I CSO prediction	MCED-V2 CSO prediction	V2-based Predicted Cancer(s)	Expected Diagnostic Evaluation	Type of Diagnostic Evaluation
Skin	Melanocytic Lineage	Melanoma	Full body skin exam	Non-invasive procedure
Stomach or Esophagus	Stomach, Esophagus (upper GI)	Stomach, Esophagus	Upper endoscopy	Endoscopy
Thyroid	Thyroid Gland	Thyroid Gland	Ultrasound of thyroid	Imaging
Uterus	Uterus	Uterus	Transvaginal ultrasound	Imaging

CONFIDENTIALITY
This document is the confidential and proprietary information of GRAIL, LLC. Except as required by federal, state, or local laws or regulations, this document may not be disclosed to others, in whole or in part without prior written permission of GRAIL, Inc.

Appendix 12 USPSTF cancer screening recommendations

Cancer	Grade	Eligible population	Cancer screening recommendations
Breast[32]	B	Women 40-74 years	Mammography or digital breast tomosynthesis every 2 years
Cervix[33]	A	Women 21-65 years	● Pap smear (cervical cytology) every 3 years<br>● Pap smear with high-risk HPV testing every 5 years<br>● High-risk HPV testing alone every 5 years
Colorectal[34]	A	Adults 50-75 years	● High-sensitivity gFOBT or FIT every year<br>● sDNA-FIT every 1-3 years<br>● CT colonography every 5 years<br>● Flexible sigmoidoscopy every 5 years<br>● Flexible sigmoidoscopy every 10 years plus FIT every year<br>● Colonoscopy every 10 years
	B	Adults 45-49 years	
	C	Adults 76-85 years	Selective screening offered on an individual basis based on health status
Lung[35]	B	Adults 50-80 years with ≥20 pack-year smoking history who currently smoke or have quit within the past 15 years	Annual low-dose computed tomography (LDCT)
Prostate[36]	C	Men 55-69 years	Individual decision for periodic PSA-based screening after discussion of benefits and harms with clinician

Note: Expanded breast cancer screening includes MRI[37] or ultrasound in addition to tests described in the table, and will be included as a separate category in the analysis

[32] https://www.uspreventiveservicestaskforce.org/uspstf/recommendation/breast-cancer-screening
[33] https://www.uspreventiveservicestaskforce.org/uspstf/recommendation/cervical-cancer-screening
[34] https://www.uspreventiveservicestaskforce.org/uspstf/recommendation/colorectal-cancer-screening
[35] https://www.uspreventiveservicestaskforce.org/uspstf/recommendation/lung-cancer-screening
[36] https://www.uspreventiveservicestaskforce.org/uspstf/recommendation/prostate-cancer-screening
[37] Monticciolo D.L. et al, Breast Cancer Screening for Women at Higher-Than-Average Risk: Updated Recommendations From the ACR, Clinical Practice Management, 2023, Volume 20, Issue 9, p902-914.

CONFIDENTIALITY
This document is the confidential and proprietary information of GRAIL, LLC. Except as required by federal, state, or local laws or regulations, this document may not be disclosed to others, in whole or in part without prior written permission of GRAIL, Inc.

Appendix 13 Description of Participant-Directed Questionnaires.

State-Trait Anxiety Inventory (STAI)

The STAI is one of the most widely used measures of anxiety. The instrument is scored on a scale of 20-80, with higher scores indicating greater anxiety The abbreviated version contains six items and four choices (1 = not at all, 2 = somewhat, 3 = moderately, and 4 = very much so) that reflect common symptoms of anxiety experienced by an individual. The scores on the three positively worded items were reverse-coded, a higher score corresponded to a higher level of individual anxiety. STAI scores are commonly classified as “no or low anxiety” (20−34), “moderate anxiety” (35−44), and “high anxiety” (45−80).

STAI-6 is a simplified version of STAI-20, and the total summed scores were prorated (multiplied by 20/6) to obtain scores that were comparable to those from the full 20-item STAI (Knight et al., 1983; Marteau and Bekker, 1992).

Figure. STAI-6

A number of statements which people have used to describe themselves are given on the following page. Read each statement and select the appropriate response to indicate how you feel right now, that is, at this very moment. There are no right or wrong answers. Do not spend too much time on any one statement but give the answer which seems to describe your present feelings best.				
Statement	Not at all	Somewhat	Moderately	Very much
I feel calm	1	2	3	4
I feel tense	1	2	3	4
I feel upset	1	2	3	4
I feel relaxed	1	2	3	4
I feel content	1	2	3	4
I am worried	1	2	3	4

MCED STAI is a questionnaire with 3 items modeled after the corresponding items for STAI-6 with the same four choices (1 = not at all, 2 = somewhat, 3 = moderately, and 4 = very much so):

1. I am tense because of my multi-cancer early detection test
2. I feel upset because of my multi-cancer early detection test
3. I am worried because of my multi-cancer early detection test

CONFIDENTIALITY
This document is the confidential and proprietary information of GRAIL, LLC. Except as required by federal, state, or local laws or regulations, this document may not be disclosed to others, in whole or in part without prior written permission of GRAIL, Inc.

Adapted MICRA

The Multidimensional Impact of Cancer Risk Assessment (MICRA) is a previously validated instrument that appropriately assessed different levels of impact of genetic test result disclosure. The MICRA was adapted with permission to measure impacts of MCED result disclosure and is composed of 21 items addressing 3 domains (distress, uncertainty, positive experience) and a total score; four conditional items from the original MICRA relating to parenthood and prior cancer were excluded. Higher scores indicate increased distress, uncertainty, and less positive experience.

Overview of scales and items of the adapted MICRA

Adapted MICRA Scale	Number of items	Scale range	Item numbers
Distress	6	0-30	1-4, 7-8
Uncertainty	9	0-45	9-12, 14-17, 20
Positive experience	4	0-20	5-6, 18-19
Total score	19	0-95	1-12, 14-20

The MICRA questionnaire consists of three subscales, a Positive Experience subscale (4 items), a Distress subscale (6 items), and an Uncertainty subscale (9 items). The questionnaire asks the respondents to indicate whether they have experienced each statement on a four-point scale (0, 1, 3, 5) in the past week. A response of 0 indicated never experiencing the statement while a 5 indicated often experiencing the statement. A higher score on any of the subscales or the total scale indicated greater psychological distress. The positive subscale is reverse scored to reflect this. The scoring of the adapted MICRA is based on the FACIT group's MICRA scoring manual.

SF-12v2

The Short Form-12 version 2 Health Survey (SF-12v2) is a 12-item, multipurpose, short-form health survey that was developed to be a brief, broad measure of eight domains of health status. The SF-12v2® Health Survey yields an eight-scale profile of functional health and wellbeing, as well as two psychometrically based physical and mental health summary measures and a preference-based health utility index.

The SF-12v2 is a generic measure of health and does not target a specific age, disease, or treatment group and has been in surveys of general and specific populations, in comparing the relative burden of diseases, and in differentiating the health benefits produced by a wide range of treatments. However, it is not intended to be a comprehensive survey of health.

The SF-12v2 includes eight health domains: physical functioning, role participation with physical health problems (role physical), bodily pain, general health, vitality, social functioning (i.e., limitations in social functioning), role participation with emotional health problems (role-emotional), and mental health. The component summary measures reflect two broad components of health: physical and mental. All eight health domain scales are used to score both component summary

CONFIDENTIALITY
This document is the confidential and proprietary information of GRAIL, LLC. Except as required by federal, state, or local laws or regulations, this document may not be disclosed to others, in whole or in part without prior written permission of GRAIL, Inc.

measures. Higher scores indicate better health.

The scoring of the SF-12v2 is based on QualityMetric Incorporated SF12v2 Administration guide (Ware, J. E., Jr., Kosinski, M., Turner-Bowker, D. M., Sundaram, M., Gandek, B., & Maruish, M. E. (2009). SF-12v2 Health Survey: Administration guide for clinical trial investigators. Lincoln, RI: QualityMetric Incorporated). Higher scores represent better health.

Satisfaction with multi-cancer early-detection test

To assess satisfaction with the MCED test, a 3-item questionnaire was developed by the study investigators. Each question included response options reflecting 5- or 7-point Likert scales, with total satisfaction scores ranging from 0 to 100 with higher scores representing higher satisfaction.

The following questions were included in the satisfaction questionnaire:

1. Overall, how confident are you that this multi-cancer early detection test is a good thing for you?
2. How certain are you that the good things about the multi-cancer early detection test outweigh the bad things?
3. Taking all things into account, how satisfied or dissatisfied are you with the multi-cancer early detection test?

CONFIDENTIALITY
This document is the confidential and proprietary information of GRAIL, LLC. Except as required by federal, state, or local laws or regulations, this document may not be disclosed to others, in whole or in part without prior written permission of GRAIL, Inc.

Appendix 14 Estimated effective radiation dose estimates for imaging tests.

Imaging test	Estimated effective dose (milliSieverts)
Barium enema	8.0
LDCT: lung cancer screening	1.2
LDCT: whole body	4.8
CT Abdomen	8.0
CT Abdomen and pelvis	10.5
CT Abdomen and pelvis with and without contrast	17.4
CT Abdomen and pelvis - multiphase	22.6
CT Brain	1.8
CT Chest	6.6
CT Chest with contrast	17.0
CT Head	2.7
CT Head and neck	8.0
CT Liver (triple phase)	15.0
CT Neck	6.8
CT Pancreas protocol	23.7
CT Pelvis	6.0
CT Spine	19.9
CT Cervical spine	2.7
CT Thoracic spine	13.1
CT Lumbar spine	11.2
CT Triple phase renal	29.4
CT Urogram	6.1
CT Whole body	15.4
Dual x-ray absorptiometry (with CT)	0.04
Dual x-ray absorptiometry (without CT)	0.001
Endoscopic retrograde cholangiopancreatography	4.0
Intravenous urography	3.0

CONFIDENTIALITY
This document is the confidential and proprietary information of GRAIL, LLC. Except as required by federal, state, or local laws or regulations, this document may not be disclosed to others, in whole or in part without prior written permission of GRAIL, Inc.

Imaging test	Estimated effective dose (milliSieverts)
Mammography	0.4
Octreotide scan	12.0
PET (18F-FDG)	14.1
Retrograde pyelogram	7.7
Screening Digital Breast Tomosynthesis	0.3
Screening Digital Mammography	0.2
Small-bowel series	5.0
Upper gastrointestinal series	6.0
X-ray Abdomen	0.7
X-ray Cervical spine	0.2
X-ray Chest	0.1
X-ray Hip	0.7
X-ray Knee	0.005
X-ray Lumbar spine	1.5
X-ray Other extremities	0.001
X-ray Pelvis	0.6
X-ray Posteroanterior and lateral study of chest	0.1
X-ray Posteroanterior study of chest	0.02
X-ray Shoulder	0.01
X-ray Skull	0.1
X-ray Thoracic spine	1.0

Effective radiation dose estimates were obtained from several sources. If more than one dose estimate was available, the estimates were chosen in order from the sources listed below:

1. University of California, San Francisco International CT Dose Registry. https://radiology.ucsf.edu/blog/uc-dose-project-and-recent-research-call-standard-protocols-ct-scan-doses. Estimates provided to GRAIL on 26 April 2024.
2. Mettler FA Jr, Huda W, Yoshizumi TT, Mahesh M. Effective doses in radiology and diagnostic nuclear medicine: a catalog. Radiology. 2008 Jul;248(1):254-63. doi: 10.1148/radiol.2481071451. PMID: 18566177.
3. Smith-Bindman R, Lipson J, Marcus R, et al. Radiation Dose Associated With Common Computed Tomography Examinations and the Associated Lifetime Attributable Risk of

CONFIDENTIALITY

This document is the confidential and proprietary information of GRAIL, LLC. Except as required by federal, state, or local laws or regulations, this document may not be disclosed to others, in whole or in part without prior written permission of GRAIL, Inc.

Cancer. *Arch Intern Med.* 2009;169(22):2078–2086. doi:10.1001/archinternmed.2009.427
4. Hemke R, Yang K, Husseini J, Bredella MA, Simeone FJ. Organ dose and total effective dose of whole-body CT in multiple myeloma patients. Skeletal Radiol. 2020 Apr;49(4):549-554. doi: 10.1007/s00256-019-03292-z. Epub 2019 Oct 15. PMID: 31612246; PMCID: PMC7021660.
5. Lipkin ME, Mancini JG, Zilberman DE et al. Reduced radiation exposure with the use of an air retrograde pyelogram during fluoroscopic access for percutaneous nephrolithotomy. J Endourol. 2011 Apr;25(4):563-7. doi: 10.1089/end.2010.0431. Epub 2011 Mar 22. PMID: 21426236.
6. van der Molen AJ, Miclea RL, Geleijns J, Joemai RMS. A Survey of Radiation Doses in CT Urography Before and After Implementation of Iterative Reconstruction. American Journal of Roentgenology 2015 205:3, 572-577
7. Quinn, B, Dauer, Z, Pandit-Taskar, N *et al.* Radiation dosimetry of 18F-FDG PET/CT: incorporating exam-specific parameters in dose estimates. BMC Med Imaging 16, 41 (2016). https://doi.org/10.1186/s12880-016-0143-y

CONFIDENTIALITY
This document is the confidential and proprietary information of GRAIL, LLC. Except as required by federal, state, or local laws or regulations, this document may not be disclosed to others, in whole or in part without prior written permission of GRAIL, Inc.

STATISTICAL ANALYSIS PLAN (SAP) for PREMARKET APPROVAL (PMA) SUBMISSION

GRAIL-012 The PATHFINDER-2 Study

GRAIL Multi-Cancer Test

**Study Title:**	The PATHFINDER 2 Study: Evaluating the Safety and Performance of the GRAIL Multi-Cancer Early Detection Test in an Eligible Screening Population
**Study Number:**	GRAIL-012, NCT05155605
**Sponsor:**	GRAIL, LLC<br>1525 O'Brien Dr<br>Menlo Park, CA 94025
**SAP Version:**	2.0
**SAP Date:**	Feb 7, 2025

CONFIDENTIALITY
This document is the confidential and proprietary information of GRAIL, LLC. Except as required by federal, state, or local laws or regulations, this document may not be disclosed to others, in whole or in part without prior written permission of GRAIL, Inc.

DOCUMENT HISTORY

Version	Date	Replaces	Description of Change
1.0	May 13, 2024	N/A	Initial Release
2.0	Feb 7, 2025	1.0	1. Added the description and analysis methods for MCED-I cancer biology signal prediction, in section 6.3, 8.1, 8.2 and 14.2.2.1.<br>2. Updated and clarified the definition of cancer types, CSO and CBS in Appendices 3B, 6, 8.<br>3. Updated the acceptance criteria to study success criteria, with a simplified hierarchical testing. Clarified the study success criteria based on the lower bound of the two-sided 95% confidence interval, in section 3.1, 14.2.2.2, 17.<br>4. Updated safety and diagnostic evaluation analyses to focus on MCED-V2 in sections 14.2.1 and 14.3.2.<br>5. Removed exploratory objective related to evaluation of DNA based and other biomarkers in tissue because tissue collection was discontinued (sections 3.3, 11, 14.4).

CONFIDENTIALITY
This document is the confidential and proprietary information of GRAIL, LLC. Except as required by federal, state, or local laws or regulations, this document may not be disclosed to others, in whole or in part without prior written permission of GRAIL, Inc.

The signature below constitutes the approval of this SAP.

[redacted]

08-Feb-2025

Date

Distinguished Scientist, Medical Affairs

[redacted]

10-Feb-2025

Date

Vice President, Measurement Data Science

[redacted]

07-Feb-2025

Date

Sr. Director, Biostatistics & Translational BioInformatics

CONFIDENTIALITY
This document is the confidential and proprietary information of GRAIL, LLC. Except as required by federal, state, or local laws or regulations, this document may not be disclosed to others, in whole or in part without prior written permission of GRAIL, Inc.

Co-authors of the SAP:

[redacted]

07-Feb-2025

Date

Sr Director, Biostatistics

[redacted]

07-Feb-2025

Date

Vice President, Clinical Science

CONFIDENTIALITY

This document is the confidential and proprietary information of GRAIL, LLC. Except as required by federal, state, or local laws or regulations, this document may not be disclosed to others, in whole or in part without prior written permission of GRAIL, Inc.

Contents

1. Definitions and Abbreviations 9
2. Background and Rationale 11
3. Study Objectives 11
3.1 Primary Objectives 11
3.2 Secondary Objectives 12
3.3 Exploratory Objectives 13
4. Study Design 14
4.1 PATHFINDER 2 Study Design 14
4.2 Bridging Analysis for MCED-I Test 15
5. Study Population 16
6. Data 16
6.1 Data Sources 16
6.2 Clinical Data 16
6.3 MCED Test Data 17
6.4 Data Lock Process and Merge 18
7. Measures to Minimize Bias 19
8. Outcome Definitions 19
8.1 GRAIL MCED Test Result 19
8.2 Target Clinical Outcome 20
8.2.1 Cancer 20
8.2.2 Cancer Clinical Stage 20
8.2.3 Cancer Type 21
8.2.4 Clinical CSO 21
8.2.5 Clinical CBS 21
8.2.6 Cancer of Unknown Primary Site 22
8.2.7 Multiple Cancers 22
8.2.8 Definition of Cancer Participant for Specific Analysis 22
8.2.9 Definition of Non-cancer Participant for Specific Analysis 23
9. Handling of Dropouts or Missing Data 23
10. Analysis Sets 23
10.1 Consented Analysis Set 24
10.2 Clinically Eligible and Clinically Evaluable Analysis Set 24
10.3 Analyzable Sets 24
10.4 Cancer Signal Detected Analysis Sets 24

CONFIDENTIALITY

This document is the confidential and proprietary information of GRAIL, LLC. Except as required by federal, state, or local laws or regulations, this document may not be disclosed to others, in whole or in part without prior written permission of GRAIL, Inc.

10.5 No Cancer Signal Detected Analysis Sets 24
11. Study Analyses 25
11.1 PMA Analysis 26
11.2 First Post PMA Analysis 26
11.3 Additional Post PMA Analyses 27
11.4 IDMC Analyses 27
12. Multiplicity and Multiple Hypothesis Testing 27
13. Study Cohort Characteristics and Participant Disposition 27
13.1 General Considerations 27
13.2 Enrollment 28
13.3 Demographics and Baseline Characteristics 28
13.4 Participant Disposition 29
14. Statistical Analysis Methods 30
14.1 General Considerations 30
14.1.1 Study Objectives and Analysis Sets 30
14.1.2 Subgroup Considerations and Confidence Intervals 31
14.1.3 Cancer Status Assessments and Loss to Follow-Up 32
14.1.4 Weighted Analyses for MCED-I 33
14.1.5 Analysis Time Window for PRO Questionnaires 34
14.2 Analysis Methods for Primary Objectives 34
14.2.1. Primary Objective One (Safety) 34
14.2.1.1 Safety Endpoints 34
14.2.1.2 Analysis Methods for Safety Analyses of MCED-V2 Test 35
14.2.2. Primary Objective Two (Test Performance) 38
14.2.2.1 Test Performance Endpoints 38
14.2.2.1.1 Test Performance Endpoints for Cancer Signal Detection 38
14.2.2.1.2 Test Performance Endpoints for CSO Prediction 41
14.2.2.1.3 Test Performance Endpoints for MCED-I CSO or CBS Prediction 42
14.2.2.1.4 Test Performance Endpoints for Combined Cancer Signal Detection and CSO Prediction or CBS Prediction 43
14.2.2.1.5 Analyses by Stage 44
14.2.2.1.6 Concordance of MCED-V2 Test and MCED-I Test Results 45
14.2.2.1.7 Descriptive Summaries 45
14.2.2.2 Study Success Criteria for PMA Analysis 47
14.2.2.3 Evaluation of MCED-V2 Test Performance 48
14.2.2.3.1 Analysis at the time of diagnostic resolution time point 49
14.2.2.3.2 Analysis at 12 months follow-up time point 49

CONFIDENTIALITY
This document is the confidential and proprietary information of GRAIL, LLC. Except as required by federal, state, or local laws or regulations, this document may not be disclosed to others, in whole or in part without prior written permission of GRAIL, Inc.

14.2.2.3.3 Analysis at 24 months time point 50
14.2.2.4 Evaluation of MCED-I Test Performance 50
14.2.2.4.1 Analysis at 12 months follow-up time point 50
14.2.2.4.2 Analysis at 24 months time point 50
14.3 Analysis Methods for Secondary Objectives 50
14.3.1 Participant reported outcomes and behavioral intentions 50
14.3.1.1 Evaluate intention and utilization of guideline recommended cancer screening procedures after use of the MCED test 50
14.3.1.2 Assess Participants Reported Anxiety Resulting from Use of the MCED Test 53
14.3.1.3 Assess participants' reported outcomes and perceptions about the MCED test 54
14.3.1.3.1 Adapted MICRA 55
14.3.1.3.2 SF-12v2 55
14.3.1.3.3 Satisfaction with MCED Test 55
14.3.1.3.4 Attitude Towards Subsequent MCED Test 55
14.3.2 Diagnostic evaluations 56
14.3.2.1 Characterize the number and type of diagnostic evaluations by predicted cancer signal origin and outcome of diagnostic resolution 56
14.3.2.2. Evaluate the radiation exposure due to the test result-directed evaluation 58
14.3.2.3 Describe the ability of diagnostic PET-CT to confirm cancer in participants where diagnostic resolution of cancer diagnosis is not achieved through the targeted diagnostic testing triggered by MCED test results 59
14.3.3 Test performance 59
14.3.3.1 Evaluate Performance of the MCED Test in Selected Demographic and Clinical Subgroups. 59
14.3.3.2 Evaluate MCED Test Positive Rate in Subgroups with Potential Cross-Reactive Conditions 61
14.3.3.3 Evaluate concordance of initial GRAIL test result and CSO prediction, with results from the research blood draw 62
14.4 Analysis Methods for Exploratory Objectives 62
14.4.1 Evaluate performance of the MCED test over three years of follow-up. 62
14.4.2 Evaluate changes in blood-based biomarkers among the subset of participants with a research blood draw. 63
14.4.3 Evaluate different CSO reporting classes based on clinically relevant subgroups. 63
15. Analysis Methods for Adverse Events 63
16. Protocol Deviations 63

CONFIDENTIALITY
This document is the confidential and proprietary information of GRAIL, LLC. Except as required by federal, state, or local laws or regulations, this document may not be disclosed to others, in whole or in part without prior written permission of GRAIL, Inc.

17. Sample Size and Power **64**
Appendix 1 PATHFINDER 2 Inclusion/Exclusion Criteria **67**
Appendix 2 Participant-reported outcome data collection time points **68**
Appendix 3A CSO predictions for the MCED-V2 test **69**
Appendix 3B MCED-V2 CSO specification **70**
Appendix 4A CSO predictions for the MCED-I test **92**
Appendix 4B CBS Predictions for MCED-I Test **93**
Appendix 5A Crosstabulation of MCED-I and MCED-V2 CSO Predictions **94**
Appendix 5B: Matrix of MCED-I CSO Predictions and Corresponding MCED-V2 CSO Predictions among test positives for both tests **95**
Appendix 6A MCED-I CSO Specification **97**
Appendix 6B MCED-I CBS Specification **118**
Appendix 7 Prespecified Cancer Subgroups **121**
Appendix 8 Cancer Type Specifications **123**
Appendix 9 Sample Size and Power for Bridging Analysis, Stratification Factors, Approximate Sampling and Analysis Weights **127**
Appendix 10 Examples of invasive procedures **130**
Appendix 11 Recommended diagnostic evaluations by CSO prediction **131**
Appendix 12 USPSTF cancer screening recommendations **134**
Appendix 13 Description of Participant-Directed Questionnaires. **135**
Appendix 14 Estimated effective radiation dose estimates for imaging tests. **138**

CONFIDENTIALITY
This document is the confidential and proprietary information of GRAIL, LLC. Except as required by federal, state, or local laws or regulations, this document may not be disclosed to others, in whole or in part without prior written permission of GRAIL, Inc.

1. Definitions and Abbreviations

Term	Definition
AE	Adverse event
AJCC	American Joint Committee on Cancer
BMI	body mass index
CBS	cancer biology signal
CSO	cancer signal origin
CCGA	Circulating Cell-free Genome Atlas (NCT02889978)
cfDNA	cell-free deoxyribonucleic acid
CI	confidence interval
CSR	clinical study report
UPS	(cancer of) unknown primary site
eCRF	electronic case report form
EDC	electronic data capture
EMR	electronic medical record
FN	false negative
FP	false positive
ICD-O	International Classification of Diseases for Oncology
IDE	investigational device exemption
IDMC	Independent Data Monitoring Committee
LDCT	low-dose computed tomography
MICRA	Multidimensional Impact of Cancer Risk Assessment
MCED	multi-cancer early detection
NCCSO	No clinical cancer signal origin

CONFIDENTIALITY
This document is the confidential and proprietary information of GRAIL, LLC. Except as required by federal, state, or local laws or regulations, this document may not be disclosed to others, in whole or in part without prior written permission of GRAIL, Inc.

Term	Definition
NCCN	National Comprehensive Cancer Network
NGS	next generation sequencing
NNTS	number needed to screen
NPV	negative predictive value
PET-CT	positron emission tomography-computed tomography scan
PMA	premarket application
PPV	positive predictive value
PRO	participant reported outcomes
PSA	prostate-specific antigen
SAE	serious adverse event
SAP	statistical analysis plan
SF-12	short form health survey
SOC	standard of care
STAI	State-Trait Anxiety Inventory
TNM	tumor, lymph node, metastasis description for cancer staging
TN	true negative
TP	true positive
USPSTF	United States Preventive Services Task Force
WHO	World Health Organization

CONFIDENTIALITY
This document is the confidential and proprietary information of GRAIL, LLC. Except as required by federal, state, or local laws or regulations, this document may not be disclosed to others, in whole or in part without prior written permission of GRAIL, Inc.

2. Background and Rationale

This statistical analysis plan (SAP) describes the statistical methods to be used in the analysis of GRAIL-012 The PATHFINDER 2 Study: Evaluating the Safety and Performance of the GRAIL Multi-Cancer Early Detection Test in an Eligible Screening Population. The study is conducted under an IDE (G210176) and is registered at clinicaltrials.gov (NCT05155605).

PATHFINDER 2 is a prospective, interventional multi-center study of the GRAIL MCED test in healthcare systems in North America in which approximately 35,000 participants aged 50 years or older are expected to be enrolled. The purpose is to evaluate the safety and performance of the GRAIL MCED test in an eligible screening population. Safety for the MCED test will be evaluated during the study in terms of diagnostic procedures performed during the diagnostic journey, including imaging, biopsy, endoscopy, and surgery, in participants with positive MCED test results and adverse events reported for all study participants.

The GRAIL MCED test version 2.0 (henceforth referred to as MCED-V2) will be ordered by and results returned to a study investigator. In cases with a “cancer signal detected” test result (henceforth referred to interchangeably as “positive” or “cancer signal detected” result), the diagnostic work-up will be coordinated by the study medical team at the enrolling sites. In cases with a “no cancer signal detected” test result (henceforth referred to interchangeably as “negative” or “no cancer signal detected” result), participants will be instructed not to interpret negative test result as the absence of cancer. All participants are encouraged to continue to adhere to guideline-recommended cancer screening tests. Study participants will be followed for 3 years after enrollment with annual assessments of cancer status and utilization of guideline-recommended (routine) cancer screening tests.

A subset of blood samples collected from PATHFINDER 2 participants will be analyzed retrospectively with the final to-be-marketed version of the GRAIL MCED test (version 3.0, henceforth referred to as MCED-I) in order to demonstrate concordance between the two versions of the test. The sampling scheme and rationale for the sample size of the subset selected for MCED-I analysis are included in this document.

This SAP will describe the methods for the following analyses:

1. Safety and test performance for MCED-V2
2. Test performance for MCED-I
3. Concordance between MCED-V2 and MCED-I

3. Study Objectives

3.1 Primary Objectives

There are two primary study objectives:

1. Evaluate the safety of the MCED test in terms of diagnostic testing triggered by the MCED test result (Section 14.2.1).

CONFIDENTIALITY
This document is the confidential and proprietary information of GRAIL, LLC. Except as required by federal, state, or local laws or regulations, this document may not be disclosed to others, in whole or in part without prior written permission of GRAIL, Inc.

Safety for this objective will be evaluated in terms of the number and type of invasive procedures (Appendix 10) and the number and type of adverse events reported during diagnostic testing in participants with a positive MCED test result.

2. Evaluate performance of the MCED test in individuals eligible for cancer screening (Section 14.2.2).

   As part of the second primary objective, the study success criteria for PMA analysis will be evaluated for MCED-I test based on the 12 months follow-up time analysis period using hierarchical testing. For each criterion, the passing threshold represents the target which the lower bound of the two-sided 95% confidence interval needs to exceed.

   - First, two co-primary endpoints of episode sensitivity and specificity will be tested to demonstrate that episode sensitivity for all cancers is significantly higher than 10% (with lower bound of the two-sided 95% confidence interval >10%) and that specificity is significantly higher than 99% (with lower bound of the two-sided 95% confidence interval >99%). To claim success on the primary study success criteria, statistical significance needs to be achieved for both of the co-primary endpoints.
   - Subsequently, conditional on the success of the co-primary endpoints, accuracy of CSO prediction in true positives will be tested to demonstrate that it is significantly higher than 70% (with lower bound of the two-sided 95% confidence interval > 70%)

   The hierarchical testing sequence for MCED-I test study success criteria is illustrated in Figure 1 below.

Figure 1. MCED-I test study success criteria testing sequence

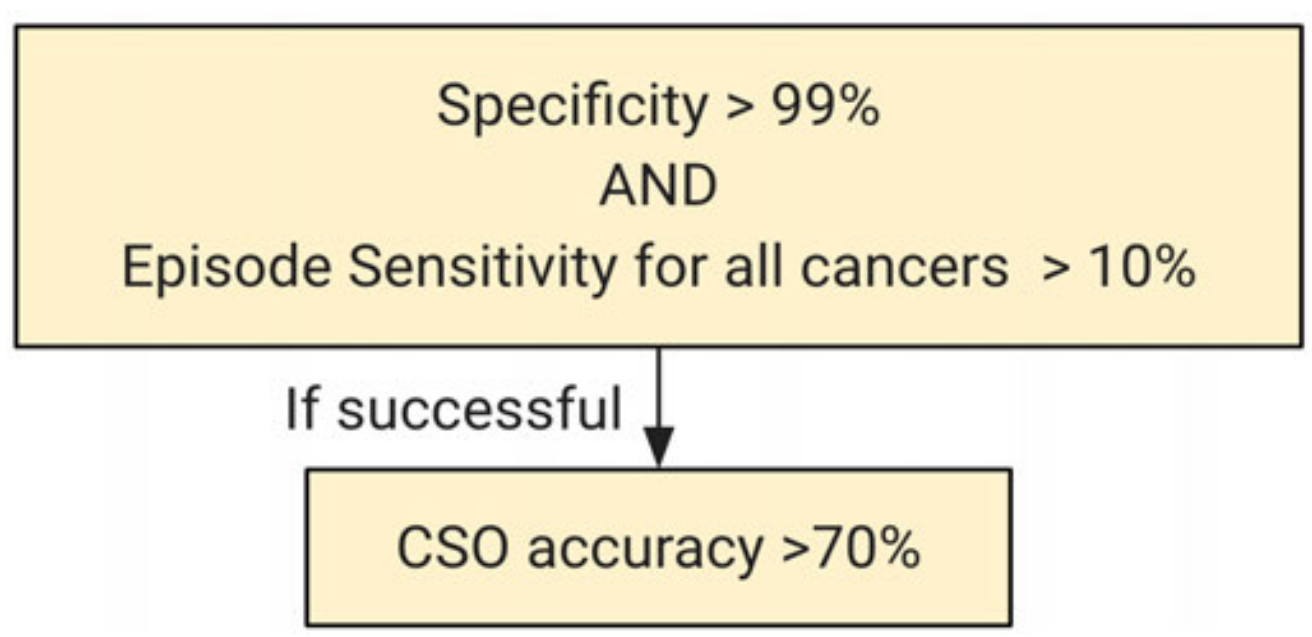

In addition, a set of clinically relevant cancer and CSO-specific performance endpoints will be provided descriptively for evaluation of MCED-I test performance.

3.2 Secondary Objectives

The secondary objectives of the study fall into three categories: participant reported outcomes and

CONFIDENTIALITY
This document is the confidential and proprietary information of GRAIL, LLC. Except as required by federal, state, or local laws or regulations, this document may not be disclosed to others, in whole or in part without prior written permission of GRAIL, Inc.

behavioral intentions, diagnostic evaluations, and test performance, as follows:

1. Participant reported outcomes and behavioral intentions

   1.1. Evaluate intention and utilization of guideline recommended cancer screening procedures after use of the MCED test (Section 14.3.1.1) (study protocol secondary objective 2).

   1.2. Assess participant-reported anxiety resulting from use of the MCED test (Section 14.3.1.2) (study protocol secondary objective 1).

   1.3. Assess participants' reported outcomes and perceptions about the MCED test (Section 14.3.1.3) (study protocol secondary objective 7).

2. Diagnostic evaluations

   2.1. Characterize the number and type of diagnostic evaluations by predicted cancer signal origin and outcome of diagnostic resolution (Section 14.3.2.1) (study protocol secondary objective 4).

   2.2. Evaluate the radiation exposure during diagnostic evaluation by imaging tests to inform safety (Section 14.3.2.2) (study protocol secondary objective 5).

   2.3. Describe the ability of diagnostic PET-CT to confirm cancer in participants where diagnostic resolution of cancer diagnosis is not achieved through the targeted diagnostic testing triggered by MCED test results (Section 14.3.2.3) (study protocol secondary objective 3).

3. Test performance

   3.1. Evaluate performance of the MCED test in selected demographic and clinical subgroups (Section 14.3.3.1) (secondary objective not included in the study protocol).

   3.2. Evaluate MCED test positive rate in subgroups with potential cross-reactive conditions (Section 14.3.3.2) (secondary objective not included in the study protocol)

   3.3. Evaluate concordance of initial GRAIL test result and Cancer Signal Origin (CSO) prediction, with results from the research blood draw (Section 14.3.3.3) (study protocol secondary objective 6).

3.3 Exploratory Objectives

The exploratory objectives of the study include:

1. Evaluate performance of the MCED test over three years of follow-up
2. Evaluate changes in blood based biomarkers among the subset of participants with a

CONFIDENTIALITY
This document is the confidential and proprietary information of GRAIL, LLC. Except as required by federal, state, or local laws or regulations, this document may not be disclosed to others, in whole or in part without prior written permission of GRAIL, Inc.

research blood draw

3. Evaluate different CSO reporting classes based on clinically relevant subgroups

4. Study Design

4.1 PATHFINDER 2 Study Design

This section briefly describes the design of the PATHFINDER 2 study. More details can be found in the study protocol.

PATHFINDER 2 is a prospective, interventional multi-center study of the GRAIL MCED test in which approximately 35,000 participants aged 50 years or older (inclusion/ exclusion criteria in Appendix 1) are expected to be enrolled at clinical sites in North America. The MCED test will be ordered by, and results returned to, a study investigator. In cases with a positive test result, the diagnostic work-up will be coordinated by the study medical team at the enrolling sites. Up to two cancer signal origin (CSO) predictions may be returned. The healthcare providers are strongly encouraged to evaluate participants for cancer based on the organ or anatomical location described by first CSO prediction using the CSO-based diagnostic evaluations described in the study protocol. If no cancer is identified based on the first CSO, the healthcare provider should evaluate the participant for the second CSO, if it was reported.

For participants where diagnostic resolution of cancer diagnosis is not achieved through the CSO-guided diagnostic testing and PET-CT has not been used as part of diagnostic evaluation, a full body (skull vertex to mid-thighs) diagnostic PET-CT will be performed to assess cancer status (referred to the 'confirmatory PET-CT' in the study protocol). Participants in this group will also undergo a research blood draw around the time of this diagnostic PET-CT. Results of this research blood draw will not be provided back to the physician or participant (per informed consent).

If a participant is unable to undergo the recommended diagnostic evaluations or the healthcare provider determines an alternative or additional test is necessary, the healthcare provider will document the reason for deviating from the recommended evaluation(s) and list the alternative and additional test(s) performed. In cases with a negative test result, participants are instructed not to interpret a negative test result as the absence of cancer and to continue to adhere to guideline-recommended cancer screening.

Safety for the MCED test will be evaluated during the study in terms of diagnostic procedures performed in participants with positive MCED test results and adverse events reported for all study participants.

Additionally, participant-reported outcomes (PRO) will be collected at several time points (Appendix 2) to assess participants' perceptions about the MCED test, including anxiety, health-related quality of life, and the potential impact of test results on attitude towards adherence to guideline-recommended screening.

Participants will be followed for three (3) years from the time of enrollment, and cancer status and utilization of guideline-recommended cancer screening will be assessed annually.

All study procedures were approved by a central or local Institutional Review Board.

CONFIDENTIALITY

This document is the confidential and proprietary information of GRAIL, LLC. Except as required by federal, state, or local laws or regulations, this document may not be disclosed to others, in whole or in part without prior written permission of GRAIL, Inc.

4.2 Bridging Analysis for MCED-I Test

In the PATHFINDER 2 study, results of the MCED-V2 test are returned to healthcare providers, and will guide diagnostic evaluation for cancer in participants with MCED-V2 test positive results. In addition, the MCED-I test will be performed on the frozen plasma samples from a subset of PATHFINDER 2 participants at a time that is at least 12 months after the date of the blood draw. This will enable evaluation of performance of the MCED-I test in the PATHFINDER 2 study, and the concordance between the MCED-V2 and MCED-I test versions.[1,2]

A sampling scheme will be implemented so that a subset of samples will be tested with the MCED-I test in order to ensure that the analysis results are representative of the overall study population. Plasma samples (if available) from the following groups of clinically eligible and clinically evaluable participants (as defined in section 10.2) will be selected:

- All participants with positive MCED-V2 test results
- All participants with cancer diagnosis during 12 months of follow-up time
- Samples from a subset of participants with negative MCED-V2 test results and no cancer diagnosis during 12 months of follow-up time and completed cancer status assessment at 12 months (as defined in section 14.1.3).

The subset selection of participants with negative MCED-V2 test results and no cancer diagnosis during 12 months of follow-up time will be stratified on the following factors (see Appendix 9 for details):

- Age
- Race
- Ethnicity
- Prior cancer history
- Smoking status
- Selected conditions that may affect cfDNA shedding or methylation such as inflammatory conditions (e.g. pancreatitis), use of demethylating agents or prior transplant

Stratification factors were chosen to provide sufficient representation of subsets of interest, especially relatively small subsets of the population that are important to characterize:

- Diversity and representativeness in terms of race and ethnicity
- MCED test performance in terms of false positive rate (FPR)

The detailed description of sampling strata, approximate size of the strata and sampling weights are summarized in Appendix 9.

---

[1] Principles for Codevelopment of an In Vitro Companion Diagnostic Device with a Therapeutic Product, Draft Guidance for Industry and Food and Drug Administration Staff, July 2016

[2] Li M. Statistical consideration and challenges in bridging study of personalized medicine. J Biopharm Stat. 2015;25(3):397-407. doi: 10.1080/10543406.2014.920340. PMID: 24897254

CONFIDENTIALITY

This document is the confidential and proprietary information of GRAIL, LLC. Except as required by federal, state, or local laws or regulations, this document may not be disclosed to others, in whole or in part without prior written permission of GRAIL, Inc.

5. Study Population

The study will enroll approximately 35,000 participants aged 50 years or older as described in the inclusion/exclusion criteria (see Appendix 1). The goal is to enroll a diverse participant population, generally representative of the US population aged 50 years and older with respect to race, ethnicity and sex. Target distributions for race, ethnicity, age and sex are listed in the study protocol (Section 6, Table 5).

If inclusion/exclusion criteria are modified through a protocol amendment, the latest inclusion/exclusion criteria available during preparation for analysis will be utilized for the main analysis. If changes to these criteria are substantial, an exploratory analysis may be conducted with earlier inclusion/exclusion criteria. SAP Appendix 1 will specify which inclusion/exclusion criteria will be used in the main analysis.

6. Data

This section briefly describes the types of data collected in the PATHFINDER 2 study. More details can be found in the PATHFINDER 2 study protocol.

6.1 Data Sources

The main sources of data in PATHFINDER 2 study include the following:

- Laboratory data generated from the MCED test
  - MCED-V2 test result
  - MCED-I test result

- Clinical data entered into and stored in the electronic case report forms (eCRF) within Medrio, a validated 21CFR Part 11 compliant electronic data capture system (EDC). These data are collected from:
  - Blood collection tube label
  - Test Requisition Form
  - Test Result Report
  - Participant electronic medical records (EMR)
  - Self-reported participant information for baseline study questionnaires
  - PRO questionnaires administered either online or by paper questionnaires, depending on participant ability to access PROs online and preference.

A more comprehensive description can be found in the PATHFINDER 2 study protocol, Clinical Data Management Plan, and eCRF Specifications.

6.2 Clinical Data

The clinical data will include participant related fields, demographics and participant information, clinical information, information about imaging, laboratory tests and invasive procedures, and tumor-related information. Examples of key clinical and demographic variables include:

CONFIDENTIALITY
This document is the confidential and proprietary information of GRAIL, LLC. Except as required by federal, state, or local laws or regulations, this document may not be disclosed to others, in whole or in part without prior written permission of GRAIL, Inc.

- Demographic and Participant Information: age, sex, race/ethnicity, personal and family history of cancer, smoking status, alcohol use, body mass index (BMI), cancer screening history.
- Clinical Information: type and number of diagnostic tests and procedures, outcome of diagnostic resolution, cancer status at 12, 24 and 36 months follow-up.
- Tumor Information: cancer type, clinical stage.

Tumor stage will be defined based on the clinical stage reported by clinical sites. Sites are instructed to report stage according to the staging system that is relevant for the type of cancer that is diagnosed.

PRO data include ratings for the following questionnaires (see details in Appendix 13) collected at various time points during the study (as described in Appendix 2):

- State Trait Anxiety Inventory (STAI) questionnaire and MCED STAI questionnaires
- Adapted Multidimensional Impact of Cancer Risk Assessment (MICRA)
- The 12-Item Short Form Health Survey version 2 (SF-12v2)
- Satisfaction with the MCED test
- Attitude towards adherence to guideline-recommended screening
- Attitude towards subsequent MCED test

Based on the collected information, we will derive and document the logic for defining variables in the dataset specifications per the GRAIL Analysis Variable Specifications Process standard operating procedure (SOP).

6.3 MCED Test Data

GRAIL's MCED test is a targeted methylation, next-generation sequencing (NGS)-based assay that analyzes cell-free DNA (cfDNA) isolated from peripheral blood. The test measures the extent and location of DNA methylation in the genome by sequencing a subset of genomic regions, and applies a computational algorithm to determine whether or not a shared cancer signal is present, regardless of cancer type. If a shared cancer signal is detected, the algorithm then makes a prediction of the origin(s) of the detected signal. CSO predictions are based on observed methylation patterns consistent with the biologic origins of a cancer, and are the result of the classifier, bioinformatics pipeline and medical device software. CSO predictions are intended to inform the workup of a positive MCED test result. An additional feature called Cancer Biology Signal (CBS) was introduced for MCED-I. CBS refers to observed cell-free DNA methylation patterns associated with specific cellular lineages that may be reported in addition to certain CSO predictions when a cancer signal is detected. CBS predictions are output by the bioinformatics pipeline and are intended to provide additional information during a diagnostic workup of a positive MCED test result.

Source data for sample processing will be curated and managed as described in the GRAIL Sample and Data Analysis Workflow SOP.

CONFIDENTIALITY
This document is the confidential and proprietary information of GRAIL, LLC. Except as required by federal, state, or local laws or regulations, this document may not be disclosed to others, in whole or in part without prior written permission of GRAIL, Inc.

The MCED-V2 Test data will be generated from processing one plasma tube for each participant. In case the assay fails, a back up tube may be used to rerun the assay. The MCED-V2 test results are returned to the study investigator. A similar process will be used to generate MCED-I test data.

All elements of the V2 assay including assay processing method, sample quality criteria, classifier and CSO reporting, were locked prior to sample processing. In addition, staff involved in assay result generation are blinded and do not have access to clinical data except for the limited information collected on the test requisition form (pregnancy, solid organ transplant and bone marrow transplant status) and/or used for generation of assay results (age (based on date of birth), sex) until the test report sign-off for release to study investigator.

All elements of the MCED-I test including assay processing method, sample quality criteria, classifier and signal origin reporting, will be locked prior to PATHFINDER 2 samples being processed with MCED-I test. The MCED-I classifier was locked after the start of the PATHFINDER 2 study. To ensure that MCED-I developers are not exposed to the MCED-V2 PATHFINDER 2 data, a study-specific Blinding Plan was developed according to the Clinical Study Data Blinding SOP.

The MCED-V2 test data were generated from processing sample data (refer to related Multi-Cancer Test V2 Automated Process and Multi-Cancer Test Sequencing documents for details) and through the bioinformatics analysis (refer to RES-DEV-0017, *Multicancer V2 Bioinformatics Pipeline Architecture and Design Specification* and related Multi-Cancer V2 Targeted Methylation Pipeline documents for details). Similarly, the MCED-I test data were generated from processing sample data (ARD-REF-0010, MCED-I System Assay User Guide) and through the bioinformatics analysis (RES-DEV, MCED-I Bioinformatics Pipeline Software Architecture and Design Specification and Multi-Cancer V2 Targeted Methylation Pipeline documents and related MCED-I Bioinformatics Pipeline Software documents).

6.4 Data Lock Process and Merge

MCED test data will undergo a quality control (QC) process. The data will need to pass the predefined sample QC criteria as documented in the GRAIL Multi-Cancer Test V2 Quality Specification and GRAIL Multi-Cancer Test V3 (MCED-I) Quality Specification.

Likewise, before being released for analysis, clinical data will undergo the cleaning and clinical data lock process including cross-functional approval of the clinical data approval form and export of the EDC data for analysis, as described in *CDM-SOP-0004, Clinical Data Lock Process*, the study-specific Clinical Data Lock Plan and the PATHFINDER 2 Clinical Data Management Plan. Site staff will be permitted to make changes to clinical data after each specified analysis. If data updates occur, additional review and Principal Investigator signature are required as part of the clinical data lock process prior to the inclusion of the data in a subsequent analysis. The eCRFs will be locked to remove edit access at the time of the end-of-study analysis.

After clinical data are cleaned and/or locked, the data will be deposited into TidyData, a repository of GRAIL MCED test data and clinical data with system infrastructure to assemble the data, maintain versions of the data, and manage access to the data, where it will be merged for analysis

CONFIDENTIALITY
This document is the confidential and proprietary information of GRAIL, LLC. Except as required by federal, state, or local laws or regulations, this document may not be disclosed to others, in whole or in part without prior written permission of GRAIL, Inc.

purposes. The PATHFINDER 2 TidyData Plan describes the flow of merging the GRAIL MCED test data and clinical data into the TidyData and its contents along with other features of TidyData.

Releasing the TidyData with merged GRAIL Multi-Cancer MCED-V2 test data, MCED-I test data and clinical data will be considered as unblinding to MCED-V2 and MCED-I test results and will follow the GRAIL Clinical Study Data Blinding SOP.

7. Measures to Minimize Bias

In this prospective longitudinal study, information related to demographics, clinical characteristics, diagnostic procedure types, counts, dates, and cancer diagnoses will be captured from the EMR, which will minimize most biases (e.g., recall bias, observer bias, missing data bias).

To assess bias in enrollment, GRAIL will monitor and continuously compare distributions of key demographic and clinical characteristics of the enrolled population to the target population. In particular, if there is disproportionately high enrollment of a particular stratum (based on age and sex), further enrollment of individuals in this stratum may be limited.

To minimize conscious and unconscious bias, test developers and SAP developers were blinded to the study data according to the PATHFINDER 2 Data Access and Blinding Plan.

8. Outcome Definitions

8.1 GRAIL MCED Test Result

The outcome of GRAIL's MCED test (irrespective of version) includes:

- A binary result of "Cancer signal detected" or "No cancer signal detected"
  - The terms "Cancer signal detected" and "positive" MCED test result are used interchangeably, and
  - The terms "No Cancer Signal Detected' and "negative" MCED test result are used interchangeably in this document.
- Cancer signal origin (CSO) prediction(s) for a positive test result

CSO predictions come from a predefined list of CSOs. Appendices 3 and 4 contain the full lists of CSOs for the MCED-V2 test and MCED-I test, respectively. CSO specifications in Appendix 6 describe which cancers are associated with each MCED-I CSO prediction. This allows the accuracy of the CSO predictions to be rigorously assessed against the clinical diagnosis of cancer in the study (clinical CSO).

The MCED-V2 test provides up to two CSO predictions to inform diagnostic workup. If there is a strong match between the observed methylation patterns and a CSO, then one CSO prediction is returned. Otherwise the two most likely CSO predictions are returned. In this SAP, CSO will be described as follows:

- The first CSO prediction is referred to as CSO1
- The second CSO prediction is referred to as CSO2 (if more than one CSO is returned by the MCED-V2 test)

CONFIDENTIALITY
This document is the confidential and proprietary information of GRAIL, LLC. Except as required by federal, state, or local laws or regulations, this document may not be disclosed to others, in whole or in part without prior written permission of GRAIL, Inc.

The MCED-I test will provide one CSO prediction, which will be referred to as CSO1.

In addition, for certain CSO predictions, the MCED-I test may return one CBS prediction. CBS predictions come from a predefined list of CBSs. The full list and description of CBS predictions for the MCED-I test are included in Appendices 4B and 6B.

8.2 Target Clinical Outcome

8.2.1 Cancer

The target clinical outcome of cancer in the study is defined as any of the following cancers diagnosed during the study period:

- Invasive solid tumors, excluding non-metastatic basal cell carcinoma and squamous cell carcinoma of the skin
- Hematologic malignancies:
    - Lymphoma
    - Lymphoid leukemia
    - Plasma cell neoplasm/Multiple myeloma
    - Myeloid neoplasm
    - Additional malignant hematologic conditions representing proliferative or dysplastic disorders or neoplasms with behavior code 3 based on ICD-O-3

Note that ICD-O-3 is used as a reference system for what is considered to be a hematologic malignancy. Documentation of the ICD-O-3 behavior code is not required to establish a diagnosis of cancer.

Cancer diagnosis is supported by pathologic confirmation of an invasive solid tumor or hematologic malignancy, or by imaging confirmation in the absence of pathology, or by assigned clinical stage of I-IV by the treating physician, or by other clinical confirmation of disease per treating clinician (e.g. biochemical evidence of recurrence of prior cancer).

Cancer status for a specific analysis period is determined based on whether or not a participant has at least one confirmed clinical outcome of cancer (cancer participant vs non-cancer participant) in the specific analysis period (as defined in Sections 8.2.8 and 8.2.9).

8.2.2 Cancer Clinical Stage

A cancer is considered stageable if a recognized staging system exists for its histology (morphology) with or without anatomic site (topography). The “stageable cancer” will be used in descriptive summaries of cancer type/ stage distribution.

Clinical stage is determined by the healthcare providers through clinical information, pathology reports and/or imaging findings for new primary cancers. Cancers that do not have a tumor, node, metastasis (TNM)-type staging system defined in the AJCC cancer staging manual[3], such as

[3] Amin MB, Edge S, Greene F, Byrd DR, Brookland RK, Washington MK, Gershenwald JE, Compton CC, Hess KR, et al.

CONFIDENTIALITY
This document is the confidential and proprietary information of GRAIL, LLC. Except as required by federal, state, or local laws or regulations, this document may not be disclosed to others, in whole or in part without prior written permission of GRAIL, Inc.

cancers of the brain and spinal cord, lymphoid leukemias, myeloid neoplasms and additional malignant hematologic conditions, will be analyzed based on the clinical stage provided by the study site using the relevant staging system for each condition, if applicable. If a clinical stage is not available, a pathologic stage based on an abstracted pathology report will be used.

For recurrent cancers, the extent of recurrence is documented as local, lymph nodes (regional) or distant (metastatic).

8.2.3 Cancer Type

For each observed clinical cancer diagnosis, a cancer type is assigned. Cancer types are listed in Appendix 8. Cancers that are not included in the defined cancer types are reported under the "Other" category. Cancer type is a clinical construct for staging, prognosis and treatment, and is used in the context of data analysis and results presentation.

Participants who do not have a cancer type assigned will be considered to have "Unassigned" cancer type.

8.2.4 Clinical CSO

For each observed cancer diagnosis, a clinical CSO may be assigned based on histologic type (morphology), anatomic site (topography) and behavior code according to ICD-O-3. The Clinical CSO represents the clinical truth of the CSO as determined by clinical diagnosis. The clinical CSO is used to evaluate whether a MCED CSO prediction was accurate. CSO definitions relevant to CSO predictions and clinical CSOs are documented in the CSO specification (Appendix 3B for MCED-V2 and Appendix 6A for MCED-I).

There are a number of cancer types which are not included in the CSO specifications. Typically these are less common and rare cancers (e.g., cancers which fall into the "Other" cancer type including penis, eyelid carcinoma). For both test versions, the CSO classifier has not been trained on cfDNA methylation data from these cancers since they are insufficiently common to warrant a separate CSO and have methylation biology or clinical work-up that is too different to be merged with existing CSOs. However, cancer signal detection of such cases is still expected due to shared cancer signal, and consequently it is important to demarcate these categories. These will be considered "Cancer type without clinical CSO". This is distinct from the scenario where the study cancer data is insufficient to assign a clinical CSO; these will be considered "Unassigned clinical CSO". Note that many of these cancers may be detected by the test since they are included in training of the Cancer Signal Detection classifier and also because a portion of the methylation signal may be shared across cancer types.

8.2.5 Clinical CBS

For MCED-I, an additional prediction output called clinical Cancer Biology Signal (CBS) was introduced. The clinical CBS reflects cell-free DNA methylation patterns associated with oncogenic

---

(Eds.). AJCC Cancer Staging Manual (8th edition). Springer International Publishing: American Joint Commission on Cancer; 2017.

CONFIDENTIALITY

This document is the confidential and proprietary information of GRAIL, LLC. Except as required by federal, state, or local laws or regulations, this document may not be disclosed to others, in whole or in part without prior written permission of GRAIL, Inc.

drivers (e.g. HPV), histology, or specific cellular lineages. Clinical CBS are assigned based on ICD-O-3 topography, morphology, and behavior code data using predefined assignment rules. Consequently, the accuracy of CBS predictions can be assessed in diagnosed cancer patients within the study.

There are a number of cancer types which are not included in the CBS specifications (Appendix 6B). These cancer types are used to train the Cancer Signal Detection classifier but may not have been used to train the CBS classifier. These will be considered “Cancer type without clinical CBS”. This is distinct from the scenario where the study cancer data are insufficient to assign a clinical CBS; these will be considered “Unassigned clinical CBS”. Note that CBS predictions are only returned in instances where there is additional information in the methylation signal that is compatible with the CSO prediction and may help guide the diagnostic workup.

8.2.6 Cancer of Unknown Primary Site

A cancer of unknown primary site (UPS) will not be assigned a clinical CSO. A cancer with UPS diagnosed during the specific analysis period will be considered for analysis. UPS cancers are unstageable; if the cancer primary site is unknown then it cannot be staged under the appropriate system. UPS cancer will have “Unassigned clinical CSO” and “Unassigned clinical CBS”. Specific considerations with respect to UPS will be discussed at the definition of each endpoint.

8.2.7 Multiple Cancers

Participants with more than one cancer diagnosed during the analysis period are considered to have multiple cancers, irrespective of whether these were recurrent or new primary cancers. For example, participants with a prior cancer history who had both a recurrent cancer and a new subsequent primary cancer diagnosed during the analysis period will be included in the multiple cancers group. Participants with a prior cancer history who are diagnosed with a new subsequent primary cancer during the analysis period and who do not have any additional cancer diagnoses (i.e., no recurrences) will not be included in the multiple cancers group for the purposes of this analysis.

Participants with multiple cancers may have multiple clinical CSOs.

When test performance endpoints are summarized by cancer type, participants with multiple cancers are treated as separate cancer types, namely ‘Multiple Cancers’, besides the cancer types defined in Appendix 8. In order to avoid double counting, participants for whom there were multiple cancer diagnoses where at least one of them was UPS or unassigned are included in the ‘Multiple Cancers” category, for reporting purposes. In addition, as a sensitivity analysis for test performance endpoints by cancer type, participants will be categorized based on the first diagnosed cancer as the cancer type.

8.2.8 Definition of Cancer Participant for Specific Analysis

A cancer participant is defined as a participant for whom a cancer diagnosis (e.g., at least one clinical outcome of “cancer” as defined in section 8.2.1) has been confirmed within a specific analysis period (e.g. 12 months follow-up time analysis period for PMA analysis).

CONFIDENTIALITY
This document is the confidential and proprietary information of GRAIL, LLC. Except as required by federal, state, or local laws or regulations, this document may not be disclosed to others, in whole or in part without prior written permission of GRAIL, Inc.

8.2.9 Definition of Non-cancer Participant for Specific Analysis

The clinical outcome of non-cancer is defined as absence of the diagnosis of cancer as described in section 8.2.1. Absence of cancer diagnosis is determined at several time points for participants with MCED-V2 positive test results: at the time of the diagnostic resolution and at annual assessments. For participants with MCED-V2 negative test results, absence of cancer diagnosis is determined at the annual assessments only.

Non-cancer status in participants with MCED-V2 negative test results is determined by review of the medical records at approximately 12, 24, and 36 months after the blood draw for evidence of cancer diagnosis in the participant's EMR or participant self-report through a phone call. See the study protocol for additional details of the follow-up process.

Thus a non-cancer participant is defined as a participant for whom an absence of cancer diagnosis is determined at the end of the specific analysis period (e.g. 12 months follow-up time analysis period for PMA analysis).

9. Handling of Dropouts or Missing Data

We expect the key demographic and baseline clinical characteristics that will be used to describe the cohort (e.g., age, race/ethnicity, sex) to be available for the majority of the participants (<5% missing data expected). The number of participants with missing clinical characteristics will be provided.

Cancer staging is determined by study sites through clinical information, pathology reports and/or imaging findings (<5% missing data expected for cancers which have an AJCC TNM-type staging system). We will report the number of cancer participants with data for cancer stage.

Approximately 5% of non-cancer participants in this study are expected not to have a 12-month cancer status assessment. A similar proportion (5%) of the remaining participants is expected not to complete 24-month cancer assessment and 36-month cancer assessment, respectively. This loss to follow-up is estimated based on the previously conducted similar study[4].

As described in Section 14, only complete case analysis, i.e. analysis of participants with completed cancer status assessment at the end of the analysis period, will be used to evaluate test performance.

Sensitivity analyses that include participants lost to follow-up will be conducted for measures such as time to diagnostic resolution and PPV.

10. Analysis Sets

Analysis sets define the participants to be included in an analysis. Analysis sets (including subsets

[4] Schrag et. al. Blood-based tests for multicancer early detection (PATHFINDER): a prospective cohort study, Lancet, 2023 Oct 7;402(10409):1251-1260

CONFIDENTIALITY
This document is the confidential and proprietary information of GRAIL, LLC. Except as required by federal, state, or local laws or regulations, this document may not be disclosed to others, in whole or in part without prior written permission of GRAIL, Inc.

for specific analyses) and their definitions are provided in this section.

10.1 Consented Analysis Set

This set comprises all participants who consented to take part in the study. The summaries specified in Section 13.4 (Participant Disposition) will be based on consented participants.

10.2 Clinically Eligible and Clinically Evaluable Analysis Set

This set comprises consented participants who are clinically eligible (satisfy the inclusion and exclusion criteria) and are clinically evaluable.

First, participants who satisfy study inclusion/exclusion criteria (Appendix 1) are determined. During the data cleaning process, it is possible that some consented participants who did not satisfy study inclusion/exclusion criteria are identified. For example, a study site may determine that a study participant had a history of invasive cancer less than 3 years prior to study enrollment. Such participants will be considered as eligibility failures and excluded from the clinically evaluable analysis set.

Second, consented participants who satisfy study inclusion/exclusion criteria and clinical evaluability criteria are determined. The clinical evaluability criteria are described in the PATHFINDER 2 Clinical Data Management Plan. Examples include the presence of an evaluable blood draw.

Participants who withdraw consent after the MCED-V2 test result becomes available may be eligible for inclusion in some of the analyses. See Section 14 for details.

10.3 Analyzable Sets

Participants who were in the Clinically Evaluable Analysis Set and had an evaluable MCED-V2 test result will comprise the MCED-V2 Analyzable Set.

Participants who were in the Clinically Evaluable Analysis Set and had an evaluable MCED-I test result will comprise the MCED-I Analyzable Set.

10.4 Cancer Signal Detected Analysis Sets

The MCED-V2 Cancer Signal Detected Analysis Set will be composed of MCED-V2 analyzable participants with positive MCED-V2 test results.

The MCED-I Cancer Signal Detected Analysis Set will be composed of MCED-I analyzable participants with positive MCED-I test results.

10.5 No Cancer Signal Detected Analysis Sets

The MCED-V2 No Cancer Signal Detected Analysis Set will be composed of analyzable participants with negative MCED-V2 test results.

CONFIDENTIALITY

This document is the confidential and proprietary information of GRAIL, LLC. Except as required by federal, state, or local laws or regulations, this document may not be disclosed to others, in whole or in part without prior written permission of GRAIL, Inc.

The MCED-I No Cancer SIgnal Detected Analysis Set will be composed of analyzable participants with negative MCED-I test results.

11. Study Analyses

Several analyses are planned for the PATHFINDER 2 study: PMA analysis, first post PMA analysis, additional post-PMA analyses and IDMC analyses. Study objectives addressed in the PMA-related analyses are described in Table 1.

Table 1. Study objectives in PMA-related analyses.

Objective type	N	Study objective	PMA analysis	First post PMA analysis	Additional post PMA analyses
**Primary**	1	Evaluate the safety of the MCED test in terms of diagnostic testing triggered by the MCED test result	✓	✓	
	2	Evaluate performance of the MCED Test in individuals eligible for cancer screening	✓	✓	
**Secondary**	1	**Participant reported outcomes and behavioral intentions**			
	1.1	Evaluate intention and utilization of guideline recommended cancer screening procedures after use of the MCED test	✓	✓	✓
	1.2	Assess participant-reported anxiety resulting from use of the MCED test	✓	✓	
	1.3	Assess participants' reported outcomes and perceptions about the MCED test	✓	✓	
	2	**Diagnostic evaluations**			
	2.1	Characterize the number and type of diagnostic evaluations by predicted cancer signal origin and outcome of diagnostic resolution	✓	✓	
	2.2	Evaluate the radiation exposure during diagnostic evaluation by imaging tests to inform safety	✓	✓	
	2.3	Describe the ability of diagnostic PET-CT to confirm cancer in participants where diagnostic resolution of cancer diagnosis is not achieved through the targeted diagnostic testing triggered by MCED test results	✓	✓	

CONFIDENTIALITY
This document is the confidential and proprietary information of GRAIL, LLC. Except as required by federal, state, or local laws or regulations, this document may not be disclosed to others, in whole or in part without prior written permission of GRAIL, Inc.

Objective type	N	Study objective	PMA analysis	First post PMA analysis	Additional post PMA analyses
	3	**Test performance**			
	3.1	Evaluate performance of the MCED test in selected demographic and clinical subgroups	✓	✓	
	3.2	Evaluate MCED Test Positive Rate in Subgroups with Potential Cross-Reactive Conditions	✓		
	3.3	Evaluate concordance of initial GRAIL test result and Cancer Signal Origin (CSO) prediction, with results from the research blood draw		✓	
**Selected exploratory**	1	Evaluate performance of the MCED test over three years of follow-up.			✓

11.1 PMA Analysis

The first analysis of the PATHFINDER 2 study will take place when the majority of the consented participants will have 12 months of follow-up time after the blood draw.

This analysis is expected to include approximately 25,000 participants with 12 months of follow-up time and the remaining approximately 10,000 enrolled participants with at least 4 months of follow-up time after the blood draw. It is expected that most MCED test positive participants will finish their diagnostic evaluation in less than 6 months. Therefore, safety of the diagnostic evaluations (primary objective 1 [safety], secondary objective 2 [2.1 extent of diagnostic testing, 2.2 radiation exposure, 2.3 PET-CT]); as described in Table 1) will be summarized for 25,000 participants with 12 months of follow-up, and for 35,000 participants. Test performance (primary objective 2) and secondary objective 1 (1.1 intention and utilization of guideline recommended cancer screening, 1.2 anxiety and 1.3 PROs) as described in Table 1 will be evaluated for the 25,000 participants with 12 months of follow-up. In addition, PPV and CSO Accuracy will be assessed for MCED-V2 on 35,000 participants.

This analysis will serve as the basis for the review of the Pre-Market Application (PMA) to establish the safety and evaluate performance of the MCED test.

11.2 First Post PMA Analysis

The first post PMA analysis will take place when all approximately 35,000 consented participants will have 12 months of follow-up time after the blood draw. This analysis will provide additional evidence for the MCED-I test performance.

CONFIDENTIALITY
This document is the confidential and proprietary information of GRAIL, LLC. Except as required by federal, state, or local laws or regulations, this document may not be disclosed to others, in whole or in part without prior written permission of GRAIL, Inc.

11.3 Additional Post PMA Analyses

Additional analyses of the PATHFINDER 2 study will include all analyzable participants with

- 24 months of follow-up time
- 36 months of follow-up time

11.4 IDMC Analyses

An independent Data Monitoring Committee (IDMC) composed of external clinical and statistical experts will monitor the safety of the interventions in the PATHFINDER 2 study, evaluate participant recruitment and accrual and make recommendations to GRAIL regarding the continuation, modification, or termination of the study according to its charter. Several interim analyses of the PATHFINDER 2 study for the IDMC are planned when approximately 4,000, 9,000, 16,000 and 25,000 participants are enrolled. No formal evaluation of MCED test performance is planned at these interim time points. Data reviews will be scheduled based on the enrollment progress, and data for participants enrolled up to a projected date will be reviewed. Additional IDMC data reviews may be requested by the IDMC members and/or GRAIL.

For the purposes of the IDMC interim analyses described in this section, data will undergo cleaning, including cross-functional approval of the clinical data export of EDC data for analysis, as described in CDM-SOP-0004 Clinical Data Lock Process and the study-specific Clinical Data Cleaning Plan.

12. Multiplicity and Multiple Hypothesis Testing

At the time of the PMA analysis, the study success criteria for MCED-I test performance will be evaluated by comparing the lower bound of the two-sided 95% CI to the study success criteria threshold, following a hierarchical testing approach (as described in Sections 3.1 and 14.2.2) and therefore no type I error adjustment is needed.

No hypothesis testing is planned for first post PMA Analysis, additional post PMA analyses or IDMC analyses and therefore no type I error adjustment is needed.

13. Study Cohort Characteristics and Participant Disposition

13.1 General Considerations

Analysis results will be presented using descriptive statistics. For categorical (including binary) variables, the number and percentage of participants in each category will be presented. For continuous variables, the number of participants (n), mean, standard deviation (SD), median, first quartile (Q1), third quartile (Q3), minimum, and maximum will be presented.

CONFIDENTIALITY
This document is the confidential and proprietary information of GRAIL, LLC. Except as required by federal, state, or local laws or regulations, this document may not be disclosed to others, in whole or in part without prior written permission of GRAIL, Inc.

13.2 Enrollment

Enrollment by network and site will be summarized using descriptive statistics in the enrolled participants.

13.3 Demographics and Baseline Characteristics

Participant demographics and baseline characteristics will be summarized for the following groups in the MCED-V2 Analyzable Set and MCED-I Analyzable Set, separately:

- Overall
- By MCED test result: positive vs negative

Participant characteristics will be summarized using descriptive statistics for the following variables:

1. Age (at enrollment) as a continuous variable (years)
2. Age groups (50-59, 60-69,70-79, 80+ years) and (50-64 and 65+ years)
3. Biological sex (male, female)
4. Gender identity (male, female, transgender, non-binary, other, choose not to respond)
5. Sexual orientation (heterosexual, gay or lesbian, bisexual, other, choose not to respond)
6. Race categories aligned with FDA guidance[5]
    - American Indian or Alaska Native [6] only
    - Asian only
    - Black or African American only
    - Native Hawaiian or Other Pacific Islander only
    - White[7] only
    - More than one race reported
7. Race categories for full representation, i.e. if reported alone or as part of multiple race categories (not mutually exclusive):
    - American Indian or Alaska Native
    - Asian, Native Hawaiian or Other Pacific Islander
    - Black or African American
    - White
8. Ethnicity (Hispanic or Latino vs not Hispanic or Latino)
9. Marital status (never married, married or domestic partnership, previously married, separated, divorced, widowed)
10. Highest level of education completed (less than high school degree, high school degree or equivalent, some college, bachelor's degree or higher)
11. Smoking status (never smoker, former smoker, current smoker)
12. United States Preventive Services Task Force (USPSTF) lung cancer risk indicator (Yes, No) defined as follows:

---

[5] Race categories are aligned with the FDA guidance 'Collection of Race and Ethnicity Data in Clinical Trials and Clinical Studies for FDA-Regulated Medical Products', 2024

[6] This category includes participants who reported their race as North American Indigenous in order to accommodate a clinical site in Canada, where a different nomenclature is used in census.

[7] The US census includes persons having origins in any of the original peoples of Europe, the Middle East, or North Africa.

CONFIDENTIALITY

This document is the confidential and proprietary information of GRAIL, LLC. Except as required by federal, state, or local laws or regulations, this document may not be disclosed to others, in whole or in part without prior written permission of GRAIL, Inc.

- Participants who met the draft updated USPSTF recommendation for low-dose computed tomography (LDCT) screening published on July 7, 2020: adults aged 50 to 80 years who have at least a 20 pack-year smoking history and currently smoke or have quit within the past 15 years, will be assigned a “Yes”.

13. Alcohol use (none, light, moderate, heavy)
14. Prior history of cancer (Yes, No)
15. History of cancer in a first-degree relative (biologic parents, siblings or children) (Yes, No)
16. Presence of genetic cancer predisposition (Yes, No)
17. Body-mass index (BMI) categories (<25 kg/m^2, 25 to <30 kg/m^2, ≥30 kg/m^2)
18. Prior history of in situ cancer (Yes, No)
19. Prior history of benign hematologic condition (Yes, No)
    - Langerhans cell histiocytosis
    - Lymphoproliferative disorder
    - Mastocytoma
    - Monoclonal B-cell lymphocytosis
    - Monoclonal gammopathy of undetermined significance
    - Post-transplant lymphoproliferative disorder
    - Other
20. Prior history of selected medical conditions (Yes, No)
    - Acute pancreatitis
    - Barrett's esophagus
    - Chronic gastritis
    - Chronic hepatitis B infection
    - Chronic hepatitis C infection
    - Chronic obstructive pulmonary disease (COPD), emphysema or chronic bronchitis
    - Chronic pancreatitis
    - Cirrhosis
    - Crohn's disease or ulcerative colitis
    - Diabetes
    - Esophageal reflux disease
    - Fatty liver disease
    - Human immunodeficiency virus (HIV)
    - Liver cirrhosis
    - Multiple sclerosis
    - Rheumatoid arthritis
    - Stomach ulcers
    - Systemic lupus erythematosus (lupus)
21. Use of cytotoxic medications in the past year (Yes, No)
22. Use of demethylating agents in the past year (Yes, No)
23. Use of hormone therapy in the past 90 days (Yes, No)

13.4 Participant Disposition

A summary of participant disposition will be provided for all PATHFINDER 2 participants. This summary will present the number and percentage of participants in each of the categories listed below:

CONFIDENTIALITY
This document is the confidential and proprietary information of GRAIL, LLC. Except as required by federal, state, or local laws or regulations, this document may not be disclosed to others, in whole or in part without prior written permission of GRAIL, Inc.

- Consented Analysis Set
    - Not clinically eligible
    - Clinically eligible and not clinically evaluable
- Clinically Evaluable Analysis Set
    - No evaluable assay results
- MCED-V2 Analyzable Set
    - MCED-V2 Cancer Signal Detected Analysis Set
    - MCED-V2 No Cancer Signal Detected Analysis Set
- MCED-I Analyzable Set
    - MCED-I Cancer Signal Detected Analysis Set
    - MCED-I No Cancer Signal Detected Analysis Set

In addition, the number of participants with and without a cancer diagnosis in the MCED-V2 Cancer Signal Detected Analysis Set, MCED-V2 No Cancer Signal Detected Analysis Set, MCED-I Cancer Signal Detected Analysis Set, and MCED-I No Cancer Signal Detected Analysis Set will be reported.

14. Statistical Analysis Methods

This section will describe the statistical analysis methods for the evaluation of the PATHFINDER 2 study objectives.

14.1 General Considerations

14.1.1 Study Objectives and Analysis Sets

Table 2 describes the analysis sets used for the primary, secondary and selected exploratory study objectives.

Table 2. Study objectives and analysis sets.

Objective type	N	Study objective	Analysis Set
**Primary**	1	Evaluate the safety of the MCED test in terms of diagnostic testing triggered by the MCED test result	MCED-V2 Analyzable Set
	2	Evaluate performance of the MCED Test in individuals eligible for cancer screening	MCED-V2 Analyzable Set<br>MCED-I Analyzable Set
**Secondary**	1	**Participant reported outcomes and behavioral intentions**	
	1.1	Evaluate intention and utilization of guideline recommended cancer screening procedures after use of the MCED test	MCED-V2 Analyzable Set
	1.2	Assess participant-reported anxiety resulting from use of the MCED test	MCED-V2 Analyzable Set

CONFIDENTIALITY
This document is the confidential and proprietary information of GRAIL, LLC. Except as required by federal, state, or local laws or regulations, this document may not be disclosed to others, in whole or in part without prior written permission of GRAIL, Inc.

Objective type	N	Study objective	Analysis Set
	1.3	Assess participants' reported outcomes and perceptions about the MCED test	MCED-V2 Analyzable Set
	2	**Diagnostic evaluations**	
	2.1	Characterize the number and type of diagnostic evaluations by predicted cancer signal origin and outcome of diagnostic resolution	MCED-V2 Cancer Signal Detected Analysis Set
	2.2	Evaluate the radiation exposure during diagnostic evaluation by imaging tests to inform safety	MCED-V2 Cancer Signal Detected Analysis Set
	2.3	Describe the ability of diagnostic PET-CT to confirm cancer in participants where diagnostic resolution of cancer diagnosis is not achieved through the targeted diagnostic testing triggered by MCED test results	MCED-V2 Cancer Signal Detected Analysis Set
	3	**Test performance**	
	3.1	Evaluate performance of the MCED test in selected demographic and clinical subgroups	MCED-V2 Analyzable Set<br>MCED-I Analyzable Set
	3.2	Evaluate MCED test positive rate in subgroups with potential cross-reactive conditions	MCED-V2 Analyzable Set<br>MCED-I Analyzable Set
	3.3	Evaluate concordance of initial GRAIL test result and CSO prediction, with results from the research blood draw	MCED-V2 Cancer Signal Detected Analysis Set<br>MCED-I Analyzable Set
**Exploratory (selected)**	1	Evaluate performance of the MCED test over three years of follow-up	MCED-V2 Analyzable Set<br>MCED-I Analyzable Set

14.1.2 Subgroup Considerations and Confidence Intervals

Distribution of variables will be presented using descriptive statistics as described in Section 13.1.

If the number of participants within a certain subgroup (e.g. cancer type) is less than 5 (five), performance endpoints such as episode sensitivity or PPV for the corresponding subgroups will not be reported individually in all analyses, unless otherwise specified. Instead, the results for these subgroups with small sample sizes (<5) will be provided in the by-participant listings in the clinical study report (CSR).

CONFIDENTIALITY
This document is the confidential and proprietary information of GRAIL, LLC. Except as required by federal, state, or local laws or regulations, this document may not be disclosed to others, in whole or in part without prior written permission of GRAIL, Inc.

For MCED-V2, two-sided 95% confidence intervals (CIs) will be constructed using the Wilson (score) method[8,9,10] for performance measures such as PPV which are not expected to be very near 0 or 1. Modified Wilson (score) confidence intervals[11,12,13] will be used for performance measures such as specificity and NPV which are expected to be near 0 or 1.

For MCED-I:

- For test performance endpoints based on participants with cancer with analysis weights =1 (see Appendix 9 for details on analysis weights) confidence intervals will be constructed as described above for MCED-V2. Examples of such endpoints include episode sensitivity and CSO accuracy.
- For test performance endpoints based on participants with analysis weights not equal to 1 which are expressed as proportions (specificity, FPR, PPV, NPV), confidence intervals will be constructed using logit transformation[14]. Logistic regression models will be fitted with intercept only. To account for inverse probability weights used in the logistic regression models, the robust sandwich variance estimator will be used[15,16]. Confidence intervals for the intercept on the logit scale will then be transformed back to the scale of test performance endpoint.
- For test performance endpoints based on participants with analysis weights not equal to 1 which are expressed as ratios (PLR, NLR), confidence intervals will be constructed using the delta method.

The analyses will be conducted using software R version 3.6.0 or later.

14.1.3 Cancer Status Assessments and Loss to Follow-Up

Cancer status assessment will be conducted annually:

1. Assessment of cancer status for the 12 months of follow-up is considered to be completed (i.e. no loss to follow-up) when
    - cancer diagnosis date is within the first 12 months of follow-up period, or

[8] Wilson, E. B. (1927). Probable Inference, the Law of Succession, and Statistical Inference. *Journal of the American Statistical Association, 22*(158), 209-212
[9] EP12-A3 (2023), Evaluation of Qualitative, Binary Output Examination Performance; Approved Guideline-Third Edition. CLSI document EP12-A3. Budd J.A et al. Clinical Laboratory Standards Institute; 2023.
[10] FDA (2007), "Guidance for industry and FDA staff: Statistical guidance on reporting results from studies evaluating diagnostic tests," Silver Spring: US Food and Drug Administration.
[11] Newcombe, R. G. (1998a), "Two-sided confidence intervals for the single proportion: Comparison of seven methods," *Statistics in Medicine*, 17, 857–872
[12] Newcombe, R. G. (1998b), "Interval estimation for the difference between independent proportions: Com- parison of eleven methods," *Statistics in Medicine*, 17, 873–890
[13] Agresti, A., and Coull, B. A. (1998), "Approximate is better than "exact" for interval estimation of binomial proportions," *The American Statistician*, 52, 119–126
[14] Pepe MS. The Statistical Evaluation of Medical Tests for Classification and Prediction. Oxford University Press; 2004, chapter 3.
[15] Tsiatis AA. Semiparametric theory and missing data. New York: Springer, 2006, pp.206−207
[16] Joffe, M. M., Ten Have, T. R., Feldman, H. I., & Kimmel, S. E. (2004). Model selection, confounder control, and marginal structural models: Review and new applications. The American Statistician, 58, 272−279

CONFIDENTIALITY
This document is the confidential and proprietary information of GRAIL, LLC. Except as required by federal, state, or local laws or regulations, this document may not be disclosed to others, in whole or in part without prior written permission of GRAIL, Inc.

- no cancer diagnosis has been documented within the first 12 months of follow-up period based on EMR/direct contact
    - Note: participants with MCED-V2 positive test results who are undergoing diagnostic evaluation and did not achieve a diagnostic resolution at 12 months will be considered as having no cancer diagnosis at the time of 12 months for the purposes of evaluating test performance.

Participants with incomplete cancer status assessment at 12 months are defined as those with no cancer diagnosis reported at the time of the last EMR/direct contact and the last EMR/direct contact occurred less than 11 months after the blood draw (e.g. participants withdrew from the study, died, cancer status assessment was conducted prior to 11 months, participants were not reachable after 11 months).

2. Assessment of cancer status for the first 24 months of follow-up is considered to be completed (i.e. no loss to follow-up) when
    - cancer diagnosis is reported at any time during the first 24 months of follow-up, or
    - no cancer diagnosis has been documented within the first 24 months of follow-up period based on EMR/direct contact.

    Participants with incomplete cancer status assessment at 24 months are defined as those with no cancer diagnosis reported at the time of the last EMR/direct contact and the last EMR/direct contact occurred less than 23 months after the blood draw (e.g. participants withdrew from the study, died, cancer status assessment was conducted prior to 23 months, participants were not reachable after 23 months).

3. Assessment of cancer status for the entire 36 months of follow-up (end of study cancer status assessment) is considered to be completed (i.e. no loss to follow-up) when
    - cancer diagnosis is reported at any time during the entire study period, or
    - no cancer diagnosis has been documented during the entire study period based on EMR/direct contact.

    Loss to follow-up is defined as no cancer diagnosis reported at the time of the last EMR/direct contact and the last EMR/direct contact less than 35 months after the blood draw (e.g. participants withdrew from the study, died, cancer status assessment was conducted prior to 35 months, participants were not reachable after 35 months).

Note that if a participant was not reachable during the first 12 months but the site was able to conduct cancer status assessment at 24 months, this participant will be included in the analysis of 24 months follow-up. In addition, such participants will be included in the analysis of the first 12 months follow-up if this analysis is conducted after the 24 months assessment was completed. Similar logic applies for the 36 months follow-up period.

14.1.4 Weighted Analyses for MCED-I

As described in Section 4.2, blood samples from a subset of PATHFINDER 2 participants will be analyzed with MCED-I test as part of the bridging analysis. Participants for the bridging analysis will be selected via stratified random sampling as described in Section 4.2 and Appendix 9.

Estimates of test performance endpoints for MCED-I will be calculated with adjustment for

CONFIDENTIALITY
This document is the confidential and proprietary information of GRAIL, LLC. Except as required by federal, state, or local laws or regulations, this document may not be disclosed to others, in whole or in part without prior written permission of GRAIL, Inc.

sampling weights. The weight will be 1 for participants with a cancer diagnosis or MCED-V2 positive test result because all of these participants will be selected for the bridging analysis. The weights for participants with MCED-V2 negative test results and no cancer diagnosis will be calculated as the reciprocal of the sampling probabilities. Sampling probabilities are calculated as the proportion of participants to be selected out of all participants in the MCED-V2 Analyzable Set eligible for selection within each stratum.

14.1.5 Analysis Time Window for PRO Questionnaires

The analysis of the PRO questionnaires will be based on questionnaires completed ≤ 2 months (61 days) after questionnaire release for pre-test, post-test (following return of MCED test results) and diagnostic resolution timepoints, and ≤ 3 months (91 days) after questionnaire release for the 12 months timepoint. Questionnaires completed outside of these windows are considered to be 'out of window' and not included in the analysis. Questionnaires will be considered to be filled out at a 'Different timepoint' and not included in the analysis if they were completed in the time interval not intended for a given timepoint, for example, if a pre-test questionnaire was filled out after the test result was communicated.

As a sensitivity analysis, a window of ≤ 1 month (30 days) will be applied to the post-test and diagnostic resolution timepoints as those assessments are most likely to be impacted by return of MCED test result and diagnostic resolution, respectively.

14.2 Analysis Methods for Primary Objectives

There are two primary objectives for the PATHFINDER 2 study:

1. Evaluate the safety of the MCED test in terms of diagnostic testing triggered by the MCED test result.

2. Evaluate performance of the MCED Test in individuals eligible for cancer screening.

14.2.1. Primary Objective One (Safety)

14.2.1.1 Safety Endpoints

Safety endpoints during diagnostic testing triggered in participants with a positive MCED-V2 test result include:

- Number and type of invasive procedures (as described in Appendix 10).
- Number and type of adverse events

Number and type of invasive procedures will be assessed in analyzable participants who had:

- Positive MCED-V2 test result communicated
- Evidence that at least one diagnostic evaluation was performed
- Diagnostic evaluation was initiated after communication of MCED-V2 test result
    - If diagnostic testing was initiated prior to the return of the MCED-V2 test results based on signs and symptoms suspicious for cancer, incidental findings or other reasons not related to the MCED-V2 test result, these participants will not be included in the analysis. Their diagnostic testing will be presented in a listing in the

CONFIDENTIALITY
This document is the confidential and proprietary information of GRAIL, LLC. Except as required by federal, state, or local laws or regulations, this document may not be disclosed to others, in whole or in part without prior written permission of GRAIL, Inc.

CSR.

All safety endpoints will be evaluated for each participant from the date of the first diagnostic evaluation performed after the MCED-V2 test result is communicated to the participant and up to the date of diagnostic resolution or the end of the analysis period, whichever comes first.

In addition, safety with respect to the number and type of invasive procedures will be evaluated using the following endpoints[17]:

- Primary endpoint
    - Safety Measure A: Number of participants with a positive MCED test result and an invasive procedure divided by the total number of analyzable participants.
- Secondary endpoints:
    - Safety Measure B: Number of invasive procedures among all participants with a positive MCED test result divided by the total number of analyzable participants.
    - Safety Measure C: Number of participants with a positive MCED test result and an invasive procedure divided by the total number of participants with a positive MCED test result
    - Safety Measure D: Number of invasive procedures among all participants with a positive MCED test result divided by the number of participants with a positive MCED test result

14.2.1.2 Analysis Methods for Safety Analyses of MCED-V2 Test

Invasive Procedures

The number and type of invasive procedures conducted during diagnostic testing triggered by MCED-V2 positive test results in participants with a positive MCED-V2 test result will be summarized descriptively for each of the following categories (see details in Appendix 10):

- All invasive procedures
    - Surgical procedures
    - Non-surgical procedures
        - Endoscopy
        - Biopsy
        - Other non-surgical procedures

The following will be summarized:

- The number of participants with at least one invasive procedure from each of the categories described above will be reported overall and by type
- The total number of invasive procedures from each of the categories will be reported overall and by type

---

[17] Note, that participants who had diagnostic evaluation initiated before MCED test result was communicated and participants with no diagnostic evaluations are excluded from these calculations.

CONFIDENTIALITY
This document is the confidential and proprietary information of GRAIL, LLC. Except as required by federal, state, or local laws or regulations, this document may not be disclosed to others, in whole or in part without prior written permission of GRAIL, Inc.

- The distribution of the per-participant counts for each of the categories will be summarized using descriptive statistics

These analyses will be reported for the following groups of participants overall and by type of invasive procedure as described above:

- Cancer vs no cancer diagnosis
    - Participants with cancer diagnosis (TP)
        - Participants with cancer diagnosis at diagnostic resolution
        - Participants with no cancer diagnosis at diagnostic resolution who have a cancer diagnosis at the end of the analysis period
    - Participants with no cancer diagnosis (FP)
        - Participants with no cancer diagnosis at diagnostic resolution and no cancer diagnosis at the end of the analysis period
        - Participants who did not reach a diagnostic resolution and so do not have a cancer diagnosis at the end of the analysis period

- Initial vs subsequent diagnostic evaluation
    - Participants with an invasive procedure performed as part of their initial diagnostic evaluation: e.g., the first test(s) completed to evaluate a positive MCED-V2 test result.
    - Participants with an invasive procedure performed after initial diagnostic evaluation: e.g., follow up test(s) ordered after review of results from initial diagnostic evaluation.

- Targeted diagnostic evaluation vs evaluation after diagnostic PET-CT (Figure 2)

    - Participants with an invasive procedure performed as part of a targeted diagnostic evaluation: any initial or subsequent testing performed before the diagnostic PET-CT (if any) or prior to diagnostic resolution. Diagnostic PET-CT was referred to as ‘confirmatory PET-CT’ in the study protocol and was intended to be performed only after a negative targeted diagnostic evaluation.

    - If the diagnostic PET-CT after a negative targeted diagnostic evaluation was performed, participants with an invasive procedure performed after the diagnostic PET-CT.

- CSO-guided vs not CSO-guided diagnostic evaluation
    - CSO-guided diagnostic evaluation based on the diagnostic evaluation including imaging tests, laboratory tests, non-invasive procedures and invasive procedures: participants with the diagnostic evaluations consistent with the recommended targeted diagnostic testing for predicted CSO(s) as described in the study protocol (Appendix 11)[18].
    - Not CSO-guided diagnostic evaluation based on the diagnostic evaluation including imaging tests, laboratory tests, non-invasive procedures and invasive procedures:

[18] Evaluation of consistency is based on whether the participant received the expected CSO-guided diagnostic evaluations described in the protocol during their initial diagnostic evaluation.

CONFIDENTIALITY
This document is the confidential and proprietary information of GRAIL, LLC. Except as required by federal, state, or local laws or regulations, this document may not be disclosed to others, in whole or in part without prior written permission of GRAIL, Inc.

participants with diagnostic evaluations inconsistent with the recommended targeted diagnostic testing for predicted CSO(s)

Figure 2. Initial, subsequent and targeted diagnostic evaluations in the presence (panel A) or absence (panel B) of diagnostic PET-CT.

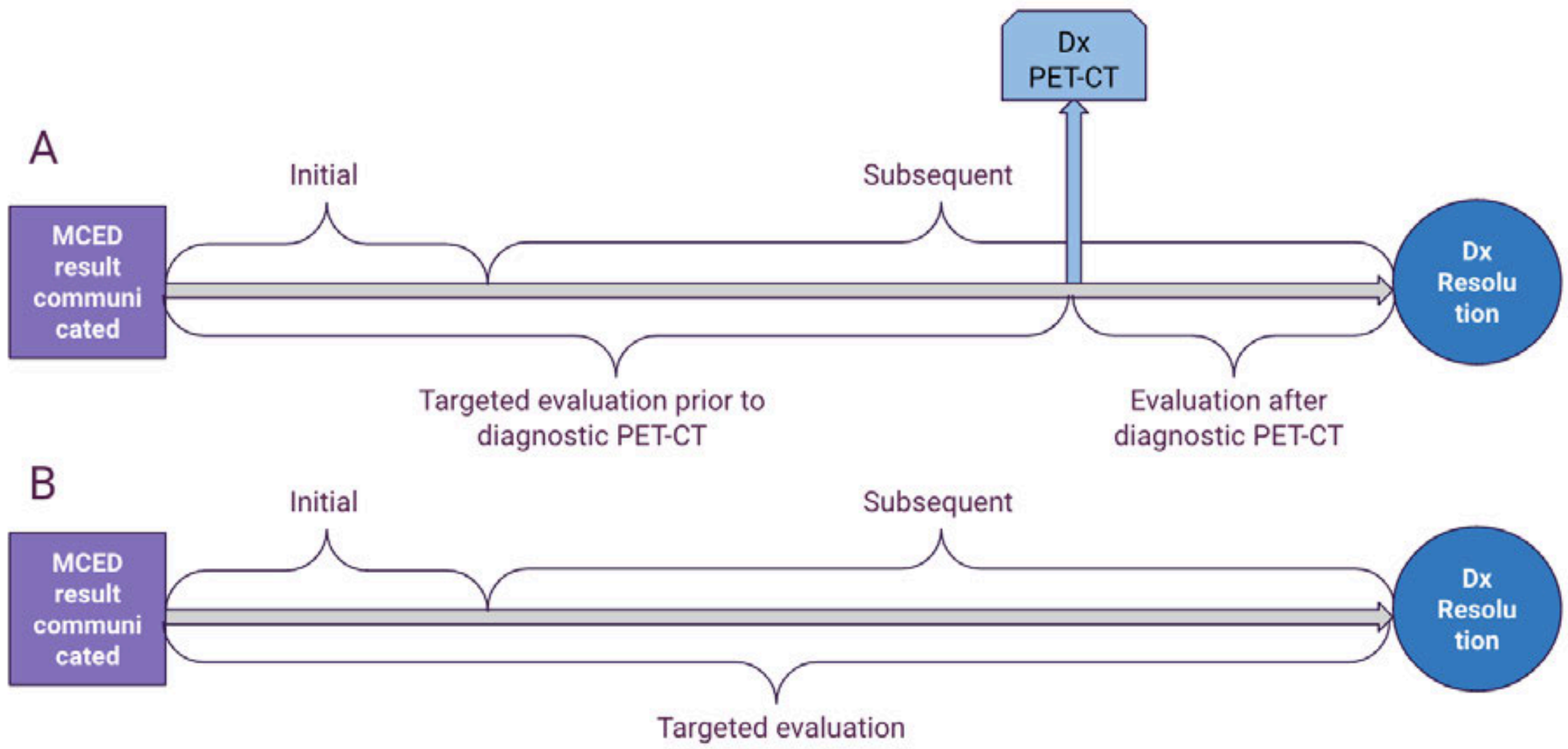

Adverse Events During Diagnostic Testing

The number of adverse events (AE) reported during diagnostic testing triggered by MCED positive test results in participants with a positive MCED-V2 test result will be described. This includes AEs reported from the date of the first diagnostic evaluation performed after the positive MCED-V2 test result is communicated to the participant to the date of diagnostic resolution or the end of the analysis period, whichever comes first (as described in section 14.2.1.1). The AEs will be summarized by the following characteristics:

- Seriousness (whether AE was considered an SAE)
    - If yes, seriousness criteria (death, life threatening, required hospitalization-initial or prolonged, results in persistent or significant disability/incapacity, required intervention to prevent permanent impairment, congenital anomaly/birth defect, other medically important serious event)
- Severity (mild, moderate, severe)
- Relatedness to study activities (i.e., study activities listed in the protocol Schedule of Activities) or device
- Outcome (e.g. recovered/ resolved, recovered/ resolved with sequelae, recovering/ resolving, not recovered/ not resolved, fatal)

AEs will be described for the following subsets:

- Related to study activities, related to device

CONFIDENTIALITY
This document is the confidential and proprietary information of GRAIL, LLC. Except as required by federal, state, or local laws or regulations, this document may not be disclosed to others, in whole or in part without prior written permission of GRAIL, Inc.

- SAEs

AEs will be reported for the following time periods:

- All AEs reported after return of MCED-V2 test result and prior to diagnostic resolution
- AEs reported after diagnostic PET-CT (among participants who had confirmatory PET-CT) and prior to diagnostic resolution

These analyses will be conducted for all participants and for the following groups of participants, separately:

- Participants with a cancer diagnosis at diagnostic resolution
- Participants with no cancer diagnosis at diagnostic resolution
- Participants who did not reach a diagnostic resolution during the analysis period.

A per-participant listing of SAEs in participants with no cancer diagnosis at diagnostic resolution will be provided.

14.2.2. Primary Objective Two (Test Performance)

14.2.2.1 Test Performance Endpoints

Test performance of each MCED test version will be evaluated for:

- Cancer signal detection – based on the comparison of the MCED test result for cancer signal detection (positive or negative) with the target clinical outcome for a participant (presence or absence of cancer diagnosis during a follow-up period)

- CSO prediction – based on the comparison of the CSO prediction with the clinical CSO

- Combined cancer signal detection and CSO prediction

In addition, concordance of the MCED-V2 test and MCED-I test results will be evaluated.

14.2.2.1.1 Test Performance Endpoints for Cancer Signal Detection

Performance of the MCED test will be evaluated using test performance endpoints defined based on a 2x2 contingency table (Table 3) which will be constructed based on the cancer status at a given analysis time point. Participants with incomplete cancer status assessment as defined in section 14.1.3 are not included in this 2x2 table.

Table 3. 2x2 Contingency Table

		**Cancer Status**	
		**Present**	**Absent**
**MCED Test Result for Cancer SIgnal Detection**	**Positive**	TP	FP
	**Negative**	FN	TN

CONFIDENTIALITY
This document is the confidential and proprietary information of GRAIL, LLC. Except as required by federal, state, or local laws or regulations, this document may not be disclosed to others, in whole or in part without prior written permission of GRAIL, Inc.

Where

- TP = True Positives: number of participants with positive MCED test result and a cancer diagnosis
- FP = False Positives: number of participants with positive MCED test result and no cancer diagnosis
- FN = False Negatives: number of participants with negative MCED test result and a cancer diagnosis
- TN = True Negatives: number of participants with negative MCED test result and no cancer diagnosis

The following endpoints will be used to evaluate overall test performance for cancer signal detection:

Positive Predictive Value

Positive Predictive Value (PPV) for cancer signal detection is defined as the proportion of participants with a cancer diagnosis out of all participants with a positive MCED test result. It is calculated as TP / (TP + FP) from the 2x2 contingency table above (Table 3).

Negative Predictive Value

Negative Predictive Value (NPV) for cancer signal detection is defined as the proportion of participants with no cancer diagnosis out of all participants with a negative MCED test result. It is calculated as TN / (TN + FN).

Episode Sensitivity

Episode sensitivity is defined as the ability of a screening test to detect cancers that would be diagnosed during a follow-up period after a specific round of a screening test [19,20,21,22].

Episode Sensitivity for cancer signal detection is estimated as the proportion of participants with a positive MCED test result out of all participants with a cancer diagnosis within the follow-up period. It is calculated as TP / (TP + FN).

Specificity

Specificity for cancer signal detection is defined as the proportion of participants with a negative MCED test result out of all participants with no cancer diagnosis within the follow-up period. Specificity is calculated as TN / (TN + FP). Additionally, false positive rate (FPR), a complement to specificity, will be calculated as FP / (TN + FP).

Positive Likelihood Ratio

The Positive Likelihood Ratio (PLR) for cancer signal detection is defined as the ratio of the probability that a participant with cancer diagnosis has a positive test result vs the probability that a participant with no cancer diagnosis has a positive test result. It is calculated as episode sensitivity

[19] Day NE, Walter SD. Simplified models of screening for chronic disease: estimation procedures from mass screening programmes. Biometrics 1984 40: 1–14.
[20] Cole P, Morrison AS. Basic Issues in Population Screening for Cancer, JNCI 1980 64: 1263–72.
[21] Hakama et al, Sensitivity in cancer screening. J Med Screen 2007:174–177
[22] Sarkeala et al, Episode sensitivity in association with process indicators in the Finnish breast cancer screening program. Int J of Cancer Screening 2006; 118: 174-179

CONFIDENTIALITY
This document is the confidential and proprietary information of GRAIL, LLC. Except as required by federal, state, or local laws or regulations, this document may not be disclosed to others, in whole or in part without prior written permission of GRAIL, Inc.

divided by (1 - specificity), i.e., [TP/ (TP + FN)] / [1 - TN/ (TN + FP)] = TP * (TN + FP) / [FP * (TP + FN)].

Negative Likelihood Ratio

The Negative Likelihood Ratio (NLR) for cancer signal detection is defined as the ratio of the probability that a participant with cancer diagnosis has a negative test result vs the probability that a participant with no cancer diagnosis has a negative test result. It is calculated as (1 - episode sensitivity) divided by specificity, i.e., [1 - TP/ (TP + FN)] / [TN/ (TN + FP)] = FN * (TN + FP) / [TN * (TP + FN)].

MCED Cancer Detection Rate

MCED Cancer Detection Rate (CDR) is defined as the proportion of participants with a positive MCED test result and a cancer diagnosis (true positives) out of all participants in the analyzable set. It is calculated as TP / (TP + FP + FN + TN).

Number Needed to Screen

Number needed to screen is defined as the number of participants that need to be screened with an MCED test to detect a single cancer. It is calculated as 1 / CDR, i.e. (TP + FP + FN + TN) / TP.

In addition, the following measures will be used to evaluate CSO prediction or cancer type specific test performance for cancer signal detection:

PPV for cancer signal detection by CSO prediction

PPV for cancer signal detection by a specific CSO prediction is calculated for each CSO prediction (Appendix 3A for MCED-V2 and Appendix 4A for MCED-I). It is calculated as the proportion of participants with a cancer diagnosis out of all participants with positive MCED test result and a specific CSO1 prediction within the follow-up period.

Episode Sensitivity for cancer signal detection by cancer type

Episode sensitivity for cancer signal detection by a specific cancer type is calculated as the proportion of participants with a positive MCED test result out of all participants with a cancer diagnosis of a specific cancer type within the follow-up period.

Episode Sensitivity for cancer signal detection for prespecified cancer subgroups

Episode sensitivity for cancer signal detection for prespecified cancer subgroups is calculated as the proportion of participants with a positive MCED test result out of all participants with a cancer diagnosis of one of the cancer types in the prespecified cancer subgroup within the follow-up period.

Prespecified cancer subgroups (defined in Appendix 7) include the following:

- Cancers responsible for 2/3 of all cancer deaths in the US
- Most aggressive cancers with lowest 5-year survival in the US
- Cancers without common screening options

CONFIDENTIALITY
This document is the confidential and proprietary information of GRAIL, LLC. Except as required by federal, state, or local laws or regulations, this document may not be disclosed to others, in whole or in part without prior written permission of GRAIL, Inc.

- Solid cancers with common screening options
- Solid cancers without common screening options
- Hematologic malignancies

14.2.2.1.2 Test Performance Endpoints for CSO Prediction

Test performance endpoints for CSO prediction are focused on participants with a positive MCED test result and a cancer diagnosis at a given time point, i.e., TP.

Accuracy of CSO prediction is defined as the proportion of participants with a correct CSO prediction (i.e. CSO prediction matching clinical CSO) among TP participants, including cases with "Cancer type without clinical CSO" and "Unassigned clinical CSO".

Specifically, the following CSO accuracy calculations are used in this study:

- **Primary endpoint: accuracy of CSO prediction in true positives** is defined as the proportion of correct CSO1 predictions among TP participants.
    - For participants with multiple cancers, if CSO1 prediction matches one of the clinical CSOs, then it is considered a correct prediction and this participant with multiple cancers will be included in the numerator.
- **Secondary endpoint: accuracy of CSO1/2 prediction in true positives** is defined as the proportion of correct CSO1 or CSO2 predictions among TP participants. In this calculation, if any of the CSO1 or CSO2 predictions match the clinical CSO, then it is considered a correct prediction. This measure will only be calculated for the MCED-V2 test.
    - For participants with multiple cancers, if CSO1 or CSO2 predictions match one of the clinical CSOs, then it is considered a correct prediction and this participant with multiple cancers will be included in the numerator.
- **Tertiary endpoint: accuracy of CSO2 prediction when CSO1 is inaccurate in true positives with 2 CSOs returned** is defined as the proportion of correct CSO2 predictions among TP participants with two CSOs returned and inaccurate CSO1. This measure will only be calculated for the MCED-V2 test.
    - For participants with multiple cancers, if CSO2 prediction matches one of the clinical CSOs, then it is considered a correct prediction and this participant with multiple cancers will be included in the numerator.

CSO Confusion Matrix

A confusion matrix comparing the CSO1 prediction (rows) with the clinical CSOs (columns) will be constructed for TP participants (Table 4).

Participants diagnosed with a cancer type that does not have a corresponding clinical CSO or a cancer with an unassigned clinical CSO will not be included in the confusion matrix. For TP participants with multiple cancers, if at least one of the cancers has an assigned clinical CSO, those participants will be included in the confusion matrix. If one of the clinical CSOs is matched with the CSO prediction, then this participant will be counted under that clinical CSO diagonal entry. If none of the clinical CSOs is matched with the CSO1 prediction, then this participant will be counted under the clinical CSO linked to the first cancer diagnosed during the follow-up period.

CONFIDENTIALITY
This document is the confidential and proprietary information of GRAIL, LLC. Except as required by federal, state, or local laws or regulations, this document may not be disclosed to others, in whole or in part without prior written permission of GRAIL, Inc.

Table 4. Sample Confusion Matrix

CSO1 for a given MCED test	Clinical CSO 1	Clinical CSO 2	Clinical CSO 3	…	Clinical CSO_n	Total	CSO-Specific Precision
**CSO Prediction 1**	a_{11}	a_{12}	a_{13}	…	a_{1n}	X1	a_{11} / X1
**CSO Prediction 2**	a_{21}	a_{22}	a_{23}	…	a_{2n}	X2	a_{22} / X2
**CSO Prediction 3**	a_{31}	a_{32}	a_{33}	…	a_{3n}	X3	a_{33} / X3
**…**	…	…	…	…	…	…	…
**CSO Prediction n**	a_{n1}	a_{n2}	a_{n3}	…	a_{nn}	Xn	a_{nn} / Xn
**Total**	Y1	Y2	Y3	…	Yn		
**CSO-Specific Accuracy**	a_{11} / Y1	a_{22} / Y2	a_{33} / Y3	…	a_{nn} / Yn		

The following endpoints are calculated from the confusion matrix:

- **Accuracy of a CSO prediction in true positives by clinical CSO** is defined as the proportion of participants with a correct CSO prediction out of all the TP participants who have been diagnosed with a cancer corresponding to that clinical CSO. It is estimated by the proportion of diagonal cells in each column of the confusion matrix.
- **Precision of a CSO prediction in true positives by CSO prediction** is defined as the proportion of participants whose CSO prediction matches their clinical CSO out of all TP participants with a given CSO prediction. It is estimated as the proportion of diagonal cells in each row of the confusion matrix.

In addition to the overall confusion matrix constructed for all TP participants, two confusion matrices will be constructed for female and male TP participants, separately.

14.2.2.1.3 Test Performance Endpoints for MCED-I CSO or CBS Prediction

The following endpoint is defined to evaluate accuracy of CSO or CBS prediction:

Accuracy of CSO or CBS prediction in true positives is defined as the proportion of TP participants with a correct CSO1 or CBS prediction among TP participants. In this calculation, if either the CSO1 prediction matches the clinical CSO or the CBS prediction matches the clinical CBS, then it is considered a correct prediction. For participants with multiple cancers, if the CSO1 prediction matches any one of the clinical CSOs, or the CBS prediction matches any one of the clinical CBS, then it is considered a correct prediction and this participant with multiple cancers will be included in the numerator.

A listing of all participants with CBS predictions will be provided. CBS predictions and clinical CBS

CONFIDENTIALITY
This document is the confidential and proprietary information of GRAIL, LLC. Except as required by federal, state, or local laws or regulations, this document may not be disclosed to others, in whole or in part without prior written permission of GRAIL, Inc.

will be cross-tabulated.

In addition, an exploratory endpoint for CBS accuracy will be calculated among the subset of TP participants who have both a CBS prediction and a clinical CBS available.

Exploratory endpoint: Accuracy of CBS prediction in true positives is defined as the proportion of TP participants with a CBS prediction that matches their clinical CBS among TP participants with clinical CBS and CBS prediction available. For participants with multiple cancers, if the CBS prediction matches any one of the clinical CBS, then it is considered a correct prediction and this participant with multiple cancers will be included in the numerator.

14.2.2.1.4 Test Performance Endpoints for Combined Cancer Signal Detection and CSO Prediction or CBS Prediction

Test performance endpoints for combined cancer signal detection and CSO prediction are based on Table 5 and include performance endpoints described below. In this table NCCSO reflects "Cancer type without clinical CSO" and "Unassigned clinical CSO".

Table 5. CSO prediction by clinical CSO.

CSO1 for a given MCED test	Clinical CSO 1	..	Clinical CSO n	Cancers with no clinical CSO (NCCSO)	No cancer diagnosis	Total
**CSO Prediction 1**	$N_{1,1}$	...	$N_{1,n}$	$NCCSO_1$	NC_1	N_1
**..**	…	…	…	…	…	…
**CSO Prediction n**	$N_{n,1}$	...	$N_{n,n}$	$NCCSO_n$	NC_n	N_n
**TOTAL**	C_1	..	C_n	NCCSO	NC	N

PPV accounting for CSO accuracy

PPV accounting for CSO accuracy is calculated as the proportion of participants whose CSO prediction matches their clinical CSO out of all participants with positive MCED test results. This is defined as $\sum N_{J,J}$ / N using nomenclature of Table 5.

PPV accounting for CSO accuracy by CSO prediction

PPV accounting for CSO accuracy by CSO prediction is calculated as the proportion of participants whose CSO prediction matches their clinical CSO out of all participants with positive MCED test results with a specific CSO1 prediction. This is defined as $N_{J,J}$ / N_J using nomenclature of Table 5.

CONFIDENTIALITY
This document is the confidential and proprietary information of GRAIL, LLC. Except as required by federal, state, or local laws or regulations, this document may not be disclosed to others, in whole or in part without prior written permission of GRAIL, Inc.

Episode Sensitivity accounting for CSO accuracy

Episode sensitivity accounting for CSO1 accuracy is calculated as the proportion of participants whose CSO1 prediction matches their clinical CSO out of all participants with a cancer diagnosis within the follow-up period. This is defined as $\sum N_{J,J}$ / ($\sum C_J$ + NCCSO + FN) using nomenclature of Table 5 and Table 3.

Episode sensitivity accounting for CSO1 or CSO2 accuracy is calculated as the proportion of participants whose CSO1 or CSO2 prediction matches their clinical CSO out of all participants with a cancer diagnosis within the follow-up period. This measure will only be calculated for the MCED-V2 test.

Episode Sensitivity accounting for CSO accuracy by clinical CSO

Episode sensitivity accounting for CSO accuracy by clinical CSO is calculated as the proportion of participants whose CSO prediction matches their clinical CSO out of all participants with a cancer diagnosis and specific clinical CSO assigned. This is defined as $N_{J,J}$ / ($C_J + FN_J$) using nomenclature of Table 5, where FN_J is the number of cancers with clinical CSO_J and MCED negative test result.

Episode Sensitivity accounting for CSO or CBS accuracy

Episode sensitivity accounting for CSO or CBS accuracy is calculated as the proportion of participants whose CSO1 prediction matches their clinical CSO or CBS prediction matches their clinical CBS; out of all participants with a cancer diagnosis within the follow-up period. This measure will only be calculated for the MCED-I test.

14.2.2.1.5 Analyses by Stage

Distribution of stage for new cancers and extent of recurrence for recurrent cancers will be summarized for all cancers with:

- MCED positive test results
- MCED negative test results

Stage-specific estimates of episode sensitivity (as defined in Section 14.2.2.1.1) may be biased because cancer status is not ascertained for all study participants at the time of blood draw. This phenomenon has been reported in a recent simulation study[23]. Specifically, while the stage for an MCED test detected cancer would be determined close to the time of the blood draw, the stage for a cancer that is not detected by the MCED test would be determined at the time of clinical presentation, which may occur much later than the time of blood draw. Therefore clinically presenting cancers are more likely to be diagnosed at a more advanced stage than the screen-detected cancers, which is expected to cause differential bias when describing episode

[23] Pinsky P, Lange J, Etzioni R, Estimating stage-specific sensitivity for cancer screening tests, Journal of Medical Screening, 2023

CONFIDENTIALITY
This document is the confidential and proprietary information of GRAIL, LLC. Except as required by federal, state, or local laws or regulations, this document may not be disclosed to others, in whole or in part without prior written permission of GRAIL, Inc.

sensitivity by stage.

14.2.2.1.6 Concordance of MCED-V2 Test and MCED-I Test Results

For participants in both the MCED-V2 Analyzable Set and MCED-I Analyzable Set, a 2x2 matrix of MCED-V2 and MCED-I test results will be constructed separately for participants with cancer diagnosis, participants without cancer diagnosis, and all participants combined (see Table 6).

Table 6. Concordance of MCED-V2 and MCED-I test results for cancer signal detection

Category	MCED-I positive	MCED-I negative
MCED-V2 positive	A	B
MCED-V2 negative	C	D

Percent agreement between the two test versions and corresponding confidence intervals will be calculated:

- Positive Percent Agreement (PPA) = 100% * A / (A+B)
- Negative Percent Agreement (NPA) = 100% * D / (C+D)
- Overall Percent Agreement (OPA) = 100% * (A+D) / (A+B+C+D)

In addition, a descriptive summary of the concordance between MCED-V2 CSO predictions and MCED-I CSO predictions in participants with positive test results for both test versions will be provided. It will be presented as a matrix (Appendix 5B) and the proportion of participants with the same MCED-V2 CSO1 prediction and MCED-I CSO1 prediction will be reported in participants with cancer diagnosis, participants without cancer diagnosis, and all participants combined.

14.2.2.1.7 Descriptive Summaries

Participants with TP, FP, FN and TN results at each time point will be described as follows:

- TP: distribution of
    - CSO predictions
    - CBS predictions
    - Cancer types
    - Number (proportion) of cancers in each of the prespecified cancer groups (as defined in Appendix 7)
    - Stage for new cancers (stage I, II, III, IV, I-II, I-III)
    - Extent of recurrence for recurrent cancers
    - Multiple cancers
    - Pathology-confirmed cancers by cancer type
    - Treatment
        - Number of participants who had a treatment planned for their cancer
        - Type of treatment among participants with treatment planned

CONFIDENTIALITY
This document is the confidential and proprietary information of GRAIL, LLC. Except as required by federal, state, or local laws or regulations, this document may not be disclosed to others, in whole or in part without prior written permission of GRAIL, Inc.

(Chemotherapy, Hormone therapy, Immunotherapy, Radiation therapy, Targeted therapy, Stem cell or bone marrow transplant, Surgery, Other)
        - Intent of treatment among participants with treatment planned (Curative, Palliative, Surveillance, Other, Unknown)
- FP: distribution of
    - CSO predictions
    - CBS predictions
    - Non-cancer conditions identified during diagnostic evaluation
        - Benign neoplasms (benign solid neoplasm, lymphoproliferative disorder, monoclonal gammopathy of undetermined significance, other)
        - Benign non-neoplastic conditions (Autoimmune disease, Chronic viral infection, Esophageal disease (e.g., Barrett's esophagus, GERD), Gastritis or gastric (peptic) ulcers, Inflammatory bowel disease (i.e., Crohn's disease or ulcerative colitis), Liver disease (e.g., cirrhosis or fatty liver disease), Lung disease (e.g., pulmonary fibrosis, emphysema, chronic bronchitis), Pancreatic disease (e.g., pancreatitis), Other
- FN: distribution of
    - Cancer types
    - Number (proportion) of cancers in each of the prespecified cancer groups (as defined in Appendix 7)
    - Stage for new cancers (stage I, II, III, IV, I-II, I-III)
    - Extent of recurrence for recurrent cancers
    - Multiple cancers
    - Pathology-confirmed cancers by cancer type
    - Treatment
        - Number of participants who had a treatment planned for their cancer
        - Type of treatment among participants with treatment planned (Chemotherapy, Hormone therapy, Immunotherapy, Radiation therapy, Targeted therapy, Stem cell or bone marrow transplant, Surgery, Other)
        - Intent of treatment among participants with treatment planned (Curative, Palliative, Surveillance, Other, Unknown)
    - Method of cancer diagnosis (Follow-up surveillance of prior imaging test finding, Incidental finding, GRAIL MCED test taken as part of study, GRAIL MCED test taken outside of study, Patient reported signs or symptoms, Post treatment surveillance for recurrence, Cancer screening test, Other)
- TP, FP, TN, FN: distribution of
    - Age: 50-59, 60-69,70-79, 80+ years
    - Biological sex
    - Race categories aligned with FDA guidance (as described in Section 13.3)
    - Ethnicity
    - BMI categories (<25 kg/m^2, 25 to <30 kg/m^2, ≥ 30 kg/m^2)
    - Smoking status (never smoker, former smoker, current smoker)
    - USPSTF lung cancer risk indicator (as described in Section 13.3)
    - Prior history of cancer (Yes, No)
    - Presence of genetic cancer predisposition (Yes, No)

CONFIDENTIALITY
This document is the confidential and proprietary information of GRAIL, LLC. Except as required by federal, state, or local laws or regulations, this document may not be disclosed to others, in whole or in part without prior written permission of GRAIL, Inc.

We will also describe participants with positive MCED test results, no cancer diagnosis at time of diagnostic resolution and cancer diagnosis at 12, 24 or 36 months in terms of:

- CSO predictIons
- CBS predictions
- Cancer type
- Number (proportion) of cancers in each of the prespecified cancer groups (as defined in Appendix 7)
- Stage for new cancers (stage I, II, III, IV, I-II, I-III)
- Extent of recurrence for recurrent cancers
- Multiple cancers
- Pathology-confirmed cancers by cancer type
- Method of cancer diagnosis
- Age: 50-59, 60-69,70-79, 80+ years
- Biological sex
- Race categories aligned with FDA guidance (as described in Section 13.3)
- Ethnicity
- BMI categories (<25 kg/m^2, 25 to <30 kg/m^2, ≥30 kg/m^2)
- Smoking status (never smoker, former smoker, current smoker)
- USPSTF lung cancer risk indicator (as described in Section 13.3)
- Prior history of cancer (Yes, No)
- Presence of genetic cancer predisposition (Yes, No)

14.2.2.2 Study Success Criteria for PMA Analysis

Study success criteria

As part of the primary objective, the study success criteria for PMA analysis will be evaluated for the MCED-I test based on the 12 months follow-up time analysis period using hierarchical testing.

- First, two co-primary endpoints of episode sensitivity and specificity will be tested to demonstrate that episode sensitivity for all cancers is significantly higher than 10% and that specificity is significantly higher than 99%. These targets represent what the lower bound of the two-sided 95% confidence interval for each endpoint should exceed.

  Hypothesis testing for the study success criteria co-primary endpoints:

  1. H_0: Episode sensitivity for all cancers ≤ 10% vs

     H_A: Episode sensitivity for all cancers > 10%

  2. H_0: Specificity ≤ 99% vs

     H_A: Specificity > 99%

  To claim success on the primary study success criteria, statistical significance needs to be achieved for both of the co-primary endpoints (with lower bound of the two-sided 95%

CONFIDENTIALITY
This document is the confidential and proprietary information of GRAIL, LLC. Except as required by federal, state, or local laws or regulations, this document may not be disclosed to others, in whole or in part without prior written permission of GRAIL, Inc.

confidence interval for episode sensitivity >10% and lower bound of the two-sided 95% confidence interval for specificity >99%).

- Subsequently, conditional on the success of the co-primary endpoints, accuracy of CSO prediction in true positives will be tested to demonstrate that it is significantly higher than 70% (with lower bound of the two-sided 95% confidence interval > 70%).

Hypothesis testing for the study success criteria secondary endpoint:

H_O: accuracy of CSO prediction in true positives ≤ 70% vs

H_A: accuracy of CSO prediction in true positives > 70%

Key cancer and CSO-specific performance endpoints as a descriptive summary

In addition, a set of clinically relevant cancer and CSO-specific performance endpoints will be provided descriptively for comprehensive evaluation of MCED-I test performance. No study success criteria will be set for these endpoints.

- Episode Sensitivity for cancer signal detection for additional prespecified cancer subgroups (as defined in Appendix 7)
    - Cancers responsible for ⅔ of all cancer deaths in the US
    - Most aggressive cancers with lowest 5-year survival
    - Cancers without common screening options
    - Solid cancers with common screening options
    - Solid cancers without common screening options
    - Hematologic malignancies
- Episode Sensitivity for cancer signal detection by cancer type
- Episode Sensitivity accounting for CSO accuracy by clinical CSO
- Accuracy of CSO prediction in true positives by clinical CSO
- Precision of CSO prediction in true positives by CSO prediction
- PPV for cancer signal detection by CSO prediction
- PPV accounting for CSO accuracy by CSO prediction

14.2.2.3 Evaluation of MCED-V2 Test Performance

MCED-V2 test performance will be evaluated at several time points (see Section 14.1.3 for details on annual cancer status assessments):

- Time of diagnostic resolution - secondary time point
- 12 months after the date of the first blood draw[24] - primary time point
- 24 months after the date of the first blood draw - secondary time point

[24] The blood draw date will be the first blood draw date if multiple blood draws have been performed

CONFIDENTIALITY
This document is the confidential and proprietary information of GRAIL, LLC. Except as required by federal, state, or local laws or regulations, this document may not be disclosed to others, in whole or in part without prior written permission of GRAIL, Inc.

14.2.2.3.1 Analysis at the time of diagnostic resolution time point

Note that at the time of diagnostic resolution only performance endpoints based on positive MCED-V2 test results can be evaluated because there is no corresponding cancer status evaluation in MCED-V2-negative participants (endpoints defined as described in Section 14.2.2.1):

- PPV for cancer signal detection
- PPV for cancer signal detection by CSO prediction
- PPV accounting for CSO accuracy by CSO prediction
- Accuracy of CSO prediction in true positives
- Accuracy of a CSO prediction in true positives by clinical CSO
- Precision of a CSO prediction in true positives by CSO prediction

PPV endpoints will be evaluated in MCED-V2 positive participants who achieved diagnostic resolution.

14.2.2.3.2 Analysis at 12 months follow-up time point

Test performance endpoints described in Section 14.2.2.1 (except for section 14.2.2.1.3) will be evaluated based on the 12 months follow-up time period.

MCED Cancer Detection in Addition to Existing Cancer Screening Tests

To illustrate the benefit of MCED when used in addition to existing standard of care (SOC) cancer screening tests for cancer detection, the number of participants with cancers detected during the 12 months follow-up period will be reported for the following groups:

- Group 1: Participants with MCED test positive results and cancer diagnosis
- Group 2: Participants with MCED test negative results whose cancer diagnosis was triggered by an existing screening test with grade A or B recommendation by USPSTF: breast, cervix, colorectal, and lung cancers
- Group 3: Participants with MCED test negative results whose cancer diagnosis was triggered by other cancer screening tests
    - Screening tests with grade C recommendation by USPSTF: prostate cancer
    - Screening tests without grade A, B or C USPSTF recommendations such as for thyroid or melanoma cancers
- Group 4: Participants with MCED test negative results whose cancer diagnosis was not triggered by cancer screening (e.g., clinically detected cancers)

Note, participants with multiple cancers will be included in the group which appears earlier in the list above so participant groups are mutually exclusive. For example, a participant with MCED negative test result, a colorectal cancer diagnosis triggered by a FIT test and a clinically detected liver cancer will be included in Group 2.

Distribution of cancer type and stage will be summarized for each group.

CONFIDENTIALITY
This document is the confidential and proprietary information of GRAIL, LLC. Except as required by federal, state, or local laws or regulations, this document may not be disclosed to others, in whole or in part without prior written permission of GRAIL, Inc.

14.2.2.3.3 Analysis at 24 months time point

Analyses described in Section 14.2.2.3.2 will be evaluated based on the 24 months follow-up.

14.2.2.4 Evaluation of MCED-I Test Performance

Evaluation of MCED-I test performance at the time of diagnostic resolution is not possible because cancer status for MCED-V2-negative & MCED-I-positive cases may not be directly assessed via diagnostic workup. Therefore, MCED-I test performance will be evaluated at 12 months and 24 months time points.

14.2.2.4.1 Analysis at 12 months follow-up time point

All performance endpoints described in Section 14.2.2.1 will be evaluated, including test performance endpoints for cancer signal detection, CSO prediction, combined cancer signal detection and CSO prediction, stage distribution, concordance of MCED-V2 test and MCED-I test results. All analyses for MCED-I will be adjusted for sampling weights as described in Section 14.1.4.

Note that MCED-I test performance estimates are expected to be conservative because MCED-I positive cases that are MCED-V2 negative will not have a diagnostic workup for cancer at the time of the PMA analysis and therefore their cancer status is based on cancer status assessment at 12 months of follow-up time. Such cases therefore will be treated as FP in the PMA analysis if participants are not diagnosed with cancer within the 12 month follow-up period. If such participants have a cancer diagnosis during year 2 follow-up, they will be treated as TP in the additional post-PMA analyses.

Analyses described in Section 14.2.2.3.2 will be carried out.

14.2.2.4.2 Analysis at 24 months time point

All performance endpoints described in Section 14.2.2.1 will be evaluated based on the 24 months follow-up.

14.3 Analysis Methods for Secondary Objectives

The following analyses will be performed to achieve the secondary objectives of this study.

14.3.1 Participant reported outcomes and behavioral intentions

14.3.1.1 Evaluate intention and utilization of guideline recommended cancer screening procedures after use of the MCED test

CONFIDENTIALITY

This document is the confidential and proprietary information of GRAIL, LLC. Except as required by federal, state, or local laws or regulations, this document may not be disclosed to others, in whole or in part without prior written permission of GRAIL, Inc.

Intention to follow cancer screening recommendations

Intention to follow cancer screening recommendations will be assessed using participant-reported questionnaire pre-test, post-test and at 12 months (+30 days) in all participants. Responses to the question "How likely are you to follow your healthcare provider's cancer screening recommendations?" were represented by a 4-point Likert-type scale (i.e., Very Unlikely, Unlikely, Likely, and Very Likely).

Number and proportion of participants with each rating will be reported at each time point for the following analysis sets:

- MCED-V2 No Cancer Signal Detected
  - No Cancer Signal Detected and cancer diagnosis (i.e. FN)
  - No Cancer Signal Detected and no cancer diagnosis (i.e. TN)
- MCED-V2 Cancer Signal Detected
  - Cancer Signal Detected and cancer diagnosis (i.e. TP)
  - Cancer Signal Detected and no cancer diagnosis (i.e. FP)

Individual item responses for baseline vs post-baseline assessment will be cross-tabulated. Change from baseline (pre-test) to post-baseline assessments at each time point will be illustrated for each analysis set by:

- Proportion of participants who changed their rating from Likely or Very Likely at baseline to Unlikely or Very Unlikely post-baseline

- Proportion of participants who changed their rating from Unlikely or Very Unlikely at baseline to Likely or Very Likely post-baseline

Utilization of guideline recommended cancer screening

The utilization of guideline recommended standard of care (SOC) cancer screening tests will be assessed in the period of time prior to study enrollment (5 years for all screening modalities and at any time for colonoscopies), during the first 12 months, 24 months and 36 months after the MCED test is assessed. The analysis will focus on the cancer screening tests with grade A or B recommendations from USPSTF (Appendix 12). The number of men who have ever received screening for prostate cancer will be reported. However, prostate cancer screening will not be included in the analyses described below because the USPSTF makes no recommendation for or against routine provision of this screening test (grade C recommendation), and the recommendation for periodic prostate-specific antigen (PSA)-based screening is intended to be made on an individual basis after discussion of benefits and harms with clinician.

Colorectal cancer screening for participants aged 76 years and older will also not be included in these analyses because the USPSTF makes no recommendation for or against routine provision of this screening test (grade C recommendation), and suggest that decision to screen should be made on an individual basis in consideration of health status.

Based on the information about cancer screening for a given cancer <u>prior to study enrollment</u>,

CONFIDENTIALITY
This document is the confidential and proprietary information of GRAIL, LLC. Except as required by federal, state, or local laws or regulations, this document may not be disclosed to others, in whole or in part without prior written permission of GRAIL, Inc.

participants eligible for screening per USPSTF grade A or B recommendations with be characterized at the time of enrollment as:

- Up to date with their recommended cancer screening (per USPSTF screening recommendations in Appendix 12). “Up to date” is defined as being within a recommended cancer screening interval + 90 days from the date of the last reported cancer screening.
- Not up to date with their recommended cancer screening
- Number of participants with at least one cancer screening test prior to enrollment will be reported overall and by type of test.

The definition of being up to date with recommended SOC cancer screening tests will be also applied throughout the analysis period. The following information will be reported for cancers with USPSTF Grade A or B recommendations by cancer type and overall:

- Number of cancers diagnosed in all study participants
    - Number of interval cancers[25] (cancers diagnosed while a participant is considered to be up to date with their recommended SOC cancer screening test for a given cancer during the analysis period)
    - Number of cancers diagnosed in participants who are not considered to be up to date with their recommended SOC cancer screening test for a given cancer during the analysis period
- Number of cancers diagnosed in participants with MCED-V2 positive test results
    - Number of interval cancers (cancers diagnosed while a participant is considered to be up to date with their recommended SOC cancer screening test for a given cancer during the analysis period)
- Number of cancers diagnosed in participants who are not considered to be up to date with their recommended SOC cancer screening test for a given cancer during the analysis period
- Number of cancers diagnosed in participants with MCED-I positive test results
    - Number of interval cancers (cancers diagnosed while a participant is considered to be up to date with their recommended SOC cancer screening test for a given cancer during the analysis period)
    - Number of cancers diagnosed in participants who are not considered to be up to date with their recommended SOC cancer screening test for a given cancer during the analysis period

Based on utilization of cancer screening for a given cancer <u>during the analysis period after MCED-V2 testing,</u> participants eligible for screening at enrollment will be characterized as:

- Due for cancer screening during the analysis period
- Due for cancer screening during the analysis period and had at least one cancer screening test during the analysis period

---

[25] Farber, Rachel, Nehmat Houssami, Isabelle Barnes, Kevin McGeechan, Alexandra Barratt, and Katy J. L. Bell. 2022. "Considerations for Evaluating the Introduction of New Cancer Screening Technology: Use of Interval Cancers to Assess Potential Benefits and Harms" International Journal of Environmental Research and Public Health 19, no. 22: 14647. https://doi.org/10.3390/ijerph192214647

CONFIDENTIALITY

This document is the confidential and proprietary information of GRAIL, LLC. Except as required by federal, state, or local laws or regulations, this document may not be disclosed to others, in whole or in part without prior written permission of GRAIL, Inc.

- Up to date with their recommended cancer screening at the end of the analysis period
- Not up to date with their recommended cancer screening at the end of the analysis period

The utilization of cancer screening during the analysis period after MCED-V2 testing will be assessed for:

- All analyzable participants
- Participants who were up to date with their recommended screening at the time of enrollment
- Participants who were not up to date with their recommended screening at the time of enrollment

The utilization of cancer screening prior to enrollment and during the analysis period after MCED testing will be assessed in participants with MCED-V2 test negative results (the main focus of this analysis), those with MCED-V2 test positive results and in all participants.

In addition, the utilization of cancer screening tests during the analysis period after MCED testing will be compared to utilization prior to enrollment descriptively.

Cancer screening rates declined sharply in March through May of 2020, during the first peak of COVID-19 incidence, followed by a "return to normal or near-normal levels" in the summer of that year[26,27]. A subsequent surge in COVID-19 incidence in Jan-Mar 2022 seems to have had a low impact on the cancer screening rates[21]. PATHFINDER 2 study enrollment started in December 2021, which is more than 18 months after the start of the COVID-19 pandemic, so we expect the initial rise in COVID-19 incidence to have limited, if any, impact on our evaluation of cancer screening utilization prior to enrollment. As an exploratory analysis, we will examine the patterns of cancer screening utilization over time before and after the start of the COVID-19 pandemic where sufficient data are available.

14.3.1.2 Assess Participants Reported Anxiety Resulting from Use of the MCED Test

Participant reported anxiety is assessed using STAI (6 items) and MCED STAI (3 items) questionnaires in all participants at baseline (pre-test), post-test and at 12 months. In addition, MCED test positives will be assessed at diagnostic resolution (Appendix 2).

For the STAI (6 item) questionnaire, descriptive statistics will be provided for individual item responses on a 4-point scale (i.e., Not at all, Somewhat, Moderately, Very much so) and a total score calculated according to the STAI manual[28] at each time point. In addition, change in the total score from baseline at post-test, diagnostic resolution and 12 months will be assessed. A two-sided t-test will be used to determine if mean changes from baseline are significantly different from zero at each post-baseline assessment at 0.05 significance level. If differences in scores do

---

[26] Doan C et al, Breast and Lung Cancer Screening Among Medicare Enrollees During the COVID-19 Pandemic, JAMA Netw Open. 2023 Feb; 6(2)
[27] Chen RC et al, Association of Cancer Screening Deficit in the United States With the COVID-19 Pandemic, *JAMA Oncol.* 2021;7(6):878-884
[28] Spielberger C.D., 1983. Manual for the State-Trait Anxiety Inventory STAI (Form Y). Palo Alto, CA: Consulting Psychologists Press

CONFIDENTIALITY
This document is the confidential and proprietary information of GRAIL, LLC. Except as required by federal, state, or local laws or regulations, this document may not be disclosed to others, in whole or in part without prior written permission of GRAIL, Inc.

not follow a normal distribution, Wilcoxon signed rank test[29] will be used instead. Hochberg method[30] will be used to adjust for multiple testing and adjusted p-values reported.

In the STAI user manual, mean scores were 32-34 (SD=8-10) for men and women aged 50-69 years, 42.38 (SD=13.79) for patients undergoing general, medical and surgical procedures and 49.02 (SD:11.67) for those with anxiety disorders. STAI scores are commonly classified as “no or low anxiety” (20–37), “moderate anxiety” (38–44) , and “high anxiety” (45–80)[31]. Distribution of these anxiety categories will be summarized at each timepoint. Change from baseline in the proportion of participants with ‘high anxiety’ will be assessed at each timepoint. Descriptive statistics will be provided for STAI standardized total scores for each timepoint.

In addition, distribution of the individual responses to item 6 (“I am worried”) will be summarized at each timepoint. Individual item responses for baseline vs post-baseline assessment will be cross-tabulated as a 4 x 4 matrix. Change from baseline in the proportion of participants with “Moderately” or “Very much so” responses assessed at each timepoint.

For MCED STAI (3 item) questionnaire, descriptive statistics will be provided for individual item responses at each timepoint. Individual item responses for baseline vs post-baseline assessment will be cross-tabulated. Change from baseline in the proportion of participants with “Moderately” or “Very much so” responses to the question “I am worried because of my multi-cancer early detection test“ will be assessed at each timepoint.
The results will be summarized for:

- All MCED-V2 analyzable participants
- Participants with MCED-V2 test positive and negative results, separately
- Participants with MCED-V2 test positive results with and without cancer diagnosis at diagnostic resolution, separately.

The number of participants who responded to at least one question for each questionnaire at each time point will be reported.

14.3.1.3 Assess participants’ reported outcomes and perceptions about the MCED test

Analysis methods

Participants’ reported outcomes and perceptions will be assessed using participant-directed questionnaires evaluated at different time points during the study (see Appendix 2). Description of each questionnaire and scoring mechanism is provided in Appendix 13. Missing responses will be handled according to the corresponding scoring manual for each questionnaire.

The results will be reported for all participants with at least one analyzable score for a given questionnaire for the following participant groups:

- MCED-V2 Analyzable Set

---

[29] Wilcoxon, F (1945) Individual comparisons by ranking methods. Biometrics 1:80–83
[30] Hochberg Y. A sharper Bonferroni procedure for multiple tests of significance. Biometrika 1988;75:800-2.
[31] Millar, K., Jelilic, M., et al. 1995. Assessment of preoperative anxiety: comparison of measures in patients awaiting surgery for breast cancer. British Journal of Anaesthesia

CONFIDENTIALITY
This document is the confidential and proprietary information of GRAIL, LLC. Except as required by federal, state, or local laws or regulations, this document may not be disclosed to others, in whole or in part without prior written permission of GRAIL, Inc.

- MCED-V2 Cancer Signal Detected Set
  - MCED-V2 Cancer Signal Detected and cancer diagnosis within 12 months
  - MCED-V2 Cancer Signal Detected and no cancer diagnosis within 12 months
- MCED-V2 No Cancer Signal Detected Set
  - MCED-V2 No Cancer Signal Detected and cancer diagnosis within 12 months
  - MCED-V2 No Cancer Signal Detected and no cancer diagnosis within 12 months

The number of participants who responded to at least one question for each questionnaire at each time point will be reported.

For health-related quality of life (SF-12v2 questionnaire), a two-sided t-test will be used to determine if mean changes from baseline are significantly different from zero at each post-baseline assessment at 0.05 significance level. If differences in scores do not follow a normal distribution, Wilcoxon signed rank test will be used instead. The Hochberg method will be used to adjust for multiple testing and adjusted p-values reported.

14.3.1.3.1 Adapted MICRA

Adapted MICRA is assessed in all participants post-MCED test only. Descriptive statistics for the following scale scores will be provided:

- Distress scale
- Uncertainty scale
- Positive experience scale
- Total score

14.3.1.3.2 SF-12v2

Health-related quality of life is assessed in all participants at baseline (pre-test), post-test, at diagnostic resolution (MCED-V2 test positive only), and at 12 months (Appendix 2). Descriptive statistics will be provided for the scale scores derived from SF-12v2 (as described in Appendix 13) at each time point and change from baseline for each component summary measure:

- Mental component summary
- Mental health
- Role-emotional
- Social functioning

14.3.1.3.3 Satisfaction with MCED Test

Satisfaction with the MCED test is assessed post-test in MCED-V2 test negative participants and at diagnostic resolution in MCED-V2 test positive participants (Appendix 2). Descriptive statistics will be provided for a total score across all questions and individual ratings for each question.

14.3.1.3.4 Attitude Towards Subsequent MCED Test

Attitude towards subsequent MCED testing is assessed post-test in MCED-V2 test negative participants and at diagnostic resolution in MCED-V2 test positive participants (Appendix 2). Responses to the question "How likely are you to undergo a subsequent multi-cancer early detection test?" were represented by a 4-point Likert-type scale (i.e.Very Unlikely, Unlikely, Likely,

CONFIDENTIALITY
This document is the confidential and proprietary information of GRAIL, LLC. Except as required by federal, state, or local laws or regulations, this document may not be disclosed to others, in whole or in part without prior written permission of GRAIL, Inc.

and Very Likely). The number and proportion of participants with each rating will be reported.

14.3.2 Diagnostic evaluations

14.3.2.1 Characterize the number and type of diagnostic evaluations by predicted cancer signal origin and outcome of diagnostic resolution

Diagnostic evaluations

The number and type of diagnostic evaluations triggered by MCED-V2 positive test results in participants with a positive MCED test result will be summarized for the following four categories:

1. Imaging tests
    a. With radiation
    b. Without radiation
    c. Whole body imaging defined as PET scan (i.e., F-18 fluorodeoxyglucose (FDG) PET), PET-CT, PET-MRI, whole body MRI, whole body CT, or CT scan that includes all three of the following imaging fields : chest, abdomen and pelvis
2. Laboratory tests (including blood- and urine-based tests)
3. Non-invasive procedures (including physical exam)
4. Invasive procedures as described in Appendix 10
    a. Non-surgical invasive procedures
        i. Endoscopy
        ii. Biopsy
        iii. Other non-surgical invasive procedure
    b. Surgical invasive procedures

The following will be summarized:

- The number of participants with at least one diagnostic evaluation from each of the four categories described above will be reported overall and by type
- The total number of diagnostic evaluations from each of the four categories will be reported overall and by type
- The distribution of the per-participant counts for each of the four categories will be summarized using descriptive statistics.

Similar to Section 14.2.1.2, these analyses will be reported for the following groups of participants:

- Cancer vs no cancer diagnosis
    - Participants with cancer diagnosis (TP)
        - Participants with cancer diagnosis at diagnostic resolution
        - Participants with no cancer diagnosis at diagnostic resolution who have a cancer diagnosis at the end of the analysis period
    - Participants with no cancer diagnosis (FP)
        - Participants with no cancer diagnosis at diagnostic resolution and no cancer diagnosis at the end of the analysis period
        - Participants who did not reach a diagnostic resolution and so do not have a cancer diagnosis at the end of the analysis period

CONFIDENTIALITY
This document is the confidential and proprietary information of GRAIL, LLC. Except as required by federal, state, or local laws or regulations, this document may not be disclosed to others, in whole or in part without prior written permission of GRAIL, Inc.

- Initial vs subsequent diagnostic evaluation
    - Participants with an invasive procedure performed as part of their initial diagnostic evaluation (e.g., the first test(s) completed to evaluate a positive MCED-V2 test result)
    - Participants with an invasive procedure performed as part of the subsequent diagnostic evaluation after initial diagnostic evaluation (e.g., test(s) ordered after review of results from initial diagnostic evaluation)
    - In addition, we will describe how many participants had a biopsy performed
        - Without any prior or concurrent diagnostic evaluations
        - Concurrent with diagnostic evaluation that included
            - Imaging
            - Endoscopy
            - Surgery
        - After a diagnostic evaluation that included
            - Imaging
            - Endoscopy
            - Laboratory testing
            - Non-invasive procedure (i.e., physical exam)
- Targeted diagnostic evaluation vs evaluation after diagnostic PET-CT
    - Participants with an invasive procedure performed as part of a targeted diagnostic evaluation: any initial or subsequent testing performed before the diagnostic PET-CT (if any) or prior to diagnostic resolution. Diagnostic PET-CT was referred to as ‘confirmatory PET-CT’ in the study protocol and was intended to be performed only after a negative targeted diagnostic evaluation.
    - If the diagnostic PET-CT after a negative targeted diagnostic evaluation was performed, participants with an invasive procedure performed after the diagnostic PET-CT.
- CSO guided vs not CSO guided diagnostic evaluation
    - CSO guided diagnostic evaluation based on the diagnostic evaluation including imaging tests, laboratory tests, non-invasive procedures and invasive procedures: participants with the diagnostic evaluations consistent with the recommended targeted diagnostic testing for predicted CSO(s) as described in the study protocol (Appendix 11).
    - Not CSO guided diagnostic evaluation based on the diagnostic evaluation including imaging tests, laboratory tests, non-invasive procedures and invasive procedures: participants with diagnostic evaluations inconsistent with the recommended targeted diagnostic testing for predicted CSO(s)

The extent of diagnostic testing will be assessed in the following subgroups:

- CSO prediction groups (solid cancers, hematologic malignancies)
- CSO prediction
- Number of reported CSOs (one vs two) for MCED-V2

CONFIDENTIALITY
This document is the confidential and proprietary information of GRAIL, LLC. Except as required by federal, state, or local laws or regulations, this document may not be disclosed to others, in whole or in part without prior written permission of GRAIL, Inc.

The extent of diagnostic testing will be summarized for:

- All diagnostic evaluations prior to diagnostic resolution,
- The diagnostic evaluations starting from the diagnostic PET-CT performed after the initial negative diagnostic evaluation (referred to as the ‘confirmatory PET-CT’ in the study protocol) (inclusive) and conducted until diagnostic resolution in participants who had such diagnostic PET-CT.

Time to diagnostic resolution

The time required to achieve diagnostic resolution is defined as the number of days from the date when the MCED-V2 test result is communicated to the participant to the date of diagnostic resolution; it will be summarized using descriptive statistics by outcome of diagnostic resolution. If some participants did not reach a diagnostic resolution at the end of the analysis period, Kaplan-Meier methods will be used to estimate the time to diagnostic resolution.

14.3.2.2. Evaluate the radiation exposure due to the test result-directed evaluation

The number and type of imaging procedures with radiation exposure will be reported for participants with diagnostic evaluations triggered by MCED-V2 test positive results. To quantify radiation exposure for a participant as a result of their diagnostic workup, the radiation dose for each imaging test will be assessed based on the effective radiation dose estimates in Appendix 14.

The total amount of radiation exposure and the extent of radiation exposure on a per-participant basis will be summarized using descriptive statistics across:

- All MCED-V2 test positive participants
- Participants with MCED-V2 test positive results and cancer diagnosis at diagnostic resolution
- Participants with MCED-V2 test positive results and no cancer diagnosis at diagnostic resolution
- Participants with MCED-V2 test positive results who did not reach diagnostic resolution during the analysis period

This will be assessed overall and by CSO1 prediction.

The results will be reported for:

- All imaging tests performed prior to diagnostic resolution
- Imaging tests performed after targeted diagnostic testing (including diagnostic PET-CT [confirmatory PET-CT, as referred to in the study protocol]) and until diagnostic resolution in participants **where diagnostic resolution of cancer diagnosis is not achieved through the targeted diagnostic testing triggered by MCED test results**

CONFIDENTIALITY
This document is the confidential and proprietary information of GRAIL, LLC. Except as required by federal, state, or local laws or regulations, this document may not be disclosed to others, in whole or in part without prior written permission of GRAIL, Inc.

14.3.2.3 Describe the ability of diagnostic PET-CT to confirm cancer in participants where diagnostic resolution of cancer diagnosis is not achieved through the targeted diagnostic testing triggered by MCED test results

The ability of the PET-CT to detect cancer in cases for which CSO-guided workups do not result in diagnosis of cancer will be evaluated using the following endpoints:

1. Proportion of participants with cancer diagnosis at diagnostic resolution out of all participants who had a PET-CT only after initial negative diagnostic evaluation (no PET-CT during initial diagnostic evaluation)
2. Proportion of participants with cancer diagnosis at diagnostic resolution out of all participants with initial negative diagnostic evaluation who had a diagnostic PET-CT at any time during diagnostic evaluation

The distribution of cancer types, stage for new cancers and extent of recurrence for recurrent cancers among cancers diagnosed in participants with negative targeted diagnostic evaluation after diagnostic PET-CT will be reported.

14.3.3 Test performance

14.3.3.1 Evaluate Performance of the MCED Test in Selected Demographic and Clinical Subgroups.

Evaluation of MCED Test Performance in Selected Demographic and Clinical Subgroups

The following test performance endpoints will be evaluated:

- Cancer signal detection
    - PPV, NPV
    - Episode sensitivity, specificity
    - PLR, NLR
- CSO prediction
    - Accuracy of CSO1 prediction in TP
- Accuracy of CSO or CBS prediction in TP (MCED-I test only)
- Combined cancer signal detection and CSO1 prediction
    - PPV accounting for CSO accuracy
    - Episode sensitivity accounting for CSO accuracy

Selected Demographic and Clinical Subgroups

Test performance for MCED-V2 test and MCED-I test will be summarized descriptively using endpoints described above in the following subgroups:

1. Age: 50-64 vs 65+ years
2. Age: 50-59, 60-69,70-79, 80+ years

CONFIDENTIALITY
This document is the confidential and proprietary information of GRAIL, LLC. Except as required by federal, state, or local laws or regulations, this document may not be disclosed to others, in whole or in part without prior written permission of GRAIL, Inc.

3. Biological sex: male, female
4. Race categories for full representation, i.e. if reported alone or as part of multiple race categories (not mutually exclusive):
    - American Indian or Alaska Native
    - Asian, Native Hawaiian or Other Pacific Islander
    - Black or African American
    - White
5. Ethnicity (Hispanic or Latino vs not)
6. BMI: <25 kg/m^2, 25 to <30 kg/m^2, ≥30 kg/m^2
7. Smoking: never smoker, ever smoker
8. Prior cancer history: yes, no
9. Genetic cancer predisposition: yes, no

Evaluation of MCED Test Performance in Specific Cancer Subtypes

A descriptive summary of the distribution of the following cancer subtypes will be provided. In addition, the episode sensitivity for MCED-V2 and MCED-I will be summarized descriptively.

- Breast
    - Estrogen-receptor (ER) and progesterone-receptor (PR) negative and human epidermal growth factor receptor 2 (HER2) negative ('triple negative')
    - ER/PR positive and HER2 negative ('hormone receptor positive')
    - HER2 positive (regardless of ER/PR status)

- Lung
    - Adenocarcinoma non-small cell lung cancer
    - Squamous cell carcinoma non-small cell lung cancer
    - Neuroendocrine carcinoma (high grade - includes small cell carcinoma and large cell neuroendocrine carcinoma)
    - Carcinoid tumors
    - Other (includes Large cell carcinoma and NOS)

- Prostate (based on Gleason Grade Group[32])
    - Gleason Grade Group 1
    - Gleason Grade Group 2
    - Gleason Grade Group 3-5

[32] Grade Group 1 (Gleason score ≤ 6), Grade Group 2 (Gleason score 3+4), Grade Group 3 (Gleason score 4+3), Grade Group 4 (Gleason score 8), Grade Group 5 (Gleason score 9-10)
https://journals.lww.com/ajsp/abstract/2016/02000/the_2014_international_society_of_urological.10.aspx

CONFIDENTIALITY
This document is the confidential and proprietary information of GRAIL, LLC. Except as required by federal, state, or local laws or regulations, this document may not be disclosed to others, in whole or in part without prior written permission of GRAIL, Inc.

14.3.3.2 Evaluate MCED Test Positive Rate in Subgroups with Potential Cross-Reactive Conditions

Endpoint and Subgroups

MCED test positive rate is defined as the proportion of participants with MCED positive test results out of all participants with analyzable MCED test results.

MCED test positive rate will be evaluated in participants with the following potentially cross-reactive conditions that could interfere with the ability of the test to detect a cancer signal:

1. Non-malignant hematologic conditions (Langerhans cell histiocytosis, Lymphoproliferative disorders, Mastocytoma, Monoclonal B-cell lymphocytosis, Monoclonal gammopathy of undetermined significance, Post-transplant lymphoproliferative disorder, other)[33]
2. Participants who took any cytotoxic medications in the past year
3. Participants who took any demethylating agents in the past year
4. Participants with a prior cancer history
5. Conditions described in Table 7 which may shed cfDNA and/or may affect methylation

Table 7. Medical conditions to be evaluated for cross-reactivity

**Condition[34]**	**Description of condition**
Autoimmune disease	Multiple sclerosis, Rheumatoid arthritis, Systemic lupus erythematosus (lupus), Crohn's disease, ulcerative colitis
Chronic lung disease	Chronic obstructive pulmonary disease (COPD), emphysema, chronic bronchitis
HIV Infection	HIV infection
Pancreatitis	Acute pancreatitis, Chronic pancreatitis
Chronic liver disease	Chronic hepatitis B infection, Chronic hepatitis C infection, Cirrhosis (Liver cirrhosis), Fatty liver disease
Non-malignant gastrointestinal conditions	Barrett's esophagus, Chronic gastritis, Esophageal reflux disease, Stomach ulcers
Diabetes	Diabetes
Transplantation	Solid organ or stem cell (bone marrow) transplant

[33] Answered "Yes" to the question "Has the participant ever been diagnosed with any of the following hematologic conditions prior to study enrollment?"
[34] Answered "Yes" to "Please confirm if a doctor has ever told the participant that they have any of the following health/medical conditions (check all that apply)."

CONFIDENTIALITY
This document is the confidential and proprietary information of GRAIL, LLC. Except as required by federal, state, or local laws or regulations, this document may not be disclosed to others, in whole or in part without prior written permission of GRAIL, Inc.

Analysis Methods

A Pearson chi-square test will be used to assess if participants with any of the potential cross-reactive conditions have a significantly different MCED test positive test rate compared to the MCED test positive rate in participants without the condition. If any condition is found to have a significantly higher MCED test positive rate, an estimate of the impact on false positives and specificity will be assessed based on the prevalence of these conditions in the intended use population.

This analysis will be completed for MCED-V2 test and MCED-I test.

14.3.3.3 Evaluate concordance of initial GRAIL test result and CSO prediction, with results from the research blood draw

The results of the MCED test from the blood draw at enrollment (initial blood draw) and research blood draw at the time of the diagnostic PET-CT will be compared in participants who had both blood draws. It should be noted that research blood draws can be obtained only in participants with positive initial MCED-V2 test results. For participants with MCED results from both the initial and research blood draws, a 2x2 matrix will be constructed separately for participants with cancer diagnosis, participants without cancer diagnosis, and all participants combined (similar to the example in Table 6). Similar to methods described in section 14.2.2.1.6, percent agreement (PPA, NPA, OPA) and corresponding confidence intervals will be calculated.

In addition, a descriptive summary of the concordance between MCED CSO1 predictions from the initial and research blood draws will be provided. It will be presented as a matrix and the proportion of participants with the same CSO1 predictions will be reported in participants with cancer diagnosis, participants without cancer diagnosis, and all participants combined.

14.4 Analysis Methods for Exploratory Objectives

14.4.1 Evaluate performance of the MCED test over three years of follow-up.

MCED test performance endpoints for overall cancer signal detection and overall CSO accuracy (as described in Sections 14.2.2.1.1 and 14.2.2.1.2) will be assessed based on cancer status after 3 years (36 months) or less of follow-up time for both test versions. The distribution of cancer types and stages for cancers reported in the last 12 months of study follow-up will be summarized for participants with MCED test positive results and MCED test negative test results. This analysis will be performed after the 3 year follow-up period for all study participants has ended.

CONFIDENTIALITY

This document is the confidential and proprietary information of GRAIL, LLC. Except as required by federal, state, or local laws or regulations, this document may not be disclosed to others, in whole or in part without prior written permission of GRAIL, Inc.

14.4.2 Evaluate changes in blood-based biomarkers among the subset of participants with a research blood draw.

Blood biomarker signals from the initial and research blood draws will be assessed in the subset of participants with a research blood draw.

14.4.3 Evaluate different CSO reporting classes based on clinically relevant subgroups.

MCED test results for CSO prediction and blood biomarker signals from the initial and research blood draws will be evaluated in the context of diagnostic testing triggered by the test results.

15. Analysis Methods for Adverse Events

The number of adverse events (AEs) observed in participants in the Consented Analysis set (as described in Section 10.1) will be summarized. The number of AEs in participants with MCED-V2 positive, MCED-V2 negative, no analyzable test results and all consented participants will be characterized by the following:

- Setting (phlebotomy, anticipation or communication of MCED-V2 test result, diagnostic procedure prompted by a positive MCED-V2 test result, other setting)
- Seriousness (whether AE was considered an SAE)
  - If yes, seriousness criteria (death, life threatening, required hospitalization-initial or prolonged, results in persistent or significant disability/incapacity, required intervention to prevent permanent impairment, congenital anomaly/birth defect, other medically important serious event)
- Severity (mild, moderate, severe)
- Relatedness to study activities (i.e., study activities listed in the protocol Schedule of Activities) or device
- Outcome (e.g. recovered/ resolved, recovered/ resolved with sequelae, recovering/ resolving, not recovered/ not resolved, fatal)

AEs will be described separately for participants with MCED-V2 positive, MCED-V2 negative, no analyzable test results and all consented participants for the following subsets:

- Related to study activities, related to device
- SAEs

A per-participant listing of SAEs will be provided.

16. Protocol Deviations

Protocol deviations that occur after participants entered the study are documented during routine monitoring.

Important protocol deviations are a subset of protocol deviations that might significantly affect the completeness, accuracy, and/or reliability of the study data or that might significantly affect

CONFIDENTIALITY

This document is the confidential and proprietary information of GRAIL, LLC. Except as required by federal, state, or local laws or regulations, this document may not be disclosed to others, in whole or in part without prior written permission of GRAIL, Inc.

participant's rights, safety, or well-being[35].

Important protocol deviations will be summarized for all PATHFINDER 2 participants for the following categories:

- Participants who had at least one important protocol deviation
- Participants who did not meet at least one eligibility criterion but were enrolled into the study
- Participants whose informed consent form deviated from protocol version.

A per-participant listing of important protocol deviations will also be provided.

17. Sample Size and Power

The expected number of positive test results, number of cancers diagnosed, number of cancers detected through diagnostic evaluation triggered by the MCED test in analyzable participants and test performance endpoints were estimated using microsimulations[36].

Overall Expected Results

The results of the microsimulations in Table 8 describe the number of participants with MCED positive test results expected among 25,000 enrolled participants (23,750 analyzable participants assuming 5% exclusions) with 12 months of follow-up for the PMA analysis. The results are provided for two scenarios: based on specificity of 99.0% and 99.5%. In the scenario with 99.5% specificity, a total of 218 MCED positive test results are expected with approximately 102 TP and 116 FP (47% PPV). Under conservative assumptions about cancer incidence used in these microsimulations to account for a possible healthy volunteer effect (assuming that underlying cancer incidence in the enrolled population is 65% of SEER cancer incidence with sex and age distribution described in the study protocol) we expect 244 cancers diagnosed among 25,000 enrolled participants during 12 months follow-up time. Note, that the microsimulation accounts for 17 cancer types only (cancer types for which dwell time scenarios have been developed and which have sufficient stage data in SEER to support modeling); if cancers not included in the simulation are taken into account, we estimate the total number of cancers to be 264 or higher. Table 8 provides expected estimates of PPV, NPV and episode sensitivity for all cancers.

---

[35] FDA (2013). *E3 Structure and Content of Clinical Study Reports - Questions and Answers (R1)*. Retrieved https://www.fda.gov/downloads/Drugs/GuidanceComplianceRegulatoryInformation/Guidances/UCM336889.pdf

[36] Dai JY et al, 'Clinical performance and utility: A microsimulation model to inform the design of screening trials for a multi-cancer early detection test', Journal of Medical Screening OnlineFirst, https://journals.sagepub.com/doi/full/10.1177/09691413241228041

CONFIDENTIALITY

This document is the confidential and proprietary information of GRAIL, LLC. Except as required by federal, state, or local laws or regulations, this document may not be disclosed to others, in whole or in part without prior written permission of GRAIL, Inc.

Table 8. Results of Microsimulations for 25,000 enrolled participants

Specificity	Number of Test Positive Results (95% CI)	% Test Positive (95% CI)	PPV (%) (95% CI)*	NPV (%) (95% CI)*	Episode Sensitivity (%) (95% CI)*
99.0%	335 (304,365)	1.41 (1.28, 1.54)	30.1 (25.4, 35.1)	99.4 (99.3-99.5)	41.5 (35.5, 47.3)
99.5%	218 (193, 248)	0.92 (0.81, 1.04)	47.0 (40.3, 53.2)	99.4 (99.3-99.5)	42.2 (36.9, 48.1)

*Entries in the table represent median (2.5 and 97.5 percentiles) of a specific measure across 200 simulations

Using similar logic, a total of approximately 370 cancers are expected to be diagnosed among 35,000 enrolled participants (or 33,250 analyzable participants) during 12 months of follow-up time.

Power Calculations for Study Success Criteria

The study success criteria for PATHFINDER 2 study are described in Section 14.2.2.2. Power calculations for specificity, taking sampling into account, are described in Appendix 9. Power calculations for study success criteria based on episode sensitivity for all cancers, and accuracy of CSO prediction in participants with TP MCED test results are provided in Table 9 and Table 10, respectively. As described above, a total of 264 cancers are expected to be diagnosed among 25,000 participants with 12 months of follow-up at the time of the PMA analysis. Power calculations were conducted using the Wilson method to compute two-sided 95% confidence intervals based on 10,000 simulations using binomial distribution at expected sample sizes for each endpoint. The power was determined as the proportion of the simulated results where the lower bound of the 95% confidence interval exceeded the prespecified study success criteria threshold for the corresponding endpoint.

Table 9. Power calculations for episode sensitivity study success criteria

Cancer Group	Acceptance Criteria Threshold	Potential Episode Sensitivity Estimate	Power
All cancers (N=264)	10%	20%	99%
		30%	>99%
		40%	>99%

CONFIDENTIALITY

This document is the confidential and proprietary information of GRAIL, LLC. Except as required by federal, state, or local laws or regulations, this document may not be disclosed to others, in whole or in part without prior written permission of GRAIL, Inc.

Table 10. Power calculations for study success criterion for the accuracy of CSO prediction in participants with a true positive MCED test result

Study Success Criteria Threshold for the accuracy of CSO prediction	Potential CSO accuracy Estimate*	Power
70%	75%	16%
	80%	54%
	85%	91%
	90%	>99%

*Based on conservative estimate of having at least 86 expected true positive participants

CONFIDENTIALITY
This document is the confidential and proprietary information of GRAIL, LLC. Except as required by federal, state, or local laws or regulations, this document may not be disclosed to others, in whole or in part without prior written permission of GRAIL, Inc.

Appendix 1 PATHFINDER 2 Inclusion/Exclusion Criteria

Inclusion and exclusion criteria below are from the "The PATHFINDER 2 Study: Evaluating the Safety and Performance of the GRAIL Multi-Cancer Early Detection Test in an Eligible Screening Population" Protocol Version Number: 3.0, dated 09Aug2023. Any modifications to these criteria will be reflected in the clinical study report. Participants are eligible to be included in the study only if all of the following criteria apply:

Inclusion Criteria

Participants are eligible to be included in the study only if all of the following criteria apply.

1. Participants must be at least 50 years of age, inclusive, at the time of signing the Informed Consent Form (ICF).

2. Informed Consent
   - Capable of giving signed and legally effective informed consent as described in Section 5.4, Study Design Schema, which includes compliance with the requirements and restrictions listed in the Informed Consent Form (ICF) and in this protocol. Consent provided by a legally authorized representative is not permitted in this protocol.

Exclusion Criteria

1. Undergoing or referred for diagnostic evaluation due to clinical suspicion for cancer (e.g., referred to a medical or surgical oncologist, or scheduled for biopsy on the basis of a suspicious imaging abnormality).

2. Personal history of invasive solid tumor or hematologic malignancy, diagnosed within the 3 years prior to expected enrollment date, or diagnosed greater than 3 years prior to expected enrollment date and never treated.
   - Individuals with a diagnosis of non-metastatic basal cell carcinoma and squamous cell carcinoma of the skin are not excluded.

3. Prior/Concurrent Concomitant Therapy (Medications/Treatments):
   - Definitive treatment for invasive solid tumor or hematologic malignancy within the 3 years prior to expected enrollment date. Adjuvant hormone therapy for cancer (e.g. for breast or prostate cancer) is not an exclusion criterion.

4. Individuals who will not be able to comply with the protocol procedures.

5. Individuals who are not currently registered patients at a participating center.

6. Previous or current participation in another GRAIL-sponsored study. "Participation" is defined as having signed consent and provided a blood sample.

7. Previous or current employees or contractors of GRAIL.

8. Current pregnancy (by self-report of pregnancy status)

CONFIDENTIALITY
This document is the confidential and proprietary information of GRAIL, LLC. Except as required by federal, state, or local laws or regulations, this document may not be disclosed to others, in whole or in part without prior written permission of GRAIL, Inc.

APPENDIX 2 PARTICIPANT-REPORTED OUTCOME DATA COLLECTION TIME POINTS

Instrument	Pre-Test	Post-Test	Post-Test	At Diagnostic Resolution	12 months
Participant Group	All	No Signal Detected	Signal Detected	Signal Detected	ALL
SF-12v2 Health Survey	X	X	X	X	X
Multidimensional Impact of Cancer Risk Assessment (Adapted MICRA)		X	X		
State Trait Anxiety Inventory (STAI)+ MCED STAI questionnaires	X	X	X	X	X
Satisfaction with the MCED test		X		X	
Attitude towards adherence to guideline-recommended screening	X	X	X		X
Attitude towards subsequent MCED test		X		X	

CONFIDENTIALITY
This document is the confidential and proprietary information of GRAIL, LLC. Except as required by federal, state, or local laws or regulations, this document may not be disclosed to others, in whole or in part without prior written permission of GRAIL, Inc.

Appendix 3A CSO predictions for the MCED-V2 test

N	CSO predictions
1	Anus
2	Bladder, Urothelial tract
3	Breast
4	Bone and Soft tissue
5	Cervix
6	Colon, Rectum
7	Head and Neck
8	Kidney
9	Liver, Bile duct
10	Lung
11	Lymphoid Lineage
12	Melanocytic Lineage
13	Myeloid Lineage
14	Neuroendocrine cells of lung or other organs*
15	Ovary
16	Pancreas, Gallbladder
17	Plasma Cell Lineage
18	Prostate
19	Stomach, Esophagus
20	Thyroid gland
21	Uterus

* Neuroendocrine cancers include
- lung neuroendocrine cancers (low grade or high grade)
- high grade neuroendocrine tumors for: bladder, cervix, colon/rectum, esophagus, head and neck, gallbladder, stomach, pancreas, prostate, urothelial tract, uterus

CSOs are mutually exclusive so the endocrine tumors included in “Neuroendocrine” CSO are excluded from other CSOs. For example, high grade neuroendocrine bladder cancer will be included in the “Neuroendocrine” CSO and not be included in the “Bladder / Urothelial tract” CSO.

CONFIDENTIALITY
This document is the confidential and proprietary information of GRAIL, LLC. Except as required by federal, state, or local laws or regulations, this document may not be disclosed to others, in whole or in part without prior written permission of GRAIL, Inc.

Appendix 3B MCED-V2 CSO specification

CSO class	Description	Examples for included cancers	Examples for excluded cancers	Topography name	Morphology group
Anus	Methylation signal associated with carcinomas of the anus and anal canal. The main contributor to this class is HPV-associated squamous cell carcinoma of the anus.	HPV-associated squamous cell carcinoma of the anus	Skin cancers reported in the anal region	Rectosigmoid junction	squamous cell neoplasms
		Anal gland adenocarcinoma	Squamous cell carcinoma reported in the colon, even if HPV-positive		
		HPV-positive squamous cell carcinoma reported with primary site rectum	Sarcoma or lymphoma reported in the anal region		
				Rectum, NOS	squamous cell neoplasms
				Anus, NOS (excludes Skin of anus and Perianal skin (C44.5)<br>Anal canal<br>Cloacogenic zone<br>Overlapping lesion of rectum, anus and anal canal	adenocarcinomas
					epithelial neoplasms, NOS
					medullary cancers
					squamous cell neoplasms
Bladder, Urothelial tract	Methylation signal associated with carcinomas of the bladder, ureter, urethra and renal pelvis, excluding high grade neuroendocrine tumors	Adenocarcinoma of the bladder	Renal cell carcinomas reported at the renal pelvis as anatomic site	Renal pelvis<br>Ureter<br>Trigone, bladder<br>Dome, bladder<br>Lateral wall bladder	adenocarcinomas
		Transitional cell carcinoma of the bladder	Sarcoma or lymphoma reported in the bladder,		carcinoid neuroendocrine neoplasms

CONFIDENTIALITY
This document is the confidential and proprietary information of GRAIL, LLC. Except as required by federal, state, or local laws or regulations, this document may not be disclosed to others, in whole or in part without prior written permission of GRAIL, Inc.

CSO class	Description	Examples for included cancers	Examples for excluded cancers	Topography name	Morphology group
			ureter or renal pelvis	Posterior wall bladder<br>Ureteric orifice<br>Urachus<br>Overlapping lesion of bladder<br>Bladder NOS<br>Urethra	
		Urothelial carcinoma of the renal pelvis	Small cell carcinoma of the bladder		complex epithelial neoplasms - adenocarcinoma
		Carcinoma of the ureter			complex epithelial neoplasms - all other
		Adenocarcinoma NOS in the renal pelvis			cystic, mucinous and serous neoplasms
		Urothelial carcinoma in the kidney			epithelial neoplasms, NOS
					medullary cancers
					squamous cell neoplasms
					transitional cell papillomas and carcinomas
					unspecified neoplasms
				Kidney NOS	transitional cell papillomas and carcinomas
Bone and Soft tissue	Methylation signal associated with sarcomas and other cancers of mesenchymal origin in bone and soft tissue independent from anatomic site	Uterine leiomyosarcoma		Any	blood vessel tumors
		Malignant solitary fibrous tumor			chordomas
		Osteosarcoma			complex mixed and stromal neoplasms - sarcoma

CONFIDENTIALITY
This document is the confidential and proprietary information of GRAIL, LLC. Except as required by federal, state, or local laws or regulations, this document may not be disclosed to others, in whole or in part without prior written permission of GRAIL, Inc.

CSO class	Description	Examples for included cancers	Examples for excluded cancers	Topography name	Morphology group
		Gastrointestinal stromal tumor			fibromatous neoplasms
		Malignant fibrous histiocytoma (of skin)			giant cell tumors
		Malignant Hemangiopericytoma in the brain			glomus tumors
					lipomatous neoplasms
					lymphatic vessel tumors
					miscellaneous bone tumors (C40._, C41._)
					myomatous neoplasms
					myxomatous neoplasms
					nerve sheath tumors
					osseous and chondromatous neoplasms
					phyllodes
					soft tissue tumors and sarcomas, NOS
					synovial-like neoplasms

CONFIDENTIALITY
This document is the confidential and proprietary information of GRAIL, LLC. Except as required by federal, state, or local laws or regulations, this document may not be disclosed to others, in whole or in part without prior written permission of GRAIL, Inc.

CSO class	Description	Examples for included cancers	Examples for excluded cancers	Topography name	Morphology group
				Skull and facial bone<br>Connective tissue head<br>Connective tissue arm<br>Connective tissue leg<br>Connective tissue thorax (excludes Thymus, Heart and Mediastinum C37. , C38.)<br>Connective tissue abdomen<br>Connective tissue pelvis<br>Connective tissue trunk, NOS<br>Overlapping lesion of connective, subcutaneous and other soft tissues<br>Connective tissue NOS	unspecified neoplasms
Breast	Methylation signal associated with carcinomas of the breast, excluding high grade neuroendocrine tumors	Invasive ductal breast carcinoma	Sarcoma or lymphoma reported in the breast	Nipple<br>Central portion of breast<br>Upper inner quadrant of breast<br>Lower inner quadrant of breast<br>Upper outer quadrant of breast<br>Lower outer	acinar cell neoplasms
		Invasive lobular breast carcinoma	Any invasive skin cancer of the breast		adenocarcinomas
		Inflammatory carcinoma			carcinoid neuroendocrine neoplasms
					complex epithelial neoplasms -

CONFIDENTIALITY
This document is the confidential and proprietary information of GRAIL, LLC. Except as required by federal, state, or local laws or regulations, this document may not be disclosed to others, in whole or in part without prior written permission of GRAIL, Inc.

CSO class	Description	Examples for included cancers	Examples for excluded cancers	Topography name	Morphology group
				quadrant of breast<br>Axillary tail of breast<br>Overlapping lesion of breast<br>Breast NOS (excludes Skin of breast C44.5)	adenocarcinoma
					complex epithelial neoplasms - all other
					cystic, mucinous and serous neoplasms
					ductal and lobular neoplasms
					epithelial neoplasms, NOS
					medullary cancers
					squamous cell neoplasms
					unspecified neoplasms
Cervix	Methylation signal associated with carcinomas of the cervix. The main contributor to this class is HPV-associated squamous cell carcinoma of the cervix, excluding high grade neuroendocrine tumors	HPV-associated squamous cell carcinoma of the cervix		Endocervix<br>Exocervix<br>Cervix uteri<br>Overlapping lesion of cervix uteri	adenocarcinomas
		HPV-associated adenocarcinoma of the cervix			adenoid basal
		Low grade neuroendocrine carcinoma of the cervix			carcinoid neuroendocrine neoplasms
		Non-HPV-associated adenocarcinoma of the cervix			complex epithelial neoplasms - adenocarcinoma
					complex epithelial neoplasms - all

CONFIDENTIALITY
This document is the confidential and proprietary information of GRAIL, LLC. Except as required by federal, state, or local laws or regulations, this document may not be disclosed to others, in whole or in part without prior written permission of GRAIL, Inc.

CSO class	Description	Examples for included cancers	Examples for excluded cancers	Topography name	Morphology group
					other
					cystic, mucinous and serous neoplasms
					epithelial neoplasms, NOS
					medullary
					mesonephromas
					squamous cell neoplasms
					transitional cell papillomas and carcinomas
					unspecified neoplasms
Colon, Rectum	Methylation signal associated with carcinomas of the colon, rectum and appendix, excluding high grade neuroendocrine tumors	Colorectal adenocarcinoma	HPV-positive squamous cell carcinoma reported in the rectum	Cecum<br>Appendix<br>Ascending colon<br>Hepatic flexure of colon<br>Transverse colon<br>Splenic flexure of colon<br>Descending colon<br>Sigmoid colon<br>Overlapping lesion of colon<br>Colon NOS	adenocarcinomas
		Signet ring cell carcinoma of colon	Sarcoma or lymphoma reported in colon or rectum		carcinoid neuroendocrine neoplasms
		Carcinoid in colon	Adenocarcinoma or carcinoid of the small intestines		complex epithelial neoplasms - adenocarcinoma
		Adenocarcinoma in the appendix			complex epithelial neoplasms - all other
		Carcinoid of the appendix			cystic, mucinous and serous neoplasms

CONFIDENTIALITY
This document is the confidential and proprietary information of GRAIL, LLC. Except as required by federal, state, or local laws or regulations, this document may not be disclosed to others, in whole or in part without prior written permission of GRAIL, Inc.

CSO class	Description	Examples for included cancers	Examples for excluded cancers	Topography name	Morphology group
		Squamous cell carcinoma in colon			ductal and lobular neoplasms
					epithelial neoplasms, NOS
					medullary cancers
					squamous cell neoplasms
					unspecified neoplasms
				Rectosigmoid junction<br>Rectum, NOS	adenocarcinomas
					carcinoid neuroendocrine neoplasms
					complex epithelial neoplasms - adenocarcinoma
					complex epithelial neoplasms - all other
					cystic, mucinous and serous neoplasms
					ductal and lobular neoplasms
					epithelial neoplasms, NOS
					unspecified neoplasms
Head and	Methylation signal associated with HPV-associated	Oropharyngeal HPV-associated squamous cell	Sarcoma or lymphoma reported	Upper lip, mucosa<br>Lower lip, mucosa	acinar cell neoplasms

CONFIDENTIALITY
This document is the confidential and proprietary information of GRAIL, LLC. Except as required by federal, state, or local laws or regulations, this document may not be disclosed to others, in whole or in part without prior written permission of GRAIL, Inc.

CSO class	Description	Examples for included cancers	Examples for excluded cancers	Topography name	Morphology group
Neck	and HPV-negative carcinomas of the head and neck region including oropharynx, hypopharynx, nasopharynx, larynx, lip and oral cavity (including oral tongue), nasal cavity, paranasal sinuses and major salivary glands, excluding high grade neuroendocrine tumors	carcinoma	in the H&N region	Mucosa lip, NOS<br>Base of tongue, NOS<br>Dorsal surface tongue, NOS<br>Border of tongue<br>Ventral surface of tongue NOS<br>Anterior 2/3 of tongue NOS<br>Lingual tonsil<br>Overlapping lesion of tongue<br>Tongue NOS<br>Upper gum<br>Lower gum<br>Gum NOS<br>Anterior floor of mouth<br>Lateral floor of mouth<br>Floor of mouth NOS<br>Hard palate<br>Soft palate NOS (excludes Nasopharyngeal surface C11.3)<br>Uvula<br>Palate NOS<br>Cheek mucosa	
		HPV-negative squamous cell carcinoma of the larynx	Skin cancers reported in the H&N region		adenocarcinomas
		Salivary gland adenocarcinoma			carcinoid neuroendocrine neoplasms
					complex epithelial neoplasms - adenocarcinoma
					complex epithelial neoplasms - all other
					complex mixed and stromal neoplasms - epithelial
					ductal and lobular neoplasms
					epithelial neoplasms, NOS
					mucoepidermoid neoplasms
					medullary cancers
					squamous cell neoplasms

CONFIDENTIALITY
This document is the confidential and proprietary information of GRAIL, LLC. Except as required by federal, state, or local laws or regulations, this document may not be disclosed to others, in whole or in part without prior written permission of GRAIL, Inc.

CSO class	Description	Examples for included cancers	Examples for excluded cancers	Topography name	Morphology group
				Vestibule of mouth<br>Retromolar area<br>Overlapping lesion of other and unspecified parts of mouth<br>Mouth NOS<br>Parotid gland<br>Submaxillary gland<br>Sublingual gland<br>Major salivary gland, NOS<br>Tonsillar fossa<br>Tonsillar pillar<br>Overlapping lesion of tonsil<br>Tonsil NOS (excludes Lingual tonsil C02.4 and Pharyngeal tonsil C11.1)<br>Vallecula<br>Anterior surface of epiglottis<br>Lateral wall oropharynx<br>Posterior wall oropharynx<br>Branchial cleft (site of neoplosm)<br>Overlapping lesion of oropharynx	unspecified neoplasms

CONFIDENTIALITY
This document is the confidential and proprietary information of GRAIL, LLC. Except as required by federal, state, or local laws or regulations, this document may not be disclosed to others, in whole or in part without prior written permission of GRAIL, Inc.

CSO class	Description	Examples for included cancers	Examples for excluded cancers	Topography name	Morphology group
				Oropharynx NOS<br>Superior wall of nasopharynx<br>Posterior wall nasopharynx<br>Lateral wall nasopharynx<br>Anterior wall nasopharynx<br>Overlapping lesion of nasopharynx<br>Nasopharynx NOS<br>Pyriform sinus<br>Postcricoid region<br>Hypopharyngeal aspect of aryepiglottic fold<br>Posterior wall hypopharynx<br>Hypopharynx, NOS<br>Pharynx NOS<br>Nasal cavity (excludes Nose, NOS C76.0)<br>Maxillary sinus<br>Ethmoid sinus<br>Glottis<br>Supraglottis<br>Subglottis<br>Larynx NOS	

CONFIDENTIALITY
This document is the confidential and proprietary information of GRAIL, LLC. Except as required by federal, state, or local laws or regulations, this document may not be disclosed to others, in whole or in part without prior written permission of GRAIL, Inc.

CSO class	Description	Examples for included cancers	Examples for excluded cancers	Topography name	Morphology group
				Overlapping lesion of floor of mouth<br>Overlapping lesion of palate<br>Overlapping lesion of major salivary glands<br>Overlapping lesion of hypopharynx	
				Head, face or neck NOS	squamous cell neoplasms
Kidney	Methylation signal associated with carcinomas of the renal cortex and renal tubules, excluding high grade neuroendocrine tumors	Renal cell carcinoma	Sarcoma or lymphoma reported in the kidney	Kidney NOS	adenocarcinomas
		Carcinoid in kidney	Transitional or urothelial carcinomas reported in the kidney		carcinoid neuroendocrine neoplasms
		Adenocarcinoma NOS in the kidney			complex mixed and stromal neoplasms - epithelial
					epithelial neoplasms, NOS
					medullary cancers
					renal cell carcinomas
					unspecified neoplasms
				Renal pelvis	renal cell carcinomas
Liver, Bile duct	Methylation signal associated with carcinomas of the	Hepatocellular carcinoma	Carcinoma of the perihilar bile duct	Liver<br>Intrahepatic bile	adenocarcinomas

CONFIDENTIALITY
This document is the confidential and proprietary information of GRAIL, LLC. Except as required by federal, state, or local laws or regulations, this document may not be disclosed to others, in whole or in part without prior written permission of GRAIL, Inc.

CSO class	Description	Examples for included cancers	Examples for excluded cancers	Topography name	Morphology group
	liver and intrahepatic bile duct, excluding high grade neuroendocrine tumors	Intrahepatic cholangiocarcinoma	Sarcoma or lymphoma reported in the liver	duct	carcinoid neuroendocrine neoplasms
		Carcinoid in the liver	Small cell carcinoma in the liver		combined hepatocellular carcinoma (HCC) / intrahepatic cholangiocarcinoma iCC
					epithelial neoplasms, NOS
					hepatocellular carcinomas
					medullary cancers
					squamous cell neoplasms
					unspecified neoplasms
Lung	Methylation signal associated with carcinomas of the lung and bronchus, excluding neuroendocrine tumors	Lung adenocarcinoma	Sarcoma or lymphoma reported in the lung	Main bronchus<br>Upper lobe, lung<br>Middle lobe, lung<br>Lower lobe, lung<br>Overlapping lesion of lung<br>Lung NOS	acinar cell neoplasms
		Lung squamous cell carcinoma	Small Cell Lung Cancer (SCLC)		adenocarcinomas
		Non Small Cell Lung Cancer (NSCLC) NOS	Carcinoid in the lung		complex epithelial neoplasms - adenocarcinoma
					complex epithelial neoplasms - all other
					complex mixed and stromal neoplasms - epithelial

CONFIDENTIALITY
This document is the confidential and proprietary information of GRAIL, LLC. Except as required by federal, state, or local laws or regulations, this document may not be disclosed to others, in whole or in part without prior written permission of GRAIL, Inc.

CSO class	Description	Examples for included cancers	Examples for excluded cancers	Topography name	Morphology group
					cystic, mucinous and serous neoplasms
					epithelial neoplasms, NOS
					medullary cancers
					mucoepidermoid neoplasms
					squamous cell neoplasms
					unspecified neoplasms
Lymphoid Lineage	Methylation signal associated with blood, lymph nodes and bone marrow with a cell of origin with lymphoid lineage (but not a plasma cell).	Hodgkin and non-hodgkin lymphoma	Plasma Cell Neoplasms	any	hodgkin lymphomas
		T-cell lymphoma			lymphoid leukemia/lymphoma
		Chronic Lymphocytic Leukemia (CLL)/ Small Lymphocytic Lymphoma (SLL)			malignant lymphomas, NOS or diffuse
		Primary cutaneous follicle center lymphoma			non-hodgkin lymphoma - mature b-cell lymphomas
		Precursor B and T lymphoblastic leukemia			non-hodgkin lymphoma - mature t and nk-cell lymphomas
		Gastric mucosa-associated lymphoid		Bone marrow	lymphoid leukemias

CONFIDENTIALITY
This document is the confidential and proprietary information of GRAIL, LLC. Except as required by federal, state, or local laws or regulations, this document may not be disclosed to others, in whole or in part without prior written permission of GRAIL, Inc.

CSO class	Description	Examples for included cancers	Examples for excluded cancers	Topography name	Morphology group
		tissue lymphoma<br><br>Waldenstrom macroglobulinemia			
Melanocytic Lineage	Methylation signal associated with melanomas, including ocular melanomas, sinonasal melanomas, and anogenital melanomas. Other skin cancers, including sweat gland carcinomas and Merkel cell carcinoma, digital papillary adenocarcinoma, basal and squamous cell carcinomas are not included in this class.	Melanoma of extremities<br><br>Melanoma in the H&N region	Basal cell carcinoma of skin<br><br>Squamous cell carcinoma of skin	External lip upper<br>External lip lower<br>External lip NOS<br>Commissure lip<br>Upper gum<br>Anus, NOS (excludes Skin of anus and Perianal skin (C44.5)<br>Anal canal<br>Nasal cavity (excludes Nose, NOS C76.0)<br>Maxillary sinus<br>Ethmoid sinus<br>Skin lip, NOS<br>Eyelid NOS<br>External ear<br>Skin face<br>Skin scalp, neck<br>Skin trunk<br>Skin limb, upper<br>Skin limb, lower<br>Labium majus<br>Labium minus	nevi and melanomas

CONFIDENTIALITY

This document is the confidential and proprietary information of GRAIL, LLC. Except as required by federal, state, or local laws or regulations, this document may not be disclosed to others, in whole or in part without prior written permission of GRAIL, Inc.

CSO class	Description	Examples for included cancers	Examples for excluded cancers	Topography name	Morphology group
				Clitoris<br>Vulva, NOS<br>Vagina, NOS<br>Prepuce<br>Glans penis<br>Body penis<br>Penis NOS<br>Scrotum, NOS<br>Conjunctiva<br>Retina<br>Choroid<br>Ciliary body	
Myeloid Lineage	Methylation signal associated with blood, lymph nodes and bone marrow with a cell of origin with myeloid lineage. Myeloid leukemias are the dominant contributor in this class.	Acute Myeloid Leukemia (AML) and Chronic Myeloid Leukemia (CML)		any	mast cell tumors
		Myelodysplastic Syndrome (MDS)			myelodysplastic syndrome
		Malignant mastocytosis			neoplasm of histiocytes and accessory lymphoid cells
		Myeloid sarcoma		Bone marrow	chronic myeloproliferative disorders
		Acute erythroid leukemia			myeloid leukemias
		Essential thrombocythemia			
		Primary			

CONFIDENTIALITY
This document is the confidential and proprietary information of GRAIL, LLC. Except as required by federal, state, or local laws or regulations, this document may not be disclosed to others, in whole or in part without prior written permission of GRAIL, Inc.

CSO class	Description	Examples for included cancers	Examples for excluded cancers	Topography name	Morphology group
		Myelofibrosis			
		Polycythemia vera			
Neuroendocrine cells of lung or other organs	Methylation signal associated with neuroendocrine tumors of the lung, or high grade neuroendocrine tumors at all other organs	Carcinoids of the lung		Main bronchus<br>Upper lobe, lung<br>Middle lobe, lung<br>Lower lobe, lung<br>Overlapping lesion of lung<br>Lung NOS	carcinoid neuroendocrine neoplasms
		Small cell carcinomas<br>Large cell neuroendocrine carcinomas at all organs (including lung)		any	high grade neuroendocrine tumors
		High grade neuroendocrine tumors at all organs (including lung)		any	small cell carcinomas, large cell neuroendocrine tumors
Ovary	Methylation signal associated with carcinomas and other solid tumors with cell of origin from the ovary, fallopian tubes or primary peritoneum, excluding high grade neuroendocrine tumors	Fallopian tube-derived serous cystadenocarcinoma of the ovaries	Germ cell tumors	Peritoneum<br>Peritoneum NOS<br>Overlapping lesion of retroperitoneum and peritoneum	adenocarcinomas
		Endometrioid ovarian carcinoma	Sarcoma or lymphoma reported in the ovaries or peritoneal region		carcinoid neuroendocrine neoplasms
		Malignant mullerian mixed	Mesothelioma in the peritoneal		complex mixed and stromal neoplasms -

CONFIDENTIALITY
This document is the confidential and proprietary information of GRAIL, LLC. Except as required by federal, state, or local laws or regulations, this document may not be disclosed to others, in whole or in part without prior written permission of GRAIL, Inc.

CSO class	Description	Examples for included cancers	Examples for excluded cancers	Topography name	Morphology group
		carcinoma of the ovaries	region		epithelial
		Clear cell carcinoma of the ovaries	Small cell carcinoma of the ovaries		cystic, mucinous and serous neoplasms
		Malignant Brenner tumor			epithelial neoplasms, NOS
					fibroepithelial neoplasms without phyllodes
					medullary cancers
					mesonephromas
					specialized gonadal neoplasms
					squamous cell neoplasms
					unspecified neoplasms
				Ovary<br>Fallopian tube	adenocarcinomas
					carcinoid neuroendocrine neoplasms
					complex mixed and stromal neoplasms - epithelial
					cystic, mucinous and serous neoplasms
					epithelial neoplasms, NOS

CONFIDENTIALITY
This document is the confidential and proprietary information of GRAIL, LLC. Except as required by federal, state, or local laws or regulations, this document may not be disclosed to others, in whole or in part without prior written permission of GRAIL, Inc.

CSO class	Description	Examples for included cancers	Examples for excluded cancers	Topography name	Morphology group
					fibroepithelial neoplasms without phyllodes
					medullary cancers
					mesonephromas
					specialized gonadal neoplasms
					squamous cell neoplasms
					transitional cell papillomas and carcinomas
					unspecified neoplasms
Pancreas, Gallbladder	Methylation signal associated with carcinomas of the exocrine and endocrine pancreas, gallbladder, and extrahepatic bile ducts (perihilar, distal, and cystic ducts), excluding high grade neuroendocrine tumors	Pancreatic Ductal Adenocarcinoma	Sarcoma or lymphoma reported in pancreas, gallbladder, or bile ducts	Gallbladder<br>Extrahepatic bile duct<br>Ampulla of Vater<br>Distal (extrahepatic) bile duct<br>Perihilar (or proximal) bile duct<br>Head of pancreas<br>Body pancreas<br>Tail pancreas<br>Pancreatic duct<br>Islets of Langerhans<br>Neck of pancreas	acinar cell neoplasms
		Gallbladder adenocarcinoma	Intrahepatic cholangiocarcinoma		adenocarcinomas
		Extrahepatic bile duct carcinoma			carcinoid neuroendocrine neoplasms
		Cystic duct cholangiocarcinoma			complex epithelial neoplasms - adenocarcinoma
		Pancreatic neuroendocrine tumor			complex epithelial neoplasms - all other
					complex mixed and stromal neoplasms -

CONFIDENTIALITY
This document is the confidential and proprietary information of GRAIL, LLC. Except as required by federal, state, or local laws or regulations, this document may not be disclosed to others, in whole or in part without prior written permission of GRAIL, Inc.

CSO class	Description	Examples for included cancers	Examples for excluded cancers	Topography name	Morphology group
				Overlapping lesion of pancreas<br>Pancreas NOS	epithelial
					cystic, mucinous and serous neoplasms
					ductal and lobular neoplasms
					epithelial neoplasms, NOS
					medullary cancers
					squamous cell neoplasms
					unspecified neoplasms
Plasma Cell Lineage	Methylation signal associated with blood, lymph nodes and bone marrow with a cell of origin with plasma cell lineage.	Multiple myeloma		any	plasma cell tumors
		Plasma cell myeloma			
Prostate	Methylation signal associated with carcinomas of the prostate, excluding high grade neuroendocrine tumors	Invasive ductal prostate adenocarcinoma	Sarcoma or lymphoma reported in the prostate	Prostate gland	acinar cell neoplasms
		Invasive acinar adenocarcinoma of the prostate	Small cell carcinomas of the prostate		adenocarcinomas
					carcinoid neuroendocrine neoplasms
					cystic, mucinous and serous neoplasms
					ductal and lobular

CONFIDENTIALITY
This document is the confidential and proprietary information of GRAIL, LLC. Except as required by federal, state, or local laws or regulations, this document may not be disclosed to others, in whole or in part without prior written permission of GRAIL, Inc.

CSO class	Description	Examples for included cancers	Examples for excluded cancers	Topography name	Morphology group
					neoplasms
					epithelial neoplasms, NOS
					medullary cancers
					squamous cell neoplasms
					unspecified neoplasms
Stomach, Esophagus	Methylation signal associated with carcinomas of the stomach or esophagus, excluding high grade neuroendocrine tumors	Gastric adenocarcinoma	Gastrointestinal stromal tumor GIST	Cervical esophagus<br>Thoracic esophagus<br>Abdominal esophagus<br>Upper third of esophagus<br>Middle third of esophagus<br>Esophagus lower third<br>Overlapping lesion of esophagus<br>Esophagus NOS<br>Cardia, NOS<br>Fundus stomach<br>Body stomach<br>Gastric antrum<br>Pylorus<br>Lesser curvature of stomach, NOS (not classifiable to	adenocarcinomas
		Esophageal adenocarcinoma	MALT lymphoma in stomach		carcinoid neuroendocrine neoplasms
		Esophageal squamous cell carcinoma	Cancers of the small intestines		complex epithelial neoplasms - adenocarcinoma
		Carcinoid in stomach	Small cell carcinoma in stomach		complex epithelial neoplasms - all other
					complex mixed and stromal neoplasms - epithelial
					cystic, mucinous and serous neoplasms
					ductal and lobular neoplasms
					epithelial neoplasms, NOS

CONFIDENTIALITY
This document is the confidential and proprietary information of GRAIL, LLC. Except as required by federal, state, or local laws or regulations, this document may not be disclosed to others, in whole or in part without prior written permission of GRAIL, Inc.

CSO class	Description	Examples for included cancers	Examples for excluded cancers	Topography name	Morphology group
				C16.1 to C16.4)<br>Greater curvature of stomach, NOS (not classifiable to C16.0 to C16.4)<br>Overlapping lesion of stomach<br>Stomach NOS	medullary cancers<br>squamous cell neoplasms<br>unspecified neoplasms
Thyroid	Methylation signal associated with carcinomas of the thyroid, excluding high grade neuroendocrine tumors	Medullary carcinoma of the thyroid<br>Papillary carcinoma of the thyroid	Sarcoma or lymphoma reported in the thyroid	Thyroid gland	adenocarcinomas<br>carcinoid neuroendocrine neoplasms<br>ductal and lobular neoplasms<br>epithelial neoplasms, NOS<br>medullary cancers<br>unspecified neoplasms
Uterus	Methylation signal associated with carcinomas of the endometrium, excluding high grade neuroendocrine tumors	Endometrial carcinoma<br>Carcinosarcoma of the uterus<br>High-grade serous cystadenocarcinoma reported with uterus as primary site	Germ cell tumors<br>Uterine sarcomas<br>Endometrial stromal tumors	Isthmus uteri<br>Endometrium<br>Myometrium<br>Fundus uteri<br>Overlapping lesion of corpus uteri<br>Corpus uteri<br>Uterus NOS	adenocarcinomas<br>carcinoid neuroendocrine neoplasms<br>complex epithelial neoplasms - adenocarcinoma<br>complex epithelial neoplasms - all other<br>complex mixed and stromal neoplasms - epithelial

CONFIDENTIALITY
This document is the confidential and proprietary information of GRAIL, LLC. Except as required by federal, state, or local laws or regulations, this document may not be disclosed to others, in whole or in part without prior written permission of GRAIL, Inc.

CSO class	Description	Examples for included cancers	Examples for excluded cancers	Topography name	Morphology group
					cystic, mucinous and serous neoplasms
					epithelial neoplasms, NOS
					medullary cancers
					squamous cell neoplasms
					unspecified neoplasms

CONFIDENTIALITY
This document is the confidential and proprietary information of GRAIL, LLC. Except as required by federal, state, or local laws or regulations, this document may not be disclosed to others, in whole or in part without prior written permission of GRAIL, Inc.

APPENDIX 4A CSO PREDICTIONS FOR THE MCED-I TEST

Note: any changes to the displayed names will be documented in the clinical study report.

N	CSO predictions
1	Anus
2	Bladder, Urothelial tract
3	Hematopoietic and Lymphoid Organs
4	Bone and Soft tissue
5	Breast
6	Cervix
7	Colon, Rectum
8	Head and Neck
9	Kidney (excluding renal pelvis)
10	Liver, Bile Duct
11	Lung
12	Ovary
13	Pancreas, Gallbladder
14	Prostate
15	Melanocyte-containing Tissues/Skin
16	Stomach, Esophagus
17	Thyroid
18	Uterus

CONFIDENTIALITY
This document is the confidential and proprietary information of GRAIL, LLC. Except as required by federal, state, or local laws or regulations, this document may not be disclosed to others, in whole or in part without prior written permission of GRAIL, Inc.

Appendix 4B CBS Predictions for MCED-I Test

Note: any changes to the displayed names will be documented in the clinical study report.

N	CBS predictions
1	Squamous Cell Signal of Head and Neck, Lung and Esophagus
2	Human Papillomavirus (HPV) Associated Methylation Signal
3	Female Reproductive Tract Signal
4	Neuroendocrine Signal
5	Lymphoid Lineage
6	Myeloid Lineage
7	Plasma Cell Lineage

CONFIDENTIALITY

This document is the confidential and proprietary information of GRAIL, LLC. Except as required by federal, state, or local laws or regulations, this document may not be disclosed to others, in whole or in part without prior written permission of GRAIL, Inc.

Appendix 5A Crosstabulation of MCED-I and MCED-V2 CSO Predictions

N	MCED-I CSO	MCED-V2 CSO
1	Anus	Anus
2	Bladder, Urothelial tract	Bladder, Urothelial Tract
3	Hematopoietic and Lymphoid Organs	Lymphoid Lineage
		Myeloid Lineage
		Plasma Cell Lineage
4	Bone, Soft Tissue	Bone and Soft Tissue
5	Breast	Breast
6	Cervix	Cervix
7	Colon, Rectum	Colon, Rectum
8	Head and Neck	Head and Neck
9	Kidney (excluding renal pelvis)	Kidney
10	Liver, Bile Duct	Liver, Bile Duct
11	Lung	Lung
12	Ovary	Ovary
13	Pancreas, Gallbladder	Pancreas, Gallbladder
14	Prostate	Prostate
15	Melanocyte-containing Tissues/Skin	Melanocytic Lineage
16	Stomach, Esophagus	Stomach, Esophagus
17	Thyroid	Thyroid Gland
18	Uterus	Uterus

Note: MCED-V2 has an additional CSO of "Neuroendocrine Cells of Lung or other Organs" which has no corresponding MCED-I CSO. Instead, the classification algorithm will redistribute cases with this MCED-V2 CSO to MCED-I CSO for each respective organ or organ system affected by cancer.

CONFIDENTIALITY
This document is the confidential and proprietary information of GRAIL, LLC. Except as required by federal, state, or local laws or regulations, this document may not be disclosed to others, in whole or in part without prior written permission of GRAIL, Inc.

Appendix 5B: Matrix of MCED-I CSO Predictions and Corresponding MCED-V2 CSO Predictions among test positives for both tests

MCED-I CSO	MCED-V2 CSO1																				
	Anus	Bladder, Urothelial tract	Lymphoid Lineage	Myeloid Lineage	Plasma Cell Lineage	Bone and Soft Tissue	Breast	Cervix	Colon, rectum	Head, neck	Kidney	Liver, Intrahepatic bile duct	Lung	Neuroendocrine	Ovary	Pancreas, Gallbladder	Prostate	Melanocytic Lineage	Stomach, Esophagus	Thyroid	Uterus
Anus																					
Bladder, Urothelial tract																					
Hematopoietic and Lymphoid Organs																					
Bone and Soft Tissue																					
Breast																					
Cervix																					
Colon, Rectum																					
Head and Neck																					
Kidney (excluding renal pelvis)																					
Liver, Bile Duct																					
Lung																					
Ovary																					
Pancreas, Gallbladder																					
Prostate																					
Melanocyte-containing Tissues/Skin																					
Stomach, Esophagus																					

CONFIDENTIALITY

This document is the confidential and proprietary information of GRAIL, LLC. Except as required by federal, state, or local laws or regulations, this document may not be disclosed to others, in whole or in part without prior written permission of GRAIL, Inc.

MCED-I CSO	MCED-V2 CSO1																				
	Anus	Bladder, Urothelial tract	Lymphoid Lineage	Myeloid Lineage	Plasma Cell Lineage	Bone and Soft Tissue	Breast	Cervix	Colon, rectum	Head, neck	Kidney	Liver, Intrahepatic bile duct	Lung	Neuroendocrine	Ovary	Pancreas, Gallbladder	Prostate	Melanocytic Lineage	Stomach, Esophagus	Thyroid	Uterus
Thyroid																					
Uterus																					

Note: MCED-V2 CSO of “Neuroendocrine Cells of Lung or other Organs” has no direct corresponding MCED-I CSO. Instead, the MCED-I classification algorithm will seek to predict the organ or organ system affected by neuroendocrine cancers.

CONFIDENTIALITY
This document is the confidential and proprietary information of GRAIL, LLC. Except as required by federal, state, or local laws or regulations, this document may not be disclosed to others, in whole or in part without prior written permission of GRAIL, Inc.

Appendix 6A MCED-I CSO Specification

CSO class	Description	Examples for included cancers	Examples for excluded cancers	Topography name	Morphology group
Anus	Methylation signal associated with carcinomas of the anus and anal canal. The main contributor to this class is HPV-associated squamous cell carcinoma of the anus.	HPV-associated squamous cell carcinoma of the anus	Skin cancers reported in the anal region	Rectosigmoid junction	squamous cell neoplasms
		Anal gland adenocarcinoma	Extramammary Paget disease in this region		
		HPV-positive squamous cell carcinoma reported with primary site rectum	Squamous cell carcinoma reported in the colon, even if HPV-positive		
			Sarcoma or lymphoma reported in the anal region	Rectum, NOS	squamous cell neoplasms
				Anus, NOS (excludes Skin of anus and Perianal skin (C44.5)<br>Anal canal<br>Cloacogenic zone<br>Overlapping lesion of rectum, anus and anal canal	adenocarcinomas
					epithelial neoplasms, NOS
					medullary cancers
					squamous cell neoplasms
Bladder or Urothelial	Methylation signal associated with carcinomas of the bladder, ureter, urethra and renal pelvis.	Adenocarcinoma of the bladder	Renal cell carcinomas reported at the renal pelvis as anatomic site	Renal pelvis<br>Ureter<br>Trigone, bladder<br>Dome, bladder<br>Lateral wall bladder<br>Posterior wall bladder	adenocarcinomas
		Urothelial carcinoma of the bladder	Sarcoma or lymphoma reported in the bladder, ureter or renal pelvis		complex epithelial neoplasms - adenocarcinoma
		Urothelial			complex epithelial

CONFIDENTIALITY

This document is the confidential and proprietary information of GRAIL, LLC. Except as required by federal, state, or local laws or regulations, this document may not be disclosed to others, in whole or in part without prior written permission of GRAIL, Inc.

CSO class	Description	Examples for included cancers	Examples for excluded cancers	Topography name	Morphology group
		carcinoma of the renal pelvis		Ureteric orifice<br>Urachus<br>Overlapping lesion of bladder<br>Bladder NOS<br>Urethra	neoplasms - all other
		Urothelial carcinoma of the ureter			cystic, mucinous and serous neoplasms
		Small cell carcinoma of the bladder			epithelial neoplasms, NOS
		Adenocarcinoma NOS in the renal pelvis			medullary cancers
		Urothelial carcinoma in the kidney			neuroendocrine neoplasms
					squamous cell neoplasms
					transitional cell papillomas and carcinomas
					unspecified neoplasms
				Kidney NOS	transitional cell papillomas and carcinomas
Blood, Lymphatic system or Bone marrow	Methylation signal associated with cancers of the blood, the lymphatic system, or bone marrow of lymphoid, myeloid, or plasma cell origin.	Lymphomas, including CNS lymphomas and MALT lymphomas of the GI tract		any	chronic myeloproliferative disorders (C42.1)
		Lymphoid leukemia			hodgkin lymphomas
		Myeloid leukemia			immunoproliferative diseases

CONFIDENTIALITY
This document is the confidential and proprietary information of GRAIL, LLC. Except as required by federal, state, or local laws or regulations, this document may not be disclosed to others, in whole or in part without prior written permission of GRAIL, Inc.

CSO class	Description	Examples for included cancers	Examples for excluded cancers	Topography name	Morphology group
		Multiple myeloma or plasma cell myeloma			leukemias, NOS
					lymphoid leukemia/lymphoma
					lymphoid leukemias (C42.1)
					malignant lymphomas, NOS or diffuse
					mast cell tumors
					myelodysplastic syndrome
					myeloid leukemias (C42.1)
					neoplasm of histiocytes and accessory lymphoid cells
					non-hodgkin lymphoma - mature b-cell lymphomas
					non-hodgkin lymphoma - mature t and nk-cell lymphomas
					non-hodgkin lymphoma - precursor cell lymphoblastic lymphoma
					other leukemias (C42.1)

CONFIDENTIALITY
This document is the confidential and proprietary information of GRAIL, LLC. Except as required by federal, state, or local laws or regulations, this document may not be disclosed to others, in whole or in part without prior written permission of GRAIL, Inc.

CSO class	Description	Examples for included cancers	Examples for excluded cancers	Topography name	Morphology group
					plasma cell tumors
Breast	Methylation signal associated with carcinomas of the breast.	Invasive ductal breast carcinoma	Sarcoma or lymphoma reported in the breast	Nipple<br>Central portion of breast<br>Upper inner quadrant of breast<br>Lower inner quadrant of breast<br>Upper outer quadrant of breast<br>Lower outer quadrant of breast<br>Axillary tail of breast<br>Overlapping lesion of breast<br>Breast NOS (excludes Skin of breast C44.5)	acinar cell neoplasms
		Invasive lobular breast carcinoma	Any invasive skin cancer of the breast		adenocarcinomas
		Inflammatory carcinoma			complex epithelial neoplasms - adenocarcinoma
					complex epithelial neoplasms - all other
					cystic, mucinous and serous neoplasms
					ductal and lobular neoplasms
					epithelial neoplasms, NOS
					medullary cancers
					neuroendocrine neoplasms
					squamous cell neoplasms
					unspecified neoplasms
Cervix	Methylation signal associated with carcinomas of the cervix. The main contributor to this	HPV-associated squamous cell carcinoma of the cervix		Endocervix<br>Exocervix<br>Cervix uteri	adenocarcinomas

CONFIDENTIALITY
This document is the confidential and proprietary information of GRAIL, LLC. Except as required by federal, state, or local laws or regulations, this document may not be disclosed to others, in whole or in part without prior written permission of GRAIL, Inc.

CSO class	Description	Examples for included cancers	Examples for excluded cancers	Topography name	Morphology group
	class is HPV-associated squamous cell carcinoma of the cervix.	HPV-associated adenocarcinoma of the cervix		Overlapping lesion of cervix uteri	adenoid basal
		Neuroendocrine carcinoma of the cervix			complex epithelial neoplasms - adenocarcinoma
		Non-HPV-associated adenocarcinoma of the cervix			complex epithelial neoplasms - all other
					cystic, mucinous and serous neoplasms
					epithelial neoplasms, NOS
					medullary cancers
					mesonephromas
					neuroendocrine neoplasms
					squamous cell neoplasms
					transitional cell papillomas and carcinomas
					unspecified neoplasms
Colon or Rectum	Methylation signal associated with carcinomas of the colon, rectum and appendix.	Colorectal adenocarcinoma	HPV-positive squamous cell carcinoma reported in the rectum	Cecum<br>Appendix<br>Ascending colon<br>Hepatic flexure of colon	adenocarcinomas
		Signet ring cell carcinoma of	Sarcoma or lymphoma		complex epithelial neoplasms -

CONFIDENTIALITY
This document is the confidential and proprietary information of GRAIL, LLC. Except as required by federal, state, or local laws or regulations, this document may not be disclosed to others, in whole or in part without prior written permission of GRAIL, Inc.

CSO class	Description	Examples for included cancers	Examples for excluded cancers	Topography name	Morphology group
		colon	reported in colon or rectum	Transverse colon<br>Splenic flexure of colon<br>Descending colon<br>Sigmoid colon<br>Overlapping lesion of colon<br>Colon NOS	adenocarcinoma
		Large cell neuroendocrine carcinoma in colon	Adenocarcinoma or carcinoid of the small intestines		complex epithelial neoplasms - all other
		Carcinoid in colon			cystic, mucinous and serous neoplasms
		Adenocarcinoma in the appendix			ductal and lobular neoplasms
		Carcinoid of the appendix			epithelial neoplasms, NOS
		Squamous cell carcinoma in colon			medullary cancers
					neuroendocrine neoplasms
					squamous cell neoplasms
					unspecified neoplasms
				Rectosigmoid junction<br>Rectum, NOS	adenocarcinomas
					complex epithelial neoplasms - adenocarcinoma
					complex epithelial neoplasms - all other
					cystic, mucinous and serous neoplasms
					ductal and lobular

CONFIDENTIALITY
This document is the confidential and proprietary information of GRAIL, LLC. Except as required by federal, state, or local laws or regulations, this document may not be disclosed to others, in whole or in part without prior written permission of GRAIL, Inc.

CSO class	Description	Examples for included cancers	Examples for excluded cancers	Topography name	Morphology group
					neoplasms
					epithelial neoplasms, NOS
					neuroendocrine neoplasms
					unspecified neoplasms
Head and Neck	Methylation signal associated with HPV-associated and HPV-negative carcinomas of the head and neck region including oropharynx, hypopharynx, nasopharynx, larynx, lip and oral cavity (including oral tongue), nasal cavity, paranasal sinuses and major salivary glands.	Oropharyngeal HPV-associated squamous cell carcinoma	Sarcoma or lymphoma reported in the H&N region	Upper lip, mucosa<br>Lower lip, mucosa<br>Mucosa lip, NOS<br>Base of tongue, NOS<br>Dorsal surface tongue, NOS<br>Border of tongue<br>Ventral surface of tongue NOS<br>Anterior 2/3 of tongue NOS<br>Lingual tonsil<br>Overlapping lesion of tongue<br>Tongue NOS<br>Upper gum<br>Lower gum<br>Gum NOS<br>Anterior floor of mouth<br>Lateral floor of mouth	acinar cell neoplasms
		HPV-negative squamous cell carcinoma of the larynx	Skin cancers reported in the H&N region		adenocarcinomas
		Salivary gland adenocarcinoma			complex epithelial neoplasms - adenocarcinoma
					complex epithelial neoplasms - all other
					complex mixed and stromal neoplasms - epithelial
					ductal and lobular neoplasms
					epithelial neoplasms, NOS
					medullary cancers
					mucoepidermoid neoplasms

CONFIDENTIALITY
This document is the confidential and proprietary information of GRAIL, LLC. Except as required by federal, state, or local laws or regulations, this document may not be disclosed to others, in whole or in part without prior written permission of GRAIL, Inc.

CSO class	Description	Examples for included cancers	Examples for excluded cancers	Topography name	Morphology group
				Floor of mouth NOS<br>Hard palate<br>Soft palate NOS (excludes Nasopharyngeal surface C11.3)<br>Uvula<br>Palate NOS<br>Cheek mucosa<br>Vestibule of mouth<br>Retromolar area<br>Overlapping lesion of other and unspecified parts of mouth<br>Mouth NOS<br>Parotid gland<br>Submaxillary gland<br>Sublingual gland<br>Major salivary gland, NOS<br>Tonsillar fossa<br>Tonsillar pillar<br>Overlapping lesion of tonsil<br>Tonsil NOS (excludes Lingual tonsil C02.4 and Pharyngeal tonsil C11.1)	neuroendocrine neoplasms<br>squamous cell neoplasms<br>unspecified neoplasms

CONFIDENTIALITY
This document is the confidential and proprietary information of GRAIL, LLC. Except as required by federal, state, or local laws or regulations, this document may not be disclosed to others, in whole or in part without prior written permission of GRAIL, Inc.

CSO class	Description	Examples for included cancers	Examples for excluded cancers	Topography name	Morphology group
				Vallecula<br>Anterior surface of epiglottis<br>Lateral wall oropharynx<br>Posterior wall oropharynx<br>Branchial cleft (site of neoplasm)<br>Overlapping lesion of oropharynx<br>Oropharynx NOS<br>Superior wall of nasopharynx<br>Posterior wall nasopharynx<br>Lateral wall nasopharynx<br>Anterior wall nasopharynx<br>Overlapping lesion of nasopharynx<br>Nasopharynx NOS<br>Pyriform sinus<br>Postcricoid region<br>Hypopharyngeal aspect of aryepiglottic fold<br>Posterior wall hypopharynx<br>Hypopharynx, NOS	

CONFIDENTIALITY
This document is the confidential and proprietary information of GRAIL, LLC. Except as required by federal, state, or local laws or regulations, this document may not be disclosed to others, in whole or in part without prior written permission of GRAIL, Inc.

CSO class	Description	Examples for included cancers	Examples for excluded cancers	Topography name	Morphology group
				Pharynx NOS<br>Nasal cavity (excludes Nose, NOS C76.0)<br>Maxillary sinus<br>Ethmoid sinus<br>Glottis<br>Supraglottis<br>Subglottis<br>Larynx NOS<br>Overlapping lesion of floor of mouth<br>Overlapping lesion of palate<br>Overlapping lesion of major salivary glands<br>Overlapping lesion of hypopharynx	
				Head, face or neck NOS	squamous cell neoplasms
Kidney	Methylation signal associated with carcinomas of the renal cortex and renal tubules.	Renal cell carcinoma	Sarcoma or lymphoma reported in the kidney	Kidney NOS	adenocarcinomas
		Carcinoid in kidney	Transitional or urothelial carcinomas reported in the kidney		complex mixed and stromal neoplasms - epithelial
		Adenocarcinoma NOS in the kidney			epithelial neoplasms, NOS
					medullary cancers

CONFIDENTIALITY
This document is the confidential and proprietary information of GRAIL, LLC. Except as required by federal, state, or local laws or regulations, this document may not be disclosed to others, in whole or in part without prior written permission of GRAIL, Inc.

CSO class	Description	Examples for included cancers	Examples for excluded cancers	Topography name	Morphology group
					neuroendocrine neoplasms
					renal cell carcinomas
					unspecified neoplasms
				Renal pelvis	renal cell carcinomas
Liver or Intrahepatic bile duct	Methylation signal associated with carcinomas of the liver and intrahepatic bile duct.	Hepatocellular carcinoma	Carcinoma of the perihilar bile duct	Liver<br>Intrahepatic bile duct	adenocarcinomas
		Intrahepatic cholangiocarcinoma	Sarcoma or lymphoma reported in the liver		combined hepatocellular carcinoma (HCC) / intrahepatic cholangiocarcinoma (iCC)
		Small cell carcinoma in the liver			epithelial neoplasms, NOS
		Carcinoid in the liver			hepatocellular carcinomas
					medullary cancers
					neuroendocrine neoplasms
					squamous cell neoplasms
					unspecified neoplasms
Lung	Methylation signal associated with carcinomas of the lung and	Lung adenocarcinoma	Sarcoma or lymphoma reported in the	Main bronchus<br>Upper lobe, lung	acinar cell neoplasms

CONFIDENTIALITY
This document is the confidential and proprietary information of GRAIL, LLC. Except as required by federal, state, or local laws or regulations, this document may not be disclosed to others, in whole or in part without prior written permission of GRAIL, Inc.

CSO class	Description	Examples for included cancers	Examples for excluded cancers	Topography name	Morphology group
	bronchus.	Lung squamous cell carcinoma	lung	Middle lobe, lung<br>Lower lobe, lung<br>Overlapping lesion of lung<br>Lung NOS	adenocarcinomas
		Non Small Cell Lung Cancer (NSCLC) NOS			complex epithelial neoplasms - adenocarcinoma
		Small Cell Lung Cancer (SCLC)			complex epithelial neoplasms - all other
		Carcinoid in the lung			complex mixed and stromal neoplasms - epithelial
					cystic, mucinous and serous neoplasms
					epithelial neoplasms, NOS
					medullary cancers
					mucoepidermoid neoplasms
					neuroendocrine neoplasms
					squamous cell neoplasms
					unspecified neoplasms
Ovary or Fallopia n Tubes	Methylation signal associated with carcinomas and other solid tumors with cell of origin from the ovary, fallopian tubes or primary	Fallopian tube-derived serous cystadenocarcino ma of the ovaries	Germ cell tumors	Peritoneum<br>Peritoneum NOS<br>Overlapping lesion of retroperitoneum and peritoneum	adenocarcinomas
		Endometrioid	Sarcoma or lymphoma		complex mixed and stromal neoplasms

CONFIDENTIALITY
This document is the confidential and proprietary information of GRAIL, LLC. Except as required by federal, state, or local laws or regulations, this document may not be disclosed to others, in whole or in part without prior written permission of GRAIL, Inc.

CSO class	Description	Examples for included cancers	Examples for excluded cancers	Topography name	Morphology group
	peritoneum.	ovarian carcinoma	reported in the ovaries or peritoneal region		- epithelial
		Malignant mullerian mixed carcinoma of the ovaries	Mesothelioma in the peritoneal region		cystic, mucinous and serous neoplasms
		Clear cell carcinoma of the ovaries			epithelial neoplasms, NOS
		Small cell carcinoma of the ovaries			fibroepithelial neoplasms without phyllodes
		Malignant Brenner tumor			medullary cancers
					mesonephromas
					neuroendocrine neoplasms
					specialized gonadal neoplasms
					squamous cell neoplasms
					unspecified neoplasms
				Ovary<br>Fallopian tube	adenocarcinomas
					complex mixed and stromal neoplasms - epithelial
					cystic, mucinous and serous neoplasms
					epithelial

CONFIDENTIALITY
This document is the confidential and proprietary information of GRAIL, LLC. Except as required by federal, state, or local laws or regulations, this document may not be disclosed to others, in whole or in part without prior written permission of GRAIL, Inc.

CSO class	Description	Examples for included cancers	Examples for excluded cancers	Topography name	Morphology group
					neoplasms, NOS
					fibroepithelial neoplasms without phyllodes
					medullary cancers
					mesonephromas
					neuroendocrine neoplasms
					specialized gonadal neoplasms
					squamous cell neoplasms
					transitional cell papillomas and carcinomas
					unspecified neoplasms
Pancreas, Extrahepatic bile duct, or Gallbladder	Methylation signal associated with carcinomas of the exocrine and endocrine pancreas, gallbladder, and extrahepatic bile ducts (perihilar, distal, and cystic ducts).	Pancreatic Ductal Adenocarcinoma	Sarcoma or lymphoma reported in pancreas, gallbladder, or bile ducts	Gallbladder<br>Extrahepatic bile duct<br>Ampulla of Vater<br>Distal (extrahepatic) bile duct<br>Perihilar (or proximal) bile duct<br>Head of pancreas<br>Body pancreas<br>Tail pancreas	acinar cell neoplasms
		Gallbladder adenocarcinoma	Intrahepatic cholangiocarcinoma		adenocarcinomas
		Extrahepatic bile duct carcinoma			complex epithelial neoplasms - adenocarcinoma
		Cystic duct cholangiocarcinoma			complex epithelial neoplasms - all other

CONFIDENTIALITY
This document is the confidential and proprietary information of GRAIL, LLC. Except as required by federal, state, or local laws or regulations, this document may not be disclosed to others, in whole or in part without prior written permission of GRAIL, Inc.

CSO class	Description	Examples for included cancers	Examples for excluded cancers	Topography name	Morphology group
		Pancreatic neuroendocrine tumor		Pancreatic duct<br>Islets of Langerhans<br>Neck of pancreas<br>Overlapping lesion of pancreas<br>Pancreas NOS	complex mixed and stromal neoplasms - epithelial
					cystic, mucinous and serous neoplasms
					ductal and lobular neoplasms
					epithelial neoplasms, NOS
					medullary cancers
					neuroendocrine neoplasms
					squamous cell neoplasms
					unspecified neoplasms
Prostate	Methylation signal associated with carcinomas of the prostate.	Invasive ductal prostate adenocarcinoma	Sarcoma or lymphoma reported in the prostate	Prostate gland	acinar cell neoplasms
		Invasive adenocarcinoma of the prostate			adenocarcinomas
		Small cell carcinomas of the prostate			cystic, mucinous and serous neoplasms
					ductal and lobular neoplasms
					epithelial neoplasms, NOS

CONFIDENTIALITY
This document is the confidential and proprietary information of GRAIL, LLC. Except as required by federal, state, or local laws or regulations, this document may not be disclosed to others, in whole or in part without prior written permission of GRAIL, Inc.

CSO class	Description	Examples for included cancers	Examples for excluded cancers	Topography name	Morphology group
					medullary cancers
					neuroendocrine neoplasms
					unspecified neoplasms
Sarcoma	Methylation signal associated with sarcomas and other cancers of mesenchymal origin in bone and soft tissue independent from anatomic site	Uterine leiomyosarcoma	Myeloid Sarcoma	any	blood vessel tumors
		Malignant solitary fibrous tumor			chordomas
		Osteosarcoma			complex mixed and stromal neoplasms - sarcoma
		Gastrointestinal stromal tumor			fibromatous neoplasms
		Malignant fibrous histiocytoma (of skin)			giant cell tumors
		Sarcomas of the limbs, chest, or retroperitoneum			glomus tumors
		Malignant Hemangiopericytoma in the brain			lipomatous neoplasms
					lymphatic vessel tumors
					miscellaneous bone tumors (C40._, C41._)
					myomatous neoplasms
					myxomatous

CONFIDENTIALITY
This document is the confidential and proprietary information of GRAIL, LLC. Except as required by federal, state, or local laws or regulations, this document may not be disclosed to others, in whole or in part without prior written permission of GRAIL, Inc.

CSO class	Description	Examples for included cancers	Examples for excluded cancers	Topography name	Morphology group
					neoplasms
					nerve sheath tumors
					osseous and chondromatous neoplasms
					phyllodes
					soft tissue tumors and sarcomas, NOS
					synovial-like neoplasms
				Skull and facial bone<br>Connective tissue head<br>Connective tissue arm<br>Connective tissue leg<br>Connective tissue thorax (excludes Thymus, Heart and Mediastinum C37. , C38.)<br>Connective tissue abdomen<br>Connective tissue pelvis<br>Connective tissue trunk, NOS<br>Overlapping lesion of connective,	unspecified neoplasms

CONFIDENTIALITY
This document is the confidential and proprietary information of GRAIL, LLC. Except as required by federal, state, or local laws or regulations, this document may not be disclosed to others, in whole or in part without prior written permission of GRAIL, Inc.

CSO class	Description	Examples for included cancers	Examples for excluded cancers	Topography name	Morphology group
				subcutaneous and other soft tissues<br>Connective tissue NOS	
Skin	Methylation signal associated with cancers of the skin. These are predominantly melanomas. Other skin cancers include sweat gland carcinomas and Merkel cell carcinomas. Basal and squamous cell carcinomas that have not metastasized are not included in this class.	Melanoma of extremities	Basal cell carcinoma of skin (unless metastatic)	Skin lip, NOS<br>Eyelid NOS<br>External ear<br>Skin face<br>Skin scalp, neck<br>Skin trunk<br>Skin limb, upper<br>Skin limb, lower	adnexal and skin appendage neoplasms
		Melanoma in the H&N region	Squamous cell carcinoma of skin (unless metastatic)		basal cell neoplasms (only stages III and IV)
		Merkel cell carcinoma	Cutaneous lymphomas		mucinous adenocarcinoma
		Melanoma of mucosal tissue			neuroendocrine neoplasms
		Melanoma of the eye (uveal melanoma)			squamous cell neoplasms (only stages III and IV)
		Digital papillary adenocarcinoma of sweat glands			unspecified neoplasms
				External lip upper<br>External lip lower<br>External lip NOS<br>Commissure lip<br>Upper gum<br>Anus, NOS (excludes Skin of anus and Perianal skin (C44.5)<br>Anal canal<br>Nasal cavity	nevi and melanomas

CONFIDENTIALITY

This document is the confidential and proprietary information of GRAIL, LLC. Except as required by federal, state, or local laws or regulations, this document may not be disclosed to others, in whole or in part without prior written permission of GRAIL, Inc.

CSO class	Description	Examples for included cancers	Examples for excluded cancers	Topography name	Morphology group
				(excludes Nose, NOS C76.0)<br>Maxillary sinus<br>Ethmoid sinus<br>Skin lip, NOS<br>Eyelid NOS<br>External ear<br>Skin face<br>Skin scalp, neck<br>Skin trunk<br>Skin limb, upper<br>Skin limb, lower<br>Labium majus<br>Labium minus<br>Clitoris<br>Vulva, NOS<br>Vagina, NOS<br>Prepuce<br>Glans penis<br>Body penis<br>Penis NOS<br>Scrotum, NOS<br>Conjunctiva<br>Retina<br>Choroid<br>Ciliary body	
Stomach or	Methylation signal associated with	Gastric adenocarcinoma	Gastrointestinal stromal tumor	Cervical esophagus	adenocarcinomas

CONFIDENTIALITY
This document is the confidential and proprietary information of GRAIL, LLC. Except as required by federal, state, or local laws or regulations, this document may not be disclosed to others, in whole or in part without prior written permission of GRAIL, Inc.

CSO class	Description	Examples for included cancers	Examples for excluded cancers	Topography name	Morphology group
Esophagus	carcinomas of the stomach or esophagus.		GIST	Thoracic esophagus<br>Abdominal esophagus<br>Upper third of esophagus<br>Middle third of esophagus<br>Esophagus lower third<br>Overlapping lesion of esophagus<br>Esophagus NOS<br>Cardia, NOS<br>Fundus stomach<br>Body stomach<br>Gastric antrum<br>Pylorus<br>Lesser curvature of stomach, NOS (not classifiable to C16.1 to C16.4)<br>Greater curvature of stomach, NOS (not classifiable to C16.0 to C16.4)<br>Overlapping lesion of stomach<br>Stomach NOS	
		Esophageal adenocarcinoma	MALT lymphoma in stomach		complex epithelial neoplasms - adenocarcinoma
		Esophageal squamous cell carcinoma	Cancers of the small intestines		complex epithelial neoplasms - all other
		Small cell carcinoma in stomach			complex mixed and stromal neoplasms - epithelial
		Carcinoid in stomach			cystic, mucinous and serous neoplasms
					ductal and lobular neoplasms
					epithelial neoplasms, NOS
					medullary cancers
					neuroendocrine neoplasms
					squamous cell neoplasms
					unspecified neoplasms
Thyroid	Methylation signal associated with carcinomas of the thyroid.	Medullary carcinoma of the thyroid	Sarcoma or lymphoma reported in the thyroid	Thyroid gland	adenocarcinomas
		Papillary			ductal and lobular

CONFIDENTIALITY
This document is the confidential and proprietary information of GRAIL, LLC. Except as required by federal, state, or local laws or regulations, this document may not be disclosed to others, in whole or in part without prior written permission of GRAIL, Inc.

CSO class	Description	Examples for included cancers	Examples for excluded cancers	Topography name	Morphology group
		carcinoma of the thyroid			neoplasms
					epithelial neoplasms, NOS
					medullary cancers
					neuroendocrine neoplasms
					unspecified neoplasms
Uterus	Methylation signal associated with carcinomas of the endometrium.	Endometrial carcinoma	Germ cell tumors	Isthmus uteri<br>Endometrium<br>Myometrium<br>Fundus uteri<br>Overlapping lesion of corpus uteri<br>Corpus uteri<br>Uterus NOS	adenocarcinomas
		Carcinosarcoma of the uterus	Uterine sarcomas		complex epithelial neoplasms - adenocarcinoma
		High-grade serous cystadenocarcinoma reported with uterus as primary site	Endometrial stromal tumors		complex epithelial neoplasms - all other
					complex mixed and stromal neoplasms - epithelial
					cystic, mucinous and serous neoplasms
					epithelial neoplasms, NOS
					medullary cancers
					neuroendocrine neoplasms
					squamous cell neoplasms
					unspecified neoplasms

CONFIDENTIALITY
This document is the confidential and proprietary information of GRAIL, LLC. Except as required by federal, state, or local laws or regulations, this document may not be disclosed to others, in whole or in part without prior written permission of GRAIL, Inc.

APPENDIX 6B MCED-I CBS SPECIFICATION

CBS class	Description	Examples for included cancers	Examples for excluded cancers
Female Reproductive Tract	Methylation signals associated with cancers arising from cells of the female reproductive tract progenitors; the Müllerian ducts including ovary, fallopian tubes, uterus and cervix.	Ovarian serous cystadenocarcinoma	HPV-positive squamous cell carcinomas of the cervix
		Fallopian tube-derived serous cystadenocarcinoma of the ovaries	Cancers reported in ovary or uterus from a different cellular lineage (for example uterine leiomyosarcomas)
		Endometrioid adenocarcinoma of ovary or uterus	
		Clear cell carcinoma of ovary or uterus	
		Malignant Mullerian Mixed tumor	
		Carcinosarcoma of the uterus	
		HPV-negative adenocarcinoma of the cervix	
HPV-associated Methylation Signal	Methylation signal associated with carcinomas associated with the Human Papilloma Virus (HPV)	HPV-positive oropharyngeal squamous cell carcinomas	HPV-negative squamous cell carcinomas of the larynx
		HPV-positive SCC of the cervix	Squamous cell carcinomas in the H&N region without clinical or molecular evidence of HPV status
		HPV-positive SCC of the anus	Squamous cell carcinomas in the cervix or anus with a conclusive negative test result for HPV status
		HPV-positive adenocarcinomas of the cervix	Squamous cell carcinoma of the skin in the anal region
		HPV-positive SCC of the penis, vulva or vagina	Non HPV-associated cancers in a patient with an active HPV infection
Lymphoid neoplasm	Methylation signal associated with malignancies of lymphoid lineage excluding plasma cells.	Hodgkin and non-hodgkin lymphoma	Plasma Cell Neoplasms
		T-cell lymphoma	
		Chronic Lymphocytic Leukemia (CLL)/ Small Lymphocytic Lymphoma (SLL)	

CONFIDENTIALITY
This document is the confidential and proprietary information of GRAIL, LLC. Except as required by federal, state, or local laws or regulations, this document may not be disclosed to others, in whole or in part without prior written permission of GRAIL, Inc.

CBS class	Description	Examples for included cancers	Examples for excluded cancers
		Primary cutaneous follicle center lymphoma	
		Precursor B and T lymphoblastic leukemia	
		Gastric mucosa-associated lymphoid tissue lymphoma	
		Waldenstrom macroglobulinemia	
Myeloid neoplasm	Methylation signal associated with malignancies of myeloid lineage	Acute Myeloid Leukemia (AML) and Chronic Myeloid Leukemia (CML)	
		Myelodysplastic Syndrome (MDS)	
		Malignant mastocytosis	
		Myeloid sarcoma	
		Acute erythroid leukemia	
		Essential thrombocythemia	
		Primary Myelofibrosis	
		Polycythemia vera	
Neuroendocrine carcinoma or tumor	Methylation signals associated with neuroendocrine tumors or carcinomas from all organs and body regions.	Small Cell Lung Cancer (SCLC)	Adenocarcinoma with neuroendocrine differentiation
		Small cell carcinoma in the prostate	Mixed neuroendocrine non-neuroendocrine neoplasms with low-grade neuroendocrine component
		Large cell neuroendocrine carcinoma in the colon	
		Medullary carcinoma of the thyroid	
		Typical and atypical carcinoids, both functional and non-functional	
		Neuroendocrine carcinomas, NOS	
		Pancreatic neuroendocrine tumors	
		Collision tumor of SCLC and adenocarcinoma in the lung or combined small cell carcinomas	

CONFIDENTIALITY
This document is the confidential and proprietary information of GRAIL, LLC. Except as required by federal, state, or local laws or regulations, this document may not be disclosed to others, in whole or in part without prior written permission of GRAIL, Inc.

CBS class	Description	Examples for included cancers	Examples for excluded cancers
Plasma cell neoplasm	Methylation signal associated with malignancies of plasma cell of origin	Multiple myeloma	
		Plasma cell myeloma	
Squamous Cell Signal of Head and Neck, Lung and Esophagus	Methylation signal associated with squamous cell carcinomas primarily of the lung, bronchus, head and neck and esophagus	Keratinizing and non-keratinizing squamous cell carcinoma of bronchus or lung	HPV-positive squamous cell carcinomas have the CSO label for HPV-associated cancers
		HPV-negative squamous cell carcinoma of the larynx	Squamous cell carcinoma if the CSO prediction for organ group is not one of Lung, or Esophagus, Head or Neck
		Squamous cell carcinoma of the esophagus	
		Squamous cell carcinoma of the salivary glands	

CONFIDENTIALITY
This document is the confidential and proprietary information of GRAIL, LLC. Except as required by federal, state, or local laws or regulations, this document may not be disclosed to others, in whole or in part without prior written permission of GRAIL, Inc.

APPENDIX 7 PRESPECIFIED CANCER SUBGROUPS

N	Cancer Type	Cancers Responsible for 2/3 of all Cancer Deaths in the US[1]	Most aggressive cancers with lowest 5-year survival	Cancers without Common Screening Options	Solid Cancers with Common Screening Options[2]	Solid Cancers without Common Screening Options	Hematologic Malignancies
1	Anus	X		X		X	
2	Bladder or Urothelial tract	X		X		X	
3	Bone or Soft tissue sarcoma			X		X	
4	Breast				X		
5	Cervix				X		
6	Colon or Rectum	X			X		
7	Esophagus	X	X	X		X	
8	Head and Neck	X		X		X	
9	Kidney			X		X	
10	Liver or Intrahepatic bile duct	X	X	X		X	
11	Lung	X	X	X		X	
12	Myeloid Lineage			X			X
13	Lymphoid Lineage	X		X			X
14	Ovary or Fallopian tube	X	X	X		X	
15	Pancreas, Extrahepatic bile duct or Gallbladder	X	X	X		X	
16	Plasma cell lineage	X		X			X
17	Prostate				X		
18	Skin (excluding BCC/SCC)			X		X	
19	Stomach	X	X	X		X	
20	Thyroid			X		X	
21	Uterus			X		X	
22	Other[3]			X		X	

[1] Data source for cancers responsible for ⅔ of all cancer deaths in the US: SEER*Stat Database: Mortality - All Causes of Death, Total US, 2018–2019, released June 2022, underlying data provided by National Center for Health Statistics.

CONFIDENTIALITY

This document is the confidential and proprietary information of GRAIL, LLC. Except as required by federal, state, or local laws or regulations, this document may not be disclosed to others, in whole or in part without prior written permission of GRAIL, Inc.

[2] Cancers with common screening options include cancers of the breast, cervix, colon or rectum and prostate. For these cancers, population-wide cancer screening is recommended by several medical societies, and there is an age-based A,B, or C recommendation for screening made by the USPSTF. Lung cancer is not included in this definition because screening recommendations apply to a subset of individuals who are at high risk for this cancer due to their smoking history. Source: https://www.uspreventiveservicestaskforce.org/uspstf/

[3] Cancers that are not included in the defined cancer types are reported under the "Other" category. Examples of such cancers are described in Appendix 8.

CONFIDENTIALITY

This document is the confidential and proprietary information of GRAIL, LLC. Except as required by federal, state, or local laws or regulations, this document may not be disclosed to others, in whole or in part without prior written permission of GRAIL, Inc.

Appendix 8 Cancer Type Specifications

Based on AJCC Cancer Staging Manual, 8th edition for solid cancer types and WHO guidance for hematologic malignancies

Cancer Type	Cancer Type Description
Anus	Anus
Bladder or Urothelial Tract	Urinary Bladder<br>Renal Pelvis and Ureter
Bone or Soft tissue sarcoma	Bone<br>Soft Tissue Sarcoma of the Head and Neck<br>Soft Tissue Sarcoma of the Trunk and Extremities<br>Soft Tissue Sarcoma of the Abdomen and Thoracic Visceral Organs<br>Gastrointestinal Stromal Tumors<br>Soft Tissue Sarcoma of the Retroperitoneum<br>Soft Tissue Sarcoma - Unusual Histologies and Sites<br>Corpus Uteri: Leiomyosarcoma, Endometrial Stromal Sarcoma and Adenosarcoma<br>Orbital Sarcoma<br>Subset: Brain and Spinal Cord Sarcomas (CNS Sarcomas [Mesenchymal, Non-meningothelial Tumors])
Breast	Breast
Cervix	Cervix Uteri
Colon or Rectum	Colon Rectum<br>Appendix - Carcinoma<br>Neuroendocrine Tumors of the Appendix<br>Neuroendocrine Tumors of the Colon and Rectum
Esophagus	Esophagus and Esophagogastric Junction
Head and Neck	Mucosal Melanoma of the Head and Neck<br>Cutaneous carcinoma of the Head and Neck<br>Oral Cavity<br>Major Salivary Glands<br>Nasopharynx<br>HPV-Mediated (p16+) Oropharyngeal Cancer<br>Oropharynx (p16-) and Hypopharynx<br>Nasal Cavity and Paranasal Sinuses<br>Larynx
Kidney	Kidney
Liver or Intrahepatic bile duct	Liver<br>Intrahepatic bile ducts
Lung	Lung

CONFIDENTIALITY

This document is the confidential and proprietary information of GRAIL, LLC. Except as required by federal, state, or local laws or regulations, this document may not be disclosed to others, in whole or in part without prior written permission of GRAIL, Inc.

Cancer Type	Cancer Type Description
Myeloid Lineage	Myeloproliferative Neoplasms (Chronic myeloid leukemia,<br>Polycythemia vera,<br>Essential thrombocythemia,<br>Primary myelofibrosis,<br>Chronic neutrophilic leukemia,<br>Chronic eosinophilic leukemia,<br>Juvenile myelomonocytic leukemia,<br>Myeloproliferative neoplasm, NOS)<br>Mastocytosis (Cutaneous mastocytosis, Systemic mastocytosis, Mast cell sarcoma)<br>Myelodysplastic/Myeloproliferative Disorders (Myelodysplastic neoplasms (with defining genetic abnormalities or morphologically defined),<br>Childhood Myelodysplastic Neoplasms,<br>Chronic myelomonocytic leukemia,<br>Myelodysplastic/myeloproliferative neoplasm with neutrophilia,<br>Myelodysplastic/myeloproliferative neoplasm with SF3B1 mutation and thrombocytosis, myelodysplastic/myeloproliferative neoplasm NOS)<br>Acute Myeloid Leukemia (AML) and related neoplasms<br>Dendritic cell and histiocytic neoplasms (Plasmacytoid dendritic cell neoplasms, Langerhans cell and other dendritic neoplasms, Histiocytic neoplasms)
Lymphoid Lineage	B Cell Lymphoid Proliferations and Lymphomas (B-cell predominance,<br>B-cell lymphoblastic leukaemias/lymphomas,<br>Mature B cell,<br>Small Lymphocytic Proliferations,<br>Splenic B-cell lymphomas and leukaemias,<br>Lymphoplasmacytic lymphoma,<br>Marginal zone lymphoma,<br>Follicular Lymphoma,<br>Cutaneous follicle center lymphoma,<br>Mantle cell lymphoma,<br>Transformations of indolent B-cell lymphomas<br>Large B-cell lymphomas,<br>Burkitt lymphoma,<br>Hodgkin Lymphoma,<br>KSHV/HHV8-associated B-cell lymphoid proliferations and lymphomas,<br>Lymphoid proliferations and lymphomas associated with immune deficiency and dysregulation)<br>T Cell and NK Lymphoid Proliferations and Lymphomas (T-cell predominance,<br>T-lymphoblastic leukemia/lymphoma,<br>Mature T-cell and NK-cell neoplasms,<br>Primary cutaneous T-cell lymphomas)<br>Stroma Derived Neoplasms of Lymphoid Tissues (Mesenchymal dendritic cell neoplasms,<br>Myofibroblastic tumor,

CONFIDENTIALITY
This document is the confidential and proprietary information of GRAIL, LLC. Except as required by federal, state, or local laws or regulations, this document may not be disclosed to others, in whole or in part without prior written permission of GRAIL, Inc.

Cancer Type	Cancer Type Description
	Spleen-specific vascular-stromal tumors)
Ovary or Fallopian tubes	Ovary, Fallopian Tube, and Primary Peritoneal Carcinoma
Pancreas, Extrahepatic bile duct or Gallbladder	Gallbladder<br>Perihilar Bile Ducts<br>Distal Bile Duct<br>Exocrine Pancreas<br>Neuroendocrine Tumors of the Pancreas
Plasma Cell Lineage	Plasma Cell Myeloma<br>Solitary plasmacytoma of bone<br>Extraosseous plasmacytoma
Prostate	Prostate
Skin (excluding BCC/SCC)	Merkel Cell Carcinoma<br>Melanoma of the Skin
Stomach	Stomach<br>Neuroendocrine Tumors of the Stomach
Thyroid	Thyroid - Differentiated and Anaplastic Carcinoma<br>Thyroid - Medullary
Uterus	Corpus Uteri - Carcinoma and Carcinosarcoma
Other	Examples include:<br>Brain and Spinal Cord tumors (other than CNS Lymphomas, CNS Sarcomas Mesenchymal, Non-meningothelial Tumors)<br>(Cancer of) Unknown Primary Site<br>Conjunctival Melanoma<br>Uveal Melanoma<br>Parathyroid<br>Ampulla of Vater<br>Neuroendocrine Tumors of the Duodenum and Ampulla of Vater<br>Small Intestine<br>Neuroendocrine Tumors of the Jejunum and Ileum<br>Thymus<br>Malignant Pleural Mesothelioma<br>Vulva<br>Vagina<br>Gestational Trophoblastic Neoplasms<br>Penis<br>Testis<br>Eyelid Carcinoma<br>Conjunctival Carcinoma<br>Retinoblastoma

CONFIDENTIALITY
This document is the confidential and proprietary information of GRAIL, LLC. Except as required by federal, state, or local laws or regulations, this document may not be disclosed to others, in whole or in part without prior written permission of GRAIL, Inc.

Cancer Type	Cancer Type Description
	Lacrimal Gland Carcinoma<br>Adrenal Cortical Carcinoma<br>Adrenal - Neuroendocrine Tumors<br>Prostatic Urethra (Urothelial Carcinomas, Squamous Cell Carcinoma)

CONFIDENTIALITY
This document is the confidential and proprietary information of GRAIL, LLC. Except as required by federal, state, or local laws or regulations, this document may not be disclosed to others, in whole or in part without prior written permission of GRAIL, Inc.

Appendix 9 Sample Size and Power for Bridging Analysis, Stratification Factors, Approximate Sampling and Analysis Weights

The objective of bridging sampling for MCED-I re-testing among true negatives (TN) for MCED-V2 is to assess the false positive (FP) rate (or specificity) for MCED-I, and to ensure that the lower bound of the 95% confidence interval for the estimated MCED-I specificity after sampling will exceed the specificity target of 99% with 90% power.

Other than the 99% specificity threshold, two factors affect power and sample size planning for bridging sampling: the true specificity in the PATHFINDER 2 study population and the degree of overlap for FP results between MCED-V2 and MCED-I. The more sharing of FPs between MCED-V2 and MCED-I, the more MCED-I FPs will be contained in MCED-V2 FPs, and so fewer samples will be needed from the TN for MCED-V2 group to assess MCED-I FPs.

The test design features and the preliminary data from GRAIL suggest that the true specificity of MCED-I in PATHFINDER 2 will be most likely in the range of 99.3% to 99.5%, and the degree of FP sharing is about ½, which means that about half of MCED-V2 FPs will also be FPs for MCED-I. To be conservative in sample size planning, we will assume that about 1/3 of FPs are shared between MCED-V2 and MCED-I.

By selecting approximately 7,500 samples from the PATHFINDER 2 MCED-V2 TN group for MCED-I testing, we will have >90% power to pass the study success criterion target of 99% specificity under the most conservative scenario for true specificity and the amount of FP sharing between MCED-V2 and MCED-I.

As discussed in Section 4.2, the subset selection of participants with negative MCED-V2 test results and no cancer diagnosis during 12 months of follow-up time and completed cancer status assessment at 12 months (i.e., MCED-V2 TN) will be stratified based on the following factors:

1. Age (50-59, 60-69, 70-79, 80+ years)
2. Race (American Indian or Alaska Native; Asian; Black or African American; Native Hawaiian or Other Pacific Islander; White; More than one race, Not reported)
3. Ethnicity (Hispanic or Latino, Not Hispanic or Latino, Missing)
4. Prior cancer history (yes, no)
5. Smoking status (Current smoker, Former Smoker, Non-Smoker)
6. Selected conditions that may affect cfDNA shedding or methylation (as described in Section 14.3.3.2) and which represent relatively small subsets of the PATHFINDER 2 population:
    - 6.1. Those who took any cytotoxic medications in the past year
    - 6.2. Those who took any demethylating agents in the past year
    - 6.3. Participants with solid organ or stem cell (bone marrow) transplant
    - 6.4. HIV Infection
    - 6.5. Pancreatitis
    - 6.6. Chronic lung disease

CONFIDENTIALITY

This document is the confidential and proprietary information of GRAIL, LLC. Except as required by federal, state, or local laws or regulations, this document may not be disclosed to others, in whole or in part without prior written permission of GRAIL, Inc.

The proposed approximate sampling weights for each stratum, defined by the stratification factors, are presented in the table below. The aim of this sampling approach is to ensure sufficient representation of subsets of interest (especially relatively small subsets of the population) important to characterize diversity and representativeness and MCED test performance in terms of FP rate. The final sampling and analysis weights will be defined based on the locked analysis dataset.

Note, that enumeration of all possible combinations of the 11 stratification factors listed above would lead to an unfeasibly large number of strata (>16,000). Therefore, the following approach was applied:

1. Small important subsets were selected sequentially including participants with
    - Cytotoxic medication use
    - Demethylating agent use
    - Transplant
    - HIV Infection
    - Pancreatitis
    - Chronic lung disease
    - Current smoker
    - Those who checked "Native Hawaiian or Pacific Islander"
    - Those who checked "American Indian or Alaska Native"
    - More than one race
2. Cross-tabulation of the remaining factors was examined to identify relatively large subsets which could be downsampled (such as White, Non Hispanic or Latino, No prior cancer history) for each age category.
3. The remaining smaller subsets for each age category were pooled together and upsampled compared to step #2 above.
4. After steps 1, 2 and 3 were implemented, the distribution of all stratification factors was examined to assure sufficient representation.

Stratification Factors, Approximate Sampling and Analysis Weights

Note: strata are defined sequentially and should be implemented in the order in which they appear in the table.

N	Stratum	N (estimated)	Sampling probability	N Sampled (approximate)	Analysis Weight (approximate)
		A		B	A/B
1	Demethylating agents = Yes	17	100%	17	1.0
2	Cytotoxic medications = Yes	375	50%	188	2.0
3	Transplant = Yes	48	100%	48	1.0
4	HIV = Yes	78	100%	78	1.0

CONFIDENTIALITY
This document is the confidential and proprietary information of GRAIL, LLC. Except as required by federal, state, or local laws or regulations, this document may not be disclosed to others, in whole or in part without prior written permission of GRAIL, Inc.

N	Stratum	N (estimated)	Sampling probability	N Sampled (approximate)	Analysis Weight (approximate)
		A		B	A/B
5	Pancreatitis = Yes	234	100%	234	1.0
6	Chronic lung disease = Yes	567	35%	198	2.9
7	Current smoker = Yes	784	60%	470	1.7
8	Native Hawaiian or Other Pacific Islander	89	100%	89	1.0
9	American Indian or Alaska Native	295	100%	295	1.0
10	More than one race	122	100%	122	1.0
11	Age >=80, White, Not Hispanic or Latino, No prior hx	550	75%	412	1.3
12	Age >=80 all others	248	100%	248	1.0
13	Age 70-79, White, Not Hispanic or Latino, No prior hx	4134	15%	620	6.7
14	Age 70-79, White, Not Hispanic or Latino, Prior hx	755	40%	302	2.5
15	Age 70-79, Black, Not Hispanic or Latino, No prior hx	323	40%	129	2.5
16	Age 70-79, Asian, Not Hispanic or Latino, No prior hx	175	40%	70	2.5
17	Age 70-79, White, Hispanic or Latino, No prior hx	161	40%	64	2.5
18	Age 70-79 all others	240	100%	240	1.0
19	Age 60-69, No prior hx, White, Not Hispanic or Latino,	7567	10%	757	10.0
20	Age 60-69, White, Not Hispanic or Latino, Prior hx	762	35%	267	2.9
21	Age 60-69, Black, Not Hispanic or Latino, No prior hx	671	35%	235	2.9
22	Age 60-69, Asian, Not Hispanic or Latino, No prior hx	416	35%	146	2.8
23	Age 60-69, White, Hispanic or Latino, No prior hx	392	35%	137	2.9
24	Age 60-69 all others	447	100%	447	1.0
25	Age 50-59, White, Hispanic or Latino, No prior hx	422	35%	148	2.9
26	Age 50-59, White, Not Hispanic or Latino, No prior hx	3787	15%	568	6.7
27	Age 50-59, White, Not Hispanic or Latino, Prior hx	286	50%	143	2.0
28	Age 50-59, Black, Not Hispanic or Latino, No prior hx	592	35%	207	2.9
29	Age 50-59, Asian, Not Hispanic or Latino, No prior hx	667	35%	233	2.9
30	Age 50-59 all others	475	100%	475	1.0

CONFIDENTIALITY
This document is the confidential and proprietary information of GRAIL, LLC. Except as required by federal, state, or local laws or regulations, this document may not be disclosed to others, in whole or in part without prior written permission of GRAIL, Inc.

Appendix 10 Examples of invasive procedures

The National Cancer Institute definition states that invasive procedures are medical procedures which invade (enter) the body, usually by cutting or puncturing the skin or by inserting instruments into the body.[37]

Invasive procedures include surgical and non-surgical procedures. Surgical procedures are associated with the greatest risk of developing complications and involve a structural alteration of the human body and/or incision or destruction of tissues using instruments.[38]

Type of Diagnostic Testing	Category	Examples of specific procedures
Surgical Procedure	All	Laparoscopy
		Thoracoscopy
		Mediastinoscopy
		Thoracotomy
		Laparotomy
Non-Surgical Procedure	Endoscopy	Anoscopy
		Colonoscopy
		Sigmoidoscopy
		Esophagogastroduodenoscopy (EGD)
		Endoscopic Ultrasound
		Endoscopic retrograde cholangiopancreatography (ERCP)
		Bronchoscopy
		Cystoscopy
	Biopsy	Fine needle aspiration biopsy
		Core needle biopsy
		Bone marrow biopsy
		Incisional biopsy
		Excisional biopsy (of skin)
	Other	Pap smear
		Paracentesis
		Thoracocentesis

37 https://www.cancer.gov/search/results?swKeyword=invasive+procedure

38 This language is consistent with the SNOMED definition of surgical procedure.

CONFIDENTIALITY

This document is the confidential and proprietary information of GRAIL, LLC. Except as required by federal, state, or local laws or regulations, this document may not be disclosed to others, in whole or in part without prior written permission of GRAIL, Inc.

Appendix 11 Recommended diagnostic evaluations by CSO prediction

MCED-V2 CSO prediction	V2-based Predicted Cancer(s)	Expected Diagnostic Evaluation	Type of Diagnostic Evaluation
Anus	Anus	Anoscopy or sigmoidoscopy	Endoscopy
Bladder, Urothelial tract	Bladder, Renal Pelvis, Ureter, Urethra	Flexible cystoscopy and CT urogram or retrograde pyelogram with contrast	Endoscopy<br><br>Imaging
Bone and Soft Tissue	Skeletal Muscle and Other Connective Tissue, Vascular Tissue, Bone and Cartilage	MRI with contrast or CT of chest, abdomen, pelvis or PET-CT	Imaging
Breast	Breast	Diagnostic mammography<br><br>MRI if participant has recently had screening mammogram	Imaging
Cervix	Cervix	Cytology (i.e., Pap smear), HPV testing, colposcopy	Other non-surgical procedure<br><br>Endoscopy
Colon, Rectum	Colon, Rectum, Appendix	Colonoscopy	Endoscopy
Head and Neck	Oropharynx, Hypopharynx, Nasopharynx, Larynx, Lip and Oral Cavity (including Oral tongue), Nasal Cavity, Paranasal Sinuses, Major Salivary glands	Flexible nasal endoscopy and Head and Neck CT or MRI	Endoscopy<br><br>Imaging
Kidney	Kidney	Triple phase renal CT	Imaging
Liver, Bile duct	Liver, Intrahepatic bile duct	Triple phase abdominal CT, AFP	Imaging<br><br>Lab test
Lung	Lung, Bronchus	Chest CT<br><br>PET-CT	Imaging

CONFIDENTIALITY
This document is the confidential and proprietary information of GRAIL, LLC. Except as required by federal, state, or local laws or regulations, this document may not be disclosed to others, in whole or in part without prior written permission of GRAIL, Inc.

MCED-V2 CSO prediction	V2-based Predicted Cancer(s)	Expected Diagnostic Evaluation	Type of Diagnostic Evaluation
Lymphoid Lineage	Lymphoid leukemia<br><br>Lymphoma	CBC with differential, peripheral blood smear, LDH, Hepatitis B and C serology, comprehensive metabolic panel (CMP)<br><br>Bone marrow biopsy after blood tests if needed<br><br>CT of chest, abdomen, pelvis with contrast or PET-CT if needed	Lab test<br><br>Biopsy<br><br>Imaging
Melanocytic Lineage	Melanoma	Full body skin exam	Non-invasive procedure
Myeloid Lineage	Myeloid neoplasm	CBC with differential, peripheral blood smear, LDH, uric acid, CMP<br><br>Bone marrow biopsy after blood tests if needed	Lab test<br><br>Biopsy
Neuroendocrine Cells of Lung or other Organs	Neoplasms arising from cells of neuroendocrine lineage in the lung and high-grade cancer arising from neuroendocrine lineage in all body regions	CT of chest with or without contrast, CT of abdomen, pelvis, head & neck	Imaging
Ovary	Ovary, Fallopian tube, Primary Peritoneum	Transvaginal ultrasound and CA-125	Imaging<br><br>Lab test
Pancreas, Gallbladder	Pancreas, Extrahepatic bile duct, Gallbladder	Pancreas CT protocol or MRCP	Imaging
Plasma Cell Lineage	Plasma cell neoplasm	CBC with differential, peripheral blood smear, LDH, uric acid, beta-2-microglobulin, creatinine clearance, serum immunoglobulins, serum free light chain assay, serum and urine protein electrophoresis, CMP<br><br>Bone marrow biopsy after blood and urine tests if needed<br><br>Whole-body low-dose CT or PET-CT if needed	Lab test<br><br>Biopsy<br><br>Imaging

CONFIDENTIALITY
This document is the confidential and proprietary information of GRAIL, LLC. Except as required by federal, state, or local laws or regulations, this document may not be disclosed to others, in whole or in part without prior written permission of GRAIL, Inc.

MCED-V2 CSO prediction	V2-based Predicted Cancer(s)	Expected Diagnostic Evaluation	Type of Diagnostic Evaluation
Prostate	Prostate	PSA and multi-parametric MRI of the prostate	Imaging<br><br>Lab test
Stomach, Esophagus (upper GI)	Stomach, Esophagus	Upper endoscopy	Endoscopy
Thyroid Gland	Thyroid Gland	Ultrasound of thyroid	Imaging
Uterus	Uterus	Transvaginal ultrasound	Imaging

CONFIDENTIALITY
This document is the confidential and proprietary information of GRAIL, LLC. Except as required by federal, state, or local laws or regulations, this document may not be disclosed to others, in whole or in part without prior written permission of GRAIL, Inc.

APPENDIX 12 USPSTF CANCER SCREENING RECOMMENDATIONS

Cancer	Grade	Eligible population	Cancer screening recommendations
Breast[39]	B	Women 40-74 years	Mammography or digital breast tomosynthesis every 2 years
Cervix[40]	A	Women 21-65 years	• Pap smear (cervical cytology) every 3 years<br>• Pap smear with high-risk HPV testing every 5 years<br>• High-risk HPV testing alone every 5 years
Colorectal[41]	A	Adults 50-75 years	• High-sensitivity gFOBT or FIT every year<br>• sDNA-FIT every 1-3 years<br>• CT colonography every 5 years<br>• Flexible sigmoidoscopy every 5 years<br>• Flexible sigmoidoscopy every 10 years plus FIT every year<br>• Colonoscopy every 10 years
	B	Adults 45-49 years	
	C	Adults 76-85 years	Selective screening offered on an individual basis based on health status
Lung[42]	B	Adults 50-80 years with ≥20 pack-year smoking history who currently smoke or have quit within the past 15 years	Annual low-dose computed tomography (LDCT)
Prostate[43]	C	Men 55-69 years	Individual decision for periodic PSA-based screening after discussion of benefits and harms with clinician

Note: Expanded breast cancer screening includes MRI[44] or ultrasound in addition to tests described in the table, and will be included as a separate category in the analysis

[39] https://www.uspreventiveservicestaskforce.org/uspstf/recommendation/breast-cancer-screening
[40] https://www.uspreventiveservicestaskforce.org/uspstf/recommendation/cervical-cancer-screening
[41] https://www.uspreventiveservicestaskforce.org/uspstf/recommendation/colorectal-cancer-screening
[42] https://www.uspreventiveservicestaskforce.org/uspstf/recommendation/lung-cancer-screening
[43] https://www.uspreventiveservicestaskforce.org/uspstf/recommendation/prostate-cancer-screening
[44] Monticciolo D.L. et al, Breast Cancer Screening for Women at Higher-Than-Average Risk: Updated Recommendations From the ACR, Clinical Practice Management, 2023, Volume 20, Issue 9, p902-914.

CONFIDENTIALITY

This document is the confidential and proprietary information of GRAIL, LLC. Except as required by federal, state, or local laws or regulations, this document may not be disclosed to others, in whole or in part without prior written permission of GRAIL, Inc.

Appendix 13 Description of Participant-Directed Questionnaires.

State-Trait Anxiety Inventory (STAI)

The STAI is one of the most widely used measures of anxiety. The instrument is scored on a scale of 20-80, with higher scores indicating greater anxiety The abbreviated version contains six items and four choices (1 = not at all, 2 = somewhat, 3 = moderately, and 4 = very much so) that reflect common symptoms of anxiety experienced by an individual. When calculating the total summed STAI score, the scores on the three positively worded items (calm, relaxed, content) will be reverse-coded, so that a higher score corresponds to a higher level of individual anxiety.

STAI-6 is a simplified version of STAI-20, and the total summed scores were prorated (multiplied by 20/6) to obtain scores that were comparable to those from the full 20-item STAI (Knight et al., 1983; Marteau and Bekker, 1992).

Figure. STAI-6

A number of statements which people have used to describe themselves are given on the following page. Read each statement and select the appropriate response to indicate how you feel right now, that is, at this very moment. There are no right or wrong answers. Do not spend too much time on any one statement but give the answer which seems to describe your present feelings best.				
Statement	Not at all	Somewhat	Moderately	Very much
I feel calm	1	2	3	4
I feel tense	1	2	3	4
I feel upset	1	2	3	4
I feel relaxed	1	2	3	4
I feel content	1	2	3	4
I am worried	1	2	3	4

MCED STAI is a questionnaire with 3 items modeled after the corresponding items for STAI-6 with the same four choices (1 = not at all, 2 = somewhat, 3 = moderately, and 4 = very much so):

1. I am tense because of my multi-cancer early detection test
2. I feel upset because of my multi-cancer early detection test
3. I am worried because of my multi-cancer early detection test

Adapted MICRA

CONFIDENTIALITY
This document is the confidential and proprietary information of GRAIL, LLC. Except as required by federal, state, or local laws or regulations, this document may not be disclosed to others, in whole or in part without prior written permission of GRAIL, Inc.

The Multidimensional Impact of Cancer Risk Assessment (MICRA) is a previously validated instrument that appropriately assessed different levels of impact of genetic test result disclosure. The MICRA was adapted with permission to measure impacts of MCED result disclosure and is composed of 21 items addressing 3 domains (distress, uncertainty, positive experience) and a total score; four conditional items from the original MICRA relating to parenthood and prior cancer were excluded. Higher scores indicate increased distress, uncertainty, and less positive experience.

Overview of scales and items of the adapted MICRA

Adapted MICRA Scale	Number of items	Scale range	Item numbers
Distress	6	0-30	1-4, 7-8
Uncertainty	9	0-45	9-12, 14-17, 20
Positive experience	4	0-20	5-6, 18-19
Total score	19	0-95	1-12, 14-20

The MICRA questionnaire consists of three subscales, a Positive Experience subscale (4 items), a Distress subscale (6 items), and an Uncertainty subscale (9 items). The questionnaire asks the respondents to indicate whether they have experienced each statement on a four-point scale (0, 1, 3, 5) in the past week. A response of 0 indicated never experiencing the statement while a 5 indicated often experiencing the statement. A higher score on any of the subscales or the total scale indicated greater psychological distress. The positive subscale is reverse scored to reflect this. The scoring of the adapted MICRA is based on the FACIT group's MICRA scoring manual.

SF-12v2

The Short Form-12 version 2 Health Survey (SF-12v2) is a 12-item, multipurpose, short-form health survey that was developed to be a brief, broad measure of eight domains of health status. The SF-12v2® Health Survey yields an eight-scale profile of functional health and wellbeing, as well as two psychometrically based physical and mental health summary measures and a preference-based health utility index.

The SF-12v2 is a generic measure of health and does not target a specific age, disease, or treatment group and has been in surveys of general and specific populations, in comparing the relative burden of diseases, and in differentiating the health benefits produced by a wide range of treatments. However, it is not intended to be a comprehensive survey of health.

The SF-12v2 includes eight health domains: physical functioning, role participation with physical health problems (role physical), bodily pain, general health, vitality, social functioning (i.e., limitations in social functioning), role participation with emotional health problems (role-emotional), and mental health. The component summary measures reflect two broad components of health: physical and mental. All eight health domain scales are used to score both component summary measures. Higher scores indicate better health.

CONFIDENTIALITY
This document is the confidential and proprietary information of GRAIL, LLC. Except as required by federal, state, or local laws or regulations, this document may not be disclosed to others, in whole or in part without prior written permission of GRAIL, Inc.

The scoring of the SF-12v2 is based on QualityMetric Incorporated SF12v2 Administration guide (Ware, J. E., Jr., Kosinski, M., Turner-Bowker, D. M., Sundaram, M., Gandek, B., & Maruish, M. E. (2009). SF-12v2 Health Survey: Administration guide for clinical trial investigators. Lincoln, RI: QualityMetric Incorporated). Higher scores represent better health.

Satisfaction with multi-cancer early-detection test

To assess satisfaction with the MCED test, a 3-item questionnaire was developed by the study investigators. Each question included response options reflecting 5- or 7-point Likert scales, with total satisfaction scores ranging from 0 to 100 with higher scores representing higher satisfaction.

The following questions were included in the satisfaction questionnaire:

1. Overall, how confident are you that this multi-cancer early detection test is a good thing for you?
2. How certain are you that the good things about the multi-cancer early detection test outweigh the bad things?
3. Taking all things into account, how satisfied or dissatisfied are you with the multi-cancer early detection test?

CONFIDENTIALITY
This document is the confidential and proprietary information of GRAIL, LLC. Except as required by federal, state, or local laws or regulations, this document may not be disclosed to others, in whole or in part without prior written permission of GRAIL, Inc.

Appendix 14 Estimated effective radiation dose estimates for imaging tests.

Imaging test	Estimated effective dose (milliSieverts)
Barium enema	8.0
LDCT: lung cancer screening	1.2
LDCT: whole body	4.8
CT Abdomen	8.0
CT Abdomen and pelvis	10.5
CT Abdomen and pelvis with and without contrast	17.4
CT Abdomen and pelvis - multiphase	22.6
CT Brain	1.8
CT Chest	6.6
CT Chest with contrast	17.0
CT Head	2.7
CT Head and neck	8.0
CT Liver (triple phase)	15.0
CT Neck	6.8
CT Pancreas protocol	23.7
CT Pelvis	6.0
CT Spine	19.9
CT Cervical spine	2.7
CT Thoracic spine	13.1
CT Lumbar spine	11.2
CT Triple phase renal	29.4
CT Urogram	6.1
CT Whole body	15.4
Dual x-ray absorptiometry (with CT)	0.04
Dual x-ray absorptiometry (without CT)	0.001
Endoscopic retrograde cholangiopancreatography	4.0
Intravenous urography	3.0

CONFIDENTIALITY
This document is the confidential and proprietary information of GRAIL, LLC. Except as required by federal, state, or local laws or regulations, this document may not be disclosed to others, in whole or in part without prior written permission of GRAIL, Inc.

Imaging test	Estimated effective dose (milliSieverts)
Mammography	0.4
Octreotide scan	12.0
PET/CT (18F-FDG)	14.1
Retrograde pyelogram	7.7
Screening Digital Breast Tomosynthesis	0.3
Screening Digital Mammography	0.2
Small-bowel series	5.0
Upper gastrointestinal series	6.0
X-ray Abdomen	0.7
X-ray Cervical spine	0.2
X-ray Chest	0.1
X-ray Hip	0.7
X-ray Knee	0.005
X-ray Lumbar spine	1.5
X-ray Other extremities	0.001
X-ray Pelvis	0.6
X-ray Posteroanterior and lateral study of chest	0.1
X-ray Posteroanterior study of chest	0.02
X-ray Shoulder	0.01
X-ray Skull	0.1
X-ray Thoracic spine	1.0

Effective radiation dose estimates were obtained from several sources. If more than one dose estimate was available, the estimates were chosen in order from the sources listed below:

1. University of California, San Francisco International CT Dose Registry. https://radiology.ucsf.edu/blog/uc-dose-project-and-recent-research-call-standard-protocols-ct-scan-doses. Estimates provided to GRAIL on 26 April 2024.
2. Mettler FA Jr, Huda W, Yoshizumi TT, Mahesh M. Effective doses in radiology and diagnostic nuclear medicine: a catalog. Radiology. 2008 Jul;248(1):254-63. doi: 10.1148/radiol.2481071451. PMID: 18566177.
3. Smith-Bindman R, Lipson J, Marcus R, et al. Radiation Dose Associated With Common Computed Tomography Examinations and the Associated Lifetime Attributable Risk of

CONFIDENTIALITY
This document is the confidential and proprietary information of GRAIL, LLC. Except as required by federal, state, or local laws or regulations, this document may not be disclosed to others, in whole or in part without prior written permission of GRAIL, Inc.

Cancer. *Arch Intern Med.* 2009;169(22):2078–2086. doi:10.1001/archinternmed.2009.427

4. Hemke R, Yang K, Husseini J, Bredella MA, Simeone FJ. Organ dose and total effective dose of whole-body CT in multiple myeloma patients. Skeletal Radiol. 2020 Apr;49(4):549-554. doi: 10.1007/s00256-019-03292-z. Epub 2019 Oct 15. PMID: 31612246; PMCID: PMC7021660.
5. Lipkin ME, Mancini JG, Zilberman DE et al. Reduced radiation exposure with the use of an air retrograde pyelogram during fluoroscopic access for percutaneous nephrolithotomy. J Endourol. 2011 Apr;25(4):563-7. doi: 10.1089/end.2010.0431. Epub 2011 Mar 22. PMID: 21426236.
6. van der Molen AJ, Miclea RL, Geleijns J, Joemai RMS. A Survey of Radiation Doses in CT Urography Before and After Implementation of Iterative Reconstruction. American Journal of Roentgenology 2015 205:3, 572-577
7. Quinn, B, Dauer, Z, Pandit-Taskar, N *et al.* Radiation dosimetry of 18F-FDG PET/CT: incorporating exam-specific parameters in dose estimates. BMC Med Imaging 16, 41 (2016). https://doi.org/10.1186/s12880-016-0143-y

CONFIDENTIALITY

This document is the confidential and proprietary information of GRAIL, LLC. Except as required by federal, state, or local laws or regulations, this document may not be disclosed to others, in whole or in part without prior written permission of GRAIL, Inc.
