# Supplementary material for: Performance and safety of a multi-cancer early detection test: the PATHFINDER 2 study
Source: Nat Med. 2026 Sep 22;32(10):3760–9. doi: 10.1038/s41591-026-04618-w (PMC13668646; doi:10.1038/s41591-026-04618-w)
Supplement: Supplementary file 2 [file 41591_2026_4618_MOESM2_ESM.pdf]

nature portfolio

Corresponding author(s): Nima Nabavizadeh

Last updated by author(s): Jul 15, 2026



# Reporting Summary

Nature Portfolio wishes to improve the reproducibility of the work that we publish. This form provides structure for consistency and transparency in reporting. For further information on Nature Portfolio policies, see our Editorial Policies and the Editorial Policy Checklist.

## Statistics

For all statistical analyses, confirm that the following items are present in the figure legend, table legend, main text, or Methods section.

| n/a | Confirmed | |
|---|---|---|
| ☐ | ☒ | The exact sample size (*n*) for each experimental group/condition, given as a discrete number and unit of measurement |
| ☒ | ☐ | A statement on whether measurements were taken from distinct samples or whether the same sample was measured repeatedly |
| ☒ | ☐ | The statistical test(s) used AND whether they are one- or two-sided<br>*Only common tests should be described solely by name; describe more complex techniques in the Methods section.* |
| ☒ | ☐ | A description of all covariates tested |
| ☒ | ☐ | A description of any assumptions or corrections, such as tests of normality and adjustment for multiple comparisons |
| ☐ | ☒ | A full description of the statistical parameters including central tendency (e.g. means) or other basic estimates (e.g. regression coefficient) AND variation (e.g. standard deviation) or associated estimates of uncertainty (e.g. confidence intervals) |
| ☒ | ☐ | For null hypothesis testing, the test statistic (e.g. *F*, *t*, *r*) with confidence intervals, effect sizes, degrees of freedom and *P* value noted<br>*Give P values as exact values whenever suitable.* |
| ☒ | ☐ | For Bayesian analysis, information on the choice of priors and Markov chain Monte Carlo settings |
| ☒ | ☐ | For hierarchical and complex designs, identification of the appropriate level for tests and full reporting of outcomes |
| ☒ | ☐ | Estimates of effect sizes (e.g. Cohen's *d*, Pearson's *r*), indicating how they were calculated |

*Our web collection on statistics for biologists contains articles on many of the points above.*

## Software and code

Policy information about availability of computer code

| | |
|---|---|
| Data collection | Clinical data were entered into and stored in the electronic case report forms within Medrio, a validated 21CFR Part 11 compliant electronic data capture system. |
| Data analysis | R version 4.3.2 was used for analysis. |

For manuscripts utilizing custom algorithms or software that are central to the research but not yet described in published literature, software must be made available to editors and reviewers. We strongly encourage code deposition in a community repository (e.g. GitHub). See the Nature Portfolio guidelines for submitting code & software for further information.

## Data

Policy information about availability of data

All manuscripts must include a data availability statement. This statement should provide the following information, where applicable:

- Accession codes, unique identifiers, or web links for publicly available datasets
- A description of any restrictions on data availability
- For clinical datasets or third party data, please ensure that the statement adheres to our policy

Anonymized participant data will be available following the completion of the 3-year follow-up and database lock (anticipated December 2027). Requests for access to anonymized data may be emailed with a proposal to nabaviza@ohsu.edu. Investigators will assess proposals for scientific merit. Proposals should have a clearly specified research question supported by a methodologically sound analysis proposal and a prespecified statistical analysis plan, together with documentation of

the requesting investigators' qualifications and any applicable ethics and data-protection approvals. Data will be provided after approval of the proposal and completion of a data transfer agreement. Decisions regarding the proposals will be communicated within 4-6 weeks.

## Research involving human participants, their data, or biological material

Policy information about studies with human participants or human data. See also policy information about sex, gender (identity/presentation), and sexual orientation and race, ethnicity and racism.

| | |
|---|---|
| Reporting on sex and gender | Distribution of biological sex is provided in Table 1. Secondary analysis of data stratified by sex is included in this manuscript (Supplemental Table S11). Enrollment targets for sex distribution were specified in the protocol to ensure a representative study population. The sample size of this descriptive study was driven by the ability to enroll participants in the specific study subgroups based on age and sex to obtain information on diagnostic evaluation triggered by the MCED test result. Sex was determined by the participants. |
| Reporting on race, ethnicity, or other socially relevant groupings | Distribution of race and ethnicity is included in the analysis (Table 1). Secondary analyses of data stratified by race and ethnicity are not included in the manuscript. Categories aligned with FDA guidance. Race and ethnicity identities were determined by the participants. |
| Population characteristics | Distribution of demographic and baseline characteristics, such as age, sex, race/ethnicity, education, smoking status, and prior cancer history is provided in Table 1. Briefly, The analysis population comprised 35,350 analyzable participants aged ≥50 years without clinical suspicion of cancer who had not been diagnosed with or treated for cancer within 3 years prior to enrollment. As shown in Table 1, the median age was 64.0 years (Q1-Q3, 58.0-70.0), and participants were 56.2% female and 43.8% male. By self-reported race, 80.1% were White, 8.8% Black or African American, 5.9% Asian, 0.7% American Indian or Alaska Native, 0.2% Native Hawaiian or other Pacific Islander, 1.3% multiple races, and 2.9% missing. By self-reported ethnicity, 91.3% were Not Hispanic or Latino, 7.4% Hispanic or Latino, and 1.3% missing.<br><br>A higher percentage of participants reported characteristics associated with better health status compared with data from the United States National Health and Nutrition Examination Survey, consistent with what has been observed in other cancer screening trials. Such participants tend to follow existing screening recommendations and hence the number of cancers with existing screening options detected during the study may be lower. |
| Recruitment | Participants were recruited at clinical sites via messages through the electronic health record, at health fairs, and through promotional materials in hospitals and clinics. |
| Ethics oversight | The study was performed in accordance with the Declaration of Helsinki. All study procedures were approved by a central or local Institutional Review Board. All participants provided written informed consent.<br>The Institutional Review Boards that approved the protocol were: Cleveland Clinic IRB, Duke Health Institutional Review Board, Advarra Institutional Review Board, WCG IRB, HFHS Institutional Review Board, USOR IRB, Mayo Clinic Institutional Review Boards, Ochsner Clinic Foundation IRB, OHSU IRB #3, Sutter Health IRB, University Health Network Research Ethics Board, UIC Research, and Biomedical Research Alliance of New York IRB. |

Note that full information on the approval of the study protocol must also be provided in the manuscript.

# Field-specific reporting

Please select the one below that is the best fit for your research. If you are not sure, read the appropriate sections before making your selection.

☒ Life sciences ☐ Behavioural & social sciences ☐ Ecological, evolutionary & environmental sciences

For a reference copy of the document with all sections, see nature.com/documents/nr-reporting-summary-flat.pdf

# Life sciences study design

All studies must disclose on these points even when the disclosure is negative.

| | |
|---|---|
| Sample size | N=35,878. The sample size of this descriptive study was driven by the ability to enroll participants in the specific study subgroups based on age and sex to obtain information on diagnostic evaluation triggered by the MCED test result. The study was designed to enroll ≥35,000 participants. Assuming that approximately 5% of participants would be excluded due to various clinical and assay evaluability reasons, approximately 33,250 analyzable participants were expected for analyses related to receiving MCED test results and associated diagnostic evaluation. As described in the section 'Sample Size and Power,' a study of this size was expected to yield at least 370 cancers and 300 MCED positive results, which would provide sufficient precision for estimating test performance and safety of the MCED.<br><br>No adjustments for covariates were prespecified in the Statistical Analysis Plan. As part of secondary study objectives, MCED test performance was evaluated in selected demographic and clinical subgroups (Supplemental Tables S10, S11, S12). |
| Data exclusions | Data from participants without cleaned data, from participants who were not clinically eligible or evaluable, and from participants who did not have a valid MCED test result were excluded. |
| Replication | Study was not designed as a replication study. |

| | |
|---|---|
| Randomization | Study was not randomized. |
| Blinding | Study was not randomized so blinding did not occur. |

# Reporting for specific materials, systems and methods

We require information from authors about some types of materials, experimental systems and methods used in many studies. Here, indicate whether each material, system or method listed is relevant to your study. If you are not sure if a list item applies to your research, read the appropriate section before selecting a response.

## Materials & experimental systems

| n/a | Involved in the study | |
|---|---|---|
| ☒ | ☐ | Antibodies |
| ☒ | ☐ | Eukaryotic cell lines |
| ☒ | ☐ | Palaeontology and archaeology |
| ☒ | ☐ | Animals and other organisms |
| ☐ | ☒ | Clinical data |
| ☒ | ☐ | Dual use research of concern |
| ☒ | ☐ | Plants |

## Methods

| n/a | Involved in the study | |
|---|---|---|
| ☒ | ☐ | ChIP-seq |
| ☒ | ☐ | Flow cytometry |
| ☒ | ☐ | MRI-based neuroimaging |

## Clinical data

Policy information about clinical studies

All manuscripts should comply with the ICMJE guidelines for publication of clinical research and a completed CONSORT checklist must be included with all submissions.

| | |
|---|---|
| Clinical trial registration | NCT05155605 |
| Study protocol | Study protocol and statistical analysis plan will be published as Supplemental Information with the manuscript. |
| Data collection | The 32 clinical sites in US and Canada are detailed in Supplemental Table S19. Enrollment dates were 08 December 2021 (first participant in) to 10 July 2024 (last participant in). Data collected for the first 12 months of follow-up were included in this analysis. Data lock for this analysis was 11 February 2026. Data were collected by clinical site staff. Clinical data were collected from the blood collection tube, test requisition form, test result report, participants' medical records and self-reported information, and participant questionnaires. Data collection was monitored by a contract research organization, PPD/Thermo Fisher. |
| Outcomes | Primary and secondary endpoints were predefined in the study protocol and statistical analysis plan. A detailed description of all study endpoints is provided in Supplemental Table S23. The clinical outcome of cancer was defined as a diagnosis of an invasive solid tumor, excluding nonmetastatic basal cell carcinoma and squamous cell carcinoma of the skin, or hematologic malignancy. |

## Plants

| | |
|---|---|
| Seed stocks | *Report on the source of all seed stocks or other plant material used. If applicable, state the seed stock centre and catalogue number. If plant specimens were collected from the field, describe the collection location, date and sampling procedures.* |
| Novel plant genotypes | *Describe the methods by which all novel plant genotypes were produced. This includes those generated by transgenic approaches, gene editing, chemical/radiation-based mutagenesis and hybridization. For transgenic lines, describe the transformation method, the number of independent lines analyzed and the generation upon which experiments were performed. For gene-edited lines, describe the editor used, the endogenous sequence targeted for editing, the targeting guide RNA sequence (if applicable) and how the editor was applied.* |
| Authentication | *Describe any authentication procedures for each seed stock used or novel genotype generated. Describe any experiments used to assess the effect of a mutation and, where applicable, how potential secondary effects (e.g. second site T-DNA insertions, mosiacism, off-target gene editing) were examined.* |
